# Ancient DNA reveals the prehistory of the Uralic and Yeniseian peoples

**DOI:** 10.1038/s41586-025-09189-3

**Published:** 2025-07-02

**Authors:** Tian Chen Zeng, Leonid A. Vyazov, Alexander Kim, Pavel Flegontov, Kendra Sirak, Robert Maier, Iosif Lazaridis, Ali Akbari, Michael Frachetti, Alexey A. Tishkin, Natalia E. Ryabogina, Sergey A. Agapov, Danila S. Agapov, Anatoliy N. Alekseev, Gennady G. Boeskorov, Anatoly P. Derevianko, Viktor M. Dyakonov, Dmitry N. Enshin, Alexey V. Fribus, Yaroslav V. Frolov, Sergey P. Grushin, Alexander A. Khokhlov, Kirill Yu. Kiryushin, Yurii F. Kiryushin, Egor P. Kitov, Pavel Kosintsev, Igor V. Kovtun, Nikolai P. Makarov, Viktor V. Morozov, Egor N. Nikolaev, Marina P. Rykun, Tatyana M. Savenkova, Marina V. Shchelchkova, Vladimir Shirokov, Svetlana N. Skochina, Olga S. Sherstobitova, Sergey M. Slepchenko, Konstantin N. Solodovnikov, Elena N. Solovyova, Aleksandr D. Stepanov, Aleksei A. Timoshchenko, Aleksandr S. Vdovin, Anton V. Vybornov, Elena V. Balanovska, Stanislav Dryomov, Garrett Hellenthal, Kenneth Kidd, Johannes Krause, Elena Starikovskaya, Rem Sukernik, Tatiana Tatarinova, Mark G. Thomas, Maxat Zhabagin, Kim Callan, Olivia Cheronet, Daniel Fernandes, Denise Keating, Candilio Francesca, Lora Iliev, Aisling Kearns, Kadir Toykan Özdoğan, Matthew Mah, Adam Micco, Megan Michel, Iñigo Olalde, Fatma Zalzala, Swapan Mallick, Nadin Rohland, Ron Pinhasi, Vagheesh M. Narasimhan, David Reich

**Affiliations:** 1 Department of Human Evolutionary Biology, Harvard University, Cambridge, MA, USA; 2 Department of Biology and Ecology, Faculty of Science, University of Ostrava, Ostrava, Czechia; 3 Department of Genetics, Harvard Medical School, Boston, MA, USA.; 4 Department of Anthropology, Harvard University, Cambridge, MA, USA.; 5 Institute of Parasitology, Biology Centre CAS, České Budějovice, Czech Republic; 6 Howard Hughes Medical Institute, Harvard Medical School, Boston, MA, USA.; 7 Department of Anthropology, Washington University in St. Louis, St. Louis, MO, USA.; 8 School of Cultural Heritage, Northwest University, Xi’an, Shaanxi Province, China.; 9 Department of Archaeology, Ethnography and Museology, Altai State University, Barnaul, Russia; 10 Department of Historical Studies, University of Gothenburg, Gothenburg, Sweden; 11 Historical, Ecological and Cultural Association “Povolzhye”, Samara, Russia; 12 Institute of Studies the Humanities and Problems of Indigenous People of the North, FIC Yakutsk Scientific Center of the Siberian Branch of the Russian Academy of Sciences, Yakutsk, Russia; 13 Diamond and Precious Metals Geology Institute, Siberian Branch of the Russian Academy of Sciences, Yakutsk, Russia; 14 Institute of Archaeology and Ethnography, Siberian Branch of the Russian Academy of Sciences, Novosibirsk, Russia; 15 Tyumen Scientific Center of the Siberian Branch of Russian Academy of Sciences, Institute of Problems of Northern Development, Tyumen, Russia; 16 Department of Archaeology of Central Asia and the Caucasus, Institute for the History of Material Culture of Russian Academy of Science, Saint-Petersburg, Russia; 17 Department of Archaeology, Kemerovo State University, Kemerovo, Russia; 18 Samara State University of Social Sciences and Education, Samara, Russia; 19 Department of Recreational Geography, Service, Tourism and Hospitality, Institute of Geography, Altai State University, Barnaul, Russia; 20 Center of Human Ecology, Institute of Ethnology and Anthropology, Russian Academy of Science, Moscow, Russia; 21 Institute of Plant and Animal Ecology, Ural Branch of the Russian Academy of Sciences; 22 Ural Federal University, Ekaterinburg, Russia; 23 Independent Researcher, Kemerovo, Russia; 24 Polikarpovich Krasnoyarsk Regional Museum of Local Lore, Krasnoyarsk, Russia; 25 LLC “Archaeology of the East European Plain”, Moscow, Russia; 26 National Research Tomsk State University, Tomsk Russia; 27 V.F. Voino-Yasenetsky Krasnoyarsk State Medical University, Krasnoyarsk, Russia; 28 Institute of Natural Sciences, M.K. Ammosov North-Eastern Federal University, Yakutsk, Russia; 29 Institute of History and Archaeology, Ural Branch of the Russian Academy of Sciences, Ekaterinburg, Russia; 30 Siberian State University of Physical Education and Sport, Omsk, Russia; 31 Arctic research center of Sakha Republic, Yakutia, Russia; 32 Human Population Genetics Laboratory, Research Center for Medical Genetics, Moscow, Russia; 33 Laboratory of Archaeogenetics, Nazarbayev University, Astana, Kazakhstan; 34 Laboratory of Human Genetics, National Center for Biotechnology, Astana, Kazakhstan; 35 Department of Genetics, Evolution and Environment, University College London Genetics Institute (UGI), University College London, London, UK.; 36 Department of Genetics, Yale Medical School, New Haven, CT, USA.; 37 Department of Archaeogenetics, Max Planck Institute for Evolutionary Anthropology, Leipzig, Germany; 38 Department of Biology, University of La Verne, La Verne, CA, USA; 39 Laboratory of Human Molecular Genetics, Institute of Molecular and Cellular Biology, Siberian Branch of the Russian Academy of Sciences (SBRAS), Novosibirsk, Russia; 40 Human Evolution and Archaeological Sciences, University of Vienna, Vienna, Austria.; 41 Department of Evolutionary Anthropology, University of Vienna, Vienna, Austria; 42 CIAS, Department of Life Sciences, University of Coimbra, Coimbra, Portugal; 43 School of Archaeology, University College Dublin, Ireland; 44 Servizio di Bioarcheologia, Museo delle Civiltà, Rome, Italy.; 45 Utrecht University, Utrecht, Netherlands; 46 BIOMICs Research Group, Department of Zoology and Animal Cell Biology, University of the Basque Country UPV/EHU, Spain.; 47 Ikerbasque-Basque Foundation of Science, Bilbao, Spain.; 48 Department of Integrative Biology, University of Texas, Austin, Texas, USA; 49 Department of Statistics and Data Science, University of Texas, Austin, Texas, USA; 50 Broad Institute of MIT and Harvard, Cambridge, MA, USA

## Abstract

The North Eurasian forest and forest-steppe zones have sustained millennia of sociocultural connections among northern peoples, but much of their history is poorly understood. In particular, the genomic formation of populations that speak Uralic and Yeniseian languages today is unknown. By generating genome-wide data for 180 ancient individuals spanning this region, we show the Early to Mid-Holocene hunter-gatherers harbored a continuous gradient of ancestry from fully European-related in the Baltic, to fully East Asian-related in the Transbaikal. Contemporaneous groups in Northeast Siberia were off-gradient, and descended from a population that was the primary source for Native Americans, which then mixed with populations of Inland East Asia and the Amur River Basin to produce two populations whose expansion coincided with the collapse of pre-Bronze Age population structure. Ancestry from the first population, Cis-Baikal Late Neolithic–Bronze Age (Cisbaikal_LNBA), is associated with Yeniseian-speaking groups and those that admixed with them, and ancestry from the second, Yakutia Late Neolithic–Bronze Age (Yakutia_LNBA), is associated with migrations of prehistoric Uralic speakers. We show that Yakutia_LNBA first dispersed westwards from the Lena River Basin around 4000 years ago into the Altai-Sayan region and into West Siberian communities associated with Seima-Turbino metallurgy—a suite of advanced bronze casting techniques that expanded explosively from the Altai.^1^ The 16 Seima-Turbino-period individuals were diverse in their ancestry, also harboring DNA from Indo-Iranian-associated pastoralists and from a range of hunter-gatherer groups. Thus, both cultural transmission and migration were key to the Seima-Turbino phenomenon, which was involved in the initial spread of early Uralic-speaking communities.

Uralic languages are spoken across Northern Eurasia, from Central Europe to Northeastern Siberia, but their homeland has been debated, with theories pointing to the Altai-Sayan mountains, between the Ob’ and Yenisei in Siberia, Europe around the confluence of the Volga and Kama rivers, or the East Baltic^2^ (Supplementary Information section 1 gives a guide to geographic terms). Present-day Uralic-speakers differ systematically from their Indo-European speaking neighbors in having substantial Siberian ancestry (from ~2% in Estonians to almost all in Nganasans), and a high frequency of Y-chromosome haplogroup N lineages of Siberian origin^3^. Time transects of ancient DNA show this ancestry arrived in Europe around ~3.5 thousand year ago (kya) in Karelia^4^ and ~2.6kya in the East Baltic^5^. In contrast to Indo-European languages, which can be traced by the dispersal of ancestry from the Yamnaya of the European steppe, no genetic “tracer-dye” has been found for the prehistoric dispersals of Uralic-speaking populations.

Yeniseian languages are attested only in populations along the middle and upper Yenisei, and Ket is the sole extant language. However, Yeniseian languages had a broader geographic spread in the past, and are linked in deep time with Na-Dene (Athabaskan–Eyak–Tlingit) languages of North America such as Chipewyan and Navajo, spoken from Alaska to Arizona^6^. Prior studies tried to find a genetic connection between Athabaskans and Kets^7–10^, but this has been challenging due to the genetic similarity of Kets to their non-Yeniseian neighbors.^11^ The disruptive effects of migrations associated with the later spread of Indo-European, Turkic and Mongolic languages^12,13^ also make it difficult to reconstruct the prehistoric migrations of Uralic and Yeniseian speakers based on genetic variation in present-day people.

We generated genome-wide data for 180 individuals across Northern Eurasia from the archaeological cultures from the Mesolithic (~11kya) to the Bronze Age (~4.0 kya), from the Volga-Ural region to the Lena River Valley of Central Siberia (Figure 1, see Extended Data Figure 1 for a map of sites; see Extended Data Figure 2 for a comprehensive chart showing the placements of the sites within geographic regions and in the archaeological cultures in each region’s cultural chronology; Archaeological context for each site and culture is provided and organized by region in Supplementary Information section 3; the Supplementary Information Guide provides the information needed to precisely localize the section of the Supplementary Information corresponding to each reference in the text, as well as descriptions of 35 Supplementary Tables (Tables S1–35) and 101 Supplementary Figure (Figures S1–101) referred to only within the Supplementary Information). We used in-solution enrichment for more than 1.2 million single nucleotide polymorphisms (SNPs) (Methods). We merged the data with 1,312 previously reported ancient individuals from relevant locations and time-periods. We also report 88 direct radiocarbon dates, which should be viewed with caution due to freshwater reservoir effects which can cause overestimates up to a millennium^14^ (Supplementary Information section 2). Our population labels identify genetically homogeneous individuals from a site—*Region_Site_ArchaeologicalPeriod_Time*—but for some analyses we use more aggregated groupings (glossary in Table 1; see Extended Data Figure 2 for the geographic and temporal placements of sites and their archaeological cultures).

We performed unsupervised genetic analyses, including Principal Component Analysis (PCA) and ADMIXTURE (Figure 1; Supplementary Information section 4 & 5), which show that individuals from a belt of pottery-using foraging cultures in the North Eurasian forest-steppe and southern edge of the forest zone ~10–5kya, form a genetic gradient stretching across ~7000km (Figure 1B,C; Extended Data Figure 3,4,5) that no longer exists today. We call this the North Eurasian hunter-gatherer (NEAHG) cline. The center of this cline lies close to the Ancient North Eurasian (ANE) individual Afontova-Gora 3 (AG3), and early Bronze Age people of the Tarim Basin (Tarim_EMBA^15^, Figure 1B; Extended Data Figure 3,4,5). However, many other populations do not fall on this cline, including Central and Northeast Siberian populations from further North (from deeper in the forest zone or from the Arctic), populations of the Amur Basin, and populations from the Cis-Baikal region after ~5kya.

To obtain insight into the genetic differences and population changes from ~17kya to ~4.0kya in this region, we proceeded to group individuals along the NEAHG cline, those from deeper into Northeastern Siberia, as well as those around the Lake Baikal into population groupings, which we then analyzed with a suite of population-genetic methods. Extended Data Figure 6 verbally summarized our five key findings, which are also graphically depicted in Extended Data Figure 7; we recommend the reader to peruse them before continuing. In the following sections, we present our analyses in order: first, on the population histories of Northeastern Siberia; next, on the NEAHG cline; and then after that, we dive into the connections that link two Bronze Age population groupings (Cisbaikal_LNBA and Yakutia_LNBA) and Yeniseian- and Uralic-speaking populations respectively.

## Paleosiberian Legacy in Asia and America

To investigate the population history of this region, we clustered 100 Holocene individuals from Northeastern Siberia and the Cis-Baikal and Transbaikal regions into genetic populations using f_4_-statistics. These groupings largely coincide with archaeological cultures (Supplementary Information section 6; Supplementary Data 1). We identified seven clusters: five with multiple members and two with single individuals. In chronological order these are: MiddleLena_KhatystyrCave_M_10.2kya (a newly-reported ~10.2kya individual from Khatystyr Cave along the Middle Lena; M is Mesolithic), MiddleVitim_Dzhilinda1_M_N_8.4kya (at the Mesolithic-Neolithic (M-N) boundary, from the Dzhilinda-1 site along the Vitim river from the Ust’-Yumurchen culture^16^), Transbaikal_EMN (~8.8–6.2 kya from the Early and Middle Neolithic (EMN) Kitoi culture east of the Baikal), Cisbaikal_EN (~8.0–6.6kya from the Early Neolithic (EN) Kitoi culture west of the Baikal), Syalakh-Belkachi (~6.8–6.2 kya from the Early Neolithic Syalakh and Middle Neolithic Bel’kachi cultures of the Middle Lena Basin), Cisbaikal_LNBA (~5.1–3.7kya from the Late Neolithic and Bronze Age (LNBA) Serovo, Isakovo, and Glazkovo cultures west of the Baikal), and Yakutia_LNBA (~4.5–3.2kya, associated chiefly with the Ymyyakhtakh culture). The remaining individuals were genetically intermediate and consistent with being admixtures of other groups we analyzed. In addition to these seven clusters, we added three older individuals—MiddleLena_Khaiyrgas_16.7kya^16^, Selenge_Ust-Kyakhta_14kya^17^, and Kolyma_M_10.1kya^9^—producing a ten-member transect (Extended Data Figure 8).

We used qpAdm to model each target population as derived from ones preceding or contemporary to them with the “outgroup rotation” method, that directly competes initially fitting models against each other to find best fits^18^ (Supplementary Information section 7, 8; Methods). For each of the ten populations, we found one or a small number of qualitatively similar passing models (p>0.05). Scans through large numbers of models should fail to falsify a subset of models that are in truth incorrect^19,18^, so our protocol should be viewed as a model-rejection, not model-selection procedure. The data were also generated through a mixture of wet lab processes (SNP enrichment ^8^ and shotgun sequencing), which raises concerns about false inferences due to technical biases having nothing to do with population history^20^. However, our inferences can be replicated in qpAdm setups utilizing sequences generated with a single wet laboratory process (Supplementary Information section 7). Our qualitative findings are also consistent with simple f_4_-statistics that test for affinities with distantly related populations plausibly relevant to the peopling of Northeast Siberia (Figures 2A,B; Supplementary Information section 8).

The oldest individual in our transect, MiddleLena_Khaiyrgas_16.7kya^16^ (from the Middle Lena in Yakutia, of the Dyuktai culture; Supplementary Information section 3) fits as a sister group of Native Americans, and can be modeled as descending completely from a Native American-related source (Supplementary Information section 8). The term “Ancient Paleosiberians” (APS) was used to designate the ancestry of the third individual in our transect (Kolyma_M_10.1kya)^9^, and here we broaden this term to designate the pre-Holocene (meta)population, admixed between ANE and East Asian ancestries, that gave rise to Native Americans, of which MiddleLena_Khaiyrgas_16.7kya may be a near-unadmixed representative. APS ancestry played a key role in the genetic formation of all later groups in our Siberian transect and the North American Arctic.

Our findings indicate that APS ancestry may have spread in the Northeast Siberian Upper Paleolithic with the “Beringian tradition” of lithics rich in conical and wedge-shaped microcores^21^. It persists in high levels, but admixed with additional East Asian ancestry, in two later individuals, Selenge_Ust-Kyakhta_14kya^17^ (south of Lake Baikal on the Selenge River with lithics from this same tradition) and Kolyma_M_10.1kya (close to the Bering Straits^9^; Supplementary Information section 8). Further west in the Altai, by the early Holocene (~9kya), admixture between APS and an ANE-related source formed Altai_N on the NEAHG cline (Supplementary Information sections 8), associated with the Neolithic Kuznetsk-Altai culture of the Upper Ob’ and Altai foothills (Supplementary Information section 3).

Prior work has shown that “Neosiberian” East Asian ancestry increased while APS ancestry declined in Northeast Siberia throughout the Holocene^9^. We find this increasing East Asian ancestry can be traced to at least two sources: Inland Northeast Asian-related ancestry, which we proxy by the Inner Mongolian Yumin individual ~8.4kya ^22^ (China_NEastAsia_Inland_EN), and Amur Basin-related ancestry, represented by pre-Holocene hunter-gatherers of the Amur Basin ~14kya ^23^ (China_AmurRiver_14K). The oldest individual in our Siberian transect with high East Asian and low APS ancestry, MiddleLena_KhatystyrCave_M_10.2kya, had strong affinities to Amur River hunter-gatherers (Supplementary Information section 8; Figure 2B), but subsequent populations further south (including the Kitoi-associated Transbaikal_EMN and Cisbaikal_EN at ~8.8–6kya, and Mongolia_N_North at ~7.5kya) have increasing affinities to the Inland Northeast Asian source (Figure 2B). We find that later groups are differentiated by their mix of East Asian ancestries: individuals falling along the North Eurasian Hunter-Gatherer (NEAHG) cline, including Cisbaikal_EN and Transbaikal_EMN, have a characteristic mixture of East Asian ancestries intermediate in affinity between the Inland and Amur-related sources, but non-NEAHG populations, such as foragers from the Amur River Basin or Cisbaikal_LNBA, have different ratios (Supplementary Information sections 8).

**Figure 1 |:**
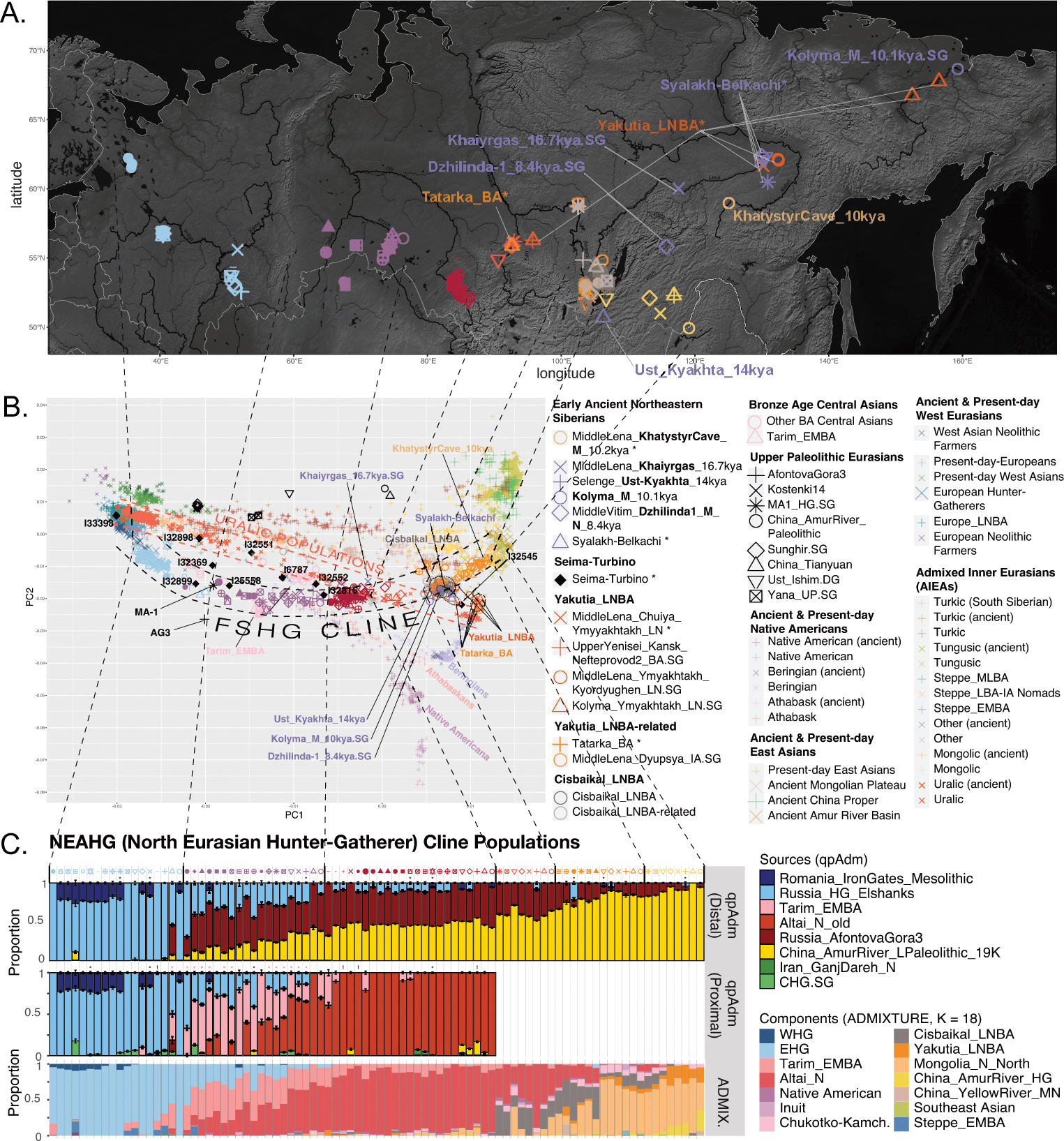
The North Eurasian Hunter-Gatherer (NEAHG) Cline and its legacy through admixture in ancient northern Eurasia. (Top) Map. A higher-resolution version of this image, with all population labels indicated, can be found in Extended Data Figure 12. Sampling locations of all individuals, and selected samples not on the cline mentioned in the text. **(Center) PCA.** We project ancient and present-day data onto variation from 122 genotyped present-day Eurasian and Native American populations selected to have minimal sub-Saharan African and Oceanian admixture. We observe a continent-spanning NEAHG cline, as well as a cline for Uralic populations stretching from European and Bronze Age Steppe populations to present-day Nganasans, Yakutia_LNBA individuals, and the ST-period site of Tatarka. **(Bottom)** Admixture Proportions. The first row of bar graphs presents qpAdm estimates of ancestry related to four sources (*Russia_AfontovaGora* for ANE, *China_AmurRiver_LPaleolithic_19K* for East Asian, *Russia_HG_Elshanks* for EHG, and *Romania_IronGatesMesolithic* for WHG) for all populations on the NEAHG cline. 84 out of 93 have passing models (p>0.01); populations that do not have a star above the bar plot. In these cases we show the model with the highest p-value. The second row of graphs display estimated admixture proportions for all 8 sources in the legend (expanding to include *Tarim_EMBA1, Altai_N_9kya, Iran_GanjDareh_N* and *CHG*); a pink dot above the bar plot indicates that all passing *qpAdm* models have *Tarim_EMBA* in the sources. A cross indicates a population used as a source (*Altai_N_9kya; Russia_MiddleVolga_Elshanka_Chekhalino_4_10kya*). For the Elshanka individual, we replaced the EHG source with Russia_Veretye_Mesolithic.SG.

**Figure 2 |:**
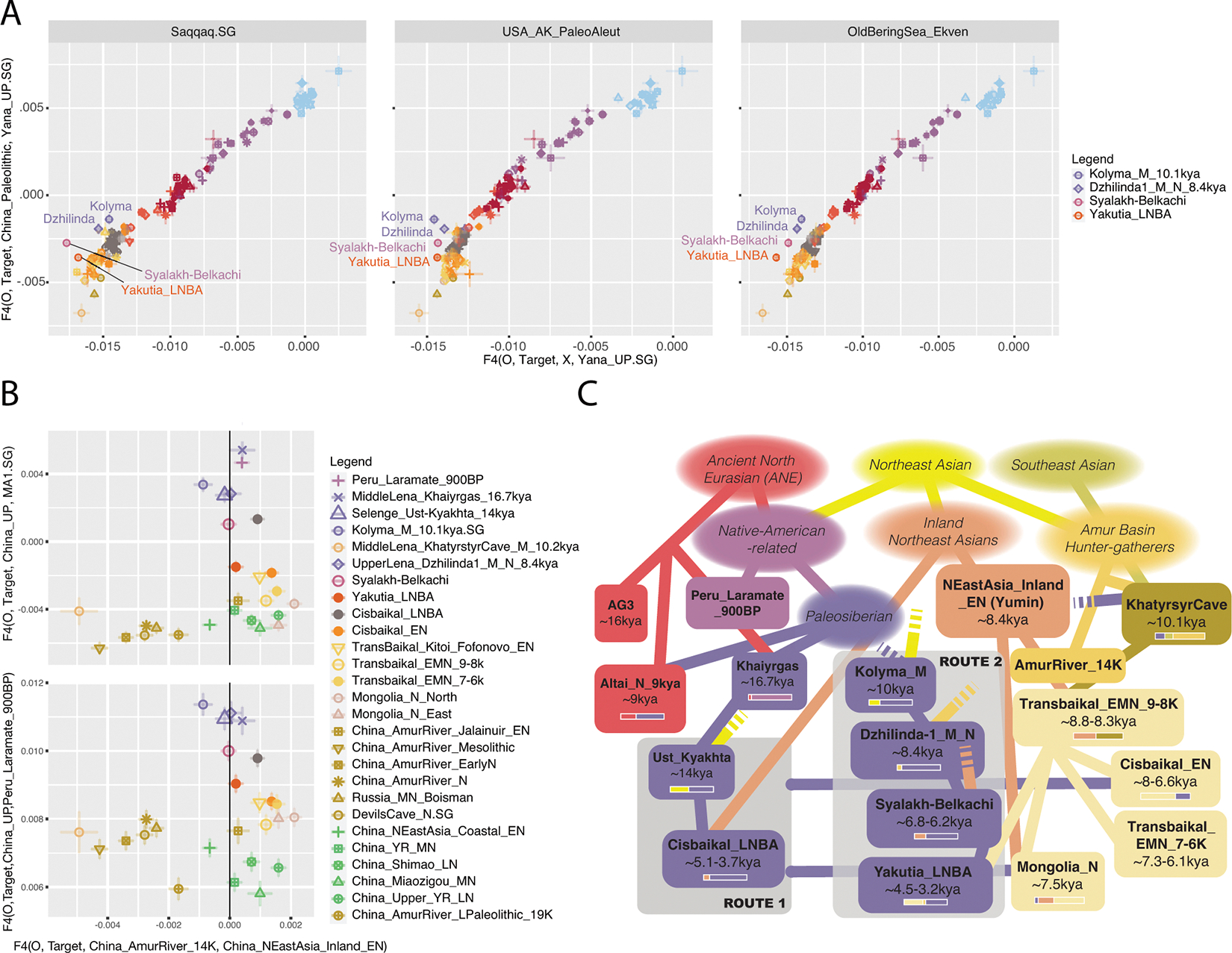
Middle Holocene populations and admixture events that formed them. **(A, Top)** Statistics of the form f4(Ethiopia_4500BP, Target, China_Paleolithic, Yana_UP), vs. f4(Ethiopia_4500BP, Target, X, Yana_UP), where X are ancient Native Americans or populations from the Bering Straits. The Target population’s position on the y-axis is proportional to its ratio of ANE and East Asian ancestry. *Kolyma_M_10.1kya, MiddleVitim_Dzhilinda1_M_N_8.4kya*, *Syalakh-Belkachi,* and *Yakutia_LNBA* are shifted left, indicating they share more drift with ancient Bering Straits groups than other populations with similar ratios of ANE and East Asian ancestry. **(B, bottom left)** Statistics of the form f4(Ethiopia_4500BP, X, China_NEastAsia_Inland_EN, China_AmurRiver_Mesolithic 14K), against f4(Ethiopia_4500BP, X, China_Paleolithic, MA1_HG) (left top) and f4(Ethiopia_4500BP, X, China_Paleolithic, Peru_Laramate_900BP) (left bottom), where X are ancient populations in Northeast Asia and Siberia. These statistics detect differentiation between an Inland East Asian-related source (proxied by the Yumin hunter-gatherer *China_NEastAsia_Inland_EN*), and an Amur-River-related source (represented by the *China_AmurRiver_Mesolithic_14K*). Populations from the Amur River region always have high affinity to *China_AmurRiver_Mesolithic_14K*, while those on the Mongolian Plateau and the Baikal area share more affinity with Yumin. The earliest strongly East Asian individual in Siberia, the Mesolithic *MiddleLena_KhatystyrCave_M_10.2kya*, is extremely Amur-River-related; other Northeastern Siberian groups high in APS ancestry, such as *MiddleVitim_Dzhilinda1_M_N_8.4kya, Kolyma_M_10.1kya*, and *Syalakh-Belkachi*, have both affinities; *Cisbaikal_LNBA* has extreme Inland Northeast Asian-relatedness. Affinity to *China_NEastAsia_Inland_EN* increases among agriculturalist populations along the Yellow River Valley. **(C, bottom right)** Schematic of population relationships in Northeast Asia and East Siberia, deduced from qpAdm in a ten-member transect from ~17kya to ~4kya. Major findings are: 1) That the MiddleLena_Khaiyrgas_16.7kya population is a near-unadmixed representative of an “Ancient Paleosiberian” (APS) population with Native American affinities, 2) APS ancestry persisted through two routes, and 3) the East Asian ancestry of Siberians derives from an Amur Basin-related source and an Inland East Asian-related source.

**Figure 3 |:**
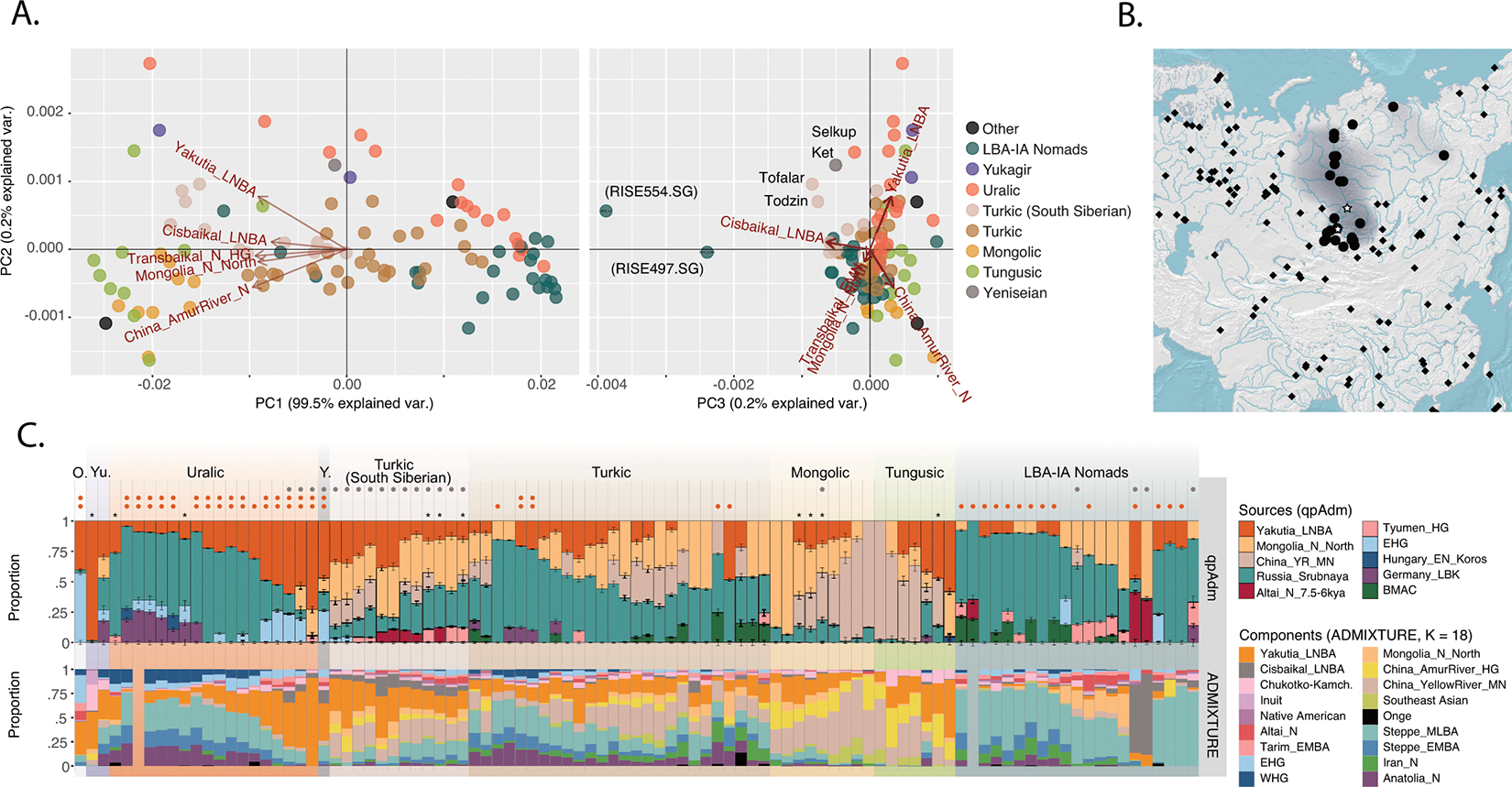
Contribution of Yakutia_LNBA and Cisbaikal_LNBA to Admixed Inner Eurasians (AIEAs). A version with all population labels indicated is in Extended Data Figure 11. **(A) PCA of f**_**4**_**-statistics.** A version with all population labels indicated is in Supplementary Information section 13. PCA of statistics of the form f_4_(*Ethiopia_4500BP, AIEA, AG3, East Asian*) measure the affinity between an *AIEA* population’s East Asian ancestry and a panel of tested East Asian populations: *China_AmurRiver_N, Mongolia_N_North, Transbaikal_EMN, Cisbaikal_LNBA,* or *Yakutia_LNBA*. PC1 is correlated with proportion of any type of East Asian ancestry. At a given proportion of East Asian ancestry, ancient and present-day Uralic-speaking populations shift in PC2 in the direction suggesting disproportionate *Yakutia_LNBA-*relatedness. PC3 highlights similarity to Cisbaikal_LNBA (right), with most affinity in Yeniseians, South Siberian Turks, Samoyeds, and two Upper Yenisei outliers (~3.0–2.9kya, RISE497.SG and RISE554.SG, which our archaeological research suggests are from the Lugavskaya culture). **(B) Cisbaikal_LNBA contribution to present-day populations.** Populations with >4% Cisbaikal_LNBA ancestry, large black dots. Likely Lugavskaya culture outliers of the Minusinsk Basin, white stars. **(C) Ancestry modeling.** Top row gives qpAdm results for AIEA populations. One orange dot above the bars indicates that all East Asian ancestry can be modeled as *Yakutia_LNBA*; two indicate that—additionally—all passing models include *Yakutia_LNBA* among the sources. We also performed qpAdm with Cisbaikal_LNBA among the references and sources (Supplementary Information section 11); a grey dot indicates that all passing models include *Cisbaikal_LNBA* in the sources. The bottom row displays ADMIXTURE results. Almost all Uralic-speaking populations have East Asian ancestry nearly exclusively assigned to the Yakutia_LNBA component; Yeniseians, South Siberian Turks and Samoyeds are the only populations with appreciable levels of the Cisbaikal_LNBA-related component. The two likely Lugavskaya culture outliers of the Minusinsk Basin are the only individuals with almost all their ancestry assigned to the Cisbaikal_LNBA component.

In the Cis-Baikal region during the mid-Holocene, ancestry from Cisbaikal_EN (8–6.6kya) was replaced by Cisbaikal_LNBA (~5.1–3.7kya), in a turnover coinciding with the transition from the Early Neolithic Kitoi to the Late Neolithic and Bronze Age Serovo, Isakovo and Glazkovo cultures (Supplementary Information section 8). Cisbaikal_LNBA is much higher in APS ancestry than its predecessors, which can only be modeled as deriving from an Ust-Kyakhta_14kya-related source, but this result should be viewed with caution due to the long time-gap separating the two populations. Cisbaikal_LNBA is also distinctive in having the most strongly Inland-related East Asian ancestry in our transect (Figure 2B; Supplementary Information sections 8; 11). Despite elevated APS ancestry, Cisbaikal_LNBA does not have increased shared drift with Native Americans or Bering Straits populations compared to other groups that are similar mixtures of ANE and East Asian ancestry (such as Ust-Kyakhta_14kya, Khaiyrgas_16.7kya.SG or NEAHG populations from the Upper Yenisei; Figure 1B, Figure 2A). Instead, it shares high levels of drift with present-day populations from Central Siberia, especially the Yenisei River Basin (Extended Data Figure 9; Supplementary Information section 8). In upcoming sections, we show that Cisbaikal_LNBA-related ancestry may be the first of two routes by which APS ancestry persisted into present-day populations, here those of in Central Siberia—I.e., it is a “Route 1” population (Figures 2C, 3B; Supplementary Information section 8).

North of the Baikal region along the Lena, the MiddleLena_KhatystyrCave_M_10.2kya individual derived most ancestry from Amur-Basin hunter-gatherers, but admixture from a Kolyma_M_10.1kya-related source caused an increase in APS ancestry in the following MiddleVitim_Dzhilinda1_M_N_8.4kya (Supplementary Information section 8). APS ancestry then declined with admixture from East Asian sources, in a set of population turnovers that seem to coincide with transitions between archaeological cultures. The first saw the transition from MiddleVitim_Dzhilinda1_M_N_8.4kya to the Syalakh-Belkachi population (~6.8–6.2kya), with ~20% admixture from an East Asian source from the Baikal region. The second saw another ~50% admixture into Syalakh-Belkachi from Transbaikal_EMN to create the Ymyyakhtakh-associated Yakutia_LNBA population (~4.5–3.2kya).

This sequence of four populations in Northeast Siberia (Kolyma_M_10.1kya, MiddleVitim_Dzhilinda1_M_N_8.4kya, Syalakh-Belkachi, and Yakutia_LNBA) is uniquely shifted towards Native Americans and Bering Straits populations in PCAs (Figure 1; Supplementary Information section 8; Extended Data Figures 5,9). In f_4_-statistics, they share more drift with ancient and present-day Bering Straits populations than any groups with similar proportions of ANE and East Asian ancestry (Khaiyrgas_16.7kya, Ust-Kyakhta_14kya, Cisbaikal_LNBA, and all NEAHG east of the Altai; Figure 2A). Using qpAdm, we confirm that the third member of this sequence—Syalakh-Belkachi—made a major (~70%) contribution to people of the Arctic Small Tool Tradition in North America (represented by the Paleo-Eskimo Greenland_Saqqaq.SG and other individuals from the Dorset and related cultures, also reported in ^16^; Supplementary Information section 9). This Syalakh-Belkachi-related Paleo-Eskimo ancestry persisted in all later populations around the Bering Straits, including those related to present-day Eskimo-Aleuts, Chukotko-Kamchatkans and Yukaghirs, accounting for the unique trans-Beringian genetic connections of this four-member sequence of populations. We propose that this represents the second major route by which APS ancestry persisted: I.e., these are “Route 2” populations (Figure 2C; Supplementary Information section 8).

However, Ancient Athabaskans are an exception in that they do not require this ancestry from Greenland_Saqqaq.SG, corroborated by their behavior in f_4_-statistics (Figures S101,S102). This suggests that Athabaskans and Paleo-Eskimos do not derive APS ancestry from the same source—in tension with previous findings by our group^7,8^ and confirming suggestions of multiple Holocene migrations from Eurasia into the Americas ^9,10,24^. Instead, we find suggestive, but weak, evidence for the involvement of a “Route 1” population in the APS admixture into Ancient Athabaskans (Supplementary Information section 9). Linguists have discovered a connection between Yeniseian languages of Central Siberia and the Na-Dene languages of North America^6^, and our results may provide some genetic support for the Dene-Yeniseian hypothesis.

## North Eurasian Hunter-Gatherer (NEAHG) cline

Further south, from ~10–4kya, all 150 newly reported and 81 previously published individuals from the North Eurasian forest-steppe and the southern edge of the forest zone fall into a genetic arc—the NEAHG cline—connecting pottery-using Eastern European foragers to their counterparts in the Transbaikal region, visible in ADMIXTURE (Figure 1C, third row), and in multiple PCAs (Figure 1B; Extended Data Figure 3,4,5). We grouped NEAHG individuals by site, time, then genetic similarity in PCA and ADMIXTURE (Extended Data Figures 3,4,5; Extended Data Figure 10; Figure 1A; resulting group labels in Extended Data Figure 11 & Supplementary Data 1). The great majority can be modeled in *qpAdm* (restricted to 1240K data; for analytic details of all qpAdm analyses in this paper, refer to Extended Data Table 1) as mixtures of four ancestries (84 of 93 populations P>0.01): Western Hunter-Gatherer ancestry (WHG, represented by samples from Serbia after ~10kya ^25^), EHG ancestry (by I6413 from the Elshanka culture of the Middle Volga, the oldest pottery-using culture in Eastern Europe, ~8kya), ANE (by the AG3 individual from ~16kya ^26^), and East Asian (by Amur Basin foragers from ~19kya ^23^; Figure 1 bottom; Supplementary Information section 10; Supplementary Data 2). In the West, hunter-gatherers from the Baltic to the Urals in such cultures as the Elshanka, Pit-Comb Ware/Lyalovo, and Volosovo cultures have mostly EHG with low WHG, consistent with previous findings ^27,28^. East of the Urals, in Neolithic populations of the Tobol and Middle Irtysh rivers, and in the circle of Eneolithic West Siberian cultures using Comb-Pit Ware pottery, EHG admixed with ANE and low levels of East Asian ancestry, similar to the Botai population of the Kazakh Steppes (~5.4–5.1 kya)^12^ and previously-described West Siberian Hunter-gatherers^29^ (~6.6–8.1kya). Further east, individuals from the Kuznetsk-Altai culture of the upper Ob’ and the Altai foothills can be modeled as two-way admixtures of ANE and East Asian ancestry. This continues into individuals from Neolithic sites of the Upper Yenisei and Kansk River Basin, where ANE ancestry declines and East Asian ancestry increases. The gradient extends into the Kitoi culture of the Baikal region through Cisbaikal_EN, to terminate in Transbaikal_EMN.

We sought temporally proximal sources for the ANE ancestry of the NEAHG populations west of the Altai using qpAdm restricted to 1240k data (Extended Data Table 1). Two sources can account for all this ancestry (Figure 1C, second row; Supplementary Information section 10): a Tarim_EMBA-like population from Central Asia (~4kya ^15^), and the population of the Kuznetsk-Altai Neolithic (proxied by Altai_N_9kya). Tarim_EMBA postdates NEAHG populations, but ADMIXTURE and PCA suggest gene flow between a source related to them and NEAHGs in West Siberia (Figure 1B, third row of Figure 1C). West Siberian NEAHGs cannot be modeled without a Tarim_EMBA-related source (Figure 1C; Supplementary Information section 10), implying that hunter-gatherer populations related to Tarim_EMBA lived in Central Asia before the Bronze Age^15,30^, and contributed to groups living in the north.

The NEAHG cline fragmented in the mid-Holocene following migrations from both West and East (Extended Data Figure 7). From the West, these brought Steppe_EMBA ancestry with Yamnaya pastoralists, followed by Europe_LNBA and Steppe_LNBA with the expansion of the Fatyanovo, Sintashta and Andronovo cultures ^28,29,31^. In the East, other migrations drove a wedge of Cisbaikal_LNBA ancestry into the Baikal region of the NEAHG cline ~5.4 kya. Subsequently, admixture between Steppe_MLBA and other East Asian ancestries gave rise to admixed groups in multiple genetic clines that connect Turkic-, Mongolic-, Tungusic-, and Uralic-speaking populations^12^ (Figure 1C; Extended Data Figure 3,4,5). To evaluate the legacy that the NEAHG cline and Central Siberian populations left in later populations across Eurasia, we analyzed a set of AIEA (Admixed Inner Eurasian) populations—our term for ancient and present-day Uralic, Turkic, Mongolic, Tungusic and Yeniseian-speaking populations plus pastoralists of the Late Bronze Age and Iron Age such as Scythians, Sarmatians, and Xiongnu ^32–35^. We find that NEAHG populations contributed little to these later groups, but two non-NEAHG populations—Cisbaikal_LNBA and Yakutia_LNBA—contributed in important ways.

## Cisbaikal_LNBA tracks Yeniseian languages

The Cisbaikal_LNBA group (~5.1–3.6 kya; Extended Data Figure 8) is rich in APS ancestry, occupies a distinct position in PCAs (Extended Data Figure 7C) and has a uniquely strong affinity to Inland Northeast Asians (Figure 2B, Supplementary Information sections 8; Extended Data Figure 9). While other APS-rich groups from Northeast Siberia (i.e., all four “Route 2” populations) are more closely related to Bering Straits groups, four lines of analysis show Cisbaikal_LNBA shares more drift with present-day populations of the Yenisei Basin.

First, ADMIXTURE (Figure 3 and Extended Data Figure 12) shows that present-day Yenisei Basin groups such as Kets, Samoyeds, and Siberian Turkic-speakers are unique in harboring a Cisbaikal_LNBA-related component (Figure 3B & Extended Data Figure 12B; bottom rows of Figure 3C and Extended Data Figure 12C). Second, qpAdm models for these groups consistently fail when Cisbaikal_LNBA is used as a reference population; Cisbaikal_LNBA is a source in all passing models (bottom rows of Figure 3C & Extended Data Figure 12C; Supplementary Information section 11). Third, in a PCA over f4-statistics designed to detect differences between AIEA populations in affinities to different East Asian groups, Yeniseian-, Samoyedic- and South Siberian Turkic-speakers are shifted systematically in the direction produced by increased shared drift with Cisbaikal_LNBA (PC3 of Figure 3A & Extended Data Figure 12A; Supplementary Information section 11). Fourth, Y-chromosome sequences related to haplogroup Q-YP1691 found at high frequencies in Kets and at lower frequencies in Samoyedic and Siberian Turkic populations such as Selkups and Tuvinians ^27,36–38^ have been recovered only from Glazkovo males belonging to Cisbaikal_LNBA (Supplementary Information section 11).

Ethnolinguistic data and historical records indicate South Siberian Turks assimilated Yeniseian speakers, beginning with the arrival of the Yenisei Kyrgyz in the 6^th^ century CE and lasting to early modern times. Other Siberian Turkic languages—Yakut and Dolgan—are spoken by populations whose ancestors migrated in the last millennium from the region where South Siberian Turks live today ^39^. Further north, ethnographic records indicate that some Samoyedic-speaking groups sustained close relationships with Yeniseian speakers, with much intermarriage (Supplementary Information section 12).

Unexpectedly, we also found that two published ^32^ Late Bronze Age (~3.0–2.9kya) East Asian outliers from the Minusinsk Basin along the Upper Yenisei (RISE497.SG, and RISE554.SG) were consistent with having near-complete Cisbaikal_LNBA ancestry (~85–95%, Figure 3B; Supplementary Information section 11). These individuals had by far the strongest genetic affinity to Cisbaikal_LNBA among all modern or ancient AIEAs (Figures 3A, 3C; Extended Data Figure 12). They were labeled as from the Karasuk culture in the original publication, but our archaeological investigations indicate instead an alternative assignment to the Lugavskaya culture (Supplementary Information section 3). Thus, populations with very high Cisbaikal_LNBA were present along the Upper Yenisei, near where Cisbaikal_LNBA is maximized today, by the Late Bronze Age ~3.0kya (Figure 3B). Except for Ket, all six other now-extinct Yeniseian languages were spoken in the region where Cisbaikal_LNBA peaks today (Figure 3B). The Ket themselves reached their current northward location in a recent expansion as late as the 17^th^ century (Supplementary Information section 12).

These findings match reconstructions—based on the distribution of Yeniseian hydronyms—of a Yeniseian homeland between the Cis-Baikal region and the Upper Yenisei (Supplementary Information section 12). The Cisbaikal_LNBA population first appears genetically ~5.4–3.8kya in the Serovo, Isakovo, and Glazkovo cultures (Supplementary Information section 11). Along the Middle Angara (which drains out of Lake Baikal into the Yenisei), it appears alongside Glazkovo artifacts in samples buried according to Glazkovo traditions (Supplementary Information section 3). Cisbaikal_LNBA ancestry may thus trace the movements of Yeniseian speakers even further into prehistory.

## Yakutia_LNBA tracks Uralic languages

Yakutia_LNBA (Figure 1, Extended Data Figure 8) individuals belong chiefly to the Ymyyakhtakh culture of the Lena River Valley, and are among the “Route 2” populations sharing distinctive genetic drift with Bering Straits groups (Figure 2A, Supplementary Information section 8). They can be modeled as a ~50–50% mixture between the preceding Syalakh-Belkachi population of the Lena Valley and the Trans-Baikal Kitoi population (Transbaikal_EMN; Supplementary Information section 8). The connection with Transbaikal_EMN is also supported by shared subclades of Y-chromosome haplogroup N (Supplementary Information section 13) and is consistent with archaeological reconstructions of Ymyyakhtakh origins (Supplementary Information section 3). However, an individual recovered from the Krasnoyarsk-Kansk forest-steppe far to the southwest of the Lena River Valley at ~4.2kya (Kra001.SG from the Nefteprovod-2 site^16^), in a location otherwise occupied by populations from the NEAHG cline, was also genetically Yakutia_LNBA, suggesting that Yakutia_LNBA individuals may have dispersed from Northeast Siberia to the forest-steppes North of the Altai-Sayan shortly before 4.0kya, which coincides with the spread of Ymyakhtakh pottery to this region at that time.

Yakutia_LNBA is unambiguously associated with ancient and present-day Uralic-speaking populations. First, in ADMIXTURE at K=18, a component maximized in Yakutia_LNBA appears that peaks today in Nganasans and accounts for almost all East Asian ancestry in Uralic-speakers; non-Uralic AIEAs have no Yakutia_LNBA, or other East Asian components in addition to Yakutia_LNBA (Figure 3C; Extended Data Figure 12C). Second, in a PCA of f_4_-statistics, Uralic speakers are shifted in the direction indicating increased affinity towards Yakutia_LNBA relative to other East Asian ancestries (PC2 in Figure 3A & Extended Data Figure 12A; Supplementary Information section 13). Third, a different set of f_4_-statistics indicates that, at any level of East Asian admixture, the AIEA population with the highest affinity to Yakutia_LNBA over other East Asian ancestries is always a Uralic-speaking population (Extended Data Figure 13; Supplementary Information section 13). Fourth, qpAdm models for Uralic speakers always require Yakutia_LNBA as a source, usually accounting for all their East Asian ancestry (top rows of Figure 3C & Extended Data Figure 12C; Supplementary Information section 13), in contrast to other ethnolinguistic groupings of AIEAs who always have other East Asian sources. Finally, Yakutia_LNBA males carry Y-chromosome subclades of haplogroup N that are present at high frequency in present-day speakers of Uralic languages ^3^ (Supplementary Information section 13).

## The spread of Yakutia_LNBA west in the ST phenomenon

Populations from Eastern Europe to West Siberia as late as the MLBA (Fatyanovo, Sintashta and Andronovo cultures) do not show any Yakutia_LNBA ancestry^3–5^, but present-day Uralic speakers from the same regions do, suggesting a westward spread of Yakutia_LNBA ancestry partially replacing Steppe_MLBA and Europe_LNBA ancestry at ~4kya at the earliest^28,29,31^. This transition was potentially accompanied by the dispersal of Uralic-associated Y-haplogroup N, which is absent in Eastern Europe and West Siberia prior to the arrival of Yakutia_LNBA ancestry. Here we show that the earliest stages of this westward dispersal of Yakutia_LNBA ancestry occurred within the Seima-Turbino (ST) phenomenon.

The ST phenomenon refers to the sudden appearance of a similar suite of bronze artifacts made with advanced casting techniques that spread across a vast region of Northern Eurasia, from China to the Baltic, ~4.0kya^1,40^. Archaeologists agree it was responsible for the introduction of metallurgy into East Asia and the dissemination of advanced casting methods for tin bronze into Europe^41,42^. ST items are noted for their sophistication and refinement (Extended Data Figure 14); most are weapons, but some are objects of ritual significance. Most ST objects are isolated finds scattered across sites of diverse cultures, but many occur in ceremonial necropoli found across Western Siberia and Eastern Europe, which are large complexes of burials and sometimes empty ritual graves (cenotaphs) with rich collections of ST artifacts and casting molds. This unusual distribution has fueled speculation about the social nature of the ST phenomenon, as well as the identity of their bearers^1,43,40,44^. So far, the only material evidence found for the manufacture of ST bronze artifacts were recovered from residential sites of metal-using fisher-foragers of the Ob’-Irtysh basin and the region between the Upper Ob’ and Upper Yenisei—an extraordinary cultural association that has generated much comment in the archeological literature^1,43,44^.

**Figure 4 |:**
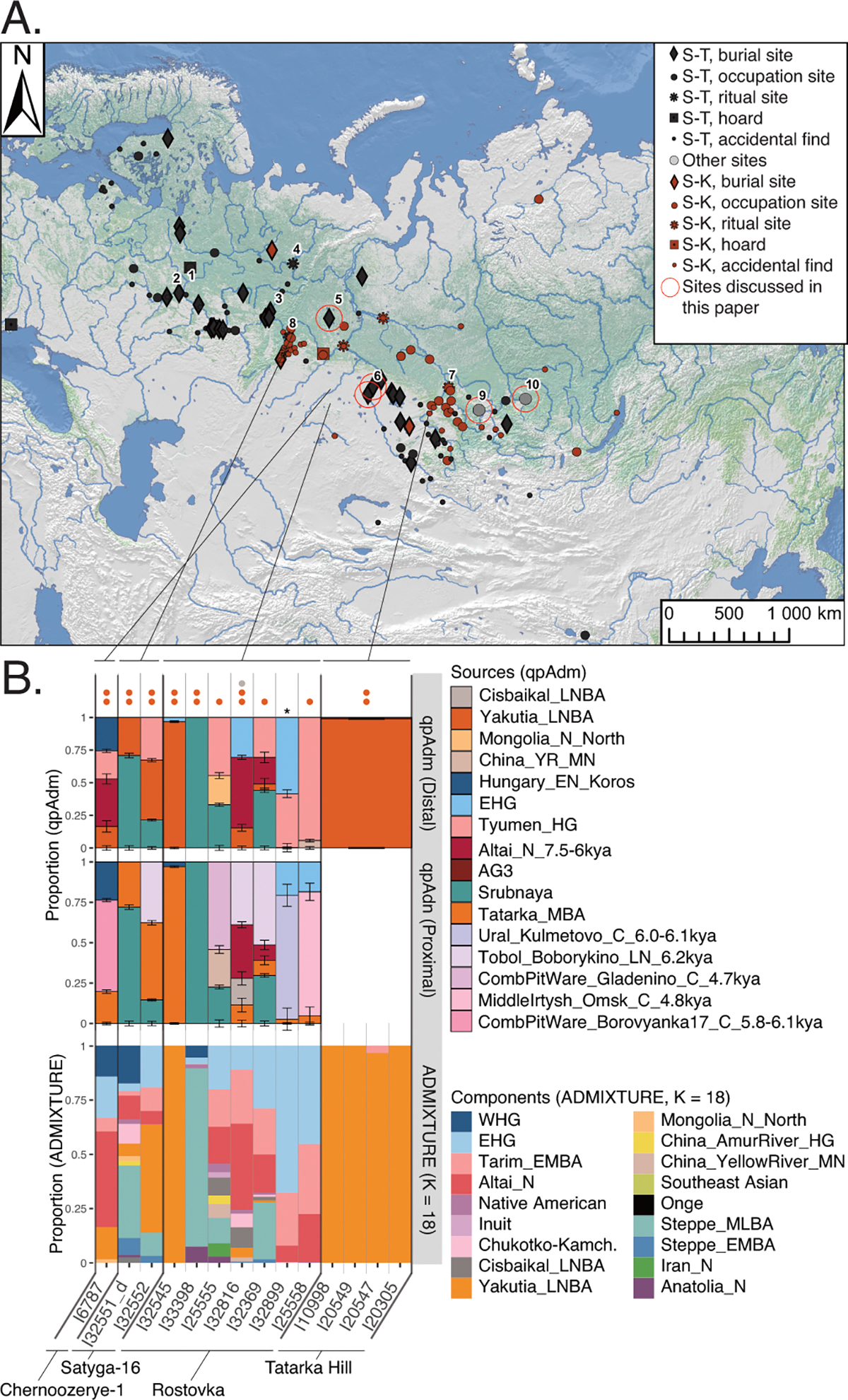
Genetics of the Seima-Turbino Phenomenon. **(A) Map of Seima-Turbino sites and finds.** Chernoozerye-1, Rostovka, Satyga-16 and Tatarka from which we have genetic data are marked in red circles; other important sites are numbered: 1, Seima. 2, Reshnoe. 3, Turbino. 4, Kaninskaya Cave. 5, Satyga-16. 6, Rostovka. 7, Samus-4. 8, Shaitanskoe Ozero. 9, Tatarka. **(B) ADMIXTURE and qpAdm proportions for 16 Seima-Turbino-period individuals.** 9 Rostovka, 2 Satyga-16, 1 Chernoozerye-1, 4 Tatarka. **top row:** selection of qpAdm models using a distal set of sources, with the simplest passing (p>0.01) qpAdm model with the highest p-value always displayed (same modeling as Figure 3B). No model passes for I32899 at p>0.01 as indicated by an asterisk above the bar). **center row**: qpAdm results for a more proximal set of sources, with individuals from Tatarka used as the source for Yakutia_LNBA and populations from Late Neolithic or Eneolithic Western Siberia (between the Urals and the Altai) as the source for NEAHG; *Yakutia_LNBA* has been added to the references. Ancestry from the population at Tatarka suffices to account for all the Yakutia_LNBA-related ancestry of the ST individuals even when Yakutia_LNBA is among the references. Samples that can be modeled with their East Asian ancestry derived completely from a *Yakutia_LNBA*-related source are highlighted by a single orange dot above their qpAdm bar charts, while those that require such a source in every passing model are highlighted by two such orange dots. One individual (I32816) requires *Cisbaikal_LNBA* ancestry among the sources (gray dot); the model displayed for this individual is the simplest passing model that contains both *Cisbaikal_LNBA* and *Yakutia_LNBA* ancestry among the sources. In both sets of qpAdm, the individual (I6787) from Chernoozersky-1 requires a contribution from a source from far West of the Urals (*WHG* ancestry. **bottom row:** ADMIXTURE proportions at K=18.

We generated genome-wide data from 16 individuals from four sites, dated to a tight interval around 4.0 kya (Supplementary Information section 2; Supplementary Data 1). Two—Rostovka on the banks of the middle Irtysh in the Ob’-Irtysh Basin with 9 individuals, and Satyga-16, east of the Mid-Ural Mountains with 2 individuals—are ST necropoli. We add to these samples from two ST-period sites that have less direct evidence of involvement with the ST phenomenon, but that our genetic analyses suggest may be connected with it: one from Chernoozerye-1, located close to Rostovka, and four males from a previously undescribed site, Tatarka Hill along the Upper Yenisei, on the Krasnoyarsk-Kansk forest-steppe North of the Altai. In our genetic modeling using *qpAdm*, the four individuals from Tatarka Hill are consistent with being entirely Yakutia_LNBA. In contrast, the individuals from Rostovka, Satyga-16, and Chernoozerye-1 harbor variable proportions of three primary and two minor sources of ancestry (Figure 1B, Figure 4; Extended Data Figures 4, 5; Supplementary Information section 15). Based on qpAdm, these ancestries are: 1) Yakutia_LNBA, 2) ANE-rich ancestry from the NEAHG cline, and 3) Steppe_MLBA, occuring in unadmixed individual representatives, or intermingled within admixed individuals (2-way: NEAHG ancestry + Steppe_MLBA, or 3-way: NEAHG ancestry + Steppe_MLBA + Yakutia_LNBA; top row of Figure 4B). Both individuals from Satyga-16 from further west are admixed (carrying all three ancestry types), contrasting with Rostovka (4/9 single-ancestry individuals: 2 NEAHG, 1 Yakutia_LNBA and 1 Steppe_MLBA; Figure 4E, Supplementary Information section 15).

Proximal qpAdm provides insight into the immediate ancestors of ST people. The Yakutia_LNBA ancestry in Rostovka, Satyga-16 and Chernoozerye-1 is related to the people of Tatarka Hill, with no additional mixture from Yakutia_LNBA in Central Siberia (Figure 4B). This link is reinforced by the presence, at Rostovka, of haplogroup N-L1026 (in the Yakutia_LNBA individual, I32545), also carried by all four males from Tatarka Hill (Supplementary Information section 13). The subclade in Rostovka (N-L1026 > Z1936) is widespread in present-day Uralic populations from West Siberia to the Baltic Sea, attaining maximal frequencies today (up to ~40%) near the Baltic in Finns, Veps and Karelians^45^. The NEAHG ancestry of ST individuals comes in large part from preceding, local Neolithic and Eneolithic populations of West Siberia (Figure 4E), consistent with an origin in the metallurgical foragers of the nearby Odinovo and Krotovo cultures, who engaged in the systematic casting of ST artifacts^44^. However, some individuals require additional NEAHG ancestry from further afield, from EHG-related or Altai_N-related sources.

The three primary ancestry sources are accompanied by two minor ancestries: non-Yakutia_LNBA East Asian ancestry, and WHG ancestry from as far west as the Baltic region (Figure 4E). The ST-period individual from Chernoozerye-1 (I6787) requires a large fraction of WHG in ADMIXTURE analysis and in all fitting qpAdm models (Supplementary Information section 15; Figure 4E). This is a remarkable case of a person whose recent ancestry traces to at least three hunter-gatherer populations from widely-separated regions of Eurasia (the Baltic, West Siberia, and the Altai-Sayan). Two individuals from Rostovka, I32816 and I33369, have ancestry from the east, in the former case from a Cisbaikal_LNBA-related source, possibly foragers of the contemporaneous Glazkovo culture.

Our results suggest that ST artifacts were manufactured, exchanged, and dispersed in a sociocultural context that integrated people from multiple populations across a continent-spanning network into coherent social groups interred together at single necropolises. Our samples capture a snapshot of this process, indicating a pattern of human mobility that is a genetic correlate to archaeological evidence for similarity in artifacts over vast geographic distances, unusual in cultural groups of the period ^1,46^.

## Discussion

Linguistic transmission in large-scale societies need not involve the movement of people, but the same process in smaller-scale societies is likely to require at least some degree of human mobility visible as genetic admixture. One major analytic finding is our identification of Cisbaikal_LNBA as a genetic tracer-dye for the spread of early Yeniseian language speakers. We further show that ancient Athabaskans from Alaska ~1.1kya are unique among Arctic North Americans in lacking ancestry from Paleo-Eskimo populations but possess tentative signals of ancestry from a “Route 1” APS population that also contributed distinctively to Yeniseian-speaking groups. These results help connect the movements of early Yeniseian-speaking groups to the Cis-Baikal region and may also provide—for the first time—tentative genetic support for the linguistic connection between Yeniseian languages of Siberia and Athabaskan languages of North America: the “Dene-Yeniseian hypotheses” ^5^. In our second major analytic finding, we show that Yakutia_LNBA may serve as an excellent tracer-dye for the spread of early Uralic-speaking communities, and that the earliest dispersals of this ancestry west was mediated by people associated with the Seima-Turbino (ST) phenomenon.

Archaeologists debate the social processes that drove the rapid spread of ST artifacts across such a wide range of cultures^1,43,44,46^. We find that people buried at ST necropolises were highly genetically variable, contradicting hypotheses of a homogeneous “Seima-Turbino people”^43,40^. Our results suggest either the one-time amalgamation of individuals from genetically and culturally distinct social groups into a mobile population (an event which may have taken place at a different location and prior to the ST sites themselves), or—based on the multi-way admixtures in ST necropolis individuals—the active, continuous interaction of people from multiple groups in activities that produced the sites over many generations. These findings are consistent with the heterogeneity of other cultural artifacts at ST necropolises, such as pottery (similar to that produced by West Siberian foragers^1,43,46^ and people of the Krasnoyarsk-Kansk forest-steppe around Tatarka Hill^47^); artifacts of flint, bone, or jade (similar to cultures of far Northeast Siberia and the Baikal); and metal items from non-ST traditions (from the Sintashta and Abashevo cultures)^1,43,46^. The three sources of material culture parallel the three major genetic ancestries at ST sites, also detected in an archaeogenetic study on Rostovka published simultaneously.^48^ Lastly, the presence of ancestry from multiple hunter-gatherer populations across vast distances (from the Cis-Baikal to as far West as the Baltic) in ST sites highlights the transformative social impacts of metal exchange networks in the Bronze Age^49,50^, and the accumulating but oft-neglected evidence for sociopolitical and economic dynamism in foraging populations^51,52,53^.

Our finding that Yakutia_LNBA ancestry first dispersed westwards, almost to Europe, with the ST phenomenon has archaeological and linguistic significance. The Kra001 individual at Nefteprovod-2 ~4.2kya, close to and just prior to the Tatarka Hill individuals, shows that Yakutia_LNBA ancestry first penetrated onto the Krasnoyarsk-Kansk forest-steppe by 4.2kya and persisted there before contributing to ST necropolises even further west. Nefteprovod-2 and Tatarka Hill share similar burial rites—suggesting that the genetic population bringing the Yakutia_LNBA ancestry to the Krasnoyarsk-Kansk forest-steppes that impacted ST necropolises, was also culturally cohesive (that we term the “Anzhevsky complex”, Supplementary Information section 3). Another material counterpart to the genetic link between ST necropolises and groups of ultimately Northeast Siberian origin can be found in suits of armor made of bone plates, which have been found from the Glazkovo and especially the Ymyyakhtakh cultures. One set was buried with a Yakutia_LNBA male (N4a1.SG from the Kyordyughen site) and others are from the Krasnoyarsk-Kansk forest-steppe around Nefteprovod-2 and Tatarka Hill (Supplementary Information section 3). Three sets can be found in Rostovka, one associated with a male (I32816 from Grave 33; Supplementary Information section 3) that bore both Yakutia_LNBA and Cisbaikal_LNBA ancestries.

Linguists have documented hundreds of Indo-Iranian loanwords that present-day Uralic languages have inherited from the Proto-Uralic speech community or from early Uralic communities right after its breakup^54,55^. The Indo-Iranian expansion has been linked to the spread of Steppe_MLBA ancestry from the Sintashta population of the Trans-Ural region into other parts of Central and West Asia (where it persisted into historically attested Iranic-speakers^33–35,56–58^), and further into South Asia^29^. Our findings from Rostovka and Satyga-16, showing contact and admixture between a Steppe_MLBA population (which, from archaeological considerations, is plausibly that of the Abashevo culture^1,41,43,59^) and Yakutia_LNBA, provides an attractive context in which this linguistic exchange could have first begun, and offers another line of evidence for Uralic-speaking groups being present at ST sites, in line with prior suggestions.^55,59^

Uralic languages, distributed from Western Siberia to Central Europe, are geographically separated from languages of the Eastern Steppes and far Northeast Siberia, but linguists have discovered traces of ancient connections with Yukagiric and Eskimo-Aleut languages on the one hand, and high levels of typological similarity with languages in the “Altaic” language area (Mongolic, Tungusic, and Turkic) on the other (Supplementary Information section 14). To resolve this conundrum, some linguists have suggested a recent eastern origin of the population giving rise to later expansions of Uralic speakers (e.g., a “pre-proto-Uralic spoken further east… probably somewhere… near both Mongolia and the watershed area between the Yenisei and the Lena, possibly as recently as 3000BC”^60^)—a scenario compatible with our results. Future ancient DNA sampling from this region would allow for a more precise determination of the archaeological identity of the Proto-Uralic-speaking community, and illuminate the relationship between it and the wider social world of the West Siberian Bronze Age.

## Methods

### Sampling of ancient individuals

All skeletal samples screened for ancient DNA were analyzed with permission from the appropriate authorities including in every case archaeologist or anthropologist custodians of the samples, and/or cultural institutions curating the samples. Descriptions of the archaeological and cultural contexts for all ancient samples analyzed, including their grave position within archaeological sites, their grave numbers and burial inventory, as well as references to archaeological publications for the sites themselves (where available), are provided in Supplementary information section 3. Contact information for finding out more about the samples we analyzed are listed in Column G in the sheet labeled “Ancient Indiviuduals” in Supplementary Data 1 (all samples, including previously-published samples) and “Bone Samples and Libraries” in Supplementary Data 1 (samples analyzed for this paper, including material that did not yield enough DNA for analysis). Samples may be identified by their skeletal code listed in the “Ancient Individuals” and “Bone Samples and Libraries” sheets of Supplementary Data 1.

### Sampling of present-day individuals

We newly genotyped 229 present-day individuals from 10 ethnolinguistic groups using the Affymetrix Human Origins SNP array. All DNA samples were collected with written informed consent for broad studies of population history and full public release of de-identified genetic data, using a protocol approved by the Ethics Committee of the Research Centre for Medical Genetics, Moscow, Russia. All newly reported data are represented either by co-authors of this study or individuals who wished to be mentioned in the Acknowledgments who were involved in sample collection. Details of all present-day genetic samples analyzed (all samples, including previously-published samples) are given in Table 1 in the sheet “Present-Day Individuals” Supplementary Data 1, while details entirely of newly-published samples are provided in the sheet called “Newly-Published Individuals” in Supplementary Data 1.

### Ancient DNA data generation, bioinformatic processing, and quality control

Approximately 37mg of bone powder was collected from each set of skeletal remains, after which DNA was extracted using a protocol that retains short and damaged DNA fragments ^61,62^. The bone powder was collected from petrous bones, long bones, teeth, and ossicles. Individually barcoded double ^63,64^ and single-stranded libraries ^65^ were built after incubation with uracil DNA glycosylase (UDG treatment, to reduce errors characteristic of ancient DNA damage). We performed in-solution enrichment for ~1.2 million SNPs (“1240k enrichment” ^66^) and also enriched for the mitochondrial genome ^67^. Two rounds of enrichment were performed, after which sequencing was performed on the Illumina NextSeq 500 or HiSeq X 10 instruments.

The resulting read pairs were separated using library-specific barcode pairs or index pairs (for double-stranded and single-stranded libraries respectively) and merged prior to alignment. Read pairs were merged if 1) 15 or more base pairs (bp) overlap, 2) at most one mismatch occurred and base quality was at least 20, 3) at most three mismatches occurred and base quality was lower than 20. The resulting sequences were aligned to the human genome reference sequence (hg19) ^68^ and the mitochondrial RSRS genome using samse from bwa-v.0.6.1 ^69,70^. Duplicated sequences were removed if they shared start and stop positions, orientation, and (for double-stranded libraries) barcode pairs. Analysis was performed on sequences at least 30 bp in length. We trimmed 2 bp from the ends of each read to reduce deamination errors. For each sample, we merged the sequences from all libraries. Most of the datasets used for population genetic analysis were generated by randomly sampling at each SNP on chromosomes 1–22 and X, with a mapping threshold of 10 and base quality 20.

We flagged as “questionable” libraries that had evidence of contamination based on the upper bound of the match rate to the mitochondrial consensus sequence (assessed using contamMix v1.0–10, ^71^) being less than 95%; we also flagged as “critical” libraries if this value was less than 90% (sheet labeled “Ancient Individuals” in Supplementary Data 1). We flagged as “questionable” males with evidence of high polymorphism on the X chromosome (lower bound of the 95% confidence interval for mismatch rate >1%), or as “critical” (if >5%), estimated using ANGSD v0.923 ^72^. For high-coverage contaminated individuals, we generated alternative sequences restricting to molecules showing signs of characteristic ancient DNA damage (designated by a suffix “_d” in the Genetic ID of the sample in the “Ancient Individuals” sheet of Supplementary Data 1).

For a subset of 15 individuals with high percentages of human DNA, we generated shotgun sequences (designated by the suffix “.SG” in the “Ancient Individuals” sheet of Supplementary Data 1) using the pre-enrichment libraries. We carried out sequencing on an Illumina HiSeq X Ten instrument. These shotgun sequences were used for analysis only in PCAs (Supplementary Information, Section 4).

### Uniparental analysis

Mitochondrial haplogroups were determined with Haplogrep v2.1.1 ^73^. Y-chromosome haplogroups were evaluated using the methodology described in ^74^, section S5, using both targeted and off-target SNPs. Allelic status was determined by majority rule.

### ADMIXTURE and PCA

All relatives and shotgun sequences were excluded from ADMIXTURE analysis. For relative pairs or groups, the lower-coverage individual was excluded.

We used ADMIXTURE v.1.3.0 ^75^ after pruning SNPs with high missingness in plink v.1.0.7 (using option --geno 0.5 ^76^), after which 597,573 autosomal SNPs were retained. We used K=18 as the first K value where Yakutia_LNBA and Cisbaikal_LNBA were separated from East Asian components characteristic of NEAHG populations (e.g. the components maximized in Mongolia_N_North and AmurRiver_14K). Further details of our application of ADMIXTURE can be found in Supplementary Materials Section 5, including our ADMIXTURE cross-validation error (Supplementary Information section 5).

We pruned individuals from PCA analysis if they were found to be a first-degree relative of another individual in the dataset with high coverage. PCA was performed using smartPCA in the EIGENSOFT package ^77^, using numoutlier: 0 and lsqproject: YES for three out of four PCAs. Further details on our PCAs can be found in Supplementary Information, Section 4.

### qpAdm analyses and f4 statistics

All f4-statistics were calculated using the *qpDstat* package of *ADMIXTOOLS* v.7.0 ^78^ with the f4mode: YES parameter. Further details of each set of f4-statistic calculations can be found where they are presented, in Supplementary Information sections 8,9,11,13.

All qpAdm analyses were run using the R package ADMIXTOOLS2 ^79^. Precalculated *f*_*2*_-statistics, used to speed up the process of *f4*-ratio estimation central to *qpAdm*, were performed allowing for maximal missingness = .99 over multiple datasets. Further details for each set of qpAdm can be found in Supplementary Information sections 8, 9, 10, 11, 13. Additionally, details on all our sets of qpAdm analyses can be found in Extended Data Table 1.

The results of these qpAdm analyses are found in Supplementary Datas S2–S7. In these files, the tables listing qpAdm results are sorted first by target; then for each target, models with all positive coefficients are listed first, ahead of the rest. The all-positive-coefficient models for each target are themselves sorted, first by simplicity (i.e., one-source all-positive models listed first, then two-source all-positive models, then three-source, etc.), and then (among the all-positive models with the same number of sources) ranked by p-value. This same ordering is used for models with negative coefficients for each target (that is, they are listed first by simplicity, then by p-value). Results for each target population are easily accessible by filtering on the “Target” column, and then by the threshold p-value one picks, which would automatically list all passing models starting with the simplest all-positive models with the highest p-value.

We made sure whenever possible that the populations included in left/sources and right/references in our qpAdm sets were always processed through only one set of wet laboratory procedures: through 1240K enrichment. For analyses where population groups in the left/sources and right/references included both 1240K and shotgun sequences, wherever possible, we performed replicate analyses where shotgun individuals were purged from all the group labels in the left/sources and right/references. Our replicate analyses show that our main conclusions in qpAdm are relatively robust to the effects of allelic bias (SI Sections VI.C.ii.a and VI.D.ii.a).

### Relatedness and Runs-of-Homozygosity

We looked for kinship relationships between the individuals included in our study. We computed pairwise allelic mismatch rates in the autosomes by randomly sampling one DNA sequence at each ‘1240k’ polymorphic position, following the same strategy as in ^80^, ^81^ and ^82^, which is similar to that in ^83^. We then estimated relatedness coefficients r for each pair as in ^80^:

r=1−((x−b)/b)

with x being the mismatch rate of the pair under analysis and b the base mismatch rate expected for two genetically identical individuals from the population under analysis, which we estimated by computing intra-individual mismatch rates. We also computed 95% confidence intervals using block jackknife standard errors over 5 Megabase (Mb) blocks.

## Extended Data

**Extended Data Figure 1 |:**
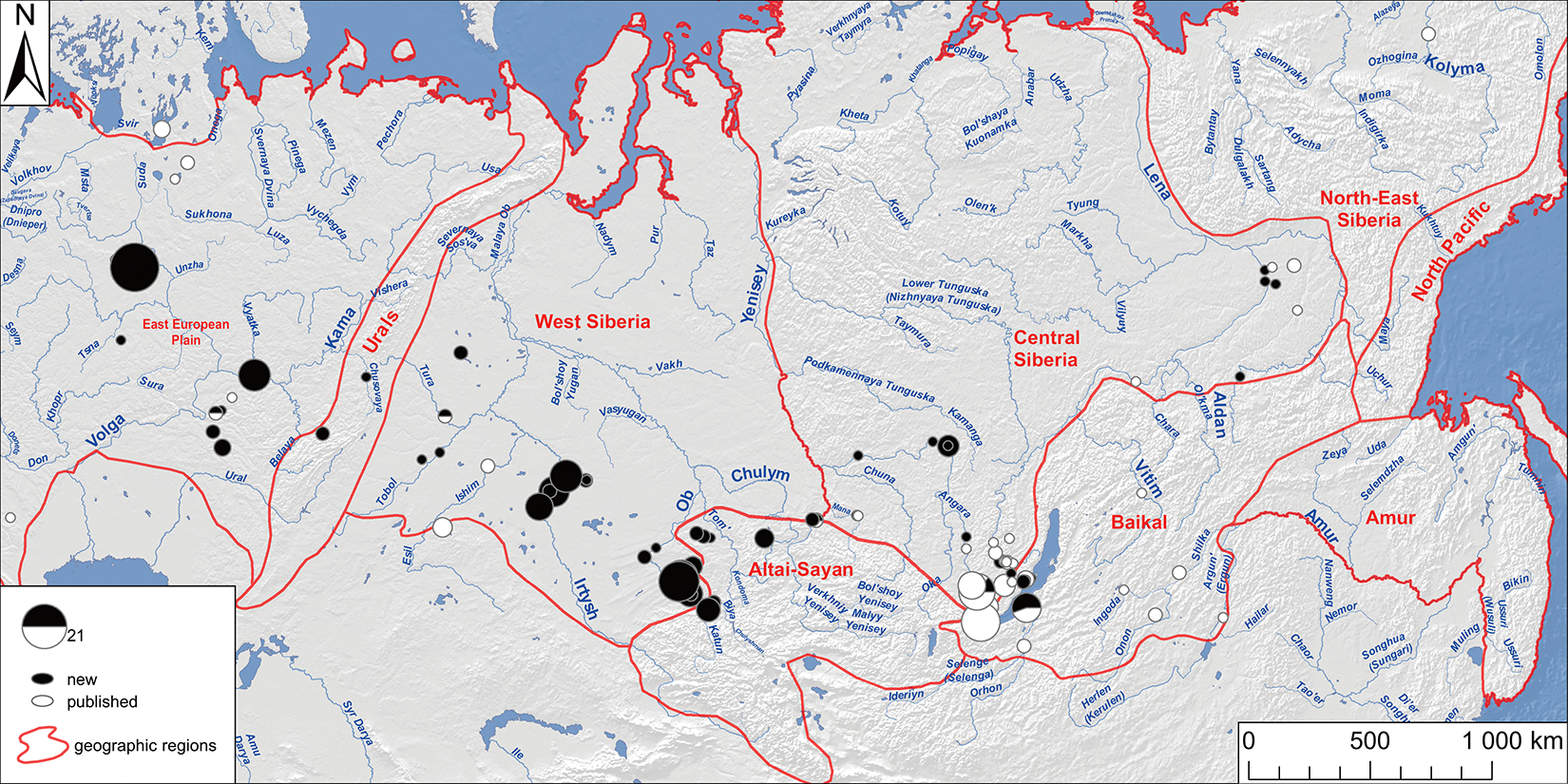
Sites with newly-reported samples. This map displays all sites from which samples that fall in all major populations that are the subject of focus in this paper have come. These include all sites 1) whose samples fall on the NEAHG cline, 2) whose samples fall in the Cisbaikal_LNBA cluster or are admixed with it, 3) whose samples fall in the Yakutia_LNBA cluster or are admixed with it, 4) who are a part of the ten-population East Siberian transect described in our qpAdm modelling, and 5) who are Seima-Turbino period individuals. Each site is represented by a pie chart, whose size is proportional to the number of individuals from that site; the white fraction represents previously-published samples, and the black newly-published samples. Our sampling fills geographic and temporal lacunae.

**Extended Data Figure 2 |:**
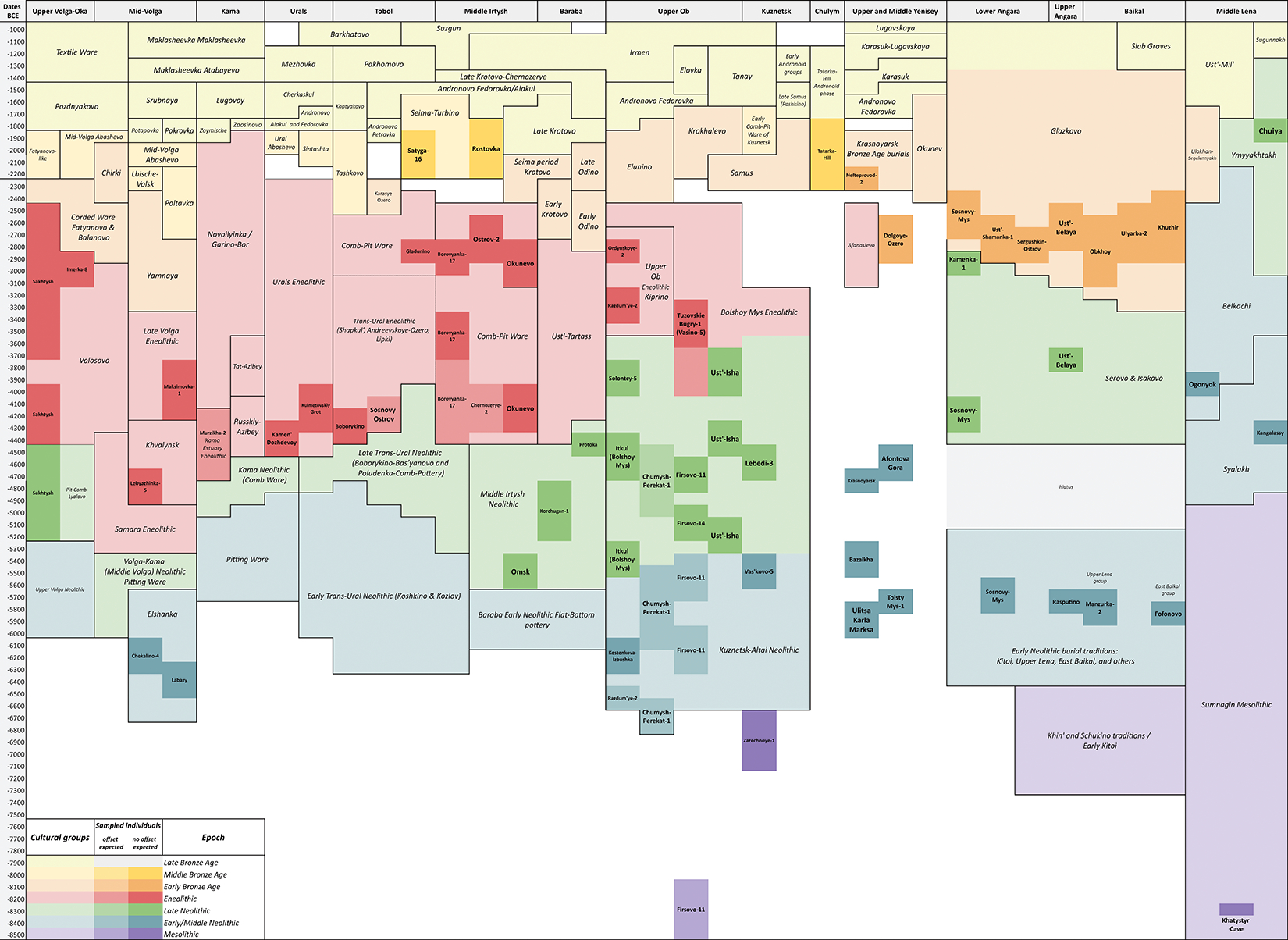
Chronology of sites and cultures in each geographic region. Temporal and geographic disposition of cultures from the Mesolithic to the Late Bronze and Iron Ages across Northern Eurasia. Sites whose samples are analyzed in our paper are highlighted in darker boxes, within containing boxes indicating archaeological cultures. Sites whose colors are darker are those that we believe are most securely dated (based on radiocarbon, isotopic, and archaeological evidence).

**Extended Data Figure 3 |:**
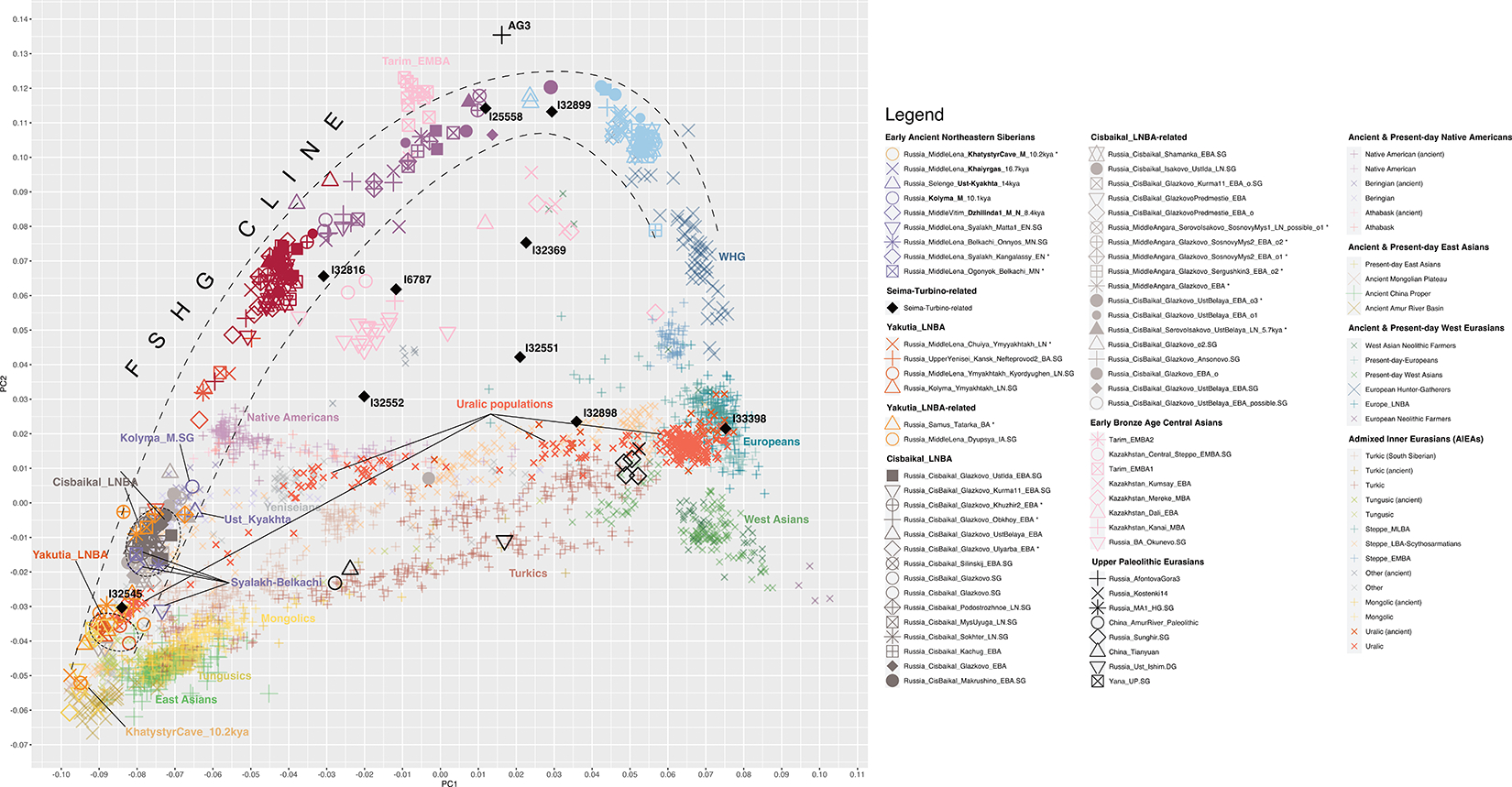
PCA with target populations projected onto ancient populations with an especially high fraction of ANE ancestry. To illuminate the role that levels of *ANE* ancestry plays in generating variation among the populations we analyze, we use as a basis for another projection 71 shotgun-sequenced ancient individuals from across Eurasia, of which a large proportion are enriched in *ANE* ancestry and fall outside the range of present-day variation (e.g. individuals from populations like *Tyumen_HG.SG* or *Kazakhstan_Botai.SG*; for full list, see Supplementary Information section 4). The North Eurasian Hunter-Gatherer cline forms a curved arc stretching from *EHG* populations to present-day East Asians; the center of the arc dominated by populations rich in *ANE* ancestry is moved toward the positive direction in PC2. The individual furthest along the positive direction in PC2 is AG3. Clines formed by later Inner Asian populations, such as present-day Uralic, Turkic, and Mongolic speakers, as well as Late Bronze Age and Iron Age steppe populations such as Scythians and Sarmatians, are distinguished from the *NEAHG* cline by their much lower values along PC2, suggesting a much lower level of ANE ancestry. This PCA shows that populations along the NEAHG cline, remaining stable for many millennia, were substantially outside the range of present-day genetic variation in Northern Eurasia.

**Extended Data Figure 4 |:**
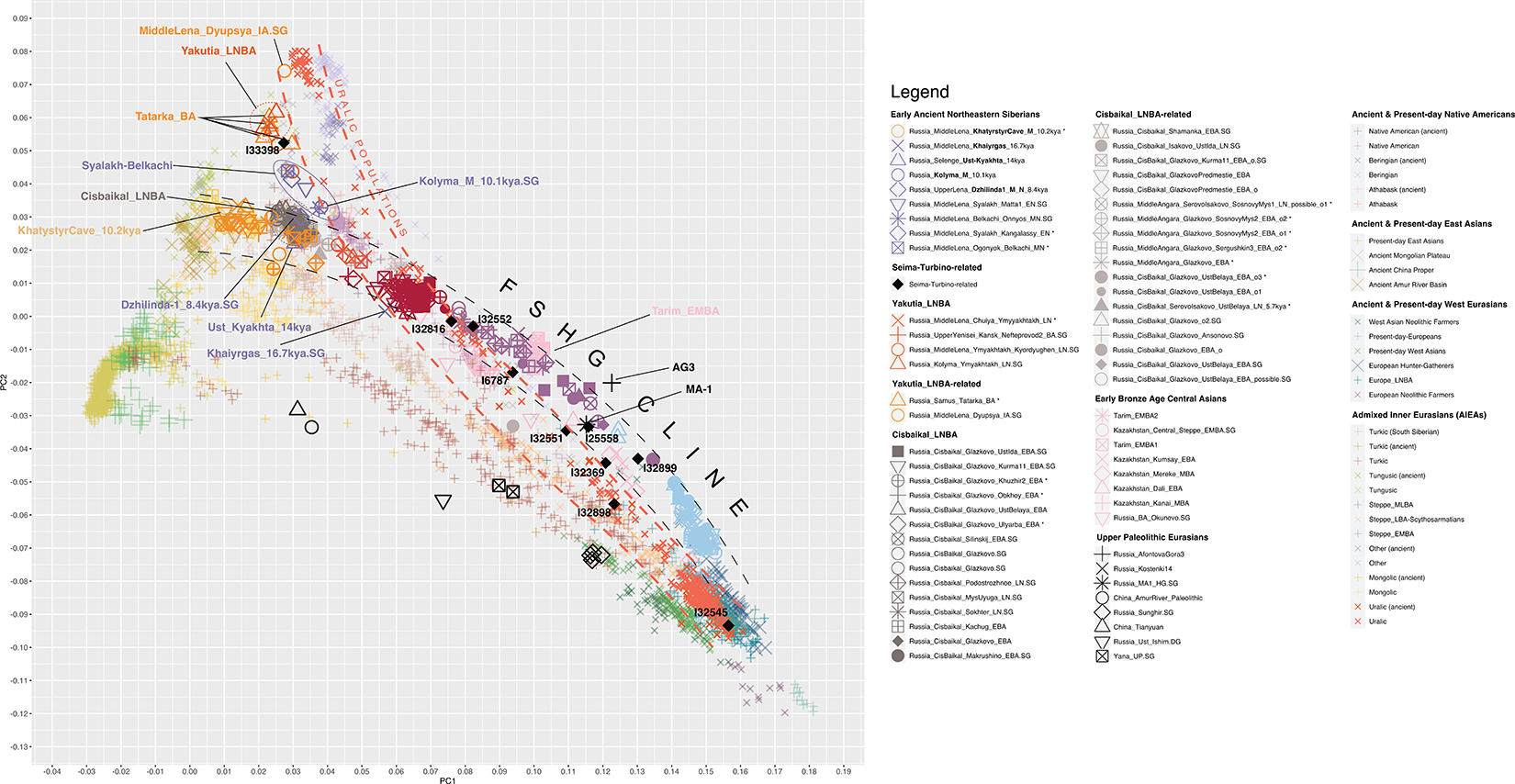
PCA focusing on East Eurasian populations. To further uncover possible structure among the East Asian ancestries within the populations that we analyze, we constructed a third PCA, using as a basis 37 East Asian present-day populations that have minimal West Eurasian admixture, and a single West Eurasian population (Norwegian), all genotyped on the Affymetrix Human Origins array (for a full list of populations analyzed, refer to Supplementary Information section 4). We projected all other shotgun-sequenced and hybridization-captured ancient and present-day individuals onto this basis. Once again, the North Eurasian Hunter-Gatherer cline forms a curved arc stretching from West Eurasian populations to present-day East Asians, with the center of the arc deflected toward the AG3 individual. East Asian populations are now differentiated along PC2, with Southeast Asians and East Asian agriculturalists taking on especially negative values along that dimension; populations from the Amur River Basin taking on intermediate values; then populations on the Mongolian Plateau and surrounding areas. A large gap separates these populations from *Yakutia_LNBA* and Russia_Tatarka_BA, which take on very positive values along PC2, close to present-day Nganasans and a genetically very similar Iron-Age individual from Yakutia who clusters with Nganasans in the previous two PCAs (Yakutia_IA.SG; also see Extended Data Figure 10). As one moves East along the *NEAHG* cline, their positions along PC2 tend to converge to the values found among populations of the Mongolian Plateau. In contrast, the Dzhilinda1_M_N_8.4kya and Kolyma_M_10.1kya individuals, and the Syalakh_Belkachi, *Yakutia_LNBA* and Russia_Tatarka_BA populations do not fall on the *NEAHG* cline and are shifted in the positive direction on PC2, toward the positions occupied by Nganasans, Beringian populations, and Native Americans. Lastly, Uralic populations possess the most positive values among PC2 when compared to Turkic, Mongolic and Tungusic populations.

**Extended Data Figure 5 |:**
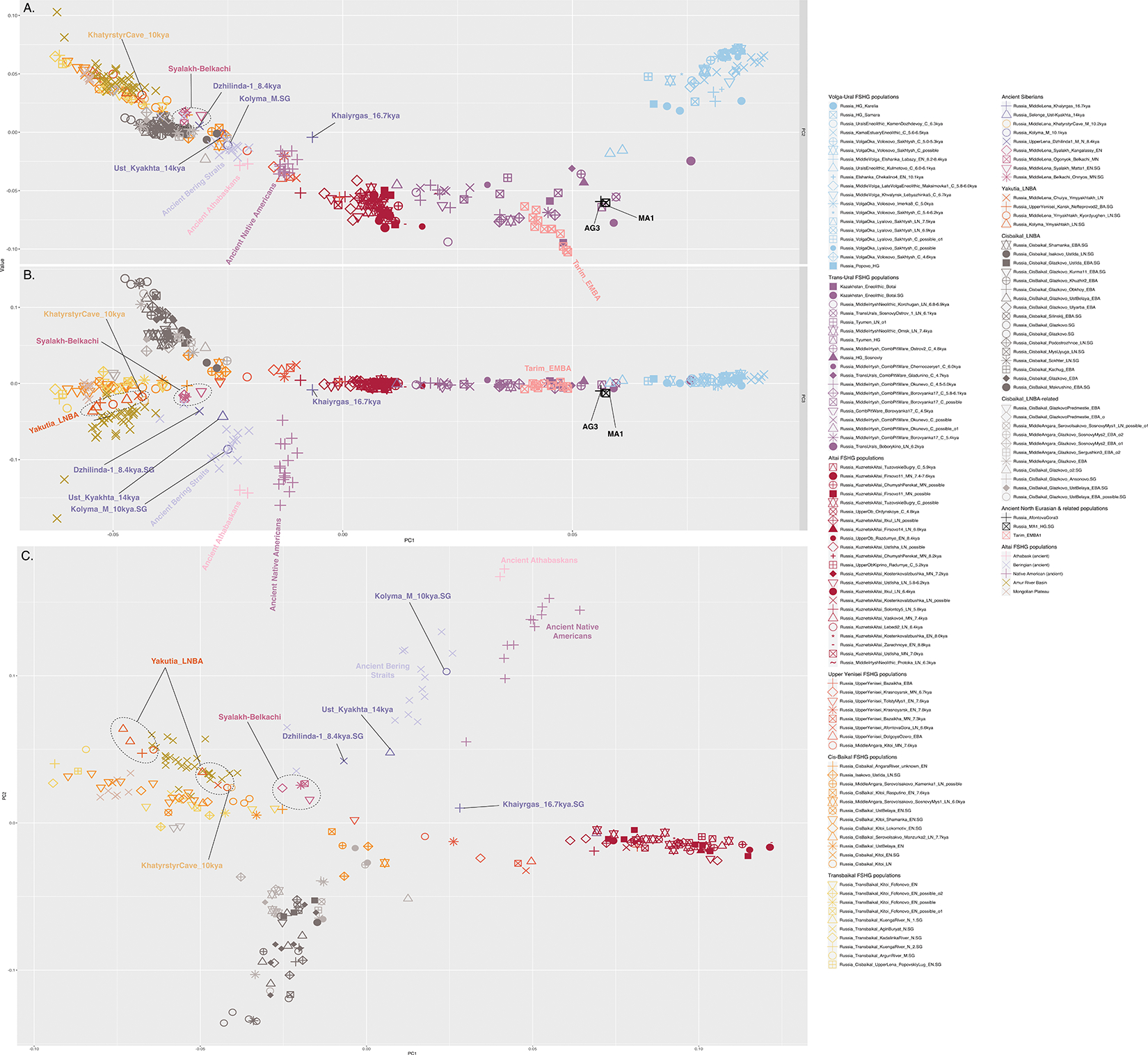
PCA focusing on ancient individuals from Northern Eurasia and the Americas. To understand structure among *NEAHG* populations and non-*NEAHG* Siberians, we constructed two PCAs with ancient individuals including all individuals from the *NEAHG* cline, ancient non-*NEAHG* Siberians, and a selection of ancient Beringians and Native Americans. Notably, all these populations possess combinations of only *WHG*, *EHG*, *ANE* and East Asian ancestries. No individuals were projected in these PCAs. The first PCA (Extended Data Figure 7A) includes all individuals in the set, and the second (Extended Data Figure 7B) includes only individuals East of the Altai mountains. **(A)** In the first PCA we highlight several patterns. 1) the North Eurasian Hunter-Gatherer cline forms a curved arc stretching from West Eurasian populations to East Asian populations along PC1 and PC2. In PC3, populations along the *NEAHG* cline also form a straight line. However, populations rich in East Asian ancestry are differentiated along PC3, with individuals and populations within or closely related to the *Cisbaikal_LNBA* cluster having the most positive values, followed by those in the *Transbaikal_EMN* cluster and populations of the Mongolian Plateau, followed by individuals and populations in the *Yakutia_LNBA* cluster, followed by those from the Amur River Basin, followed by populations from the Bering Straits and the Americas. Notably, all individuals along the *NEAHG* cline, including individuals rich in East Asian ancestry (e.g. *Cisbaikal_EN, Transbaikal_EMN*, and all *NEAHG* individuals from the Krasnoyarsk region) form a straight line in PC3, suggesting a constant source of East Asian ancestry at the East Asian terminus of the *NEAHG* cline. *2) Khairygas_16.7kya* occupies a central position among the other groups rich in East Asian ancestry in East Siberia, Beringia and the Americas, suggesting a lack of shared drift with later populations of the Bering region or the Americas. The situation is different for later populations: *Kolyma_M_10.1kya* falls among ancient Beringian populations, while the more East Asian-admixed *Ust-Kyakhta_14kya* and *Dzhilinda1_M_N_8.4kya* occupy a position in between *Syalakh-Belkachi* and ancient Bering Straits populations, with the even more East Asian-admixed *Syalakh-Belkachi* population showing even less of this displacement towards ancient Bering Straits populations. **(B)** We find a similar pattern in the second PCA, except with an opposite ordering of the clusters along PC3. Our results suggest that the distinctions we discover between groupings produced by the clustering analyses in Supplementary Information section 6 can be recovered in PCA analyses aimed at recovering fine-scale structure, despite underlying similarities in deep ancestry in populations in East Siberia, Beringia, and the Americas—all the products of admixture between ANE and East Asian ancestry.

**Extended Data Figure 6 |:**
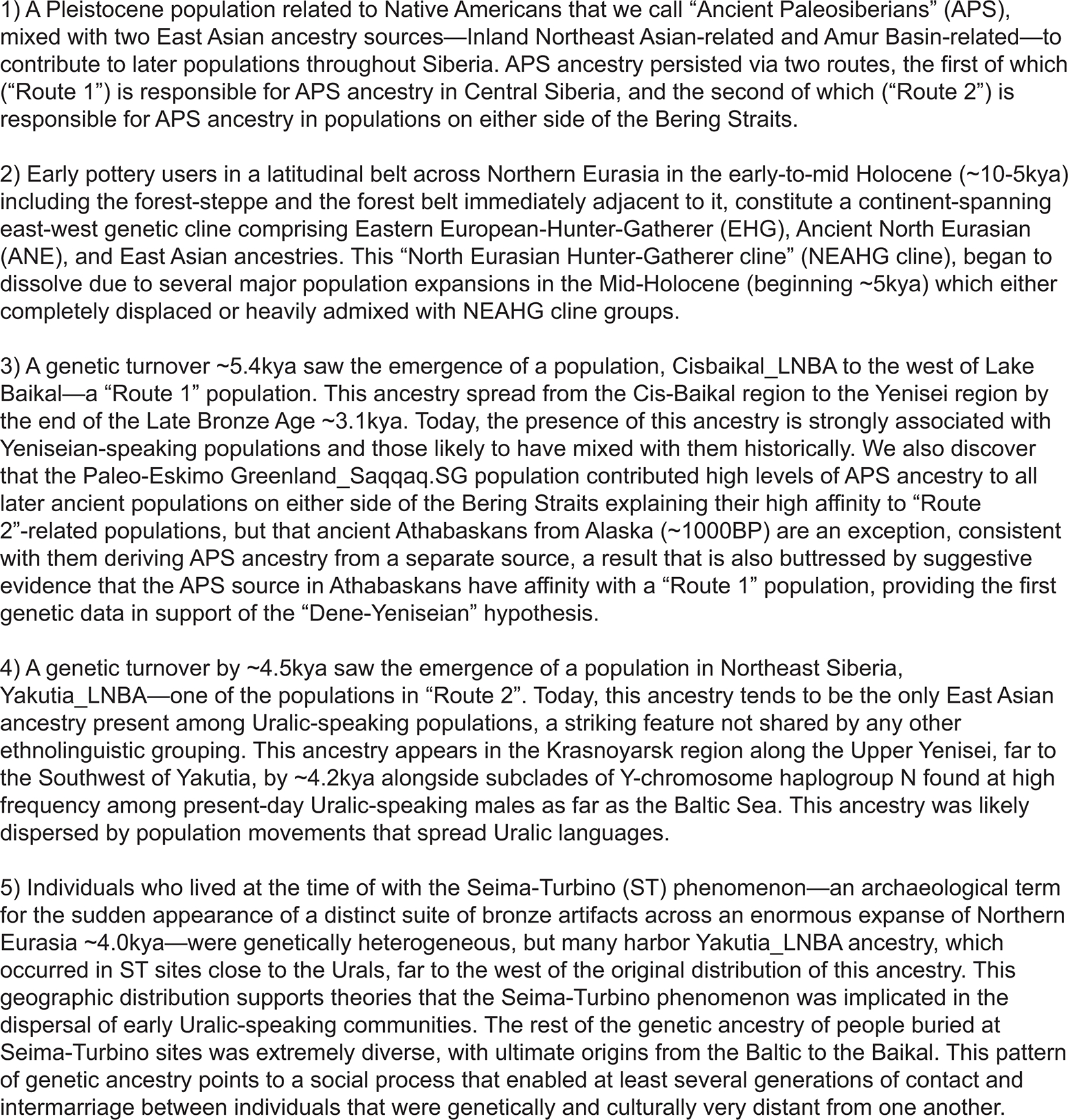
Summary of Genetic Changes Taking Place in Northern Eurasia. Textual summary of our main findings.

**Extended Data Figure 7 |:**
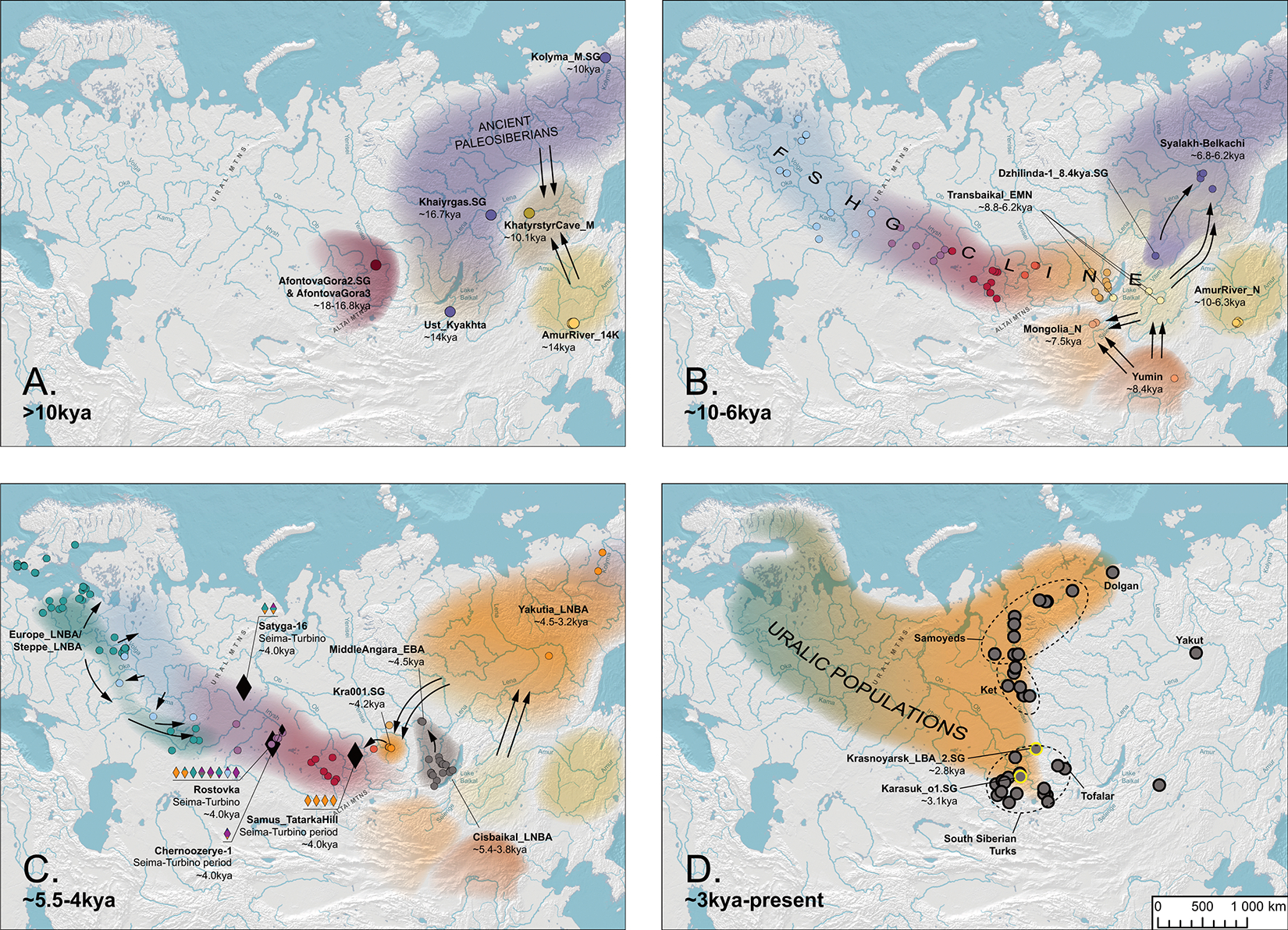
Graphical Summary of Genetic Changes Taking Place in Northern Eurasia. Panel A shows the widespread distribution of individuals with Ancient Paleosiberian (APS) ancestry in Siberia before the Holocene, >10kya. Panel B shows the formation of the NEAHG cline by ~10kya, and the formation of the population on its eastern terminus (Transbaikal_EMN) through admixture between Amur River and Inland East Asian ancestries. Panel C shows the emergence of Cisbaikal_LNBA and Yakutia_LNBA in genetic turnovers in the Cis-Baikal and Northeastern Siberian regions in the Mid-Holocene, and the genetic diversity of Seima-Turbino period individuals ~4.0kya. Panel D shows the genetic gradient between West Eurasian ancestry and Yakutia_LNBA formed by present-day Uralic populations, along with all locations from which present-day populations with Cisbaikal_LNBA ancestry were sampled (grey dots ringed with black), alongside the geographic locations of two late Bronze Age/early Iron Age individuals (grey dots ringed with yellow) with >90% Cisbaikal_LNBA ancestry.

**Extended Data Figure 8 |:**
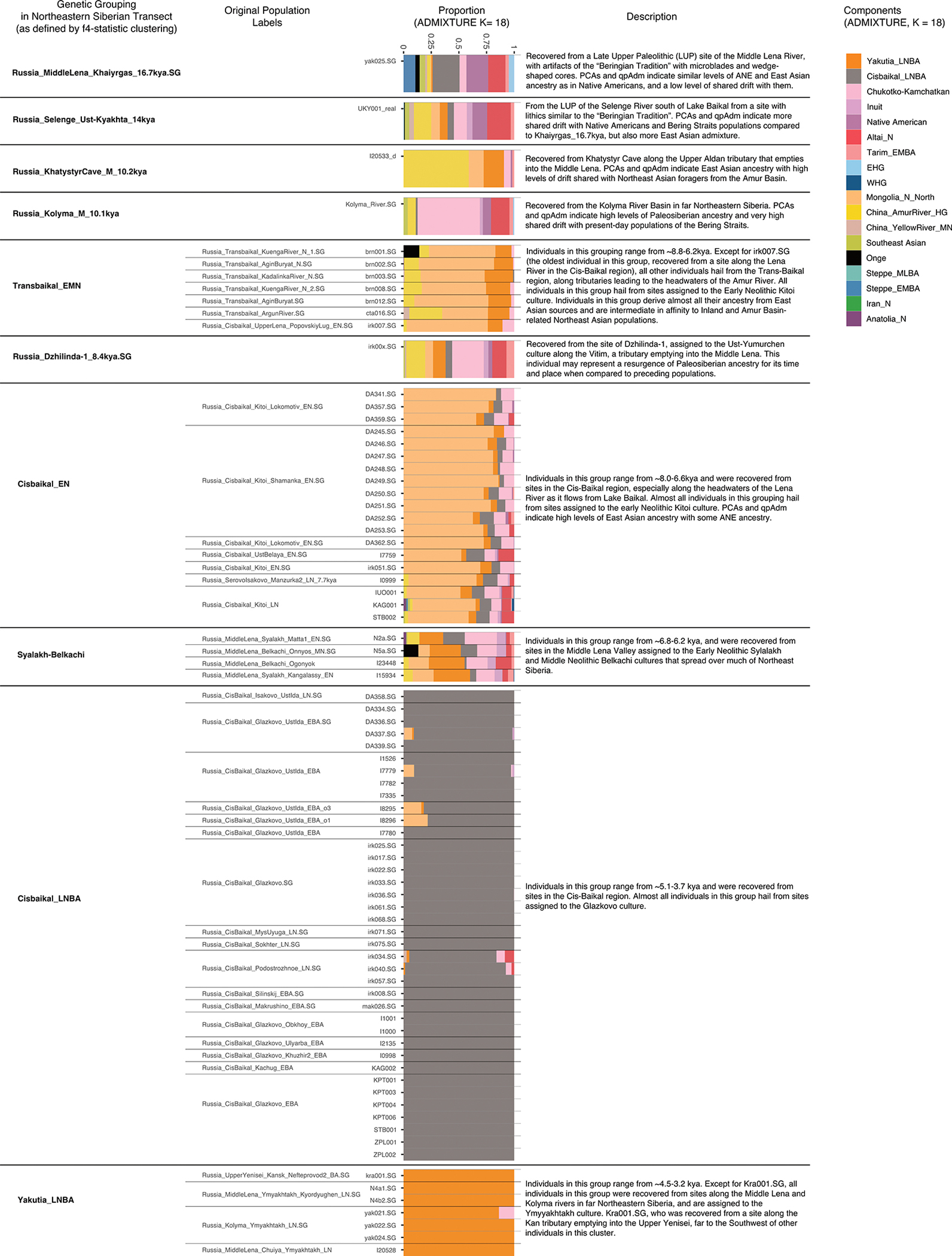
Populations created by genetic grouping procedure applied over Northeast Siberians. Details of populations created by the grouping procedure applied to individuals in Northeastern Siberia.

**Extended Data Figure 9 |:**
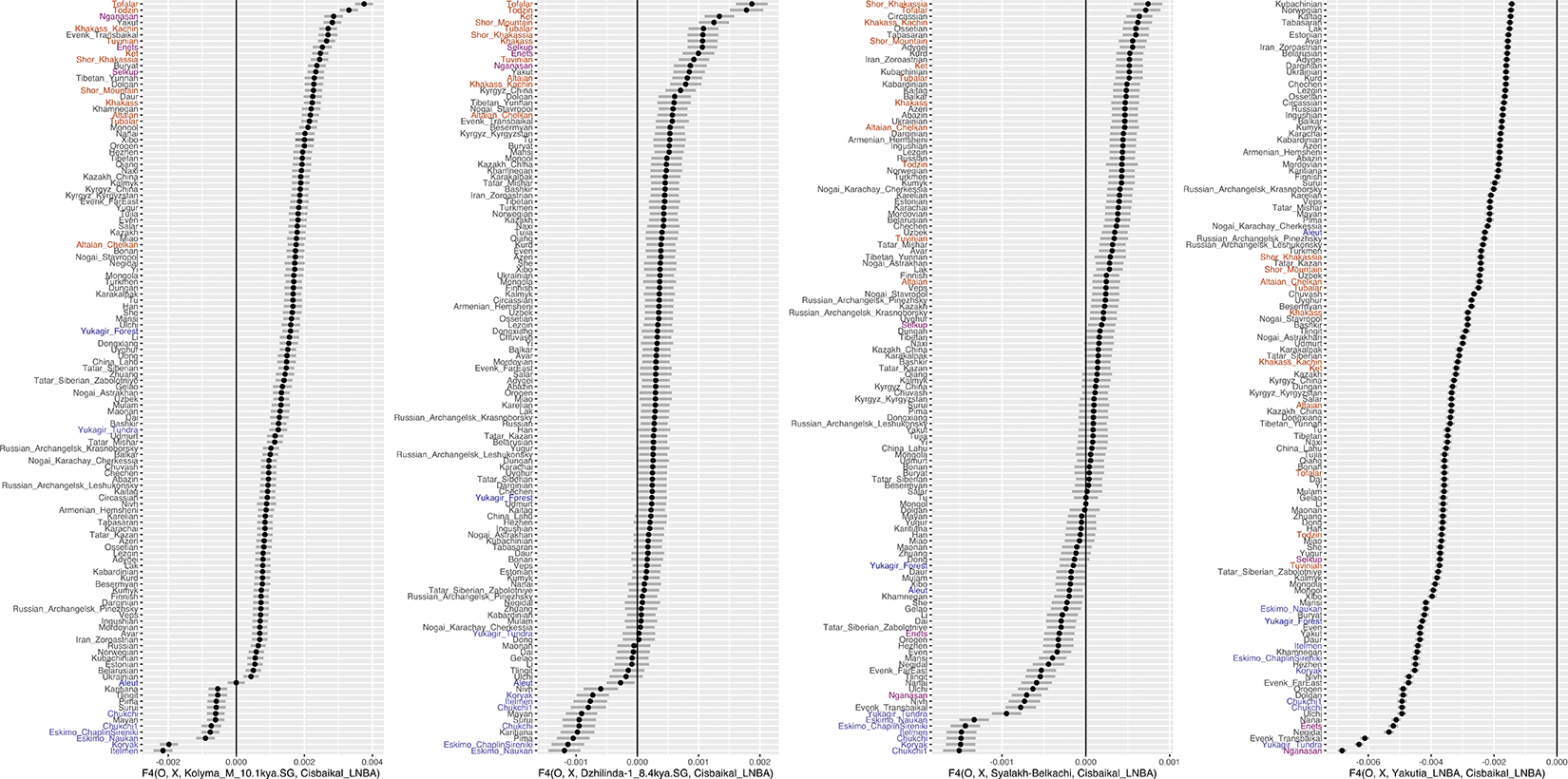
Statistics of the form f4(Ethiopia_4500BP.SG, Target, “Route 2” population, Cisbaikal_LNBA). Central Siberian populations from the Yenisei Basin (including Kets and South Siberian Turks) are highlighted in brown, while Arctic North American and Asian populations on either side of the Bering Straits populations are highlighted in blue. Bering Straits populations that are heavily European-admixed (Aleut and Yukagir_forest) are colored dark blue, while Samoyedic populations (Enets, Selkup, and Nganasan) are colored violet. Despite the similarity of the APS-rich populations in this comparison (all being admixtures between APS ancestry and East Asian ancestry), present-day groups of the Bering Straits are always closer to groups with “Route 2” APS ancestry (i.e., Kolyma_M_10.1kya → Dzhilinda1_8.4kya → Syalakh-Belkachi → Yakutia_LNBA), while Central Siberian populations of the Yenisei Basin are always closer to Cisbaikal_LNBA. For the version including a comparison with Ust-Kyakhta, refer to Supplementary Information section 8; Figures S94 & S95.

**Extended Data Figure 10 |:**
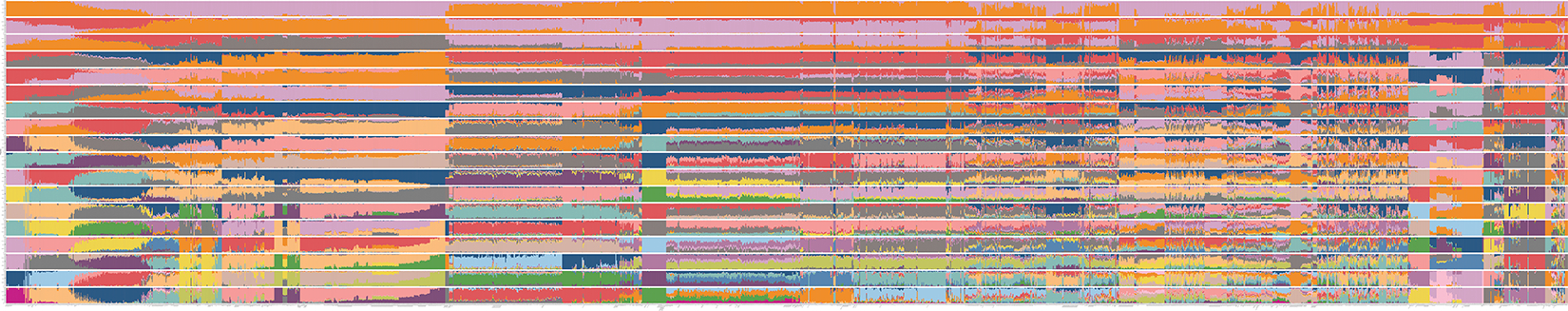
ADMIXTURE results. For details, refer to Supplementary Information section 5.

**Extended Data Figure 11 |:**
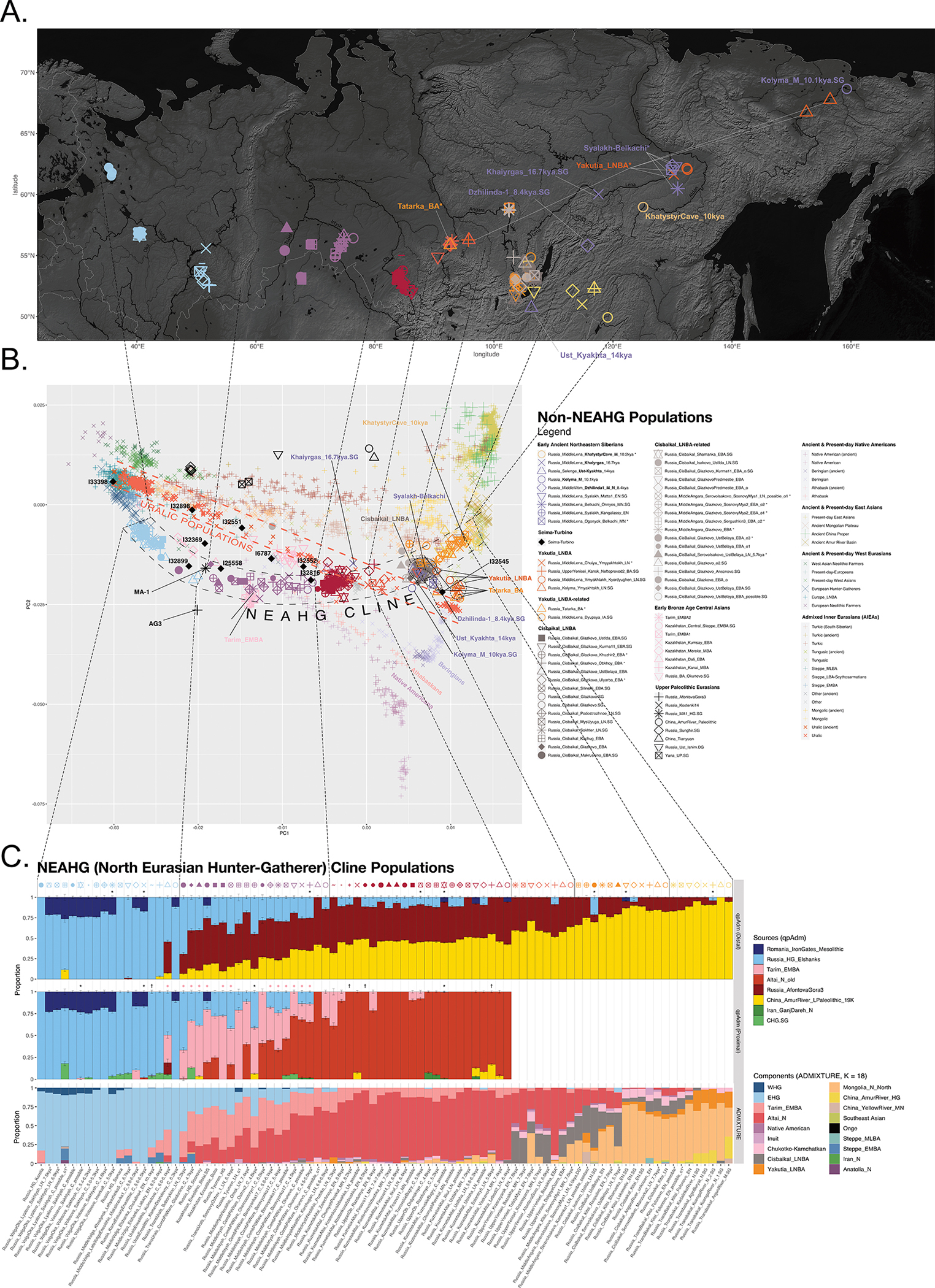
The North Eurasian Hunter-Gatherer (NEAHG) Cline and its legacy through admixture in ancient northern Eurasia. Higher-resolution version of Figure 1, containing the group/population labels of Figure 1C.

**Extended Data Figure 12 |:**
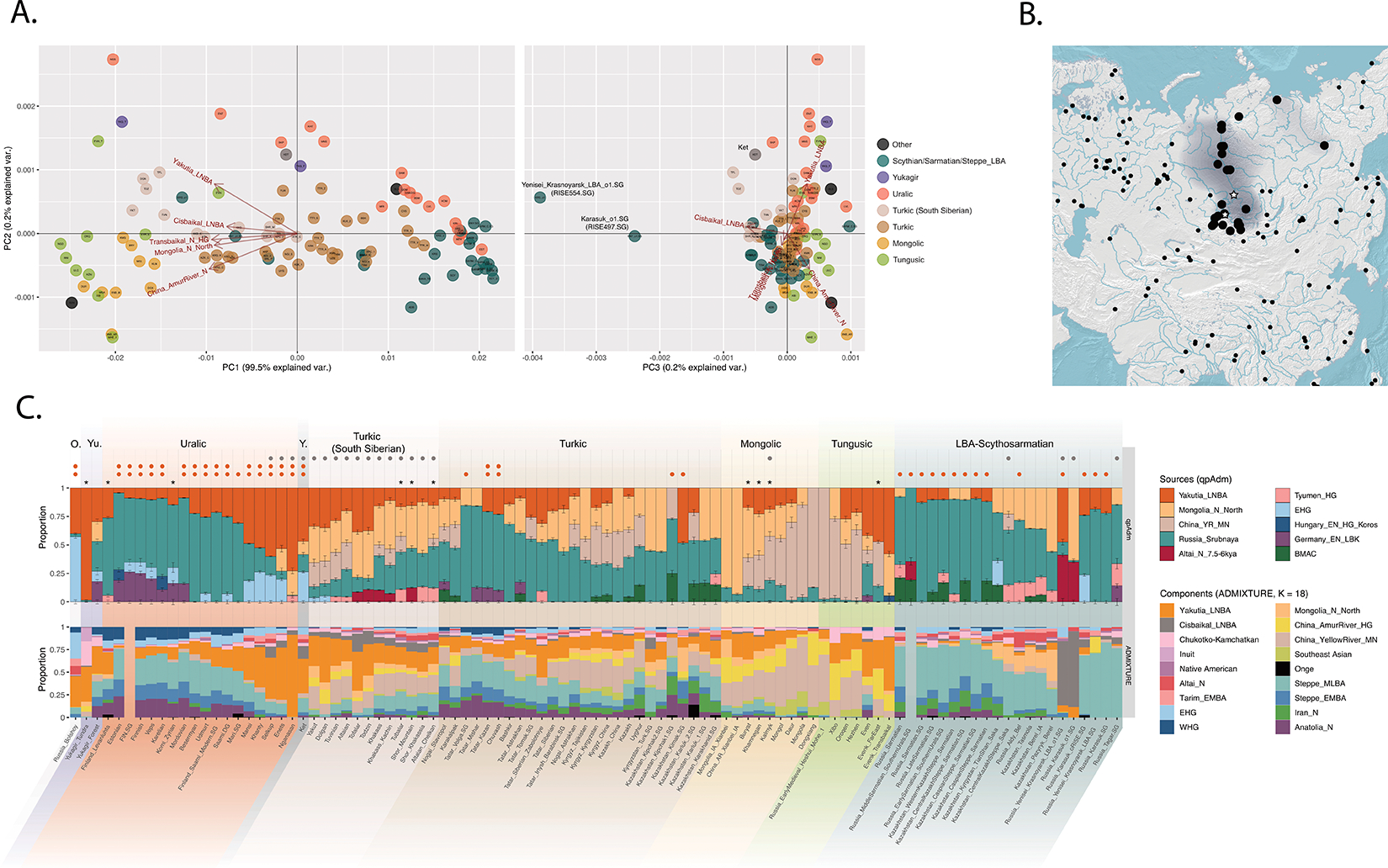
Contribution of Yakutia_LNBA and Cisbaikal_LNBA to Admixed Inner Eurasians (AIEA). Higher-resolution version of Figure 3, containing the group/population labels of Figure 3C, and three-letter population codes in Figure 3A. The codes are: ATN, Altaian; ATN_C, Altaian_Chelkan; BSK, Bashkir; BSM, Besermyan; BRY, Buryat; XNB_AR, China_AR_Xianbei_IA; CVS, Chuvash; DUR, Daur; DGN, Dolgan; DGX, Dongxiang; ENT, Enets; EST, Estonian; EVN, Even; EVN_E, Evenk_FarEast; EVN_T, Evenk_Transbaikal; FIN.SG, FIN.SG; LVL, Finland_Levanluhta; SAM, Finland_Saami_Modern.SG; FIN, Finnish; HZN, Hezhen; KLM, Kalmyk; KKP, Karakalpak; KRL, Karelian; KZK, Kazakh; KZK_C, Kazakh_China; BRL, Kazakhstan_Berel_IA; SARM_C, Kazakhstan_CaspianSteppe_Sarmatian; SARM_C.SG, Kazakhstan_CaspianSteppe_Sarmatian.SG; SAKA_K, Kazakhstan_CentralKazakhSteppe_Saka; SARM_K, Kazakhstan_CentralKazakhSteppe_Sarmatian.SG; KRK, Kazakhstan_Karakhanid.SG; KLK_1, Kazakhstan_Karluk_1.SG; KLK_2, Kazakhstan_Karluk_2.SG; KMK, Kazakhstan_Kimak.SG; KPC_1, Kazakhstan_Kipchak1.SG; KPC_2, Kazakhstan_Kipchak2.SG; SAKA_TS, Kazakhstan_Kyrgystan_TianShan_Saka; BRL_P, Kazakhstan_Pazyryk_Berel; TSM, Kazakhstan_Tasmola; SARM_W, Kazakhstan_WesternKazakhSteppe_Sarmatian; KET, Ket; KKS, Khakass; KKS_K, Khakass_Kachin; KMG, Khamnegan; KHT, Khanty; KOM, Komi_Zyrian; KRG_C, Kyrgyz_China; KRG_K, Kyrgyz_Kyrgyzstan; KRG_T, Kyrgyz_Tajikistan; TUR, Kyrgyzstan_Turk.SG; MNS, Mansi; MRI, Mari.SG; SCY, Moldova_Scythian; MGL, Mongol; MGA, Mongola; XNB_M, Mongolia_IA_Xianbei; MDV, Mordovian; NNI, Nanai; NGD, Negidal; NGS, Nganasan; NVH, Nivh; NGI_A, Nogai_Astrakhan; NGI_K, Nogai_Karachay_Cherkessia; NGI_S, Nogai_Stavropol; ORQ, Oroqen; ADB, Russia_Aldy_Bel; BLS, Russia_Bolshoy; MHE_1, Russia_EarlyMedieval_Heshui_Mohe_1; MHE_2, Russia_EarlyMedieval_Heshui_Mohe_2; SARM_S, Russia_EarlySarmatian_SouthernUrals.SG; KRS_o1, Russia_Karasuk_o1.SG; KRS_o, Russia_Karasuk_oRISE.SG; KRS, Russia_Karasuk.SG; SARM_L, Russia_LateSarmatian.SG; SARM_S.SG, Russia_MiddleSarmatian_SouthernUrals.SG; SARM, Russia_Sarmatian; SARM.SG, Russia_Sarmatian.SG; TGR, Russia_Tagar.SG; SAM.DG, Saami.DG; SKP, Selkup; SHR_K, Shor_Khakassia; SHR_M, Shor_Mountain; TTR_A, Tatar_Astrakhan; TTR_I, Tatar_Irtysh_Barabinsk.SG; TTR_K, Tatar_Kazan; TTR_M, Tatar_Mishar; TTR_S, Tatar_Siberian; TTR_Z, Tatar_Siberian_Zabolotniye; TTR_T, Tatar_Tomsk.SG; TTR_V, Tatar_Volga.SG; TDZ, Todzin; TFL, Tofalar; TBL, Tubalar; TKM, Turkmen; TVN, Tuvinian; UDM, Udmurt; SCY_U, Ukraine_Scythian; ULC, Ulchi; UYG, Uyghur; UZB, Uzbek; VPS, Veps; XIB, Xibo; YKT, Yakut; YKG_F, Yukagir_Forest; YKG_T, Yukagir_Tundra; KNY.SG, Russia_Yenisei_Krasnoyarsk_LBA.SG; KNY_o1.SG, Russia_Yenisei_Krasnoyarsk_LBA_o1.SG.

**Extended Data Figure 13 |:**
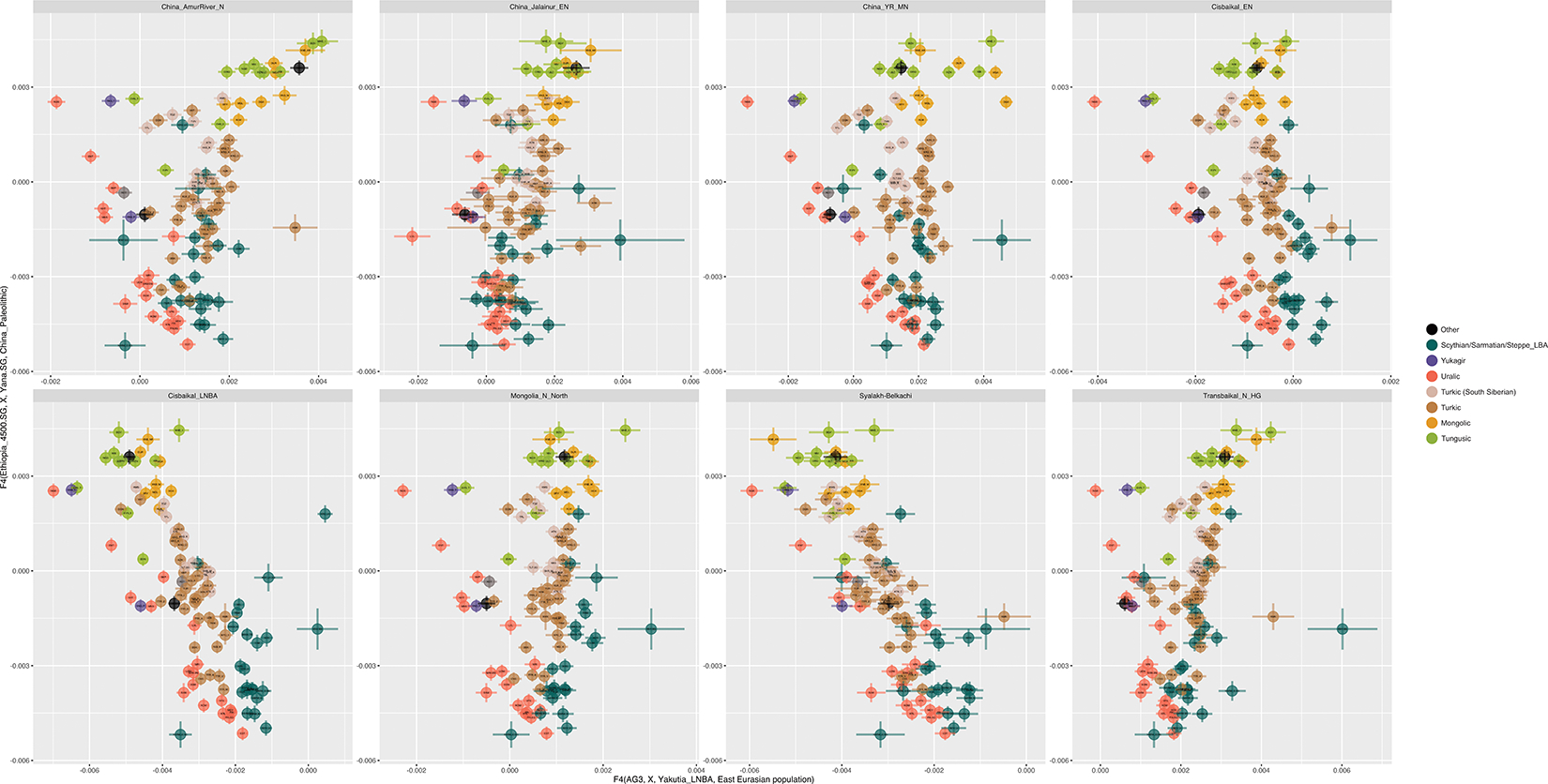
f4 statistics of the form f4(*Ethiopia_4500BP.SG, X, Yana.SG, China_Paleolithic*) plotted against f4(*AG3, X, Yakutia_LNBA, East Eurasian Population*). *China_Paleolithic* includes the Tianyuan and Amur_River_33K genomes, “East Eurasian Population” is some population grouping in Siberia or Northeast Asia other than *Yakutia_LNBA*, and X are Admixed Inner Eurasian populations (AIEA populations) including ancient Central Asian nomads from the Late Bronze to Iron Age down to the Scytho-Sarmatian period, as well as modern or ancient populations that speak languages from the Yukaghiric, Yeniseian (Kets), Uralic, Turkic, Mongolic, Tungusic, and Nivkh language families. Modern Uralic-speaking populations, and ancient putatively Uralic-speaking populations uniformly prefer *Yakutia_LNBA* to other East Asian ancestries no matter the other population used in the comparison. Furthermore, at any level of admixture between East and West Eurasian ancestries, the population with the greatest affinity to *Yakutia_LNBA* is always a Uralic-speaking population. f4-statistics therefore highlight the connection between Uralic populations and Yakutia_LNBA ancestry over other sources of East Asian ancestry.

**Extended Data Figure 14 |:**
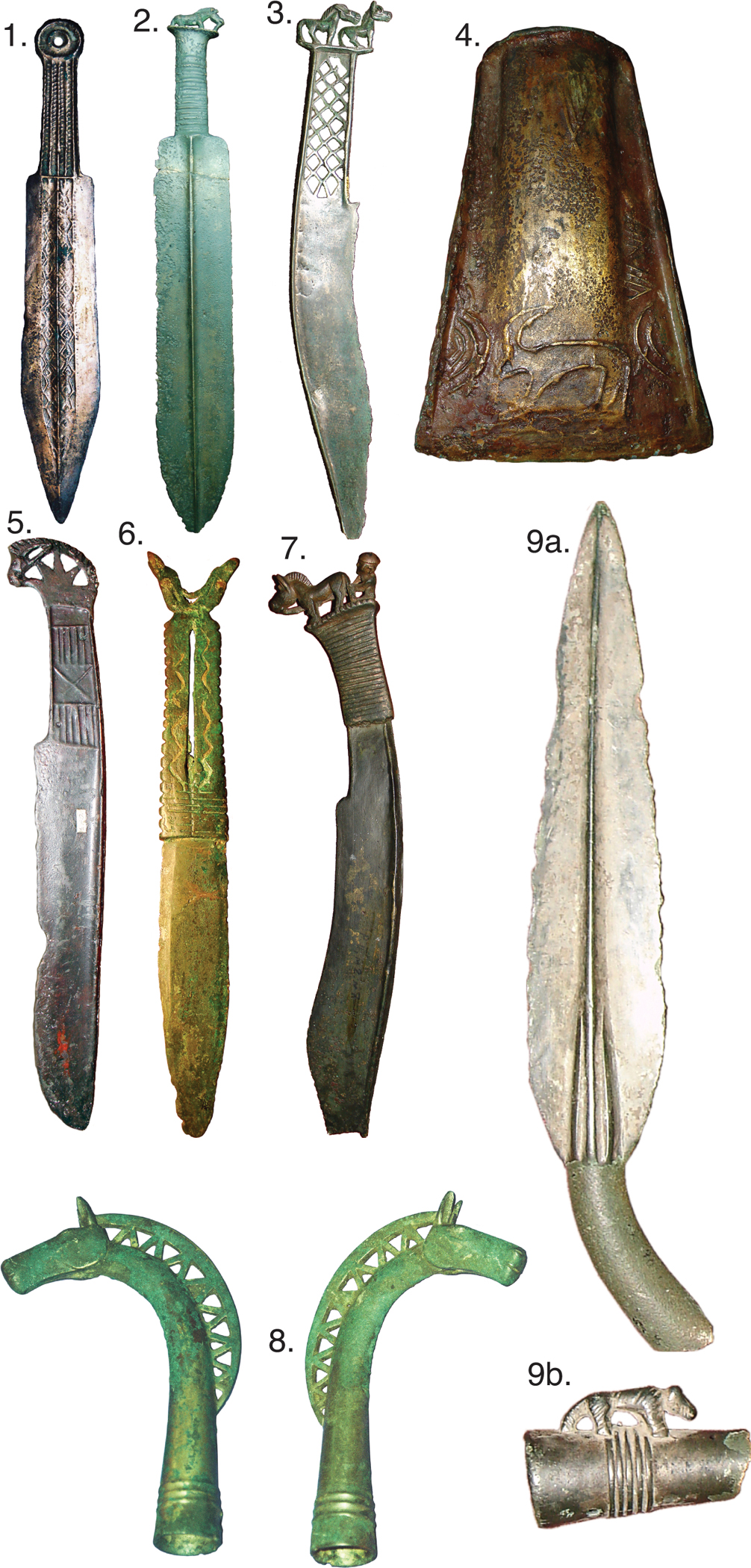
Characteristic Seima-Turbino artifacts. 1. Double-bladed dagger with a ring-shaped pommel, robbery find, unknown provenance (probable Omsk region or Rostovka). 2. Double-bladed dagger with a horse figurine on the pommel, an accidental find near Shemonaikha, East Kazakhstan. 3., 5., 7. Crook-backed knives with figurines on pommels: 3. from Seyma; 5. from Elunino-1, burial 1, 7. from Rostovka, burial 2. 4. Scapula-shaped celt with goat image, Rostovka, cluster of finds near burial 21. 6. double-bladed plate dagger with a double elk-head figurine pommel, an accidental find near Perm’ (probably associated with the Turbino site). 8. Top of staff with a horse figurine, an accidental find near Omsk. 9a. & 9b. Single-ear long spearhead with a relief figurine of a *Felidae* predator (tiger or mountain leopard) on the socket (9a. the spear tip,10b. the detail of the socket), an accidental find near Omsk.

**Extended Data Table 1 |:** Summary of qpAdm analyses. This table provides information about the Supplementary Data files that should be referred to in order to understand the details of each qpAdm analysis. There are four major rounds of qpAdm analysis (NEAHG, Seima-Turbino, AIEA populations, and 10-member Siberian transect plus Bering Straits populations). These qpAdm analyses vary in their goals. They may also involve different data types (such as 1240k data only, or mixture of 1240k and shotgun), and their analytic setup.

qpAdm analyses	Purpose	Associated Section of Supplementary Material	Associated Figures displaying results	Individuals included in f2-statistic calculation and their population labels for that set of qpAdm	Number of SNPs retained after f2blocks calculation	Supplementary Data file with full results	Wet laboratory data type
qpAdms targeting NEAHG populations (proximal)	Investigating the origin of the ANE ancestry in NEAHG populations	Supplementary Information section 10	Figure 1C, bottom row	Listed in Supplementary Data 1, in the sheet “Population Labels of Ancients” Column D	1196712	Supplementary Data 2, in the sheet “Proximal NEAHG qpAdms”	References: 1240K only Sources: 1240K only Targets: 1240K & shotgun
qpAdms targeting NEAHG populations (distal)	Investigating the deep genetic affinities of NEAHG populations	Supplementary Information section 10	Figure 1C, Middle Row	Listed in Supplementary Data 1, in the sheet “Population Labels of Ancients”, Column D	1135474	Supplementary Data 2, in the sheet “Distal NEAH qpAdms”	References: 1240K only Sources: 1240K only Targets: 1240K and shotgun
qpAdms targeting Seima-Turbino individuals (proximal models)	Investigating the proximal ancestry sources of Seima- Turbino individuals	Supplementary Information section 15	Figure 4B, bottom row	Listed in Supplementary Data 1, in the sheet “Population Labels of Ancients”, Column O	1135472	Supplementary Data 7, in the sheet “Proximal ST qpAdms”	References: 1240K & shotgun Sources: 1240K & shotgun Targets: 1240K & shotgun
qpAdms targeting Seima-Turbino individuals (distal models)	Investigating the distal ancestry sources of Seima-Turbino individuals	Supplementary Information section 15	Figure 4B, Middle Row	Listed in Supplementary Data 1, in the sheet “Population Labels of Ancients”, Column N	1196712	Supplementary Data 7, in the sheet “Distal ST qpAdms”	References: 1240K & shotgun Sources: 1240K & shotgun Targets: 1240K & shotgun
qpAdms targeting AIEA populations, and Seima- Turbino individuals (distal models)	Investigating the ancestry sources of AIEA populations (with China_AmurRiver _N and without China_AmurRiver _14K in the sources and references)	Supplementary Information sections 11,13,15	Figure 3C	Listed in Supplementary Data 1, in the sheet “Population Labels of Ancients”, Column M	593124	Supplementary Data 6, in the paired sheets “AIEA Yakutia_LNBA qpAdms" with passing models in "Results (Yakutia_LNBA)”; and in the paired sheets “AIEA Cisbaikal_LNBA qpAdms” with passing models in “Results (Cisbaikal_LNBA, 1240k)”	References: 1240K & shotgun Sources: 1240K & shotgun Targets: 1240K & shotgun
qpAdms targeting AIEA populations (1240K populations used in sources and references only)	Investigating the ancestry sources of AIEA populations, with only 1240K sequences in both sources and references	Supplementary Information sections 11,13,15	-	Listed in Supplementary Data 1, in the sheet “Population Labels of Ancients”, Column P	546292	Supplementary Data 6, in the paired sheets “AIEA Yakutia_LNBA qpAdms 1240k" with passing models in "Results (Yakutia_LNBA 1240k)”; and in the paired sheets “AIEA Cisbaikal_LNBA qpAdms1240k” with passing models in “Results (Cisbaikal_LNBA)”	References: 1240K only Sources: 1240K only Targets: 1240K & shotgun
qpAdms targeting populations in 10- member Siberian transect	Investigating the genetic origins of a ten-member transect of Siberian population history	Supplementary Information section 8	Figure 2C	Listed in Supplementary Data 1, in the sheet “Population Labels of Ancients”, Column Q	1196712	Supplementary Data 6, in the sheet “AIEA Yakutia_LNBA + Amur”	References: 1240K & shotgun Sources: 1240K & shotgun Targets: 1240K & shotgun
qpAdms targeting Bering Straits Populations	Investigating the genetic relationship between Bering Straits populations and the populations in the ten-member transect	Supplementary Information section 9	-	Listed in Supplementary Data 1, in the sheet “Population Labels of Ancients”, Column R	1135467	Supplementary Data 5, Table, in the sheets “Saqqaq qpAdms” and “Beringian qpAdms (NNA + SNA)”	References: 1240K & shotgun Sources: 1240K & shotgun Targets: 1240K & shotgun

## Supplementary Material

Supplementary Information

Supplementary Information Guide

Supplementary Data 1

Supplementary Data 2

Supplementary Data 3

Supplementary Data 4

Supplementary Data 5

Supplementary Data 6

Supplementary Data 7

## Figures and Tables

**Table 1 |:** Glossary of Acronyms

Term	Usage	Meaning
_M_	Term used to designate the Archaeological Period in population labels	Mesolithic
_N_	Term used to designate the Archaeological Period in population labels	Neolithic. Note that in Russian archaeological literature and in the archaeology of much of Northern Eurasia, the Neolithic period is defined by the presence of pottery, and not of agriculture or domesticated animals.
_EN_, _MN_, _EMN_	Term used to designate the Archaeological Period in population labels	Early Neolithic, Middle Neolithic, Early and Middle Neolithic
_BA_, _EBA_, _LBA_, _MLBA_, _LNBA_	Term used to designate the Archaeological Period in population labels	Bronze Age, Early Bronze Age, Late Bronze Age, Middle and Late Bronze Age, Late Neolithic and Bronze Age
APS	Acronym used to refer to an ancestry type	**Ancient Paleosiberian Ancestry**—A term referring to an ancient Siberian population related to the ancestors of Native American populations, who admixed into all later Eastern and Central Siberian populations as well as present-day populations on either side of the Bering Straits
NEAHG	Acronym	**North Eurasian Hunter-Gatherers**—A term designating a belt of hunter-gatherer populations spanning Northern Eurasia in the first half of the Holocene.
AIEA	Acronym	**Admixed Inner Eurasians**—A term designating all populations in Central and Northern Eurasia that are the product of Holocene admixtures between West Eurasian ancestries and East Asian ancestries, including present-day and ancient Mongolic, Turkic, Tungusic, and Uralic populations, as well as ancient Scythians, Sarmatians and pre-Scythian nomads of the Iron Age Steppes.

## Data Availability

The newly reported data in this study can be obtained from the European Nucleotide Archive under accession number PRJEB86428. Bam files of aligned reads for the 180 newly published ancient individuals and 15 newly reported whole genome sequences from a subset of these individuals can be found at secondary accession ERP169776, while the genotypes we used for analysis can be found at secondary accession ERZ25719453. Genotype files in PLINK format for the 229 modern individuals for whom we newly report SNP array can be found at secondary accession ERZ26790638. All maps in the main text and in the Supplementary Information were created using ArcGIS 10.6.1 and QGIS 3.40.6. Figures presenting genetic data were created using Rstudio running R version 4.4.1, and further edited in Adobe Illustrator version 28. Archaeological images in Supplementary Information section 3 were edited in Adobe Photoshop 25.12.2 and Adobe Acrobat 2025.001.20458.
