# Supplementary material for: Ancient DNA reveals the prehistory of the Uralic and Yeniseian peoples
Source: Nature. Author manuscript; Available in PMC 2025 Aug 12. (PMC12342343; doi:10.1038/s41586-025-09189-3)
Supplement: Supplementary Information [file NIHMS2095973-supplement-Supplementary_Information.docx]

Supplementary Information

**Ancient DNA reveals the prehistory of the Uralic and Yeniseian peoples**

# Table of Contents

[Table of Contents 1](#_Toc197475405)

[1. Geophysical context 6](#_Toc197475406)

[1.1. Geographic terms 6](#_Toc197475407)

[1.2. Climate change during the Late Pleistocene and Early Holocene 12](#_Toc197475408)

[1.2.1. Late Pleistocene Northern Asia 12](#_Toc197475409)

[1.2.2. Early Holocene Northern Asia 12](#_Toc197475410)

[1.2.3. Middle Holocene Northern Asia 13](#_Toc197475411)

[1.3. References (for Supplementary Information section 1) 15](#_Toc197475412)

[2. Radiocarbon Dating and Stable Isotope Analysis 16](#_Toc197475413)

[2.1. References (for Supplementary Information section 2.) 19](#_Toc197475414)

[3. Archaeological Context 20](#_Toc197475415)

[3.1. Eastern Part of Central Siberia 20](#_Toc197475416)

[3.1.1. Middle Lena 20](#_Toc197475417)

[3.1.1.1. Sumnagin Mesolithic culture in the Middle Lena Region & the Dyuktai culture 20](#_Toc197475418)

[3.1.1.1.1. Khatyrstyr Cave site 21](#_Toc197475419)

[3.1.1.2. Syalakh Neolithic culture (Russia_MiddleLena_SyalakhBelkachi_EN) 22](#_Toc197475420)

[3.1.1.2.1. Kangalassy site 23](#_Toc197475421)

[3.1.1.3. Belkachi Neolithic culture (Russia_MiddleLena_Belkachi_N) 25](#_Toc197475422)

[3.1.1.3.1. Ongonyok site 26](#_Toc197475423)

[3.1.1.4. Ymyyakhtakh Neolithic culture (Russia_MiddleLena_Ymyyakhtakh_LN) & Siberian Bone Armor 28](#_Toc197475424)

[3.1.1.4.1. Chuiya site 29](#_Toc197475425)

[3.2. South part of Central Siberia and the Baikal Region 29](#_Toc197475426)

[3.2.1. Transbaikalia, Cisbaikalia, and Upper Angara 29](#_Toc197475427)

[3.2.1.1. Early Neolithic.(Russia_Fofonovo_Kitoi_EN) and Bronze Age (Russia_Fofonovo_Glazkovo) in the Transbaikalia 29](#_Toc197475428)

[3.2.1.1.1. Fofonovo burial site 29](#_Toc197475429)

[3.2.1.2. Kitoi and other Early Neolithic burial traditions (Russia_Cisbaikal_Kitoi_MN) 30](#_Toc197475430)

[3.2.1.2.1. Rasputino site 30](#_Toc197475431)

[3.2.1.2.2. Manzurka-2 site 31](#_Toc197475432)

[3.2.1.3. Serovo and Isakovo Neolithic burial traditions (Russia_Cisbaikal_SerovoIsakovo_N and Russia_AngaraRiver_SerovoIsakovo_N) 33](#_Toc197475433)

[3.2.1.3.1. Ust’-Belaya site complex 33](#_Toc197475434)

[3.2.1.4. Glazkovo Early Bronze Age culture (Russia_Cisbaikal_Glazkovo_EBA and Russia_AngaraRiver_Glazkovo_EBA) 33](#_Toc197475435)

[3.2.1.4.1. Obkhoy burial ground 33](#_Toc197475436)

[3.2.1.4.2. Ulyarba site 34](#_Toc197475437)

[3.2.1.4.3. Khuzhir site 38](#_Toc197475438)

[3.2.2. Lower and Middle Angara 38](#_Toc197475439)

[3.2.2.1. Early and Middle Neolithic sites (Russia_MiddleAngara_Kitoi_MN) 38](#_Toc197475440)

[3.2.2.1.1. Sosnovy Mys site complex 38](#_Toc197475441)

[3.2.2.2. Serovo and Isakovo Neolithic burial tradition (Russia_MiddleAngara_SerovoIsakovo_LN) 39](#_Toc197475442)

[3.2.2.2.1. Sosnovy Mys site complex 39](#_Toc197475443)

[3.2.2.2.2. Kamenka-1 site 40](#_Toc197475444)

[3.2.2.3. Glazkovo Early Bronze Age culture along the Middle Angara (Russia_MiddleAngara_Glazkovo_EBA) 42](#_Toc197475445)

[3.2.2.3.1. Sergushkin Ostrov site complex 42](#_Toc197475446)

[3.2.2.3.2. Ust’-Shamanka-1 site 43](#_Toc197475447)

[3.2.2.3.3. Sosnovy-Mys site complex 43](#_Toc197475448)

[3.3. South part of West Siberia and the Altai-Sayan 44](#_Toc197475449)

[3.3.1. Yenisei River Basin 44](#_Toc197475450)

[3.3.1.1. Yenisei Forest-Steppe Neolithic (Russia_Krasnoyarsk_N and Russia_UpperYenisei_N) 44](#_Toc197475451)

[3.3.1.1.1. Afontova Gora Neolithic burials 44](#_Toc197475452)

[3.3.1.1.2. Tolsty Mys-1 45](#_Toc197475453)

[3.3.1.1.3. Krasnoyarsk finds 45](#_Toc197475454)

[3.3.1.2. Yenisei Forest-Steppe Eneolithic/EBA (Russia_UpperYenisei_EBA) 46](#_Toc197475455)

[3.3.1.2.1. Dolgoye Ozero burial ground 46](#_Toc197475456)

[3.3.2. The Upper Ob and Kuznetsk Depression 47](#_Toc197475457)

[3.3.2.1. Kuznetsk-Altai Neolithic/Upper Ob Neolithic/Bolshoy Mys Eneolithic sites (Russia_KuznetskAltai) 49](#_Toc197475458)

[3.3.2.1.1. Razdum’ye-1 site 49](#_Toc197475459)

[3.3.2.1.2. Zarechnoye-1 site 49](#_Toc197475460)

[3.3.2.1.3. Firsovo-11 burial ground 50](#_Toc197475461)

[3.3.2.1.4. Chumysh-Perekat-1 burial site 57](#_Toc197475462)

[3.3.2.1.5. Vas’kovo-4 site 61](#_Toc197475463)

[3.3.2.1.6. Archaeological site complex at the Itkul’ Lake 62](#_Toc197475464)

[3.3.2.1.7. Ust’-Isha site 68](#_Toc197475465)

[3.3.2.1.8. Firsovo-14 burial site 73](#_Toc197475466)

[3.3.2.1.9. Lebedi-2 site 74](#_Toc197475467)

[3.3.2.1.10. Solontsy-5 site 76](#_Toc197475468)

[3.3.2.1.11. Tuzovskiye Bugry-1 Late Neolithic-Eneolithic site 79](#_Toc197475469)

[3.3.2.2. Upper Ob Eneolithic Sites (Russia_UpperObKiprino_Eneolithic) 85](#_Toc197475470)

[3.3.2.2.1. Razdum’ye-1 hillfort 85](#_Toc197475471)

[3.3.2.2.2. Ordynzkoye-1 burial site 86](#_Toc197475472)

[3.3.3. Middle Irtysh and Baraba forest-steppe 86](#_Toc197475473)

[3.3.3.1. Middle Irtysh Neolithic culture (Russia_MiddleIrtyshNeolithic_LN) 86](#_Toc197475474)

[3.3.3.1.1. Omsk Neolithic occupation 86](#_Toc197475475)

[3.3.3.1.2. Korchugan-1 site 87](#_Toc197475476)

[3.3.3.1.3. Protoka site 90](#_Toc197475477)

[3.3.3.2. Eneolithic sites with Comb-Pit Ware (Russia_CombPitWare_Eneolithic) 91](#_Toc197475478)

[3.3.3.2.1. Eneolithic phase of the Borovyanka-17 site 91](#_Toc197475479)

[3.3.3.2.2. Eneolithic phase of the Okunevo site complex 97](#_Toc197475480)

[3.3.3.2.3. Ostrov-2 site 109](#_Toc197475481)

[3.3.3.2.4. Chernoozerye-1 burial site 109](#_Toc197475482)

[3.4. Urals 109](#_Toc197475483)

[3.4.1. Trans-Urals (Tobol River Basin in Western Siberia) 109](#_Toc197475484)

[3.4.1.1. Trans-Ural Late Neolithic-Eneolithic (Russia_CombPitWare_Eneolithic) 110](#_Toc197475485)

[3.4.1.1.1. Sosnovy Ostrov occupation site 110](#_Toc197475486)

[3.4.1.1.2. Boborykino-2 occupation site 111](#_Toc197475487)

[3.4.1.2. Trans-Ural Eneolithic (Russia_CombPitWare_Eneolithic) 112](#_Toc197475488)

[3.4.1.2.1. Gladunino-3 occupation site 112](#_Toc197475489)

[3.4.2. Ural Mountains and Cis-Urals 113](#_Toc197475490)

[3.4.2.1. Urals Early Eneolithic Burials (Russia_UralsEneolithic) 113](#_Toc197475491)

[3.4.2.1.1. Kulmetovskiy-Grot cave (Kulmetovskiy grotto) 115](#_Toc197475492)

[3.4.2.1.2. Kamen’-Dozhdevoy cave 115](#_Toc197475493)

[3.5. Temperate zone of Eastern Europe 117](#_Toc197475494)

[3.5.1. Lower Kama and Middle Volga Region 117](#_Toc197475495)

[3.5.1.1. Elshanka Early Neolithic culture (Russia_Elshanka_EN) 117](#_Toc197475496)

[3.5.1.1.1. Chekalino-4 occupation site 118](#_Toc197475497)

[3.5.1.1.2. Labazy burial mounds 119](#_Toc197475498)

[3.5.1.2. Khvalynsk Eneolithic culture (Russia_Khvalynsk_Eneolithic) 120](#_Toc197475499)

[3.5.1.2.1. Lebyazhinka-5 occupation site 120](#_Toc197475500)

[3.5.1.3. Eneolithic at the Kama Estuary (Russia_KamaEstuary_Eneolithic) 122](#_Toc197475501)

[3.5.1.3.1. Murzikha-2 burial site 123](#_Toc197475502)

[3.5.1.4. Volga Late Eneolithic (Russia_LateVolga_Eneolithic) 126](#_Toc197475503)

[3.5.1.4.1. Maksimovka-1 burial ground 126](#_Toc197475504)

[3.5.2. Volga-Oka Region 128](#_Toc197475505)

[3.5.2.1. Lyalovo Pit-Comb Ware Late Neolithic culture (Russia_UpperVolga_Lyalovo_LN) 128](#_Toc197475506)

[3.5.2.1.1. Lyalovo phase of the Saktysh archaeological site complex 129](#_Toc197475507)

[3.5.2.2. Volosovo Eneolithic culture (Russia_Volosovo_Eneolithic) 135](#_Toc197475508)

[3.5.2.2.1. Volosovo phase of the Saktysh archaeological site complex 136](#_Toc197475509)

[3.5.2.2.2. Imerka-8 occupation site 144](#_Toc197475510)

[3.6. Seima-Turbino-period individuals 144](#_Toc197475511)

[3.6.1. Overview of the Seima-Turbino phenomenon 144](#_Toc197475512)

[3.6.1.1. Seima-Turbino necropolises (burial sites) 145](#_Toc197475513)

[3.6.1.1.1. Rostovka burial site 145](#_Toc197475514)

[3.6.1.1.2. Satyga-16 burial ground 157](#_Toc197475515)

[3.6.2. Peripheral Area of the Seima-Turbino Phenomenon 160](#_Toc197475516)

[3.6.2.1.1. Chernoozerye-1 burial site 160](#_Toc197475517)

[3.6.2.1.2. Tatarka Hill site 161](#_Toc197475518)

[3.6.2.1.1. The Anzhevsky Complex (Nefteprovod-1 & Nefteprovod-2 sites, and Tatarka Hill) 163](#_Toc197475519)

[3.7. Cisbaikal_LNBA-rich outliers from the Krasnoyarsk region 165](#_Toc197475520)

[3.8. References (for Supplementary Information section 2) 167](#_Toc197475521)

[3.8.1. Image sources 176](#_Toc197475522)

[4. Population Genetic Analyses | PCA 177](#_Toc197475523)

[5. Population Genetic Analyses | ADMIXTURE 179](#_Toc197475524)

[6. Population Genetic Analyses | Grouping of individuals via f4-statistics 180](#_Toc197475525)

[6.1. Ungrouped pre-Holocene individuals 180](#_Toc197475526)

[6.2. Grouping individuals from the Transbaikal region and Eastern Siberia 181](#_Toc197475527)

[6.3. Grouping individuals from the Cisbaikal region and Central Siberia 184](#_Toc197475528)

[7. Population Genetic Analyses | qpAdm analysis methodology and robustness 187](#_Toc197475529)

[8. Population Genetic Analyses | qpAdm analyses of East Siberian transect 190](#_Toc197475530)

[8.1. Analysis of Siberian transect 190](#_Toc197475531)

[8.1.1. Modeling approach and parameters 190](#_Toc197475532)

[8.1.2. Naïve models 194](#_Toc197475533)

[8.1.2.1. Tranche 1: Khaiyrgas_16.7kya (Naïve model) 194](#_Toc197475534)

[8.1.2.2. Tranche 2: Ust_Kyakhta_14kya (Naïve model) 194](#_Toc197475535)

[8.1.2.3. Tranche 3: KhatyrstyrCave_M_10.2kya; Kolyma_M_10.1kya (Naïve model) 195](#_Toc197475536)

[8.1.2.4. Tranche 4: Dzhilinda1_M_N_8.4kya; Transbaikal_EMN_9-8kya; Altai_N_9kya (Naïve model) 198](#_Toc197475537)

[8.1.2.5. Tranche_5: Altai_N_7.5-6kya; Transbaikal_N_EMN_7.5-6kya; China_AmurRiver_N; Cisbaikal_EN; Mongolia_N_North; Syalakh-Belkachi (Naïve model) 200](#_Toc197475538)

[8.1.2.6. Tranche_6: Yakutia_LNBA; Cisbaikal_LNBA (Naïve model) 205](#_Toc197475539)

[8.1.3. Models with Cisbaikal_LNBA in Tranche 3 206](#_Toc197475540)

[8.1.3.1. Finding a Tranche for Cisbaikal_LNBA 206](#_Toc197475541)

[8.1.3.2. Tranche 3: KhatyrstyrCave_M_10.2kya; Kolyma_M_10.1kya (Models with Cisbaikal_LNBA in Tranche 3) 208](#_Toc197475542)

[8.1.3.3. Tranche 4: Dzhilinda1_M_N_8.4kya; Altai_N_9kya; Transbaikal_EMN_9-8kya (Models with Cisbaikal_LNBA in Tranche 3) 209](#_Toc197475543)

[8.1.3.4. Tranche 5: Altai_N_7.5-6kya; Transbaikal_EMN_7.5-6kya; China_AmurRiver_N; Cisbaikal_EN; Mongolia_N_North; Syalakh-Belkachi (Models with Cisbaikal_LNBA in Tranche 3) 214](#_Toc197475544)

[8.1.3.5. Tranche 6: Yakutia_LNBA (Models with Cisbaikal_LNBA in Tranche 3) 218](#_Toc197475545)

[8.1.4. Summary of Population Relationships in Siberia 218](#_Toc197475546)

[8.1.4.1. Genetic peculiarities of Cisbaikal_LNBA 218](#_Toc197475547)

[8.1.4.2. Khaiyrgas_16.7kya is a near-unadmixed representative of an “Ancient Paleosiberian” (APS) lineage that retains broad affinities to Native Americans 219](#_Toc197475548)

[8.1.4.3. Ancient Paleosiberian (APS) ancestry persists in Ust_Kyakhta_14kya and Kolyma_M_10.1kya, but with East Asian admixture and possible Native American backflow 220](#_Toc197475549)

[8.1.4.1. Ancient Paleosiberian (APS) ancestry persisted into late Holocene populations of Siberia and the Americas along two routes 221](#_Toc197475550)

[8.1.4.2. East Asian ancestry admixed into Siberian populations during the Holocene along two routes 224](#_Toc197475551)

[9. Population Genetic Analyses | qpAdm analyses of populations on either side of the Bering Straits 227](#_Toc197475552)

[9.1. Connection between the Syalakh-Belkachi population and the Saqqaq Paleo-Eskimo 228](#_Toc197475553)

[9.2. qpAdm models indicate that Ancient Paleosiberian ancestry persisted on both sides of the Bering Strait, especially through Saqqaq.SG-related populations 230](#_Toc197475554)

[9.3. qpAdm models provide evidence for a distinct stream of APS ancestry among Athabaskans 233](#_Toc197475555)

[9.4. Additional genetic analyses provide evidence for a distinct stream of APS ancestry among Athabaskans 236](#_Toc197475556)

[10. Population Genetic Analyses | qpAdm analyses of the NEAHG cline 241](#_Toc197475557)

[10.1. Simple qpAdm models demonstrate that NEAHG populations can be modeled as a cline of admixed populations grading between East Asian acestry, ANE, EHG, and WHG 241](#_Toc197475558)

[11. Population Genetic Analyses suggest a connection between Cisbaikal_LNBA and populations connected to Yeniseian speakers 243](#_Toc197475559)

[11.1. F4-statistics demonstrate that Cisbaikal_LNBA is ancestry plays an important role among Yeniseian, South Siberian Turkic, and Samoyedic populations 244](#_Toc197475560)

[11.2. qpAdm demonstrate that Cisbaikal_LNBA is ancestry plays an important role among Yeniseian, South Siberian Turkic, and Samoyedic populations 244](#_Toc197475561)

[11.3. The conclusion that a close connection exists between Cisbaikal_LNBA and Yeniseian, South Siberian Turkic, and Samoyedic populations is robust to the effects of allelic bias 246](#_Toc197475562)

[11.4. Y-chromosomal connections between ancient Cisbaikal_LNBA individuals and other populations 247](#_Toc197475563)

[11.5. Two Cisbaikal_LNBA-rich outliers indicate that Cisbaikal_LNBA ancestry expands into the Krasnoyarsk region in the Bronze Age 247](#_Toc197475564)

[12. Linguistic note on Yeniseian languages 250](#_Toc197475565)

[13. Population Genetic Analyses suggest a connection between Yakutia_LNBA and Uralic-speaking populations 251](#_Toc197475566)

[13.1. F4-statistics demonstrate that Yakutia_LNBA is especially related to Uralic-speaking populations 251](#_Toc197475567)

[13.2. qpAdm demonstrates that Uralic-speaking populations uniquely among Inner Eurasian populations draw their East Asian ancestry almost exclusively from Yakutia_LNBA 253](#_Toc197475568)

[13.3. The conclusion that a close connection exists between Yakutia_LNBA and Uralic populations is robust to the effects of allelic bias 256](#_Toc197475569)

[13.4. Y-chromosomal connections between ancient Yakutia_LNBA individuals, Tatarka Hill individuals, and other populations 258](#_Toc197475570)

[13.5. Modeling the initial dispersal of Yakutia_LNBA ancestry to the Urals 258](#_Toc197475571)

[14. Linguistic note on Uralic languages 260](#_Toc197475572)

[15. Population Genetic Analyses | qpAdm analyses of Seima-Turbino-period individuals 260](#_Toc197475573)

[16. References 269](#_Toc197475574)

# Geophysical context

## Geographic terms

The study area covers a vast territory of Northern Eurasia from the Far East to the Baltics, encompassing a diverse range of orographic conditions, climate, and resources. The area can be broadly divided into several sub-areas: Northeastern, Central, Western, and Southern Siberia, and the East European Plain (Figure 1).


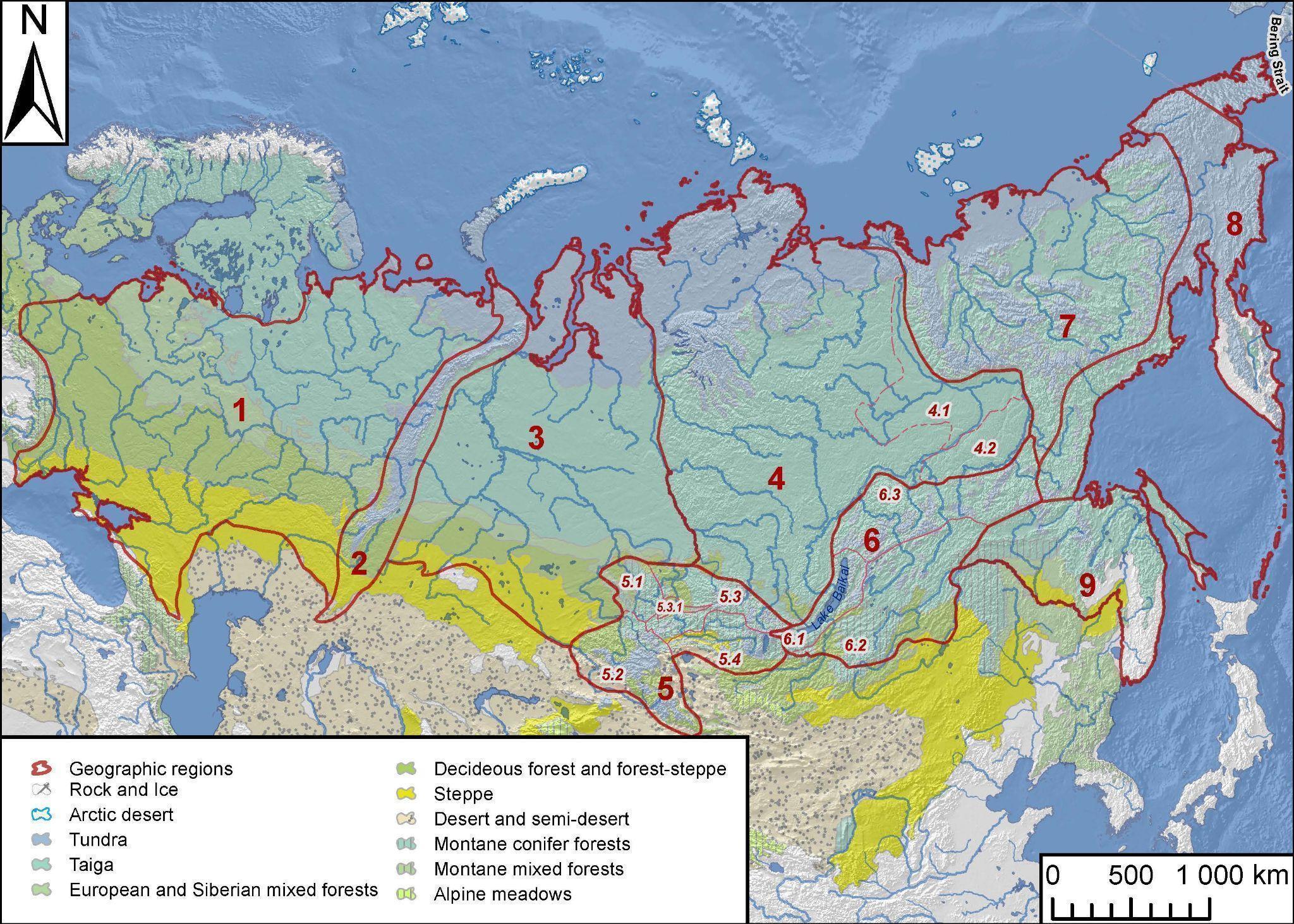


**Figure S1.** Geographic regions of Northern Eurasia. 1 - East European Plain, 2 - Urals, 3 - West Siberia, 4 - Central Siberia (4.1 - Central Yakutia, 4.2 - Aldan), 5 - Altai-Sayan (5.1 - Kuznetsk-Salair, 5.2 - Altai, 5.3 - Sayan with 5.3.1 - Minusinsk Basin, 5.4 - Tyva), 6 - Baikal (6.1 - Baikal, 6.2 - Transbaikalia, 6.3 - Baikal-Stanovoy), 7 - North-East Siberia, 8 - North Pacific, 9 - Amur-Sakhalin (8 and 9 taken together are called Russian Far East) (boundaries of regions and provinces based on Map of the physical-geographical zoning of the USSR 1983, Gvozdevskiy, N. A., & Mikhailov, N. I. (1978); biogeographic regions Olson et al. 2001, generalized).

**Table S1 | Most common geographic terms in main text**

| **Term** | **Regions (numbered in map)** |
| --- | --- |
| Forest belt | Regions colored as “Taiga” and adjoining areas colored as “European and Siberian mixed forests” in Figure S1 |
| Forest-steppe belt | Regions colored as “Decideous forest and forest-steppe” in Figure S1 |
| Northeast Siberia | Region numbered 7 in Figure S1 |
| Central Siberia | Region numbered 4 in Figure S1 |
| Altai-Sayan | Region numbered 5 in Figure S1 |
| Western Siberia | Region numbered 3 in Figure S1. Contains the Ob and Irtysh Basins (Figure S2). |
| Cis-baikal | Southernmost part of Central Siberia (region 4), just to the West of Lake Baikal, in Figure 1, including the headwaters of the Angara and Lena as they leave Lake Baikal (Figure S3). |
| Trans-baikal | Region numbered 6.2, including the region just below the number 6 in Figure S1 |
| Yakutia | Regions numbered 4.1 and 4.2, eastern part of region numbered 4 more generally, plus region numbered 7, in Figure S1 |

**Northeastern Siberia** (Figure 1, see 7) encompasses the eastern regions of Siberia up to the Lena River, as well as the river basins that flow into the Laptev Sea, East Siberian Sea, and Okhotsk Sea. The area is characterized by a complex relief, mainly composed of ridges and plateaus, which descend like an amphitheater to the north, giving way to the Yana-Indigirka and Kolyma lowlands. The climate changes from arctic to subarctic, always continental, with very cold winters and cool summers. This region is one of the "poles of cold" of the Northern Hemisphere, with winter temperatures around -50°C (the official minimum in Oymyakon is -67.8°C). Permafrost is widespread everywhere across the region. The arctic and shrubby tundra in the north of Northeastern Siberia is replaced by taiga in the south, dominated by larch forests that can tolerate the harsh conditions of the area. In the southern part of Northeastern Siberia, the larch taiga is replaced by mixed forests of larch and other coniferous trees such as spruce and pine.

**Central Siberia** (also called Middle Siberia, Figure 1, see 4), is located in the center of North Asia between the major Siberian rivers Lena and Yenisei. The Central Siberian Plateau is the main relief structure of the area, which is bordered in the south by the Eastern Sayan Mountains and the Baikal ranges. The area includes also the North Siberian Lowland and Taimyr (with the Byrranga Mountains) in the north. **Yakutia** (Figure 1, see 4.1) as a vast territory within Central Siberia is often considered within the administrative framework, but in our study it is basically understood as a flat area in the middle and lower reaches of the Lena River, the lower reaches of the Aldan River and the Vilyui Basin. One of the region's features is the Siberian Traps - a huge lava field that erupted millions of years ago, cut through by narrow canyon-like river valleys (the Putorana Plateau). The climate is sharply continental, with a large gradient of winter and summer temperatures, and frosty and dry winters, especially severe in the center of the area in Yakutia; permafrost is widespread almost everywhere. Central Siberia is isolated from the inflow of air masses from the Pacific and Atlantic Oceans, so precipitation comes only from the northwest and its amount is reduced in the easterly direction. The Taimyr Peninsula and the North Siberian Lowland are occupied by arctic tundra and shrubby tundra. Most of the region is dominated by taiga, mainly larch forests, with an admixture of pine and dark coniferous. The most southern part of Central Siberia and the upper reaches of the Angara, flowing from Baikal, is usually called Cis-Baikalia, is occupied by southern taiga landscapes with humid forests of Siberian cedar and spruce.

**Western Siberia** (Figure 1, see 3) is located in the western part of North Asia and covers a vast territory, from the Ural Mountains to the Yenisei River basin. The region is characterized by a slightly undulating plain (the West Siberian Plain), interspersed with low hills and ridges. In this flat landscape, only the Siberian Uvaly band is most noticeable - these are low moraine hills formed after one of the Quaternary glaciations and stretching in a sub-latitudinal direction throughout Western Siberia. The climate of Western Siberia is sharply continental, with large seasonal and diurnal temperature fluctuations. Winters are long, severe, and dry, with temperatures frequently dropping below -30°C. Summers are relatively short, warm, and humid. The region receives low annual precipitation, mainly during the summer months, but it does not have time to evaporate or enter the rivers. Therefore, this region contains the largest wetland areas in the world. Western Siberia is home to a diverse range of natural zones, from tundra and forest-tundra in the north to steppe in the south. The vegetation of Western Siberia is dominated by taiga forests, primarily consisting of coniferous trees such as spruce, fir, pine, and larch. The forested area in the center of the West Siberian Plain is interspersed with huge bogs and other wetlands. In the south, the forest gives way to the forest-steppe zone, characterized by a mixture of birch-pine forest and meadows. The steppe region of Western Siberia is primarily comprised of grasslands, which gradually become drier to the south.

Western, Central, and Northeastern Siberia are bordered to the south by **Southern Siberia Mountains** rugged terrain, which comprises two big regions **Altai-Sayan** (Figure 1, see 5) and **Baikal** (Figure 1, see 6) with several specific provinces features. Altai-Sayan region includes Kuznetsk-Salair mountain-hollow area (5.1); the Altai ridges (5.2); the Western and Eastern Sayan ridges (5.3) with the Minusinsk Basin (5.3.1.); and arid Tyva basin surrounded by ridges (5.4). Some mountain ranges, such as the Mongolian Altai and the Eastern Sayan, extend further to the south and are gradually replaced by the Mongolian Plateau, which descends to the plains to the east. In a broad sense big Baikal region, directly includes the Baikal province (6.1) with mountain ranges and basins directly bordering Lake Baikal from the northwest and southeast; Trans-Baikalia (6.2), which comprises the territory east of Lake Baikal and includes the Vitim Plateau, the mountain ranges in the Selenga River basin, and further southeast the Yablonevy, Daursky, Chersky, and Nerchinsky ranges; and Baikal-Stanovoy also called North Trans-Baikalia (6.3) further extends on the area of Stanovoy ranges and Aldan highlands. In the south, Trans-Baikalia passes into the Mongolian steppes, and in the north-east Baikal-Stanovoy borders the Amur region and the Far East. The mountains of Southern Siberia serve as a natural barrier between the westerlies zone (North Eurasia) and the monsoon zone (South and Southeast Asia). The climate in Southern Siberia Mountains is continental and can be quite harsh, but it is also diverse depending on the relief. Due to the great distance from the ocean, winds from the Atlantic arrive having already lost a significant amount of moisture. Nevertheless, the western and northern slopes of the mountains in Southern Siberia receive a significant amount of precipitation, which is why large Siberian rivers such as the Ob, Yenisei, and Amur originate in this region. The slopes of the mountains are covered with dense dark coniferous and deciduous-coniferous forests, which provide a variety of habitats. In contrast, the southern ranges and intermountain basins receive less moisture and are occupied by dry steppes and even semi-deserts. Additionally, the intermountain basins (like Minusinsk Basin) experience specific winter temperature regimes, as cold air flows down the slopes of the mountains and stagnates there, resulting in very severe frosts and calm weather. Long, frosty winters contribute to the formation of permafrost in this region. Summer in Southern Siberia is short, but still relatively warm.

The **Ural Mountains** (Figure 1, see 2) are a natural boundary between Europe and Asia, separating the Siberian territories from the **East European Plain** (Figure 1, see 1). The extensive East European (Russian) Plain is a vast lowland covering a significant part of Eastern Europe. It extends from north to south from the Barents and White Seas to the Black and Caspian Seas, and from the coast of the Baltic Sea to the Ural Mountains. In the northwest, it is bounded by the Scandinavian mountains, in the southwest by the Sudeten Mountains, in the southeast by the Caucasus, and in the west, the Vistula River serves as a conventional boundary of the plain. Its relief is characterized by a gently rolling terrain with a slight slope towards the north and northwest. The plain has a flat or slightly convex surface, with occasional low hills and ridges. The East European Plain is cut by a network of rivers, including the Volga, the Dnieper, and the Don, which form extensive river valleys and lowlands. The relief of the East European Plain is largely a result of glacial and alluvial processes that took place during the Pleistocene. The plain is divided into several sub-regions with unique topography. The northern part of the plain is dominated by vast wetlands and swamps. The central part is characterized by rolling hills and valleys, while the southern part is a vast flat plain with occasional low ridges. The vegetation zones of the East European Plain are expressed especially distinctly and sub-latitudinally located, and they vary from north to south, reflecting changes in temperature, precipitation, and soil. The northern part of the plain is dominated by tundra and taiga, while the central part is covered by cool mixed and then deciduous forests. The southern part of the plain lies in the forest-steppe zone, where grassy steppe gives way to forests of birch, oak, and pine. The forest-steppe zone is particularly diverse, with rich flora and fauna. The steppe belt is characterized by low landscape, interrupted only by occasional hills and river valleys. In the extreme south, in the Caspian lowland, the vegetation has a semi-desert appearance.


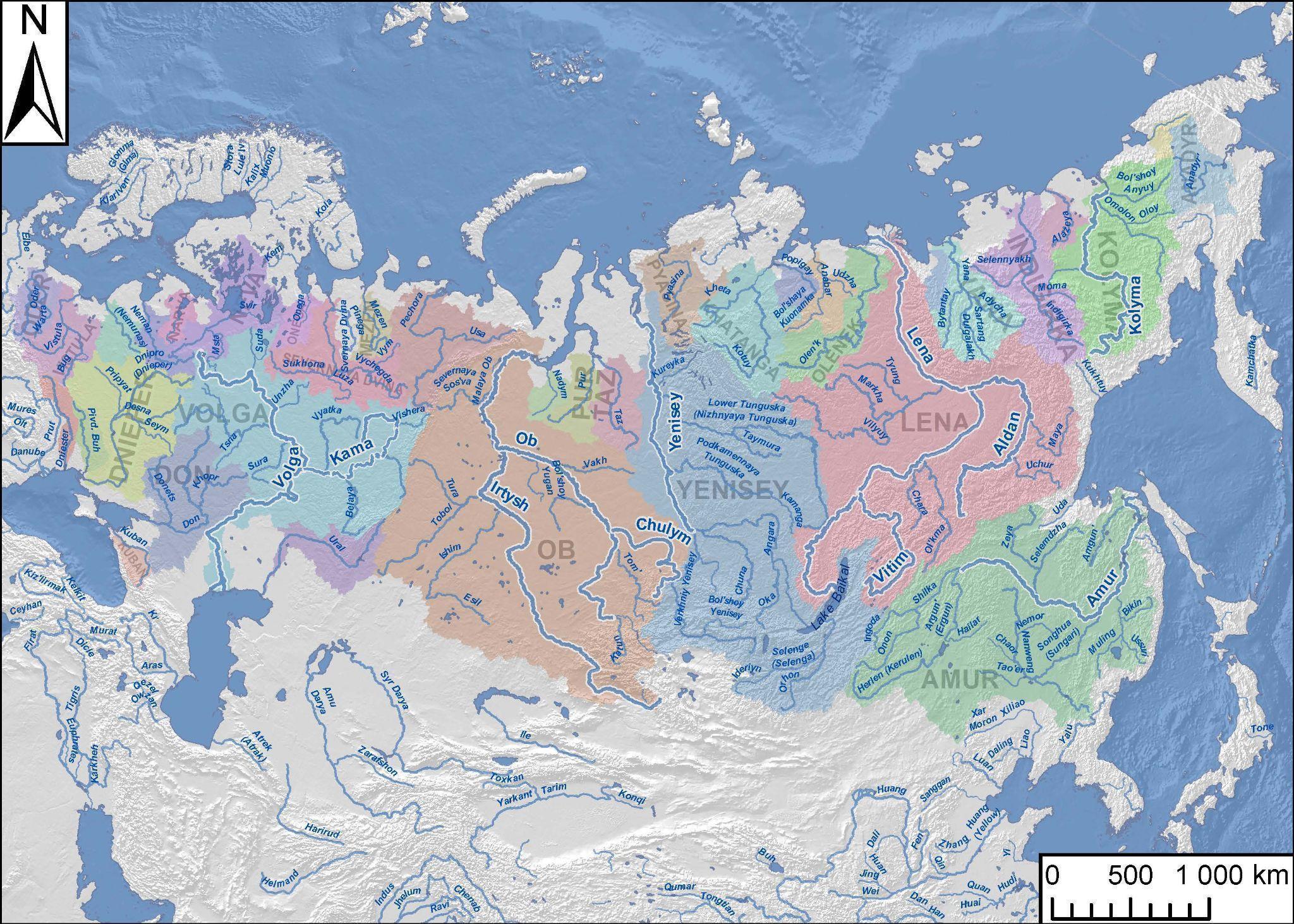


**Figure S2**. Rivers and river basins of Northern Eurasia. The rivers discussed in the article are highlighted.

Main climatic and biogeographic zones of vast territories of Eastern Europe and Siberia are:

**The tundra zone** covers the northernmost part of Eastern Europe and Siberia, stretching from the Arctic Circle to the northernmost coastlines. Tundra is a distinctive habitat characterized by its frigid climate, low biodiversity, short growing season, and poor soil drainage. Permafrost is everywhere except the extreme west of the zone. Many small thermokarst lakes and vast wetlands are impassable in summer. The tundra biome experiences extremely low temperatures and strong winds, creating harsh conditions that limit growth of most plants and animals. The climate is too harsh for trees to grow, and thus tundra biome is characterized by low-growing plants such as mosses, lichens, and dwarf shrubs that are well adapted to the nutrient-poor soils. Insects, rodents, and migratory birds are common in the tundra during the short summer months when there is a burst of vegetation growth, but many animals migrate to warmer regions during the long winter season.

**The boreal zone** covers the northern part of Eastern Europe and Siberia, stretching approximately from the Arctic Circle to the southern boundary of the taiga. The climate is harsh and cold, with long, severe winters and short, cool summers. The boreal zone is characterized by its extremely cold temperatures, with record lows reaching -54°C in some areas of northeastern Russia. The main factor in extremely cold winters in the boreal zone is the Siberian High - a large high-pressure system that forms over Siberia during the winter months, typically from October to April. The Siberian High has a significant effect on the climate, as it brings cold, dry air from the Arctic region and spreads it across much of Siberia and East Asia, and even often determines the weather in Eastern Europe. In the summer months, the Siberian High weakens and moves northward, allowing warmer, moister air to move into the region. Precipitation along the boreal zone is regulated by westerlies and therefore decreases from west to east. The dominant biome in this zone is the taiga, cold coniferous or cool mixed forests. The soil under forests is often acidic, soddy-podzolic, or podzolic. The taiga is dominated by closed, dense forests in the warmer southern regions of the biome. In the colder, northern regions, where the short growing season and low temperatures limit species diversity and tree density, sparse northern taiga forests predominate. Undergrowth is generally absent or weakly developed due to the low levels of light that penetrate the forest canopy. The ground is often covered with a monotony of grass-shrub layers and green mosses. Shrub species such as juniper, honeysuckle, and black currant, as well as dwarf shrubs like blueberries and lingonberries, and herbs such as oxalis and wintergreen, are not abundant.

In the north of Europe, spruce forests are prevalent in the taiga, and towards the south, the forest becomes mixed with deciduous species. In the taiga of the Urals and Western Siberia, light coniferous forests of Scots pine with an admixture of spruce, fir, and Siberian pine dominate. Central and Northeastern Siberia are home to a sparse larch taiga with an undergrowth of elfin Siberian pine and Daurian rhododendron.

**The temperate zone** covers the central part of Eastern Europe and south Urals. Further to the east this zone wedges out; it does not exist in Siberia and reappears only in the southern part of the Far East (in the Amur and Primorye regions). The temperate zone of Eastern Europe is characterized by mild to cool summers and cold winters, with a relatively narrow range of temperatures throughout the year. This region is influenced by both Atlantic and continental air masses, leading to variable weather patterns. Precipitation is moderate and distributed evenly throughout the year, with higher amounts in the western regions. The temperate zone in the Far East has a temperate monsoon climate, with very humid summers. The dominant biome in this zone is the temperate deciduous forest and cool mixed forest, consisting mainly of deciduous broadleaf trees such as oak and maple, as well as mixed forests of conifers and broadleaves. The deciduous forests of Eastern Europe are known for their high biodiversity. The vegetation cover is typically characterized by a multi-layered structure, with an upper layer of tall trees, a middle layer of shrubs, and an understory of herbaceous plants. The forest floor is usually covered with a thick layer of leaf litter and humus, which helps to retain moisture and nutrients. In some areas, the vegetation may also include patches of meadows or wetlands. The soils are typically nutrient-rich and have a high capacity for retaining water, which contributes to the growth of diverse plant communities. The dominant soil types in these forests are generally well-drained and moderately to highly fertile, such as luvisols and cambisols.

To the south, as the climate becomes more continental, forest zones gradually turn into **forest-steppes**. Here, areas of deciduous forests in Eastern Europe or aspen-birch forests in Western Siberia alternate with rich forb-grass meadows on chernozems. The forest-steppe zone has a favorable climate with long, cold winters, but comparatively long and warm summers and a good balance between precipitation and evaporation. Forest-steppe is an important and unique ecosystem, providing various niches and habitats for both humans and wildlife alike.

**The steppe zone** covers the southern part of Eastern Europe and Siberia, characterized by a continental climate with hot summers and cold winters. The steppe belt in the East European Plain stretches from the Carpathians to the southern Urals and the Caspian lowland and further east through Kazakhstan to the Altai mountains, and the fragmented steppes areas continues in the mountain basins of Southern Siberia. Its southern boundary is marked by the foothills of the Caucasus Mountains, while in the north it gradually gives way to the forest-steppe zone. The climate in this zone is continental with hot and dry summers and cold winters. The natural vegetation is dominated by grasses and herbs adapted to arid conditions, with a few sparse shrubs and trees found in river valleys and sheltered areas. Dry grass steppes (feather grass, fescue, couch grass) predominate, and in more humid areas - forb-cereal steppes. As a result of the decay of the rich grass cover in the steppes, chestnut and the most fertile chernozem soils were formed. In the past, the steppe belt was an important area for nomadic pastoralism, and it remains a region important for farming today. The climate along the steppe zone of Eurasia changes from west to east as the region transitions from the Atlantic influence to a more continental regime and falls under the influence of the Siberian High. In the western part of the steppe zone, the climate is characterized by mild, humid winters and warm summers, with a more even distribution of precipitation throughout the year. Moving eastward, the climate becomes increasingly continental, with colder winters and hotter summers, and a more pronounced seasonal distribution of precipitation, with more rainfall in the summer months.

## Climate change during the Late Pleistocene and Early Holocene

There is relatively poor information about how the climate and vegetation of the vast region of Siberia have changed over time, and thus the dynamics of paleoecological changes and their chronology can only be given in broad strokes.

### Late Pleistocene Northern Asia

Based on data compiled from North Eurasia, Tarasov et al. (2000) concluded that the distribution of biomes during the Late Glacial Maximum (18 thousand calibrated years before present, abbreviated as ka BP) differed significantly from that of modern biomes. The taiga belt was greatly reduced and fragmented, and cool mixed and temperate deciduous forests were absent in most areas where they now exist. The tundra belt expanded, and the steppe was the dominant vegetation type across northern Eurasia south of approximately 57°N, directly adjacent to the tundra to the north.

During the Late Pleistocene (21-14ka BP), steppe was widespread in central Yakutia and northeastern Siberia (Cao et al., 2019), reflecting the expansion of tundra-steppe as indicated by the abundance of forb species revealed by ancient sediment DNA between 46 and 12.5 ka on the Taymyr Peninsula (Jørgensen et al., 2012). Relatively closed landscapes with mixed coniferous and deciduous broad-leaved forests were present only in southeastern Siberia, northeastern China, and the Baikal region (Cao et al., 2019). The melting of permafrost caused elevated lake levels, which have been widely documented in Mongolia between 17 and 10 ka BP. Sandy dune formation occurred in western Mongolia during this time, along with development of steppe vegetation. Similar processes likely occurred in the Steppe Altai and south of Western Siberia (Rudaya et al., 2020).

### Early Holocene Northern Asia

At the last deglaciation and the beginning of the Holocene, the tundra-steppe was replaced by light taiga in southern Siberia and by tundra in northern Siberia. In addition to the general increase in temperatures at the onset of the Holocene, the summer monsoon-associated maximum of precipitation around 10.8–10.5 ka BP was pronounced in Southern Siberia, northward and eastern of the Steppe Altai, and north-west China (Rudaya et al., 2020). A change in primary vegetation in Northeastern China occurred in the early Holocene (11.5 and 10.5 ka), caused by rapid increase in abundance of temperate deciduous trees, which may reflect the warmer climate and enhanced summer monsoon (Hong et al., 2009; Liu et al., 2014).

Although the extent of non-tree vegetation decreased in Siberia during the early Holocene warming (12–7 ka BP), the boreal vegetation did not show a corresponding increasing trend. The extensive and deep permafrost in northern Asia provoked a lag in vegetation response to climate change of several millennia. During the early Holocene, only sparse larch forests could survive on these shallow active-layer permafrost regions. It was not until 8 ka BP that evergreen conifer species, dominated by pine and spruce, grew in abundance, which is consistent with the increase in permafrost-free conditions (Li et al., 2022). During the early Holocene (12-8 ka BP), a sharp increase in Northern Hemisphere temperature caused permafrost to thaw rapidly, resulting in the formation of thermokarst lakes transforming landscapes in the vast expanses of Siberia. In the south of Western Siberia, a sharp reorganization of landscape appearance occurred about 11.2-10.1 ka BP when the proportion of birch forests gradually increased although steppes still prevailed in the landscape. Pine forests began to play an important role in vegetation at about 9.6 ka cal BP, apparently spreading from the Urals refugium (Ryabogina et. al., 2020).

In contrast to Central and Northeastern Siberia, the western transfer of heat and precipitation from the Atlantic began to have a more significant impact on the climate of the East European Plain from the very beginning of the Holocene. The shift from tundra and steppe vegetation to a more forested landscape is manifested during the early Holocene as a rapid increase in the abundance of trees and shrubs, including birch, pine, and oak. From 11 to 9 ka BP taiga in Europe retreated northwards; this is reflected in the appearance of deciduous taxa at higher elevations and latitudes (Binney 2017).

The global climatic event at 8.2 ka BP is relatively poorly documented in Siberia due to the rather low temporal resolution of study sites, so its impact on habitat conditions in all regions of Siberia is difficult to trace.

### Middle Holocene Northern Asia

By the middle of the Holocene, an atmospheric circulation similar to the modern one had been established. The long-term general warming (Atlantic optimum) in the middle of the Holocene led to intensification of westerlies' impact on most of Europe and Siberia, against a backdrop of weakening of the Siberian High. The arrangement of biome borders across North Eurasia closely resembled the modern pattern by 8 ka BP (Binney et al., 2017). By this time, the mosaic landscapes of the early Holocene were finally structured, and a clear boundary emerged between the steppe and forest belts of western and central Asia.

During the Holocene optimum (7.5-4 ka BP), the southern boundary of surficial permafrost receded northward to near the Arctic Circle. After a rapid thawing period, there was a relatively slow and decreasing rate of thaw in the mid-late Holocene (7-2 ka BP) (Li et al., 2022). The presence of boreal evergreen conifers in the area that is now dominated by larch forests indicates that mid-Holocene winters were warmer than present in central and southern Yakutia (Tarasov et al., 1998). Around 6 ka BP, the northern forest limit in Siberia had extended poleward by several hundred kilometers north of the modern forest limit, specifically in the Yamal and Taymyr peninsulas. The mid-Holocene warming was most pronounced in Eastern Europe, where tundra was probably absent. Taiga and cool conifer forests occurred further north than the modern range of European Russia, while cold mixed forests were present in the extreme northwest of the region (northern Karelia and Kola). However, taiga had reached its current range in the continental interior (Central and Southern Siberia and northern Mongolia) (Tarasov et al., 1998).

In the southern regions, humidification in the Mid Holocene was not elevated everywhere. Winter precipitation in Central Asia remained relatively low until approximately 8 ka BP before increasing at an almost linear rate to the present level (Liu et al., 2014). Optimal conditions for vegetation and plant diversity in the Steppe Altai and Southern Siberia were recorded between 7.5–7 ka BP and ended 3.6–2.7 ka BP. A warm and arid interval was also recorded in the pollen record of the Baraba forest-steppe (Western Siberia) 7–5 ka BP. The maximum extent of mixed coniferous-deciduous forest in the south and southwest of Southern Siberia has been recorded between 7.2 and 2.7 ka BP (Rudaya et al., 2020).

In the Western Siberia forest-steppe, the driest phase of the Holocene was recorded between 7.1 and 5.5 ka BP. It was associated with an expansion of pine forests and adverse conditions for leaved trees but without signs of an increase in steppe plots in landscapes (Ryabogina et. al., 2020).

In the Southern Ural, a climatic optimum as a warm but also rather humid time was recorded at 7.4–6.3 ka BP. Detailed reconstructions of vegetation and climate dynamics in the forest-steppe interface zone of the Eastern European Plain demonstrated trajectories to temperatures warmer than the present level (by 1–1.4 C) at 7–4.8 ka BP. However, the forest-steppe boundary remained near its present position during this period because annual precipitation was similar to the current values, about 500 mm per year (Novenko et al., 2016). On the other hand, there is evidence of drier conditions that resulted in the formation of mid-Holocene buried humus soils in the forest belt (Aleksandrovskiy and Chichagova 1998). Paleohydrological studies in the central part of European Russia also showed that runoff in the Don and Dnieper basins was 40% less than modern values (Sidorchuk et al. 2012).

As insolation decreased and the impact of westerlies weakened, various regions experienced a shift towards cooler climates, dating back to about 5 ka BP. However, moisture dynamics during this period have been less clear (Wanner et al., 2008).

The treeline in Northeastern and Central Siberia retreated to the south between 5 and 3 ka cal yr BP (Binney 2017), while the cooling after 4.3 ka BP stimulated the freezing of wet mires in the middle taiga zone and formation of palsa bogs in the northern taiga and forest-tundra in Siberia. Against this background, the role of the light larch taiga increased, while the conditions for the dark coniferous taiga worsened.

After boreal woodland expansion the onset of the Middle Holocene in northern Mongolia, at the end of the middle Holocene (4.5 ka BP), there was a decrease in precipitation and changes in vegetation towards the steppe. Humidity increased again only during the late Holocene in concert with dropping temperatures (Hurka et al., 2019).

The Steppe biome in Southern Siberia and Steppe Altai also expanded as aridification began around 4.8 ka BP. Despite the gradual decrease of precipitation and the intensification of aridity, the interval of 4.5–2.5 ka BP cannot be described as xerothermal (Rudaya et al., 2020).

Climate deterioration in the south of the Ural Mountains started earlier, around 6.3 ka BP. But in the Western Siberia forest-steppe, a reduction in arid conditions started only from 5.5 to 4.9 ka BP, likely due to decreased evaporability from cooling, but there is little evidence of a significant increase in precipitation. A gradual rise in forest-steppe lake levels started only around 4.9 thousand years ago, likely due to increasing rainfall, and the proportion of birch in the forests increased while the proportion of pine decreased (Ryabogina et. al., 2019; 2020). The ratio of pine to birch in Siberian forest-steppe typically reflects changes in humidity levels, with an increase in the proportion of birch forests during more humid periods with elevated water tables, and a displacement of birch by pine during dry periods. Humid conditions were not constant, as there was also an impulse towards dryness at about 3.5–3.3 ka BP (Ryabogina et. al., 2019; 2020).

The expansion of pine in the steppe belt of Western Siberia and Kazakhstan occurred from the southern Ural and West Siberia to northern and central Kazakhstan after 5.5 ka BP. The vegetation cover approached its present-day appearance as pine spread throughout the steppe zone. During a short aridization period ca. 3.5 ka BP, forest-steppe and northern steppe biomes moved beyond their present-day borders to the north. Later, the late Holocene was characterized by the dominance of steppe with birch and pine forests along rivers (Hurka et al., 2019).

In the Eastern European Plane, cooling with increasing precipitation from 4.8–2.5 ka BP were partially responsible for changes in vegetation within the forest-steppe boundary. Woodland cover expanded, reaching 40% of the area at about 3.7 ka BP years ago. The proportion of broadleaf trees increased, spruce gradually penetrated the forests, and alder became abundant. A widespread shift of forest into forest-steppe and steppe areas has been traced by several pieces of evidence (Novenko et al., 2016). Climate reconstructions show that the subsequent phase (3.7-1.7 ka BP years ago) was marked by climate cooling and an increase in precipitation, consistent with other evidence for climate cooling in Northern and Central Europe around this time (Wanner et al., 2008).

## References (for Supplementary Information section 1)

Li, W., Tian, F., Rudaya, N., Herzschuh, U., and Cao, X. (2022). Pollen-Based Holocene Thawing-History of Permafrost in Northern Asia and Its Potential Impacts on Climate Change. Frontiers in Ecology and Evolution, 10. <https://doi.org/10.3389/fevo.2022.894471>

Tarasov, P. E., Volkova, V. S., Webb, T., Guiot, J., Andreev, A. A., Bezusko, L. G., Bezusko, T. V., Bykova, G. V., Dorofeyuk, N. I., Kvavadze, E. V., Osipova, I. M., Panova, N. K., and Sevastyanov, D. V. (2000). Last glacial maximum biomes reconstructed from pollen and plant macrofossil data from northern Eurasia. Journal of Biogeography, 27(3), 609-620. <https://doi.org/10.1046/j.1365-2699.2000.00429.x>

Tarasov, P. E., Webb, T. III, Andreev, A. A., Berezina, N. A., Bezusko, L. G., Blyakharchuk, T. A., Bolikhovskaya, N. S., Cheddadi, R., Chernavskaya, M. M., Chernova, G. M., Dorofeyuk, N. I., Dirksen, V. G., Elina, G. A., Filimonova, L. V., Glebov, F. Z., Guiot, J., Gunova, V. S., Harrison, S. P., Jolly, D., . . . Zernitskaya, V. P. (1998). Present-day and mid-Holocene biomes reconstructed from pollen and plant macrofossil data from the former Soviet Union and Mongolia. Journal of Biogeography, 25(6), 1029-1053. <https://doi.org/10.1046/j.1365-2699.1998.00236.x>

Rudaya, N ., Krivonogov, S., Słowiński, M., Cao, X., and Zhilich, S. (2020). Postglacial history of the Steppe Altai: Climate, fire and plant diversity. Quaternary Science Reviews, 249, 106616. <https://doi.org/10.1016/j.quascirev.2020.106616>

Cao, X., Tian, F., Li, F., Gaillard, M.-J., Rudaya, N., Xu, Q., and Herzschuh, U. (2019) Pollen-based quantitative land-cover reconstruction for northern Asia covering the last 40 ka cal BP, Clim. Past, 15, 1503–1536, <https://doi.org/10.5194/cp-15-1503-2019>

Jørgensen, T., Haile, J., Möller, P., Andreev, A., Boessenkool, S., Rasmussen, M., Kienast, F., Coissac, E., Taberlet, P., Brochmann, C., Bigelow, N.H., Andersen, K., Orlando, L., Gilbert, M. T. P., and Willerslev, E.: A comparative study of ancient sedimentary DNA, pollen and macrofossils from permafrost sediments of northern Siberia reveals long-term vegetational stability, Mol. Ecol., 21, 1989–2003, 2012.

Hong, B., Liu, C., Lin, Q., Yasuyuki, S., Leng, X., Wang, Y., Zhu, Y., and Hong, Y.: Temperature evolution from the δ18O record of Hani peat, Northeast China, in the last 14000 years, Sci. China Ser. D, 52, 952–964, 2009.

Liu, Z., Wen, X., Brady, E. C., Otto-Bliesner, B., Yu, G., Lu, H., Cheng, H., Wang, Y., Zheng, W., Ding, Y., Edwards, R. L., Cheng, J., Liu, W., and Yang, H.: Chinese cave records and the East Asia Summer Monsoon, Quaternary Sci. Rev., 83, 115–128, 2014.

Ryabogina N.E., Afonin A.S. and Ivanov S.N. Late Glacial and Holocene in the south of Western Siberia: geochemical indices and pollen data in Kyrtyma Lake sediments. IOP Conf. Ser.: Earth Environ. Sci. 438 012023. <https://doi.org/10.1088/1755-1315/438/1/012023>

Binney, H., Edwards, M., Macias-Fauria, M., Lozhkin, A., Anderson, P., Kaplan, J. O., Andreev, A., Bezrukova, E., Blyakharchuk, T., Jankovska, V., Khazina, I., Krivonogov, S., Kremenetski, K., Nield, J., Novenko, E., Ryabogina, N., Solovieva, N., Willis, K., and Zernitskaya, V. (2017). Vegetation of Eurasia from the last glacial maximum to present: Key biogeographic patterns. Quaternary Science Reviews, 157, 80-97. <https://doi.org/10.1016/j.quascirev.2016.11.022>

Liu Z. Y., Wen X. Y., Brady E. C. et al. Chinese cave records and the east Asia summer monsoon. Quaternary Science Reviews. 2014. Vol. 83. Р. 115–128.

Novenko, E.Y., Tsyganov, A.N., Rudenko, O.V. et al. Mid- and late-Holocene vegetation history, climate and human impact in the forest-steppe ecotone of European Russia: new data and a regional synthesis. Biodivers Conserv 25, 2453–2472 (2016). <https://doi.org/10.1007/s10531-016-1051-8>

Aleksandrovskiy AL, Chichagova OA (1998) Radiocarbon age of Holocene paleosols of the East European forest–steppe zone. Catena 34:197–207

Sidorchuk A, Panin A, Borisova O (2012) River runoff decrease in North-Eurasian plains during the Holocene optimum. Water Resour 39:69–81

Wanner, H., Beer, J., Bütikofer, J., Crowley, T. J., Cubasch, U., Flückiger, J., Goosse, H., Grosjean, M., Joos, F., Kaplan, J. O., Küttel, M., Müller, S. A., Prentice, I. C., Solomina, O., Stocker, T. F., Tarasov, P., Wagner, M., and Widmann, M. (2008). Mid- to Late Holocene climate change: An overview. Quaternary Science Reviews, 27(19-20), 1791-1828. <https://doi.org/10.1016/j.quascirev.2008.06.013>

Hurka H., Friesen N., Bernhardt K.-G., Neuffer B., Smirnov S., Shmakov A., Blattner F. The Eurasian steppe belt: Status quo, origin and evolutionary history. Turczaninowia, 2019. Т. 22, № 3. С. 5-71 <https://doi.org/10.14258/turczaninowia.22.3.1>

Ryabogina, N. E., Afonin, A. S., Ivanov, S. N., Li, H., Kalinin, P. A., Udaltsov, S. N., and Nikolaenko, S. A. (2019). Holocene paleoenvironmental changes reflected in peat and lake sediment records of Western Siberia: Geochemical and plant macrofossil proxies. Quaternary International, 528, 73-87. <https://doi.org/10.1016/j.quaint.2019.04.006>

Olson, D. M., Dinerstein, E., Wikramanayake, E. D., Burgess, N. D., Powell, G. V. N., Underwood, E. C., D'Amico, J. A., Itoua, I., Strand, H. E., Morrison, J. C., Loucks, C. J., Allnutt, T. F., Ricketts, T. H., Kura, Y., Lamoreux, J. F., Wettengel, W. W., Hedao, P., Kassem, K. R. 2001. Terrestrial ecoregions of the world: a new map of life on Earth. Bioscience 51(11):933-938.

Map of the physical-geographical zoning of the USSR. Scale: 1:24000000. 1983. Atlas of the USSR. Maps of Nature. Physical-geographical zoning / Main Directorate of Geodesy and Cartography under the Council of Ministers of the USSR. Moscow. 1983.

Gvozdevskiy, N. A., & Mikhailov, N. I. (1978). Physical Geography of the USSR. Asian Part. 3rd ed., revised and enlarged. Textbook for students of geographical faculties of universities. Moscow: "Mysl".

# Radiocarbon Dating and Stable Isotope Analysis

In total, we publish 82 new radiocarbon dates. At least some of these dates do not coincide with the generally accepted chronology of the archaeological context of the dated individuals. Such disparity of radiocarbon age and archaeologically-based chronology has been repeatedly reported for prehistoric populations in Northern Eurasia and are attributed to the freshwater reservoir effect (FRE). FRE is manifested in human bone samples when the population practices a fish-based diet and consumes aquatic resources enriched with non-atmospheric (i.e., aquatic) carbon (Culleton, 2006).

The assessment of the likelihood of age discrepancies in the acquired radiocarbon dates, therefore, requires a reconstruction of dietary and determination of a possible freshwater reservoir offset (FRO).

***Dietary analysis via Stable Isotopes***

Carbon (δ13C) and nitrogen (δ15N) stable isotope ratios are extensively employed for reconstructions of diet of past human populations. The most impressive were obtained in distinguishing marine and terrestrial diets, but lower δ13C with elevated δ15N values in human collagen from continental areas are also supposed to reflect the consumption of freshwater fish, including among pastoral and nomadic communities (Marchenko et. al., 2021). Although we do have not enough comparative faunal isotopic data to perform full-scale dietary research, we can elaborate on some assumptions regarding the role of freshwater resources consumption in populations studied, based on published data on δ13C and δ13N isotopes in river fish and prehistoric humans from various parts of Siberia (Marchenko etc. al., 2021; Kuzmin et. al., 2020).

The human samples in our dataset show a range from -24,5 to -16,9‰ in δ13C and from 11,6 to 16,4‰ in δ15N.

The observed range of δ15N characterizes the studied populations as non-agricultural consumers of terrestrial and aquatic fauna with a high trophic position. Privat et al. (2005) consider δ15N above 13,5 ‰ as a marker of freshwater fish consumption for the Bronze Age population of the Baraba forest steppe.

The fish samples from the rivers of Northern Eurasia show decreased values of δ13C varying mostly from -30,6‰ to -22,1‰ (Marcheno et. al., 2021; Kuzmin et. al., 2020; Святко, 2016). In lake fish, the δ13C values vary in a greater range and are higher on average than in river fish, from -29,6‰ to -8,3‰, but the analyzed excavated fish samples demonstrate values vary from -29,6 to -20,2‰, which match with river fish better (ibid.). For Altai and Minusinsk herbivores, δ13C varies from -18 to -22 ‰ (Svyatko, 2017).

Based on the observed values, we assume the individuals from our dataset who demonstrate δ13N values greater than 13,5‰ as having had a significant (or even prevailing) proportion of fish in their diet. Respectively, the fish diet of the individuals with less than -22‰ δ13C was oriented mostly on river fish consumption while the others with higher δ13C values might include more recourses from lakes and other inland waterbodies.

***Freshwater reservoir offset (FRO) estimation***

Recent comprehensive observations of the freshwater reservoir effect recorded in human bones from Eurasian riparian populations (Святко, 2016) demonstrate high inequality of biases derived from the FRO. The 14C dating of paired terrestrial and aquatic animals from archaeological records revealed the FRO measured up to ~1000 radiocarbon years in the fish from the Ob and the Irtysh as well as from several smaller rivers and lakes of the Kazakh Uplands (Svyatko, 2017).

Special observation on paired human-fauna 14C dating of the Baikal region revealed a positive correlation between δ13N and expected FRO (Schulting et. al., 2014). Another study in the Upper Lena region revealed a moderate but significant negative correlation between human δ13C values and human-animal offsets in 14C years and no relationship between the 14C offset and δ15N (Schulting et. al., 2015). Marchenko et al. (2021) noticed greater values of FRO in samples from the big rivers, the Ob, and the Irtysh, relative to small tributaries and lakes.

All observations demonstrate the regional character of the recorded relations between carbon and nitrogen isotopic values and the FRO. We see no possibility at the moment to elaborate on any general regression equation for the trans-regional estimation of the expected FRO. Also, the observed values of carbon and nitrogen isotopes in our dataset do not exclude the possibility of the FRO for any individual. However, we can assess the relative probability of FRE-driven aging of the 14C dates of studied individuals based on the observed δ13N-δ13C ratio.

We expect relatively highest FRO in dated individuals with river fish-based diet, from the sites Satyga-16, Neolithic-Early Bronze Age burials at Okunevo, Tuzovskie Bugry-1 (Vasino-5), Sosnovy Island, Firsovo-11, and Chernoozerye-1 in the Ob River basin, and Maksimovka-1, Sakhtysh-2a, and Murzikha-2 in the Volga and Kama river basins. The dates of individuals from these sites require an alternative confirmation, which can be obtained from archaeological context or additional paired human-fauna dating.

The lowest probability of the FRO we expect in the individuals with the lowest values of δ15N and highest values of δ13C. This makes dates from the sites Chuiya, Khatystyr-Cave, and Manzurka-2 in the Lena basin, Boborykino, Gladunino, and Tatarka-Hill in the Ob basin, Kulmetovskiy-Grot, and Sakhtysh-2 in the Volga-Kama basin, Afontova-Gora, Bazaikha, Krasnoyarsk finds, Rasputino, Tolsty-Mys-1, and Ust'-Shamanka-1.


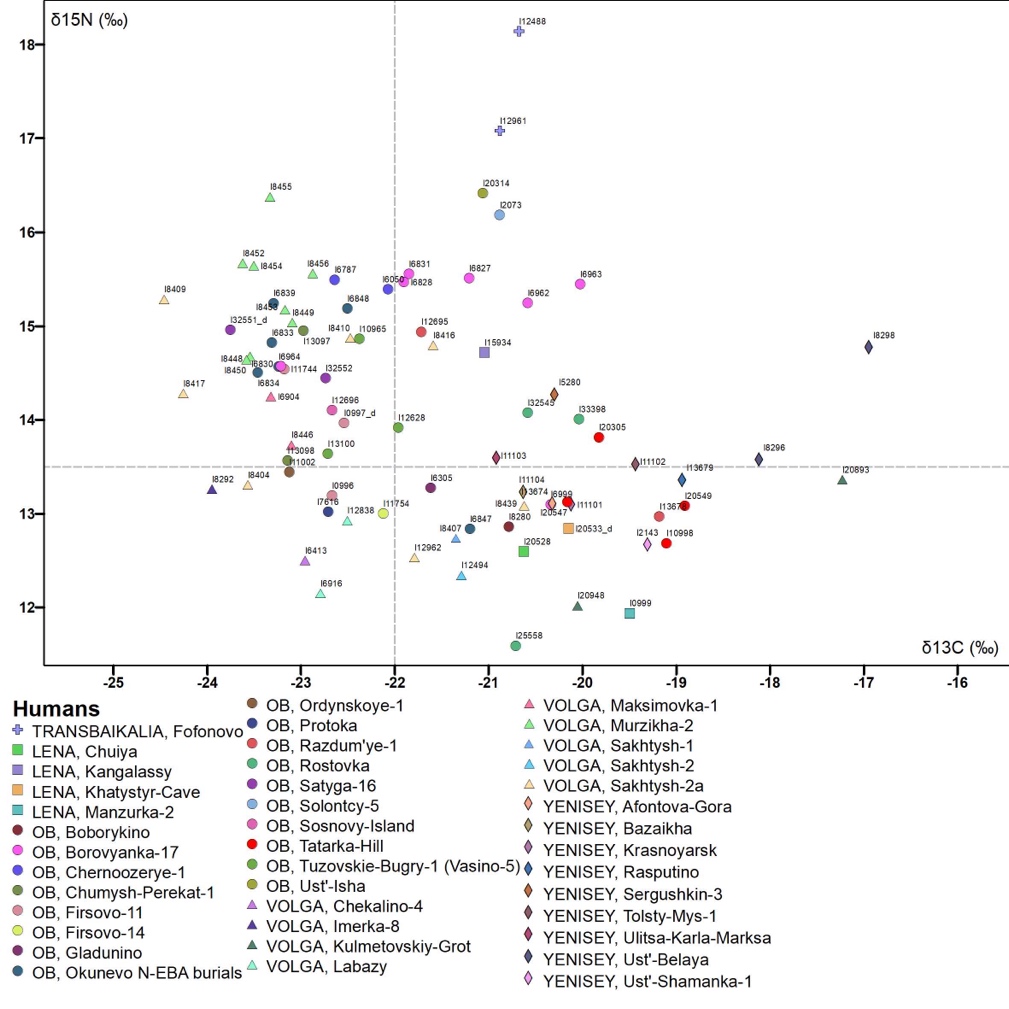


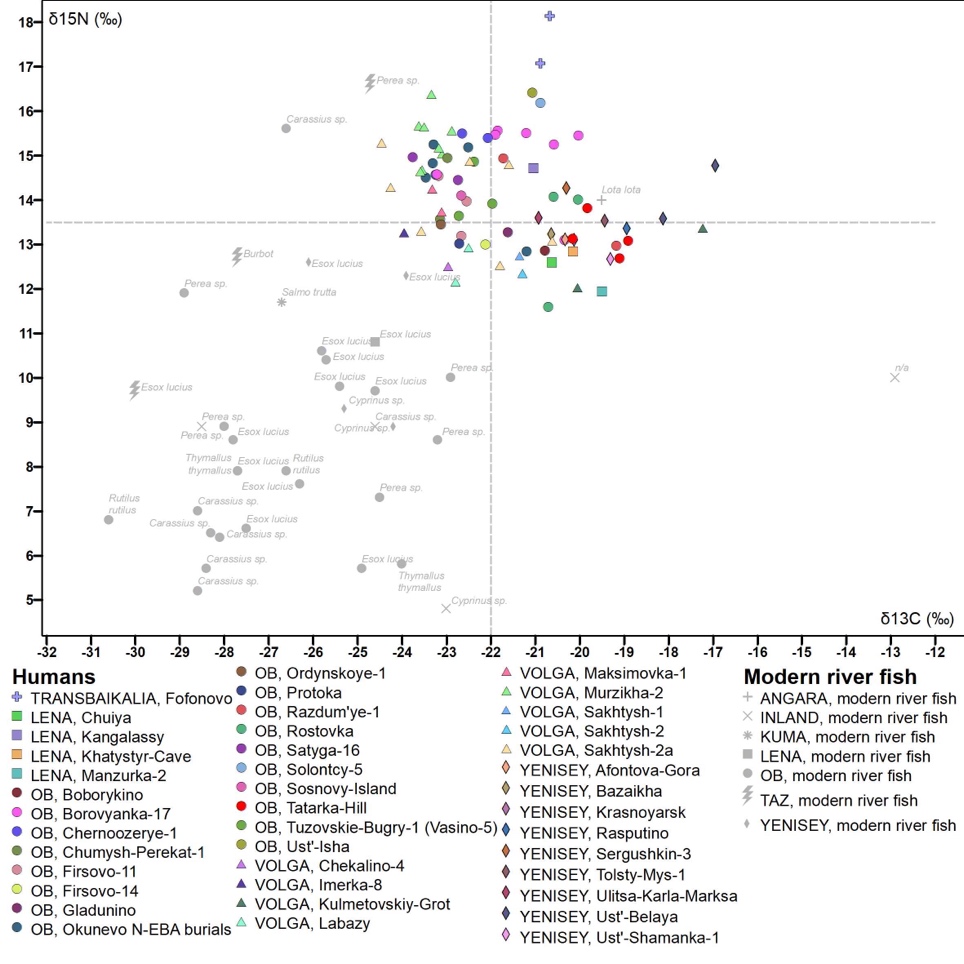


***Figure S3.*** *δ13С and δ15N isotopic collagen values of newly dated human bone samples (top), and against modern fish from Northern Eurasia* *(bottom).*

**References for Supplementary Information section 2**

Culleton, B. J. Implications of a freshwater radiocarbon reservoir correction for the timing of late Holocene settlement of the Elk Hills, Kern County, California. *Journal of Archaeological Science* **33**, 1331–1339 (2006).

Marchenko, Z. V., Svyatko, S. V. & Grishin, A. E. δ13С and δ15N isotope analysis of modern freshwater fish in the south of Western Siberia and its potential for palaeoreconstructions. *Quaternary International* **598**, 97–109 (2021).

Kuzmin, Y. V., Kosintsev, P. A., Boudin, M. & Zazovskaya, E. P. The freshwater reservoir effect in northern West Siberia: 14C and stable isotope data for fish from the late medieval town of Mangazeya. *Quaternary Geochronology* **60**, 101109 (2020).

Privat, K. L. *et al.* Economy & diet at the Late Bronze Age-Iron Age site of Cica: artefactual, archaeozoological & biochemical analyses. *Eurasia Antiqua* **11**, 419–448 (2005).

Святко, С. В. Пресноводные резервуарные эффекты в Евразийской степной зоне и их влияние на радиоуглеродный возраст костных образцов. *Вестник археологии, антропологии и этнографии* 165–173 (2016).

Svyatko, S. V., Reimer, P. J. & Schulting, R. Modern freshwater reservoir offsets in the Eurasian steppe: implications for archaeology. *Radiocarbon* **59**, 1597–1607 (2017).

Schulting, R. J., Ramsey, C. B., Bazaliiskii, V. I., Goriunova, O. I. & Weber, A. Freshwater reservoir offsets investigated through paired human-faunal 14C dating and stable carbon and nitrogen isotope analysis at Lake Baikal, Siberia. *Radiocarbon* **56**, 991–1008 (2014).

Schulting, R. J., Ramsey, C. B., Bazaliiskii, V. I. & Weber, A. Highly variable freshwater reservoir offsets found along the Upper Lena watershed, Cis-Baikal, Southeast Siberia. *Radiocarbon* **57**, 581–593 (2015).

# Archaeological Context

## Eastern Part of Central Siberia

### Middle Lena

#### Sumnagin Mesolithic culture in the Middle Lena Region & the Dyuktai culture

In 1964–1965, Yuri Mochanov discovered the first sites of the Sumnagin culture along the Aldan River, identifying them as a distinct Paleolithic culture of the Late Holocene. Later, he classified this culture as the Latest Paleolithic. The Sumnagin culture's territory spans a vast area in Northeast Asia from Taimyr (Tagenar-6) and the upper reaches of the Vilyui (Ust-Chirkuo) in the west to Chukotka in the east (Panteleikha-3), encompassing the Arctic Ocean coast in the north (Siktyakh), adjacent islands (Zhokhovo), and extending to the basins of the Aldan, Vitim, and Olekma Rivers in the south, possibly even further southward. The Sumnagin assemblage is found in layers at multiphase sites such as Ust-Timpton-1 (horizons 4A to 4G), Belkachi-1 (layers 8 to 20), and Sumnagin-1 (layers 17 to 28). Chronologically, the Sumnagin culture is dated based on numerous radiocarbon dates, generally accepted as ranging from 10/9.5 to 6.5 thousand years ago (Mochanov, Fedoseeva, 1976). Recently updated chronological data suggest the age of the Sumnagin culture to be between 8700 and 4800 calBCE (History of Yakutia, 2020).

The economy of the Sumnagin-attributed population relied primarily on elk and deer hunting. Approximately 90% of flint tools were fashioned from blades extracted from prismatic cores. These blade tools encompassed inserts, retouched knives, scrapers, angles and side burins, borers, among others. More than half of the blade tools were crafted from microliths with a width of less than 0.4 mm. Bone tools were also utilized for inserting flint blades. Among the large tools, diabase axes-head (including those with "ears") and adzes were common (Mochanov, 1969; 1977; Mochanov, Fedoseeva, 1976; 2013). At the Zhokhovo site, evidence of sled dogs was discerned (Pitulko, Kasparov, 2017). However, to date, no burials from the Sumnagin period have been unearthed. Consequently, a Khatystyr man may offer insight into the origins of the Sumnagin culture from the region to the south of Yakutia. Notably, from the northern location of Duvanny Yar in the lower reaches of the Kolyma, an isolated find of a fragment of a female skull and a series of remains at the Zhokhovo site have been documented (Pitulko et al., 2015; Pitulko and Pavlova, 2015; Sikora et al., 2019). The formation of the Sumnagin archaeological assemblage is associated with significant changes in lithic technology and most likely the replacement of earlier population groups by newcomers from southern regions during the transition to the Holocene. Some researchers attribute the rapid spread of typical Sumnagin prismatic cores to the invention of dog-assisted transport (Pitulko 2010).

In contrast to Sumnagin lithic techniques, the Late Pleistocene populations of Central and Northeastern Siberia employed wedge-shaped cores and bifacial tools, commonly described as the “Beringian tradition”. The ***Dyuktai culture*** (18-11 kya) was a part of this tradition. The principal culture-defining stone tools of the Dyuktai people included bifacially worked knives and spearheads, as well as scrapers, wedge-shaped cores, end scrapers, burins, inserts, and chisel-like tools. Blanks for these implements were typically produced from flakes and blades of various shapes and sizes, as well as from flat slabs of siliceous rock. Among the others, Dyuktai artifacts were found in the Khaiyrgas Cave in the Middle Lena region, from which the individual yak029.SG or MiddleLena_Khaiyrgas_16.7kya came (Stepanov et al., 2003). The characteristic wedge-shaped microcores of the Beringian tradition were also found at the Ust-Kyakhta site, from which the individual UKY001 or Ust-Kyakhta_14kya came, which belongs to the Selenga culture (Pavenok et. al. 2019).

The Dyuktai tradition represents the northern variant of a broad technological zone characterized by the production of microblades from wedge-shaped cores. Southern expressions of this lithic technology are known from the Baikal region, as well as from present-day Mongolia and China (Keates et al., 2019).

##### Khatyrstyr Cave site

The Khatystyr Cave is located on the right bank of the Aldan River, approximately 2 km from the village of Khatystyr. It is a limestone cavern distinguished by a shallow entrance in the form of a hole, which formed at the site of a deep fissure.

###### Single burial (individual ID I20533, male[[1]](#footnote-1))

In 1962, human bones were inadvertently discovered by A. Ivanov, a foreman of the Aldan timber industry enterprise, in a cave near the village of Khatystyr in the Aldan region of Yakutia. The skeleton, found lying on its back not far from the entrance and adjacent to the wall, was accompanied by evidence of a fire. Further exploration of the cave revealed a collection of bear, wolf, and fox bones within its depths. Initially, the bones were presumed to be recent, prompting examination by criminal investigators and, at their request, by geologist B.S. Rusanov. Subsequently, archaeologists Yuri Mochanov and Svetlana Fedoseeva also examined the cave and the skeleton. Based on their preliminary findings, the presence of modern objects (such as a metal button or buckle) near the human remains led them to conclude that the bones held no archaeological significance. Consequently, the study of the Khatystyr individual was discontinued, and the skeleton was transferred to the Geological Museum of the Yakut branch of the USSR Academy of Sciences (now the Geological Museum of the Institute of Geology of Diamond and Precious Metals of the Siberian Branch of the Russian Academy of Sciences). Nonetheless, some of the bones were retained by B.S. Rusanov for radiocarbon dating conducted in the 1970s. The results revealed remarkable antiquity of the skeleton, dating back 9800 years and making it, at the time, the oldest discovery not only in Yakutia but also in Eastern Siberia as a whole. Regrettably, these findings were not published in scientific journals and thus failed to attract the attention of researchers.

In 2017, Viktor Dyakonov reevaluated the bone remains, obtaining an AMS date of 9100 BP. Dates were also obtained for the bones of a bear and a dog, which were slightly more recent. The skull from the burial is dated to 8285–8211 calBCE (a combination of two dates from human bones, 8287–8012 calBCE (8985±30 BP, PSUAMS-8447), and 8291–8022 calBCE (9010±30 BP, IAAA-170069), the date of human bones is confirmed by dated dog skull from the same layer in the cave, 8284–7975 calBCE (8980±30 BP, IAAA-183039). The dates correspond with the early period of the Sumnagin Mesolithic culture, dated to 10700–6800 calBP (8700–4800 calBCE) (Moiseyev et al. 2023).

The find of a dog skull in the bottom layer of the Khatystyr Cave (Moiseyev et al. 2023) relates the Khatystyr burial with the Early Neolithic burials with Canidae recorded in the Baikal area. At least three of them are to be listed: the Olkhon Island of the Baikal Lake, a collective burial 26 of the Shamanka-2 site, and a Late Mesolithic burial Lokomotiv-R8 that contained human bones and bones of a wolf, dated to 6374–6031 calBCE (TO-11558) (Bazaliyskiy 2012).

#### Syalakh Neolithic culture (Russia_MiddleLena_SyalakhBelkachi_EN)

As a specific culture of the Early Neolithic, the Syalakh was first identified by Yuri Mochanov in 1964–1966 at the Aldan multiphase sites (Mochanov, 1966; 1969). One of the first-discovered sites attributed to the Syalakh culture, the site Syalakh, which gave its name to the Early Neolithic culture of Yakttia, was discovered as early as in 1943 by Aleksey Okladnikov 90 km south of Zhigansk. The main sites of the Syalakh culture are the multi-layered sites of Sumnagin-1 (layers 11 to 16), Belkachi I (layers 6-7), Ugino-1 (layer 5), Ust-Mil-1 (layer 5) on the Aldan River, Kurung-2 (layer 4), Bolshaya Kyuske (layer 6), Tensik-2 (layer 3) on the Olekma River, Khatyngnakh-2 (layer 4, 5), Suldyukar (layer 3), Talanda-2 (layer 7) on the Vilyui River, Siktyakh-1 (layer 7) on the Lena River. The area of the Syalakh culture covers a vast territory in Northeast Asia from Taimyr to Chukotka. It was formed as a result of the migration of tribes from the Cisbaikal region. Numerous radiocarbon dates obtained date the Syalakh culture in the range from 4900 to 3500 calBCE (Mochanov, Fedoseeva, 1976; 2013; Alekseev, 1987; 1996).

Together with the Syalakh culture, pottery with imprints of braided net, fully polished tools, axes and stepped adzes, and knives, were introduced to Yakutia for the first time. Among other toos, double-sided retouched arrowheads and spears, knives, adzes with core-shaped handles spread. Harpoons stand out from bone tools. Hunting for elk and deer among the Syalakh tribes was supplemented by lake and river fishing.

Numerous pieces of rock art of the Early Neolithic with images of elk and deer were discovered. The dating of these rock arts to the Early Neolithic is confirmed by sacrificial offerings in the form of flint arrowheads, scrapers, and other items that are typologically interpreted as Syalakh (Okladnikov, Zaporozhskaya, 1972; Kochmar, 1994).

Burials in the Syalakh culture were not known until the Matta burial was discovered in 1996 (Pestereva, Stepanov, Dyakonov, 2016; Zubova, Batanina, Panov, Stepanov, Kishkurno, 2017). The last two out of three radiocarbon dates showed age of 5475–5071 calBCE (6328±81 BP, NSKA-1663) and 4442–4257 calBCE (5940±30 BP, Beta-422229) (Kılınç et al., 2021), which generally refers the Matta burial to the turn of the Early Neolithic and Mesolithic in Yakutia.Kangalassy site

The burial was accidentally discovered by workers while digging a utility pit in the center of the village of Kangalassy. Kangalassy is situated at the foot of a hill, directly beneath the high left bank of the Lena River. The area is a small hill near the mouth of the Zolotinka River.

As a distinctive culture of the Early Neolithic, the Syalakh culture was first identified by Yuri Mochanov in 1964–1966 at the Aldan multiphase sites (Mochanov, 1966; 1969). One of the earliest-discovered sites attributed to the Syalakh culture, the Syalakh site, which lent its name to the Early Neolithic culture of Yakutia, was initially found in 1943 by Aleksey Okladnikov, located 90 km south of Zhigansk. The primary sites of the Syalakh culture include the multiphase sites of Sumnagin-1 (layers 11 to 16), Belkachi-1 (layers 6-7), Ugino-1 (layer 5), Ust-Mil-1 (layer 5) on the Aldan River, Kurung-2 (layer 4), Bolshaya Kyuske (layer 6), Tensik-2 (layer 3) on the Olekma River, Khatyngnakh-2 (layer 4, 5), Suldyukar (layer 3), Talanda-2 (layer 7) on the Vilyui River, and Siktyakh-1 (layer 7) on the Lena River. The geographical extent of the Syalakh culture encompasses a vast area in Northeast Asia, stretching from Taimyr to Chukotka. It is believed to emerge as a result of a migration from the Cisbaikal region. Radiocarbon dates obtained place the Syalakh culture in the timeframe of 4900 to 3500 calBCE (Mochanov, Fedoseeva, 1976; 2013; Alekseev, 1987; 1996).

With the Syalakh culture, pottery featuring imprints of braided nets, fully polished tools such as axes, stepped adzes, and knives were introduced to Yakutia for the first time. Additionally, double-sided retouched arrowheads and spears, knives, adzes with core-shaped handles, and bone harpoons became prevalent. Hunting activities among the Syalakh tribes expanded to include lake and river fishing.

Numerous pieces of rock art from the Early Neolithic period depicting elk and deer have been discovered. The dating of these rock arts to the Early Neolithic is supported by sacrificial offerings found alongside them, such as flint arrowheads and scrapers, which are typologically associated with the Syalakh culture (Okladnikov and Zaporozhskaya, 1972; Kochmar, 1994).

Burials attributed to the Syalakh culture were not known until the discovery of the Matta burial in 1996 (Pestereva et al., 2016; Zubova et al., 2017). Radiocarbon dating of the Matta burial indicated an age range of 5475–5071 calBCE (6328±81 BP, NSKA-1663) and 4442–4257 calBCE (5940±30 BP, Beta-422229) (Kılınç et al., 2021), placing it generally at the transition between the Early Neolithic and Mesolithic periods in Yakutia.

##### Kangalassy site

The Kangalassy site, where the burial was accidentally discovered by workers excavating a utility pit in the center of the village of Kangalassy, is situated at the foot of a hill directly beneath the high left bank of the Lena River. This area is characterized by a small hill near the mouth of the Zolotinka River.

###### Single burial (individual ID I15934, male)

During the excavation of a cesspit in 1958, workers unearthed human bones along with a skull, a bone knife with stone blades, four bone arrowheads, a bone awl, and several bone fragments, presumed to be from needles. Archaeologists were summoned to the site, and Ivan Novgorodov conducted excavations of the preserved portion of the burial. The following details were documented:

The burial was interred at a depth of 1.2 meters below the contemporary surface. The bones were encased in an organic layer, possibly composed of birch bark. The orientation of the burial was with the head positioned towards the east. Small beads crafted from mollusk shells, believed to adorn sewn clothing, were found "neatly arranged along the skeleton" on the femur and tibia bones. According to accounts from the workers, similar beads were also discovered on the ribs.

Adjacent to the tibia bones, on their left side, an irregularly shaped circle crafted from thick shell with two holes in the center was unearthed. Additionally, within the same vicinity, three jade tools, ten arrowheads, a small flat stone fragment with a blade on one side (possibly a cutting tool) composed of dark material, a broken whetstone comprising five pieces, two bone awls, and a smaller bone knife were uncovered. These artifacts were subsequently sent to a museum and initially attributed to the Bronze Age. However, following reassessment and radiocarbon dating, researchers reattributed the Kangalassy burial to the Belkachi culture. The most recent radiocarbon dates obtained (4354–4260 calBCE (2468±20 BP) [R_Combine: (5515±25 BP, PSUAMS-9131), (5400±30 BP, IAAA–170064)]) suggest an earlier age and cultural affiliation with the Syalakh culture for the burial. Considering its relatively early chronological attribution, we designate the Kangalassy burial as part of the Syalakh-Belkachi complex.


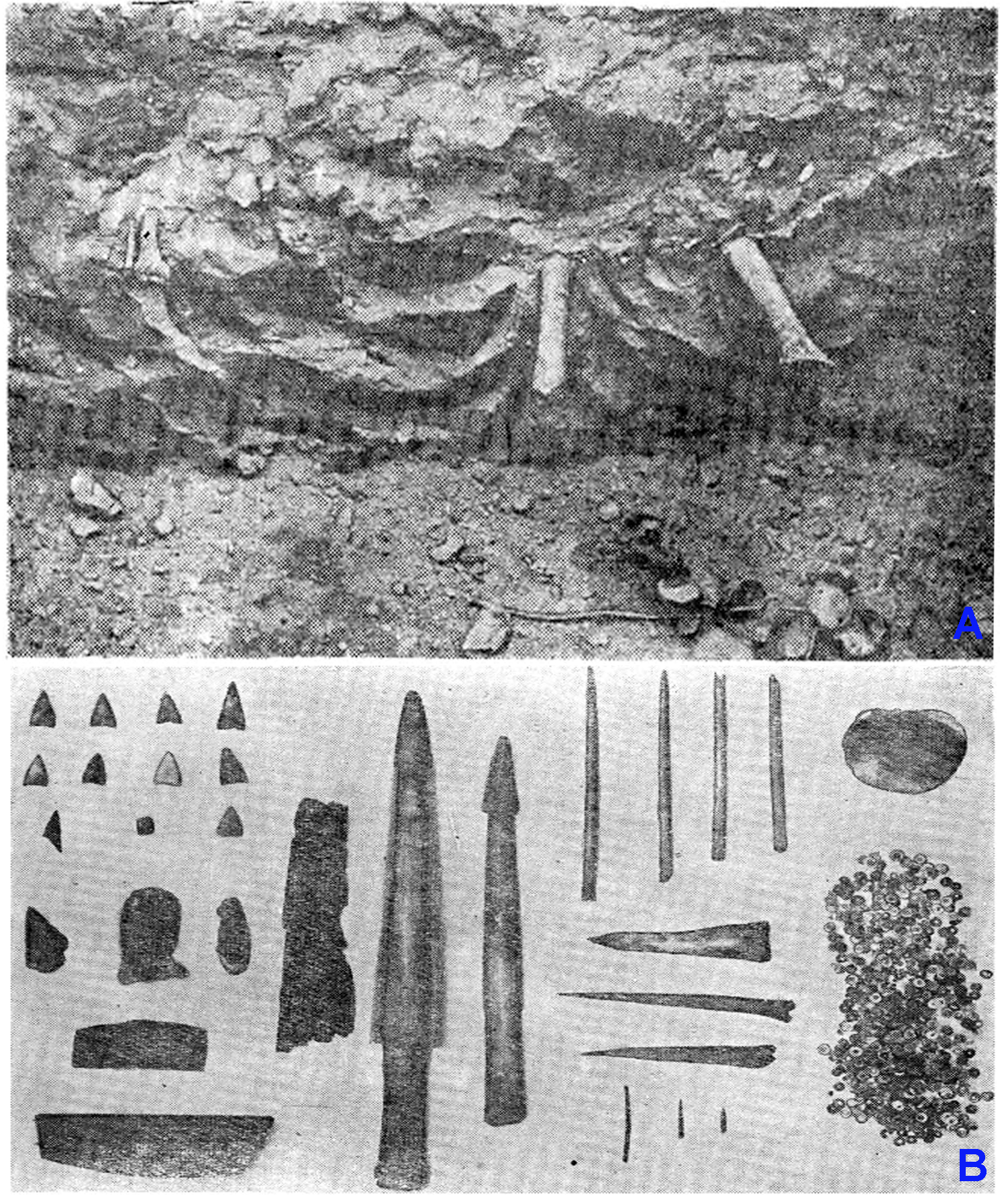


***Figure S4.*** *The Kangalassy burial.* ***A*** *- burial at the moment of discovery;* ***B*** *- grave goods (by Novgorodov, 1960, modified).*

#### Belkachi Neolithic culture (Russia_MiddleLena_Belkachi_N)

The identification of the Belkachi culture became feasible only in 1964–1966 with the discovery of multiphase sites on the Aldan River, notably Belkachi-1 and Sumnagin-1 (layers 9 to 10). At the Belkachi-1 site, the remains of the Belkachi culture were found in layers 4 and 5, sandwiched between the Ymyyakhtakh assemblage (layer 3) above and the Syalakh culture layers (layers 6 to 7) below (Mochanov, 1969; Mochanov and Fedoseeva, 1976; 2013).

Similar stratigraphic configurations were observed at Siktyakh-1, the northernmost multiphase site in Yakutia, located on the Lower Lena River, where Belkachi materials were discovered in the 6th layer, situated between the Syalakh and Ymyyakhtakh assemblages. Additional sites associated with the Belkachi culture include Ust-Timpton-1, Bilir, Ust-Mil-1, and Ugino-1 on the Aldan River, as well as Ust-Chirkuo, Talanda-2, Khatyngnaakh, and Suldyukar on the Vilyui River. Radiocarbon dating places the Belachi culture between 5200±100 to 4100±100 BP (Mochanov and Fedoseeva, 1976; 2013). Recent updates provide a chronological interval of 4200–2100 calBCE (History of Yakutia, 2020).

The Belkachi culture extended across a vast expanse of Northeast Asia, spanning from the Yenisei River tributaries to the outer reaches of Chukotka and as far south as the Amur region. Its assemblage is distinguished by pottery with cord impressions, pointed or rounded bottoms, and rims adorned with smooth or comb-shaped depressed belts. Stone tools feature polished stepped adzes, chipped and partially polished axes, adzes with ledges-ears, various types of arrowheads, multifaceted cutters-drills, beak-shaped combined tools, polished knives, bilaterally retouched spearheads and darts, and bone liner tools.

Rock art, more abundant than in the Early Neolithic, predominantly depicts animals such as elks and deer, albeit with slight variations in detail and style from earlier drawings. Anthropomorphic images are believed to have emerged during this period, often accompanied by sacrificial offerings comprising stone and bone tools characteristic of the Belkachi culture.

Burials associated with the Belkachi culture have been identified at various locations, including Tuoy-Khaya on the Vilyui River, Kangalassy, Uolba, Ogonyok, Khayirgas, and Onnyos on the Lena River, Dzhikimda on the Olyokma River, and Rodinka on the Kolyma River. Notable features of Belkachi burials include the use of ocher, extensive grave goods such as bone and stone artifacts, including flint arrowheads, bone composite tools, polished adzes, daggers, piercings, awls, bone jewelry, shell decorations for clothing, small sculptures, and animal remains.

One of the principal challenges concerning the Belkachi culture is establishing its genetic ancestry in relation to modern populations of northern Eastern Siberia and Alaska. Yuri Mochanov suggested a connection between the Belkachi population and the Na-Dene-speakers, alongside assimilation processes with the Ymyyakhtakh people.

The Belkachi culture, ranging from 4200–2100 calBCE, stands as one of the largest cultural entities in Northeast Asia, with its technocomplex not only prevalent in Yakutia but also extending to other regions such as the Lower Amur, Northern Sakhalin, Taimyr, and Chukotka. Some viewpoints propose that the cultural influences of the Belkachi culture extended to America, potentially contributing to the development of the Arctic small tool tradition in Alaska, Canada, and Greenland. Belkachi burials, characterized by soil grave pits, ocher backfilling, extended supine positioning of the deceased, and abundant accompanying grave goods, have been discovered along rivers including the Lena, Vilyui, Olekma, Amga, and Kolyma (Alekseev et al., 2022).

##### Ongonyok site

The Ogonyok burial, unearthed in October 2016 during the excavation of a utility pit by a power shovel, at a summer cottage in Yakutsk, is located 8.7 km south-southwest of the city center, situated on the southeastern slope of the second floodplain terrace of the Lena River. Adjacent to the burial site is the Late Neolithic site Ogonyok, discovered in 2001 and attributed to the Ymyyakhtakh culture. Positioned in the northern part of the terrace, adjacent to the habitation area, the burial site was originally covered by a pine forest but is now predominantly occupied by suburban structures (Alekseev et al., 2022).

###### Single burial (individual ID I23448, male)

Upon initial examination, it was evident that the burial had suffered severe damage from the power shovel. The skull and artifacts were recovered from the bottom of the pit and the spoil. The skull, in a deteriorated state, retained the parietal and frontal bones, while the facial and occipital parts were missing. The interior surface of the vault exhibited dense red ochre pigmentation. Additionally, a human tibia with traces of ochre and a fragment of a collarbone were found at the pit's bottom.

Excavations focused on the surviving part of the grave, allowing for the tentative conclusion that the grave pit had an oval shape. An ochre layer was identified within the preserved portion of the grave's fill and on its bottom. Based on the impression of the skull on the pit wall, it was deduced that the individual was interred on their back, with the head oriented towards the southeast, facing the nearby oxbow lake upstream of the Lena River. The grave goods discovered in situ included an ochre-dyed bone needle located to the right of the individual's head. Within the fill of the grave pit, two fragments of small animal bones, likely remnants from a funerary feast, were uncovered. Detailed sieving of the spoil from modern construction yielded accompanying materials from the Ogonyok burial. These included a core, three flint knife-like plates, an adze, a fragment of a polishing stone, a fragment of a composite arrowhead with a flint insert, and an anthropomorphic figurine carved from mammoth tusk.

Four radiocarbon dates were obtained from the Ogonyok burial through AMS at the Institute of Accelerator Analysis, Japan. The human skull bone was dated to 4241–4001 calBCE (IAAA-170070), and the tibia showed the date 4050–3967 calBCE (5210±20 BP, IAAA-170063). Additionally, two AMS 14C dates were obtained from animal bones found in the accompanying inventory of the burial. One of them, derived from a lynx canine, yielded a date of 3970–3790 calBCE (5090±30 BP, IAAA-162746). Similarly, the other date obtained from a reindeer bone was 3960–3790 calBCE (5070±30 BP, IAAA-162747). It is worth noting that the dates obtained from the animal bones are essentially identical (Alekseev et al., 2022). As the intervals of dates obtained from human and animal bones do not overlap, we suggest using only the combination of animal-derived dates to prevent possible freshwater reservoir offset of C14 dates, which is often recorded in the Lena region. Thus, the burial is dated to 3957–3799 calBCE (5080±22 BP) [R_Combine: (5090±30 BP, IAAA-162746), (5070±30 BP, IAAA-162747)].


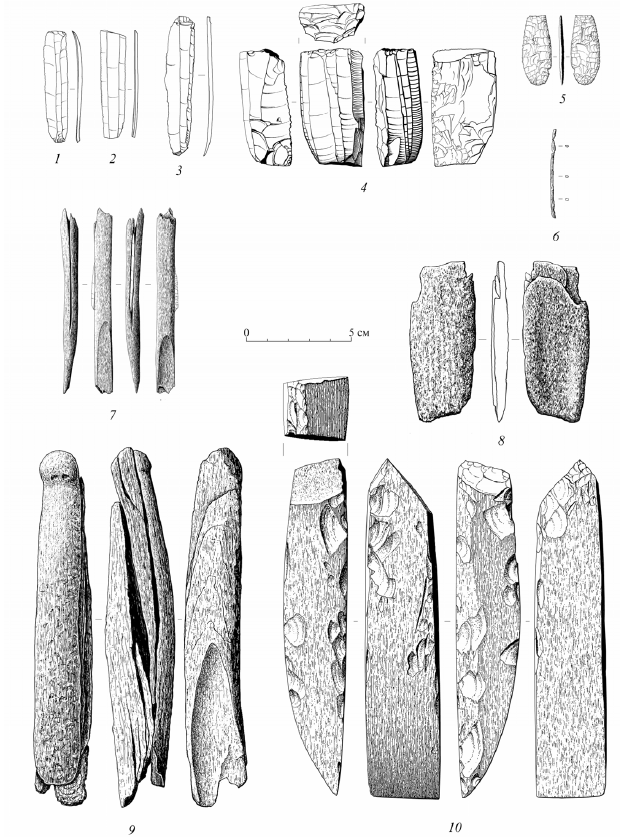


***Figure S5.*** *Grave goods from the Ogonyok burial.* ***1-3*** *- blades;* ***4*** *- prismatic core;* ***5*** *- flint arrowhead;* ***6*** *- bone needle;* ***7*** *- bone composite arrowhead with a blade in the groove;* ***8*** *- bone polisher;* ***9*** *- anthropomorphic figurine made from a mammoth tusk;* ***10*** *- polished chisel (by Alekseev et al., 2022).*

#### Ymyyakhtakh Neolithic culture (Russia_MiddleLena_Ymyyakhtakh_LN) & Siberian Bone Armor

One of the earliest known Late Neolithic sites was discovered by Aleksey Okladnikov in 1942 on the Lena River, at the Ymyyakhtakh site, which later lent its name to a distinct culture. Based on the materials from this site, Aleksey Okladnikov identified a specific stage of the Late Neolithic in the Middle Lena region (Okladnikov, 1950; 1955). An unadmixed assemblage of the Ymyyakhtakh culture was first identified by Yuri Mochanov in 1964–1965 at the multiphase sites of Belkachi-1 and Sumnagin-1 on the Aldan River (Mochanov, 1969). The age of the Ymyyakhtakh culture is determined by radiocarbon dates to be 4100±100 – 3300±100 BP (Mochanov, Fedoseeva, 1976). According to the latest updated data, the age of the Ymyyakhtakh culture is determined as 2950–1150 calBCE (History of Yakutia, 2020).

This culture occupied a vast area. Margarita Kiryak, an investigator of Chukotka, suggests a “global” character of the Ymyyakhtakh people expansion (Kiryak, 1993). In the west, the Ymyyakhtakh culture spread almost to the Yenisei River, where it is recorded in the Krasnoyarsk-Kansk forest-steppe together with ceramics of the Glazkovo tradition (Timoschenko 2013). In the north it occupied Taimyr; in the east it extended to the Northern Okhotsk and Eastern Chukotka regions, and in the south – to the Baikal and Transbaikalia. Characteristic features of the Ymyyakhtakh assemblage include multilayer waffle-style-ornamented, ribbed, and smooth-walled pottery with an admixture of organic matter (animal wool, grass, and needles) found in pottery clay (Mochanov, 1969; Fedoseeva, 1980). Additionally, the clay contains sand, grass, fireclay, small pebbles, and crushed quartzite. A distinctive element of the pottery ornamentation is a belt of through rounded holes, along with incised geometric patterns such as intersecting lines, zigzags, and triangles.

Stone tools of the Ymyyakhtakh people exhibit meticulous craftsmanship and precise forms, crafted from various types of stone including arrowheads, spears, scrapers, cutters, knives, abrasives, axes, and adzes. Ymyyakhtakh people were proficient in various stone processing techniques mastered in previous eras such as retouching, polishing, drilling, sawing, and separation of plates from cores (Fedoseeva, 1980). Bone and horn tools were also common, used for hunting and fishing implements, weapons, protective gear, jewelry, and household items (Fedoseeva, 1980).

In the Late Neolithic rock art, anthropomorphic figures became prominent, while animalistic drawings receded. The drawings, stylized to some extent, were made in red ocher of various shades (Okladnikov and Zaporozhskaya, 1972; Kochmar, 1994).

Two large Ymyyakhtakh cemeteries are known – Chochur-Muran and Deering-Yuryakh on the Middle Lena, along with several single and double burials such as Ichchilyakh on the Lower Lena, Vilyui Shosse in Yakutsk, Pomazkino, Kamenka-2 on the Kolyma River, and Ergetsy on the Vilyui River (Fedoseeva, 1980; Fedoseeva, 1988; 1992; Mochanov and Fedoseeva, 2013; Okladnikov, 1946; Dyakonov et al., 2003; 2012; Kashin and Kalinina, 1997; Kashin, 2013; Kılınç et al., 2021). In Central Yakutia, the Kyordugen burials provided vivid material allowing reconstruction of the social structure and military affairs of the ancient population of Yakutia at the end of the Stone Age (Alekseev et al., 2006; Fyodorova, 2008; Stepanov et al., 2012; Kılınç et al., 2018; 2021).

Warrior burials highlight the significance of warfare and military activity within Ymyyakhtakh society. A number of finds of bone armor plates have been recorded in Yakutia, with the most elaborate example of protective military equipment discovered at the Kyordyughen site, from which individual N4a1.SG in the Yakutia_LNBA cluster was found. Similar armor occasionally appear in the occupational layers of other Ymyyakhtakh sites. The spread of Ymyyakhtakh martial traditions beyond Yakutia is evidenced by the discovery of bone armor plates in Glazkovo-culture burials along the Upper Lena and Yenisei rivers (Alekseyev et al., 2006).

It is believed that the Ymyyakhtakh people played a direct role in the formation of the Yukagir and Nganasan ethnic groups and the Yukagir culture (Kiryak, 1993; Khlobystin, 1998; Everstov, 1999; 2014).

##### Chuiya site

The Chuiya site is located on a cape-like ledge of a 6-meter terrace along the lakeshore, intersected by a modern highway. This cape extends between two expansive basins, with Lake Chuiya occupying the southern basin and the village of Chuyayu situated in the northern basin. About 300 meters west of this cape, the Maya River emerges from Lake Chuiya, flowing westward, bypassing the village of Maya, and eventually merging into the larger Lake Maya. The Maya-Chuyuyinskaya lake system forms part of the Lena-Amga interfluve, characterized by typical landscapes of alas-lakes and erosion-accumulative landforms. In 1992, residents of the village of Chuiya discovered human bones in an erosive thermokarst ravine, formed as a result of road construction in the 1970s (Stepanov et al., 2021).

###### Destroyed burial (individual ID I20528, male)

The burial site was investigated by ethno-archaeologists N.P. Prokopiev and E.K. Zhirkov. Unfortunately, the skull and part of the upper shoulder level were lost, likely due to thermal erosion of the terrain following bulldozing during road construction. Part of the skeleton was exposed on the eroded surface of the ravine slope, and the femur was discovered by local children. Ten meters west of the burial, at a depth of 27 cm, in a small rounded pit, a human mandible was found, accompanied by a needle case characteristic of the Ymyyakhtakh culture, made of thin-walled hollow bone with decorative horizontal carved lines (Prokopiev, 1994).

Initially, in 1992, the investigators assumed that the mandible and the skeleton belonged to the same burial, and the burial was tentatively assigned to the Neolithic period based on the presence of the needle case. However, after excavations conducted in 2010, and considering analogies in the position of the side-oriented skeleton, it was proposed that the burial might be attributed to the Early Iron Age. This hypothesis was subsequently supported by radiocarbon dating, which dated the postcranial skeleton of the buried individual to the Early Iron Age, specifically between 753 and 409 calBCE. The mandible underwent two separate dating processes, both indicating a Neolithic age: 1957–1750 calBCE (3540±30 BP, Beta-432254) (Stepanov et al., 2021) and 1882–1699 calBCE (3475±25 BP, PSUAMS-9018). These two age determinations give in combination the date 1888–1750 calBCE. Therefore, two burials were discovered in the erosional thermokarst ravine: one from the Early Iron Age and the other (the mandible and the needle case) from the Late Neolithic Ymyyakhtakh culture. The second burial underwent archaeogenetic analysis (individual ID I20528).

## South part of Central Siberia and the Baikal Region

### Transbaikalia, Cisbaikalia, and Upper Angara

#### Early Neolithic.(Russia_Fofonovo_Kitoi_EN) and Bronze Age (Russia_Fofonovo_Glazkovo) in the Transbaikalia

##### Fofonovo burial site

The cemetery is situated on the right bank of the Selenga River, approximately 0.5 km upstream from the village of Fofanovo, in the Kabansky district of the Republic of Buryatia, Russia. The burials are found on the southwestern, southeastern, and eastern slopes of Fofanovskaya mountain.

The Fofanovo burial ground is a significant and ancient burial complex in the Baikal region, consisting of three distinct groups of burials from different periods, namely the Early Neolithic and the Bronze Age. The site was initially explored in 1926 by Aleksey Okladnikov, followed by excavations conducted by Mikhail Gerasimov in 1936 and 1959, Aleksey Okladnikov in 1948 and 1950, Vasily Konev from 1987 to 1991 and 1996, and since 2007 by Elena Zhambaltarova.

Mikhail Gerasimov and Evgeny Chernykh, based on the stratigraphy, planigraphy of the burial ground, and the characteristics of the burial rites, identified three distinct groups of burials.

The first group comprises 28 Early Neolithic (Kitoi) burials: 1-7 (1959), 1-17 (1987–1989 and 1996), 1, 3-5 (2007, 2008, 2013), and a ritual object with a bear skull (2008). This group is characterized by the absence of tomb structures, shallow burial depth (ranging from 0.5 to 1.4 m from the contemporary surface), oval and approximately rectangular pit shapes, deceased individuals placed on their backs or sides with bent legs, southeast orientation of the bones, and the presence of ocher in the backfill. Radiocarbon dates based on bone samples from these burials fall within the range of 7036–6073 calBCE (7610±210 BP, GIN-4477) to 5471–5215 calBCE (6350±50 BP, GIN-4128) (Mamonova and Sulerzhitsky, 1989).

The second group of the Fofanovo burial ground consists of 29 Glazkovo burials: burials 8-36 (1959) and 6 (2013). Many of these burials feature stone rubbleworks above the pits. The deceased individuals are positioned on their backs in an extended or bent leg position. The burial pits are typically long (up to 2.8 m) and narrow (0.5-0.8 m) in shape. The grave goods include arrowheads, scrapers, knives, horn and bone artifacts, metal tools, ceramic fragments, and more. Radiocarbon dating of bone samples from this group of burials yielded dates ranging from 2196–1937 calBCE (3670±40 BP, GIN-4473) to 2908–2352 calBCE (4100±100 BP, GIN-4803) (Mamonova and Sulerzhitsky, 1989).

The cultural and chronological attribution of the third group of burials, consisting of burials 37-41 (1959), remains undetermined. These burials lack tomb structures, and the pit contours were not clearly delineated. The skeletons were found at a depth of 0.45-1 m from the modern soil level. The deceased individuals were laid on their backs with bent knees, and the orientation varies from northeast to southeast. Only a needle case and fragments of a bone-based dagger were discovered in the graves of this group. According to Mikhail Gerasimov and Evgeny Chernykh, these burials were made at a later time than the Glazkovo burials (Zhambartalova and Volkov, 2016; Lbova et al., 2008; Gerasimov and Chernykh, 1975).

###### Imprecisely known burials (individual IDs I12488, I8501, I12961, I8535, identified as males)

We conducted sequencing on four individuals originating from the Fofonovo burial site, specifically recorded as coming from **excavation 4, burial 1** (individual ID I12488); **excavation 4, burial 7** (I8501); **excavation 4, burial 10** (I12961); and **excavation 7** (I8535). Regrettably, these burials cannot be precisely matched with existing published sources, precluding a comprehensive assessment of their archaeological context. Two individuals, ID I12961 and ID I8501, underwent radiocarbon dating, yielding an estimated age range of 5883–5720 calBCE (6905±35 BP, PSUAMS-13169) and 5886-5728 calBCE (6925±30 BP, PSUAMS-14539) respectively, which aligns with the established chronological framework of the site  and suggests Early Neolithic (Kitoi) attribution of these burials. For individual ID I12488 rediocardon determination showed age 2288-2058 calBCE (3770±25 BP, PSUAMS-14545), which likely attributed it to the Glazkovo tradirion.

#### Kitoi and other Early Neolithic burial traditions (Russia_Cisbaikal_Kitoi_MN)

##### Rasputino site

The village of Rasputino is situated on the left bank of the Angara River, 450 km downstream from the city of Irkutsk, on a 15-meter terrace that extends from the mouth of the Igirma River and extends far beyond the village. Upstream from the village, the terrace is intersected by numerous waterholes and rises steeply above the floodplain. It gradually merges with the upland of the bedrock coast to the southeast. Over the years, extensive plowing has caused significant damage to the terrace slopes and resulted in the destruction of ancient burials. However, two burials were accidentally discovered by local residents on the sides of waterholes.

###### Burial 2(?) (individual ID I13679, male)

The skeletal code of the individual I13679 in the Tomsk Anthropological Collection refers to **mound 13, burial 2**, excavated by Georgy Sosnovskiy in 1921. However, Georgy Sosnovskiy never excavated any mounds and even burials at Rasputino; he visited the village and collected information about two burials found by E.G. Rasputin, a resident of the Rasputino Village, in 1903 (Okladnikov 1976). We suppose mislabeling happened and treat individual I13679 as a skeleton from **burial 2** at the Rasputino site.

According to Georgy Sosnovsky's account, which was collected from E.G. Rasputin in 1921, the burial was initially found in 1903, located one verst (~1.1 km) from the village upstream along the Angara River, in a hollow on the cultivated land of E.G. Rasputin, approximately 1/2 arshin (~35 cm) from the first burial.

The bones of the skeleton from this burial were found to be stained red with ocher. The burial orientation was similar to the first one, with the skull facing northwest and the body in a supine position. According to Georgy Sosnovsky, the following items were discovered in this burial:

1. A bone needle case made from a tubular bird bone, containing two bone needles decorated with a longitudinal ornament of zigzag rows of dashes.
2. Fragments of a tetrahedral bone stick with 57 parallel notches on one of the narrow faces (possibly a counting stick or "tag"). One end of the stick is tapered.
3. Bone awls (4 pieces).
4. A curved dagger made of horn, serving as a piercing tool.
5. Two small wedge-shaped jade axes.
6. Two oblong jade knives with pointed edges.
7. A jade chisel.
8. Two sandstone whetstones.
9. Two oblong pebbles used as pestles for grinding pigments.
10. A chisel-shaped bone tool.
11. Pieces of red ocher.
12. Several fragments of bone tools and unfinished bone artifacts.
13. Additionally, two wild boar tusks were found resting on the neck of the skeleton. The artifacts were positioned along the sides of the skeleton (Okladnikov 1976).

Unfortunately, the collection is lost. The funeral rites and the typology of the finds support the Kitoi attribution of the burial (Georgievskaya 1989). The skeleton from the burial is dated to 5731–5635 calBCE (6800±30 BP, PSUAMS-9118).

##### Manzurka-2 site

The burial ground is located on the Manzurka River, on the second terrace above the floodplain. The elevation of the terrace ranged from 1 to 10 m above the water's edge. In 1974, Aleksey Okladnikov conducted excavations, uncovering 5 burials in total (Aseev, 2009).

###### Burial 2 (individual ID I0999, male)

The burial was positioned on the slope of the terrace, approximately 100 m south of burial 1. Three small stones protruding from the turf marked its presence on the surface. Upon removing the sod, the complete outline of the mound became visible. The mound exhibited a semi-oval shape and was constructed using small, slightly rounded pieces of limestone compactly laid out in a single layer. The dimensions of the stone mound measured 90 cm from north to south and 50 cm from west to east. Subsequent excavation beneath the mound's base, at a depth of 10 to 12 cm, revealed the femurs and leg bones, indicating that the buried individual was laid on their back and oriented towards the north. The upper half of the skeleton was absent, suggesting evidence of looting. However, within the pelvic region on the right side, a miniature arrowhead with a tooth-like decoration and deep recesses just above the straight attachment on the tip was discovered. It is presumed that this tooth-like feature served as a means to secure the tip to the shaft, which was split from above. Furthermore, during the excavation beneath the skeleton, a small plate knife made of green Baikal jade was found. One half of the oval blade exhibited delicate retouching on both sides (Aseev, 2009). The skeleton is radiocarbon-dated to 5831–5673 calBCE (6870В±25 BP, PSUAMS-4215).


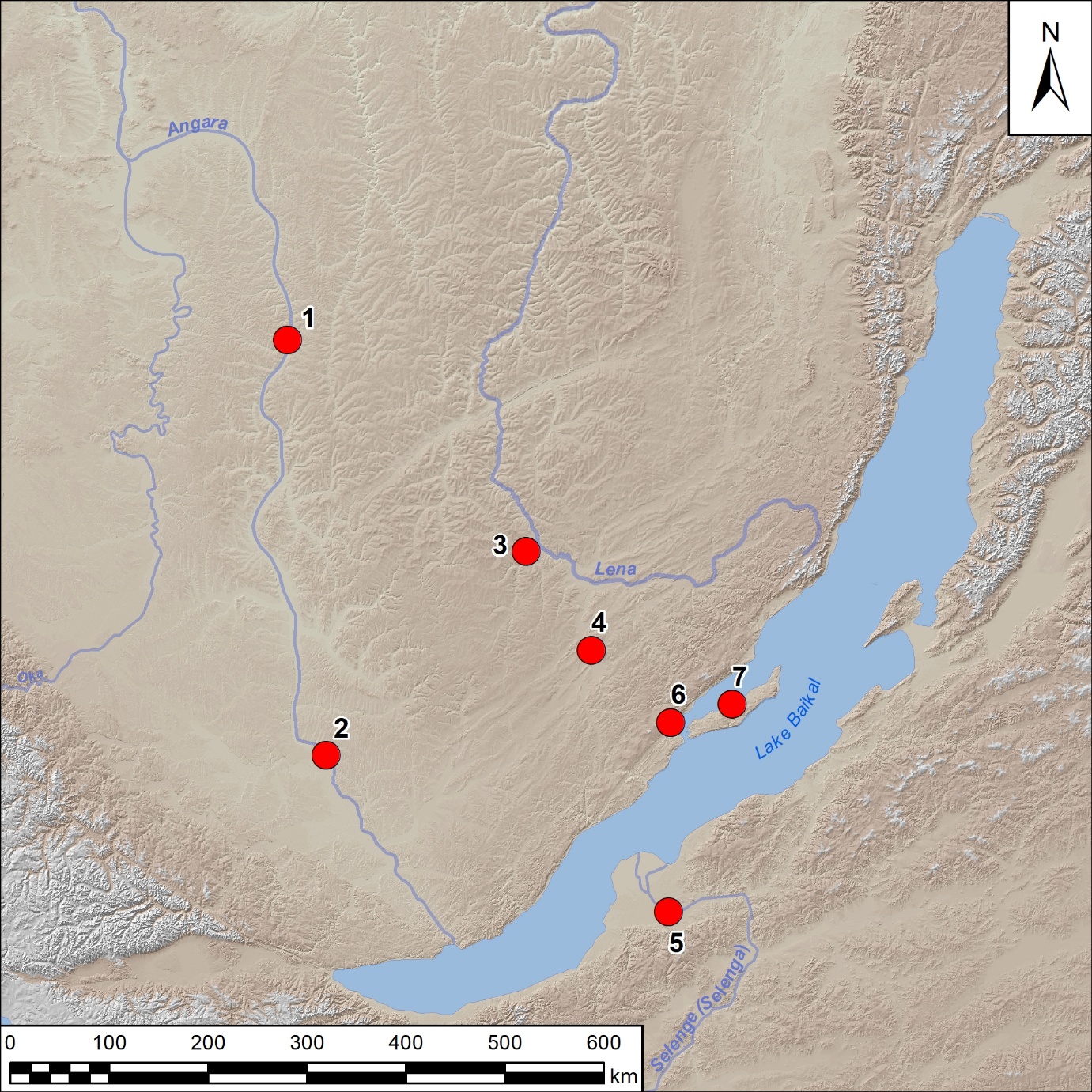


***Figure S6.*** *Neolithic sites in the Baikal region. 1 – Rasputino; 2 - Ust'-Belaya; 3 – Obkhoy; 4 - Manzurka-2; 5 – Fofonovo; 6 - Ulyarba-2; 7 - Khuzhir-2.*

#### Serovo and Isakovo Neolithic burial traditions (Russia_Cisbaikal_SerovoIsakovo_N and Russia_AngaraRiver_SerovoIsakovo_N)

##### Ust’-Belaya site complex

Situated at the confluence of the Belaya River into the Angara River, approximately 110 km northwest of Irkutsk, the Ust-Belaya site represents a multi-phase archaeological complex. It was subjected to investigations by various scholars at different times. Nina Gurina and Liya Krizhevskaya conducted initial investigations in 1957, followed by German Medvedev and Galina Georgievskaya in 1962, and subsequently by Galina Georgievskaya from the late 1960s to the 1990s (Georgievskaya 1989).

The site has yielded a diverse assemblage of over 20 cultural ceramic complexes, primarily associated with the Neolithic epoch, alongside artifacts from the Bronze and Iron Ages. Notably, burials have been identified in both the northern and southern sectors of the site. The northern area revealed four investigated burials, while the southern sector unveiled and excavated 17 burials.

These burials encompass distinct groups that are attributed to the Kitoi, Serovo-Isakovo, and Glazkovo complexes. The Kitoi burials were published by Galina Georgievskaya in 1989.

###### Unknown/unidentified burials (individual IDs I8296 and I8298, identified as females)

We publish genetic results from two burials from the Ust’-Belaya site, individual IDs I8296 and I8298. Unfortunately given the state of the records, it is not possible to attribute them to specific burials from this site. Radiocarbon analysis of individual ID I8296 yielded an estimated age range of 2860–2570 calBCE (4105±20 BP, PSUAMS-5480), while individual ID I8298's dating indicated an age range of 3790–3690 calBCE (4970±20 BP, PSUAMS-5481). The date of individual I8298 aligns within the chronological span of the Late Neolithic burial practices of the Serovo and Isakovo traditions, while the date of individual I8296 suggests the Glazkovo cultural attribution.

#### Glazkovo Early Bronze Age culture (Russia_Cisbaikal_Glazkovo_EBA and Russia_AngaraRiver_Glazkovo_EBA)

##### Obkhoy burial ground

The Obkhoy burial ground, spanning the Early Neolithic to Bronze Age, was brought to light during archaeological investigations along the Upper Lena River by Aleksey Okladnikov in 1971. It occupies the southwestern portion of the right estuarine region of the valley, with an elevation of approximately 15 meters above the water level of the Kulenga. Upon initial examination, the site had already experienced partial degradation due to human activity.

Based on distinct burial customs and accompanying artifacts, Okladnikov attributed this site to the Glazkovo culture, although no publications were made on the findings. Resuming research efforts in 2017-2018 under the guidance of V.I. Bazalinsky, further exploration was conducted. During the 2018 phase of the project, two burials, presumably from the Early Neolithic period, were identified along the borders of the previous excavations.

###### Burial 7 (individual ID I1000, male) and burial 13 (I1001, male)

We publish genetic results for two burials from the Obkhoy burial ground, **burial 7** (individual ID I1000) and **burial 13** (individual ID I1001). The skeleton from **burial 7** is dated to 2872–2581 calBCE (4132±32 BP) [R_combine: (4100±40 BP, Poz-83436); (4180±50 BP, GIN-4121)] (Mamonova and Sulerzhitskiy, 1989). Human bones from **burial 13** are dated to 3087–2895 calBCE (4350±36 BP) [R_combine: (4340±50 BP, Poz-83437); (4360±50 BP, GIN-4122)] (Mamonova and Sulerzhitskiy, 1989).

##### Ulyarba site

The Ulyarba burial ground is situated in the Olkhonsky district of the Irkutsk region, 188 km northeast of the city of Irkutsk and 4 km southwest of the village of Sarma. The ancient burials were positioned on the slope of the mountain, at the base of Cape Antukhay on the northwest coast of Mukhor Bay in the Small Sea of Lake Baikal. Leonid Zyablin discovered and partially investigated the burials in 1959, with a total of 28 burials excavated. Based on the burial locations and certain ritualistic characteristics, Leonid Zyablin identified four burial groups as Ulyarba 1-4 cemeteries. Within the Ulyarba-1 burial ground, the discoverer attributed 5 burials (1-3, 16, 19), four of which were aligned in a row along the slope at its base. The primary concentration of burials, situated 50 m up the slope from the Ulyarba-1 burials on the next terrace-like ledge, was designated as Ulyarba-2. Leonid Zyablin identified Ulyarba-3 (burials 25-28) 15 m to the north of the latter, and Ulyarba-4 (burials 21-22) 60 m to the west. The Ulyarba 1-2 cemeteries were assigned to the Bronze Age, Ulyarba-3 to the Serovo culture of the Neolithic period in the Baikal region, while the dating of the Ulyarba-4 site was not determined. In subsequent years, the Ulyarba-4 burials were attributed to the Slab Graves of the Early Iron Age. In 1976, Olga Goryunova resumed excavations of the Ulyarba burial ground, unearthing 6 burials, and the site was later studied by V.I. Smotrov in 1983 (6 additional burials were unearthed). Based on their locations, the excavated burials from 1976 and 1983 were assigned to the Ulyarba-2 burial ground, although some of them, due to the characteristics of their burial complexes, can be traced back to the Neolithic period (Serovo culture). Since the Neolithic and Bronze Age burials were situated on the same territory, O.I. Goryunova chose not to separate them into distinct burial grounds, but considered them as a single chronologically diverse necropolis. In total, 40 burials from the Neolithic to Paleometallic periods were unearthed at the burial ground throughout the years of investigation (Goryunova 2002).


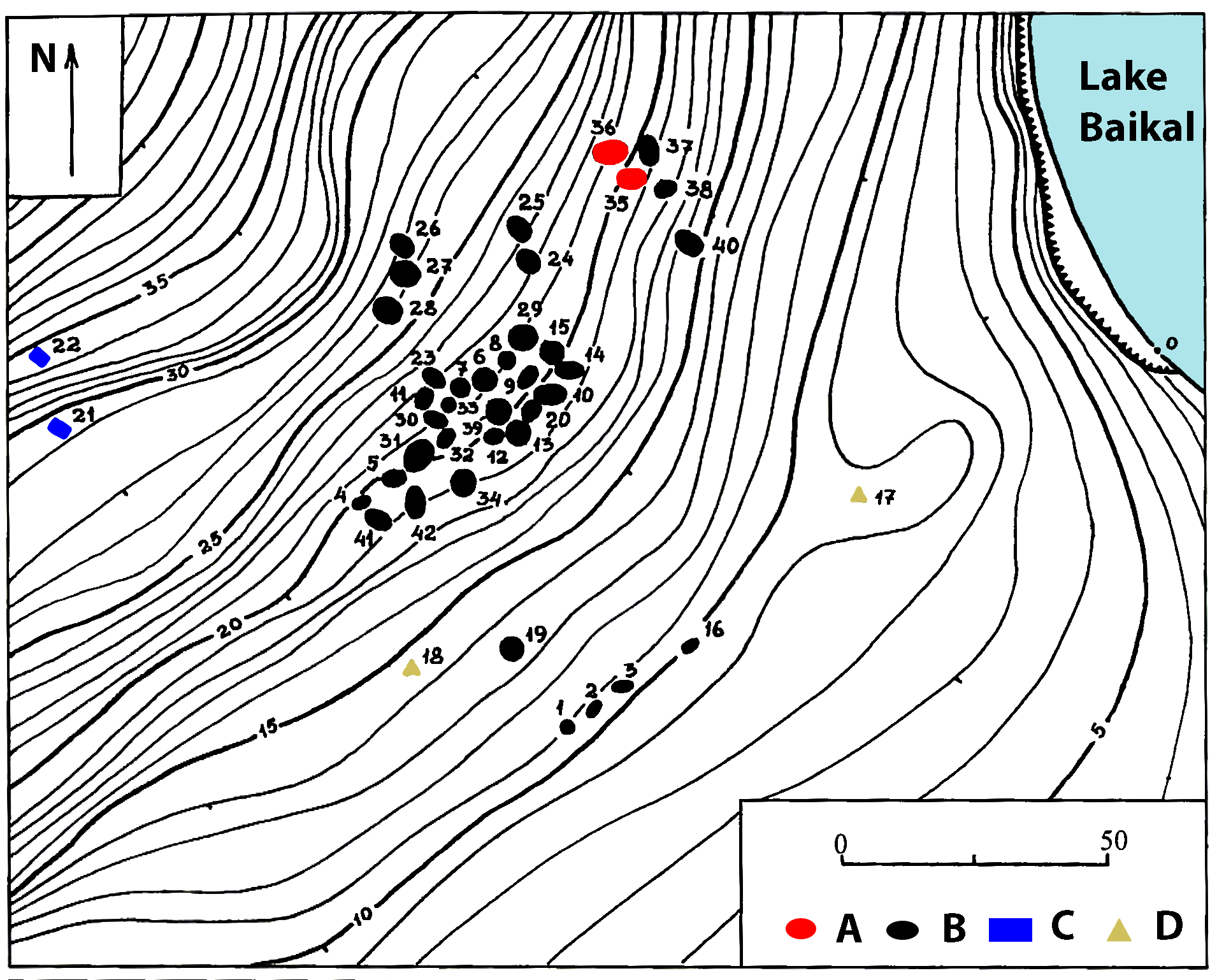


***Figure S7.*** *Map of Ulyarba site.* ***A*** *- sequenced burials;* ***B*** *- other Neolithic and Bronze Age burials;* ***C*** *- Early Iron Age burials;* ***D*** *- rocks (by Goryunova 2002, modified).*

###### Burial 35 (individual ID I2133, male)

Excavated in 1976, field number 5. The rubblework structure is oval-shaped, elongated along the southwest-northeast axis. The dimensions of the construction are 7.2 x 4.8 m, built from stone slabs arranged in several layers. Remains of animal bones (possibly canine) are found beneath the slabs of the rubblework. At the lower epiphysis of the second femur bone of the animal, an arrowhead is discovered. Three ceramic fragments are noted 0.36 m east of the pelvic bones. Beneath the animal bones, a hearth measuring 0.53 x 0.31 m is found, with a second ash spot located 0.12 m north of it. These hearths are situated on the slabs forming the interior covering of the burial.

The interior covering is a solid flat surface made of slabs, oval-shaped with dimensions of 2.0 x 1.0 m, oriented along the southwest-northeast axis. Beneath the covering slabs lies the burial. The skeleton is in good condition, with only the skull disturbed, lying 0.11 m above the other bones. The burial likely belongs to a woman aged 30-35 years. The grave is delimited by vertically standing slabs at the head and feet. The skeleton is stretched in the supine position, with the head facing southwest, the arms extended along the torso, the left hand drawn under the pelvis, and the legs in an extended position.

The burial inventory consists of a disc made of light jade and four clusters of tools. The disc, with a diameter of 8.2 cm, is found on the right shoulder bone. The first cluster of tools is located at the deceased's waist on the right side and likely packed compactly, possibly in a bag. It includes a polished, leaf-shaped southwest-northeast, a leaf-shaped spearhead with bifacial processing made of flint, two bone tools of the chisel type, a bone needle with an ear, two bone tool fragments, and a bone frame with a lateral groove for inserts. The second cluster of items, found at the waist on the left side, comprises a fishing hook and two irregularly oval-shaped bronze plates (rivets?). A retouched bifacial point on a flint flake and a triangular arrowhead are found 0.25 m north of them. The third cluster consists of nine triangular flint arrowheads, located near the left shoulder bone. The fourth cluster, situated to the right of the skull, includes 24 flint arrowheads, a retouched jade axe, an abrasive grinding stone made of fine-grained slate, three flakes, and three pendants made from sika deer teeth (Goryunova et al., 2004). The burial is dated to 2623–2458 calBCE (4000±35 BP, Poz-82201).


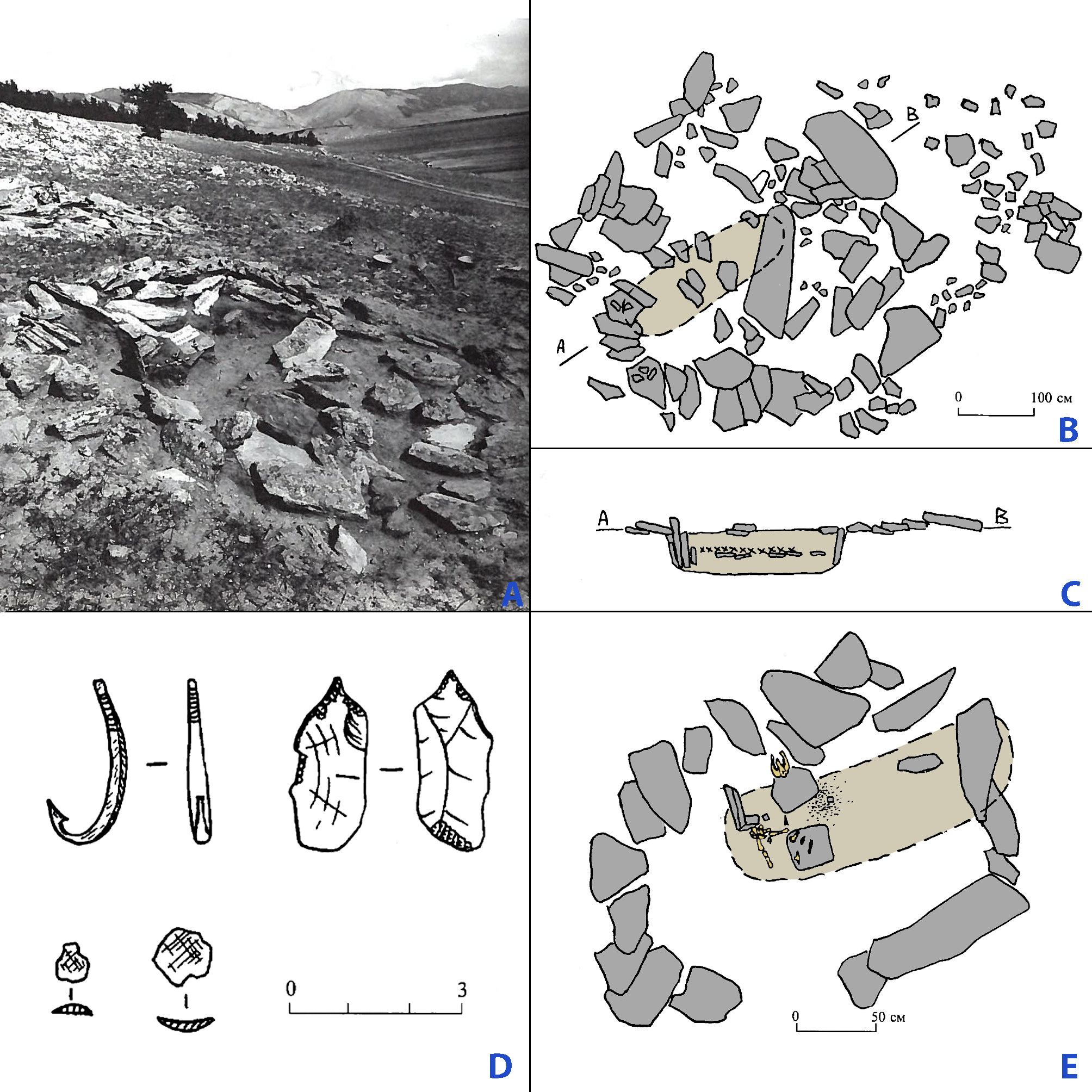


***Figure S8.*** *Ulyarba site.* ***A*** *- photo of burial 35 before excavation,* ***B*** *- plan of the rubblework;* ***C*** *- section of the burial; D - finds; E - canine skeleton position in the grave (by Goryunova et al., 2004, modified).*


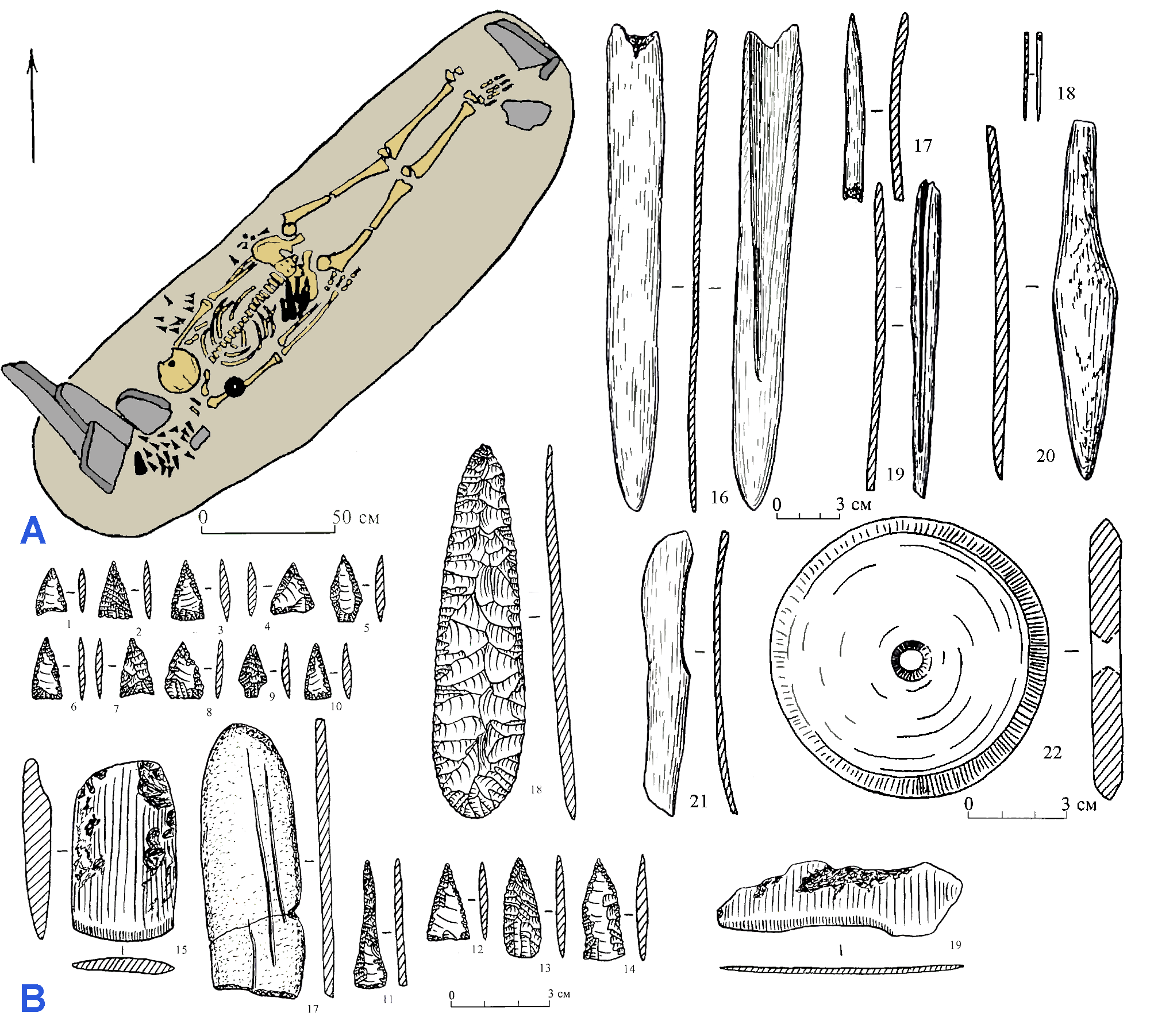


***Figure S9.*** *Ulyarba site, burial 35. A - plan of the burial; B - grave goods (by Goryunova et al., 2004, modified).*

###### Burial 36 (individual ID I2135, male)

Excavated in 1976, field number 6. The burial rubblework structure is circular, oval-shaped, and elongated along the west-east axis. It is constructed from stone slabs arranged in several layers, with dimensions of 6.0 x 4.8 m. Within the outlined area of the rubblework, single slabs, likely displaced from the walls, are encountered. The dimensions of the enclosed inner space are 4.0 x 3.0 m, with vertically standing slabs noted at the western and eastern ends. Similar slabs are fixed at the eastern end. Between these slabs, at the center of the structure, a grave pit (0.80 x 2.00 m) is discovered, oriented along the southwest-northeast axis.

In the southwest end of the grave pit (at the base of the vertically standing slabs), at a depth of 0.50 m from the contemporary surface, flint chips and fragments of animal ribs and vertebrae are found. At a distance of 0.50 m north of them, a shoulder blade of the same animal is recorded.

The skeleton of a human, approximately 20 years old, is discovered in the grave pit at a depth of 0.50 m from the contemporary surface (pit bottom). The burial is partially disturbed, with scattered fragments of the skull, ribs, cervical, and thoracic vertebrae observed in the southwest part of the grave. The bones of the arms and lower half of the torso (from the lumbar vertebrae) are in anatomical order. The arms and legs of the buried individual are placed in an extended position.

Based on the preserved bones, it appears the deceased was placed in a stretched position on their back, with the head facing SW.

Accompanying inventory is noted near the upper epiphysis of the right femur, including a polished jade blade and a fragment of a bone tip. Near the left elbow joint, two fish vertebrae are recorded (Goryunova et al. 2004). The burial is dated to 2864–2496 calBCE (4092±32 BP, OxA-33162).


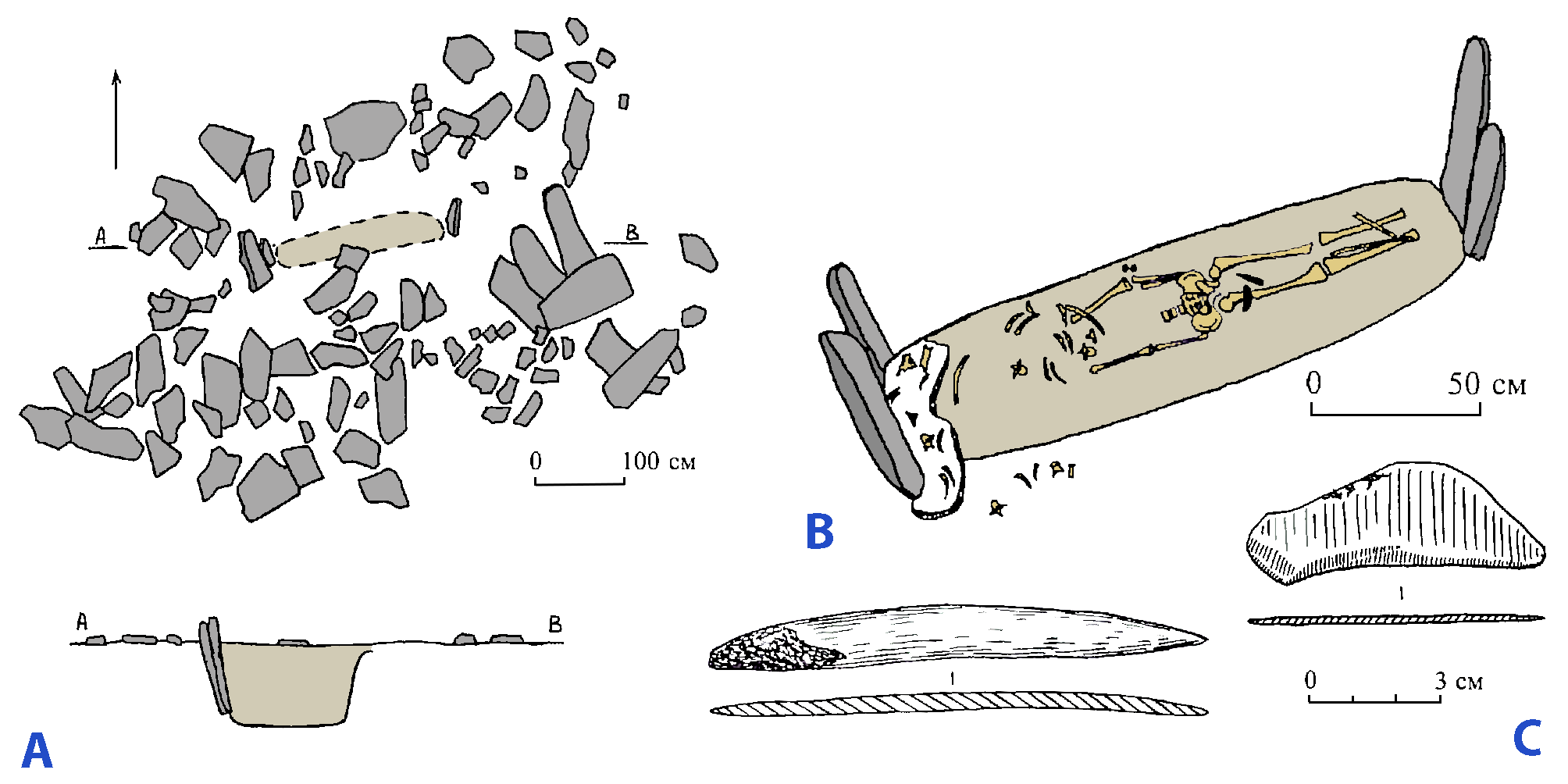


***Figure S10.*** *Ulyarba site, burial 36. A - plan and profile of the burial structure, B - plan of the burial, C - grave goods (by Goryunova et al., 2004, modified).*

##### Khuzhir site

###### Burial 2 (individual ID I0998, male)

From the Khuzhir burial site, we publish genetic data on the individual I0998 from **burial 2**, dated to 2836–2468 calBCE (4040±35 BP, Poz-83426). A combination with a previously published date from the same burial (2871–2610 BCE (4140±50 BP, GIN-4098) (Mamonova and Sulerzhitskiy, 1989) demostrates chronological interval 2851–2491 calBCE (4073±29 BP) [R_Combine: (4040±35 BP, Poz-83426), (4140±50 BP, GIN-4098)].

### Lower and Middle Angara

#### Early and Middle Neolithic sites (Russia_MiddleAngara_Kitoi_MN)

##### Sosnovy Mys site complex

The Sosnovy Mys site (or archaeological site complex) was first discovered and explored by Nikolai Drozdov in 1974-1975. The term refers to a multiphase occupation site and two burial grounds, Sosnovy Mys 1 and 2. The site complex is located on a 6-8 meter alluvial terrace on the left bank of the lower part of Sosnovy Island, stretching along the course of the Angara River, opposite the mouth of its right tributary, the Kata River. The island's surface is densely covered with pine forest, moss, and grassy litter.

The ***Sosnovy Mys-1*** burial ground was explored and excavated by Nikolay Drozdov in 1974–1975, and the excavations revealed 8 burials. One more burial was excavated in 1987 by Vasily Privalikhin. Archaeological excavations resumed in 2011-2012 as part of preparations for the flooding of the Boguchanskaya hydroelectric power station, uncovering over 8,000 m2 of the site. The investigations revealed a complex multi-layered habitation area and a burial ground spanning different periods, totaling 22 burials. Currently, there are 31 excavated burials at the site, with at least 16 of them belonging to the Neolithic period (Savelyev et al., 2020; Petrova et al., 1976; Privalikhin et al., 2013).

The ***Sosnovy Mys-2*** burial ground. In 1982, Vasily Privalikhin conducted reconnaissance investigations in the lower section of the island, leading to the identification of a new archaeological site named Sosnovy Mys-2. These investigations yielded the discovery of three burials, with one attributed to the Bronze Age, another to the Early Iron Age, and a third burial, potentially dating back to the Neolithic period, which unfortunately had been destroyed. The Sosnovy-Mys-2 burial ground is situated on a 10-12 meter terrace on the left bank of the island, 2 km above its lower end, where Sosnovy Mys-1 is located (Savelyev et al., 2020; Petrova et al., 1976; Privalikhin, 1998; Privalikhin et al., 2013).

###### Sosnovy Mys-1, burial 5 (individual ID I2134, male)

**Burial 5** was discovered in 1974 at a depth of 1.19 meters from the surface. The skeletal remains were found in a supine position with the body fully extended. The upper portion of the skeleton was partially disturbed by the grave pit of burial 4, while the lower part was covered with a layer of ocher. The funerary assemblage associated with the burial includes a flint knife, an unfinished arrowhead made of chalcedony, an ornament crafted from a musk deer tusk, a fragment of a bone projectile point, and a pendant made from a deer tusk (Petrova et al., 1976). There were no traces of rubblework above the grave, and it was overlaid by another burial, where the rubblework was recorded. This fact supports the attribution of burial 5 at Sosnovy Mys-1 to the earliest phase of the site, probably related to the Kitoi period. The burial is dated to 5718–5556 calBCE (6710В±40 BP, OxA-33163).

#### Serovo and Isakovo Neolithic burial tradition (Russia_MiddleAngara_SerovoIsakovo_LN)

##### Sosnovy Mys site complex

###### Sosnovy Mys-1, burial 9 (individual ID I1961, male)

**Burial 9** was found at a depth of 25 centimeters from the soil surface, a compact arrangement of stones in the shape of an elongated oval was discovered, stretching from the southeast to the northwest and encircled by a ring of large stones and slabs. Digging further, at a depth of 70 centimeters from the contemporary soil surface, the skeletal remains of a buried man were uncovered. The preserved dimensions of the grave pit measure 133x64x42 centimeters. The skeletal position of the man indicates that he was laid on his back, with arms extended parallel to the body, in an extended posture. The burial orientation was observed with the head facing southeast and the legs pointing northwest, corresponding to the downstream direction.

Accompanying the male skeleton were 16 grave goods, comprising various items and tools crafted from stone, horn, bone, and ceramics:

1. A chisel made of silicified shale, measuring 16.7 x 4.7 x 3.2 centimeters, was found on the bones of the chest and the thoracic spine and was turned with the dorsal side up.
2. Three sherds of a single pottery vessel adorned with dotted-comb ornamentation and pits.
3. A perforator fashioned from the slate bone of an adult elk.
4. Two antler rods of rectangular cross-section, with unknown purpose.
5. Nine flint arrowheads.
6. A beaver chisel, presumed to be an ornament or an amulet.

The presence of a rubblework, the specific skeletal position of the man on his back with extended arms, and the orientation with the head to the southeast, together with the assemblage of accompanying artifacts, lead us to attribute this burial to the spectrum of sites associated with the Serovo Neolithic culture of the Baikal region (Privalikhin et al., 2013). The date of the burial falls between 4239–4002 calBCE (5297±25 BP) [R_combine: (5320±40 BP, Poz-82202); (5283±32 BP, OxA-33488)].

###### Unidentified burial (individual ID I11107, male)

Another sampled individual from Sosnovy Mys-1 has individual ID I11107, but the burial number is unidentifiable. It was excavated in 1974 by Nikolay Drozdov, and may come from **burials** numbered from **1 to 4, or 6**.

There is no description available for **burial 1** (Petrova et al., 1976).

**Burial 2** consisted of a random heap of bones from a single individual, suggesting dismemberment or re-burial as part of the burial rite. Additionally, a vertebral column of another individual was discovered in the northeast corner of the grave pit. The grave goods associated with this burial included 17 pendants made from tubular bone and two points made from slate bone of a deer.

**Burial 3** was unfortunately destroyed due to slope erosion, leaving no further information available.

**Burial 4** was positioned within the grave pit of burial 5. The buried individual was found lying on their back with bent limbs, within a grave pit that measured 0.67 meters deep. During the clearing process, a bone needle case was uncovered beneath the pelvic bone.

**Burial 6** was located directly under the rubblework boulders, without any evident grave pit. It consisted of the skeleton of one individual and the skull of another. The skeleton was positioned on its right side, with the arms bent at the elbows and lying parallel to the right side of the body, and the legs bent at the knee and hip joints. The tibia of the right leg had been cut open. The burial was double, as indicated by the presence of skull 2 next to skull 1. The buried individuals were positioned on their backs with their legs bent at the knees. Five flint arrowheads were found between the bones, and a fragment of a large flint spearhead was discovered near one of the skeletons.

Given that all the described burials exhibit similar burial rites, resembling **burial 9**, it is probable that individual ID I11107 can be associated with the Serovo culture of the Baikal region.

##### Kamenka-1 site

The archaeological site of Kamenka-1 is situated on a limestone cape positioned between the channels of the Angara and Kamenka rivers. The cliff rises approximately 21 meters above the water level and extends for over 150 meters in a west-to-east direction, displaying an approximatelytriangular shape on the ground. The upper platform of the cape is relatively flat, covered with turf, and slopes downward towards the west, widening to about 100 meters. In 1997, Aleksandr Zaika uncovered a burial on the eastern edge of the cape.

###### Single burial (individual ID I10900, male)

At a depth of 25 centimeters, stonework consisting of medium-sized rock fragments was discovered. The rubblework exhibited an elongated shape, with its long axis aligned along a northeast-southwest line. Below the second layer, a well-preserved complete skeleton of an adult male, estimated to be 25-30 years old and 166 centimeters tall, was revealed.

Reconstruction of the funeral rite suggests that following the burial activities and placement of grave goods, the body of the deceased was initially positioned along the sides of the grave and subsequently placed on top, covered by large, flattened rock slabs. The stones were predominantly arranged in a vertical manner. The material used was likely comprised of large rock fragments that were exposed and uncovered during excavation. After the placement of the larger stones, smaller boulders were used to line the grave. The soil was carefully selected and poured over the masonry, forming a tomb mound characterized by dark-gray humus sandy loam. At a later stage, a funeral fire, possibly a ceremonial component, was laid in the northern part of the grave mound, specifically at the center of the mound's edge.

The skeleton was found in a fully anatomical order, lying in an extended position on its back. The orientation of the burial placed the individual's head towards the northeast (downstream the Kamenka River) and the feet towards the southwest, downstream the river bend. The burial location is in proximity to hangars located in this section of the channel. The head of the buried person rested on a stone "cushion" formed by an inclined sandstone slab of non-local origin. Due to the subsidence (vertical downward displacement of stones) of the masonry, the skull had turned to the left. Similarly, the middle part of the spinal column exhibited slight curvature and shifted towards the left side. The right arm extended alongside the body, with the humerus positioned within a recess formed by the rocks that delineated the grave pit. The phalanges of the hand were scattered near the right tibia. The left arm of the buried person was slightly bent at the elbow and placed on the belt. The bones of the hand and the phalanges of the fingers were randomly arranged in the pelvic region. The legs were parallel, extended, and brought together at the level of the feet. The bones of the feet were in a mixed state and were situated within a recess formed by the rocks.

Various grave goods were discovered in association with the burial. These include a polished knife with a concave blade (item 1), 32 fragments of horn used for bow lining found along the left side of the body, three stone arrowheads to the left of the right ulna (item 4), a retouched stone knife above the right pelvic bone and a polished adze below the left pelvic bone (items 2 and 3), a massive bone arrowhead near the northern wall of the grave pit at the belt level (item 5), two fragments of a bone puncture among the phalanges of the left hand in the pelvic area, three additional bone punctures located between the femurs (items 8-10), a bone harpoon positioned with its point downward near the buried individual's left thigh, and two overlaid bone tool blanks (items 11 and 12). Fragments of a broken earthen vessel were found near the northern wall of the grave at the knee level (item 13), and an accumulation of flakes and chips (40 pieces) along with bone objects including two awls, two rod blanks, and a prismatic core were discovered in the lower region of the right leg (item 14). Lastly, a horn imitation of a bear's claw or a boar's tusk was found next to the lower part of the right leg (item 15).

The burial is attributed to the Serovo culture and dated to the period earlier than 2750±120 calBCE, based on a date from charcoal from a campfire that overlaid the burial (4700±120 BP, СОАН-3780) (Zaika, 2009).


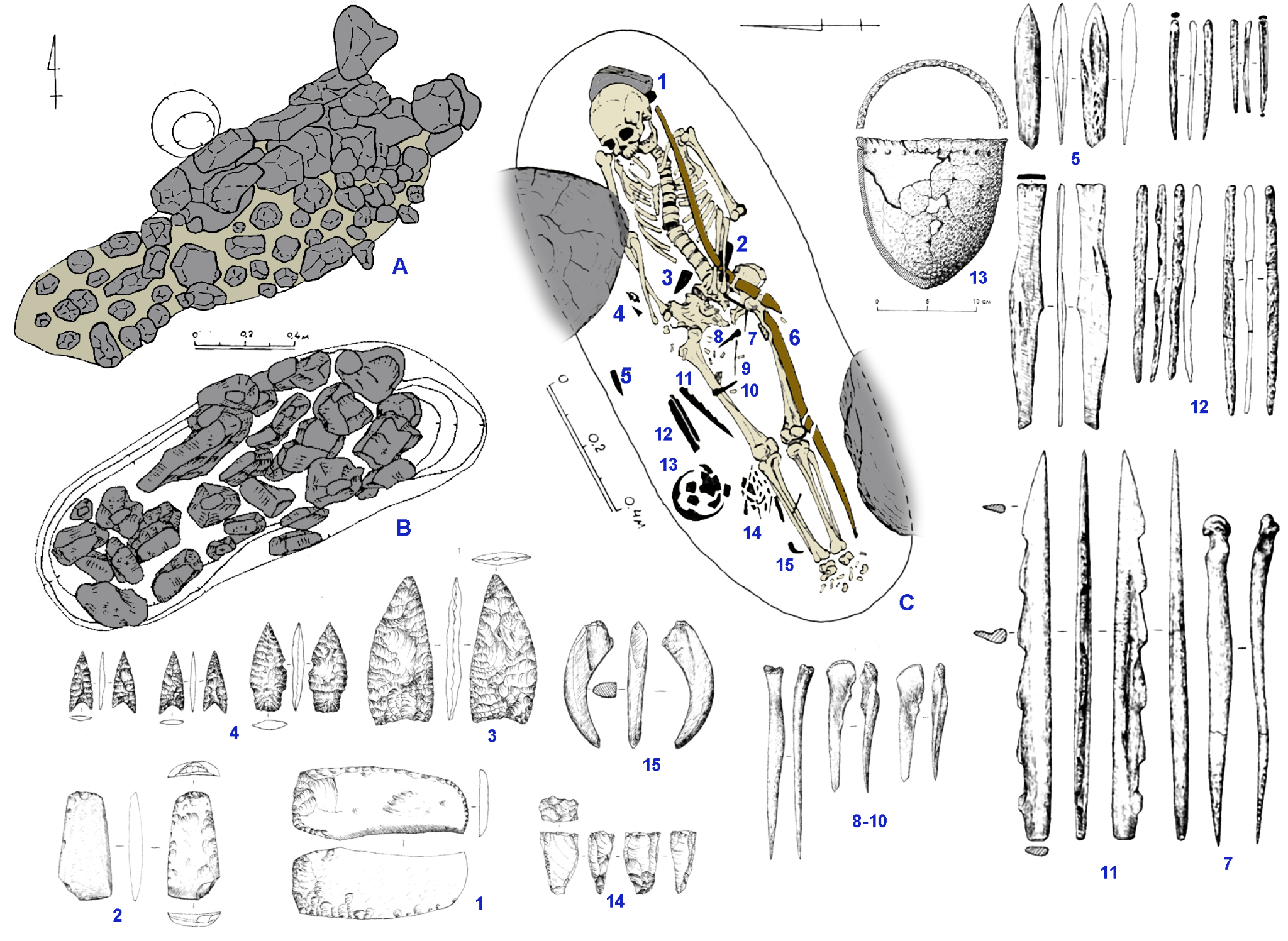


***Figure S11.*** *Burial at the Kamenka-1 site.* ***A*** *- rubblework, upper level;* ***B*** *- rubblework, lower level;* ***C*** *- burial:* ***1*** *- polished knife;* ***2*** *- chisel;* ***3*** *- retouched bifacial knife;* ***4*** *- stone arrowheads;* ***5*** *- bone arrowhead;* ***6*** *- antler bow plates;* ***7-10*** *- bone perforators;* ***11*** *- harpoon;* ***12*** *- bone tool blank;* ***13*** *- pottery vessel;* ***14*** *- accumulation of flakes, chips, cores, and bone awls;* ***15*** *- antler pendant (by Zaika, 2009, modified).*

#### Glazkovo Early Bronze Age culture along the Middle Angara (Russia_MiddleAngara_Glazkovo_EBA)

##### Sergushkin Ostrov site complex

Sergushkin Island (Sergushkin Ostrov), situated along the Angara River, spans 12 km and is densely covered with forest. The burial ground is located at the multiphase site, discovered by Vasily Privalikhin in 1974, in the central part of the island on its southwestern coast, approximately 2.5 km above the lower tip of the island. A total of seven burials were unearthed here. Three burials (1, 4, 7) are dated back to the early Iron Age, corresponding to the existence of the Tsepan archaeological culture. Three burials (2, 3, 5) are attributed to the Bronze Age, while one burial (6), characterized by a cremation rite, dates back to the 13th century CE (Privalikhin, 2009).

###### Sergushkin-3, burial 2 (individual ID I5280, male)

**Burial 2** was identified by the presence of rubblework stones protruding from beneath the turf on the gentle slope of the terrace, which gradually descends towards the river on the northwestern side of the island. A cluster of 20 masonry stones formed a small irregular oval shape measuring 114x93 cm. At a depth of 25 cm below the surface level, beneath a large masonry stone situated on the western side, the crushed skull of a male individual, aged 30-35 years, was discovered. The burial position was on the left side, with the right temporal side of the skull exhibiting fractures caused by a blow from above with a stone. The lower portion of the stone corresponded precisely in shape and size to the dent on the skull. The skeleton of the individual was found in a crouched position on the left side, with the knees bent and the arms pressed against the chest. The hands were positioned near the eye sockets and lower jaw. The general orientation of the skeleton was with the back facing the river and the head pointing downstream. The femurs, tibias, humerus, and radius bones were fractured. Additionally, a bone needle with a deliberately broken eye and sting, measuring 6.9x0.2 cm and up to 1.5 cm across, was found at the knee joint of the right leg. This burial is attributed to the Glazkovo culture of the early Bronze Age (Privalikhin, 2009).

###### Sergushkin-3, burial 5 (individual ID I20310, female)

**Burial 5** was uncovered at a depth of 45 cm below the modern surface level, below the layer attributed to the Early Iron Age. It was covered by 54 stones and slabs forming rubblework. It had an elongated oval shape, oriented along a southeast-northwest line, measuring 200 cm in length and 155 cm in width. At a depth of 110 cm from the soil surface, within an oval grave pit, the skeleton of a female individual aged 55-60 years was discovered. The skeleton was positioned on the left side, in a crouched posture, with the arms and legs tightly bent at the elbow and knee joints, pressed against the chest. The orientation of the skeleton was with the back facing the river and the head aligned with its course, i.e., to the northwest. A rich array of accompanying goods was found within the grave, including pottery vessel with handles, a needle case made of bird bone, a bone awl-piercer, two bone needles, three discs of calcite (marble?), a ring made of white jade, thirteen large beads of calcite, a roe deer bone, two flakes of siliceous rock, and fish vertebrae. This burial is attributed to the Glazkovo culture of the early Bronze Age (Privalikhin, 2009).

##### Ust’-Shamanka-1 site

###### Single burial (individual ID I2143, male)

The site was excavated in 1978 by V.N. Sokolov, who unearthed six burials, compactly placed in rows. Archaeological artifacts found in graves demonstrate similarity to the Early Bronze Age sites of the Angara region, such as Sosnovy-Mys and Sergushkin-3 (Dudarek, Lokhov 2014). Only one individual (individual ID I2143) was sequenced. Unfortunately, the excavators did not label the anthropological material from the site and we failed to identify the burial number to provide detailed observation of the archaeological context. However, the combination of two radiocarbon dates shows the chronological range of 2841–2494 calBCE (4071±21 BP) [R_Combine: (4040±25 BP, PSUAMS-9085); (4130±35 BP, Poz-82203)]. The chronological attribution of the burial as well as the presence of the Glazkovo-attributed graves at the site makes the Glazkovo attribution of the studied material the best guess.

##### Sosnovy-Mys site complex

See the description of the site complex above.

###### Sosnovy Mys-2, burial 1 (individual IDs I20308 and I20309, identified as males)

The construction of the burial consisted of an oval elongated rubblework oriented along the east-west axis. The outer edges of the construction comprised the largest and heaviest boulders and slabs, while the central part was filled with smaller stones.

At a depth of 65 cm from the soil surface and 26 cm from the lower level of the construction, human bones (individual ID I20309) were discovered in a rounded grave pit measuring 88 x 80 cm and located 60 cm deep. The skull, along with the bones of arms, legs, pelvis, vertebrae, and ribs, were neatly arranged at the bottom of the grave pit, without proper anatomical order. It is presumed that initially, the corpse of a 20-25-year-old individual remained exposed unburied for a certain period. After the decay of muscle ligaments and tissues, the bones, together with accompanying grave goods, were collected and entombed.

**Burial 1**, with its grave pit, cut through and disturbed another earlier burial, likely dating back to the Late Neolithic period. On the bottom of the disturbed Neolithic burial's grave pit, located on the southeast side of **burial 1**, fragmented leg bones (individual ID I20308) and calcaneal bone were found.

The fill of the grave pit of **burial 1** contained small charcoal fragments and various grave goods that were discovered near the skull, between the skeleton's bones, and underneath them. These grave goods included ceramics, stone and bone tools, as well as stone ornaments and animal tooth aplliques (Privalikhin, 1998).

## South part of West Siberia and the Altai-Sayan

### Yenisei River Basin

#### Yenisei Forest-Steppe Neolithic (Russia_Krasnoyarsk_N and Russia_UpperYenisei_N)

##### Afontova Gora Neolithic burials

The Neolithic burial ground is situated in the vicinity of a Paleolithic site Afontova Gora-2. The multilayer Paleolithic site was initially discovered by Ivan Savenkov in 1884. In 1924, Nikolay Auerbach, Georgy Sosnovsky, and Valerian Gromov conducted excavations and investigated the first burial, which was possibly attributed to the Neolithic. Another nearby burial was unearthed in 1932 by A.F. Katkov and V.G. Kartsov from the Krasnoyarsk Museum. In 1937, Aleksey Okladnikov and a museum employee V.I. Neshumaev excavated another burial located 3 meters away. Additionally, in 1977, schoolchildren transferred artifacts from a destroyed burial at Afontova Gora to the museum.

All the burials, comprising a minimum of 5 individuals, are positioned on the edge of the Afontova Gora slope, approximately 20 meters from the Yudin Museum estate. The estimated area of the Neolithic burial ground on Afontova Gora is between 200 and 300 square meters. The burials themselves are compactly located within a few meters distance from each other. The skeletons are oriented perpendicular to the Yenisei River but parallel to the ancient terrace of the Kacha River.

Among the significant grave goods found at the site are bone pendants depicting ducks, needles and needle cases, awls adorned with anthropomorphic images, harpoons, mother-of-pearl disc-shaped beads, stone leaf-shaped arrowheads, and clay round-bottomed vessels with surface ornamentation.

###### Neolithic burial С (individual ID I13674, male)

In 1932, A.F. Katkov excavated a Neolithic burial at Afontova Gora. The grave was situated on a small hill that terminates in cliffs on both the southwestern and southern sides. No discernible traces of the grave pit or burial structures were found. The burial was located at a depth of 40-85 cm, likely within a hole oriented from south to north, inferred from the alignment of the bone remains (skull, pelvis, thigh). The skeleton exhibited a slight reddish discoloration, possibly due to the presence of ocher.

The grave goods recovered from the burial include pear-shaped pendant decorations, animal teeth fashioned in the form of ducks, and flat iridescent disc-shaped beads.

From the 1932 excavation conducted by A.F. Katkov at Afontova Gora, a braincase belonging to an individual aged 30-35 has been preserved. The craniological features of the braincase suggest a combination of Caucasoid and Mongoloid traits. According to A.F. Katkov, the burial can be dated to the early stage of the Paleometallic Age and is contemporaneous with the burial at Bazaikha, as revealed by excavations led by Sergey Sergeev (Okladnikov, 1949, 1957; Vdovin et al., 2016, Savenkova and Makarov, 2018; Berdnikov et al., 2022).

Radiocarbon dating conducted on the human skeleton recovered during A.F. Katkov's 1932 excavation yielded a calibrated date between 4673–4461 calBCE (5720±25 BP, PSUAMS-7666).

##### Tolsty Mys-1

The Tolsty Mys-1 site is situated on the first floodplain terrace, approximately 8 meters above the right bank of the river. This location comprises Neolithic cultural layers. In 2010, excavations were conducted in a 300-square-meter area within the settlement.

###### Single burial (individual ID I11102, male)

During the excavation of the Neolithic horizon, a single burial was documented. Within the base of the grave pit, the skeletal remains of a young man aged approximately 20-25 years (age estimation by Tatyana Savenkova) were found. The burial was performed in an extended supine position, with the head oriented in the downstream direction. The preservation of the bones is poor, and the temporal region of the skull exhibits damage. A patch of pink ocher was discovered in the vicinity of the skull, suggesting its ceremonial use.

In the occipital section of the burial, the fragmented remains of a ceramic vessel displaying imprints of a "braided net" design on its exterior surface were found. Some fragments from this vessel were recovered from the fill material within the grave pit. It remains unclear whether the vessel is directly associated with the burial or relates to a separate settlement layer. (Grevtsov et al., 2010; Grevtsov, 2010). The skeleton is AMS-dated to 5757–5636 calBCE (6825±30 BP, PSUAMS-7730).

##### Krasnoyarsk finds

###### Krasnoyarsk find (individual ID I11101, female)

The burial was initially discovered in June 1955 by a geologist, A.P. Balykov, on the left bank of the Yenisei River, near the summer dachas of the employees of the City Department of Public Education (GORONO) of Krasnoyarsk. It was situated on the first floodplain terrace, approximately 8 meters above the river's edge, directly opposite Sosnovy Island. In the same year, the excavation was conducted by Z.K. Glusskaya. The museum collection was analyzed by an anthropologist V.P. Alekseev in 1960. A reconstruction of the skull found within the burial was undertaken by M.M. Gerasimov and was subsequently reexamined by Vladimir Dremov and Tatiana Savenkova. The archaeological artifacts were studied by Nikolay Makarov, Ivan Berdnikov, and Aleksandr Vdovin.

The single burial (individual ID I11101) contained the remains of a woman estimated to be 30-35 years old. The body was interred in a supine position, with the orientation across the river, the head facing north, and the feet pointing south. Grave goods recovered from the burial included ornaments comprising flat, mother-of-pearl rounded beads, pendants crafted from animal teeth, plates made of boar tusks, and a needle case with threaded ends (Glusskaya, 1963; Vdovin and Makarov, 2016; Savenkova and Makarov, 2018; Alekseev, 1960; Solodovnikov et al., 2020; Dryomov, 1988; Kozintsev, 1974). The skeleton is dated to the period between 4793–4612 calBCE (5845±30 BP, PSUAMS-7549).

###### Ulitsa Karla Marksa (Karl Marx Street, Krasnoyarsk) (individual ID I11103, male)

During the archaeological monitoring of earthworks for the reconstruction of the heat collector in Karl Marx Street in Krasnoyarsk in July 2000, several significant archaeological complexes were discovered by A.Yu. Tarasov.

One of these discoveries occurred on the southern side of the collector trench, approximately 21 meters north of the northern facade of a residential building at Karl Marx Street 21. At a depth of 1.64 meters from the present-day surface, an area stained with ocher was identified. Further excavation revealed that the postcranial human skeleton was situated in soil saturated with ocher. Unfortunately, it was not feasible to fully expose the trench side due to a trading pavilion located above the burial. As a result, anthropological materials were extracted by carefully excavating the trench wall.

The excavated cranium originates from a single burial (individual ID I11103), devoid of any accompanying grave goods. Radiocarbon dating places this skeleton within the 95% confidence interval of 5962–5735 calBCE (6945±30 BP, PSUAMS-7550).

###### Bazaikha burial ground, unnumbered burial (individual ID I11104, female)

The Bazaikha burial ground, situated in the "Bor" area on the right bank of the Yenisei River near the mouth of the Bazaikha River, is currently located within the city limits of Krasnoyarsk. It was initially discovered by Ivan Savenkov in 1883. During the excavations carried out from 1883 to 1885, Savenkov revealed several burials, tentatively attributed to the Neolithic period, with one burial containing a celt axe, suggesting a Late Bronze Age date. In 1920, Sergey Sergeev excavated another burial, which is currently under study by Tatiana Savenkova, Nikolay Makarov, and Aleksandr Vdovin using materials from the museum collection.

Based on the available data, the Bazaikha burial ground encompassed at least seven burials, with six excavated by Ivan Savenkov and one by Sergey Sergeev.

One of the burials in Bazaikha contained the skeletal remains of a woman (individual ID I11104) in a supine position with her head oriented to the west. Grave goods discovered in the burial included flat, rounded mother-of-pearl beads, pendants crafted from animal teeth, and boar tusk plates (Vdovin and Makarov, 2016; Savenkova and Makarov, 2018; Alekseev, 1961; Debets, 1948; Dus, 1923). Radiocarbon dating places the skeleton within the 95% confidence interval of 5471–5231 calBCE (6385±25 BP, PSUAMS-7674).

#### Yenisei Forest-Steppe Eneolithic/EBA (Russia_UpperYenisei_EBA)

##### Dolgoye Ozero burial ground

The Dolgoe Ozero (Dolgoe Lake) burial ground is situated in the vicinity of Kansk, near a dune in the northwestern section of the quarry adjacent to the Dolgoe Lake. It was initially discovered by Gleb Maksimenkov in 1958 during a reconnaissance survey.

At a depth of 70 cm below the soil surface, a human humerus protruded from the ground on the cliff face. Below it, within the screened area, a skull was found, accompanied by a puncture mark from an elk's slate bone. Located 50 cm to the west were two lower jaws, upper limb bones, and fragments of skulls. Further clearing of the quarry cliff at a depth of 55-60 cm revealed three damaged grave pits, consisting of two single burials and one double burial.


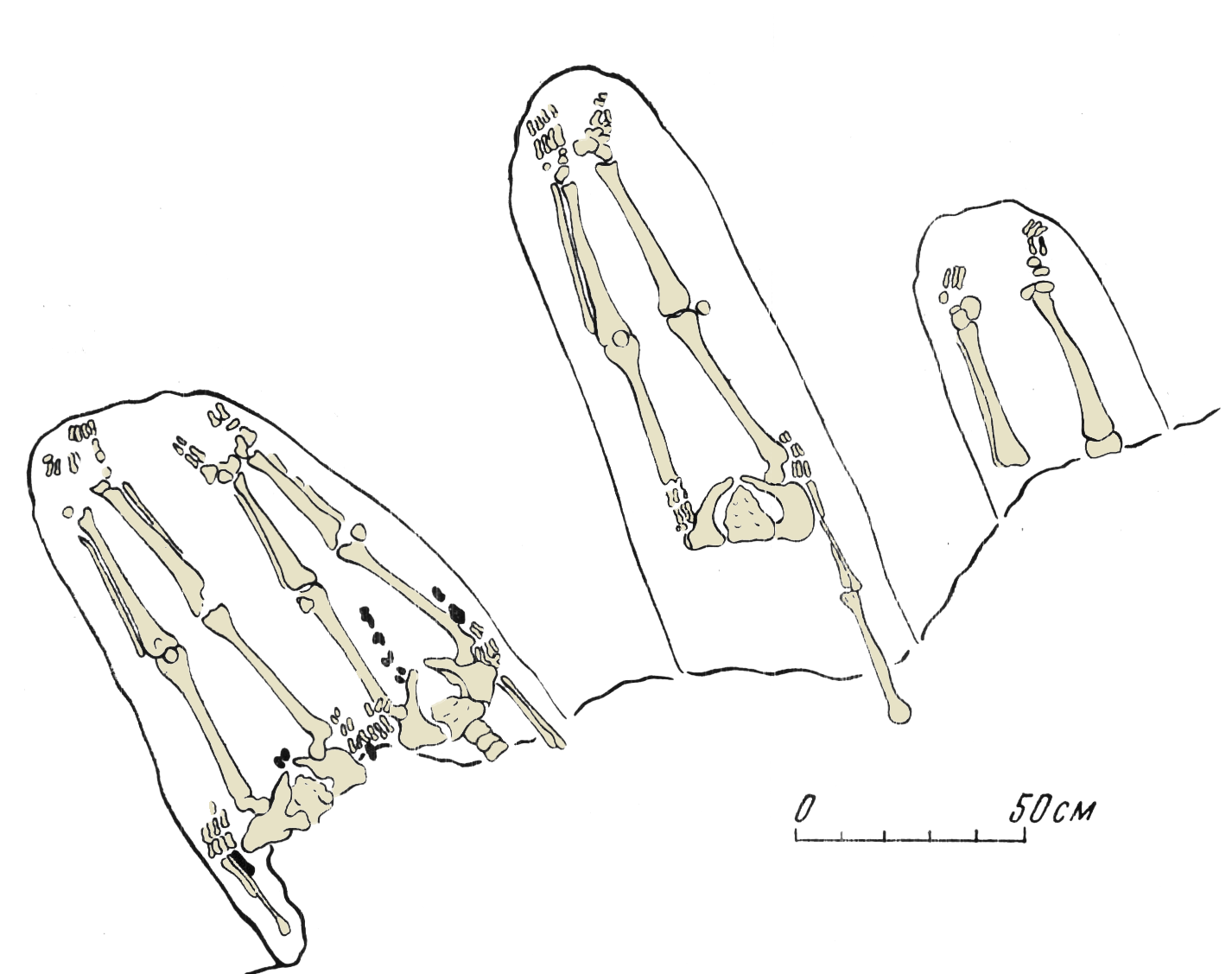


***Figure S12.*** *Partially preserved burials at the Dolgoye Ozero site (by Maksimenkov, 1964, modified).*

###### Unidentified burial (individual ID I1522, male)

The preserved parts of the grave pits measured up to 140x50 cm. Within them, four disarticulated skeletons were discovered, positioned supine with their heads oriented towards the southeast and their feet perpendicular to the oxbow lake channel, with their heads pointing northward. Grave goods recovered from these burials included three perforators crafted from elk's slate bone, pendants made from deer teeth, flint flakes, and a fragment of ocher. The skulls were found outside burials. M.M. Gerasimova conducted an examination of the skulls, revealing the following results: skull 1 belonged to a woman aged 25-35 years; skull 2 belonged to a male aged 20-25 years; skull 3 belonged to a female aged 30-35 years; and skull 4 belonged to a male aged 40-45 years (Maksimenkov, 1964; Gerasimova, 1964; Timoschenko, 2013).

The scarcity of the records makes it infeasible to determine which of the skulls and burials the individual ID I1522 pertains to. The burial is radiocarbon-dated to 2879–2586 calBCE (4150±35 BP, Poz-82193).

### The Upper Ob and Kuznetsk Depression

In 2019, a project for the preparation and publication of the "History of Altai" was implemented, in which one of the volumes reflected the results of the study of the prehistoric epoch and presented materials from archaeological sites attributed to the Neolithic period within four stages of its development (early, developed, late, and final). However, no emphasis was placed on their correlation with previously identified cultures (History of Altai, 2019, pp. 52–95). This circumstance is related to the fact that none of the previously presented concepts can be accepted as a comprehensive model for summarizing the accumulated information. There are more than enough reasons for such a situation. For example, all culture names are given based on regional designations, which do not correspond to the archaeological principles of studying sources and only demonstrate a geographical factor, hindering a genuine understanding of the specific characteristics of the accumulated materials (as a result, there are currently two chronologically distinct Upper Ob cultures in West Siberian archaeology). However, the main problem lies in the limited extent of interdisciplinary research on the still relatively small array of available data, which were obtained from dispersedly identified sites of different categories (burial grounds, settlements) and chance finds. For instance, only in the past decade have AMS dating methods begun to accumulate, allowing for a more precise determination of the chronological range of specific archaeological objects. Other important findings, obtained through the use of high-tech equipment, including paleogenetic analyses, which are only partially reflected in this article, will become significant as well. The correlation between the materials from burial grounds and settlement complexes will be of particular importance. Given the briefly mentioned information, substantial prospects for further scientific research work are evident.

An overview of the process of studying Neolithic sites and the identification of cultures in the territory of the southern part of West Siberia and Altai is reflected in the recently published volume "History of Siberia," which examines two regions: Upper Ob and Altai-Sayan Plateau (History of Siberia, 2022, pp. 216–218). The beginning of a systematic understanding can be attributed to M.N. Komarova, who in 1956 identified the Kiprino and Irba stages in the development of the Neolithic in the forest-steppe region of Upper Ob. The first generalization was made by V.I. Matyushenko, who in 1969 distinguished and characterized the Upper Ob Neolithic culture. The position of M.A. Anikovich is noted, who in the same year and on the same territory "…defined a group of sites associated with the Baikal Neolithic" (History of Siberia, 2022, p. 217). In the 1970s, V.I. Molodin refined the existing periodization, identifying the Zavyalovo and Kiprino stages in the development of the Upper Ob culture and pointing out an intercultural group of sites. In 1978, Aleksey Okladnikov and V.I. Molodin identified the Kuznetsk-Altai culture of the Neolithic period based on materials from individual burial grounds and the study of inscriptions. This perspective was critiqued in the second half of the 1980s by Yu.F. Kiryushin, who attributed some sites to his previously identified Bolshoy Mys culture of the Eneolithic period, primarily based on the study of the ceramic complex.

By the end of the 20th century, the number of archaeological materials attributed to the Neolithic period significantly increased. This was particularly true for the steppe zone of the left bank of Upper Ob, where more than 150 settlement sites with characteristic finds and individual burials are known. This allowed A.L. Kungurov, A.V. Onnikov, and A.A. Tishkin to identify the Rubtsovsk Neolithic culture based on the eponymous site, which yielded a significant volume of artifacts, the study of which continues to the present. The rationale for this culture was reflected in A.V. Schmidt's dissertation. The Neolithic sites of the forest-steppe zone on the right bank of Upper Ob demonstrate their own specific features, and the main burial grounds (Firsovo-11 and 14, Tuzovskie Bugry, Chumysh-Perekat, etc.) are considered in this article. Importantly, they show similarities with the left bank sites. This circumstance allows us to consider them all as part of a single cultural and historical entity, within which individual cultures with their distinctive stages should be considered.

Planned and multi-year excavations in the territory of the Mountainous Altai in its northern part have allowed for the study of well-stratified settlement complexes and the identification of the early, developed, late, and final stages of the Neolithic period. Within the framework of the late stage, Yu.F. Kiryushin and K.Yu. Kiryushin proposed the differentiation of the Middle Katun culture, although the researched settlements and sites are actually located in the lower reaches of the Katun River.

Another historiographical aspect highlighted is the Ural-West Siberian Neolithic community (History of Siberia, 2022, p. 218), which has not seen further development.

In the mentioned volume "History of Siberia," characteristics of the Upper Ob Neolithic culture, Kuznetsk-Altai culture, and Middle Katun culture are presented (History of Siberia, 2022, pp. 260–268, figures 155–159). These cultures possess their own local peculiarities, but they require more detailed examination at a contemporary level, as previous hypotheses have already run their course. One example of this is the rejection of dating the petroglyphs to the Neolithic period, attributed to the Kuznetsk-Altai culture. It is necessary to abandon all previously proposed cultural designations that do not adhere to the methodological principles of archaeological research and have exhausted their potential. This is evidenced, in particular, by new insights into ethnocultural processes in the Baraba and Middle Irtysh regions, where several archaeological cultures of the Neolithic period have been identified (History of Siberia, 2022, pp. 248–259).

This review and the new scientific information, including paleogenetic data, indicate the need for the study of Neolithic materials in the south of West Siberia and Altai at a contemporary research level, with a purposeful search for other archaeological sites and excavations of foundational monuments.

#### Kuznetsk-Altai Neolithic/Upper Ob Neolithic/Bolshoy Mys Eneolithic sites (Russia_KuznetskAltai)

##### Razdum’ye-1 site

Razdum'ye-1 is a Medieval hillfort located on an elevated promontory on the right bank of the Ob River. The site was extensively investigated and excavated by Alexei Umansky between 1960 and 1966. The archaeological exploration revealed a complex and multi-phased assemblage spanning from the Neolithic to the late Middle Ages. In 1965, two Neolithic/Eneolithic burials were excavated at the site (Umanskiy, 1987).

###### Early Neolithic burial 1(6) (individual ID I12695, male)

**Burial 6** was designated firstly as **burial 1**, and this code is still in use in the Tomsk anthropological collection, but the burial is recorded as **grave 6** in a publication by Umanskiy in 1987) was excavated in 1965. The grave pit had an oval shape with dimensions of approximately 1.2x0.75 meters. Its depth reached 1.3 meters from the present-day surface. The southwestern part of the grave had collapsed into the river. The deceased individual, identified as a male aged 50-60 years, was found in a supine position with the head oriented to the north. The arms were extended, while the leg bones were missing. The bones exhibited slight traces of ochre staining. Small fragments of charcoal and sheep's astragalus were discovered in the grave fill. Traces of wood ashes indicated the presence of grave siding and a vertical post positioned behind the skull of the deceased. No grave goods were present in the burial (Umanskiy 1987). The skeleton is dated to 6571–6424 calBCE (7635±35 BP, PSUAMS-9117).

##### Zarechnoye-1 site

Zarechnoye-1 consists of a group of burial mounds excavated by Viktor Zakh in 1980 and 1983. The site is located on a small promontory on the high left terrace of the Inya River. The burial ground contains approximately 80 mounds, grouped into two clusters. Excavations covered an area of approximately 900 square meters.

The excavations at Zarechnoye-1 revealed burials belonging to the Irmen culture beneath the mounds. Additionally, an Andronovo flat burial ground, a Samus’ settlement complex, and five burials dating back to the Neolithic and Early Bronze Ages were discovered. Two Neolithic burials were found beneath mound 2, which was constructed during the Irmen period (Zakh, 1985).

###### Mound 2, Early Neolithic burial 3 (individual ID I2138, female)

The burial was partially overlapped by a later grave attributed to the Irmen culture. The boundaries of the grave pit were not traced. The skeleton was placed in a vertical, standing position, fronting east. Its upper part was damaged by the later grave. The skull was located 0.4 m to the southeast from the remaining bones of the skeleton and was sprinkled with ocher. The buried person was a woman about 25 years old. The right side of the chest was abundantly covered with ocher. During excavation, in the infill of the grave on the left side of the skeleton, two microblades and a stone axe made from a slate flake were found. Near the right pelvic bone, a fragment of a bird bone polished on both sides and a bone tool made from split bone were recorded (Zakh, 1985). The burial has been dated to 7035-6651 calBCE (7920±50 BP, Poz-82211).

###### Mound 2, Early Neolithic burial 4 (individual ID I2139, female)

**Burial 4** of **mound 2** was located at a distance of 1 to 3 meters from **burial 3**. The grave pit was not clearly identified, as it was partially covered by an Irmen culture burial. The buried individual, a male estimated to be 25-30 years old, was found in a supine position with the head oriented to the north, parallel to the edge of the terrace. A piece of animal bone was discovered on the right side of the chest (Zakh, 1985). The burial has been dated to 7035–6651 calBCE (7920±45 BP, OxA-33165).


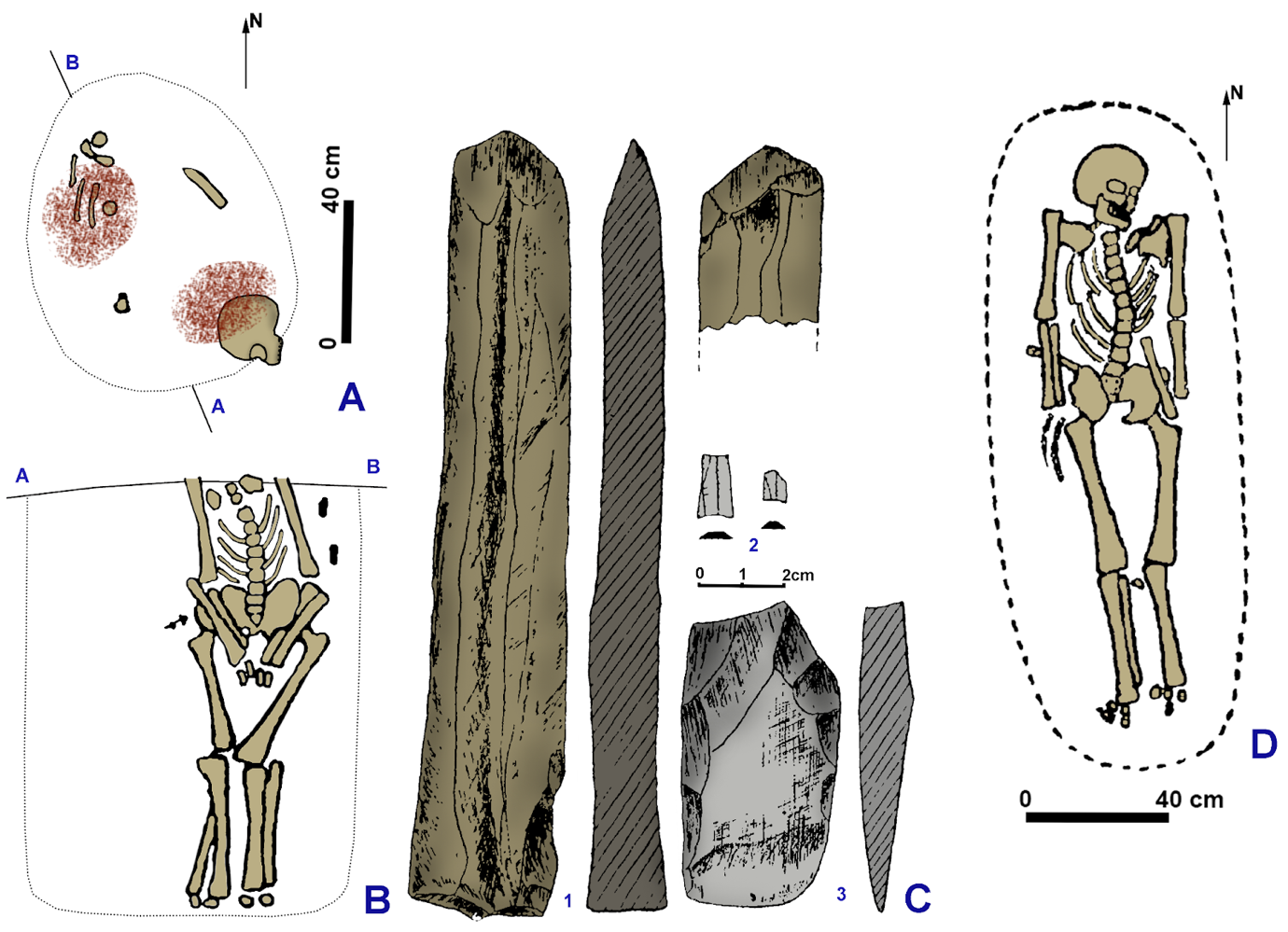


***Figure S13.*** *Early Neolithic burials at the Zarechnoye-1 site.* ***A****: mound 2, burial 3, plan;* ***B****: mound 2, burial 4, section;* ***C****: artifacts from burial 3,* ***1*** *- bone tool,* ***2*** *- blades,* ***3*** *- stone axe;* ***D****: mound 2, burial 4, plan (by Zakh, 1985, modified).*

##### Firsovo-11 burial ground

The Firsovo-11 cemetery is situated on the right bank of the River Ob, opposite the city of Barnaul. AT this location, the right bank of the Ob features a wide, swampy floodplain measuring up to 7 km, adorned with numerous oxbow lakes and remnants of the original coastline in the form of crests and mounds. The surrounding vegetation consists of meadows with thickets of shrubby willows, aspens, and birches. During periods of flooding, the water level in the Ob rises by 1.5–3 m, submerging the floodplain almost entirely and approaching the bedrock bank upon which the monument is situated.

The cemetery was initially investigated in 1977 by A.L. Kungurov and V.B. Borodaev, and later excavated by Ya.V. Frolov in 1993–1994. The monument spans an area of 0.3 hectares. Eight burials were discovered at the site, including five individual graves, two paired graves, and one collective burial. The burials are arranged in two rows, oriented along a southeast-northwest axis. The deceased individuals within the graves were found to be oriented with their heads pointing northward or northeastward. Grave depths varied from 0.4 to 1.7 m.

Thirteen AMS dates were obtained, ten for human bones and teeth, and three for animal remains. The results indicate the presence of a reservoir effect, with an average offset of approximately 700 years. Some of the dates have been published (Kiryushin et al. 2021a, Kiryushin et al. 2021b). The age of the Neolithic burials corresponds to the middle of the 5th millennium BC. The "Neolithic core” of the site comprises burials 9, 14, 15, 16, 17, 41, and 42, with burial 18 belonging to an earlier period.

Noteworthy artifacts found at the site include ornaments crafted from animal teeth (bear, wolf, horse, etc.). These tooth ornaments serve as chronological indicators, offering the potential for specialized dating methods to investigate the reservoir effect further (Kiryushin et al., 2021a, 2021b).

The Neolithic burials at the Firsovo-11 site form a cohesive cultural and chronological group together with graves 1 and 13 of the Itkul (Bolshoi Mys) cemetery. With time, this group may expand to include burials 2, 4, 6, 10, and 14 of the Itkul (Bolshoi Mys) cemetery.

The designation of the Neolithic burials at the Firsovo-11 site as belonging to the Bolshoi Mys culture of the Eneolithic period (Kiryushin, 2002) requires thorough reassessment. The other names used to label the Neolithic cultures in the region include Upper Ob and Kuznetsk-Altai Neolithic cultures.


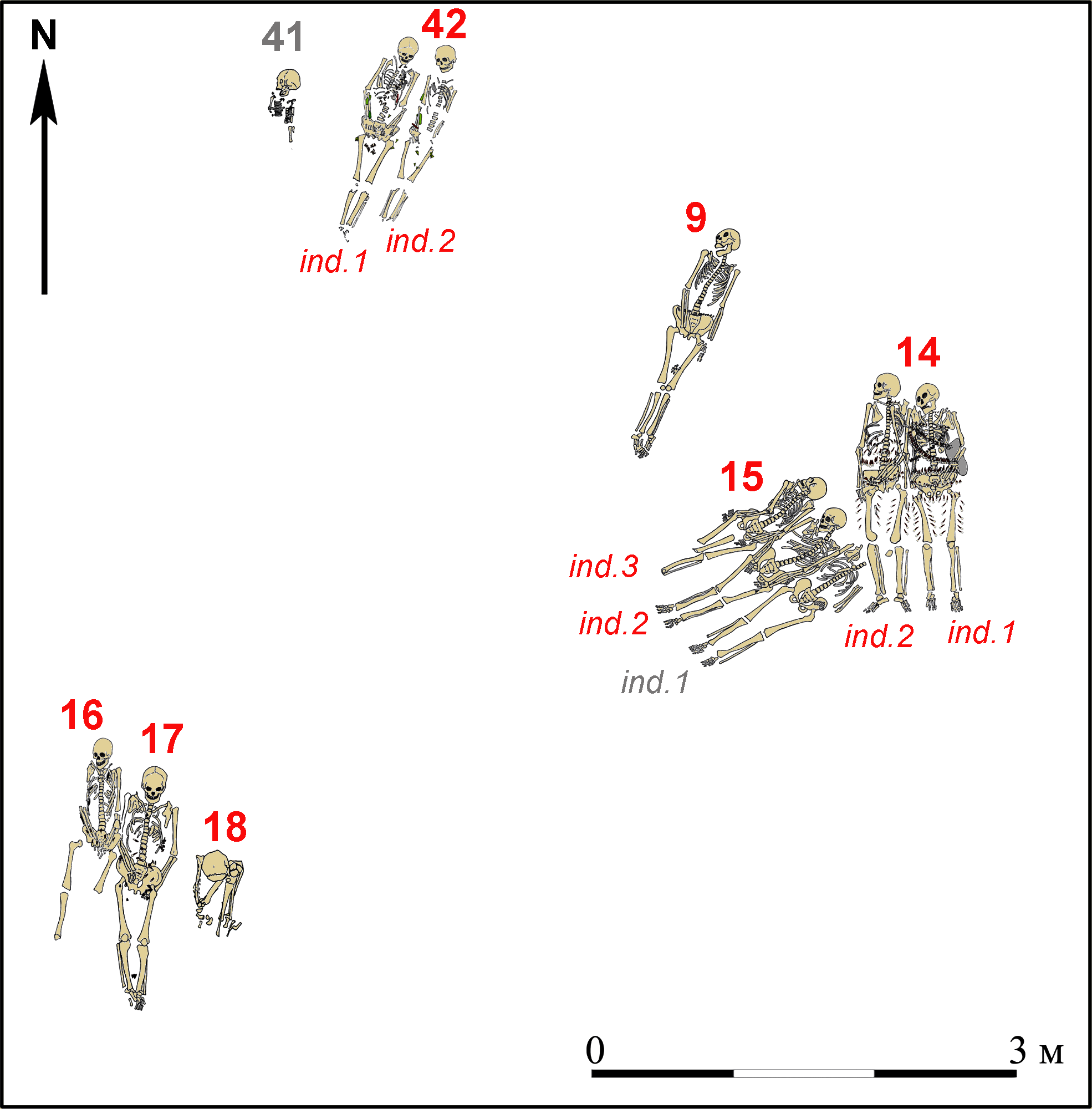


***Figure S14.*** *Neolithic burials at Firsovo-11. Involved individuals are labeled in red (image by Kirill Kiryushin).*

###### Burial 9 (individual ID I0996, male)

**Burial 9** was unearthed in 1993 during an excavation of the lower section of an Early Iron Age burial mound. It was located 2 meters southwest of **burial 42** and 1-1.5 meters northwest of **burials 14** and **15**, situated in the central part of the "northeastern row." The condition of the grave stain was indistinct and difficult to decipher. The grave had a depth of 1.7 meters from the contemporary ground surface and exhibited an elongated shape, aligned along the south-southwest-north-northeast axis. The deceased individual, identified as a male aged 40-50 years, was found in a supine position with the head oriented towards the north-northeast.

The burial was accompanied by grave goods, which included nine triangular stone arrowheads with either a straight or notched base. Additionally, there were 22 bone patches discovered: 21 of them exhibited elongated outlines with subrectangular or oval shapes and a hole in the upper part, while one patch had a round shape with a hole in the center. It should be noted that the skull showed signs of deformation due to the pressure exerted by the overlying soil (Kiryushin et al., 2021a, 2021b).

A combination of two radiocarbon dates from the burial shows interval 5479–5377 calBCE (6469±18 BP) [R_Combine: (6500±24 BP, MAMS-48706); (6435±25 BP, PSUAMS-4214)].

###### Burial 14, individuals 1 (individual ID I13096, male) and 2 (I0997, male)

**Burial 14** was uncovered in 1993, positioned 1.5 meters southeast of **burial 9** and partially intersecting with **burial 15** in the eastern section of the "northeastern" row. The state of the grave stain was indecipherable and offered no readable information. Within the burial, located at a depth of 0.8 meters from the current ground surface, the remains of two individuals were discovered, interred "shoulder to shoulder."

***Individual 1*** (individual ID I13096) (a male, aged 20-30 years) was found in a supine position with the head oriented towards the north. The skull exhibited deformation resulting from the pressure exerted by the soil. An analysis  of a human ulna from ***skeleton 1*** provided a date of 6330–5913 calBCE (7222±82 BP, GV-02887).


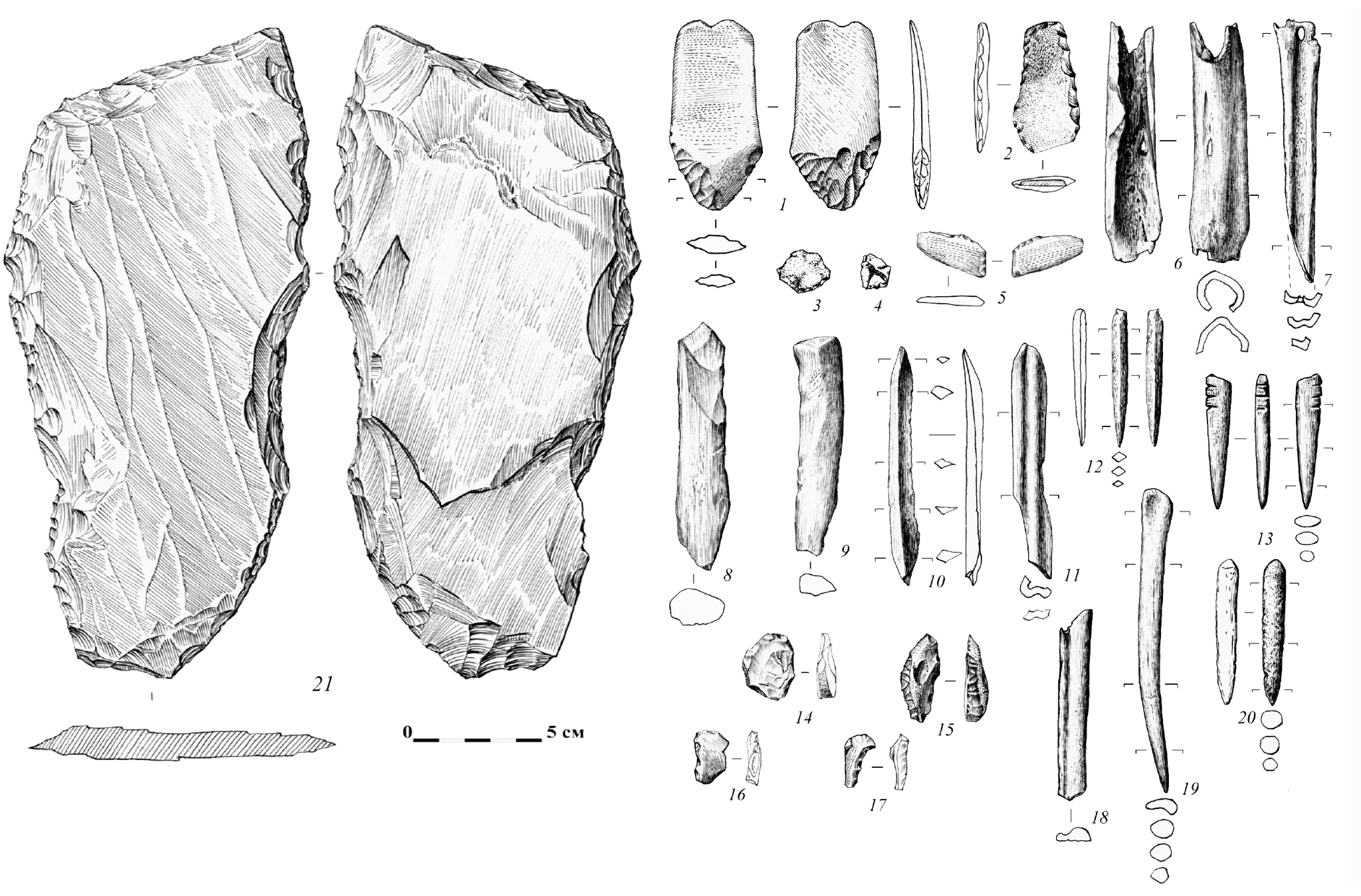


***Figure S15.*** *Grave goods from burial 14 at Firsovo-11.* ***1-13*** *- skeleton 1; 1****4-20*** *- skeleton 2;* ***21*** *- burial 14.* ***1*** *- polished knife;* ***2*** *- polished axe;* ***3, 4*** *- retouched flakes;* ***5*** *- fragment of a polished knife;* ***6*** *- fragment of a tubular bone;* ***7-9, 11*** *- tool blanks;* ***10, 12, 13, 19, 20*** *- blades;* ***14, 15*** *- scrapers;* ***16*** *- retouched flake;* ***17*** *- serrated and notched tool;* ***18*** *- fragment of a bone tool (image by Kirill Kiryushin).*

***Individual 2*** (individual ID I0997) (presumably a young female, but genetically identified as a male, aged 18-20 years) was also lying extended on her back with the head facing north. Similar to the previous individual, the skull displayed deformation due to soil pressure.

**Burial 14** represents the most affluent interment within this necropolis. The accompanying funerary inventory comprises a multitude of bone and stone artifacts. Notably, the skeletons were adorned with necklaces composed of animal teeth that had been drilled at the roots and organized in clusters (Kiryushin et al., 2021a; 2021b).

Radiocarbon dating was conducted on a fragment of an elk tooth from ***skeleton 1's*** necklace, resulting in a date of 4708–4547calBCE (5778±28 BP, GV-03572). A date from human bones of skeleton 1 shows much earlier chronology, 5617–5479 calBCE (6595±30 BP, PSUAMS-4119), most likely due to freshwater reservoir effect. Due to the observed δ13C/δ13N ratio, we expect freshwater reservoir offset in the date obtained from human bones and prefer to use the date from the elk tooth.

######
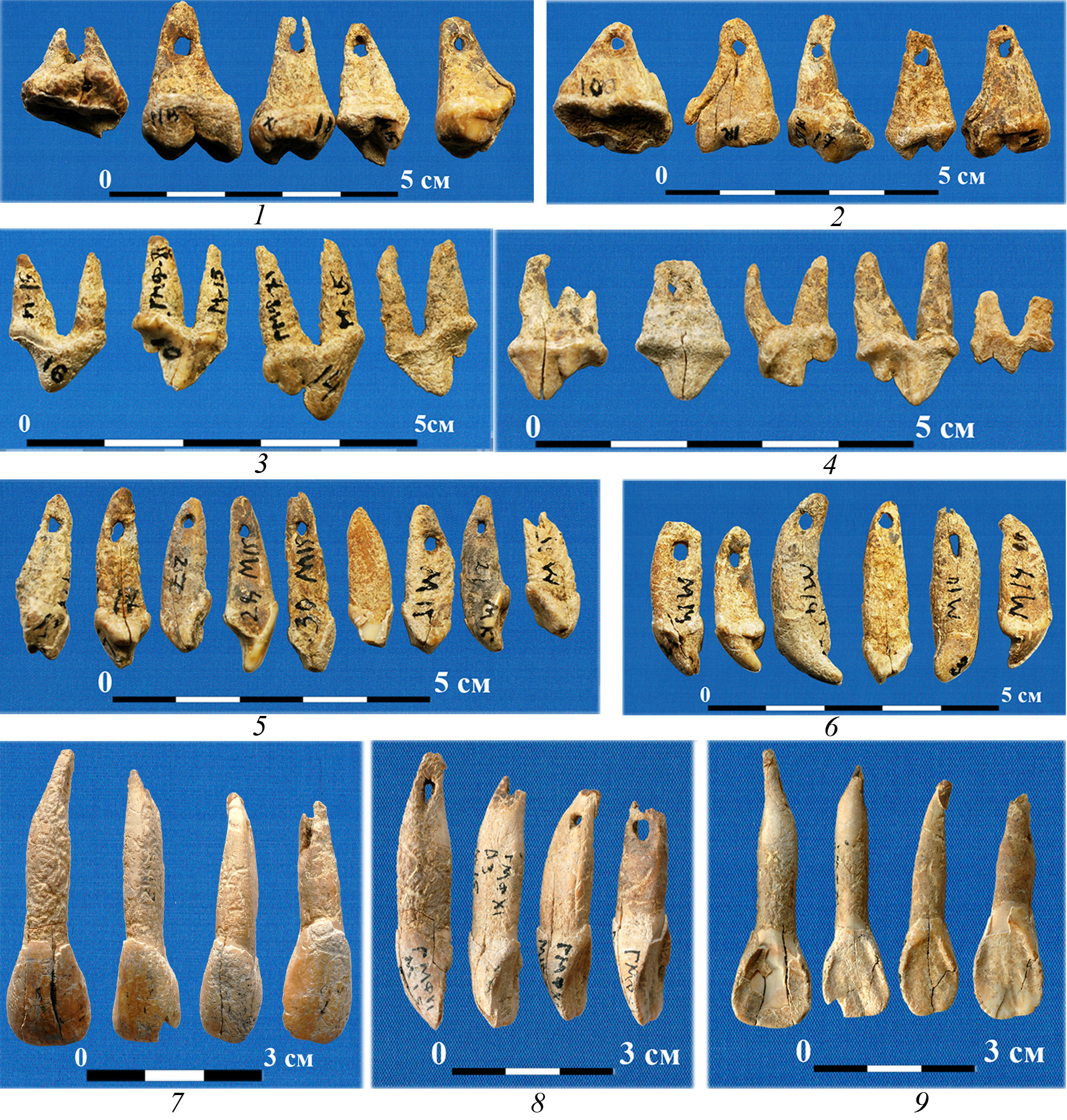


***Figure S16.*** *Ornaments and costume details from burial 14 at Firsovo-11 (photo by Kirill Kiryushin).*

###### Burial 15, individuals 2 (individual ID I11744, male) and 3 (I10963, male)

**Burial 15**, situated at the southeastern end of the "northeastern" row, has experienced partial disturbance due to burial 14. The grave pit was not recorded. This burial represents a collective interment of three elderly males, positioned in an extended supine position, placed adjacent to each other ("shoulder to shoulder"), with their heads oriented toward the east-northeast. The bottom of the grave was located at a depth of 0.87 meters from the contemporary surface.

***Individual 2*** (individual ID I11744) (a male, aged 45-55 years) was laid out fully extended on his back, with his head facing the east-northeast. The skull has suffered deformation due to the weight of the soil.

***Individual 3*** (individual ID I10963) (an elderly male, aged 55-65 years) was also laid out fully extended on his back, with his head facing the east-northeast. The skull shows deformation caused by the pressure of the surrounding earth. The bone condition is deteriorated.

A triangular stone arrowhead with a notched base was discovered on the left femur of ***skeleton 2*** (Kiryushin et al., 2021a; 2021b) (Kiryushin et al., 2021a, 2021b).

***Skeleton 2*** has been dated to 5711–5558 calBCE (6705±30 BP, PSUAMS-8933), freshwater reservoir effect is expected. In another paper, ***skeleton 1*** was dated to 5702–5485 calBCE (6684±39 BP, UBA-22954) and ***skeleton 3*** to 5734–5521 calBCE (6723±68 BP, GV-02888) (Kiryushin et al., 2021c). The combination of dates shows the interval of 5665–5561 calBCE (6700±23 BP) [R_Combine: ind. 1 (6684±39 BP, UBA-22954); ind. 2 (6705±30 BP, PSUAMS-8933); ind. 3 (6723±68 BP, GV-02888).

######
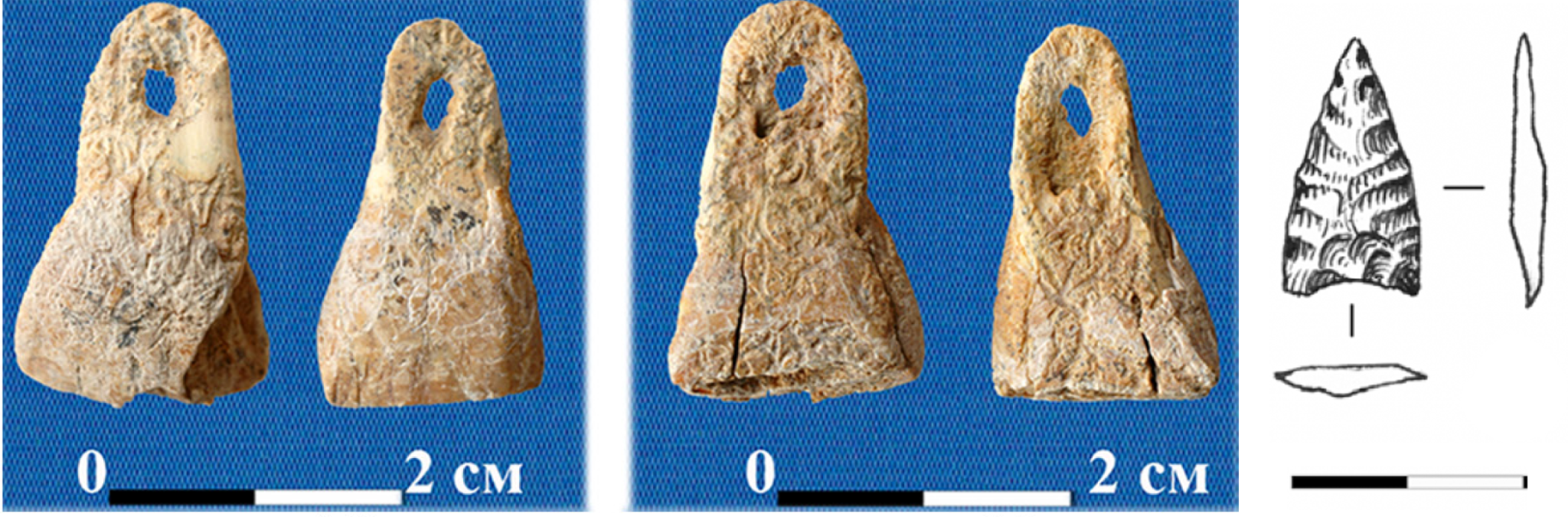


***Figure S17.*** *Ornaments from a horse chisel burial 17 and an arrowhead from burial 15 at Firsovo-11 (image by Kirill Kiryushin).*

###### Burial 16 (individual ID I10962, male)

**Burial 16** is situated at the far northwestern end of the second row. The actual grave pit was not identified or delineated. During the excavation of the area, the bones were discovered at a depth of 0.4 meters from the contemporary surface. The deceased individual was interred at the boundary between the humus layer and the underlying subsoil sand.

The deceased, a male estimated to be 30-40 years old, was laid out in a fully extended supine position, with his head facing northward, slightly inclined towards the east. The skull exhibits deformation due to significant deterioration and preservation issues. The bone condition is also poor.

Accompanying the burial are several grave goods: three arrowheads with leaf-like shapes (resembling double-edged points), a bone point, a stone knife resting on a silicified slate slab, and eight pendant teeth.

Based on an analysis of a fragment of human bone, a date was determined: 5478–5372 calBCE (6459±28 BP, GV-03573).

###### Burial 17 (individual ID I11741, male)

**Burial 17** was positioned within the second row, situated between **burials 16** and **18**. **Burial 16** partially overlaid **burial 17**. The precise outline of the grave pit has not been determined. The excavation reached the bottom of the burial at a depth of 75 centimeters from the contemporary surface. The skeletal remains of a woman, estimated to be between 45 and 55 years old, were discovered within the burial. The deceased was laid out in a fully extended supine position, with her head facing northward, slightly deviating towards the east. The skull exhibits deformation due to poor preservation, and the bone material is in a deteriorated condition.

Accompanying grave goods include 13 stone arrowheads with either straight or notched bases, as well as 34 pendants made from animal teeth, each featuring a drilled hole in the root.

A date of 5478–7361 calBCE (6448±28 BP, GV-03575) was obtained through analysis of a fragment of human bone. Additionally, a date of 4782–4553 calBCE (5818±28 BP, GV-03574) was obtained from a fragment of a bear's tooth.

###### Burial 18 (individual ID I11742, male)

**Burial 18** occupies the extreme southeast position within the second row, although the precise outline of the grave pit has not been identified. The interred male individual, estimated to be between 35 and 45 years old, was placed in a seated position within the grave. The skull was discovered at a depth of 0.5 meters from the modern surface and exhibits deformation resulting from the pressure of the surrounding soil. The excavation reached a depth of 1 meter from the modern surface, revealing a burial richly adorned with ocher. The sole funerary item present in the burial is a knife-shaped piece.

The cranial dimensions of the interred individual, differing from those of others buried at the site, further underscore the chronological and ritual distinctiveness of this burial.

Based on fragments of the human radius bone, a date of 8561–8016 calBCE (9106±80 BP, GV-02889) was obtained.

As a working hypothesis, it is proposed that the very old date for burial 18 at Firsovo-11 is not an artefact, suggesting that this burial indeed belongs to the final Mesolithic or early Neolithic period. Burial 18 at Firsovo-11 represents one of the few early Holocene burials where the deceased was interred in a seated position (Kiryushin et al., 2021a, 2021b).

###### Burial 42, individuals 1 (individual ID I12225, male) and 2 (I12224, male)

**Burial 42** was discovered in the northern part of the first row, located 2 meters northwest of **burial 9** and 0.5 meters east of **burial 41**. The exact boundaries of the grave pit have not been determined. The depth of the grave is 0.7 meters from the modern surface. Within the burial, the remains of two individuals were found.

***Individual 1*** (individual ID I12225), identified as a man between 45 and 55 years old, was laid out in a supine position with his head oriented to the north-northeast. The skull exhibits deformation caused by natural processes and the pressure exerted by the surrounding soil. The bone preservation is in poor condition. The burial assemblage includes stone and bone artifacts, as well as clothing adornments made from animal teeth that were drilled into the root (Kiryushin et al., 2021a, 2021b). A date of 5623–5363 calBCE (6534±72 BP, GV-02890) was obtained from a fragment of human bone.

***Individual 2*** (individual ID I12224), antroopologically identified as female aged 40 to 50 years, was also laid out in a supine position with her head oriented to the north-northeast. Similar to the previous individual, the skull shows signs of deformation resulting from natural processes and soil pressure. The bone preservation is also in poor condition. The accompanying grave goods consist of eleven stone arrowheads with a notched base, two small polished stone axes, a split beaver chisel, and a fragment of a bone artifact (Kiryushin et al., 2021a, 2021b). A date of 5609–2390 calBCE (6404±28 BP, GV-03576) was obtained from a fragment of human bone. Additionally, a date of 4685–4501 calBCE (5745±28 BP (GV-03577) was obtained from a fragment of a bone artifact.

######
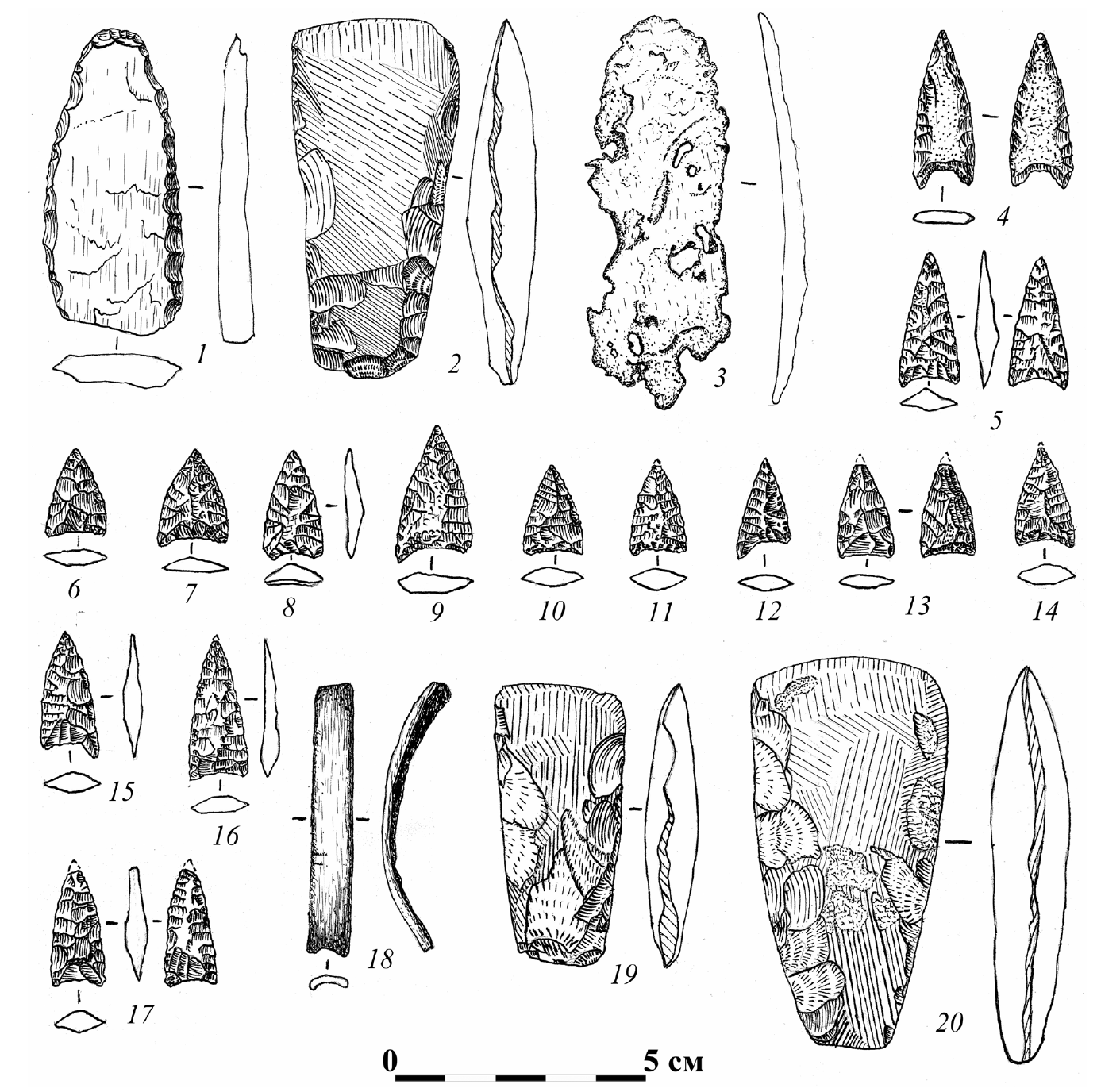


***Figure S18.*** *Grave goods from burial 42 at Firsovo-11.* ***1-9*** *- skeleton 1;* ***10-20*** *- skeleton 2.* ***1*** *- knife on a silicified shale;* ***2, 19, 20*** *- hatchets;* ***3*** *- bone slab;* ***4-17*** *- an arrowhead;* ***18*** *- a bracelet made of beaver incisors (image by Kirill Kiryushin).*

##### Chumysh-Perekat-1 burial site

The Chumysh-Perekat burial ground, discovered in 2012 by Sergey Grushin, was extensively studied between 2014 and 2019 by a joint expedition from Kemerovo State University and Altai State University, led by Aleksey Fribus and Sergey Grushin.

Chumysh-Perekat is situated on a prominent cape along the right bank of the Chumysh River, a right tributary of the Ob River. This area in the Western Salair region marks the boundary between the forest-steppe and mountain taiga landscape zones. An area of 1,718 square meters was investigated within the monument, revealing a total of 36 burials, of which 12 belong to the Neolithic period.

The Neolithic burials form distinct rows extending southeast from the western edge of the cape into the terrace. The Neolithic graves were characterized by inhumation. The grave pits are shallow, and their size and shape are not clearly discernible. With the exception of one grave, all burials are individual in nature. Burial 8 represents a double burial. The interred individuals were laid out in a supine position with their knees pressed against each other, and their heads oriented towards the northeast. The grave goods associated with these burials are not numerous and consist of stone and bone tools, ornamental items crafted from animal teeth, and a bone wand bearing an image of an elk's head. Notably, the artifact assemblage includes stone and bone implements, jewelry, and artistic objects, such as a fragment of a stone figurine depicting a fish of the Baikal type.

Four radiocarbon dates were obtained from human bones at the site, at laboratories in St. Petersburg and Tomsk.

Based on the available radiocarbon dates, the Chumysh-erekat monument can be attributed to the 6th – the first half of the 5th millennium BC. The burial ground exhibits significant similarities to the Firsovo-11 site in terms of burial practices and the complex of clothing items. It also shares similarities with other sites such as Solontsy-5, Ust-Isha, and Big Cape, as indicated by the funeral rite and the assortment of cultural artifacts (Kungurova, 2000; 2005). Currently, it is not possible to discern distinct stages in the functioning of the cemetery (Fribius and Grushin, 2017a, 2017 b, 2020).

###### Burial 6 (individual ID I11745, male)

The exact shape of the grave pit, as in other cases, could not be determined. The dimensions are estimated as follows: length 196 cm, width 78 cm, and depth approximately 35 cm from the modern surface. The bottom of the pit exhibited a slight depression from head to feet and had a southwest-northeast orientation. The burial pertains to an adult individual, whose skeleton was laid out elongated on the back, with arms alongside the body and legs slightly bent at the knees, and the head facing northeast. **Burial 6** was disturbed during the construction of **burial 7**. It is an individual burial. The only grave item found was a bone arrow or dart tip, hexagonal in shape and measuring 10.8 cm in length. Radiocarbon dating yielded an age of 5970–5414 calBCE (6739±130 BP, IMCES-14С1005). (Fribus, Grushin, 2020).

###### Burial 7 (individual ID I13097, female)

Based on the position of the preserved bones, it appears that **burial 7** partially intersected **burial 6**, resulting in damage to the skeleton of **grave 6**. The dimensions of the grave pit could only be approximated: length 103 cm, width 55 cm, and depth from the modern surface ranging from 45 to 55 cm. The orientation of the grave was northeast-southwest, and similar to **burial 6**, it exhibited a depression from head to feet. The bottom of **burial 7** was situated 20 cm below the level of the bottom of **burial 6**. This grave represents an individual burial of a child. Analysis of the in situ bones, including the skull, some thoracic bones, and leg bones, suggests that the interred individual was laid out elongated on their back, possibly with a slight deviation to the right side, and with the head pointing northeast. No grave goods were present. The estimated date of the human bones from the burial is 6742–6509 calBCE (7810±35 BP, PSUAMS-13976).

###### Burial 9 (individual ID I12626, male)

The shape of the grave pit could not be determined. The orientation was southwest-northeast, and the depth from the subsoil level was 22 cm. It was an individual burial of a man aged 45–55 years, who was laid stretched on his back, with the head pointing northeast. The skull exhibited a slight turn to the right. The legs were brought together at the knees, and the arms were positioned along the body. Many phalanges of the fingers and bones of the feet were missing.

###### Burial 12 (individual ID I13098, male)

The shape of the grave pit could not be determined. The orientation was southwest-northeast, and the depth from the subsoil level was 10 cm. The burial was individual and partially disturbed. The bones preserved in situ indicate that the buried man, approximately 20–25 years old, was laid stretched on his back, with the head pointing northeast. The skull and lower jaw were displaced towards the legs, and the right arm was slightly bent at the elbow, possibly with the hand placed on the pelvis. The bones of the left hand, pelvic and femur bones, and foot bones were missing. The bones on the left side of the thoracic region were displaced. Radiocarbon dating of human bones provided an age of 6058–5663 calBCE (6974±110 BP, IMCES-14C1003) (Fribus, Grushin, 2020). Alternatively, the human bones from the grave were dated to 6392–6095 calBCE (7400±35 BP, PSUAMS-9042). The combination of these two dates provides a date of 6059–5663 calBCE (6974±111 BP) [R_Combine: (6974±110 BP, IMCES-14C1003); (7400±35 BP, PSUAMS-9042)].

###### Burial 13 (individual ID I10961, female)

The exact shape of the grave pit could not be determined. It had a southwest-northeast orientation and a depth of 20 cm from the subsoil level. This grave represents an individual burial, where the deceased was laid stretched on their back, with the head pointing northeast. The skull exhibited a slight turn to the right, and the bones of the legs were tightly pressed together. The arms were positioned alongside the body. Most of the bones from the feet and hands were missing. Adjacent to the skeleton, at a distance of 20 cm on both the right and left sides, three vertically positioned wood pieces were discovered, possibly indicating elements of a tomb construction. The fragments measured 1-2 cm in diameter, with surviving lengths of approximately 10-12 cm. Grave goods accompanying the burial included a stone lanceolate biface knife, a flake, a bone dagger tip, a stone pendant resembling an elk's tooth with decorative patterns, a fish-shaped pendant made of mother-of-pearl, a beaver chisel, 33 pendants made from animal teeth (possibly dog or wolf) with holes in the root sections, and a bird's metacarpal bone serving as a buckle. Based on the burial materials, a reconstruction of the costume elements was undertaken (Fribus, Grushin, 2017).

The burial was radiocarbon-dated to 4991–4342 calBCE (5770±150 BP, Le-11465).


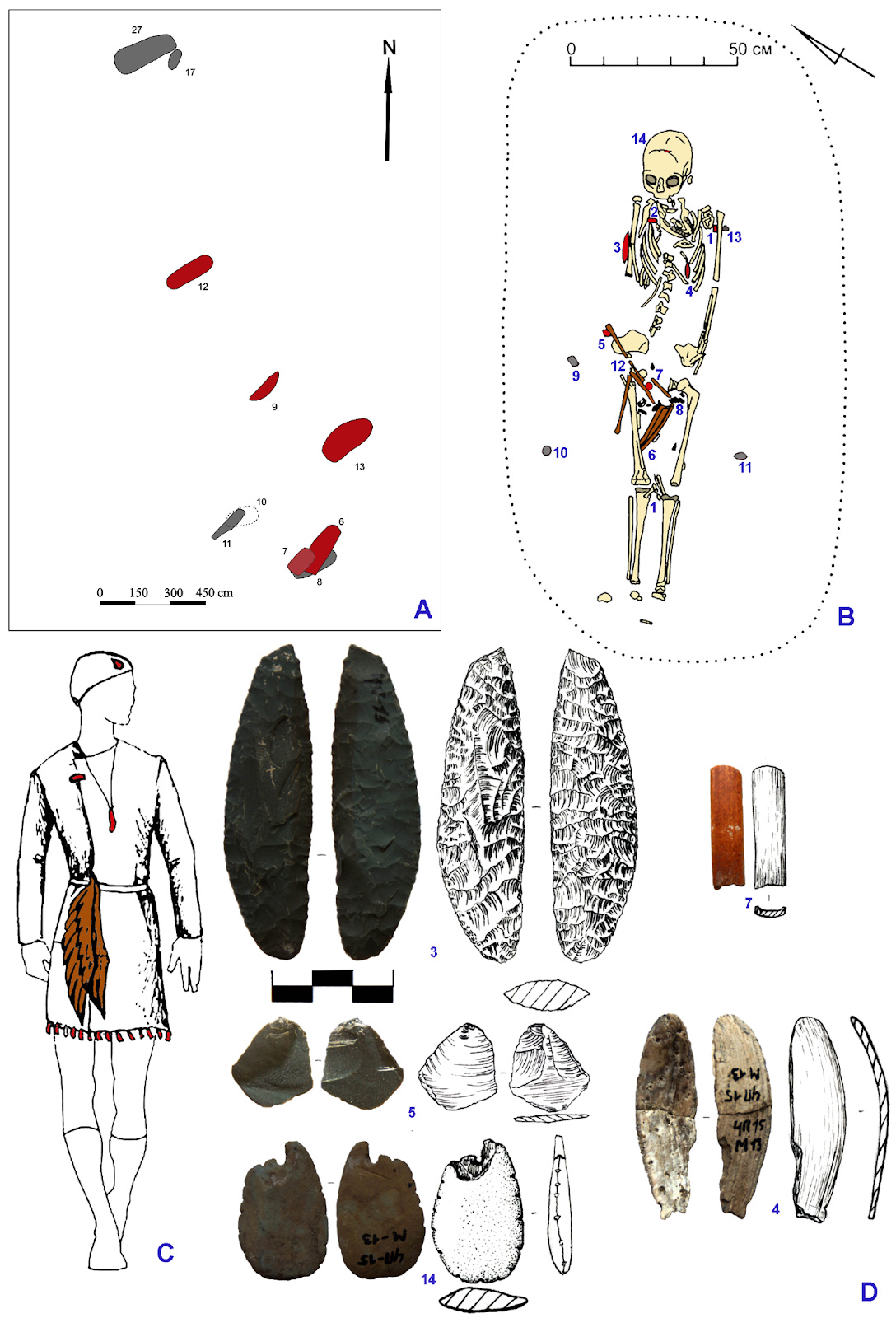


***Figure S19.*** *Chumysh-Perekat burial site.* ***A****: plan of the cemetery, sequenced burials are marked in red;* ***B****:  burial 13 at Chumysh-Perekat;* ***C****: reconstruction of the costume;* ***D****: artifacts from the grave.* ***C, D: 1*** *- animal tooth,* ***2*** *- beaver incisor,* ***3*** *- knife,* ***4*** *- fish-shaped pendant,* ***5*** *- flake,* ***6*** *- bone dagger tip,* ***12*** *- metacarpal bones of a bird;* ***7*** *- perforated bone,* ***8*** *- animal teeth,* ***9-11*** *- wood pieces,* ***13*** *- charcoal,* ***14*** *- stone pendant (image by Alexey Fribus and Sergey Grushin).*

##### Vas’kovo-4 site

The Vaskovo-4 burial site is situated on an elevated cape-like area near the village of Vas’kovo in the Promyshlennovsky district of the Kemerovo region. It is located at the confluence of the Inya River and its small right tributary, the Tykhta River. The arrangement of the burials within the site remains unpublished. The site was accidentally discovered in 1963 due to river bank erosion and subsequently investigated in 1964 by Yu.M. Borodkin. Unfortunately, the field documentation and a significant portion of the artifact collection from the site have been lost. Partial publications of the materials exist (Borodkin 1967, 1972, 1976), which are widely known and referenced in various comprehensive studies (Molodin 1977, 1992; Anikovich 1969; Bobrov 1988, etc.). The cultural attribution of the site remains controversial (Zah 2003).

Initially, the burial ground consisted of at least three burials, one of which was found in a deteriorated state. Sex and age characteristics of the individuals are not specified. It is likely that all burials were of adult individuals. The positioning of the corpses within the graves is not detailed in the publications. However, in all burials, the individuals were oriented with their heads to the east, perpendicular to the headwaters of the small tributary, and with their feet downstream in relation to the main body of water, the Inya River. Ocher was found covering the human remains in all burials (Marochkin 2014).

###### Neolithic burials 1 (individual ID I2074, male) and 3 (I2075, male)

According to the available publication (Borodkin 1972), no burials lacked grave goods. However, due to the fragmented nature of the available information, it is not possible to differentiate the burials based on the extent of their accompanying inventories. From the detailed description of **burial 1** (individual ID I2074), it can be inferred that the nature and quantity of grave goods varied significantly between burials.

The spatial arrangement of the grave goods within the burial can only be determined with certainty in the case of **burial 1**. In most instances, the objects were positioned in the pelvic area (5 objects), followed by the chest area (2). There was one recorded case of objects placed near the head and hips (Marochkin 2014).

**Burial 1** (individual ID I2074) is dated to 5610–5376 calBCE (6520±40 BP, Poz-83514), and **burial 3** (individual ID I2075) is dated to 5551–5369 calBCE (6500±40 BP, Poz-83403).


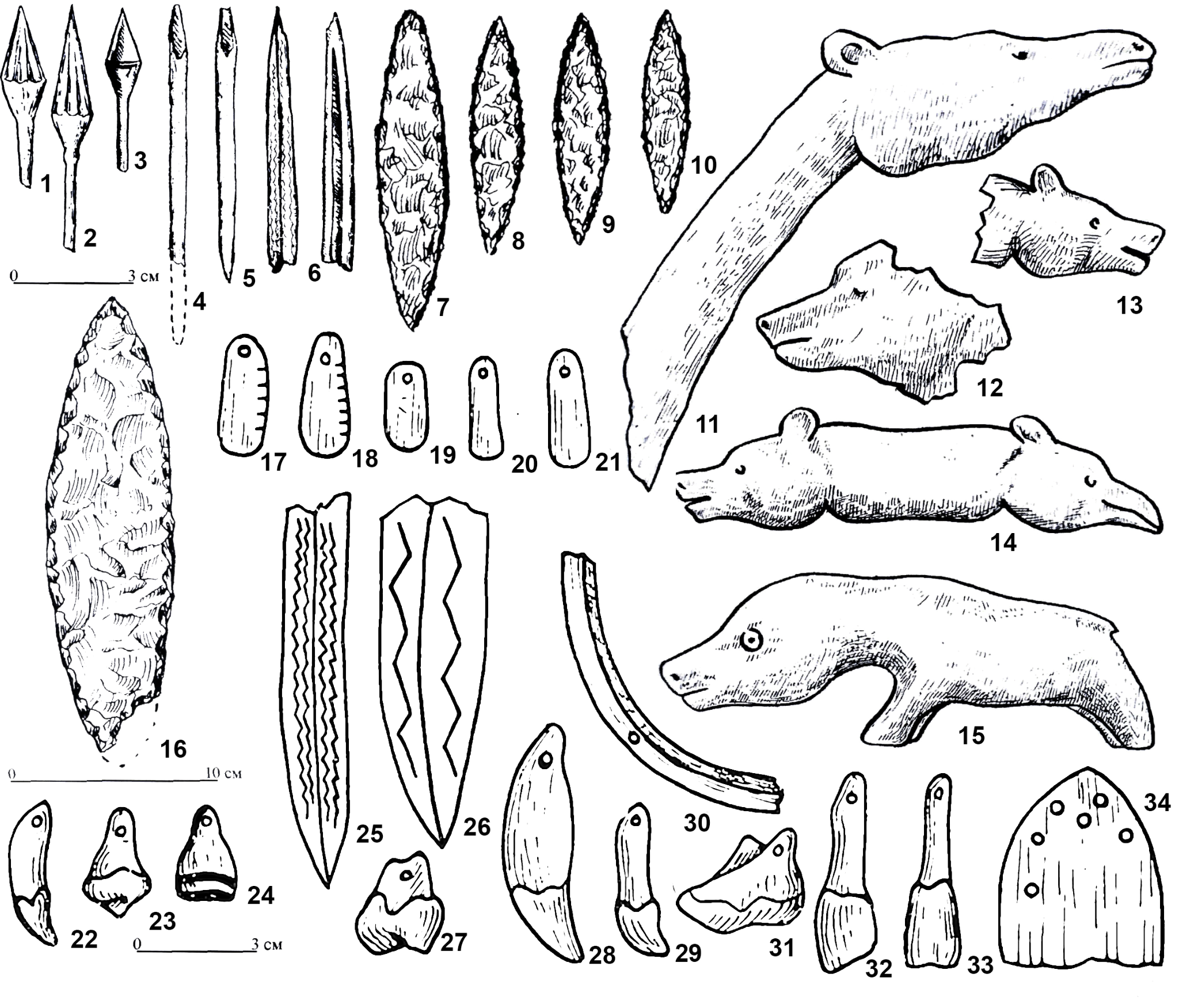


***Figure S20.*** *Grave goods from the burials at Vas’kovo-4. Attribution of the goods to specific burials is unknown.* ***1-3, 7-10*** *- arrowheads;* ***4-5*** *- pointed rods;* ***6*** *- knife base;* ***11-15*** *- zoomorphic figurines (no scale);* ***16*** *- bifacial knife;* ***17-21*** *- plate pendants;* ***22-24, 27-29, 31-33*** *- pendants made of animal teeth;* ***25-26, 30, 34*** *- other items made of bone and antler (by Borodkin 1976).*

##### Archaeological site complex at the Itkul’ Lake

The complex of Neolithic archaeological sites at the Itkul’ Lake includes two burial grounds, **Bolshoy Mys** and **Kostenkova Izbushka**.

The **Bolshoy Mys** burial site is situated across from the village of Vershinino, approximately 4 km from the village of Pleshkovo. It was initially discovered and partially damaged during economic activities in 1954 by A.D. Sambursky. Subsequent investigations were carried out in 1962 by B.Kh. Kadikov and V.I. Molodin. The findings from the excavations have been fully published (Dremov, 1997; Kiryushin et al. 2000; Molodin, 1999).

The original number of burials at the site was at least 18, including two burials that were destroyed by A.D. Sambursky. The burial ground is located at the end of a promontory that extends from the northeastern shore of Lake Itkul towards its center. According to Yuri Kiryushin, during the Neolithic-Eneolithic period, the area was an island (Kiryushin, Maloletko, 1986).

The burials are arranged in three rows across the cape, following a northwest-to-southeast line. The northeastern row consists of burials 14-16. Burials 1-8 and 10-13 are grouped in the central row. The southwestern row includes the destroyed burials discovered earlier, as well as burials 9 and 17. The excavations have investigated 16 burials, and the preservation of the remains in all of them is poor. Burial 17 was partially disturbed by a badger's burrow. Men of various ages, ranging from 30 to 70 years old, were buried in single graves 1-3, 9, 11-13, and 15. Individual burials of women (aged 18-55) include graves 4, 14, and 16. Female burials 5-6 and 7-8 appear to be paired burials (Kiryushin, Kungurova, Kadikov, 2000). Burial 17 is also a pair, but there is no consensus regarding the age and sex of the individuals buried within it. According to V.I. Molodin, it consists of a man (about 18 years old) and a woman (about 25 years old). However, V.A. Dremov believes that both buried individuals are women, aged 40 and 25, respectively. Individual burial 10 contained the remains of a child aged 1.5-2 years old.

The characteristics of the grave pits are not described in the publications, with only mentions of visual observations regarding filling spots (Kiryushin, Kungurova, Kadikov, 2000). Based on photographic illustrations of the excavation process, it can be inferred that the human remains were placed in shallow, approximately rectangular soil pits that corresponded in size and proportions to the size of the individuals being buried.

In all cases, the skeletons were positioned on their backs, in an extended posture. Burials 1-4 and 11-13 exhibit a distinctive characteristic - the hands are placed on the pelvis, and the shins are flexed. This positioning is not consistently observed in burials 8, 10, 14, and 17. The state of preservation in other burials does not permit a definitive determination of the presence or absence of this characteristic.


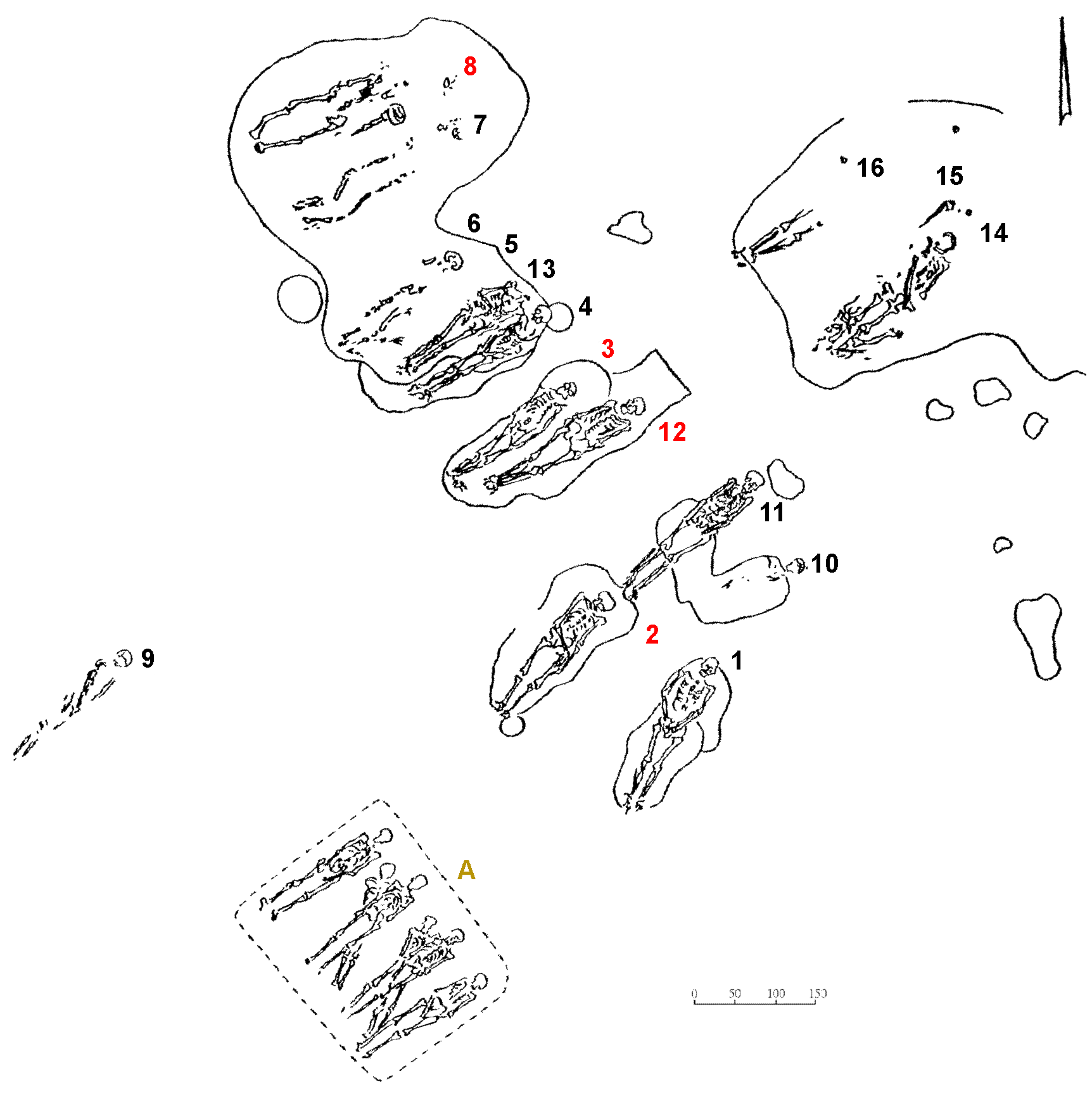


***Figure S21.*** *Bolshoy Mys burial ground. The burials containing sequenced individuals are labeled in red.* ***A*** *- approximate position of the burial destroyed in 1954 during the construction of a dugout building (an individual from this burial was sequenced) (image by Yurii Kiryushin).*

The **Kostenkova Izbushka burial ground** is located in close proximity to the village of Pleshkovo and is within direct line of sight from the Bolshoy Mys burial ground. It was discovered and studied in 1981 by Yuri Kiryushin during excavations of the Bronze Age settlement bearing the same name. The materials from the early complex have been extensively published (Kiryushin, 1983; Kiryushin, Kungurova, Kadikov, 2000). The early burials at the site are associated with the Bolshoy Mys Eneolithic culture (Kiryushin, 1986; Kiryushin, Kungurova, Kadikov, 2000). Given the current water level in Lake Itkul, the burial ground is confined to a cape-shaped ledge along the shore of Dergach Bay. Unfortunately, there is no available data regarding the planigraphy of the monument.

The burial pits have not been explicitly recorded. The individuals were interred in a supine position. Burial 4 is notable for the partial disturbance of anatomical integrity, with only the long bones of the skeleton preserved. A distinctive feature of the remains from burial 5 is the positioning of the left hand on the pelvis (as the bones of the right hand have not survived), and a slightly flexed position of the legs relative to the upper part of the skeleton (Marochkin 2014).

###### Itkul’ (Bolshoy Mys), burial 2 (individual ID I11003, male)

The grave had irregular oval outlines. Within it lay the skeleton of a man aged 30-35, positioned supine with his head facing northeast. His left arm extended alongside the torso, while the right arm was bent at the elbow, with the ulna bones resting on the pelvis and the hands clasped together. The skull exhibited a tilt to the left. Notably, the bones of the feet were absent. A red-brown schist flake was discovered approximately 5-6 cm deeper under the lower jaw of the buried individual. Adjacent to the upper part of the right humerus was a stone hatchet. A grinding rectangular block was found near the left fold on the interior. The majority of the jewelry was concentrated around the neck area and the region of the right pelvic bone. Hanging pendants made of large deer teeth were positioned along the shoulder line, beneath the lower jaw. On the left side of the chest, two large teeth from a bear and a fox were placed. Pendants crafted from small teeth of mustelids and artiodactyls (roe deer) were situated near the head of the right femur (3 pieces), beneath the right wing of the pelvis, on the ulna bones of the right hand, and under the sacrum. The height of the buried individual was measured at 1.65 m (Kiryushin et al. 2000).


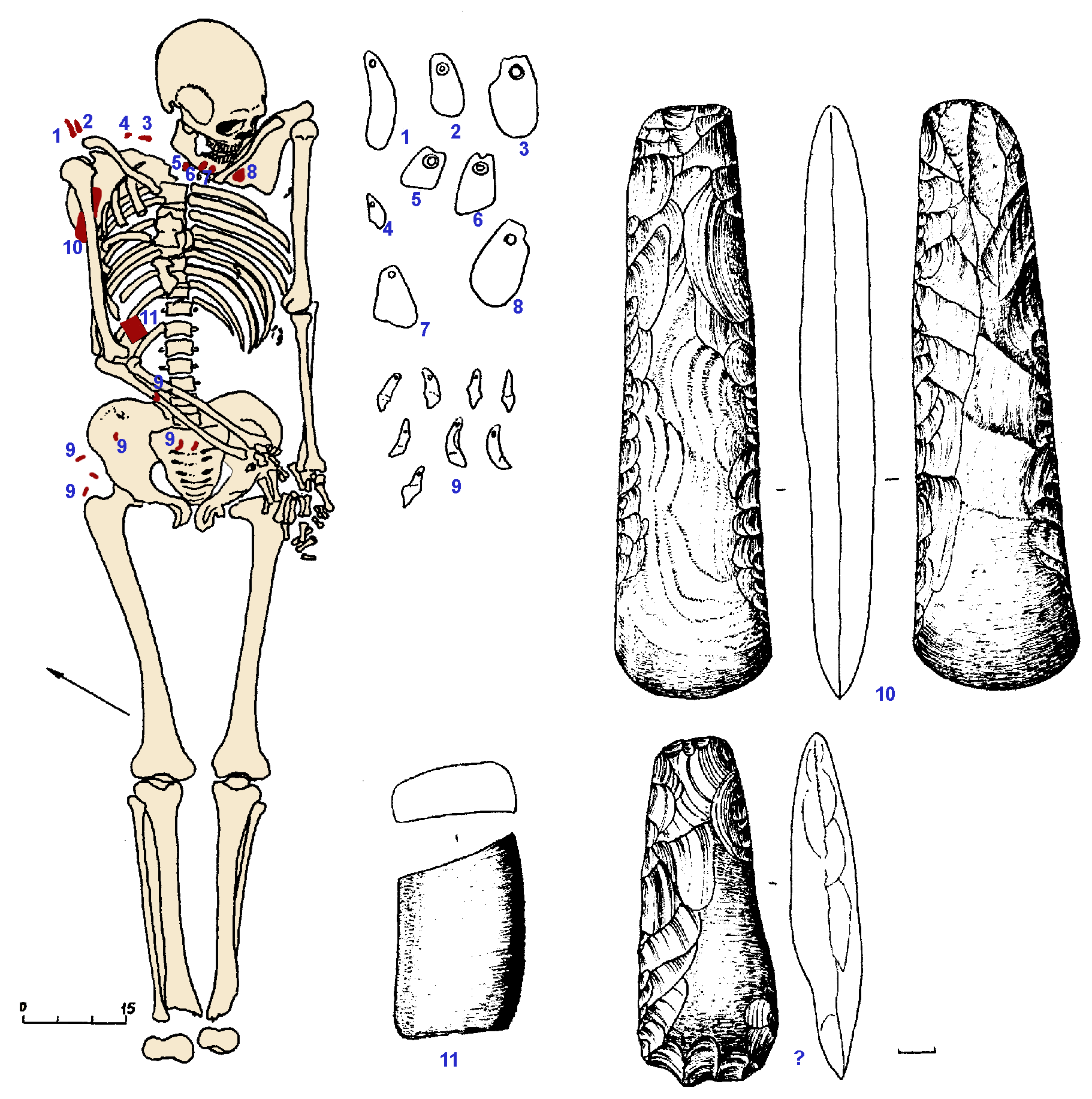


***Figure S22.*** *Burial 2 at Itkul’ (Bolshoy Mys).* ***1-11*** *- grave goods:* ***1-9*** *- pendants;* ***10*** *- stone hatchet;* ***11*** *- grinding block (image by Yurii Kiryushin).*

###### Itkul’ (Bolshoy Mys), burial 3 (individual ID I13676, male)

Located in the central row of the burial ground, this burial is paired with burial 12. The skeleton of a man aged 25-30 was found in an extended supine position with the head oriented towards the northeast. His arms were extended alongside the body, slightly bent at the elbows, with the wrists resting on the pelvic bones. Only a few phalanges of the hands and feet were preserved, and they were found in a disordered state. The right ulna bone was flexed. The clavicles, ribs, and sternum exhibited displacement, and the lower jaw was wide open. Within the burial, a small quartzite flake was discovered between the humerus and the scapula. In the area of the sternum, incisors of a rodent (beaver) were found, including a whole tooth and a fragment, as well as a badger's tooth. The total length of the skeleton was measured at 1.65 m (Kiryushin et al. 2000).

###### Itkul’ (Bolshoy Mys), burial 8 (individual ID I1964, male)

Positioned in the central row and forming a pair with **burial 3**, this burial had a rectangular shape in the northeastern portion of the grave pit. Based on the fillings of the graves, it is evident that **burial 3** was made subsequent to **burial 12**. The skeleton within **burial 12** was found in anatomical order and belonged to a man aged 60-70. He was buried in an extended supine position with the head facing northeast. The ribs on the left side displayed slight displacement, and the phalanges of the arms and legs were in a disordered state. The hands were slightly bent at the elbows, directed with the wrists towards the hip and pelvic joints. Pieces of red stone were found on the outer side of the right pelvic bone (Kiryushin et al. 2000).

###### Itkul’ (Bolshoy Mys), burial 12 (individual ID I12874, male)

This burial is paired with **burial 7**, as the bones of the individuals in both burials were located at a distance of 0.25 m from each other and at a depth of 0.85 m. The skeleton of a woman aged 25-35 was found in anatomical order, lying stretched out on her back with her head facing east, and her arms placed alongside her body. The right arm was slightly bent at the elbow. The skull was crushed and displaced towards the left shoulder, while the lower jaw remained in an anatomical position. The skeleton exhibited the absence of many small and thin bones, such as ribs, joints, and phalanges. In proximity to the right knee, a stone rod of a fishing hook was discovered at a distance of 0.50 m. Jewelry items including incisors of a beaver or marmot (13 pieces), a deer tooth pendant, and a roe deer tooth were found in the neck region and to the left of the vertebra on the chest (Kiryushin et al. 2000).


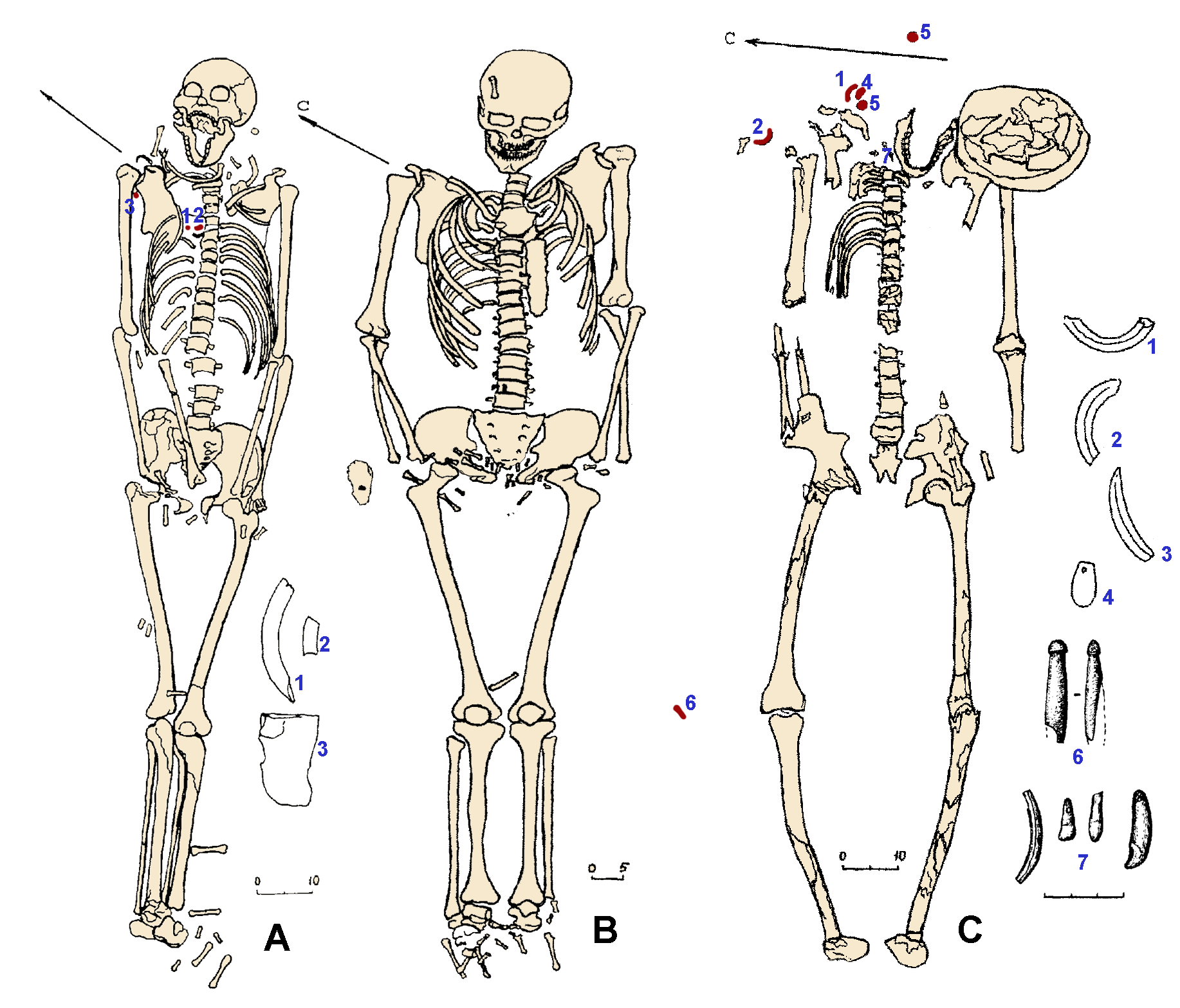


***Figure S23.*** *Burials 3, 12, and 8 at Itkul’ (Bolshoy Mys).* ***A*** *- burial 3:* ***1-2*** *- teeth of beaver and badger;* ***3*** *- quartzite flake;* ***B*** *- burial 12;* ***C*** *- burial 8:* ***1-3*** *- beaver’s and badger’s incisors;* ***4*** *- pendant;* ***5*** *- gypsum desert roses;* ***6*** *- a fragment of a fishing hook;* ***7*** *- perforated animal teeth (image by Yurii Kiryushin).*

###### Itkul’ (Bolshoy Mys), burial 17, individual 1 (individual ID I12873, male)

The burial was excavated by V.I. Molodin in 1976. Two individuals were buried at a depth of 1 m from the present surface. The skeletons were positioned supine with their heads facing northeast, and their arms were extended alongside their bodies. The upper part of both skeletons had been disturbed by a badger hole, with the skulls of both individuals found in different places within the hole.

***Skeleton 1*** (referred to as "A", individual ID I12873*)*, according to the assessment of anthropologists V.P. Alekseeva and N.N. Mamonova, belonged to an 18-year-old young man. The genetically determined sex of this individual is male.

***Skeleton 2*** (referred to as "B") belonged to a 25-year-old woman. Next to the right hand of the deceased, a large bone needle case made from a tubular bone of an animal was found.

On ***skeleton 1***, a significant number of decorations were discovered in the area of the chest and neck, as well as on both skeletons in the waist area, below the pelvic bones and hands, and around the wrists. These decorations, which were likely sewn onto their clothing, included bone pendants with hanging holes, pendants in the form of stylized fish, drop-shaped ("petal") pendants, marmot incisors, threads made from tubular bones, nacre clothing appliques made from shells. V.I. Molodin, the excavator of the burial, noted the similarity of the costumes worn by both deceased individuals (Kiryushin et al. 2000).

###### Itkul’ (Bolshoy Mys), waste of dugout (burial A) (individual ID I1962, male)

One more sample from Itkul’ (Bolshoy Mys) comes from the waste of a dugout (individual ID I1962) that destroyed a Neolithic burial in 1954. According to the description made by the developer, he unearthed two to four skeletons oriented to the northeast. This burial is labeled as A. No specific details of the funeral rite were recorded, and human remains were collected in the course of the exploration of the dugout waste by archaeologists. The individual is dated to 6226–6031 calBCE (7260±40 BP, Poz-82197).


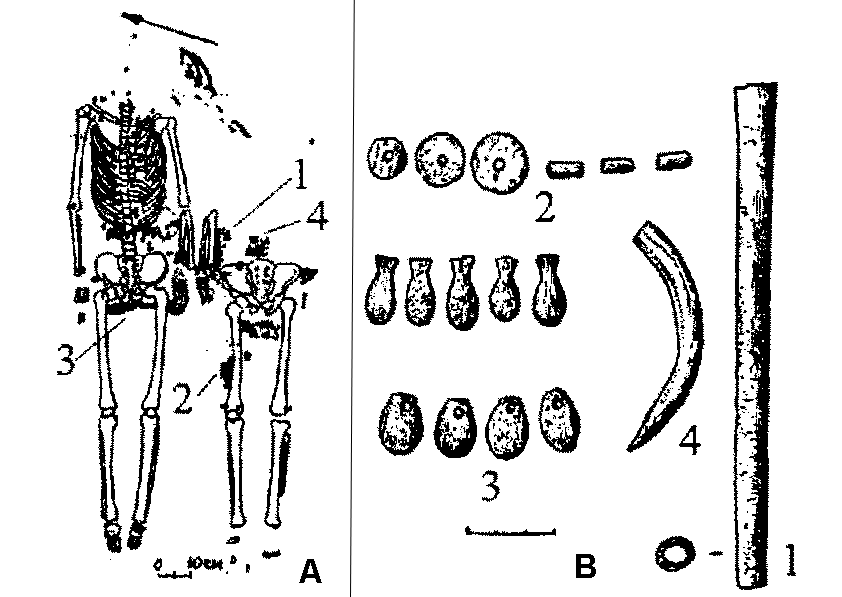


***Figure S24.*** *Burial 17 at Itkul’ (Bolshoy Mys).* ***A*** *- burial 17;* ***B*** *- grave goods:* ***1*** *- needle case;* ***2*** *- nacre clothing appliques;* ***3-4*** *- pendants (image by Yurii Kiryushin).*

###### Kostenkova-Izbushka, burial 3 (individual ID I2137, female)

**Burial 3** was attributed to the Late Bronze Age, and additional information about it has not been published in any papers describing the Neolithic burial site at Kostenkova Izbushka. However, the radiocarbon date of the human bone sample revealed it is Neolithic, 6226–6031 calBCE (7260±40 BP, Poz-82197), and the burial must be treated together with **burials 4** and **5** from the same site.

###### Kostenkova-Izbushka, burial 5 (individual ID I20312, female)

**Burial 5** was situated within the cultural layer of the settlement at a depth of 0.5 m, resulting in indistinct outlines of the grave spot. The grave contained an incomplete skeleton of an approximately 40-year-old man, interred on his back with his head oriented towards the southwest. The skeletal positioning appeared somewhat unnatural. The left leg was extended in an east-west direction, while the right leg was bent at the knee. The southwest direction was determined based on the orientation of the pelvis. The right arm and the bones of the right half of the torso were missing. The left arm was bent at the elbow, with the wrist resting on the pelvic bones. In the vicinity of the base of the skull and the cranial-maxillary cavity, a biconical bone arrowhead was discovered. Fragments of vessel walls were also found within the grave. Adjacent to the right knee, the upper jaw of a beaver was present, while the second upper jaw of a beaver was located to the left of the skull in the chin area.

The deceased individual had been struck by an arrow with a bone tip, which was buried alongside him. The shot was fired at close range from above, descending downwards. The arrow penetrated through the mouth, resulting in the breakage of the top two right incisors and the dislodgement of the bottom three incisors. The impact was so forceful that it caused the jaw, where the tooth sockets were situated, to collapse from the inside. It appears that the arrow pierced the larynx, grazed the cervical vertebrae, and continued towards the left. There are visible marks of the impact on the cervical vertebrae. In this case, distinguishing between posthumous ritual and the act of killing, along with its potential nuances, is quite challenging. The arrowhead type is similar to the needle-shaped tips found at the Shigir culture sites. It exhibits a round cross-section, wider in the region of the tip. Comparable examples include an arrowhead from burial 6 of the Protoka burial ground (Polosmak, Chikisheva, Balueva, 1989).


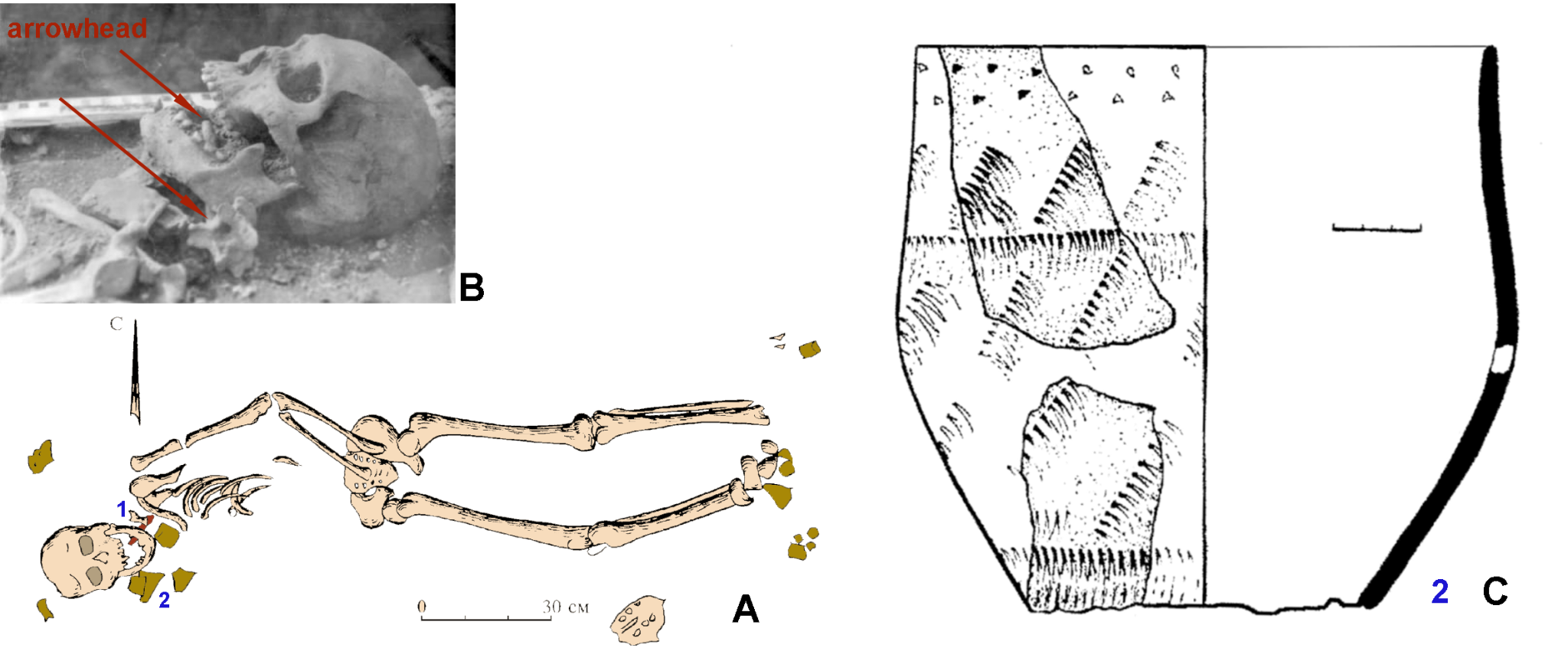


***Figure S25.*** *Burial 5 at Kostenkova Izbushka.* ***A*** *- burial 5 (****1*** *- arrowhead;* ***2*** *- pottery vessel);* ***B*** *- position of the arrowhead in the burial;* ***C*** *- pottery vessel (image by Yurii Kiryushin).*

##### Ust’-Isha site

The Ust’-Isha burial ground is situated near the village of Most-Isha, on a cape-shaped section of the elevated riverbank of the Ishi River, where a small stream flows into it. The site was initially discovered and investigated by B.Kh. Kadikov in 1961, with the majority of the findings already published.

The cemetery comprises a total of 14 burials, of which 11 are securely attributed to the Neolithic period. Two additional burials, with the skeletons positioned in a crouched posture on the left side (with no detailed description available), are presumed to also belong to the Neolithic period (Marochkin, 2014). Lastly, one female burial dates back to the early Iron Age. The burials are arranged in a single row, oriented along a northwest-southeast line, perpendicular to the cape's direction (southwest-northeast).

Several burials (no. 1, 2, 3, 12) have suffered partial destruction due to activities related to the early Iron Age settlement and burial ground. Burials 1, 4, 8, and 9 contain the remains of males aged 30-60 years. Females (about 30 years old) are interred in burials 2, 6, and 7. Burials 3, 5, and 12 contain the remains of children and adolescents, with their sex undetermined. Burials 2, 3, and 12 are double burials, while the remaining burials are single. The primary burial structure consists of soil pits, whose dimensions and proportions are determined by the size of the interred remains. In some cases, shale deposits were observed at the filling level along the pits' perimeters. The most common position for the corpses is extended on their backs. Burials 7 and 8 exhibit a distinctive position, with the hands placed on the pelvic bones and the shins tightly drawn towards the body. Two burials were made in a crouched position. Evidence of multi-stage burials (lack of joints and small skeletal elements) can be observed in burials 12 (a, b) and 7 (adolescent burials). Additionally, the teenage burials at Ust’-Isha show indications of partial cremation.

All burials are oriented with the feet pointing downstream the main reservoir, along the cape. Regarding cardinal directions, there is minimal variability: all male burials and one child burial exhibit a northeast-facing orientation, while female remains are positioned with their heads to the east (two burials) and east-southeast (one burial) (Kiryushin, Kungurova, Kadikov, 2000; Marochkin, 2014).

###### Burial 4 (individual ID I20314, male)

The skeleton of a 30-35-year-old man was found at a depth of 0.45 m from the surface, lying on his back with his head to the northeast. The back of his head was elevated, and his arms were positioned alongside the body. Adjacent to the waist, a large stone dagger and a bone-slotted dagger were discovered. Beneath the left leg, a flake and a bone awl were found. Numerous bone clothing appliques and pendants were present in the skull and torso region. The skull displayed 8-shaped appliques. Fish-shaped ornaments adorned the left side of the body, forming chains and rows. These decorations encircled the left arm and were most densely concentrated in the upper part of the sacrum, demarcating the upper third of the pelvis. Clothing appliques made from small incisors of a rodent (possibly a marmot or beaver) extended from the left elbow to the middle of the humerus. Similar decorations were also observed on the middle section of the right humerus. Large animal teeth were arranged in a chain-like pattern, extending from the back side, spanning from the right shoulder to the waist, and forming a diagonal row between the human hips. Several large animal teeth were noted beneath the pelvic bones. The height of the buried individual was estimated at 1.60 m (Kiryushin, Kungurova, Kadikov, 2000). The burial is dated to 4442–4266 calBCE (5500±25 BP, PSUAMS-9052, the date on a human bone/tooth).


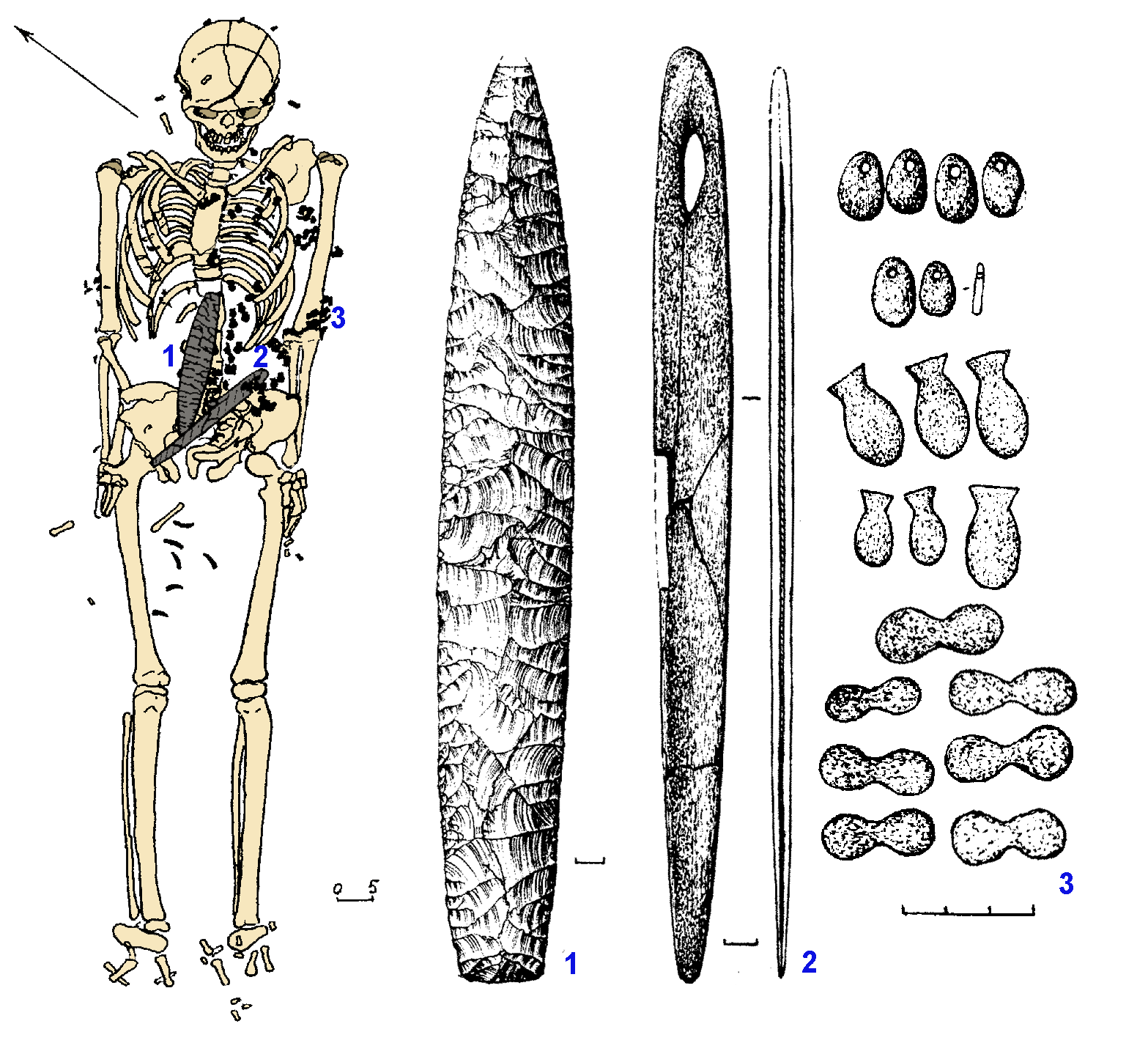


***Figure S26.*** *Burial 4 at Ust’-Isha.* ***1*** *- stone dagger;* ***2*** *- bone dagger;* ***3*** *- pendants (image by Yurii Kiryushin).*

###### Burial 6 (individual ID I1963, male)

This burial was found at a depth of 0.30 m from the modern surface. The skeletal remains were highly dispersed and incomplete within the grave. However, the arrangement of the arm, sacrum, pelvis, and skull suggests an extended supine position for the interred individual. The skull, which was fragmented, lay on the right side. Presumably, the deceased was placed with their head pointing northeast. The burial lacked any ornaments or objects, with the exception of a dart tip located 25 cm to the left of the burial. The total height of the buried individual was 1.52 m. The sex was not determined, and the estimated age ranged from 40 to 50 years (Kiryushin, Kungurova, Kadikov, 2000). The burial is dated to 3957–3796 calBCE (5076±29 BP) [R_combine: (5490±40 BP, Poz-82209); (4585±40 BP, OxA-33424)].

###### Burial 7 (individual ID I2142, male)

The grave stain was traced from a depth of 0.28 m from the modern surface. The skeleton of a 20-30-year-old woman was discovered at a depth of 0.70 m from the surface, in a supine position with her head to the southeast. The left arm was extended alongside the body, while the right arm was slightly bent at the elbow and placed on the pelvis. The woman's skull was crushed and turned towards the left side. The costal bones were poorly preserved, the pelvic bones were crushed, and the spinal bones were missing. In the neck area, several pendants made of deer and elk teeth were found. Two pendants discovered in an empty lower chest appeared to be part of a previous cluster. The length of the skeleton was 1.62 m. The skulls of **burials 6** and **7** were located approximately 0.40 m apart from each other (Kiryushin, Kungurova, Kadikov, 2000). The burial is dated to 5300–5032 calBCE (6200±40 BP, Poz-82207).


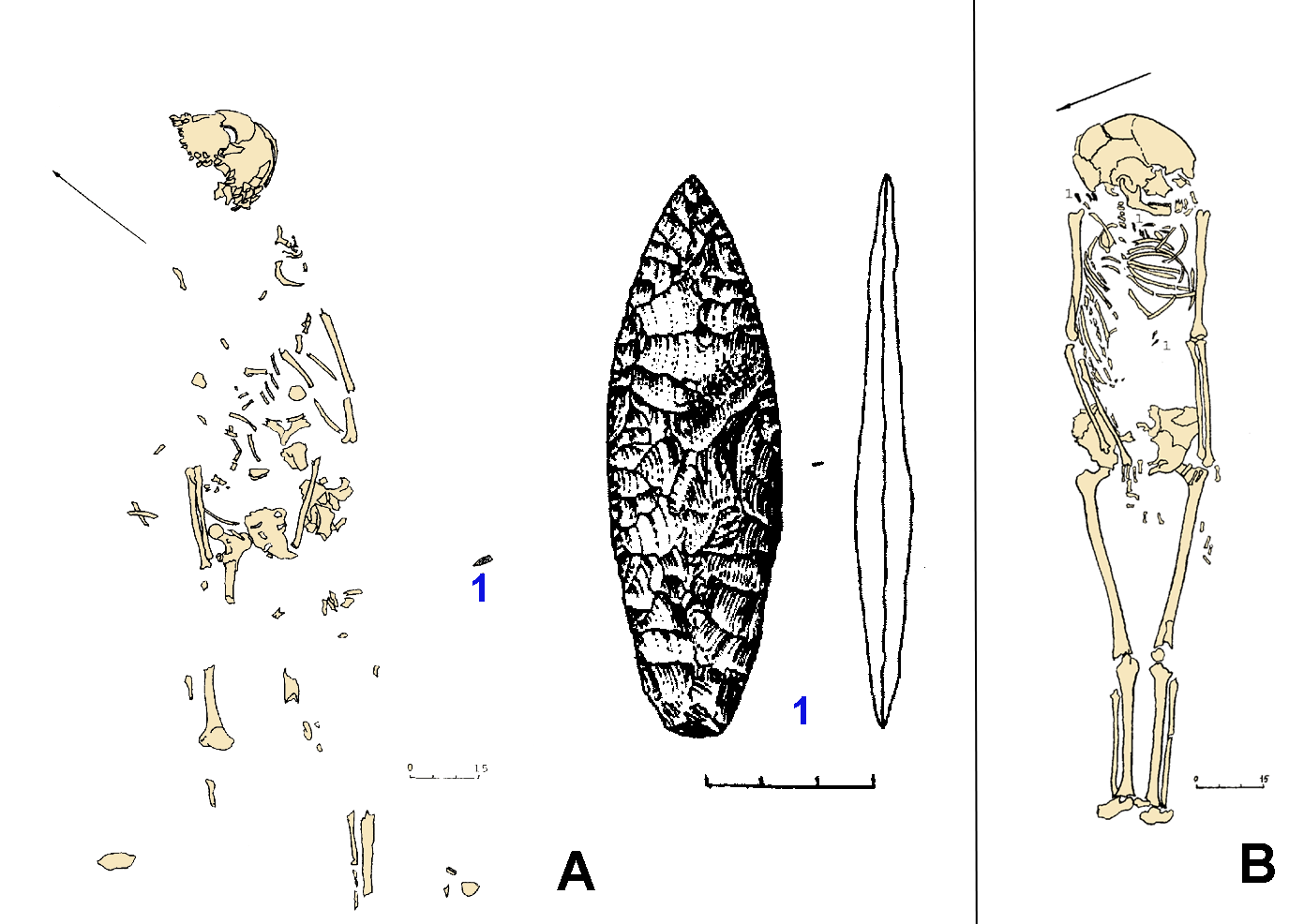


***Figure S27.*** *Burials 6 and 7 at Ust’-Isha.* ***A*** *- burial 6 (****1*** *- point of a javelin);* ***B*** *- burial 7 (image by Yurii Kiryushin).*

###### Burial 8 (individual ID I12876, male)

A 25-30-year-old male was interred in a supine position with his head facing northeast and his arms extended alongside his body. The right shoulder was inwardly rotated, while the head was slightly bowed and turned to the left side. Within the waist region of the skeleton, four daggers were present, arranged in pairs. In the center was a shortened stone dagger and a large slotted bone dagger, while on the right side, two identical slotted daggers were positioned. A compact cluster of 16 arrowheads was situated 30 cm from the left femur. The burial exhibited an abundance of decorative elements such as beads, bone patches, and pendants. A scattering of beads was found opposite the left humerus, within a 15 x 30 cm section. Along the arm, clusters of pendants made from large incisors of maral, elk, beaver, and two hollow bones of a bird were observed. A total of 3-4 clusters, resembling "ligaments" of large incisors, were noted. Beads were also present in the area spanning from the waist to the middle of the thigh bones. Positioned between the femurs in the upper part, two flaps of denticulate shells were found. On the right side, descending in a line from the belt area to the upper third of the pelvis, eight-shaped bone clothing appliques were present. The left half of the skeleton was adorned with "petal" pendants made of deer teeth, descending along the left arm, curving around it, and tightly clustered in the elbow area. Double pendants crafted from elk teeth were positioned beneath the skeleton along the spine. Concentrated on the skull, specifically along the back of the head, forehead, and crown, were clothing appliques formed from teeth of a small artiodactyl animal (roe deer). Within the cluster on the frontal part, 15 such clothing appliques were observed. The height of the buried individual was estimated at 1.70 m (Kiryushin, Kungurova, Kadikov, 2000).


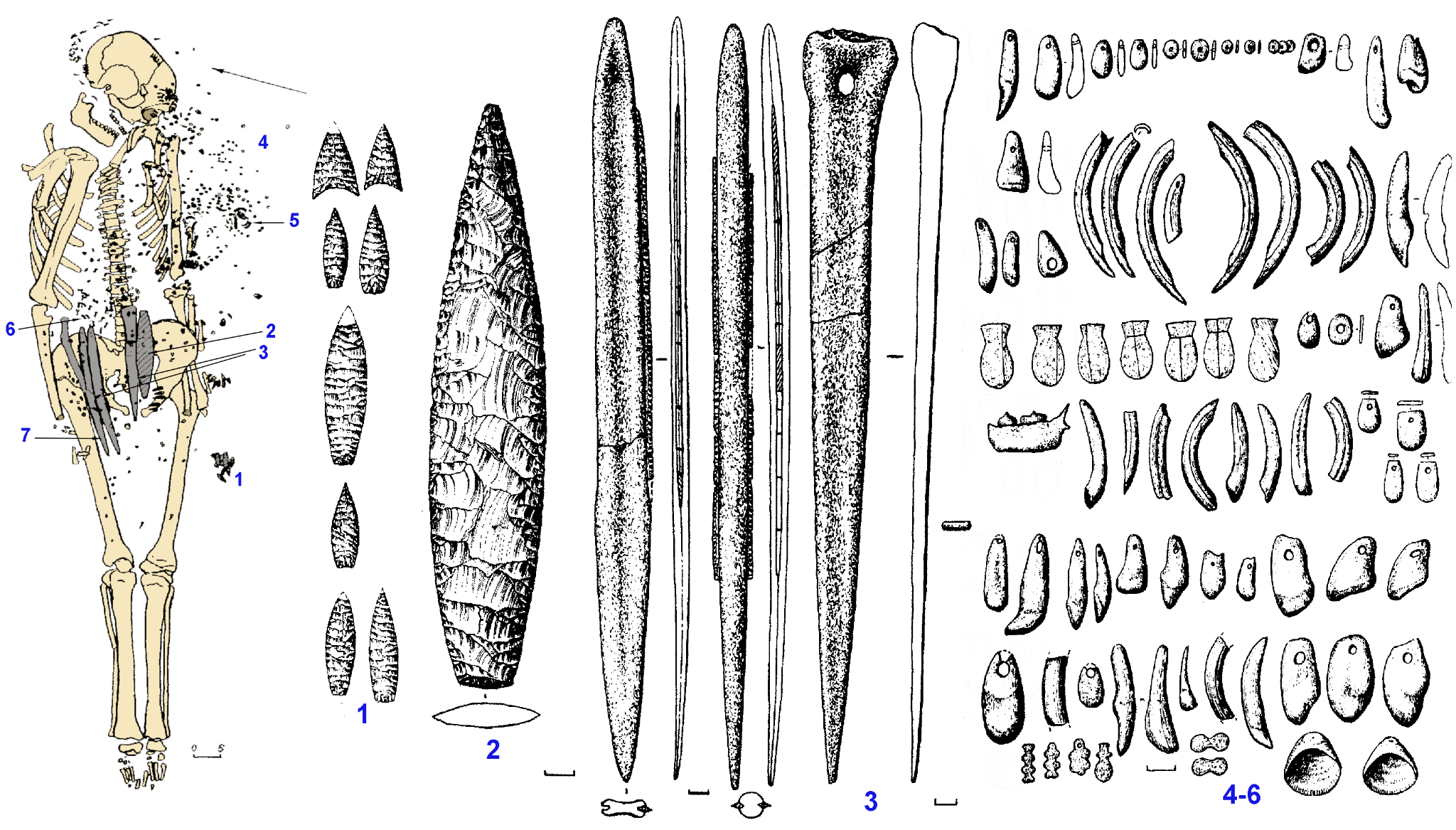


***Figure S28.*** *Burial 8 at Ust’-Isha.* ***1*** *- arrowheads;* ***2*** *- stone dagger;* ***3*** *- bone daggers;* ***4*** *- nacre beads and pendants made of stone and bone;* ***5*** *- beaver’s incisors;* ***6*** *- eight-shaped pendants;* ***7*** *- shell (image by Yurii Kiryushin).*

###### Burials 10 (individual ID I1966, female) and 11 (I1965, male)

**Burials 10** and **11** have not been described properly in the publications. In one of them, a skeleton was placed in a crouched position on its left side with its head to the north-northeast. Another one contained a skeleton in a supine position with the legs bent and put on their right side, oriented to the west-northwest (Kiryushin, Kungurova, Kadikov, 2000). **Burial 10** is dated to 4361–4174 calBCE (5450±40 BP, Poz-82208), and **burial 11** – to 4037–3797 calBCE (5120±40 BP, Poz-82206).

*
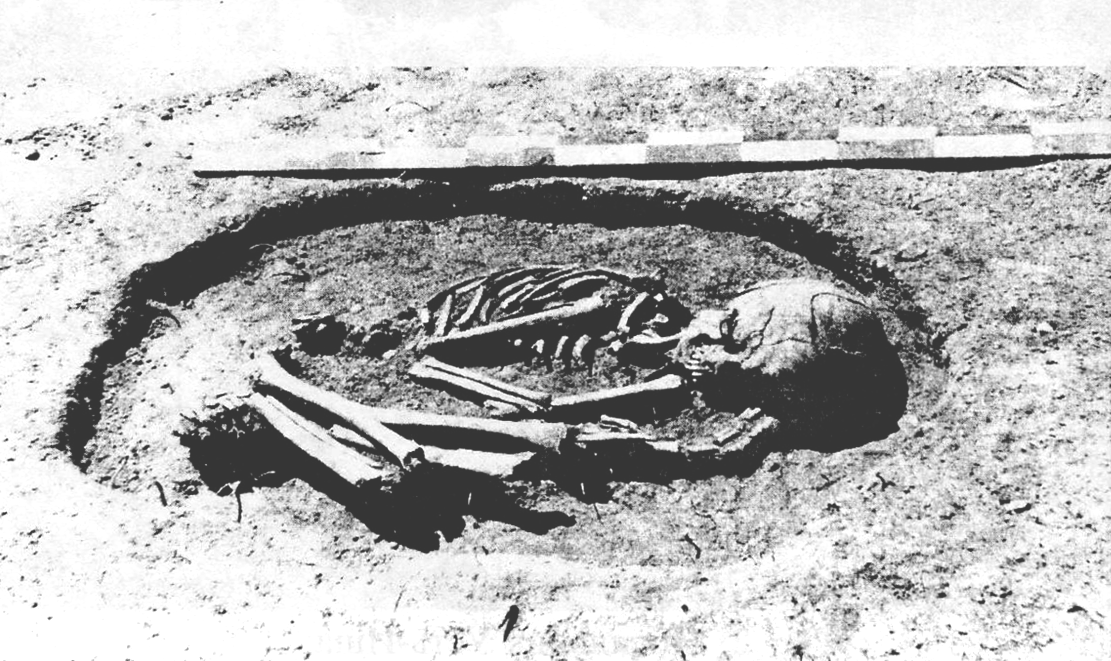
*

***Figure S29.*** *Burial 10 or 11 at Ust’-Isha (image by Yurii Kiryushin).*

##### Firsovo-14 burial site

The Firsovo-14 burial site is situated on the right bank of the Ob River, opposite Barnaul, 1 kilometer west of the village bearing the same name. It is located in the forest-steppe zone of Altai, on a promontory that rises four meters above the floodplain of the Ob. The site is positioned above an oxbow lake of the Losikha River, which is a right tributary of the Ob.

Since 1987, a total area of over 5,000 square meters has been excavated at the site, and approximately 400 burials have been investigated. Among these burials, around 200 belong to the Andronovo culture, about 20 are attributed to the Irmen culture, and over 100 are associated with the Staroaleyka culture. Additionally, single graves representing other periods and burial traditions have been discovered at the site. Over the years, one Korchazhkino burial and two burials displaying the Sargary appearance from the Late Bronze Age were excavated here (Kiryushin et al. 2013).

###### Neolithic burial 267 (individual ID I11754, male)

**Burial 267** was excavated in 1996 at the end of the promontory. The exact outline of the grave pit could not be determined. The skeletal remains were found at a depth of 0.4 meters from the modern surface, within a layer of yellow sandy loam. The deceased was positioned on their right side, with the head facing east. The legs were flexed at the knees and pressed against the body, causing the feet to be near the pelvic bones. The arms were bent at the elbow joints and pressed against the chest. The right hand was positioned near the face, while the left hand was beneath the skull. The overall posture of the skeleton can be described as resembling the "fetal position”. The positioning of the legs and back clearly indicates the limited space within which the body was placed. The placement of the head appears to be unnatural. It is likely that these factors can be attributed to the size of the grave pit, against which the deceased's knees and back were pressed. One should not exclude the possibility that prior to burial, the individual was tightly wrapped or sewn into a shroud. The buried individual was laid with their feet perpendicular to the flow of the Ob River. In relation to the nearby Losikha oxbow lake, the deceased was positioned parallel to the channel, with their back facing the water, head elevated, and feet pointing downstream.

Artifacts made of bone, antler, and stone were discovered within the grave alongside the deceased. All of the findings were grouped together in one location, near the chest, and between the arms. The accompanying funerary inventory consists of the following items:

a 16.5 cm long polished bone "point";

a 12.1 cm long polished antler spatula;

a fragment of a polished antler rod, measuring 5.1 cm in length with a cross-section of 0.9x0.6 cm;

a distal fragment of a harpoon featuring teeth from an animal's rib on one side;

the proximal part of a flake with finely retouched lateral edges;

an abrasive tool measuring 7.1 cm in length, with a side face width of 1.8–2.2 cm and a working surface width of 1.3 cm. It is made of fine-grained sandstone;

a polished knife measuring 7.9 cm in length, 1.4–2.2 cm in width.

The compact arrangement of the accompanying inventory suggests that all of these items could have been contained within some type of handbag, which was placed in the hands of the deceased (Kiryushin et al. 2013). The skeleton is dated to 4994–4797 calBCE (6005±35 BP, PSUAMS-8988). Alternatively, two other dates for the human bones were obtained in another paper, 5318–4846 calBCE (6166±96 BP, NSKA-01942), and 5206-4935 BP (6100±25 BP, IGAN-5831) (Kiryushin et al. 2021). The combination of three dates gives an age of 5194–4905 calBCE (6073±20 BP) [R_Combine: (6005В±35 BP, PSUAMS-8988); (6166±96 BP, NSKA-01942); (6100±25 BP, IGAN-5831)].


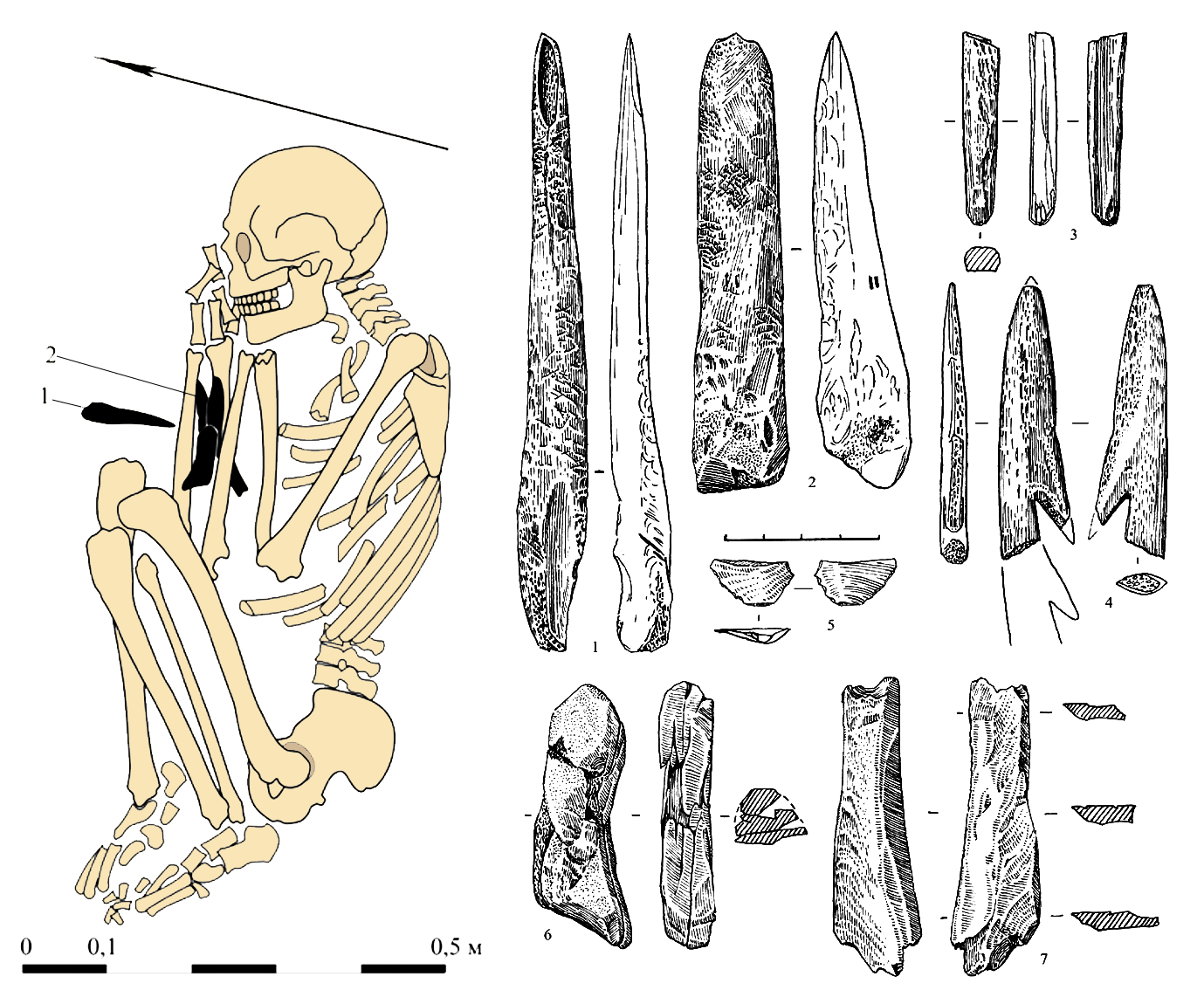


***Figure S30.*** *Burial 267 at Firsovo-14.* ***1*** *- antler spatula;* ***2*** *- grave goods (image by Yurii Kiryushin).*

##### Lebedi-2 site

The Lebedi-2 cemetery is situated on a narrow promontory of the left bank of the middle Inya River. This promontory comprises the second terrace above floodplain. It is formed by loamy, sandy, and sand deposits (Bobrov 1988).

The cemetery consists of six burials and one pit containing crushed animal bones and artifacts (referred to as Pit 5). Burials 1 and 2 were partially damaged due to construction activities. Burials 1, 2, and 7 contain remains of men over 60 years old. Burial 3 belongs to a teenager aged 12-13, and burial 4 belongs to a child approximately 8 years old (in both cases, the sex is undetermined). Burial 6 contains the remains of a woman over 60 years old. Burial 2 is a double burial, while the rest are single burials. The nature of Pit 5 is unclear and its purpose is uncertain (possibly a cenotaph).

The spatial distribution of the burials follows the direction of the cape. The burials are arranged in a row, with two distinct groups within the row: the western group (burials 6 and 7) and the eastern group (burials 1-4). Pit 5 is adjacent to the eastern group.

The burial structures consist of elongated, subrectangular, or oval earth pits. There is no discernible pattern in their form variation. The size and proportions of the pits correspond to the size of the individuals interred within them, with the pits for adolescent burials being shorter. Pit 5 shares similar proportions and shape with the burial structures. All burials are characterized by the placement of the remains in an extended position on their backs. Burials 3 (children), 6 (adult female), and 7 (adult male) exhibit a specific detail where the hands are positioned on the pelvis, and the legs are tightly pressed at the shins.

The remains of the adults (burials 1, 2, 6, and 7) are oriented with their heads towards the northeastern sector in relation to the cardinal points, and with their feet downstream relative to the river. The remains of the children (burials 3 and 4) are oriented with their heads towards the northwestern sector, perpendicular to the river flow and perpendicular to the burials of adults. Pit 5 is aligned along the southwest-northeast axis with its longer side, resembling the orientation of the adult burials (Marochkin 2014).

###### Burial 7 (individual ID I2076, male)

**Burial 7** is a narrow, almost rectangular pit; its dimensions are 2.1 x 0.57 m. The bones belonged to a man of about 60 years. He was interred extended and supine, with the head to the northeast. The position of the arms should be noted. They are extended along the body but the hands are turned toward each other and lie on the pelvis. The accompanying inventory has retained its initial position. The headdress decoration consisted of five beaver incisors (they lay on the forehead), while on each side were sewn six bear teeth. A row of animal teeth (bear, moose, and wolf) was placed along the skeleton, beginning with the neck vertebrae. At the pelvis, this row went to the right toward the radius, then turned to the center of the body, going to the left knee. Such a position conforms with clothing opened below the belt and wrapping to the right. The accompanying inventory was found on the pelvic bones (i.e., it was placed on the belt of the interred). On the left, with point up, lay a wide, leaf-shaped flint dagger. The placement of the fingers on the left hand does not permit a conclusion that the handle was grasped. Between the hands was placed, bottom-up, a round-bottomed vessel of small dimensions. It was decorated with one vertical zigzag line executed by strokes of a narrow, sharpened stick, and another line that was incised. By the vessel and partly on the base of the dagger was a polished knife with a concave blade edge. It was of green serpentine. Under it lay a small bone spoon or spatula with a poorly preserved straight base. To the right of the objects described was placed a small, polished axe. In addition, two beaver incisors were found on the belt (Bobrov 1988, Marochkin 2014). **Burial 7** is dated to 4689–4403 calBCE (5700±50 BP, Poz-83432).


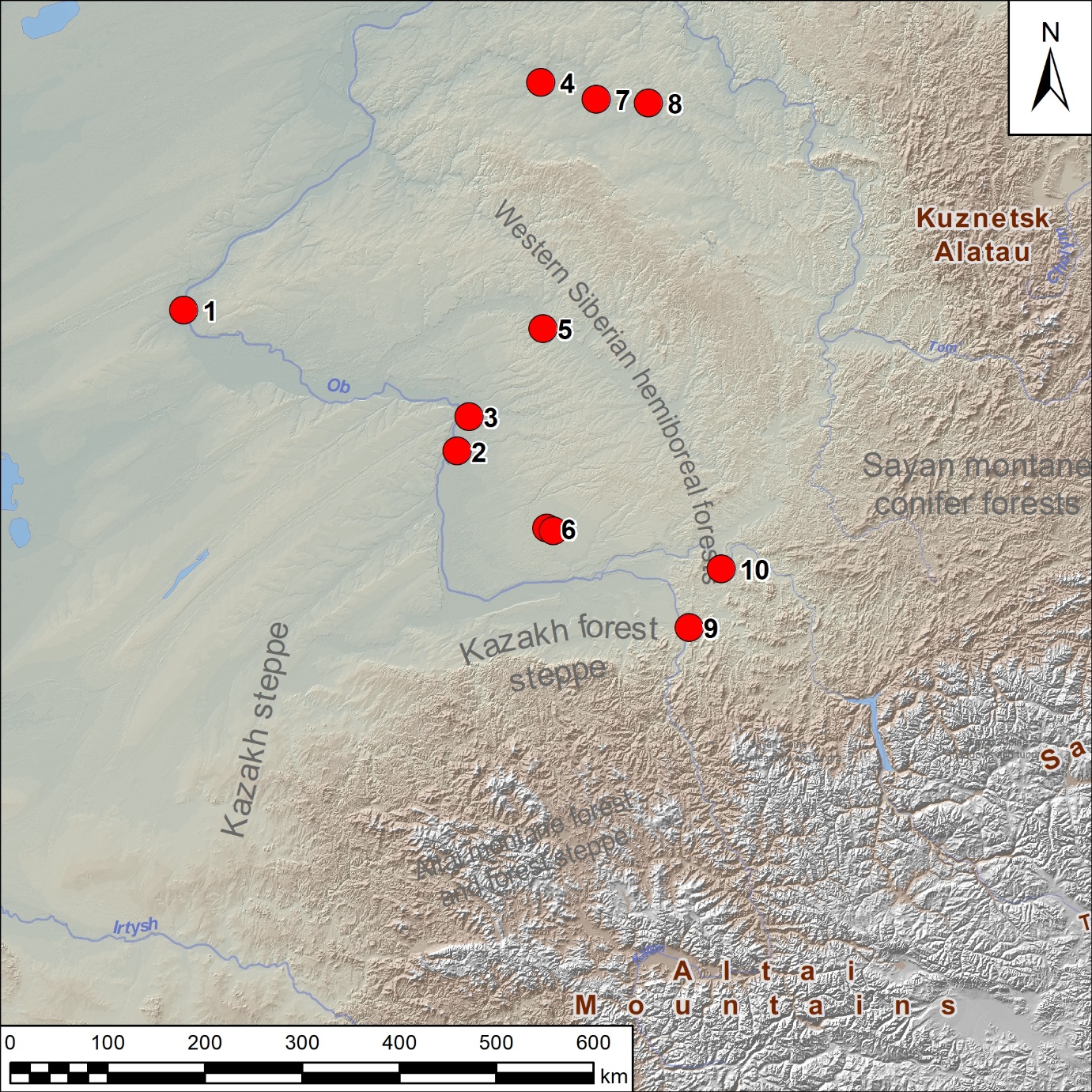


***Figure S31.*** *Neolithic sites in the Upper Ob and Kuznetsk Depression. 1 - Razdum'ye-1; 2 - Tuzovskie-Bugry-1 (Vasino-5); 3 - Firsovo-11; 4 - Zarechnoye-1; 5 - Chumysh-Perekat-1; 6 - Itkul (Bolshoy-Mys) and Kostenkova-Izbushka; 7 - Vas'kovo-4; 8 - Lebedi-2; 9 - Ust'-Isha; 10 - Solontcy-5.*

##### Solontsy-5 site

The Solontsy-5 burial ground is a component of the Solontsy archaeological site complex, situated near the village of Pilno on a narrow cape-like area where the Biya River and its tributary, the Chapshushka River, converge. The complex of sites has been known since the 1920s, with the initial excavation of two Neolithic burials conducted in the 1970s by V.D. Lunev (Kungurova 2005). The primary burial complex was unearthed in the early 2000s during N.Yu. Kungurova's investigation of the Neolithic settlement at Solontsy-2. A comprehensive publication of the materials has been accomplished (Kungurova, Chikisheva 2002; Kungurova 2003, 2005).

The burial ground consists of nine burials, one of which (burial 9) suffered partial disturbance due to medieval settlement activities. Burials 1, 3, 4, 5, and 7 contain the remains of men ranging in age from 30-40 (burials 1 and 4) to 60 years (burials 3 and 7). In one instance, the burial of a teenager was classified as male (burial 5). The remains from burials 2 (about 60 years old), 6 (undetermined age), 9 (about 50 years old), and 8 (about 12 years old) were identified as female.

All burials are individual, except for paired burials 1 (a man and a child) and 8 (two teenage girls). The burials are arranged in two rows aligned along a northwest-southeast axis (burials 1 to 9). Unfortunately, there is no available data regarding the nature of the burial structures as the excavation did not permit the identification of burial pits.

Two distinct positions of the deceased are evident. The first position is extended on the back (burials 1, 4-9). This variant may also encompass the paired burial 8, where the remains of two individuals are superimposed. Burials 1, 4, 8, and 9 exhibit a particular feature with the hands placed on the pelvis and the legs brought together at the shins. The second position is a flexed posture on the left side (burials 2 and 3).

Among the nine burials, six demonstrate anatomical disruption and the loss of small bones, indicating potential multi-stage ("secondary") burials. All burials display a consistent orientation with the heads facing the east-northeast sector and the feet positioned downstream the primary water reservoir, the Biya River. There are variations in orientation between men and women, with men predominantly oriented towards the east-northeast and women towards the east (Marochkin 2014, Kungurova 2003).

###### Burial 1, skeleton 1 (individual ID I2072, male)

The burial contained the remains of an adult male, estimated to be 35-40 years old, buried in an extended supine position with his head facing northeast. The burial was located at a depth of 0.75 meters from the soil surface. Adjacent to the bones of the right hand was a skeleton of a 1.5-year-old child.

***Skeleton 1*** (individual ID I2072): The man's skull had two stone slabs closely positioned against its occipital surface. Along the edge of one of the slabs, a large chopping tool called a biface was placed. A musk deer tusk with notches along its concave edge was placed on this slab. On the right side of the skull, there was a dense accumulation of 73 flakes. A polished axe was found beneath the lower jaw, resting on the upper ribs. Under the left shoulder bone, a dense accumulation of items was discovered, including a pestle with an expanded base, a long stone bar, five bone harpoon tips, one bone end plate for a bow, five stone arrowheads and their fragments, a bone point, and a stone rod for a hook. Underneath these items and the humerus bone, there was a dense accumulation of 34 flint flakes, including a scraper and four retouched flakes. Closer to the stone slab, two stone fishing rods were located. Near the ulna bone of the left hand, two harpoon tips were superimposed, along with a horn chipper adjacent to them, and below that, a narrow polished adze-shaped tool. Two bone rods with hook points inserted into them were found on the phalanges of the left hand. On the right side, from the lower ribs to the pubic bones, two large stone biface blades were positioned: a long dagger and a wide asymmetrical knife. A scraper made of sandstone tiles lay beneath the center of the spinal column. At the distal end of the left tibia, a piece of stone was present. Near the skull, shoulder, and hip joints, musk deer fang points were discovered. Decorative items made of large deer teeth and petal-shaped bone stripes adorned the neck and chest. Additional bone stripes made of teeth of large ungulates were located in the pelvic region and between the femurs.

***Skeleton 2***: The child's skeleton, located near the male, was slightly turned to the left, with the lower part facing the man's forearm bones. The child was buried in an extended supine position. Between the two skeletons, a bone dagger with stone inserts was placed. The bones of the child's left hand were positioned beneath it, indicating that the right hand may have been resting on top (only the ulna bones without phalanges have been preserved). The tibia bones were also found beneath the dagger and partially on it. Under the dagger, near the right knee, there was a cluster of 11 stone arrowheads and their fragments. A valve of a large shell was discovered at the articulation of the right thigh and pelvis, with two more valves of the same type located in the center of the lower part of the chest and near the left shoulder joint (likely originally placed on the chest). Four stone flakes were situated under the mandibula. A piece of hematite (red mineral paint) was found beneath the spine in the waist area. The burial also contained split teeth of a large ungulate, positioned under the dagger, on the bones of the left hand, on the ribs of the left side of the chest, and between them.

The items discovered in this burial comprise a diverse and rich assortment. Comparative morphological analysis of the items from **burial 1** indicates their possible cultural and chronological similarity with artifacts from the Neolithic Burial at Ust-Aleika-5 (Barnaul, Ob) (Borodaev et all, 2022). **Burial 1** is dated to 3958–3713 calBCE (5050±40 BP, Poz-83497).


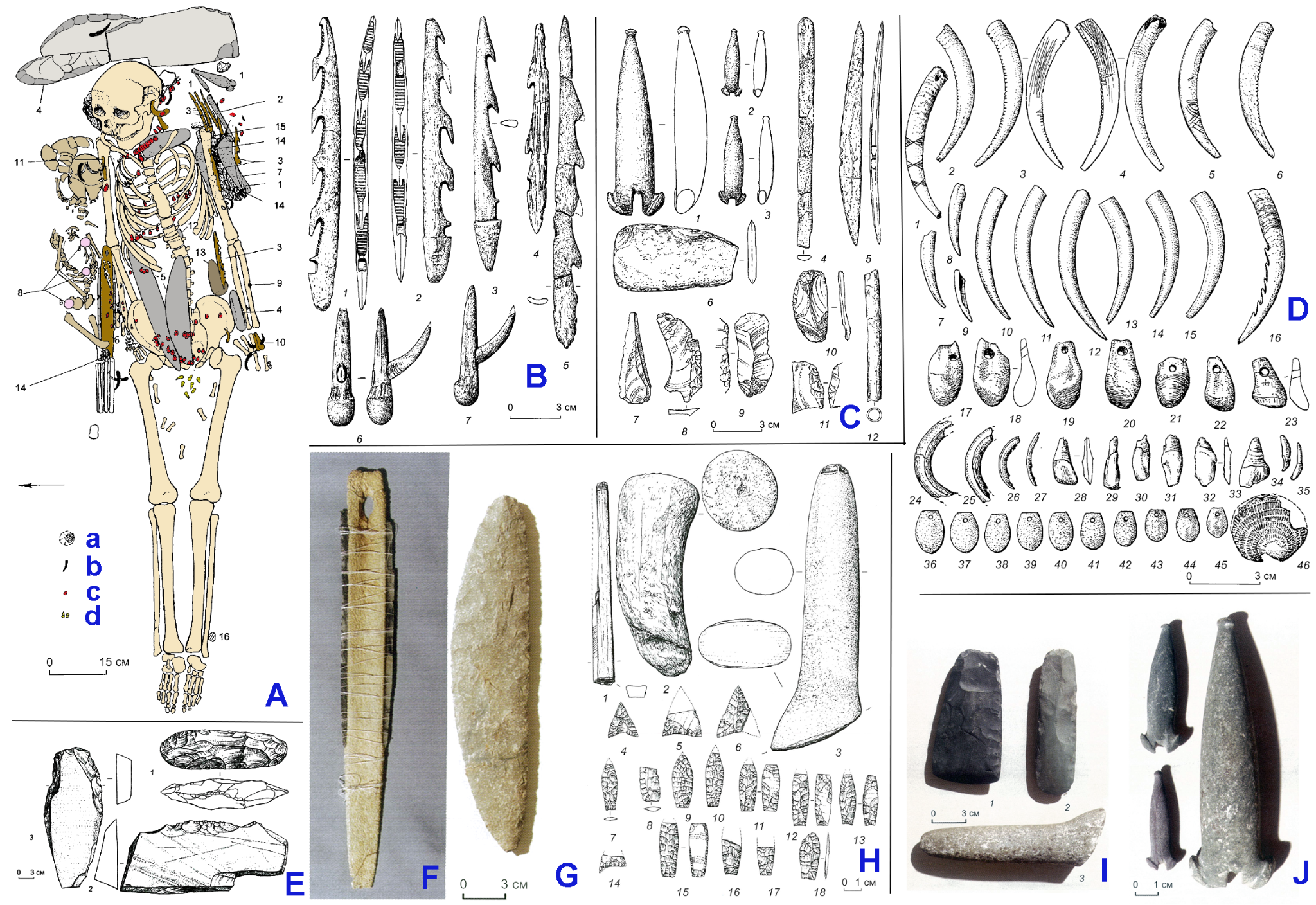


***Figure S32.*** *Burial 1 at Solontsy-5.* ***A*** *- burial 1 (dark color - child bones, light color - adult bones):* ***a*** *- flakes;* ***b*** *- musk deer tusks;* ***c*** *- bone appliques;* ***d*** *- teeth appliques;* ***1*** *- fishing rods;* ***2*** *- grinding stone;* ***3*** *- harpoon tips;* ***4*** *- chisels;* ***5*** *- knives;* ***6*** *- slotted dagger;* ***7*** *- “small flat iron” with a handle;* ***8-9*** *- shells;* ***10*** *- bone hooked rods;* ***11*** *- bones;* ***12*** *- scraper;* ***13*** *- fabricator;* ***14*** *- arrowheads;* ***15*** *- scraper made from a flake;* ***16*** *- stone;* ***B*** *- bone tools;* ***C*** *- various grave goods;* ***D*** *- ornaments, pendants, and appliques;* ***E*** *- bifacial axe and stone plates;* ***F*** *- slotted bone dagger;* ***G*** *- stone dagger;* ***H*** *- stone implements and arrowheads;* ***I*** *- photo of stone tools;* ***J*** *- photo of stone fishing rods (by Kungurova 2003, modified).*

###### Burial 7 (individual ID I2073, male)

The burial consisted of the remains of a 55-60-year-old man buried at a depth of 0.70 meters from the soil surface. The position of the body was supine, with the head oriented towards the east and the arms extended along the sides. The facial bones were compressed within the skull, and the lower jaw exhibited downward and leftward displacement. A circular scarlet pigment spot was observed in the vicinity of the skull and to its left side. Positioned on the left side of the burial was a large stone biface dagger. Additionally, a stone arrowhead was discovered in the lower lumbar region of the spine, with its point directed towards the vertebra. The burial exhibited an abundance of decorative items made of animal teeth and bone petal patches, which were arranged in ribbons. A small flake and a fragment of ferruginous rock used as a pigment were also recovered from the burial. The estimated length of the skeleton ranged from 175 to 180 cm (Kungurova 2005). The skeleton from **burial 7** is dated to 3971–3802 calBCE (5105±25 BP, PSUAMS-4120).

######
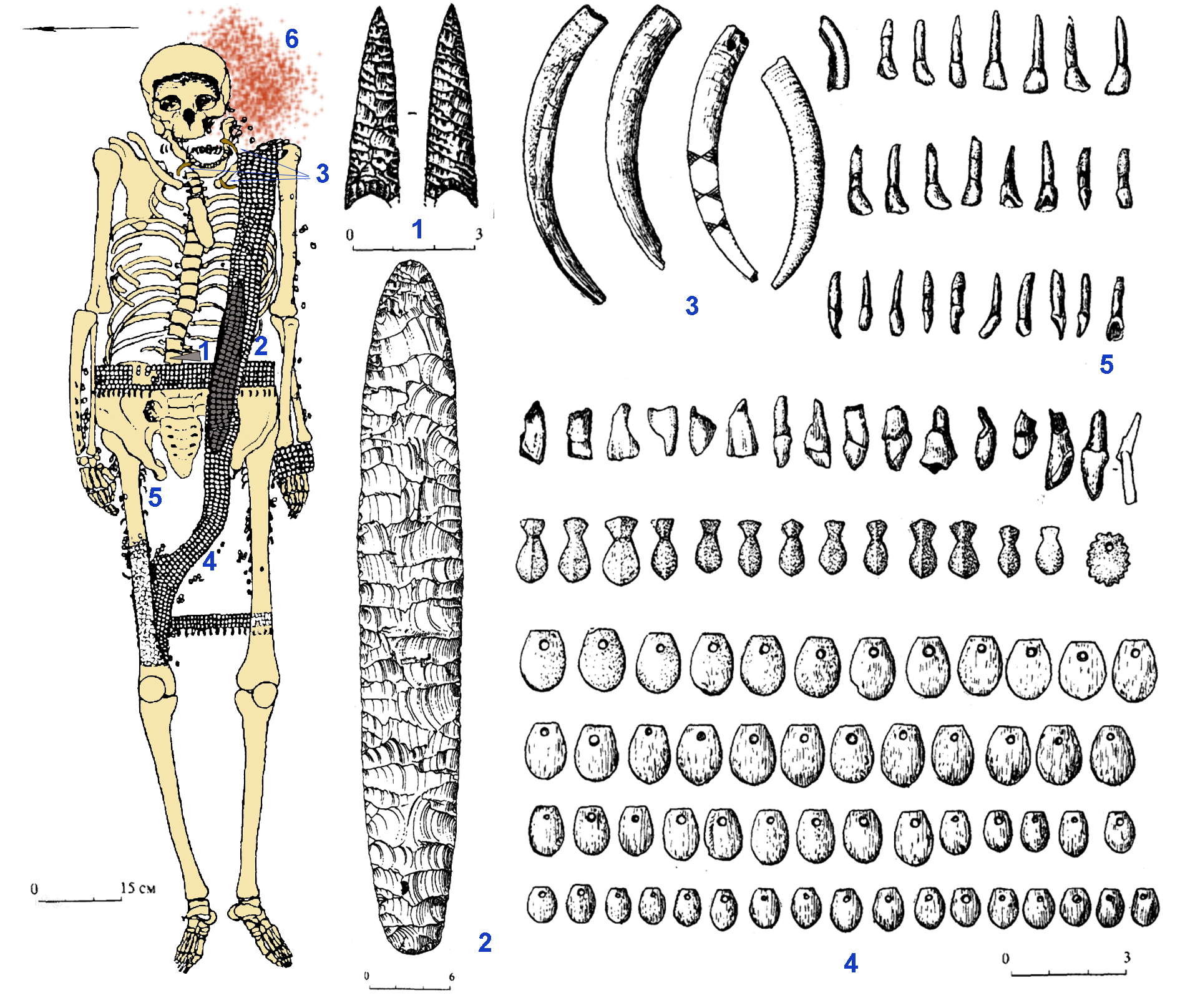


***Figure S33.*** *Burial 7 at Solontsy-5.* ***1*** *- arrowhead;* ***2*** *- bifacial dagger;* ***3*** *- musk deer tusks;* ***4*** *- appliques;* ***5*** *- animal teeth;* ***6*** *- ocher spot (by Kungurova 2005, modified).*

##### Tuzovskiye Bugry-1 Late Neolithic-Eneolithic site

The Tuzovskie Bugry-1 burial ground, also known as Vasino-5, was discovered in 1999 by D.A. Pugachev and subsequently investigated in 2000 by M.T. Abdulganeev. The site is situated on the right bank of the Ob River, characterized by a wide floodplain (up to 12 km) with swampy areas and numerous oxbow lakes. These features are interspersed with crests and hillocks that emerge from the bedrock bank. The surrounding vegetation consists of meadows with dense thickets of shrubby willows, aspens, and birches. Excavations at the site covered an area of 174 square meters, revealing a total of 37 burials. Among these, 19 can be attributed to the Eneolithic and Early Bronze Age periods. Unfortunately, some of these graves were destroyed due to the construction of an early Iron Age necropolis. The Eneolithic burials were arranged in four rows oriented along a southeast-northwest axis, each row containing 5-6 burials. Most of the deceased individuals were found in an extended supine position, with their arms lying alongside their bodies and their heads oriented towards the northeast. The majority of the burials were single, while three were paired and two were collective, containing three and five individuals, respectively.

Thus far, 12 radiocarbon AMS dates have been obtained, with 11 derived from human bones and teeth, and one from a mountain goat tooth. These results have raised the issue of a reservoir effect, which needs further investigation. Noteworthy chronological indicators in the archaeological context include decorations crafted from mollusk shells (Dentalium and Corbicula) as well as animal teeth (such as musk deer, mountain goat, or ram). Based on the available evidence, the Eneolithic burials can be dated to 3600-3200 calBCE.

One specific burial, grave 34, can be attributed to the Early Bronze Age. Burials 4 and 18 lacked any accompanying inventory, likely due to disturbance during the construction of Early Iron Age graves. Notable artifacts found in these contexts include decorations made of mollusk shells (Dentalium and Corbicula), which were imported (Kiryushin et all, 2011), and animal teeth (musk deer, mountain goat, or ram) (Kiryushin et all, 2012). Burial 7 yielded pottery of Bolshoy Mys and Comb-Pit pottery types within its fill (Kiryushin and Kiryushin, 2015).


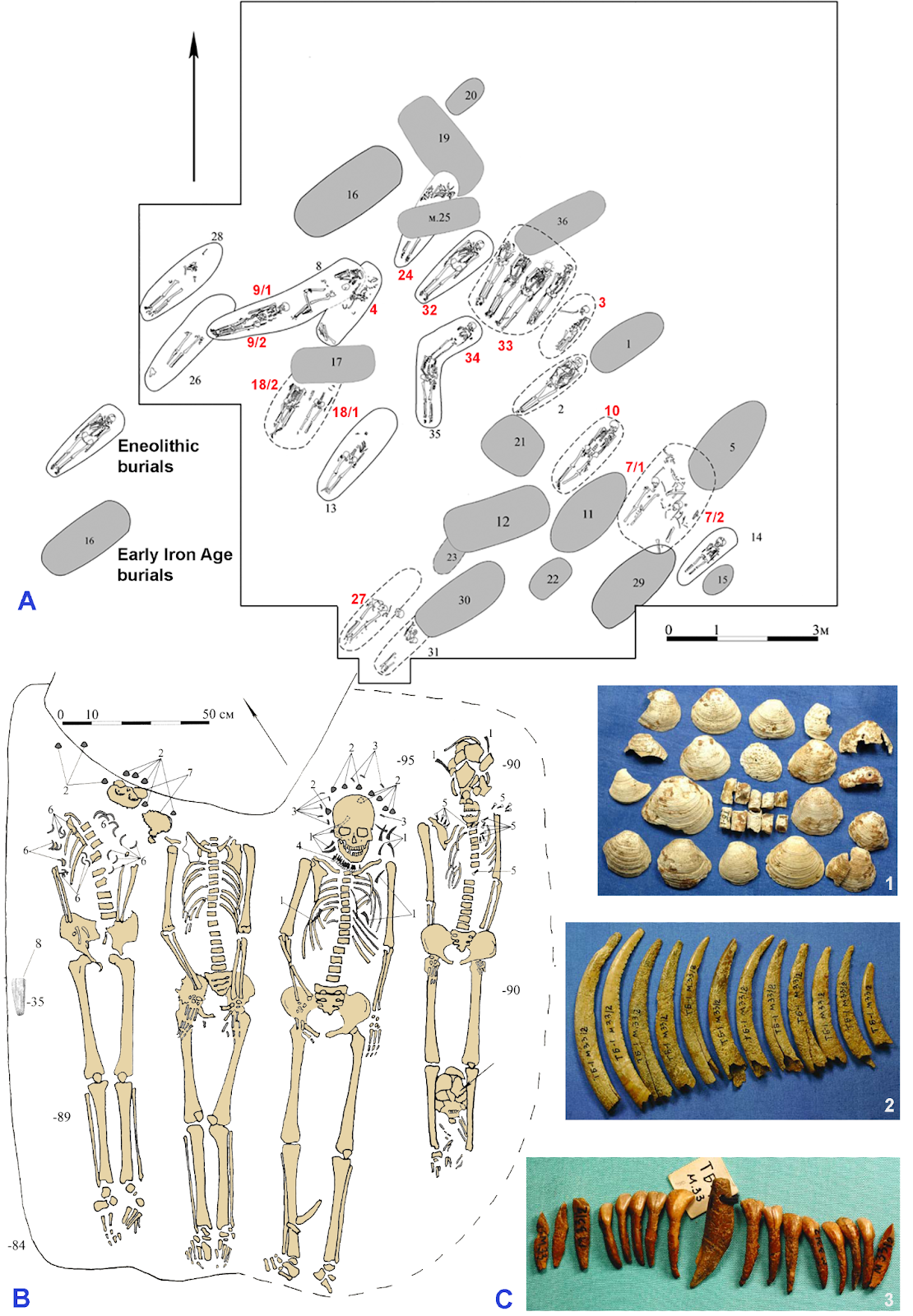


***Figure S34.*** *Tuzovskiye Bugry-1 burial ground.* ***A*** *- a plan of the cemetery, sequenced individuals are labeled in red;* ***B*** *- burial 33:* ***1*** *- tusks of musk deer,* ***2*** *- Corbicula shells,* ***3*** *- Dentalium shells,* ***4*** *- necklace with 18 small animal incisors,* ***5*** *- necklace with 6 small animal incisors,* ***6*** *- necklace with 25 marmot incisors,* ***7*** *- skull fragments;* ***C*** *- grave goods from burial 33:* ***1*** *- mollusk shells,* ***2*** *- tusks of musk deer,* ***3*** *- pendant from the upper fang of a badger and small incisors of a musk deer (image by Kirill Kiryushin).*

###### Burial 3 (individual ID I11747, male)

**Burial 3** was situated adjacent to **burial 33** and marked the southeastern end of the "first" row. It was found at a depth of 0.6 meters from the current surface, and no distinct grave stain was visible. The buried individual, a male aged 35-45 years, was interred in a supine position with the head oriented to the northeast. Some bones were missing, and the remaining ones were disordered. The burial took place long after the individual's death. The grave goods included two Corbicula shells, a horn point, and a pendant made from a deer tooth with a perforation.

###### Burial 4 (individual ID I12323, male)

**Burial 4** was located in the northwestern part of the second "central" row and was partially intersected by **burial 8**. It had a southwest-to-northeast orientation along its long axis, with a depth ranging from 0.45 to 0.6 meters. The precise shape of the grave pit could not be determined. The buried individual was a male aged 40-55 years. Similar to other burials, this one also took place long after death, as it was observed on the position of bones in the grave, and lacked any grave goods.

###### Burial 7, skeletons 1 (individual ID I11750, male) and 2 (I11748, female)

**Burial 7** was situated in the southeastern part of the second "central" row, between **burials 10** and **14**. Its approximate dimensions were 2.15 x 1.4 meters, with the long axis aligned with a southwest-to-northeast line. **Burial 7** had been disturbed by **burials 5** (east corner), **29** (south corner), and possibly **burial 11** (west corner). The burial occurred long after the individuals' deaths. The grave pit contained disarticulated bones of a male aged 30-40 years (***skeleton 1***, individual ID I11750) and a female aged 40-55 years (***skeleton 2***, individual ID I11748), with the anatomical order disrupted. Grave goods included a bone harpoon and fragments of *Colletopterum sp*. Additionally, two AMS dates were obtained based on a fragment of a human radius bone: 3810–3700 calBCE (5005±25 BP, IGAN-5832) and 4450–4030 BCE (5409±93 BP, NSKA-01943). A combination of these two dates yields an age of 3949–3714 calBCE (5034±25 BP) [R_Combine: (5005±25 BP, IGAN-5832); (5409±93 BP, NSKA-01943)].

###### Burial 9, skeletons 1 (individual ID I12628, male) and 2 (I10965, female)

**Burial 9**, along with **burial 8**, was positioned between the second and third rows, cutting through parts of **burials 4** and **26**. It had a west-to-east orientation with a slight deviation to the north. This was a paired burial, with the skeletons of an adult male aged 40-55 years (individual ID I12628) and a female child aged 4-6 years (individual ID I10965) located at the bottom of the grave. Both individuals were found in a supine position, with their heads pointing northeastwards. The adult was missing the left humerus and some ribs, which had fallen to the left due to the construction of the neighboring burial 8. The child's body was positioned atop the adult, with the skull resting on the adult's abdomen and the leg bones positioned between the adult's legs. The grave goods consisted of 15 ornaments made of Dentalium shells. The child skeleton from the grave was dated to 4037–3807 calBCE (5135±25 BP, PSUAMS-8879), and the adult skeleton to 3958–3798 calBCE (5080±25 BP, PSUAMS-9038). A combination of probability densities yields a date of 3970–3804 calBCE (5108±18 BP) [R_Combine: (5135±25 BP, PSUAMS-8879); (5080±25 BP, PSUAMS-9038)].

###### Burial 10 (individual ID I12627, female)

**Burial 10** was situated in the second "central" row, between **burials 2** and **7**. The skeleton of an adult female aged 35-45 years was found at a depth of 0.6 meters from the modern surface. The burial was in an extended supine position, with the head oriented to the northeast. The skull was turned to the left, and the hands were positioned under the pelvis. Grave goods included 105 ornaments made from incisors of a mountain goat or ram. Two AMS dates were obtained: one based on human bones, yielding a result of 4045–3804 calBCE (5146±37 BP, GV-03580), and the other based on a tooth of a mountain goat or ram, yielding a result of 3517–3358 calBCE (4637±37 BP, GV-03579). We expect a freshwater reservoir offset in the date obtained from human bones and attribute the burial based on the latter date.

###### Burial 18, skeleton 1 (individual ID I17582, male) and skeleton 2 (I13260, male)

**Burial 18 (*skeleton 1****:*individual ID I17582;***skeleton 2****:* individual ID I13260) was located in the "third" row between **burials 26** and **13**, and it had been partially disturbed by **burial 17** from the Early Iron Age. The excavated area measured approximately 1.25 x 1 meters, with its long axis aligned with a southwest to northeast line. At the bottom of the grave, at a depth of 0.81 meters, were the incomplete skeletons of three individuals: two adults and one child. One of the buried individuals was determined to be a male aged 25-35 years, while the age of the child was estimated to be 8-12 years. It was challenging to ascertain the sex and age of the third skeleton, but it is likely that it belonged to a female person.

###### Burial 24 (individual ID I17682, female)

**Burial 24** was situated adjacent to **burial 32**, with the northeastern part of **burial 24** disturbed by **burial 19** and the central part destroyed by **burial 25**. The exact dimensions of **burial 24** were challenging to determine. It had an elongated oval shape, with approximate dimensions of 2.05 x 0.6 meters, and extended from southwest to northeast. The depth from the modern surface was 1 m, while it was 0.25 m below the mainland. Two burials were recorded: an adult woman aged 35-45 years (along the southeastern wall) and an infant (to the left of the adult near the northwestern wall). The adult's remains were missing the skull, left arm, radius bones, right hand, lower part of the chest, pelvic bones, both femurs and upper part of the right tibia, which had been severed. The adult was placed in the grave in a supine position with the head oriented to the northeast. The child's body was positioned with the head pointing northeast, likely lying on the left side. Only a crushed skull and part of the chest were preserved from the child's remains. Burial goods included a fragment of a stone tool and 17 shell ornaments (round beads with a central hole) made from *Colletopterum sp*. shells. An AMS date of 3951–3656 calBCE (5016±54 BP, GV-03581) was obtained from human bones.

###### Burial 27 (individual ID I11751, female)

**Burial 27** was located in the "third" row between **burials 13** and **31**. The northeastern part of the grave had been completely destroyed, and its outline was untraceable. The filling of the grave was only observed at the mainland level from a depth of 0.97 meters at the bottom. The fixed part of the grave had dimensions of 1.4 x 0.55 meters and was extended from southwest to northeast. At the bottom, the lower part of an adult female skeleton (aged 30-40 years) was found, including the legs, pelvic bones, hands, and a broken radius of the left hand. Based on the positioning of the remains, the individual was laid in a supine position with the head oriented to the northeast. Burial goods consisted of fragments of a bone item (possibly bow plates), pebbles with fine scratches, a retouched flake, 11 shell fragments of *Colletopterum* sp., and two *Corbicula* shells. Two AMS dates were obtained from human bones: 3969-3661 calBCE (5055±57 BP, GV-03582) and 3946-3655 calBCE (5004±36 BP, UBA-22957).

###### Burial 32 (individual ID I0994)

**Burial 32** was situated in the northwestern part of the "first" row, between **graves 24** and **33**. The filling of **burial 32** consisted of light-gray sandy loam, and its depth from the modern surface was 0.7 meters, while it was 0.1 meters below the mainland. At the bottom, the skeleton of an adult male individual was found in a supine position with the head oriented to the northeast. Grave goods included 15 *Corbicula* shells, with fragments of two additional shells discovered in the grave fill. Additionally, a chisel made of a tusk of a mountain goat or ram and bones from an animal no smaller than a ram were present. An AMS date of 3932–3535 calBCE (4914±56 BP, GV-03583) was obtained on human bones. Another radiocarbon determination dates the burial to 3986–3715 calBCE (5090±50 BP, Poz-83434). A combination of probability densities yields a date of 3946–3659 calBCE (5013±38 BP) [R_Combine: (4914±56 BP, GV-03583); (5090±50 BP, Poz-83434)].

###### Burial 33 (individual IDs I0995, I13100, I13811, identified as males)

**Burial 33** contains the remains of four adults and one child, all buried in an extended supine position with their heads facing northeast. Three of them were sequenced; unfortunately, we could not find data to match the skeletons in **grave 33** to our genetic sequence IDs.

***Skeleton 1*** was positioned along the southeastern wall. The lower part of the ulna and radius bones were covered by the pelvic bones, while the hand bones were placed on the femurs. The skull is fragmented. Between the tibia bones, the skeleton of a baby was found (*skeleton 5*). Adjacent to the adult's skull, two large curved fangs of an animal were discovered. Additionally, a necklace made of 16 small incisors of the same animal was found around the shoulder and neck area, as well as on the upper ribs. The skeleton was dated to 3626–3198 calBCE (4649±54 BP, GV-03584).

***Skeleton 2*** (located near the northwest wall) was positioned close to the center of the grave. The bones of the left hand were extended along the skeleton, while the bones of the right hand were placed on the pelvis. Ten piercings of spadefoot mollusk shells were found around and beneath the skull, along with three fragments and nine intact shells of bivalve mollusks. Furthermore, ten large curved fangs of an animal were present, along with two additional fangs located on both sides of the spine, near the lower ribs. Eighteen small incisors of the same animal were found predominantly near the cervical vertebrae, with some located on the right side. The skeleton was dated to 3939–3634 calBCE (4937±56 BP, GV-03585).

***Skeletons 3 and 4***, situated near the northwest wall of the grave, were damaged during the construction of burial 36. In the case of *skeleton 3*, the bones of the hands were found on the pelvis, while the skull was missing. *Skeleton 4* was positioned along the northwestern wall, with the skull and humerus absent. The upper part of the chest and spine had shifted toward the northwest. Similar to skeleton 3, the bones of the hands were on the pelvis. Among the ribs, 25 small incisors of the same animal were discovered.

Above the bottom of the grave (at a depth of 0.5 m from the soil surface), fragments of a skull were found, accompanied by seven shells. Additional skull fragments were uncovered at the southwestern wall of the grave, at a depth of 0.25 m, and a broken abrasive tool was found at a depth of 0.35 m.

The sequenced individual ID I0995 was dated to 4220–3801 calBCE (5170±50 BP, Poz-83435), the individual ID I13100 was dated to 3947–3713 calBCE (5030±25 BP, PSUAMS-9043) (both dates are on human bones/teeth).

For general dating of **burial 33**, we prefer to use the date from the human bones of ***skeleton 1*** (3626–3198 calBCE (4649±54 BP, GV-03584)) as the only one corresponding to the dates from the animal bones found in **grave 10** (3517–3358 calBCE (4637±37 BP, GV-03579)). Other radiocarbon dates were pprobably affected by the freshwater reservoir offset. The high level of fish consumption in the Tuzovskiye Bugry Eneolithic population is supported by stable isotopic values.

###### Burial 34 (individual ID I13099, female)

**Burial 34** was situated in the central part of the second "central" row, between **burials 4** and **2**. The grave pit had an approximately rectangular shape, with dimensions of 1.3 x 0.6 m extended from southwest to northeast. The southwestern edge of **burial 34** intersected with the northern edge of **burial 35**. The filling of **burial 34** consisted of light-gray sandy loam, and its depth from the modern surface was 1.09 meters, while it was 14 centimeters below the mainland. At the bottom of the grave, the skeleton of an 8-10 year old female child was found in a supine position with the head oriented to the northeast. The skull was slightly turned to the left, and the left arm extended along the body, while the right arm was bent at the elbow, indicating that the missing hand should have been positioned on the shoulder. The feet were placed where the skull of the individual buried in **burial 35** would have been. Grave goods included lead earrings and two flat-bottomed vessels.

###### Unidentified burial (individual ID I11405, female)

The list of sequenced individuals from Tuzovskiye Bugry-1 includes individual ID I11405, which was associated with **burial 5** based on its skeletal code. However, archaeological attribution places **burial 5** in the Early Iron Age, while individual I11405 shows close kinship with Eneolithic individuals buried at Tuzovskiye Bugry-1. In light of this discrepancy, we propose that a mislabeling may have occurred, and we consider individual I11405 as originating from an Eneolithic-period burial in a grave with an unidentified number.

#### Upper Ob Eneolithic Sites (Russia_UpperObKiprino_Eneolithic)

##### Razdum’ye-1 hillfort

The Razdum'ye-1 site was described above (Supplementary Information section 3.3.2.1.1)).

###### Burial 7 (individual ID I13678, male)

**Burial 7** represents an irregular oval pit with an extension in the southwestern half. Its dimensions are approximately 1.55x0.75-1.20 m, and it has a depth of 1.35 m from the level of the modern surface. The grave is oriented along a north-northeast to south-southwest line and likely experienced damage in ancient times.

Near the center of the grave, at the bottom, a skull and a tooth belonging to a male aged 40-45 years were discovered near the western wall. In the northeastern part of the grave, close to the wall, the remains of a pillar have been preserved. The cranium exhibits a pale pink coloration.

Behind the cranium, in a pile, various artifacts were found, including a bone harpoon, two bone retouchers, a grinding plate, fragments of a river mollusk shell, bone plates, small animal bones, and stones. Among the stones, a stone sculpture was discovered. Furthermore, a rough arrowhead made of quartzite (?) with a triangular-shaped tang outline was found. Towards the southwestern end of the grave, two grinding plates, three stones, three fragments of a bone plate, a roe deer horn, and a shard of a thick-walled vessel with rough craftsmanship were uncovered. The vessel shard showed traces of surface smoothing using a notched spatula or a bundle of dry grass (Umanskiy 1987). The skeleton is dated to 3361–3102 calBCE (4525В±20 BP, PSUAMS-9045).


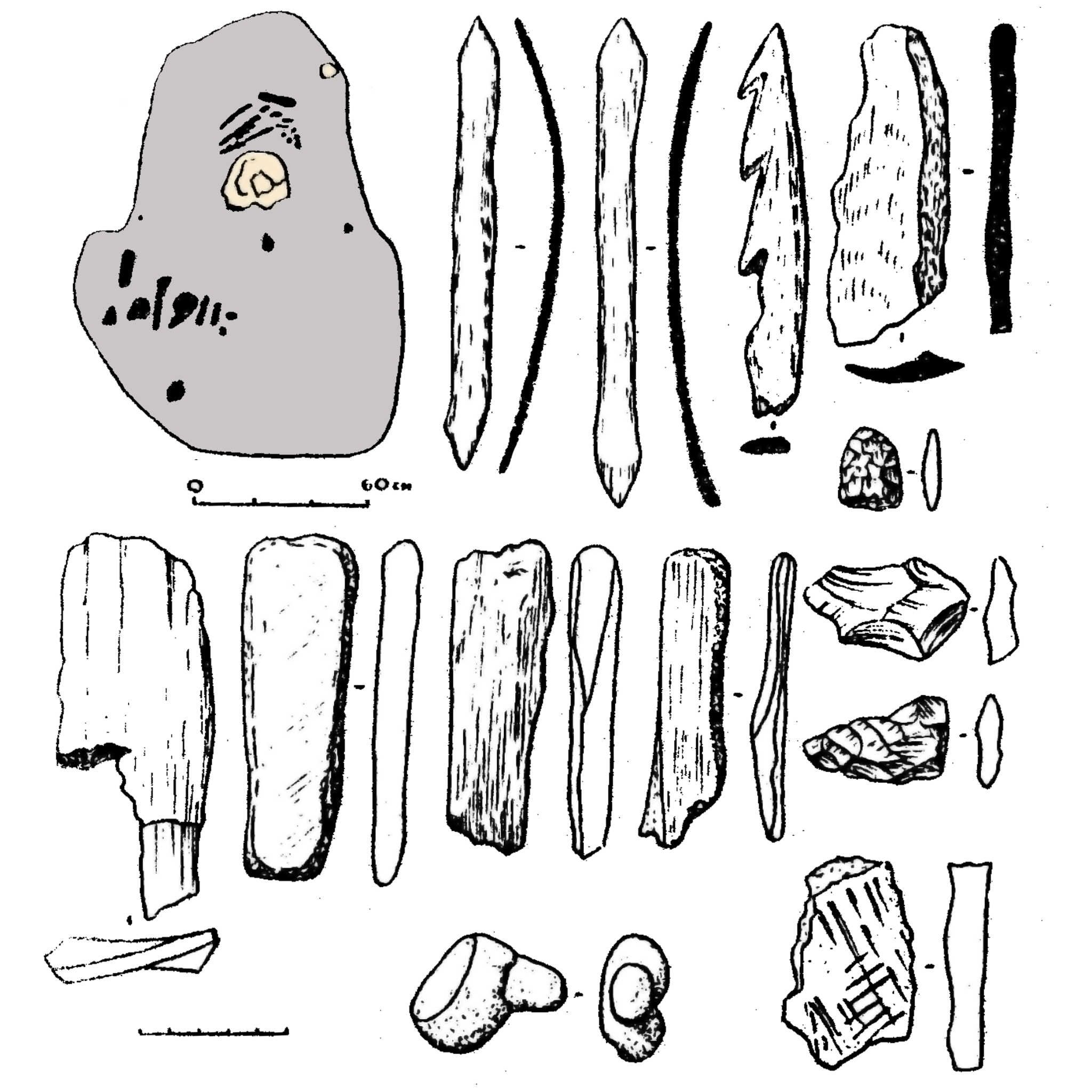


***Figure S35.*** *Burial 7 at the Razdumye-1 site (by Umanskiy 1987, modified).*

##### Ordynzkoye-1 burial site

The Ordynskoye-1 burial ground is located on the left bank of the Ob River valley, now the Ob reservoir, on a cape-shaped ledge of the terrace, at the entrance to the Ordynsky Bay. The western part of the site was excavated by M.P. Gryaznov in 1953–1954, and the eastern part was excavated by T.N. Troitskaya in 1973–1977.

Most of the burials belong to the Late Krotovo and Irmen traditions. At least three burials were identified by T.N. Troitskaya and V.I. Molodin as an earlier phase of the burial ground (Troitskaya 1973) and classified as the Kiprino stage of the Upper Ob culture (Molodin 1977), attributed by modern researchers to the Eneolithic (Kiryushin 2008).

###### Mound 11, burial 4 (individual ID I11002, female)

**Burial 4** under **mound 11** was partially destroyed as a result of coastal erosion. The depth of the grave was 25 cm. The skeleton lay on its left side, with its head oriented to the northeast. Burial goods are represented by a polished stone plate. The burial was attributed to the Kiprino culture on the basis of the find of similar plates in other early burials on the site, which also contained Kiprino pottery (Troitskaya 1973). The date of the skeleton is 2907–2704 calBCE (4235±25 BP, PSUAMS-9034).

### Middle Irtysh and Baraba forest-steppe

#### Middle Irtysh Neolithic culture (Russia_MiddleIrtyshNeolithic_LN)

##### Omsk Neolithic occupation

The archaeological complex known as the "Omsk Site" is situated in the former floodplain on the left bank of the Irtysh River within the city of Omsk. The estimated area of this complex spans at least 12 hectares. Excavations conducted at the site have revealed layers dating from the Paleolithic to the Middle Ages, representing different phases of occupation. Archaeological investigations have been ongoing at this site for over a century. During rescue excavations in 1988-89, Boris Konikov discovered a Neolithic burial ground within the Omsk site. Five burials were unearthed at depths exceeding one meter from the contemporary surface. Three of these burials had experienced significant disturbance, while the other two remained undisturbed. The skeletons were positioned in a supine orientation with their heads facing north-northeast. The discovered artifacts exhibited close parallels with Neolithic sites in the Omsk-Irtysh region. Within the cultural layer of the Neolithic settlement at the Omsk site, a well-preserved dwelling pit was identified. Additionally, a substantial collection of stone tools, including cores, blades, and scrapers, was attributed to the Neolithic period (Metel, 2016; Konikov, 2016).

###### Burial 2 (individual ID I1959, male)

The female **burial 2** was made in a supine position. Grave goods are represented by two drilled wolverine fangs, two retouched plates, and a scraper on a flake (Konikov, 2016). The burial is dated to 5612–5381 calBCE (6530±40 BP, Poz-82200).


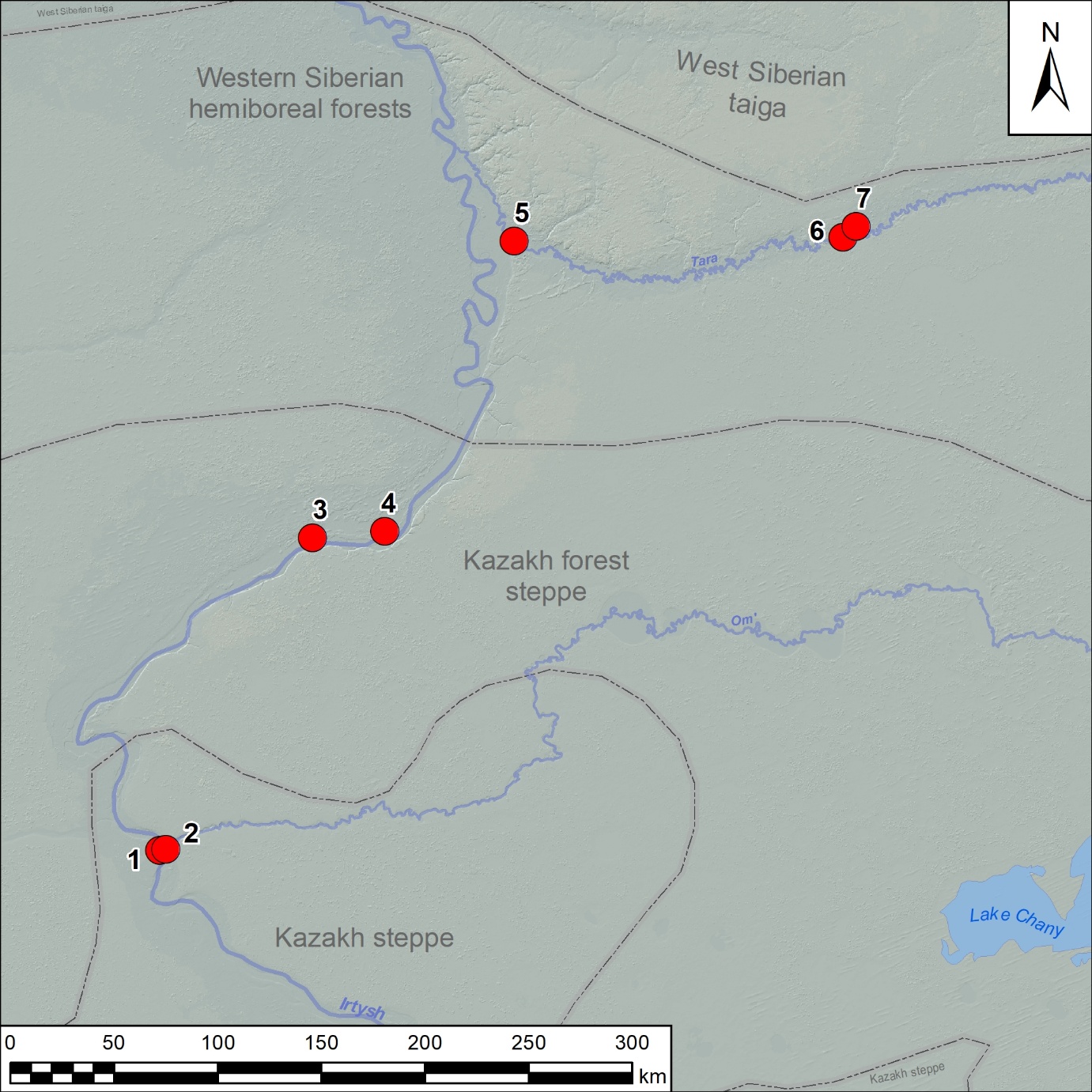


***Figure S36.*** *Neolithic sites in the Middle Irtysh region.* *1 - Omsk; 2 - Ostrov-2; 3 - Chernoozerye-1; 4 - Borovyanka-17; 5 - Okunevo; 6 - Korchugan-1; 7 - Protoka.*

##### Korchugan-1 site

The Korchugan-1 archaeological complex comprises a series of multi-period settlement and burial sites, encompassing five distinct phases. These include two burial grounds, namely Korchugan-1a and Korchugan-1b, as well as three settlement complexes known as Korchugan-1c, Korchugan-1d, and Korchugan-1e. Among these components, the Korchugan-1a burial ground is situated along the shores of an oxbow of the Kryuchnoye lake, on the elevated Korchugan terrace remnant. This remnant is characterized by steep banks, rising 2 to 5 meters above the floodplain.

Specifically, Korchugan-1a is a Neolithic soil burial ground, located approximately 8.5 meters from the northern edge of the terrace and 3.5 meters from the western edge. The necropolis consists of three burials, designated as burials 2, 3, and 7, which are arranged in a linear fashion along a northwest-southeast line. In terms of its spatial arrangement, the Korchugan-1a burial ground holds a central position within the broader complex of the site (Marchenko, 2009).


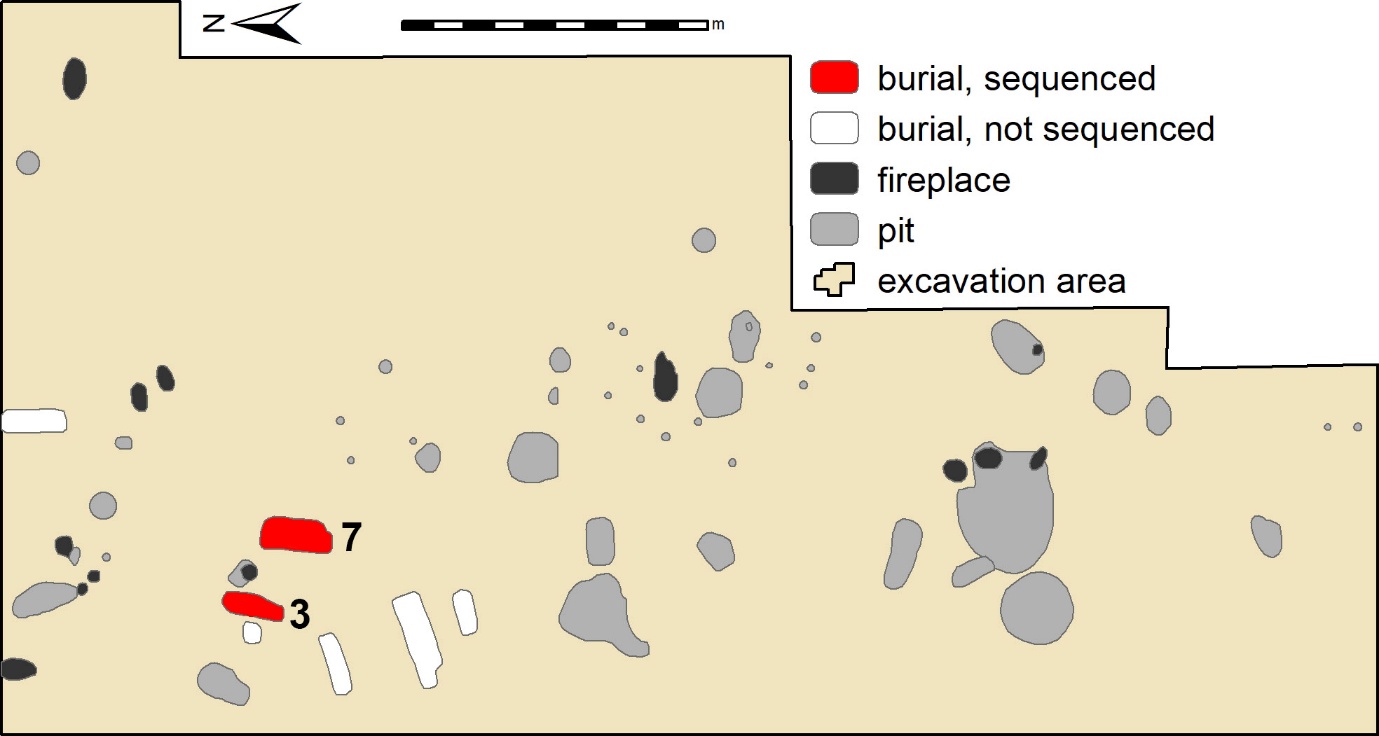


***Figure S37.*** *The Korchugan-1a cemetery (based on Marchenko, 2009).*

###### Burial 3 (individual ID I0991, male)

The grave pit of **burial 3** has an oval shape, measuring 2.2 x 0.75 x 0.5-0.17 meters, extended in the NNE-SSW direction. The walls of the pit are vertical, and the bottom has a complex shape. Consequently, the deceased individual was positioned in the grave with the upper body raised. The body was extended on the back, with the head facing NNE. The right arm of the deceased was slightly bent at the elbow, with the hand placed in the pelvic area, while the left arm was positioned under the pelvic bones. A stone axe was placed beneath the skull, and a small stone flake was found near the right hip joint (Marchenko, 2009). The burial is dated to 5206–4799 calBCE (6060±50 BP, Poz-83427).


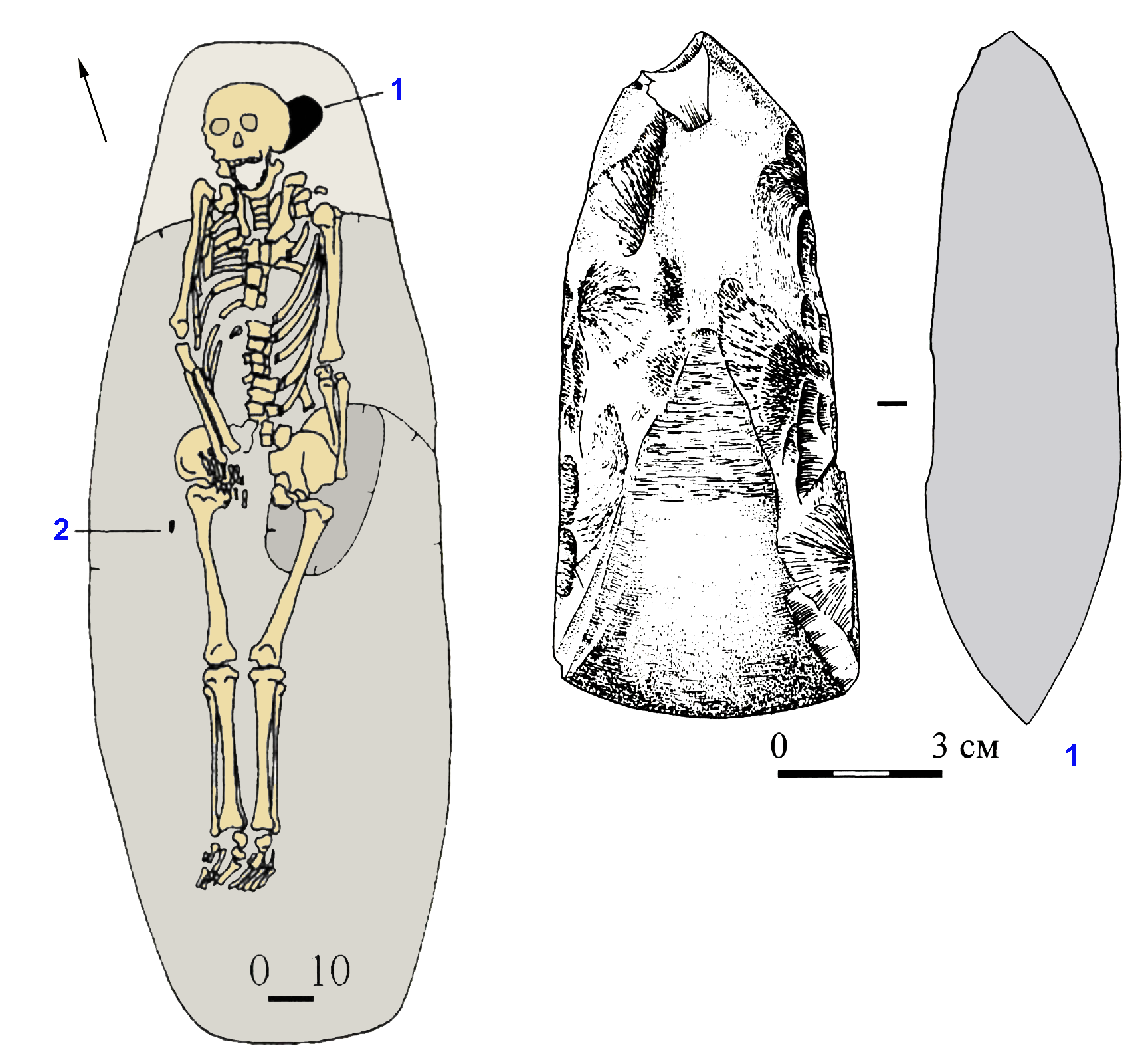


***Figure S38.*** *Burial 3 at the Korchugan-1a burial ground.* ***1*** *- stone axe;* ***2*** *- flake* *(by Molodin et al., 1999, modified).*

###### Burial 7 (individual ID I0992, female)

The grave pit of **burial 7** has an irregular shape, measuring 2.5-1.75 x 0.52 x 0.5-0.4 meters, and is extended in the north-south direction. The body was extended and the head faced north. Due to the uneven bottom of the pit, the upper part of the body and the head were elevated compared to the legs. The bones of the hands lay on the pelvic bones. Notably, pendants made of rodent incisors, possibly from squirrels, were discovered near the right knee. In addition, a necklace consisting of nine miniature bone figurines of birds, crafted in a flat style, was found in the chest area. One of the pendants in the necklace had a drop-shaped bone pendant with a hanging hole, making it the tenth component of the necklace (Marchenko, 2009). The burial is dated to 5002–4727 calBCE (5990±50 BP, Poz-83428).


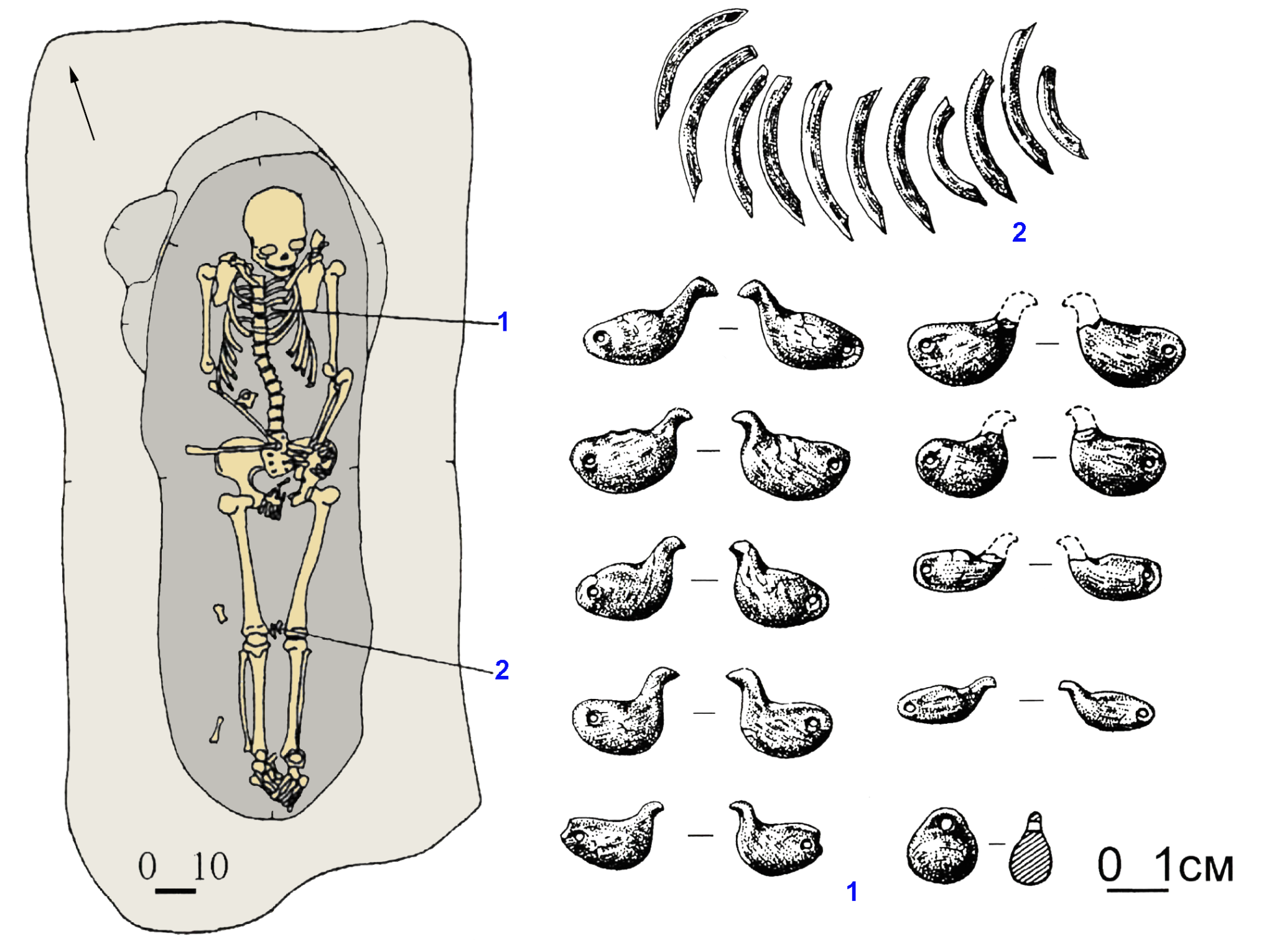


***Figure S39.*** *Burial 7 at the Korchugan-1a burial ground.* ***1*** *- necklace;* ***2*** *- rodent incisors (by Molodin et al., 1999, modified).*

##### Protoka site

The Protoka burial ground was initially discovered by Viktor Zakh in 1980. Situated on the northwestern periphery of the Baraba forest-steppe, it encompasses nine burial mounds positioned along the periphery of the Kryuchnoe lake terrace, an oxbow lake formed by the Tara River. In 1986, Natalya Polosmak conducted excavations at the site, focusing on five burial mounds originating from the Bronze and Early Iron Ages. It was during the excavation of mound 5 that Neolithic burials were unexpectedly unearthed.

The burials at Protoka were classified as secondary, as the bones were largely found in disarray, although some elements retained their anatomical position. The spatial arrangement and characteristics of the bone accumulations indicate that the deceased bodies were likely buried after a significant period of decomposition. In total, 14 burials were identified, containing the remains of 26 individuals, including men, women, and children (Polos’mak et al., 1989).

###### Mound 5, burial 3, individual B (individual ID I7616, male)

The burial includes remains of three individuals arranged side-by-side: two adults and a child aged approximately 7.5-8 years.

The upper portion of ***skeleton B*** (individual ID I7616) was preserved in anatomical order down to the pelvic bones. The interred individual, identified as a male between 40-45 years old, was positioned supine with an extended body posture, and the head oriented towards the north-northwest. Adjacent to the right hand of ***skeleton B***, the skeletal remains of a child (***skeleton C***) were discovered. Notably, fragments of the child's skull (***C***) were found resting on the left clavicle of ***skeleton B***. Additionally, a polished stone chisel was discovered in close proximity to these remains. Roughly 20 cm away from skull B, a damaged skull (***skeleton A***) belonging to a male aged 22-26 years was found.

Within the burial context, four pendants crafted from stone and bone were unearthed. These pendants were originally sewn onto clothing and were discovered in the vicinity of the chest area of ***skeleton B***. A whetstone, a fragment of a vessel, and two bone arrowheads were positioned near the waist level of the same individual's spine. Furthermore, a fragment of pottery was found in close proximity to skull A (Polos’mak et al., 1989).

The burial has been dated to 4446–4349 calBCE (5555±20 BP, PSUAMS-4856), based on a radiocarbon date from skeleton B.


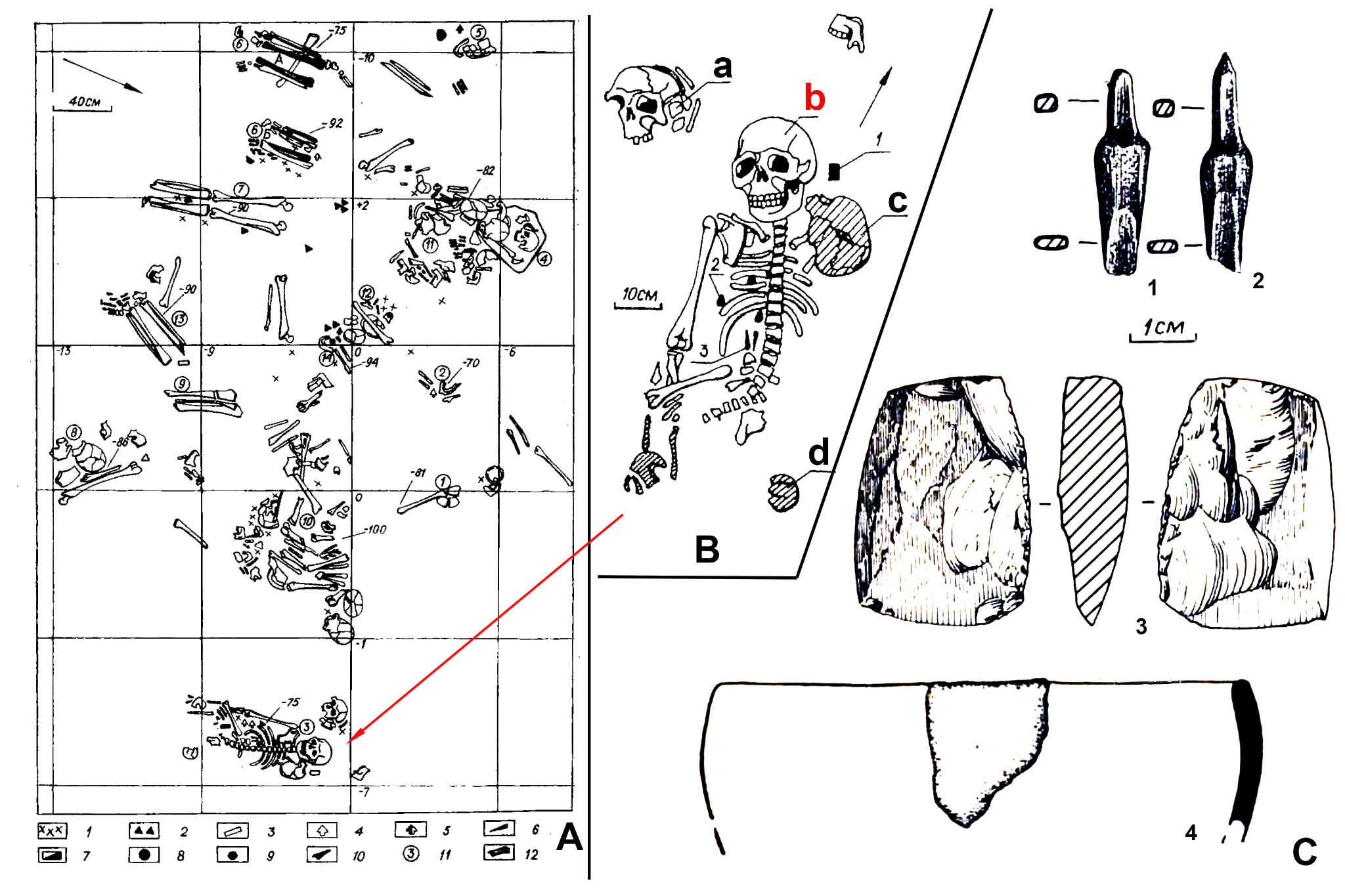


***Figure S40.*** *The Protoka cemetery.* ***A*** *- Neolithic burials covered by mound 5 (****1*** *- ceramics;* ***2*** *- pendants;* ***3*** *- adze;* ***4*** *- arrowhead (bone);* ***5*** *- arrowhead (stone);* ***7*** *- abrasive;* ***8*** *- core,* ***9*** *- vessel,* ***10*** *- a jaw of a dog,* ***11*** *- burial number;* ***12*** *- bones of the lower horizon;* ***a*** *- a vessel;* ***b*** *- knife-like plate;* ***h*** *- core);* ***B*** *- burial 3 (****1 -*** *chisel;* ***2*** *- pendants;* ***3*** *- arrowheads (bone); bones of the child's skeleton are cross-hatched);* ***C*** *- grave goods from burial 3 (****1,2*** *- arrowheads;* ***3*** *- chisel;* ***4*** *- pottery sherd) (by Polos’mak et al., 1989, modified).*

#### Eneolithic sites with Comb-Pit Ware (Russia_CombPitWare_Eneolithic)

##### Eneolithic phase of the Borovyanka-17 site

The Borovyanka-17 burial ground was discovered in 1999, revealing burials spanning various archaeological epochs from the Eneolithic to the Middle Ages. Some of the materials from early burials have been previously published (Khvostov, 2000; 2001). Through archaeological investigations, over twenty burials and pits dating back to the Eneolithic period have been unearthed at the burial ground. The remarkably well-preserved state of the skeletons in most burials allows for a clear identification of the primary burial practices of the Eneolithic population in the forest-steppe zone of the Middle Irtysh region.

Single burials are the most common, while paired and collective burials occur less frequently. The number of individuals interred in collective burials ranges from three to eight. The deceased were typically laid on their backs, often with bent legs, and their heads were oriented towards the northeast, occasionally facing north (burials 81, 82). A notable characteristic of the funeral rite is the use of ocher to fill the burial or to surround the deceased. In some instances, sizable pieces of ocher were placed near the head of the buried individual (burial 83). The majority of the deceased were buried through inhumation, while evidence of fire in funeral rites is less common, such as partial burning of the skeleton (burial 36), charred planks over the burial entrance pit (burial 83), or embers in the grave filling. The presence of light ash bedding in several burials (burials 12, 19, 36, 46) likely indicates its role in the fire cult. Cremation was observed only in one case (burial 84).

Another distinctive feature of the burial rite is the intentional disturbance of anatomical integrity. For example, in burial 64, containing a double burial, the skulls of the buried children were separated. In tiered burial 36, the tibia bones of the lower skeleton were cut off along with the foot bones and placed on top of the femurs, while in burial 46, the skull was missing. Burial 4 likely contained a special hole for burying the skull adjacent to the skeleton. Additionally, several instances of secondary burials were recorded, where the deceased were dismembered and placed within some form of container before interment. There are also burials of skulls, sometimes arranged in groups (burial 83).

Several burials (burials 42, 45, 67, 72) contained pits filled with fish scales, bones, and gills. The association of these pits with funeral rites is indisputable: in collective burial 72, such a hole was located between the heads of the buried individuals, while in burial 42, it was found near the feet. In burial 67, scattered human bones were discovered alongside intact fish skeletons.

The burial goods found within the burials vary in their abundance, ranging from richly furnished burials (burials 14, 36) to interments with no burial goods. Deliberate destruction of items is a characteristic practice. Miniature ceramic vessels were recovered from two burials (burials 12, 66).

For the dating of the Eneolithic phase of the Borovyanka-17 cemetery, we use seven radiocarbon dates, obtained from human bones. Based on them and taking into account the δ15C/δ15N ratios observed in the samples from the site, we suggest two sub-phases in the Borovyanka-17 Eneolithic cemetery, the earlier one, dated to 3700–3300 calBCE, and the later one, which is attributed to 2900–2800 calBCE.

###### Burial 4 (individual ID I6962, female)

**Burial 4** was identified as an oval spot measuring 220x90 cm, characterized by gray-brown sandy soil and elongated in the southwest-northeast direction. Another dark gray sandy loam area was observed on the south side (**burial 15**). Excavation revealed a burial pit with elongated proportions and rounded corners, measuring approximately 230x95-100 cm, oriented in the NNE-SSW direction. The northeastern part of the burial had been disturbed by a badger's hole, resulting in the destruction of the upper portion of the skeleton. The southwestern and central parts of the burial had a flat bottom and sheer walls, with a depth of 20-22 cm from the subsoil level (43-45 cm from the present surface). The northeastern part of the burial was deeper, with a hole depth reaching 40-44 cm from the subsoil level (60-65 cm from the present surface). The lower part of the skeleton remained undisturbed, and the bones were well-preserved. The deceased individual was buried in a supine position with bent legs, and the head was oriented towards the northeast. The leg bones were shifted to the left of the spinal line. The left arm extended alongside the body, while the right arm was slightly bent at the elbow, with the hands positioned at the pelvis. The leg bones exhibited slight staining with ocher, and ocher also covered the bottom of the grave pit. The skull had been displaced and was found within the fill of the badger hole, approximately 10-15 cm north of the grave pit boundary.

The grave goods discovered in this burial consisted of 56 well-preserved bone drop-shaped pendants. All the pendants were concentrated in the groin area of the deceased, with the highest concentration observed between the femurs and within the pelvic cavity.

The skeleton is dated to 4045–3819 calBCE (5160±25 BP, PSUAMS-4841).


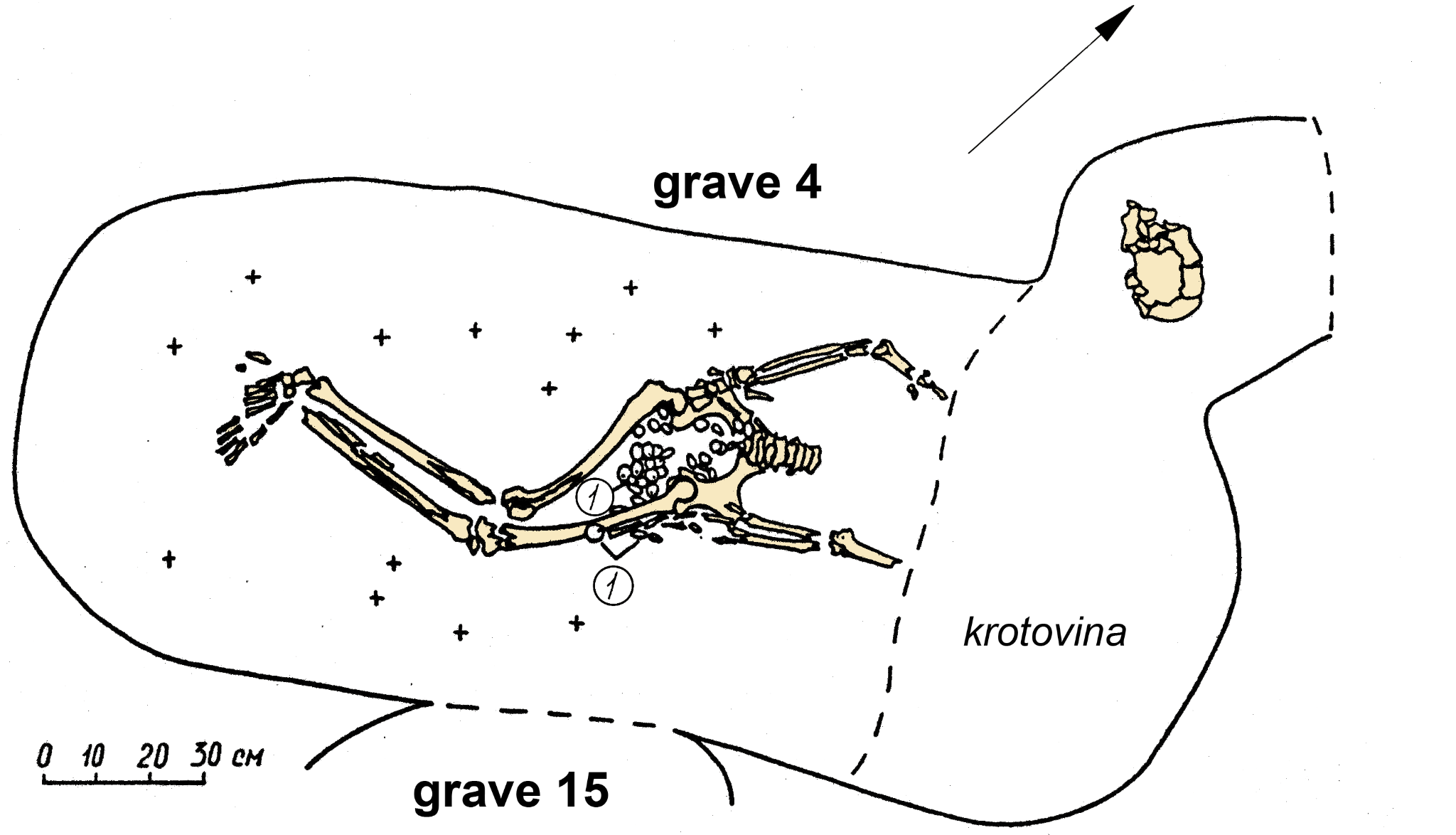


***Figure S41.*** *Burial 4 at Borovyanka-17 (image by Olga Sherstobitova).*

###### Burials 36C (individual ID I6963, male) and 42 (I6831 & I6999, males)

**Burials 42** and **36C** were initially identified as a dark-gray amorphous sandy loam area at the subsoil level. Within the infill, two rims of Comb-Pit pottery, three flakes, and a stone scraper were discovered. The large grave pit, measuring up to 465x185-190 cm, was divided into two parts: the northeastern section (**burial 36**) and the southwestern section (**burial 42**).

**Burial 36C** (individual ID I6963): Upon the complete excavation of **burial 36**, it became evident that traces of light-gray ash sandy loam were present at the bottom of the grave. Further excavation of this layer revealed the presence of another skeleton (**burial 36C**). As a result, **burial 36** was identified as a tiered burial.

The skeleton in **burial 36C** was situated in a pit measuring 225x73-78 cm, aligned along the NNE-SSW axis, and covered by a thick layer of light ash sandy loam. The skeleton was well-preserved and positioned on its back, with the head facing north-northeast. The tibias were intentionally separated and placed alongside the femur. Based on the vertical positioning of the shoulder blades, it appears that the deceased was tightly wrapped or bound. The leg bones, arm bones, and skull showed signs of burning. A significant number of bone beads (several hundred pieces) were discovered in the chest and belt areas. The largest accumulation of beads was found in the chest region. Additionally, petal-shaped bone pendants (six specimens) were located to the right of the pelvis (near the right femur). Two bone beads were found between the femurs and under the pelvis. Pendants made of hare incisors were also present.

After thorough cleaning of the tiered burial, the depth of the grave pit in the subsoil was determined to be 84-88 cm (116-120 cm from the surface).

The skeleton in **burial 36C** is dated to 4320–4052 calBCE (5340±30 BP, PSUAMS-3929).


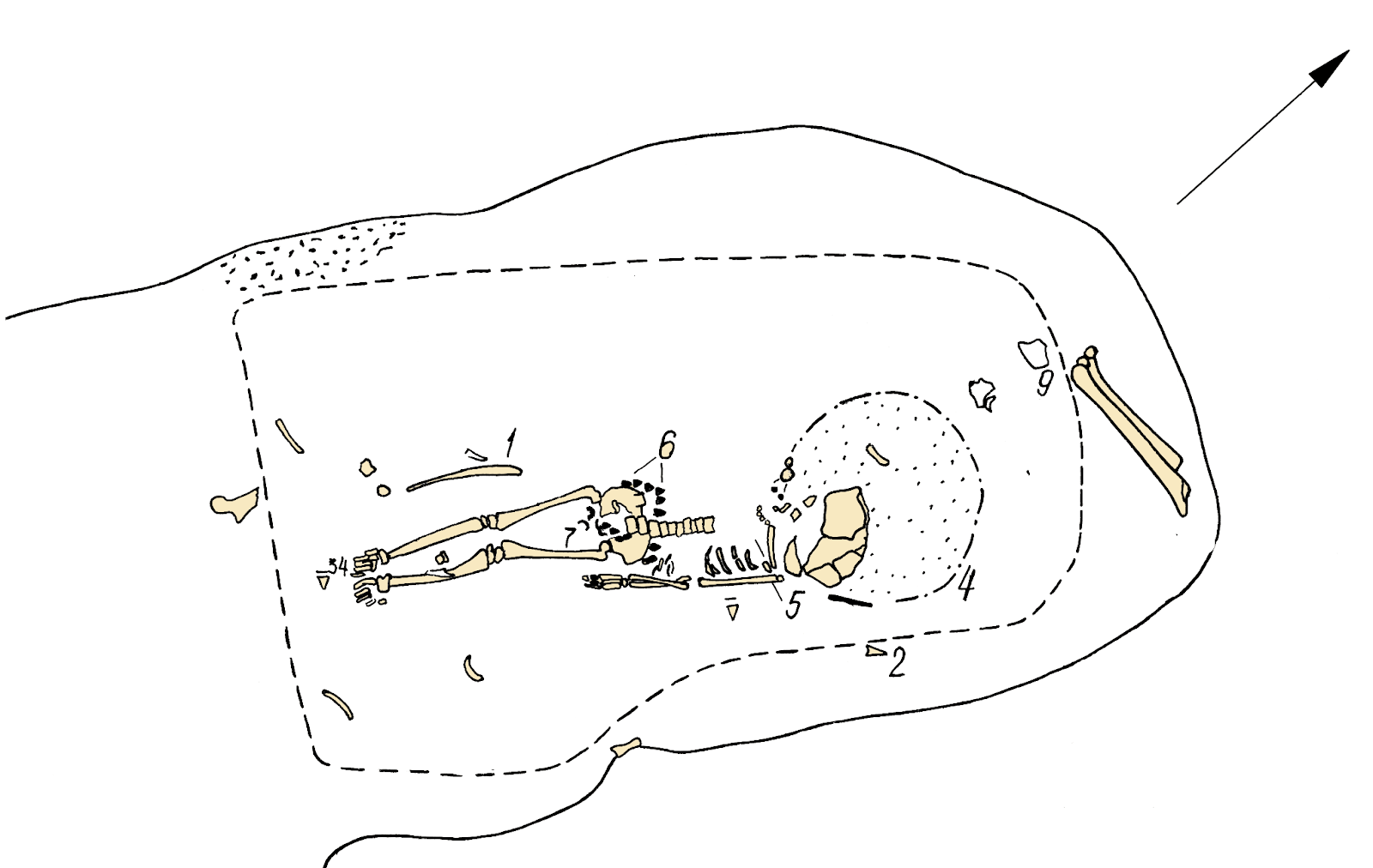


***Figure S42.*** *Burial 36C at Borovyanka-17 (image by Olga Sherstobitova).*

**Burial 42** (individual IDs I6831 and I6999). The amorphous **grave pit 42** extended along the NE-SW axis, measuring 270x160 (190) cm. Its walls exhibited sheer profiles, and the bottom gently sloped northeastwards. The pit's depth from the surface ranged from 47 to 53 cm. Unfortunately, the skeletal remains of the two interred individuals (A and B) suffered severe damage due to badger burrowing and plowing. The southwestern part of the grave was destroyed by **burial 62**, which is attributed to the Late Krotovo culture based on details of burial rites and presence of bronze artifacts.

***Skeleton A*** from **grave 42** was situated in the northwestern area of the pit. The preserved elements included the skull and upper chest components (collarbone, portions of ribs, and humerus). Based on the extant skeletal fragments, the deceased was interred supine, with the head oriented to the northeast. Notably, a slate arrowhead with a notch (1) was uncovered 20 cm southeast from the buried individual's skull. An animal bone was also discovered 10 cm northwest from the right humerus (3).

Adjacent to the southeastern wall of the grave, the remains of ***skeleton B*** were encountered, albeit highly deteriorated. Solely the pelvic bones, fragments of tubular bones, and small, unidentifiable bones were preserved. Some of these bones lay on the surface or slightly above its level. The positioning of the pelvic bones suggests that the deceased was laid to rest supine, with the head directed to the northeast. No accompanying artifacts were found in association with this individual. Notably, a hole measuring 30x15 cm was observed 20 cm northwest of the pelvis, containing fish scales within a layer approximately 10-15 cm thick.

One of the two skeletons from grave 42 was sequenced (individual ID I6831) and dated to 3985–3804 calBCE (5125±25 BP, PSUAMS-4829).

Another sequenced individual (individual ID I6999, petrous bone), according to its label, comes from **burial 62**, which cuts **burial 42** and contains Late Krotovo artifacts. However, the radiocarbon date of individual I6999 (on human bone), 3620–3375 calBCE (4710±20 BP, PSUAMS-4340), makes impossible its Late Krotovo attribution. We assume that this individual originally came from the Eneolithic **burial 42** and was displaced to a later context by pedoturbation processes. Noticeably, this individual demonstrates the lowest value of δ15N and the highest value of δ15C in the analysed dataset, which makes the radiocarbon dates obtained from the human bones of this individual the most reliable.


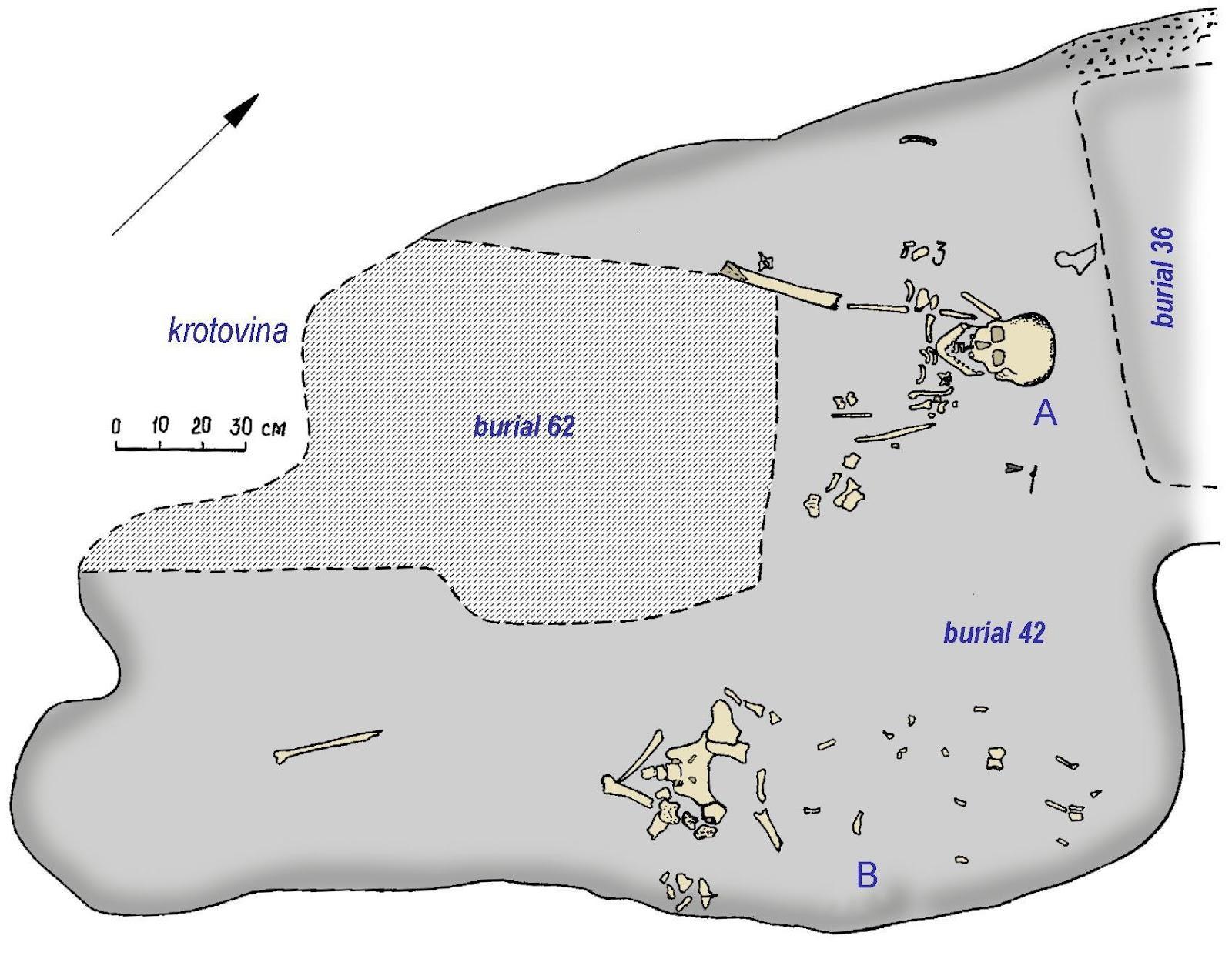


***Figure S43.*** *Burial 42 at Borovyanka-17 (image by Olga Sherstobitova).*

###### Burial 66C (individual ID I6951, male)

**Burial 66** was identified as an oval-shaped spot of dark-gray sandy loam, slightly narrowed in the northeastern part, measuring 220x120-140 cm. It was oriented along the northeast-southwest line. Within the spot's infill, two fragments of Comb-Pit pottery, a sherd of a Chernoozerye-type vessel, and a substantial amount of charcoal were discovered. The grave pit itself had an oval shape, measuring 220x110-135 cm and was oriented along a northeast-southwest line. The depth of the grave pit in the subsoil was 33-37 cm, equivalent to 55-60 cm from the modern surface. The walls were inclined, and the bottom had a slight drop towards the northeast, with a difference of approximately 5-8 cm.

In the western corner of the grave, a dense cluster of human bones was found. Upon further excavation, it was revealed to contain the bones of 7-8 individuals showing signs of deliberate dismemberment, including partially arranged spines and evidence of bone damage. Near the cluster, slightly to the northeast, the remains of two poorly preserved human skulls and a femur were discovered. The sequenced individual ID I6951 comes from ***skull*** labelled ***“C”*** (Cyrillic “В”). Additionally, poorly preserved fragments of a human skull and a tubular bone (possibly a femur) were found in the northeastern part of the grave, just above the surface level. Fragments of burnt wood and an accumulation of small charcoals were recorded in the southern corner of the grave.

Based on the condition of the remains, it appears that the deceased were first dismembered and then, after some time, buried in some form of container, possibly a sack. The skulls were placed near the bones. In the western corner of the grave, adjacent to a cluster of bones, a stone flake was found. Beneath the bones, a small pottery vessel with a sharp bottom and with jar-shaped form was discovered. Fragments of Comb-Pit pottery were found in the southern and northern corners of the grave, while a scraper was located at the bottom of the grave near the skull in the northeastern part.


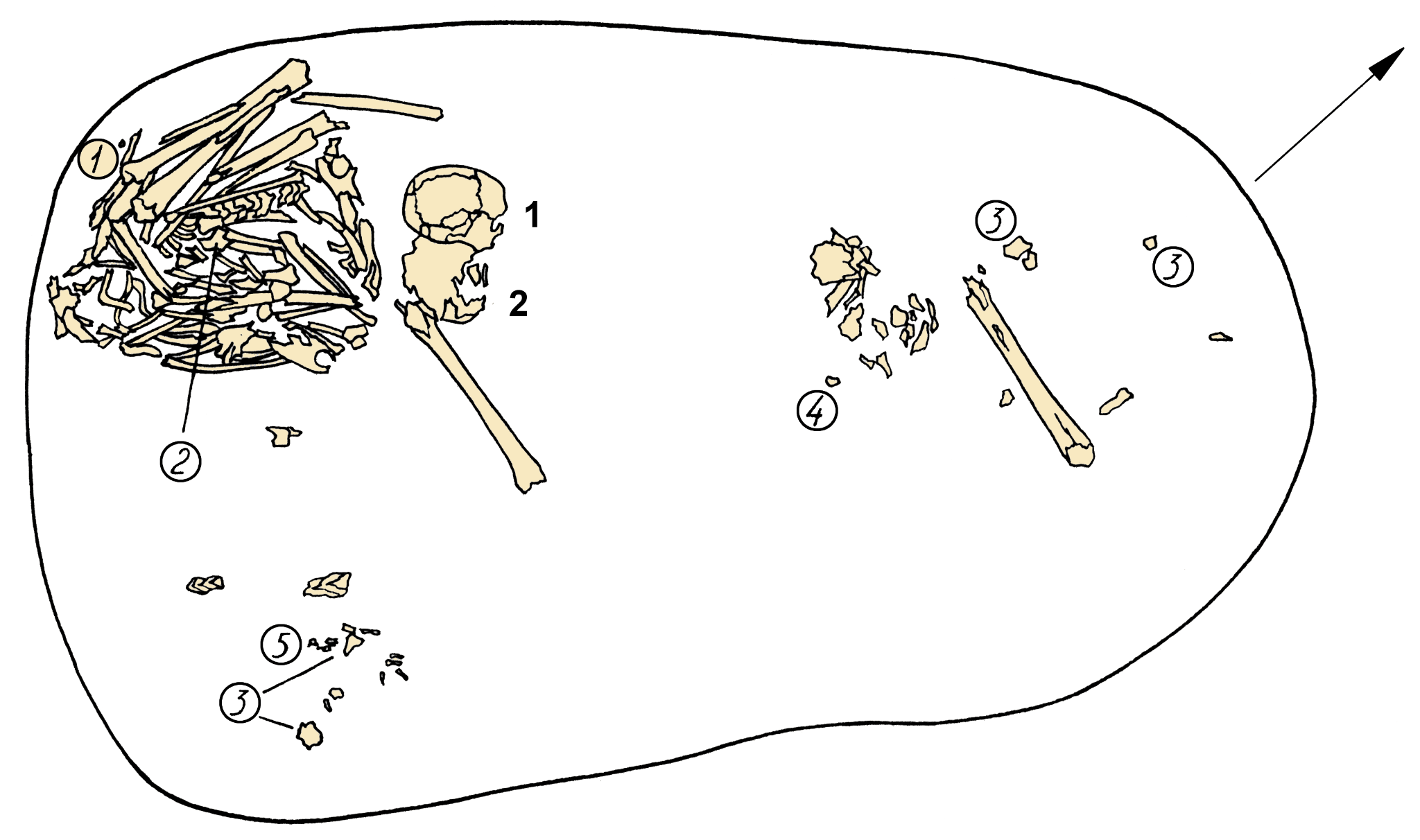


***Figure S44.*** *Burial 66 at Borovyanka-17 (image by Olga Sherstobitova).*

###### Burial 83 (skull F – Individual ID I6829, female, skull D - I6828, male)

**Burial 83** was identified as an elongated spot of black humus sandy loam, extended along a northeast-southwest line, expanding towards the southwestern part, with dimensions of 355-365x140-220 cm. An accumulation of fish scales, measuring 40x25 cm and 3-5 cm thick, was found at the southeastern side of the wall. The filling of the grave contained a fragment of Comb-Pit pottery, a knife-shaped piece, and numerous small charcoal fragments. The bottom of the grave was flat, with a slight rise towards the southwestern part, while the walls were slightly inclined and nearly sheer. A ledge was observed in the western corner, and remains of a burnt board, measuring 95 cm in length and 3-10 cm in width, were discovered near the southeastern side.

A total of eight individuals were found in the burial, including three postcranial ***skeletons (1-3)*** and five ***skulls (A-H)***. ***Skull F*** belonged to ***sleleton 2***, and ***skull G*** belonged to ***skeleton 1***. ***Skeletons 1*** and ***2*** were laid in supine positions, while ***skeleton 3*** consisted of a cluster of human bones intentionally broken and chopped.

***Skeleton 2***, found in the northeastern part of the grave, was in a supine position with the head facing northeast. The skull (***skull F***, “Е” in Cyrillic) (individual ID I6829), left femur, tibia, and partial foot bones were well-preserved. Traces of ocher, up to 3 cm thick, were observed around the skull, pelvis, and ankle bones.

The ***skulls D*** (individual ID I6828) and ***E*** (“Г” and “Д” in Cyrillic) were excavated at the northwestern wall of the grave. They were positioned on their bases.

The burial is dated to 4214–3958 calBCE (5210±30 BP, PSUAMS-3920), based on the radiocarbon date of ***skull F***.


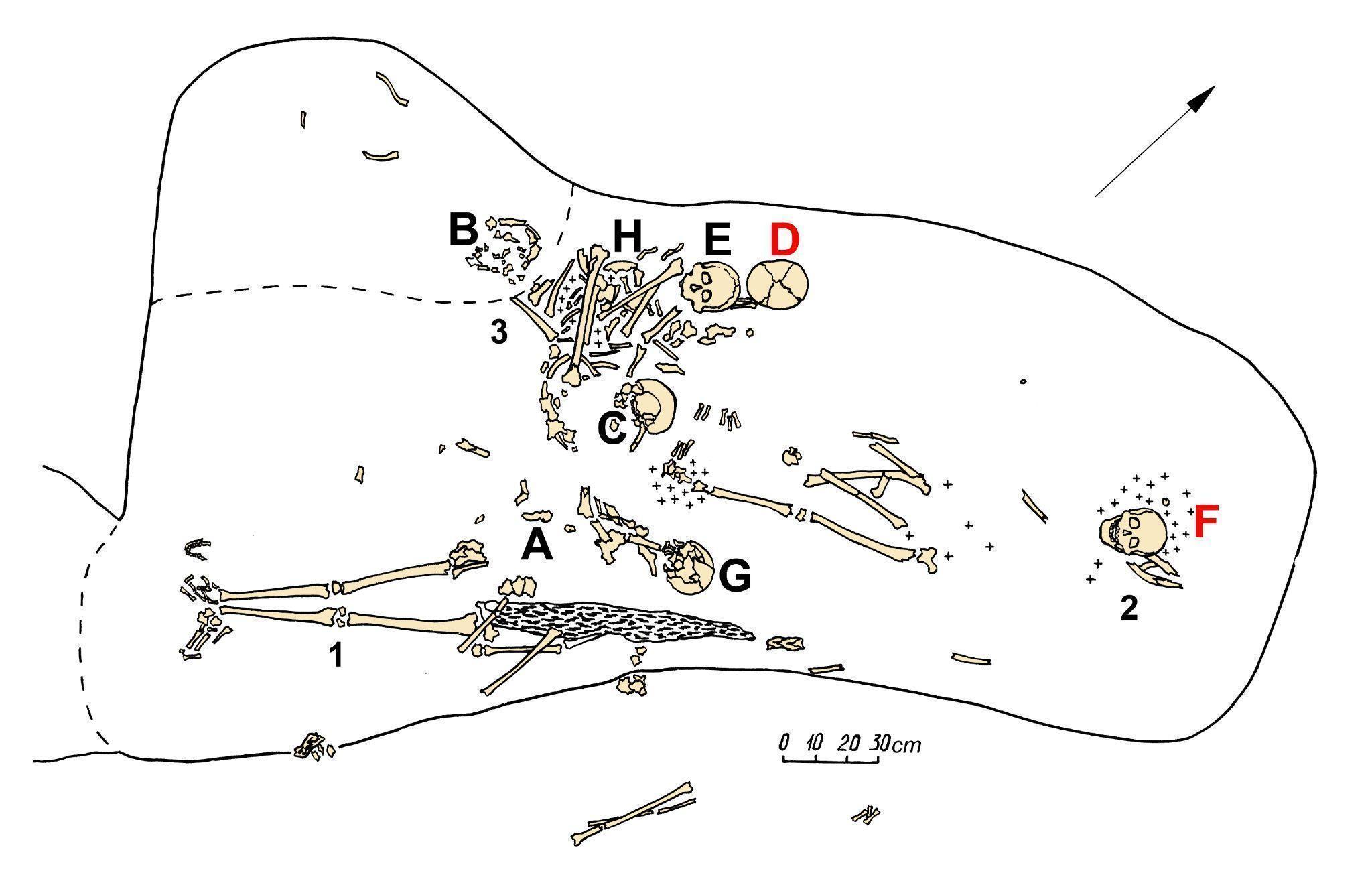


***Figure S45****. Burial 83 at Borovyanka-17. Sequenced skulls D and F are labeled in red (image by Olga Sherstobitova). Skeletons are labelled by numbers, and skulls by letters.*

###### Unidentified burials (individual IDs I6827, male, I6830, male, I6959, female)

Three skeletons from Borovyanka-17 lack burial numbers. Individual I6830 is dated to 2911–2881 calBCE (4270±20 BP, PSUAMS-4828), individual I6827 - to 3937–3654 calBCE (4990±30 BP, PSUAMS-3919).

The individual I6830 is dated to a later period than all the other skeletons from Borovyanka-17. Noticeably, it has a substantially different ratio of  δ15N/δ15C from other burials on the site, which may reflect differences in food consumption and economy.

##### Eneolithic phase of the Okunevo site complex

The archaeological site complex located in the Tatarsky Uval (ridge) area (Omsk region), collectively known as "Okunevo," was investigated by V.A. Mogilnikov, B.A. Konikov, A.I. Petrov, and V.I. Matyushchenko. Excavations were conducted in various areas, identifying separate sites within the complex: the Okunevo-3, 3a, 4 cemeteries, the fortified settlement 5, and the cemeteries 7 and 7a.

**Okunevo-3**, situated in the southern part of the Tatarsky ridge on a small promontory with a height of 10 meters, was studied by V.A. Mogilnikov (1972) and B.A. Konikov (1976-1977). The graves of the Neolithic, Early Bronze Age, Early Iron Age, and Middle Ages have been investigated at this site.

**Okunevo-3a** is a group of three burial mounds located between the Okunevo-3 burial ground (to the south) and the Sargat culture settlement Okunevo-9b, which dates back to the 1st millennium BCE. Excavations were conducted by V.A. Mogilnikov (1972) and V.I. Matyushchenko (1997).

**Okunevo-4** is a kurgan cemetery consisting of numerous small mounds, adjacent to the southern side of Okunevo-3. Burial mounds investigated at this site yielded materials from the 16th-17th centuries, associated with the southern Khanty population.

Occupying a promontory 10 meters high above the floodplain at the northern end of the Tatarsky ridge, **Okunevo-5** was studied by B.A. Konikov. The site revealed materials from the Stone Age to the Middle Ages and includes a settlement, an Early Bronze Age settlement, and a potential cult site from the Early Iron Age.

**Okunevo-7** (including its western edge, Okunevo-7a) is situated in the northern and central parts of the Tatarsky ridge, south of Okunevo-5. It was excavated by A.I. Petrov (1980), V.A. Mogilnikov (1981), and V.I. Matyushchenko (1985, 1987-1991). Described as a flat burial site, it contains burials from the late Neolithic to the late Middle Ages. The estimated area of the necropolis is 1000 square meters, with approximately one-third explored.

The Neolithic to Early Bronze Age burials are dispersed among the Okunevo-3, 5, and 7 sites, which can be viewed as parts of a single necropolis (Topleko, 2015; Bolshanik et al., 2001). We sequenced 11 individuals from these burials at Okunevo. The chronological attribution of the site is based on 6 newly obtained radiocarbon dates on human bones/teeth, which fall into two periods. The earliest date was obtained from burial 189, attributing the initial subphase of the cemetery to 4228–3979 calBCE. Other burials belong to a later subphase, which lasted from 3089 to 2784 calBCE. The chronological differences are reflected in burial rites and artifact assemblage of the studied burials. The earliest grave 189 contained a secondary burial with traces of postmortem decomposition of the skeletons, while the later graves yielded bronze artifacts and pottery.

###### Burial 47B (individual ID I6846, male)

**Grave 47**, oriented in a north-south direction, was identified as a subrectangular feature measuring 295 x 100 cm, with a depression of 10-15 cm observed on the western side. The feature was filled with dark, highly humus-rich sand. Within the southwestern corner of the burial pit, two distinct shelves were identified at depths of 35 cm and 50 cm below the surface, respectively. Along the southern wall, another 10 cm wide shelf was observed, situated 30 cm below the ground level. This burial feature yielded the discovery of two interments.

***Burial B***, also oriented along the north-south axis, exhibited an elongated subrectangular shape and was situated at a depth of 60 cm. Its dimensions measured 295 x 100 cm. At the southern extremity of the grave, a conspicuous ocher deposit, measuring 15 x 20 cm with a maximum thickness of 5-7 cm, was prominently evident below the ceiling. A similar ocher deposit, measuring 25 x 15 cm, was also identified at the northern end of the grave, positioned to the left of the cranial remains. The ceiling structure was preserved in the southern half of the grave, occupying an area measuring 170 x 100 cm. The ceiling was constructed from longitudinally arranged sod blocks, measuring 15-20 cm in width and up to 10 cm in thickness.

Upon removal of the ceiling, the skeletal remains of the primary individual were uncovered (individual ID I6846), positioned supine and fully extended, with the head oriented to the north. Unfortunately, the skeletal preservation was notably poor, with the skull fragmented into numerous pieces, and the bones of the thoracic and lumbar regions of the skeleton in a deteriorated state. In the southern portion of the grave, beneath the ceiling, poorly preserved fragments of a child's cranial remains were also encountered. Additionally, fragments of a bone ring and an asymmetric stone knife were recovered from this layer within the grave. The grave fill yielded 25 fragments from three to five vessels adorned with Comb-Pit and Krotovo culture motifs (Matyuschenko, Polevodov 1994).


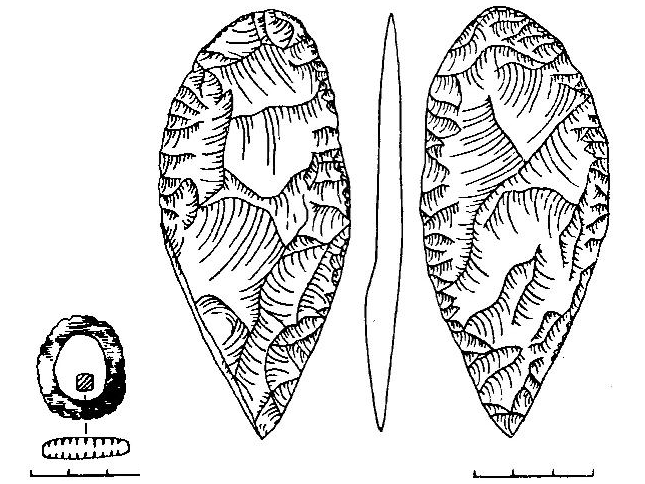


***Figure S46.*** *Okunevo site complex, burial 47B. Grave goods (by Matyuschenko, Polevodov 1994, modified).*

###### Burial 59 (individual ID I6834, male)

**Burial 59** was situated within the upper subsoil layer at a depth of 40 cm from the surface, with approximate dimensions of 180 x 50 cm. It was oriented along a north-south axis, with a slight deviation towards the northeast and southwest. Within this grave, an articulated skeleton was unearthed, positioned in an extended supine orientation with the head directed to the north. The preservation of the remains was limited to the following elements: the skull (in poor condition), several bones of the upper thoracic region, the left humerus, both ulnas, the lower portion of the vertebral column, the pelvis, and the larger bones of the lower extremities.

Adjacent to the head, positioned on the right side, was a poorly preserved lower fragment of a flat-bottomed vessel adorned with vertical zones featuring comb stamp decorations. Additionally, a copper plate torque was placed around the neck of the deceased individual. Fragments of ceramic artifacts were recovered from the grave infill (Matyuschenko, Polevodov 1994).

**Burial 59** is dated to 2909–2784 calBCE based on a human bone/tooth (4250±20 BP, PSUAMS-4831).


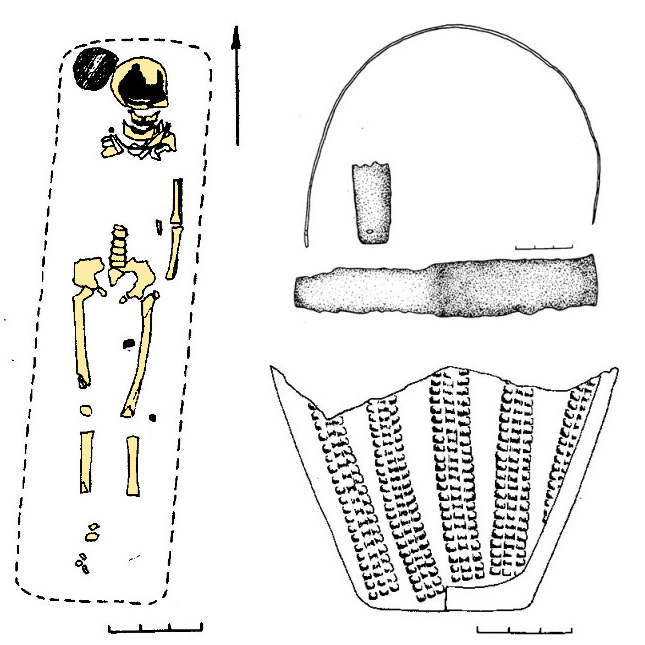


***Figure S47.*** *Okunevo site complex, burial 59. A plan of the burial and the grave goods (by Matyuschenko, Polevodov 1994, modified).*

###### Burial 62 (individual ID I6847, female)

**Burial 62**, with approximate dimensions of 170 x 75 cm, was oriented along a north-south axis. Within the northern half of the grave pit, the remains of a skeleton were preserved, represented primarily by the skull, facing southward. To the north of the skull, remains of tubular bones were discovered, albeit in a poorly preserved state. Surrounding the skull and bones, a discernible darker humus stain was observed.

Located to the south of the skull, a notable cluster consisting of 40 ring-shaped antler beads and a spectacle-shaped bronze pendant was unearthed. At the southern terminus of the grave, a clay tetrahedral flat-bottomed vessel was uncovered, accompanied by a bronze tetrahedral awl. The vessel in question exhibits characteristics of a squat closed jar, characterized by a swollen body. It features vertical lines of stick impressions that divide the middle portion of the body, delineated by double horizontal lines of oblique short impressions. The bottom of the vessel is adorned with radially arranged imprints (Matyuschenko, Polevodov 1994).

**Burial 62** is dated to 3082–2910 calBCE based on a human bone/tooth (4370±25 BP, PSUAMS-3923).


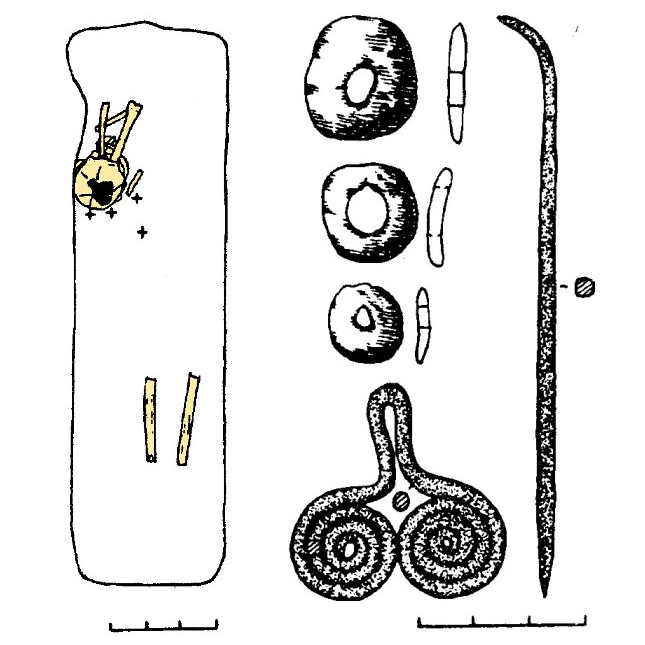


***Figure S48.*** *Okunevo site complex, burial 62. A plan of the burial and the grave goods (by Matyuschenko, Polevodov 1994, modified).*

###### Burial 65 (individual ID I6950, male)

**Burial 65** was identified as an elongated oval area with light humified soil containing numerous ocher inclusions. The dimensions of the grave pit measure 265 x 70 cm, with a depth of 90 cm, and it is aligned along a north-south axis.

Within the grave, a skeleton was discovered, positioned in an extended supine posture with the head oriented toward the north. The left hand of the individual rested upon the pubis, while the right hand was placed adjacent to the right thigh. Notably, the feet were absent, and the condition of the skull was severely compromised, having been crushed. The lower jaw was located to the left of the skull.

The entire expanse of the grave exhibited a layer of ocher, with a more concentrated presence observed around the areas corresponding to the legs and head. Adjacent to the right hand of the deceased, a stone knife was recovered (Matyuschenko, Polevodov 1994).


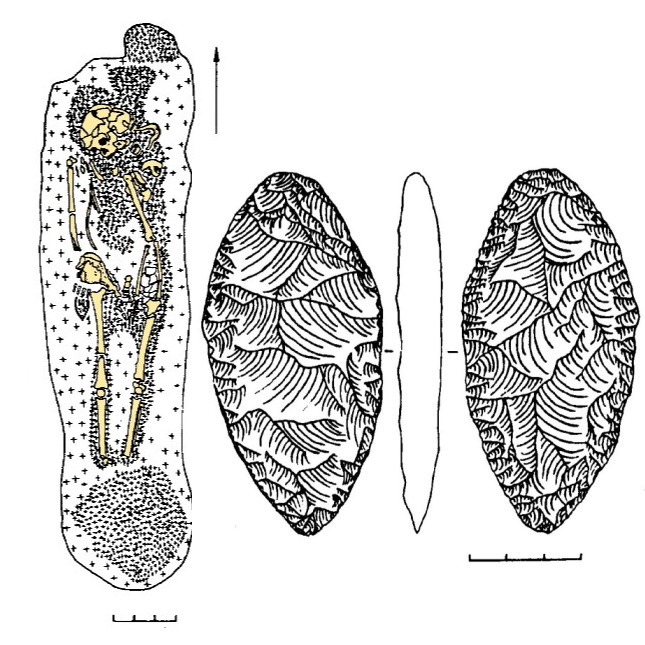


***Figure S49.*** *Okunevo site complex, burial 65. A plan of the burial and the grave goods (by Matyuschenko, Polevodov 1994, modified).*

###### Burial 69 (individual ID I6835, female)

**Burial 69** was uncovered at a considerable depth of 150 cm beneath the surface. The grave pit exhibited distinct characteristics, manifesting as a well-defined rectangular shape measuring 220 x 90 cm, with a depth of 80 cm. Notably, a significant portion of the skeleton was concealed beneath a substantial layer of large stone blocks. The stone slabs employed in the grave's construction measured approximately 25 cm in thickness, though some regions were thinner, ranging between 10-15 cm. Their width varied between 10-20 cm.

Within the confines of the grave, a relatively well-preserved skeleton was situated in a supine position with its head oriented toward the north. A fine layer of ocher had been meticulously distributed across the entirety of the bone assemblage. In proximity to the tibia bones, remains of an infant (not sequenced) were discovered, though they were severely deteriorated. Alongside this infant burial, a large quartzite side-scraper, exhibiting signs of double-sided craftsmanship, was situated by the right hand. Positioned beside the left hand, another side-scraper crafted from the same quartzite material was found. Additionally, the grave yielded a clay sphere and two fragments of early pottery (Matyuschenko, Polevodov 1994).


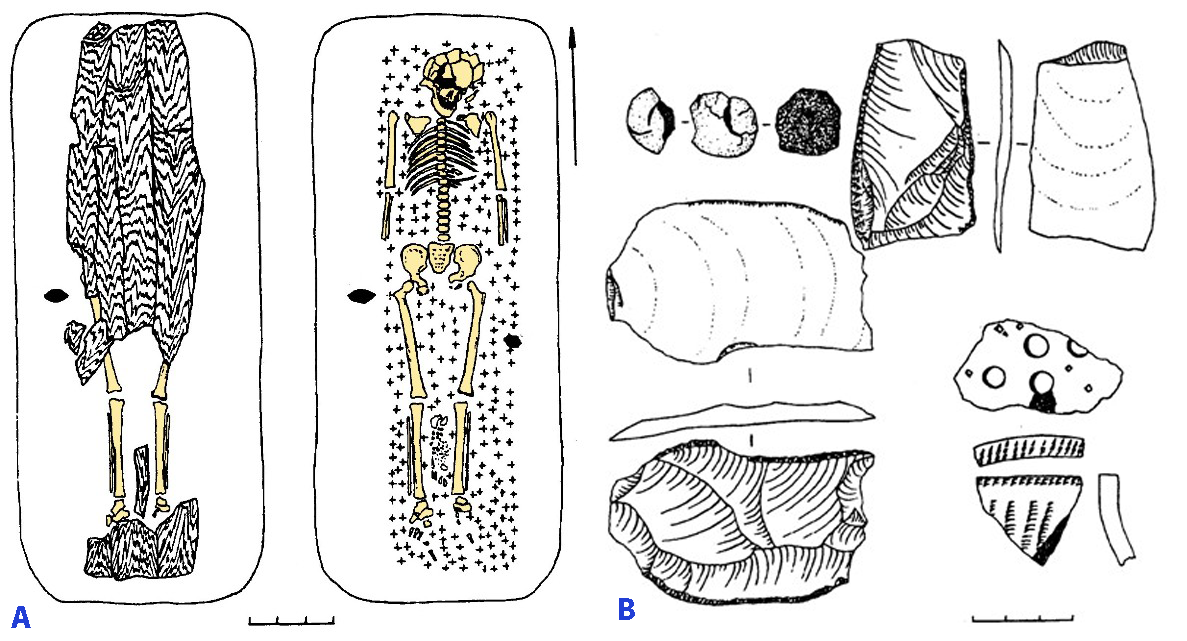


***Figure S50.*** *Okunevo site complex, burial 69.* ***A*** *- plan of the burial and* ***B*** *- grave goods (by Matyuschenko, Polevodov 1994, modified).*

###### Burial 79 (individual ID I6837, male)

**Burial 79** exhibited a pronounced elongated shape, extending from the south to the north, with rounded corners. Its dimensions measured 385 x 95 cm, and the depth reached 35 cm. The primary component, situated to the north, encompassed a standard cavity measuring 290 cm in length. Adjacent to this segment, on the southern side, lay a relatively smaller pit, measuring 100 cm in length and 25 cm in depth.

The principal grave contained remains of an individual who had been interred in a supine position with the head oriented to the north. Remnants of a fragmented skull, segments of both larger ulnas, minor fragments of the pelvis, femurs, and a solitary left fibula were found. Traces of ocher were detected in the vicinity of the skull and around the abdominal area.

The deceased had been furnished with a suite of accompanying artifacts. To the northwest of the skull, a stone flake and a fragment of a stone axe were situated. Positioned on the eastern side of the skull lay a stone whetstone, two pieces of quartzite, placed one atop the other, bearing a striking resemblance to rock crystal fragments, along with a diminutive pebble. Adjacent to the right temple of the deceased, a second comparable pebble was discovered, alongside an artifact crafted from a branch of a petrified tree infused with iron oxide. The latter artifact took on the form of an elongated, mildly curved rod, with one side potentially displaying deliberate modeling. Its appearance bore resemblance to an animal's visage: it featured a protrusion extending from the forehead to a muzzle-like structure, with a slightly projecting lower jaw. On the left side, there existed a longitudinal groove demarcating the mouth. The remaining portion of the artifact exhibited significant deformation, likely attributable to natural fracturing. The rod measured 14.5 cm in length and 1.8 cm in width. Interspersed between these items were a scraper, a chert fragment, and assorted flakes. Notably, in the southern segment of the grave, closer to its northern extremity, a cluster of human teeth was observed within a 30 x 20 cm ocher deposit (Matyuschenko, Polevodov 1994).


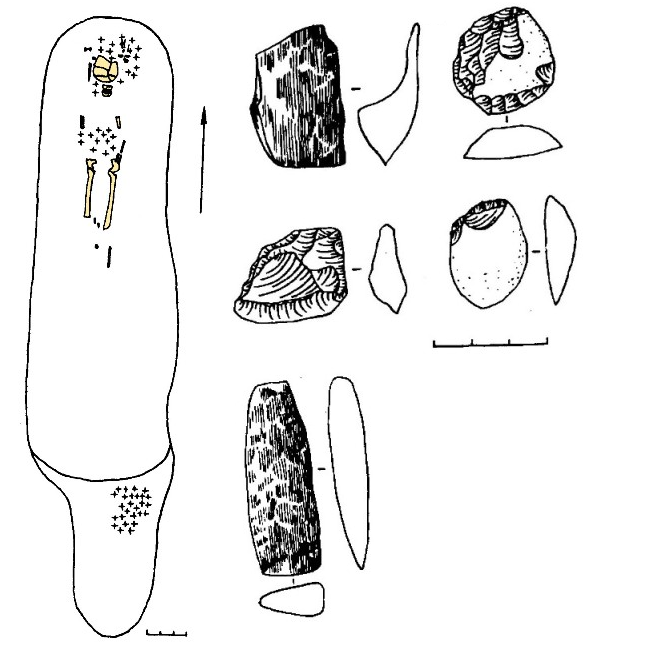


***Figure S51.*** *Okunevo site complex, burial 79. A plan of the burial and the grave goods (by Matyuschenko, Polevodov 1994, modified).*

###### Burial 168 (individual ID I6833, male)

**Burial 168**, aligned along a north-south axis, had dimensions measuring 230 x 85 cm and reached a depth of 10 cm. This burial contained a fragmented and poorly preserved skull, alongside remains of a left humerus, both femurs, and a left tibia. The deceased was interred in a supine position, fully extended, with the head oriented towards the north.

Within the confines of the grave, several artifacts were uncovered, including a stone semi-disk situated in the pelvic region and a bronze ring positioned near the right ulna. The semi-disk, crafted from limestone slabs and displaying significant damage along its entire periphery, exhibited a semi-lunar configuration, with one side appearing smooth and the other rounded. Notably, a central aperture was present within the arc, likely intended for suspension or attachment purposes (Matyuschenko, Polevodov 1994).

The burial is dated to 3089–2917 calBCE based on a human bone/tooth (4385±20 BP, PSUAMS-4830).


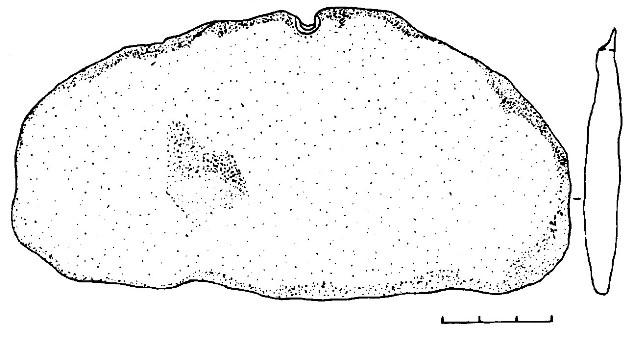


***Figure S52.*** *Okunevo site complex, burial 168. A stone artifact found in the grave pit (by Matyuschenko, Polevodov 1994, modified).*

###### Burial 172 (individual ID I6964, male)

The precise delineations of **grave 172** could not be ascertained. The human remains were discovered approximately 15 cm above the subsoil level, positioned in a supine orientation with the head oriented toward the north. Some fragments of the skull, along with a portion of the right humerus and damaged femurs, have survived. The right femur, a fragment of a tibia, and assorted bone fragments were recovered from the western portion of the grave. Notably, a fragment of a bone ring was located amid the leg bones.

In proximity to the burial, a pottery vessel was unearthed. This vessel is a flat-bottomed, partially enclosed jar with a slightly everted rim featuring a protrusion along the edge. The ornamentation comprised two horizontal bands embellished with broad-toothed comb impressions, interspersed with vertical lines created by comb impressions. Between these pairs of vertical lines, oblique comb hatching was applied. Additionally, a horizontal herringbone pattern, produced using the same comb impressions, adorned the central region of the vessel's body. The vessel's base exhibited a random arrangement of comb impressions and perforations (Matyuschenko, Polevodov 1994).

**Burial 172** is dated to 3013–2902 calBCE based on a human bone/tooth (4340±20 BP, PSUAMS-4850).


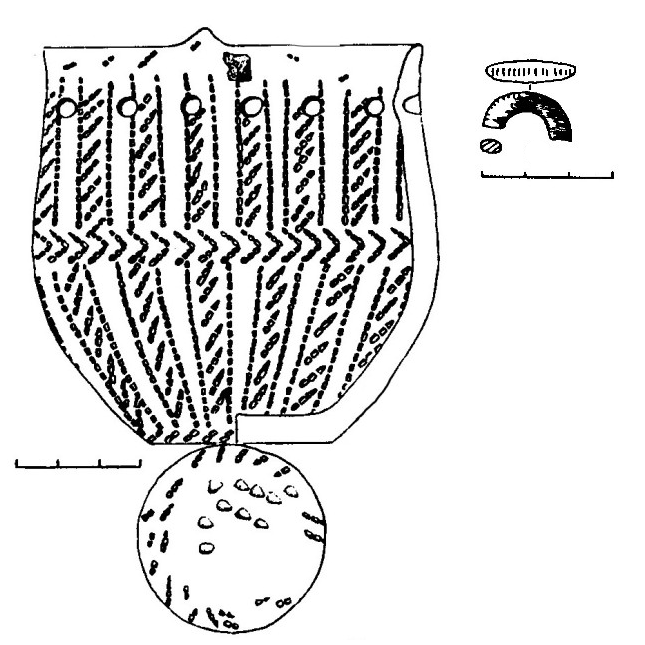


***Figure S53.*** *Okunevo, burial 172. Grave goods (by Matyuschenko, Polevodov 1994, modified).*

###### Burial 180 (individual ID I6836, female)

The precise boundaries of **grave 180** were not identified; therefore, its estimated dimensions were roughly 110 x 55 cm, with a depth of approximately 10 cm. Intriguingly, it was revealed that this particular grave served as an overlay for another grave, designated as **grave 181**.

Within this grave, a juvenile interment was discovered, arranged in a lateral recumbent position with the body fully extended and oriented to the north. Notably, the preservation of the remains was limited to the left side of the damaged cranium, fragments of the vertebral column, and portions of the femur.

Remarkably, the grave assemblage encompassed vestiges of bronze artifacts, including a distinctive bronze hryvnia fashioned in the form of a curved plate. This hryvnia was recovered in close proximity to the cranium, positioned around the neck of the interred individual (Matyuschenko, Polevodov 1994).

###### Burial 189 (individual ID I6848, male)

**Burial 189**, oriented along the NE-SW axis, exhibited an elongated configuration, extending beyond the confines of the excavation squares. In the southern portion, the spot's width was between 150-170 cm, while in the northern half, it ranged from 110 to 120 cm. This location had once accommodated a trench roughly 110 cm wide and 75 cm deep below the subsoil level. Adjacent to the ditch's western margin, on the southern side, small depressions in the subsoil, each measuring up to 35 cm in diameter, were observed.

The contents of the ditch consisted of two distinct horizons. The uppermost layer, approximately 60 cm thick, comprised humified soil devoid of any accompanying objects. Below this, a lower layer of dark humus-rich soil was encountered, with a minimal thickness that transitioned into humified sandy loam. The ditch's base remained uniformly level.

Notably, at the northern extremity of the ditch, situated 120 cm from the excavation's northern wall, a sudden drop in the floor's elevation, by 25 cm, formed a pit measuring 110 x 120 cm in size. This depression reached a depth of 65 cm below the ditch floor and 120 cm relative to the upper horizon of the mainland. Intriguingly, this pit was replete with compact humus-laden sandy loam, yet it yielded no associated objects of archaeological significance.

Within the confines of the ditch, two interments were discerned, albeit slightly displaced to the southeast. These interments are referred to as the "southwestern" (1) and "northeastern" (2) individuals.

***Individual 1*** (individual ID I6848), positioned in the southwestern portion, appeared to have been interred in a supine posture. The cranium, severely crushed and flattened, was oriented towards the right. Notably, the right shoulder and forearm bones were conspicuously absent. The right thigh exhibited a slight displacement to the right, with the bones of the right shin situated beneath the pelvis and adjacent to the right thigh's head. Remarkably, the bones of the right foot were positioned near the thoracic ribs, suggesting that the right leg may have been flexed at the knee, placing the foot in the vicinity of the buttocks. In contrast, the left thigh displayed a displacement to the left, with its head positioned beneath the pelvis. The left tibia overlapped the lower extremity of the left thigh. Notably, the left leg may have initially been inverted, and the phalanges of the left foot and other bones were located slightly to the left. The arrangement hints at the possibility that the corpse may have been bound before interment. Adjacent to the skull, a petal-shaped pendant was discovered.

***Individual 2***, presumably interred prior to the first, exhibited an unconventional resting position. The cranium was found in an upward-facing orientation. To the south of the skull, the bones of the shoulder and forearm were situated, while to the west, the femurs, lacking their heads, were discovered, with the joints oriented towards the skull. Additionally, the right thigh was superimposed over the left, and below and adjacent to the hip bones, the pelvic bones were found in a disarrayed state, with the right pelvic bone occupying the position of the left, beneath the thigh bones, and vice versa. Phalanges from this interment extended beneath the skull of the first skeleton.

The configuration of the second skeleton may be indicative of a secondary burial event, occurring subsequent to the disruption of ligaments, resulting in the bones being arranged without adherence to a specific order (Matyuschenko, Polevodov 1994).

**Burial 189,** ***individal 1*** has been dated to 4228–3979 calBCE based on a human bone/tooth (5250±30 BP, PSUAMS-3924), demonstrating the earliest date from all radiocarbon-dated skeletons at the cemetery.


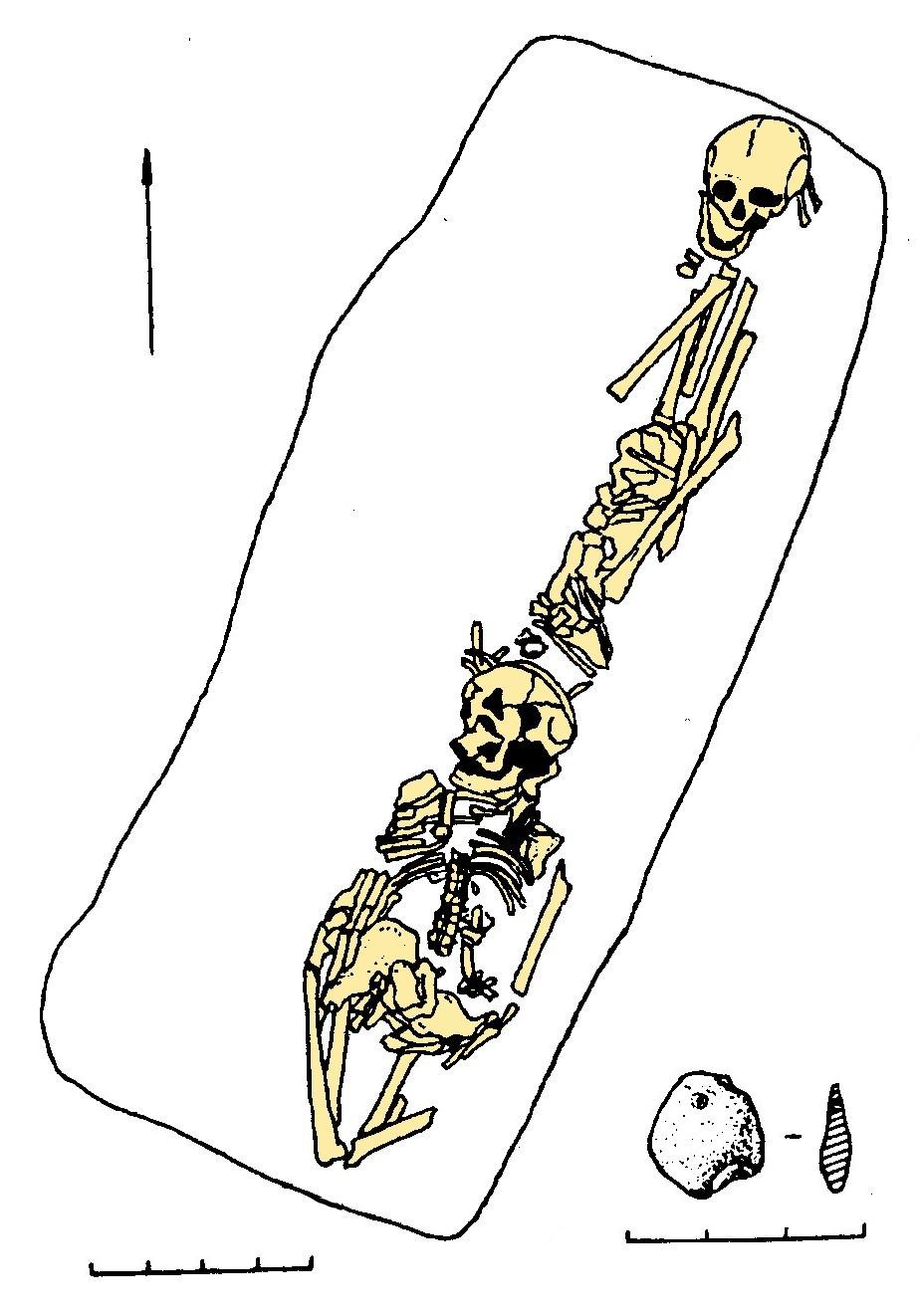


***Figure S54.*** *Okunevo site complex, burial 189. A plan of the burial and the grave goods (by Matyuschenko, Polevodov 1994, modified).*

###### Burial 222 (individual ID I6839, female)

**Burial 222**, oriented along the NE-SW axis, exhibited dimensions of 85 x 240 with a depth of 40 cm, presenting a subrectangular outline. Approximately 30 cm below the ground surface, a layer of calcined soil, measuring 20 cm in thickness, was uncovered in close proximity to the grave.

Within the confines of the grave, poorly preserved remains were found: a skull, leg bones, and several vertebrae. Notably, the skull was situated approximately 30 cm above the bottom of the grave, whereas the remaining skeletal elements were positioned at the base. The skull had become traversed by tree roots, potentially suggesting that these roots elevated the skull to its higher position within the grave. No funerary objects or artifacts were recovered from this burial pit.

The skeleton from **burial 222** has been dated to 3011–2890 calBCE (4320±25 BP, PSUAMS-3921).


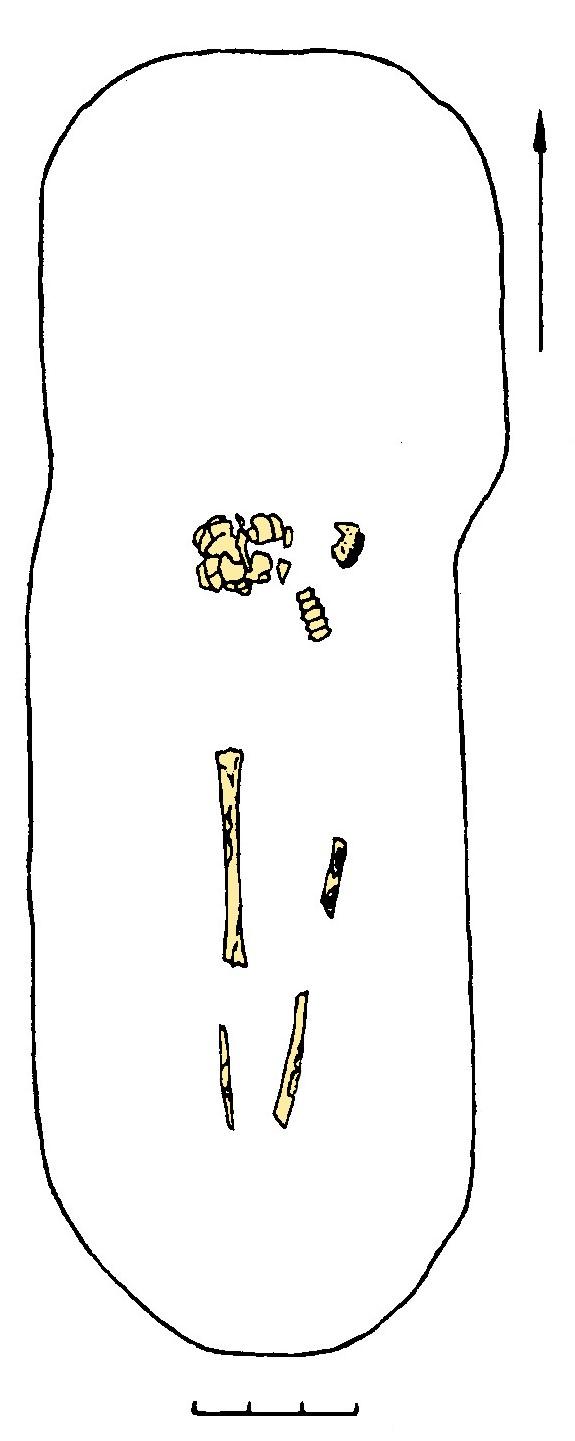


***Figure S55.*** *Okunevo site complex, burial 222. A plan of the burial (by Matyuschenko, Polevodov 1994, modified).*

##### Ostrov-2 site

###### Burial 1 (individual ID I2144)

From the Ostrov-2 site, we sequenced one individual, from **burial 1**. It is dated to 2911–2677 calBCE (4230±35 BP, OxA-33167).

##### Chernoozerye-1 burial site

The Chernoozerye-1 burial ground was excavated by Vladimir Gening between 1967 and 1969. The site is situated on the periphery of the floodplain terrace of the Irtysh River. This particular area of the terrace has been repeatedly utilized by the ancient population for habitation purposes. Alongside the burials found at the Chernoozerye-1 burial ground, other archaeological sites from the Paleolithic, Neolithic, and pre-Andronovo Bronze Age have been discovered here. Additionally, a burial ground associated with the Andronoid culture, five small burial mounds, and one large burial mound from the Early Iron Age were found. Furthermore, a burial from the Iron Age was also uncovered at the site. The excavated area of the burial ground covers approximately 3,500 square meters in total (Gening and Stefanova, 1994).

###### Burial 57 (individual ID I6050, male)

The grave pit of **Burial 57** was oval-shaped and oriented in the NNE-SSW direction, with dimensions of approximately 150x80x10 cm. Based on the well-preserved bones, the deceased individual is identified as a male, estimated to be around 50 years old. The burial position is crouched, with the individual lying on his right side, and the head oriented towards the north-northeast. The femurs are positioned at a right angle to the spine, while the lower leg bones are nearly perpendicular to the femurs. The left hand is bent with the palm resting on the stomach, while the right hand is bent with the palm towards the face. No grave goods were present in this burial (Gening and Stefanova, 1994). The skeleton in this burial is dated to 4216–3968 calBCE (5225±25 BP, PSUAMS-2944); freshwater reservoir effect is possible.

## Urals

### Trans-Urals (Tobol River Basin in Western Siberia)

According to current archaeological understanding, the Early Neolithic period in the Trans-Urals (6000–4700 BC) is characterized by the simultaneous existence of two significant cultural traditions, namely Koshkino and Kozlov-Mys (Shorin and Shorin 2021, 2022; Vybornov et al., 2014).

The Late Neolithic period (5000–3950 BC) is represented by two distinct cultural traditions: Boborykino-Bas’yanovo, and Poludenka-Comb-Pottery (Shorin and Shorin 2021, 2022; Shorin et al., 2015; Vybornov et al., 2014). Some researchers also distinguish the Sosnovy Ostrov culture (Usacheva 2016), but other treat it as a part of the Poludenka-Comb-Pottery tradition.

The Eneolithic period in the Trans-Urals is dated to 4300-3000 BC and is characterized by the coexistence of several cultures identified through the ornamental features of their pottery. These cultures, namely, Shapkul’, Andreevskaya (Andreevskoye-Ozero), Lipchinskaya (Lipki), and others, are collectively referred to as the Trans-Urals Eneolithic, which shares material culture characteristics with the Comb-Pit Pottery of southern West Siberia (Mosin, 2008; Epimakhov and Mosin, 2015). Clear boundaries between the Neolithic as well as Eneolithic cultural groups in the Trans-Urals were absent, with numerous transitional types present. The populations of the Trans-Urals during those periods engaged in mobile practices aligned with the annual economic cycle. This involved movement across foothill, forest, and forest-steppe territories, with each community having a network of long-term (winter) and short-term (summer) camps and settlements.

The primary subsistence strategies revolved around hunting and fishing. Hunting targeted animals such as horses, elks, bears, red deer, roe deer, badgers, martens, otters, as well as waterfowl. Fishing activities are inferred from the discovery of variously shaped sinkers among the artifacts, as well as the presence of fish bones and scales.

#### Trans-Ural Late Neolithic-Eneolithic (Russia_CombPitWare_Eneolithic)

##### Sosnovy Ostrov occupation site

The site of Sosnovy Ostrov is situated on a sand dune that rises approximately 2 meters above the swampy region of the lake known as Maly Tarman. This lake is part of the Tarman Lakes, which are remnants of a paleochannel of the Iska River, a left tributary of the Tobol River in the Middle Urals.

During the excavations of the site, several cultural assemblages were discovered. Assemblage 1 comprises ceramics and housing structures attributed to the Kozlov culture, which represents the early Neolithic period. Assemblage 2 consists of ceramics and housing structures associated with the Sosnovy Ostrov culture. Within Assemblage 2, a distinct subset labeled as Assemblage 2a was identified, consisting of pottery and Burial 2, both associated with the Sosnovy Ostrov culture of the Late Neolithic. Finally, Assemblage 3 is interpreted as belonging to the Eneolithic period.

###### Burial 1 (individual ID I12696, female)

Three burials were made within the central section of the western wall of a dwelling pit measuring 4 meters in length and 1.3 meters in width, attributed to Assemblage 2, corresponding to the Sosnovy Ostrov culture, shortly after its collapse.

**Burial 1** was discovered in the central area of the dwelling pit, at a depth of 170 cm. Within the burial, remains of a human skull and small calcified bones, potentially belonging to a complete skeleton, were excavated. The orientation of the pit follows a northeast-southwest line. The grave goods associated with this burial include a polished "hammer" crafted from green stone. The skeleton from this burial has been dated to 4245–4050 calBCE (5320±25 BP, PSUAMS-9040). Cultural attribution of the burial, however, is problematic. It was constructed after the Sosnovy Ostrov house was destroyed but before the site was occupied by the population with Comb-Pit pottery.

Additional burials within the site were unearthed at deeper layers within the dwelling pit (individuals from the following two burials were not sequenced).

**Burial 2** is located at a depth of 190 cm. At this depth the pit diminishes in size, measuring 1.4 x 1.75 meters. At this level, a human jawbone was discovered. The pit is oriented along a northeast-southwest line. Grave goods include a miniature vessel adorned with a comb stamp, which corresponds to Assemblage 2a of the Sosnovy Ostrov culture.

**Burial 3** was excavated at a depth of 220 cm, this grave pit measures 210 cm in length and 80 cm in width. On the southern side, the pit is bounded by a 1-meter-long charcoal deposit from Assemblage 1. Within this section of the grave, the remains of a third burial were found, including a skull, hand bones positioned across the grave pit, and traces of rib fragments. The positioning of the skull and ribs indicates that the head of the interred individual was oriented towards the east-northeast. No grave goods were discovered in association with this burial.

##### Boborykino-2 occupation site

A burial located at the Boborykino Dune was discovered approximately 0.1 km southwest of the former village of Boborykino, situated on a cliff along the left bank of the Iset River in the forest-steppe region of the Tobol River Basin.

Konstantin Salnikov and Otto Bader associated the burial with the Boborykino-2 occupation site due to its spatial proximity, being only 0.2 km away, as well as due to similarities observed in the shapes of pendants and pointed axes found in the occupation layer and as part of the grave goods (Salnikov 1962, Bader 1970). Although the cultural attribution was not explicitly specified, it was mentioned that the burial was of Neolithic origin.

At the Boborykino-2 site, pottery classified as Poludenka-type was attributed to the Neolithic period, while pottery decorated with comb stamps was assigned to the Eneolithic period. Additionally, pottery of the Boborykino type was associated with the Early Bronze Age (Salnikov 1961). Konstantin Salnikov further delineated a chronological sequence of cultural groups recorded at the site, which included the Neolithic, Late Neolithic, Boborykino, Andronovo, and Kamenogorsk cultures, Sarmatian period, and the Bakal culture (Salnikov 1961).

###### Damaged burial (individual ID I8280, female)

The female individual interred in the **damaged burial** (individual ID I8280), estimated to be around 60 years old, was in a supine position, and the bones were painted by ocher. Adjacent to the right side of the skull, the remains of another skull and a jaw belonging to a child aged 10-12 years were discovered. The bones of a young adult were also found in exposed layers at the river bank, raising the total number of buried individuals nearby the Boborykino-2 site to three.

Among the grave goods, a total of 26 polished bone drop-shaped pendants were unearthed in the vicinity of the neck of the first interred individual. Additionally, 20 flint knife-shaped pieces, measuring up to 20 cm in length, were found, along with two stone axes, three bone beads, and several end scrapers positioned to the right of the skull. Traces of red dye were observed on the tools (Salnikov 1962; Bader 1970; Vokhmentsev 2000). The burial is dated to 4338–4176 calBCE based on a human bone/tooth (5410±25 BP, PSUAMS-4278). Based on the δ15N/δ15C ratio, we do not expect a freshwater reservoir offset in this date.

Mikhail Vokhmentsev attributed the Boborykino burial to the Eneolithic period based on the presence of drop-shaped pendants, as similar decorations were predominantly found in Eneolithic burials and sites (Vokhmentsev 2000). However, the date of the burial makes the Late Neolithic attribution more plausible.

#### Trans-Ural Eneolithic (Russia_CombPitWare_Eneolithic)

##### Gladunino-3 occupation site

The Gladunino-3 site is located on the eastern shore of Lake Voronye, at a height of 2.5 – 3 m from the water surface level. The settlement supposedly occupied an area of 2100 square meters. The peripheral part of the site was excavated, where 5 storage pits, traces of post holes, and a single burial were recorded. Two phases have been identified on the site: Early Neolithic (the Koshkino and the Kozlovskaya (Kozlov-Mys) cultures) and Eneolithic (the Sosnovy Ostrov, Shapkul’, and Andreevskaya (Andreevskoye-Ozero) cultures) (Shilov and Maslyuzhenko 2002). According to recent studies, the Sosnovy Ostrov culture is attributed to the Late Neolithic period.

###### Inhumation burial (individual ID I6305, female)

The **inhumation burial** (individual ID I6305) discovered at the Gladunino-3 site was made in a rectangular grave pit measuring 219x61x20 cm and aligned along the southwest-northeast axis. The interred individual was positioned on their back, in a supine position, with the head facing eastward. The anatomical order of the skeleton was maintained, except for the deliberate dissection of the left pelvic bone, which was neatly placed between the ribs and the right half of the skeleton. The fingers of the right hand were flexed, while the left hand was laid in a resting position on the pelvis. Additionally, the left femur exhibited slight rotation.

The grave goods accompanying the burial include:

1. A fragment of a spherical stone mace, crafted from brown iron ore and featuring a perforation;
2. Fragments of pottery decorated by comb stamp patterns and pit impressions, which the excavators attributed to the Sosnovy Ostrov culture;
3. An end scraper made of gray-green jasper;
4. Two blades and two flakes made of grayish flint and quartz (Shilov and Maslyuzhenko 2002; Khohlov and Nechvaloda, 2002).

The burial is dated to 2894–2701 calBCE based on a human bone/tooth (4210±20 BP, PSUAMS-2910). Cultural attribution of the burial is problematic, but its chronological position suggests the Late Eneolithic period when the region was mostly populated by groups with Comb-Pit pottery or closely related to them.


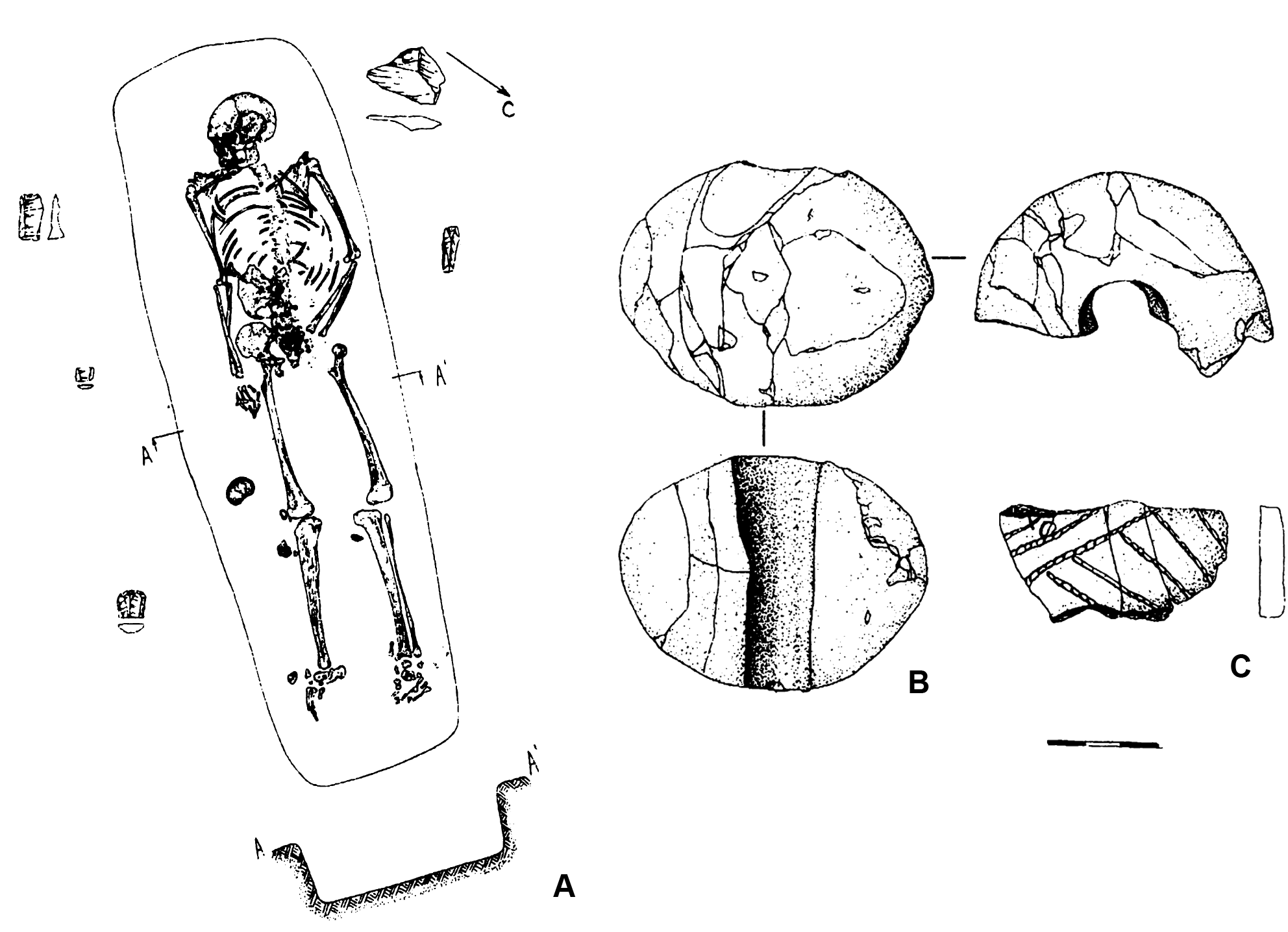


***Figure S56.*** *The Eneolithic burial at the Gladunino-3 site.* ***A*** *- burial;* ***B*** *- stone mace;* ***C*** *- pottery sherd (by Shilov and Maslyuzhenko 2002, modified).*

### Ural Mountains and Cis-Urals

In the Urals region, a wide range of archaeological cultures and cultural types from the Neolithic to the Eneolithic period have been identified. These cultures often coexisted contemporaneously, and their main distinguishing feature was the ornamentation found on everyday pottery vessels, such as comb, comb geometric, pricked, incised, false cord, and others.

The burials from the Urals studied in this paper were typically situated in remarkable locations such as caves, grottoes, and beneath rocky overhangs. These burial sites lacked cultural markers, specifically ceramic vessels, which makes it impossible to attribute them to a specific Ural Neolithic or Eneolithic culture. Therefore, the most appropriate term to describe these burials accurately would be "Urals Early Eneolithic Burials".

#### Urals Early Eneolithic Burials (Russia_UralsEneolithic)

Systematic exploration of grottoes and caves in the Urals began in the 1930s under the direction of Sergei Bibikov. In 1938, a burial was discovered in the Buranovskaya cave, which appeared to be that of a male individual. Accompanying the burial were 35 drop-shaped pendants made of ophite (serpentine). The following year, a female burial was excavated in the rocky canopy of the Starichny ridge, which contained 15 pendants made of ophite, similar to those found in the Buranovskaya cave. In the same year, remains of two individuals were found in the Ust-Kataevskaya-2 cave. One of them, presumably a child aged 5-7 years, was remarkably well-preserved. The child's bones were placed in a recess in an incorrect anatomical order and covered with stones. Among the grave goods in this burial were 20 incisors of a bobak marmot without holes, intensely colored with ocher, as well as 53 conical beads made of Dentalium mollusk shells, 36 flat and rounded shell beads, and 32 teardrop-shaped pendants made of gray slate stone (Bibikov 1950).

The next phase of research took place in the 1980s when Yuri Serikov discovered and excavated burials at Kamen’-Dozhdevoy.

In more recent years, cultural remains have been investigated in the Burmantovsky grotto and Sokolinaya Gora caves, revealing a complex assemblage of artifacts, likely originating from destroyed ground burials. In the Burmantovsky grotto, apart from a human tooth, 69 various pendants made of bone and shell, and 37 arrowheads made of flint and green tuff-porphyrite were found. A similar assortment of artifacts was uncovered in the Zhilische Sokola cave.

No established tradition of funeral rites has been identified; however, several features have been recorded:

1. Inhumation in shallow pits.
2. Burials covered with earth, stones, or interred in grottoes and caves.
3. Evidence of a fire cult associated with the burials.
4. Traces of ocher found in shallow pits (8-30 cm deep), on the bones, and on grave goods in some burials.
5. Skeletons, when laid in the correct anatomical order, displayed a northeast or southwest orientation.

Typical is also the presence of drop-shaped pendants made of stone or bone, as well as jewelry crafted from teeth of various animals, among the grave goods. The general chronological framework for the Urals Early Eneolithic burials is 4500–3900 BCE (Serikov, 1998; Nokhrina and Shorin, 2000; Epimakhov and Mosin, 2015; Shilov and Maslyuzhenko, 2002).


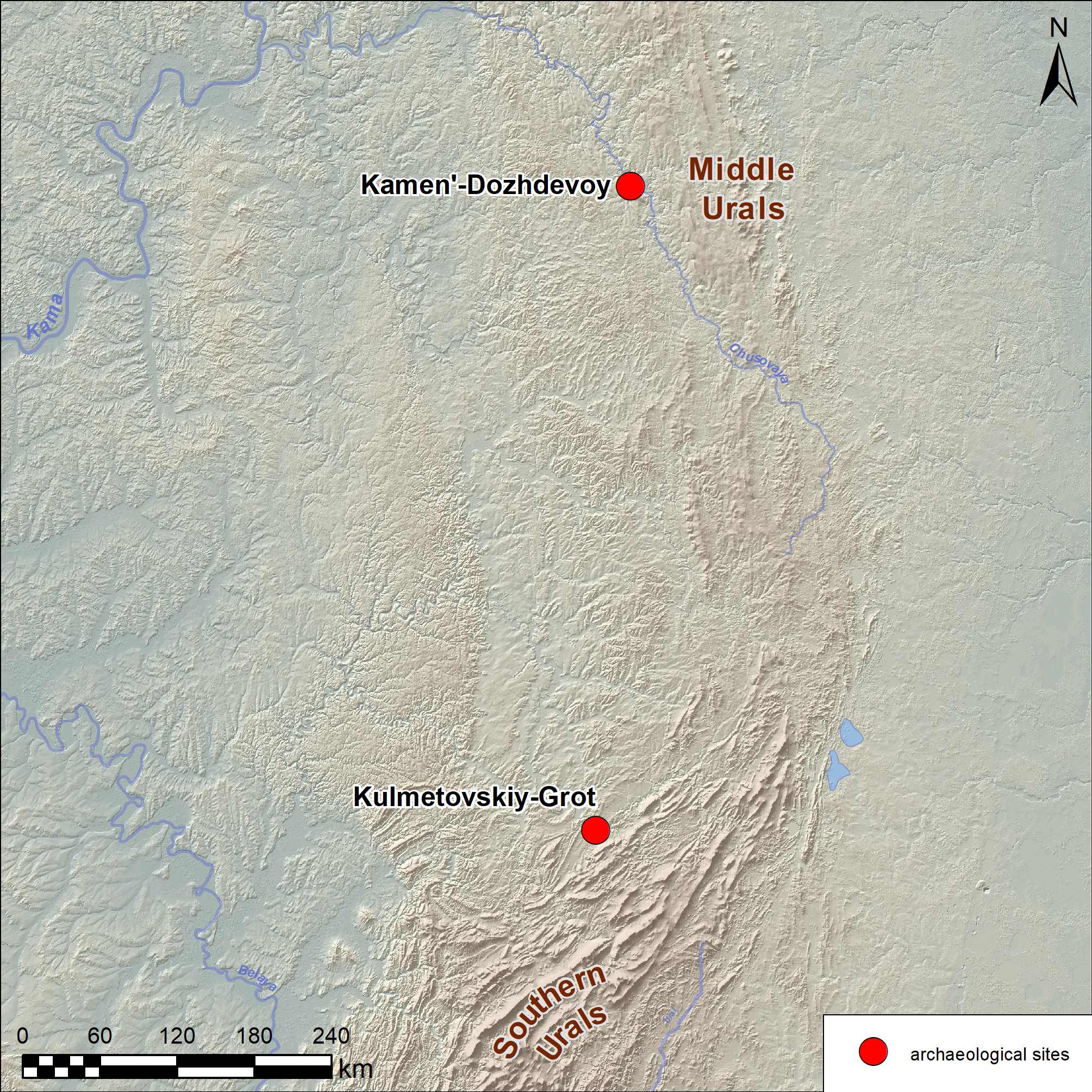


***Figure S57.*** *Caves Kulmetovskiy-Grot and Kamen’-Dozhdevoy.*

##### Kulmetovskiy-Grot cave (Kulmetovskiy grotto)

The Kulmetovskiy grotto is situated on the right bank of the Ai River, a left tributary of the Ufa River, which in turn is a right tributary of the Belaya River, a left tributary of the Kama River. The cave is located approximately 3-4 kilometers upstream from the village of Kulmetovo. At the entrance of the cave, there is a collection of rock paintings.

###### Burials 1 (individual ID I20893, male) and 2 (I20948, male)

During archaeological excavations of cultural layers dating to the Eneolithic period, fragmented remains of two human skeletons were discovered, documented as **burial 1** (individual ID I20893) and **burial 2** (individual ID I20948). Unfortunately, the burials were not excavated carefully, and as a result, the burial rites associated with these individuals remain unknown (Shirokov, 2009). **Burial 1** is dated to 4047–3961 calBCE (5195±25 BP, PSUAMS-9029), and **burial 2** to 4315–4052 calBCE (5335±25 BP, PSUAMS-9031); both results are based on a human bones/teeth.

##### Kamen’-Dozhdevoy cave

Kamen'-Dozhdevoy is a prominent rocky cliff reaching heights of up to 35 meters, situated on the right bank of the Chusovaya River. A grotto of karstic origin is located in this cliff, with an entrance situated 14 meters above the water level. In 1988, Yuri Serikov discovered burials in this grotto.

These burials consist of inhumations placed in shallow pits and adorned with a sprinkling of ocher. The first burial pertains to a female individual in a supine position, while the second burial corresponds to a male individual in a crouched position. Notably, both skeletons exhibit an orientation towards the northeast.

In the male burial, an assortment of artifacts was recovered, including 2 biconical bone arrowheads, 4 stone arrowheads, a stone knife showing signs of grinding, a bone harpoon, a scraper crafted from a chalcedony flake, 10 beaver incisors, one fox tusk, 8 bone ornithomorphic figurine-pendants, and 6 drop-shaped bone pendants made of beaver bones. Additionally, 14 flake-blanks, presumably stored in a bag, were also discovered at the burial site (Serikov, 1993).

###### Burial 1 (individual ID I11637, female)

The female burial, designated as **burial 1**, was found in a supine position, oriented towards the northeast. Among the grave goods accompanying the burial, a substantial assemblage of 76 pendants made of animal teeth was identified. Specifically, these pendants consisted of 73 lower fangs from otters, 2 moose incisors, and a bear tooth. Additionally, the burial yielded undrilled incisors from an elk and a lower canine of a fox (Serikov, 1993). The burial is dated to 4496–4253 calBCE (5530±60 BP, COAH-5154).


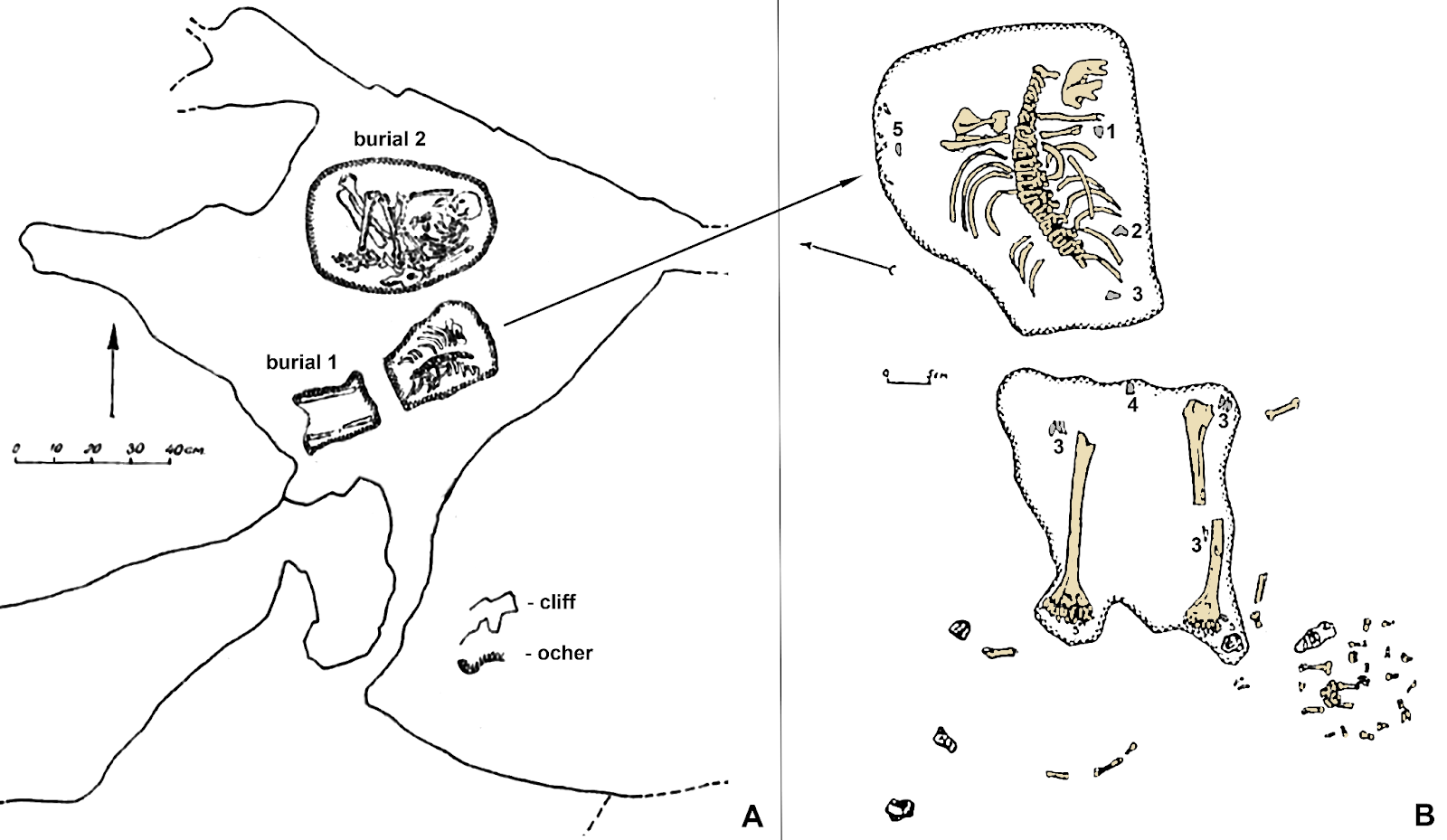


***Figure S58.*** *Burials 1 and 2 in the Kamen’-Dozhdevoy cave.* ***A*** *- excavation plan;* ***B*** *- burial 1;* ***1, 2*** *- flakes;* ***3*** *- pottery sherds;* ***4*** *- scrappers;* ***5*** *- perforated animal teeth (by Serikov, 1993, modified).*


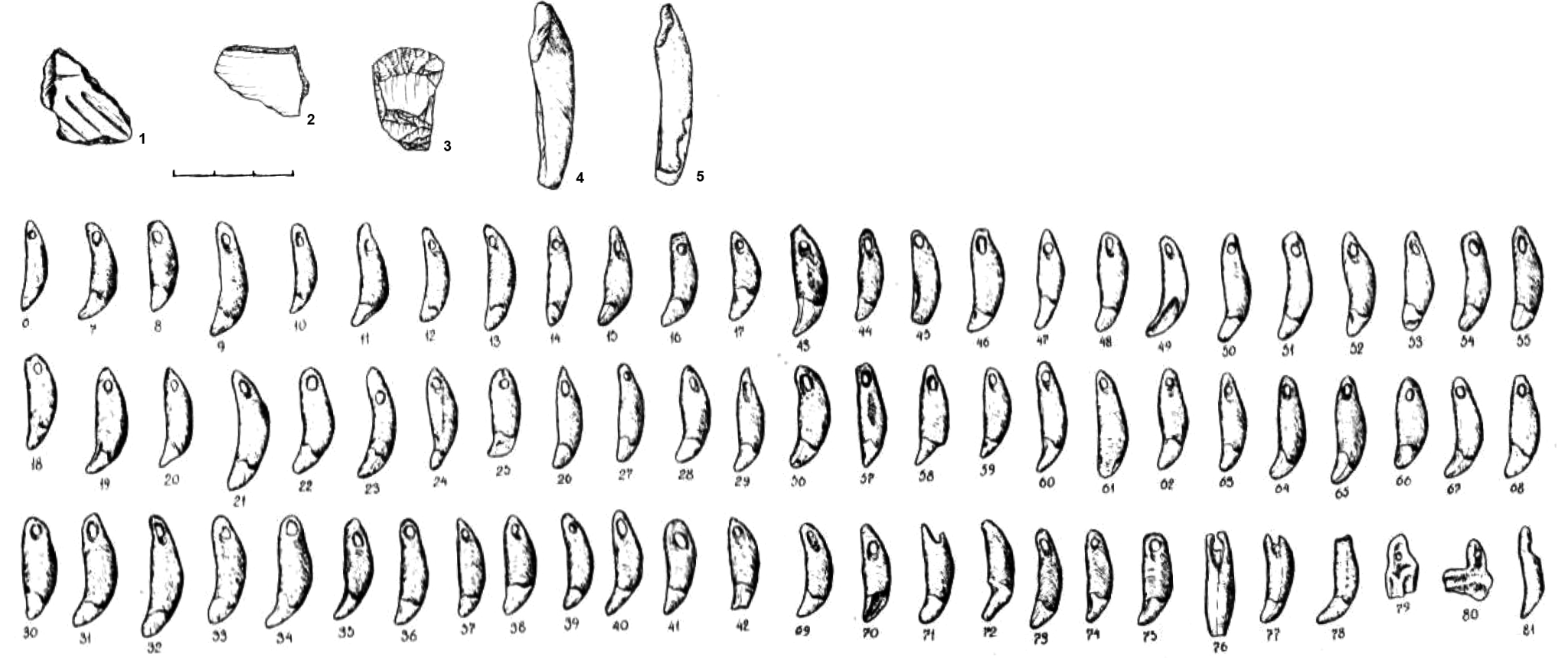


***Figure S59.*** *Grave goods from burial 1 in the Kamen’-Dozhdevoy cave.* ***1*** *- pottery sherd;* ***2*** *- flake;* ***3*** *- scrapper;* ***4, 5*** *- moose incisors (by Serikov, 1993).*

## Temperate zone of Eastern Europe

### Lower Kama and Middle Volga Region

#### Elshanka Early Neolithic culture (Russia_Elshanka_EN)

Elshanka-type sites were initially excavated during the mid-1970s in the forest-steppe and steppe regions of the Urals. The culture derived its name from the eponymous site, Staraya Elshanka-2. Presently, approximately 25 sites in the forest-steppe Volga region have been investigated, all of which contain artifacts associated with the Elshanka culture.

The pottery of the Elshanka culture exhibits characteristic features, including relatively thin walls with a smoothed or burnished outer surface, as well as a smoothed inner surface. Vessels with vertical walls are prevalent, while pointed and flat bottoms are found in equal numbers, with rounded bottoms occurring less frequently. Ornamentation is typically confined to the upper third of the vessel, primarily on the neck and occasionally at the rim's top. The typology of the flint tools remains a topic of debate.

One of the most widely accepted hypotheses proposes that the Elshanka culture emerged in the first half of the 7th millennium BCE. It persisted in the forest-steppe Volga region at least until the middle, and possibly even the third quarter, of the 6th millennium BCE. The origin of Neolithization (i.e., the appearance of pottery) in the forest-steppe Volga region remains highly contentious. However, there is a notable resemblance between the ceramics of Central Asia and the pottery of the Elshanka culture.

During its later development stage, the Elshanka population interacted with communities practicing the inscribed pottery tradition in the Lower Volga region. The initial advancement of the Lower Volga population appears to be associated with increased aridization, particularly during the latter half of the 7th millennium BCE.

At the peak of aridization (end of the 7th millennium BCE), the Lower Volga population expanded into the forest-steppe region, leading to the formation of the Middle and Late Neolithic Volga-Kama culture. The first half of the 6th millennium BCE witnessed the coexistence of late Elshanka complexes alongside early Volga-Kama culture complexes, ultimately resulting in the gradual decline of the Early Neolithic ceramic tradition (Andreev et al., 2021).

##### Chekalino-4 occupation site

The Chekalino-4 site is situated on the Sok River, specifically at the northern extremity of the first terrace above the floodplain. It is positioned between an ancient dry channel, which has left behind two remaining lakes, and a flooded floodplain depression that directly adjoins the modern river channel. Excavations have revealed that the site existed in close proximity to water during ancient times. Artifacts from the Neolithic, Eneolithic, and Bronze Ages have been discovered at this location.

Regarding the Neolithic ceramics found at the site, they can be categorized into two distinct cultural and chronological complexes: Elshanka and Volga-Kama. The Elshanka findings are covered by soil that developed on alluvial deposits and are located at the level of the buried soil surface. Radiocarbon dating conducted on pottery sherds gave a broad chronological range from 8450 BCE to 6010 BCE (Andreev et al., 2018).

###### Neolithic burial B (individual ID I6413, female)

Approximately 4 meters southeast from the channel bank, an excavated burial, commonly referred to as **"burial B"** (individual ID I6413) in recent publications, was discovered by Andrey Mamonov and Aleksandr Khoklov. The exact outlines of the grave pit were not clearly defined. The skeleton was found just below the upper level of the subsoil. The interred individual was positioned in a crouched posture, with the arms placed beneath the head and the legs bent at the knees. The orientation of the skeleton is to the northwest. The grave goods accompanying the burial consist of an unretouched knife-shaped artifact, which was located at the base of the skull. The absence of soil redeposition indicators in the layers above the burial allows us to attribute it to the Elshanka period (Mamonov, 1995; Vybornov et al., 2023). However, the radiocarbon dating of the skeleton to 8281–7971 calBCE (8970±30 BP, PSUAMS-4211) appears highly unlikely, as it contradicts generally accepted dating of the Elshanka culture and, on the other hand, coincides with at least some dates obtained from mollusk shells found at the same site (Le-4781, 8990±100 BP; Le-4782, 8000±120 BP; Gin-7086, 7950±130 BP). We anticipate the presence of a freshwater reservoir effect in these results and instead rely on the date obtained from charred plant remains in pottery clay from the same site (Poz–42051, 7250±60 BP), which corresponds to the period of 6230–6010 calBCE (Andreev et al., 2021).


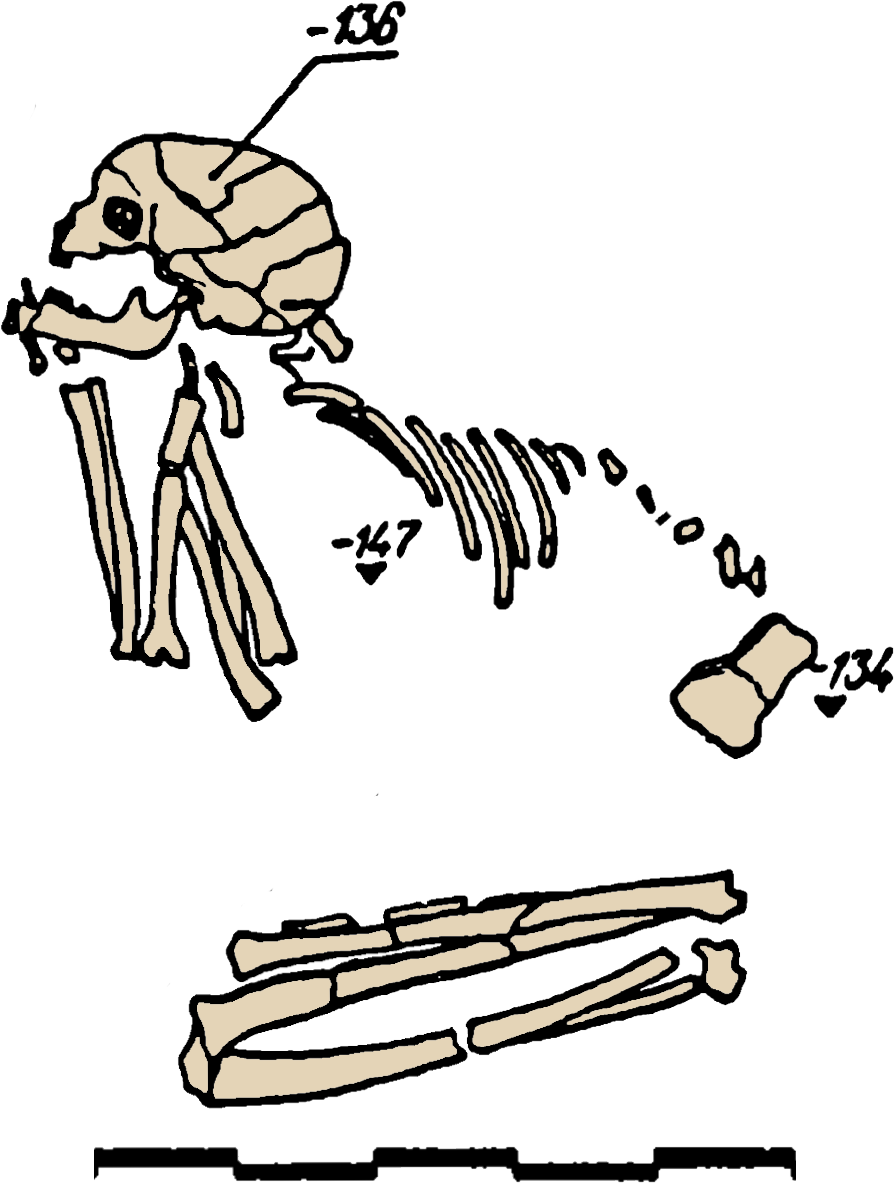


**Figure S60**. Burial B at the Chekalino-4 site (*image provided by Aleksandr Khokhlov, based on Mamonov, 1995)*.

##### Labazy burial mounds

The series of burial mounds near the village of Labazy were excavated under the supervision of Nina Morgunova in 2007-2008. They are situated in the southwestern Urals steppe region, 215 km northwest of Orenburg, on the first terrace above the floodplain of the Buzuluk River, a minor left-bank tributary of the upper Samara River, itself a major tributary of the Volga. The Buzuluk flows approximately 1 km east of the kurgans, through a lightly forested wetland that occupies the bottom of a bowl between high arid ridges. The necropolis was positioned on the left, higher section of the floodplain. Eight mounds were excavated at the site, with the majority of the burials ascribed to the Late Bronze Age Srubnaya culture. Under mound 4, earlier burials were discovered. It appears that the mound was constructed during the Late Bronze Age, potentially covering a cemetery from the Neolithic period (Kuptsova et al., 2019).


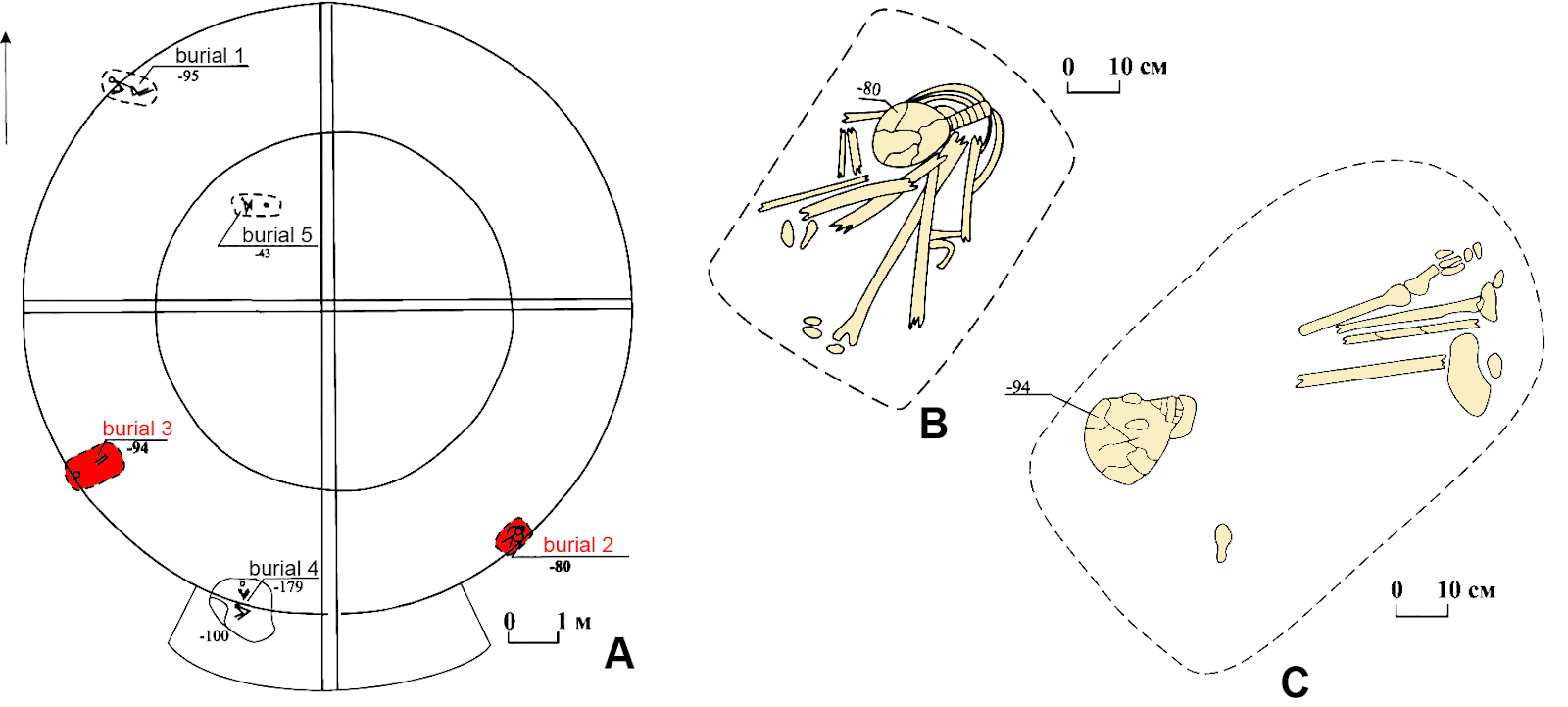


***Figure S61.*** *Labazy burial mounds.* ***A*** *- plan of mound 4 (the burials that contained sequenced individuals are labeled in red),* ***B*** *- burial 2,* ***C*** *- burial 3* *(image provided by Aleksandr Khokhlov, based on Kuptsova et al., 2019).*

###### Mound 4, burial 2 (individual ID I6916, female)

**Mound 4, burial 2** contains a skeleton of an elderly woman aged over 45. The skull was discovered 5.7 meters southeast of the central point of the mound. The skeleton was positioned in a seated posture, with the legs bent at the knees and the arms bent at the elbows, pulled up towards the knees. The spine exhibits a curved shape, causing the body to tilt forward, with the head resting in the hands. The back of the head is oriented towards the northeast, while the front part faces the southwest. It is possible that the woman was bound during the burial ceremony. No grave goods were uncovered in association with this burial. The radiocarbon dating for the skeleton from this burial indicates a period between 6387–6234 calBCE (7430±30 BP, PSUAMS-4261).

###### Mound 4, burial 3 (individual ID I12838, male)

**Mound 4, burial 3** pertains to a male individual aged between 45 and 55 years. This burial was located in the southwestern sector of the mound, approximately 5.6 meters southwest of the center. The approximate dimensions of the burial pit were 106x63 cm. The skeleton was incompletely preserved, with the skull, leg bones, pelvis, and collarbone being recovered from the grave. The head was oriented towards the southwest. No grave goods were found in association with this burial. The radiocarbon dating for the skeleton from this burial indicates a period between 6449–6264 calBCE (7525±26 BP) [Radiocarbon dates combined: (7565±50 BP, PSUAMS-7795), (7510±30 BP, PSUAMS-4260)].

**Burials 2** and **3**, located in **mound 4** at Labazy, are among the oldest burials known in the steppe region of the Volga-Ural area. They have been dated to the 7th - beginning of the 6th millennium BCE. This timeframe aligns with the presence of the Elshanka culture in the Volga forest-steppe region. Notably, the Staraya Elshanka-2 site, where Elshanka pottery was first described, is situated only 30 km north of Labazy. This correlation suggests that the individuals interred at Labazy may have belonged to the same population that introduced the earliest Neolithic pottery to the East European Plain (Kuptsova et al., 2019).

#### Khvalynsk Eneolithic culture (Russia_Khvalynsk_Eneolithic)

The Khvalynsk Eneolithic culture was first identified by Igor Vasiliev in the late 1970s and early 1980s, based on findings from the Khvalynsk-1 burial site, which was excavated by Igor Vasiliev, Sergey Agapov, and Valentina Pestrikova between 1977 and 1979. Khvalynsk culture sites have been identified across a vast territory, ranging from the Northern Caspian Sea in the south to the Middle Sura in the north, and from the Moksha River in the west to the upper reaches of the Samara River in the east.

Pottery serves as the most common and recognizable characteristic of the Khvalynsk culture. Various types of vessels are distinguished, including bell-shaped vessels with two variants - straight-walled and with a rounded body and a contracted neck (sac-shaped), spherical vessels, and bowls. A notable feature of Khvalynsk pottery is the thickened collar rim. The majority of vessels are ornamented, with the ornamentation primarily located in the upper portion, extending over the top and the inner surface of the rim, and occasionally covering the entire outer side, including the bottom. Pricking, stamping, and drawing techniques were used for ornamentation.

Khvalynsk sites mainly consist of burial grounds, although some settlements with long-term dwellings are known in the northern part of the region. Burial grounds are generally flat, and burial pits are typically indistinct. The predominant burial position is a crouched position on the back, with the legs bent at the knees, arms bent at the elbows, and hands on the pelvic bones. There are variations in the arm position, with arms either extended along the body, bent at the elbows with hands on the pelvic bones, or bent with hands on the stomach or chest. Skeletons are also found in positions on the left or right side, as well as in a sitting position. Secondary burials, characterized by bones and skulls stacked in a heap, as well as burials imitating the anatomically correct positioning of skeletal bones, have been identified. The orientation of the burials is primarily to the northeast, northwest, and north, although other orientations are also observed. Ocher use varies from minimal to abundant dressing. Altars containing bones of large and small cattle, horses, and fragments of artifacts have been discovered within the burial grounds.

The chronological timeframe of the Khvalynsk culture is 5000–4500/4300 BCE. The origins of the Khvalynsk culture are subject to debate, with most researchers suggesting connections to the Mariupol group of cultures, particularly the Samara culture in the eastern part of the region. The development of the Khvalynsk tradition is thought to continue within the context of the Yamnaya culture (Korolev and Stavitskiy, 2021b; Kuznetsov, 2020; Morgunova, 2020; Agapov, 2010; Agapov et al.,1990).

##### Lebyazhinka-5 occupation site

The Lebyazhinka-5 settlement is situated on the periphery of the Lebyazhinka farmstead, occupying the first floodplain terrace on the left bank of the Sok River, a tributary of the Volga River. The excavations conducted at the site by Mikhail Turetsky, Nadezhda Ovchinnikova, Aleksandr Khokhlov and others in 1997 have revealed a wide range of materials spanning from the Neolithic period to the late Middle Ages. Among the significant findings, several Eneolithic burials, labeled as burials 8, 9, and 12, were uncovered (Vybornov et al., 2000, 2023; Shishlina et al., 2017).

###### Eneolithic burial 12 (individual ID I6908, female)

**Burial 12** (individual ID I6908) at the Lebyazhinka-5 site contains five skeletons, with the exact contours of the grave pit remaining elusive. The approximate boundaries of the burial were established based on the arrangement of the skeletons. Unfortunately, the paucity of records means that we cannot confirm which of the skeletons in this grave was sequenced for this study.

***Skeleton 1*** was positioned in the western part of the burial as a bundled arrangement, where bones without a skull were concentrated within a small area. This skeleton likely belonged to a mature woman.

***Skeleton 2*** was located east of ***skeleton 1***, in a crouched position on its back, with the head oriented towards the north. The skull of this skeleton is preserved, and it belonged to a woman aged 30-40.

***Skeleton 3*** was situated east of ***skeleton 2***, lying supine and facing north, although the skull was absent. This skeleton belonged to a male aged 35-40.

***Skeleton 4*** was found beneath ***skeletons 2*** and ***3***. The position of the bones suggests that the individual was laid prone, with only the spine, pelvic bones, and a few ribs remaining. The skull, along with the other bones, was missing. The orientation of the buried person has not been determined, and this skeleton belonged to a young male aged 15-17.

***Skeleton 5*** partially underlay ***skeleton 3***. It preserved several vertebrae, ribs, clavicle, sternum manubrium, fragments of the left shoulder blade, and pelvis. Among the long bones, the left humerus and the lower half of the right humerus, along with bones from the right forearm, were present. The skull, however, was missing. Presumably, this skeleton was positioned in an extended position on its back, with a probable northward orientation. It belonged to a male aged 20-30.

**Burial 12** also contained various artifacts, including 250 marmot incisors, 100 carp pharyngeal teeth, one tubular piercing made of bone or shell, and two flint plates. Ocher was employed in the burial ceremony. ***Skeletons 4*** and ***5*** possessed the largest number of artifacts, with ***skeleton 4*** exhibiting a greater quantity of marmot incisors and ***skeleton 5*** displaying more fish teeth. These items were likely sewn onto clothing, possibly as adornments on belts or in the region of the spine (Shishlina et al., 2017).

Initially attributed to the Samara culture, the burial was later reevaluated as belonging to the early Khvalynsk culture. It is dated to 4838–4612 calBCE (5865±40 BP, GrA-64051), with a marmot tooth from the grave providing the basis for this dating.


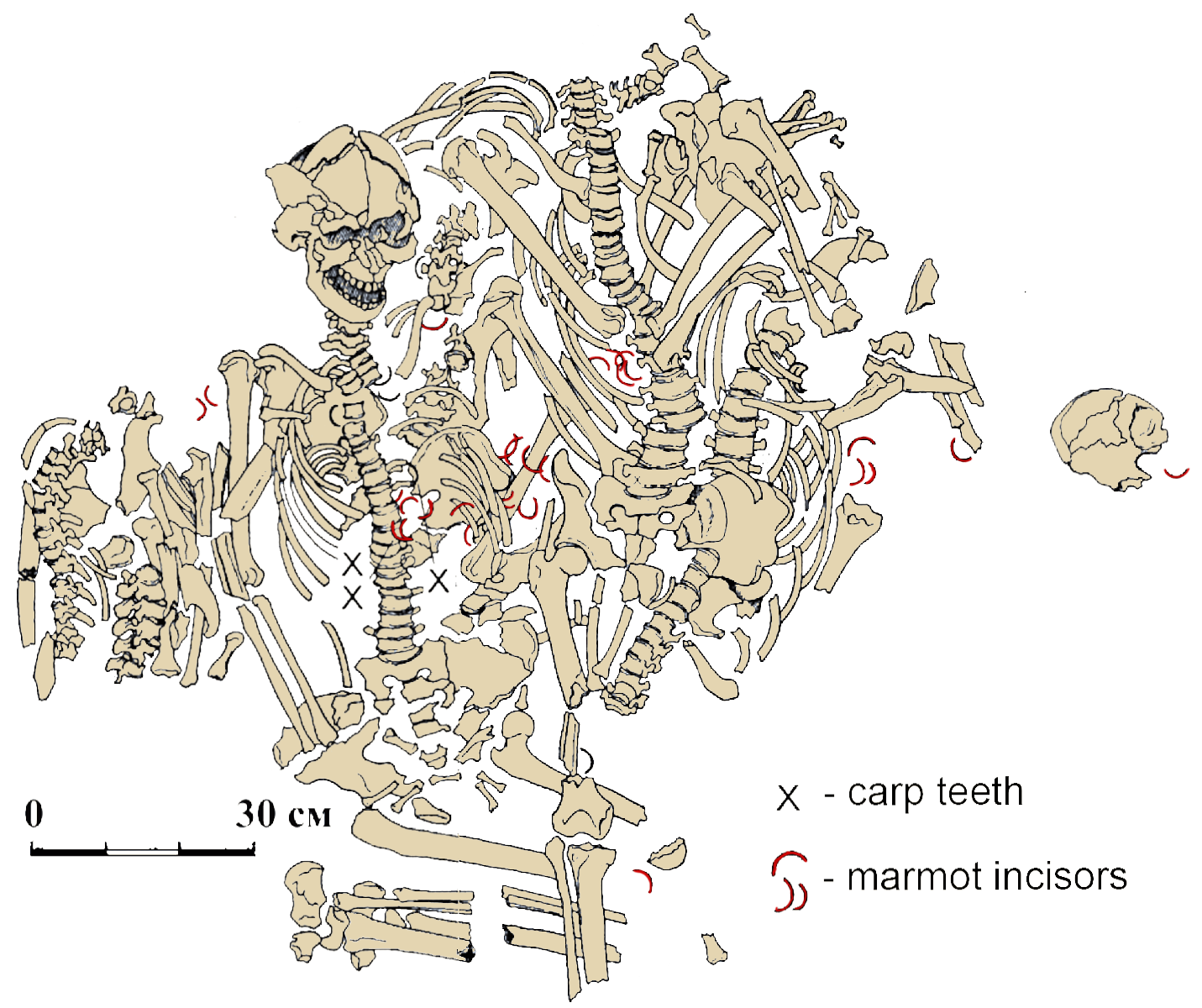


***Figure S62.*** *Burial 12 at the Lebyazhinka-5 site* *(image provided by Aleksandr Khokhlov).*

#### Eneolithic at the Kama Estuary (Russia_KamaEstuary_Eneolithic)

In the Kama Estuary region, the early phase of the Eneolithic period is attested by four sites belonging to a distinct archaeological group known as the Kama Estuary Eneolithic. This group comprises three burial grounds and one occupation site. The burial grounds were situated on flat terraces, occasionally with slight natural elevations. They are characterized by rows of graves, with the deceased interred in either a sitting or supine position. Supine burials are typically individual, while sitting burials are collective.

The grave goods found in the Kama cemeteries consist of objects made of stone, bone, and copper. Woodworking and cutting tools, such as trapezoidal adzes and chisels with grooves and distinct handles, are among the most numerous. Numerous cutting tools such as blades, scrapers, incisors, knives, and points have been discovered. Military equipment includes willow-shaped arrowheads and javelin tips, as well as axes and daggers with or without a "button" feature. Hoe-shaped tools likely pertain to agricultural activities. Additionally, a unique stone artifact with a sculptural representation of a phallus was found. Many of the stone tools found in these cemeteries share similarities with those from the Neolithic Kama culture, indicating the continuity of stone tool-making traditions from the local Neolithic period. The presence of new stone-working technologies, such as the use of quartzite and the production of large plates for tool manufacture, along with the discovery of copper artifacts and evidence of emerging craft-based economy, suggests the introduction of innovative ideas associated with the influx of population from the south.

The distinctive pottery of the Kama Eneolithic is characterized by cup-shaped open forms with round bottoms. The clay paste contains organic impurities, and the rims of most vessels are either outwardly flared or inwardly beveled. The primary decorative elements consist of comb stamps and oval depressions, with the most typical ornamental compositions including horizontal zigzag friezes and diagonal impressions of comb stamps, as well as belts of oval pits. These compositions are sparsely placed on the vessel surface. The ceramics of the Kama Estuary Eneolithic display similarities to the pottery traditions of the Bor culture.

Recent studies have dated the Kama Estuary Eneolithic to 4600–4000 BCE. The formation of this cultural group is attributed to the influence of Khvalynsk traditions in the forest zone. It is regarded either as a peripheral northern component of the Khvalynsk culture or as a peripheral southern extension of the Bor culture. In either case, the material culture of the Kama Estuary Eneolithic demonstrates a mixed nature, combining traits from both the Khvalynsk and Bor cultures (Chizhevskiy and Shipilov, 2021; 2018).

##### Murzikha-2 burial site

The Murzikha-2 cemetery was situated on two promontories of the second terrace on the left bank of the Arkharovka River, a left tributary of the Kama River. Currently, the site is submerged under the reservoir formed by the Kama River bridge dam. The majority of the burials at Murzikha-2 date to the Late Bronze Age and Early Iron Age. Eneolithic burials were discovered by Andrei Chizhevsky in 1995, located approximately 35-40 meters from the modern shoreline in the central part of the necropolis.

During the excavation, a total of 18 burials were uncovered, arranged in three rows with 3-6 burials each, along with some individual burials. The rows extend in the northeast-to-southwest direction.

Out of the 18 burials, 12 exhibited identifiable grave pits, which were predominantly round or quadrangular in shape. The depth of the burials was generally shallow, ranging from 4-30 cm, with an average depth of 11 cm.

Approximately two-thirds of the burials were collective, containing two to four individuals. All burials were inhumations, although some individuals were found with evidence of prior cremation within the graves. Ocher was observed in many of the grave pits. The skeletons were positioned in either a sitting or crouched position, with bent legs. Based on the best-preserved skeletons, it can be inferred that the deceased were placed in a sitting position during burial, with their legs half-bent, hands resting on the knees with forearms, and their heads bowed onto their chests.

The orientation of the buried individuals varied, but an eastern direction predominated. In collective burials, two different arrangements were observed: 1) in burials with two or four individuals, the skeletons were placed in a single row, shoulder to shoulder, all facing the same direction; 2) in burials with three individuals, the skeletons were oriented with their heads facing different directions, while their feet pointed towards the center of the grave pit.

The burial inventory of Murzikha-2 was abundant and diverse, comprising items crafted from stone, bone, horn, and copper. Stone artifacts included woodworking and cutting tools, tools for leather processing, weapons and hunting implements, as well as tools associated with land cultivation. Woodworking and cutting tools were the most prevalent category. Military equipment was also abundant, with flint arrowheads discovered in seven burials, along with axes and daggers. The bone inventory was similarly diverse, encompassing weaponry, hunting tools, tools for skin processing, and fishing implements. Vessels recovered from the burials exhibited comb ornamentation, often arranged in belts of zigzag and oblique lines.

Ornaments were found in 13 burials. The majority of these ornaments (12 burials) consisted of oval, round, and sub-quadrangular pendants made from green phyllite, greenish and gray-black slate, featuring drawings such as parallel lines and horizontal zigzags. Bone and amber pendants were also present. Notably, two graves within the cemetery yielded copper ornaments (Chizhevskiy and Shipilov, 2021; 2018; Chizhevskiy, 2008).

The chronological attribution of the site is based on 8 new radiocarbon dates and more than 10 previously published determinations (Chizhevskiy and Shipilov, 2021), according to them the burial ground is dated to 4700–4200 calBCE. However, δ15N and δ13C reveal a fish-based diet of the population, and probability intervals of dates obtained from the same grave do not always overlap. These observations may reflect a freshwater reservoir effect.

###### Burial 102, individuals 1A (individual ID I8448, male) and 1G (I8449, male)

**Burial 102** (individual IDs I8448 and I8449) yielded a variety of pendants crafted from greenish, gray-black, and white serpentine, ranging in size from large (4.5 cm) to small (1.5 cm). Furthermore, within the same grave, a sculptural depiction of a bird's head, possibly that of a goose or swan, was unearthed (Chizhevskiy, 2008). The ***skeleton 1A*** from **burial 102** is dated to 4358–4256 calBCE (5470±25 BP, PSUAMS-8281), and ***skull G*** from the same burial is dated to 4547–4373 calBCE (5655±25 BP, PSUAMS-8338).

###### Burial 104 (individual ID I8450, male)

Within **burial 104** (individual ID I8450) three harpoons were found, along with another figurine in the shape of a bird's head (Chizhevskiy, 2008). The skeleton is dated to 4681–4463 calBCE (5730±30 BP, PSUAMS-8282).

###### Burial 118, individual 3 (individual ID I8452, female)

From **burial 118** (individual ID I8452), an auroch’s (*Bos primigenius*) tooth was recovered, along with a sculptural representation of a phallus. The phallus, made of silicified limestone, measured 26.7 cm in length. Similar models made of wood, antler, and bone are characteristic of the Volosovo culture (Chizhevskiy, 2008). The skeleton is dated to 4532–4358 calBCE (5615±30 BP, PSUAMS-8283).

###### Burial 128, skull 1 (individual ID I8456, male) and individual 2 (I8455, male)

**Burial 128** (individual IDs I8456 and I8455) contained two axes made of soft limestone, both featuring a perforation. One of the axes had a boat-shaped form with an asymmetrical eyelet, while the other was rhomboid-shaped and crafted from a thin plate. Additionally, a dagger made of slab flint was present in the burial. Of particular significance is the discovery of a fragment of a cattle shoulder blade, representing the first reliable evidence of domesticated animals in the Volga-Kama region. This finding provides evidence of the nascent development of a productive economy in the area (Chizhevskiy, 2008). The burial is dated to 4446–4274 calBCE (5515±30 BP, PSUAMS-8293) based on a human bone/tooth of individual I8455.

###### Burials: 94, individual 2 (individual ID I11842, female), 119 (I8453, female), 123, individual 1 (I8454, female), 130, individual 1 (I8457, male), and 131 (I11841, male)

For these burials we do not have detailed archaeological descriptions. According to available data, the burial rites and grave goods from these burials share the traits described above. **Burial 119** is dated to 4653–4452 calBCE (5700±30 BP, PSUAMS-8335) and **burial 123** - to 4446–4346 calBCE (5550±25 BP, PSUAMS-8292); both results are based on human bones/teeth. A significant freshwater reservoir offset is expected in all dates from Murzikha-2 site.


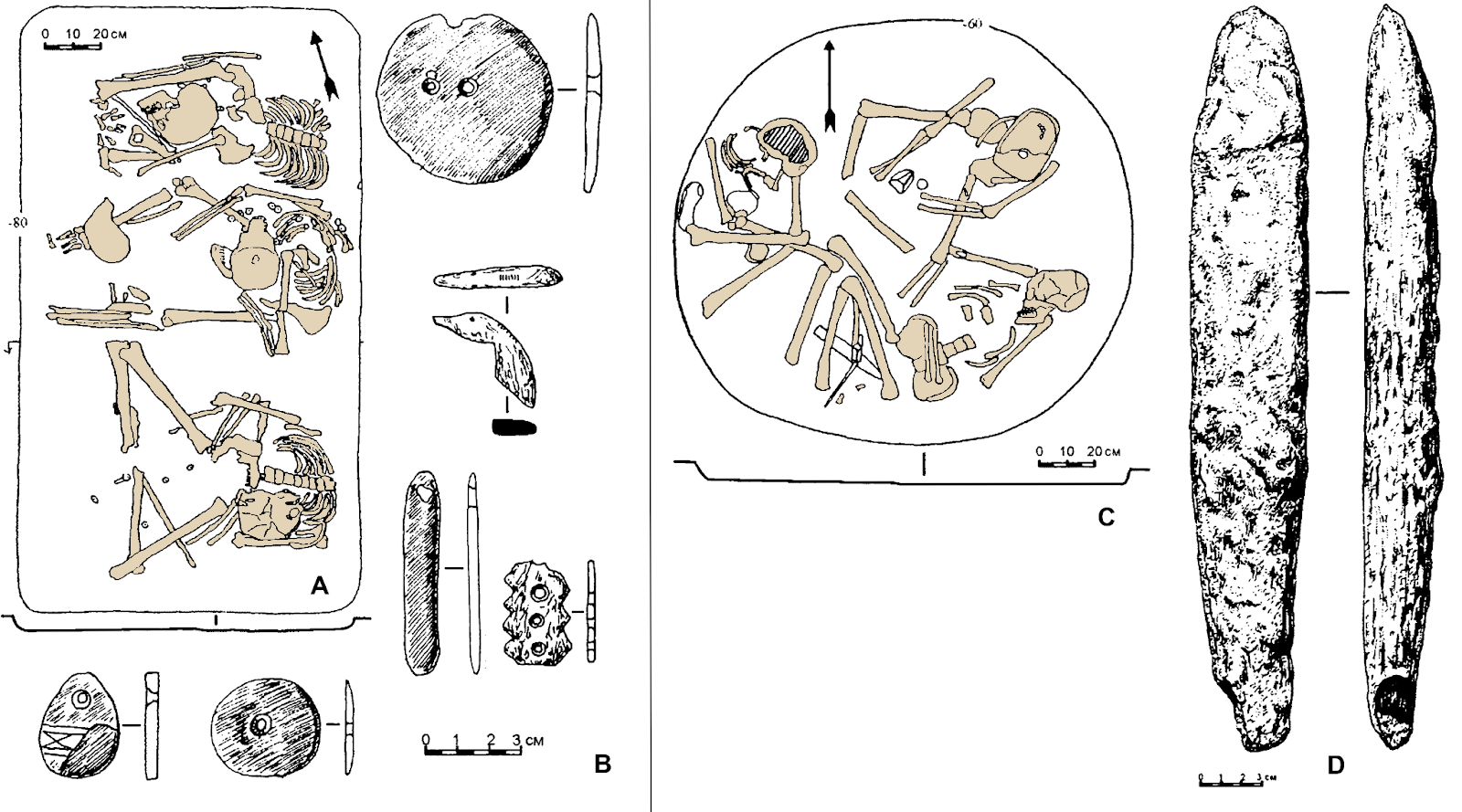


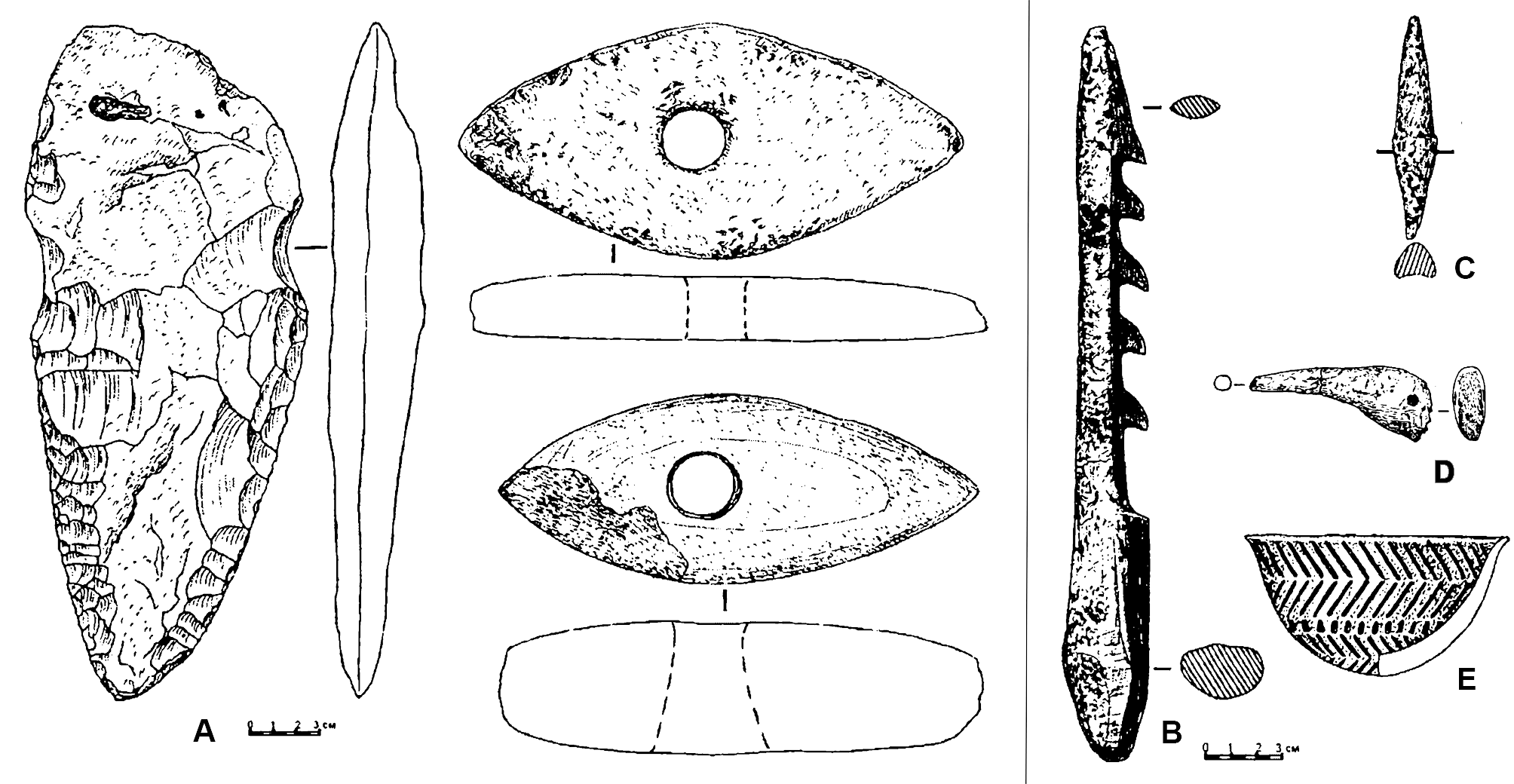


***Figure S63.*** *Eneolithic burials 102 and 118 at the Murzikha-2 site, & Grave goods from Eneolithic burials at the Murzikha-2 site.* ***Top - Eneolithic burials 102 and 118 at the Murzikha-2 site*** ***A*** *- burial 102;* ***B*** *- grave goods from burial 102;* ***C*** *- burial 118;* ***D*** *- limestone phallus sculpture from burial 118 (by Chizhevskiy, 2008, modified)**.* ***Bottom - Grave goods from Eneolithic burials at the Murzikha-2 site.*** ***A*** *- a dagger and stone axes from burial 128;* ***B*** *- harpoon from burial 104;* ***C*** *- antler arrowhead from burial 131;* ***D*** *- antler model of bird’s head from burial 104;* ***E*** *- pottery vessel from grave 124 (by Chizhevskiy, 2008, modified).*

#### Volga Late Eneolithic (Russia_LateVolga_Eneolithic)

Cultural evolution during the Late Eneolithic in the Middle Volga region was shaped by interaction between a population of steppe origin, characterized by collar ceramics, and descendants of the local Neolithic population. Three distinct cultural groups can be identified, distinguished by their unique ceramic characteristics and origins.

Group 1 comprises sites of the Tok and Chekalino-4 types, where pottery vessels display a gently curved profile, are tempered with crushed shells, and adorned with short to moderately long comb imprints and pits. The cultural traditions of this group likely share a common Volga-Kama Neolithic background, with its development associated with the influence of steppe populations who introduced collared pottery to the region.

Group 2 is represented by sites of the Lebyazhinka type, which later evolved into the "Volosovo-like" tradition observed in Gundorovka. The pottery in this group is tempered with feathers and decorated with moderately long comb imprints and cord impressions. These vessels exhibit similarities to the collared ceramics of the forest-steppe Eneolithic, while also demonstrating typical characteristics of the Volosovo culture in the Middle Volga forest zone. The origins of this group can also be traced back to the Volga-Kama culture, but it shows a stronger influence from the Samara culture compared to Group 1.

Group 3 includes sites in the Samara River Valley attributed to the Turganik type and sites in the Sok River Valley associated with the Gundorovka type, also known as the "inner rib pottery" type. Pottery vessels within this tradition feature inner ribs and are tempered with crushed shells and feathers. They are decorated with imprints from fine-toothed combs or plain stamps, as well as hatching and pits. The pottery tradition of this group exhibits similarities to the Serednii Stih, Khvalynsk, and Ivanovka phases of Samara cultures. Vessels with inner ribs are probably derived from those found in the forest-steppe Middle Eneolithic.

Radiocarbon dating places the Late Chalcolithic Mid-Volga pottery within the time frame of 4250–3500 BCE (Korolev, 2021; Shalapinin, 2018).

##### Maksimovka-1 burial ground

The flat burial ground is located on the northwestern outskirts of the village Maksimovka, 480 m east-northeast of the confluence of the Syezhaya and Samara Rivers, a left tributary of the Volga River. The site was discovered in 2017 during salvage excavations, by Viktor Tsibin (Tsibin and Shalapinin, 2018; Korolev and Shalapinin, 2020; Korolev et. al., 2021).

###### Burial 1 (individual ID I8446, male)

In **burial 1** (individual ID I8446), 3 skeletons were arranged in a linear formation along the northwest-southeast axis, with numbering from east to west. Unfortunately, because of the paucity of records, we were unable to determine which of the skeletons was sequenced for this study.

***Skeleton 1*** was positioned in the southeastern part of the burial. The deceased was laid supine, with the upper body oriented towards the northeast and the legs bent at the knees, pointing westward. The skull fragments were severely damaged and scattered around the chest and pelvis regions. The bones of the chest, arms, and pelvis were also heavily fragmented. The relatively well-preserved right humerus was aligned along the east-northeast to west-southwest axis. A flint scraper was discovered near the left knee of this skeleton. South of the femur, a stone wringer was found, and two points were located south of the pelvis.

***Skeleton 2*** occupied the central position in the burial and was oriented towards the northeast. The skeleton was supine, with the legs bent at the knees and collapsed towards the west. The skull was situated between the pelvic and chest bones. The shoulders were relatively well-preserved and maintained their anatomical order. The ulna and radius bones, extending along the body, remained intact from the arms. A sandstone pendant was found within the bones of the humerus. A fragment of a point was present in the area of the left shoulder. A flint hole was discovered to the south. A bone tool was located in the vicinity of the pelvis and knees.

***Skeleton 3*** was situated in the northwestern part of the burial, with the head oriented towards the northeast. The individual was buried in a supine position, with the legs bent at the knees and falling to the east. The skull was positioned near the chest region, with the crown facing northeast. The shoulder girdle, thorax, and pelvis were in a deteriorated state. The arm bones were extended along the body. Between ***skeleton 3*** and ***skeleton 2***, a fragment of an unadorned vessel wall was discovered.


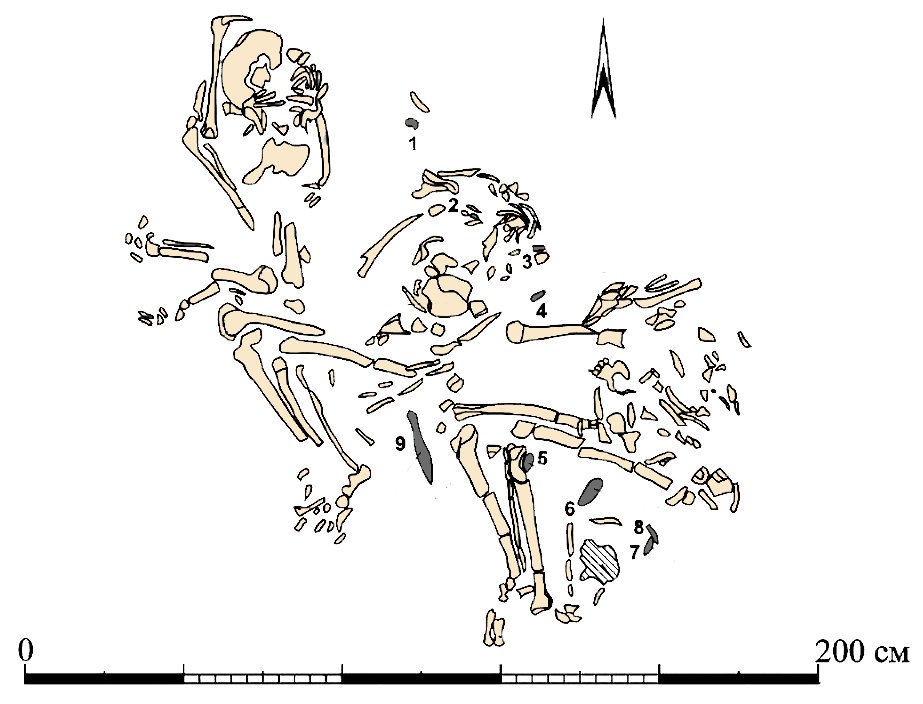


***Figure S64.*** *Burial 1 at the Maksimovka-1 site (by Tsibin and Shalapinin, 2018, modified).*

The grave goods found in the burial include a bone tool, a wringer, a sandstone pendant, flint scrapers, piercers, and arrowheads with straight or notched attachments. This burial assemblage shares similarities with the funerary practices observed in the Khvalynsk Eneolithic culture. The presence of arrowheads is a characteristic feature of the Caspian and Altata assemblages of the Volga steppe (Tsibin and Shalapinin, 2018).

The Late Eneolithic **burial 1** has been dated to 3946–3711 calBCE based on a bone/tooth of the sequenced individual (5025±25 BP, PSUAMS-5923).


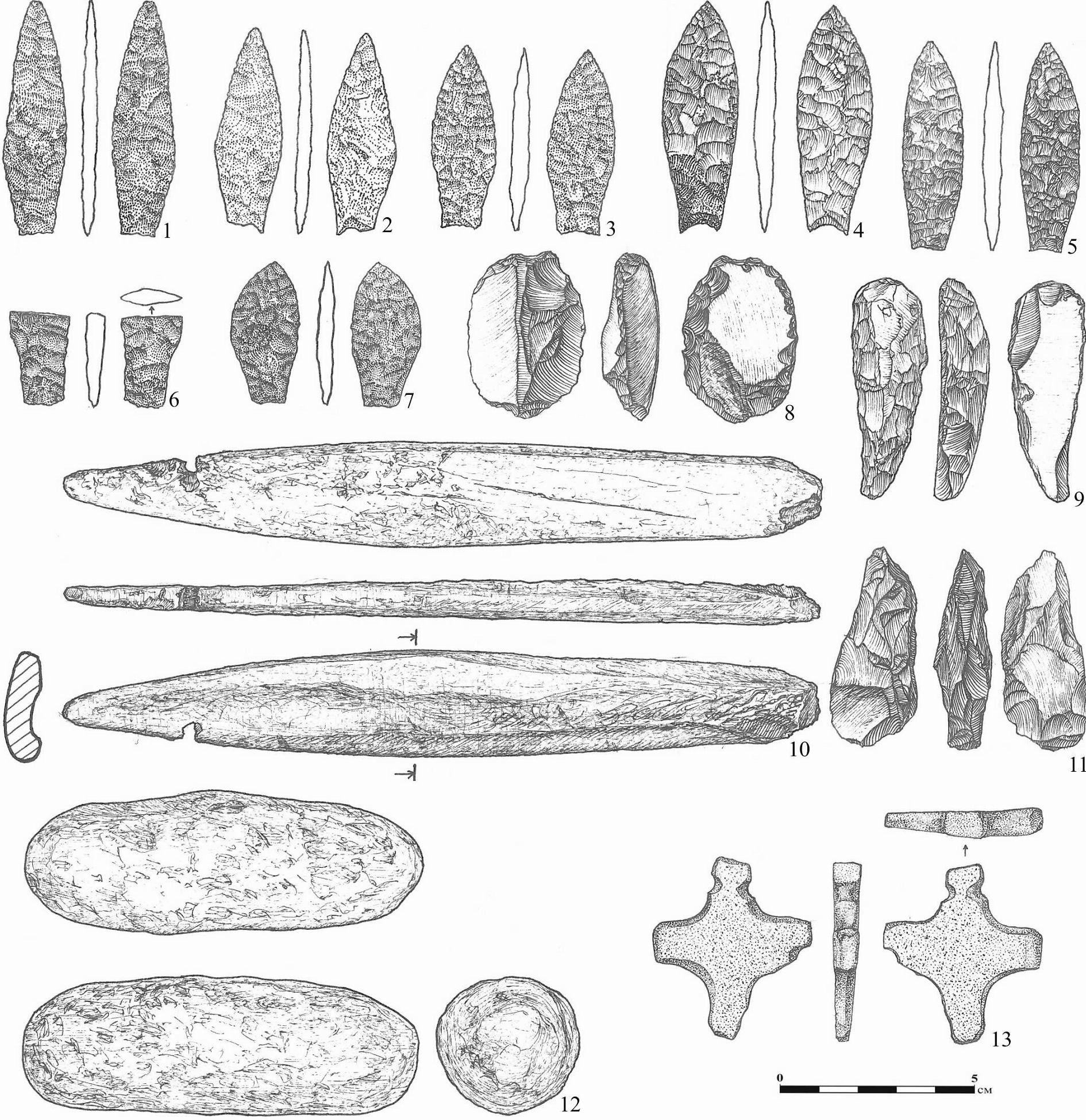


**Figure S65.** Burial 1 at the Maksimovka-1 site. Grave goods (by Tsibin and Shalapinin, 2018).

###### Unidentifiable burial (individual ID I6904, male)

Another burial from Maksimovka-1 (individual ID I6904) lacks detailed archaeological description. The skeleton is dated to 4153–3962 calBCE (5205±25 BP, PSUAMS-4255).

### Volga-Oka Region

#### Lyalovo Pit-Comb Ware Late Neolithic culture (Russia_UpperVolga_Lyalovo_LN)

The Lyalovo culture was initially defined by Boris Zhukov in 1925 following excavations conducted at the Lyalovo site near Zelenograd in the Moscow region. According to contemporary understanding, the term encompasses Late Neolithic sites within the Volga-Oka interfluve. The Lyalovo tradition is one of several that contribute to the Pit-Comb pottery area, which extended across the forested zone from the Baltic to the Mid-Volga. Broadly, Lyalovo sites are estimated to date back to 7000–6000 BP.

The categorization of the Lyalovo culture primarily relies on distinct characteristics of its pottery tradition. Handcrafted vessels feature rounded (occasionally slightly pointed) bottoms, incorporating crushed granite or sand into clay. The outer surface of the pottery exhibits a continuous zonal ornamentation comprising pits created using a belemnite, various impressions from a comb stamp, and "lunar" impressions. Tool assemblages consist of flint scrapers, knives, and hunting equipment such as arrowheads and spearheads, among others. These tools were produced from flakes, employing double-sided retouching techniques. While bone tools have not been well preserved, some arrowheads and perforators have been recovered. The economy of the Lyalovo culture revolved around hunting (elk, beaver, deer, wild boar, upland and waterfowl species, etc.), fishing, and gathering. Domesticated dogs were also present within the population.

Lyalovo habitation sites are typically located along riverbanks and lakeshores. Extensive elongated houses, both above-ground structures, and semi-dugouts with gable roofs, have been documented. In the vicinity of settlements, ground burials have been discovered, where bodies were interred in a supine position. Grave goods are relatively scarce, primarily comprising tools and ornaments. Four distinct periods have been identified in the development of the Lyalovo tradition, differentiated by specific details of vessel ornamentation: the archaic, early, developed, and late periods.

The Lyalovo culture succeeded the Upper Volga Early Neolithic culture in the Volga-Oka region around 5200–5000 BCE and gradually gave way to the Volosovo Late Neolithic-Eneolithic culture approximately 4100–4000 BCE. Some researchers propose a period of coexistence between the Lyalovo and Volosovo traditions, lasting for several hundred years, with both traditions observed occasionally at the same sites (Gurina and Kraynov, 1996; Engovatova, 2011, 2017; Berezina et al., 2021).

##### Lyalovo phase of the Saktysh archaeological site complex

The Sakhtysh peat bog is situated in the Ivanovo region. It gives rise to the Koika River, which is a left tributary of the Klyazma (Nerl) River. The swamp covers an approximate area of 50 square kilometers and formed in the location of a shallow post-glacial paleolake. A comprehensive scientific investigation of the Sakhtysh archaeological complex commenced in 1962 under the auspices of the Upper Volga expedition, affiliated with the Institute of Archaeology of the USSR Academy of Sciences (now Russian Academy of Sciences, RAS). The project was led by Dmitry Krainov (until 1993) and subsequently by M.G. Zhilin. This endeavor persisted nearly uninterrupted for over forty field seasons, concluding in 2006. During this period, fifteen ancient human habitation sites were identified.

Among the identified sites, five are long-term settlements, namely Sakhtysh-1, 2, 2a, 7, and 8. Additionally, six seasonal sites, including Sakhtysh-3, 4, 9-11, and 14, were discovered, along with four locations yielding individual artifacts, namely Sakhtysh-5, 6, 12, and 13. All of these sites are situated on low, indistinct banks of former river channels and paleolakes, and the diameter of the investigated area is 3.5 kilometers. A total of 149 burials from various epochs were unearthed. Chronologically, these burials can be assigned to the Neolithic (20) and Eneolithic periods (128), and the Late Bronze Age (1) (Kostyleva and Utkin, 2010). Recently, Sakhtysh burials were comprehensively studied with respect to their chronology (Dolbunova et al. 2017), diet (Meadows et al., 2024), and genetics (Allentoft et al., 2024).

The Lyalovo burial ground at the Sakhtysh-2a site functioned at the initial stage of the development of the Lyalovo culture.

***Figure S66.*** *Pit-Comb Lyalovo burial site at Sakhtysh-2a.* ***1*** *- male burials;* ***2*** *- female burials;* ***3*** *- child’s burial;* ***4*** *- unidentified burial;* ***5*** *- grave rows. Sequenced individuals are marked by red arrows. Burial 11 marked by blue arrow was attributed to the Lyalovo culture by Kostyleva and Utkin (2010) and later reattributed to the Corded Ware Fatyanovo culture according to its burial rite (Kostyleva and Utkin 2025), but we attribute it to the Volosovo population, based on the genetic profile of the buried individual I8419* *(by Kostyleva and Utkin 2010, modified).*

The graves are of a rectangular shape with rounded corners or elongated ovals when viewed from above. In cross-sectional and longitudinal views, they appeared as trough-shaped recesses, often displaying an uneven bottom. The dimensions of the graves were determined based on the height of the deceased individuals. No evidence of internal grave structures was discovered within the burials.

The skeletal remains of the interred individuals were remarkably preserved, retaining their anatomical arrangement. However, in some cases, small bones were absent or displaced due to rodent activity. The positioning of the bones suggests that certain corpses were tightly swaddled during burial. In adult individuals, the swaddling primarily encompassed the torso, while the legs remained unbound. This is evident from the unnatural narrowness of the upper portion of the postcranial skeleton, the pronounced compression of the chest, the close proximity of the hand bones to the chest, or their displacement onto the chest area. In burial 43, the child was completely swaddled from head to toe, possibly using animal skins. The sole instance of ocher usage in the funerary practice is discernible in burial 61, where a small area of dark pink paint was observed beneath the female skull.

In single graves, the deceased were laid out in a supine position with their limbs extended. The arm bones were either positioned parallel to the body or slightly flexed at the elbows, while the hands were placed on the abdomen. In burial 42, the left arm was extended, with the hand resting on the pelvic bones, while the right arm was bent at nearly a right angle and positioned on the belt area (Kostyleva and Utkin, 2010). Animal tooth appliques, including those from an elk and a bear, were discovered in three burials: 22 (1 ornament), 40 (1 ornament), and 43 (2 ornaments). The placement of these ornaments in relation to the skeletons did not follow a specific pattern (Kostyleva and Utkin, 2010).

###### Sakhtysh-2, burial 19 (individual ID I8413, female)

**Burial 19** was excavated in 1984. The borderline of the grave pit was documented at a depth of 55–60 cm. The pit displayed an approximately rectangular shape with rounded corners and a trough-shaped bottom. Its measurements were 2.05 × 0.65 m, with a depth of approximately 20 cm. The well-preserved skeletal remains of an adult anthropologically identified as male, but genetically determined as female, aged 20-25 years, were found lying supine, with the head oriented towards the southwest. The arms were flexed at the elbows, and the hands were positioned atop each other in the waist region. The bones of the right foot were absent, but one of its phalanges was discovered near the right knee. No accompanying grave goods were detected in association with the deceased (Kostyleva and Utkin, 2010). Burial 19 was dated to 5319-5072 calBCE (6265±38 BP, UBA-40003) by (Allentoft an al., 2024)
and to 5321-5127 calBCE (6275±30 BP, PSUAMS-14534) in the course of this research. The combination of the radiocarbon dates is 5319-5072 calBCE [R_combine: (6275±30 BP, PSUAMS-14534), (6265±38 BP, UBA-40003)].


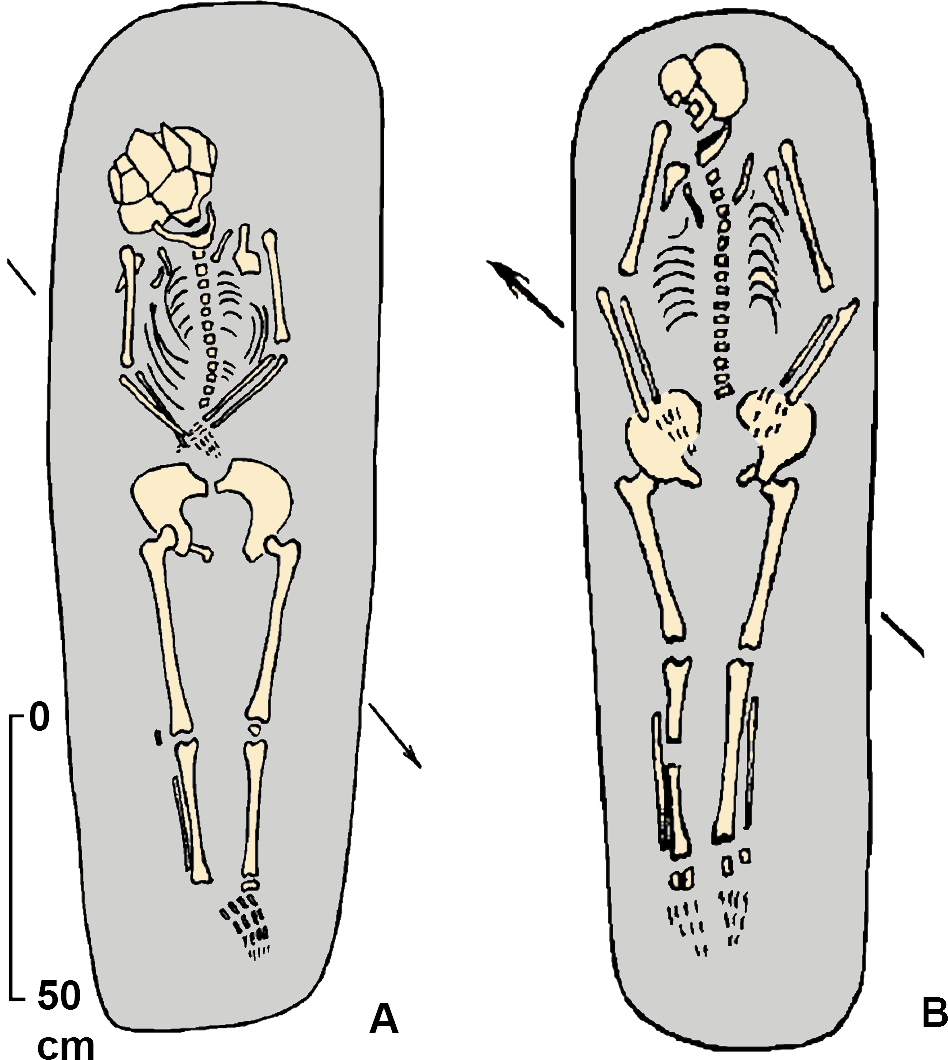


***Figure S67.*** *Pit-Comb Lyalovo burials at the Sakhtysh-2 site.* ***A*** *- burial 19;* ***B*** *- burial 20 (by Kostyleva and Utkin 2010, modified).*

###### Sakhtysh-2, burial 20 (individual ID I8412, female)

**Burial 20** was excavated in 1985. This burial pertained to a young female (as was determined both anthropologically and genetically) and was situated within an approximately rectangular grave with a trough-shaped bottom, recorded at a depth of 60 cm. The dimensions of the pit measured 1.8 × 0.8 m, with a depth of roughly 20 cm. The infill material consisted of gray loam devoid of any significant findings. The skeleton was well-preserved and positioned in a supine position, facing towards the northeast. The arms were flexed at the elbows, and the hands were clasped on the abdomen. The skull and leg bones exhibited light traces of ocher. No equipment or adornments were present within the burial (Kostyleva and Utkin, 2010). There is no radiocarbon date for this burial yet.

###### Sakhtysh-2a, burial 22 (individual ID I8439, female)

**Burial 22** contained a female (as was determined both anthropologically and genetically) aged 20-25 years lying in a prone position with an extended body oriented to the south-southeast. The arm bones were positioned alongside the body, and the hands were placed under the pelvis. The grave contained several items, including:

1. A wide double-bladed dagger with a lenticular cross-section;
2. A narrow dagger with a hilt adorned with a sculptural depiction of a human face;
3. A harpoon with a short triangular socket, two large beak-shaped teeth, and a point that has been lost over time (Kostyleva and Utkin, 2010).

The skeleton is dated to 4984-4784 calBCE (5980±30 BP, PSUAMS-14538).


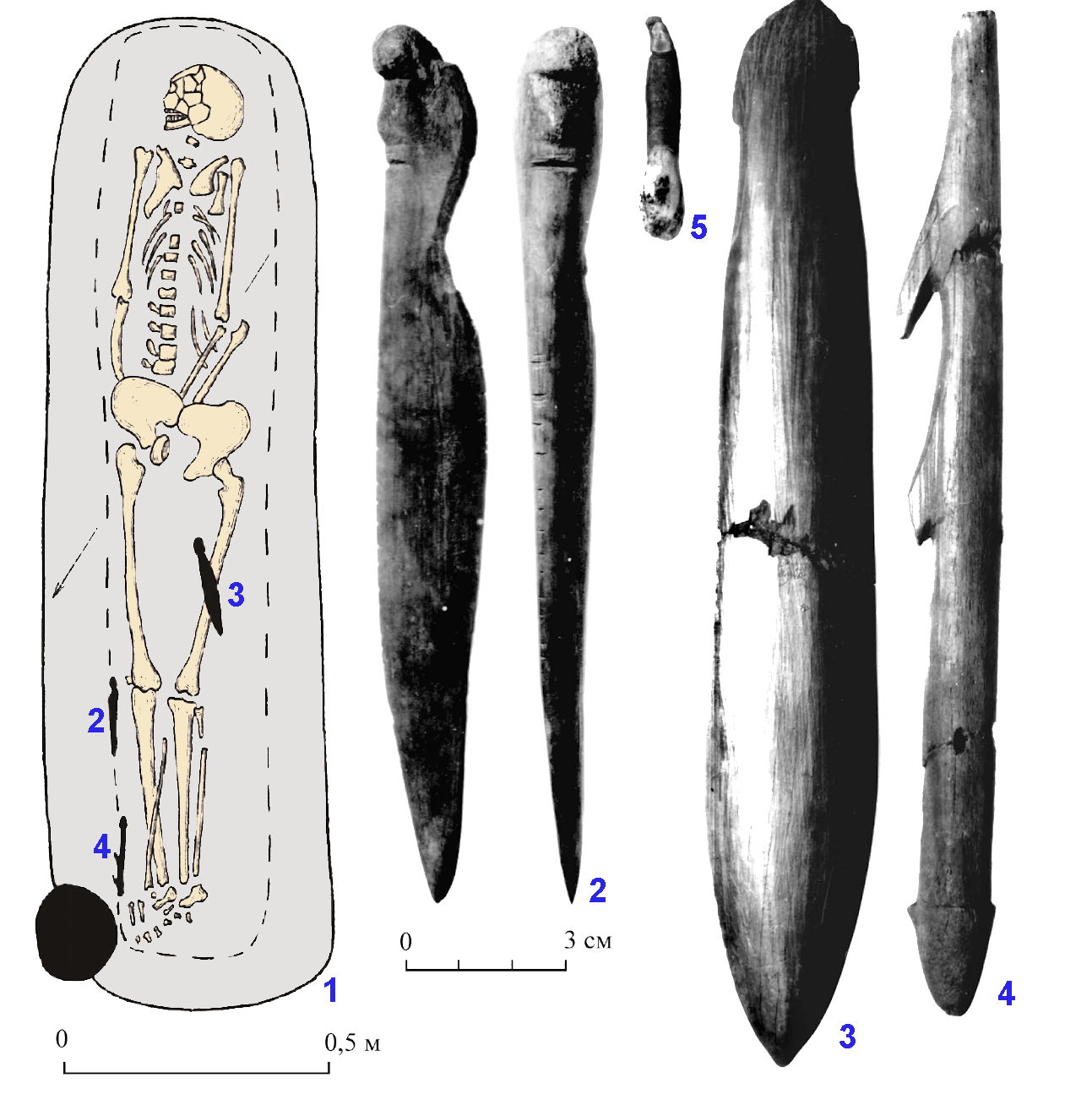


***Figure S68.*** *Burial 22 at Sakhtysh-2a.* ***1*** *- skeleton;* ***2, 3*** *- daggers;* ***4*** *- harpoon;* ***5*** *- pendant (****2-5*** *- tools made of bone) (by Kostyleva and Utkin 2010, modified).*

###### Sakhtysh-2a, burial 40 (individual ID I8410, male)

In male **burial 40**, a short segment from a metapodium of an elk was found parallel to the left humerus, at a certain distance from it (Kostyleva and Utkin, 2010). The skeleton was dated to 5474-5231 calBCE (6393±39 BP, UBA-39997) by (Allentoft et al., 2024) and to 5617–5477 calBCE (6585±35 BP, PSUAMS-9405) in the course of this research project. The intervals have poor agreement and the combination of the dates fails.

###### Sakhtysh-2a, burial 42 (individual ID I8411, male)

**In burial 42 t**he skeleton was laid in the supine position and oriented with his head to the southeast. The left arm was extended, with the hand resting on the pelvic bones, while the right arm was bent at nearly a right angle, and the hand was placed on the waist. The skeleton is dated to 5323–4610 calBCE (6060±150 BP, GIN-6586) and 5476-5052 calBCE (6317±91, UBA-39998). The combination of the radiocarbon dates is 5371-5001 calBCE [R_combine: 5323–4610 calBCE (6060±150 BP, GIN-6586), 5476-5052 calBCE (6317±91, UBA-39998)].

###### Sakhtysh-2a, burial 43 (individual ID I8438, male)

In **burial 43**, the skeleton was laid in the supine position and oriented with his head to the southeast. There is no radiocarbon date for this burial yet. Some ocher pieces were found in the infill of the grave and two pebbles were unearthed near the skull and the right knee of the deceased. Kostyleva and Utkin (2010) suggest that they were used to secure the edge of the burial "shroud" made of animal skin.

###### Sakhtysh-2a, burial 61 (individual ID I8416, female)

**Burial 61** contained the remains of three individuals. The upper portion of the female skeleton was found in an extended supine position, with the arms positioned alongside the torso and the hands resting on the pelvic bones. On top of the pelvis lay the skull of an infant, approximately two years old. The skull was positioned with the vault facing upwards, and it is assumed that the missing skeleton of the infant was lying prone between the woman’s thighs.

In burial 61 the only instance of intentional ocher usage in the burial ritual was observed. A small dark pink spot was found beneath the female skull. The ochre was applied so densely under the head of the deceased that, after the removal of the skull, the entire left side of the skull cap was soaked with the pigment.

Within burial 61, three bone tools were unearthed:

1. A piercing tool crafted from a fragment of a narrow animal rib;
2. A knife designed for cutting fish;
3. A long rod-shaped object measuring 19 cm in length and featuring a diameter ranging from 0.8 to 1.4 cm.

The sequenced skeleton is dated to 5633–5483 calBCE (6650±35 BP, PSUAMS-9406).

***Figure S69.*** *Pit-Comb Lyalovo burials at the Sakhtysh-2a site.* ***A*** *- burial 40;* ***B*** *- bone tool (kochedyk) from burial 40;* ***C*** *- burial 42 (by Kostyleva and Utkin 2010, modified).*

***Figure S70.*** *Pit-Comb Lyalovo burials at the Sakhtysh-2a site.* ***1*** *- burial 43;* ***2*** *- burial 61;* ***3, 4*** *- bone tools from burial 61 (by Kostyleva and Utkin 2010, modified).*

#### Volosovo Eneolithic culture (Russia_Volosovo_Eneolithic)

The Volosovo archaeological culture represents a Late Neolithic-Eneolithic tradition found in the forest and forest-steppe regions of the central Russian Plain and the Middle Volga region. It was initially described by Vasiliy Gorodtsov in the early 20th century based on excavations conducted at the Volosovo and Panfilovo occupation sites on the Oka River. These sites revealed similar cultural assemblages of the Neolithic and Eneolithic periods. From the 1920s to the 1950s, there was a debate regarding the chronological position of the Volosovo culture and whether it should be classified as Neolithic or Eneolithic. According to recent understanding, the early Volosovo sites are attributed to the end of the Neolithic period, while the later sites are considered Eneolithic.

The occupation sites of the Volosovo culture include both permanent and seasonal settlements. The typical dwellings were sunken-floor buildings of approximately square shapes, interconnected by passages, and constructed with a frame-pillar structure. Inside the dwellings, a series of open hearths, sometimes up to 11 in number, were located in the center and along the walls. Burials, both individual and collective (involving up to 16 individuals), were found within or near the occupation areas. The bodies were laid in supine, prone, or, sometimes in the later phases, in crouched positions and were occasionally adorned with ocher. Bonfires and bear skulls were also observed between the burials. Grave goods consisted of ornaments made of bone, stone, amber, and occasionally of tools. Pottery vessels had rounded or flat bottoms and were made with the inclusion of shells (in the Central Russian Plain) or plant materials (in the Middle Volga region, and in the Central Russian Plain during the later phase of the culture) in the clay. The pottery was decorated on the entire surface or occasionally on the rim. Stone tools were made from flakes or polished stone, while bone tools included harpoons, fishing hooks, arrowheads, and pottery stamps. Ornaments included figurines depicting humans, animals, and birds, crafted from flint, bone, and antler. Amber pendants were widespread even in the eastern regions of the culture.

The economy of the Volosovo culture was based on fishing and hunting. In the later stages, evidence of copper items and indications of copper production was recorded. The Volosovo culture did not remain static throughout its existence. In the 1970s, Andrei Nikitin proposed distinguishing two cultures within the Volosovo tradition: "Volosovo-1" and "Volosovo-2." He associated the first with the Neolithic period, suggesting its origins in northwestern sources from the Baltic and upper Dnieper regions, while he connected the second with the Middle Volga region and attributed it to the Bronze Age. Although this claim was initially criticized, modern researchers tend to support Nikitin's hypothesis. Elena Kostyleva, for example, suggests that the early development of the Volosovo culture can be traced back to the Volga-Oka interfluve and East Baltic Neolithic and to the arrival of population groups from the Eastern Baltic who left burial sites containing amber (Kostyleva and Utkin, 2018). In the late and final stages of the Volosovo culture, noticeable influences from the eastern parts of the East European plain are evident in ceramics and jewelry.

The population of the Volosovo culture is believed by archaeologists to have descended from the bearers of the Pit-Comb Lyalovo and Valdai cultural traditions in the central part of the region, as well as from the Volga-Kama culture population in the Mid-Volga region. During its later stages, the Volosovo was impacted by the Fatyanovo and Balanovo Corded Ware cultures, as well as the Abashevo culture in certain regions. The Volosovo substrate is also suggested to have influenced cultures such as the Chirki, Pozdnyakovo, Fatyanoid, and other cultures of the Central part of the East European plain and the Volga region (Kuz’minykh, 2006; Nikitin, 2021).

##### Volosovo phase of the Saktysh archaeological site complex

A group of 11 burials was excavated in an area close to a Late Volosovo dwelling pit. All of the burials discovered at the Lyalovo-Volosovo site were situated within the cultural layer of the settlement, characterized by a high concentration of organic material. No discernible grave pits were identified, suggesting that the deceased individuals were likely placed directly on the cleared ground surface and subsequently covered with soil. These Volosovo burials were predominantly individual in nature, and researchers have observed a pattern indicating rows of graves. In close proximity to the burials, two treasure hoards containing amber ornaments and arrowheads were unearthed. Additionally, archaeological interpretations have identified traces of structures believed to be sanctuaries.

In the majority of the burials, the skeletons were found in supine positions, with their arms extended alongside their bodies. Some skeletons exhibited slight bending of the arms at the elbows, with the hands positioned near the abdomen (Kostyleva and Utkin, 2010).

Based on the positioning of the clothing adornments, researchers have been able to reconstruct the attire worn by the Volosovo population. It appears that their costume consisted of a long, closed jacket with a hood. The jacket was intricately embroidered with amber objects, which were found on the chest, the hem's edge, sleeves, and framing of the hood.


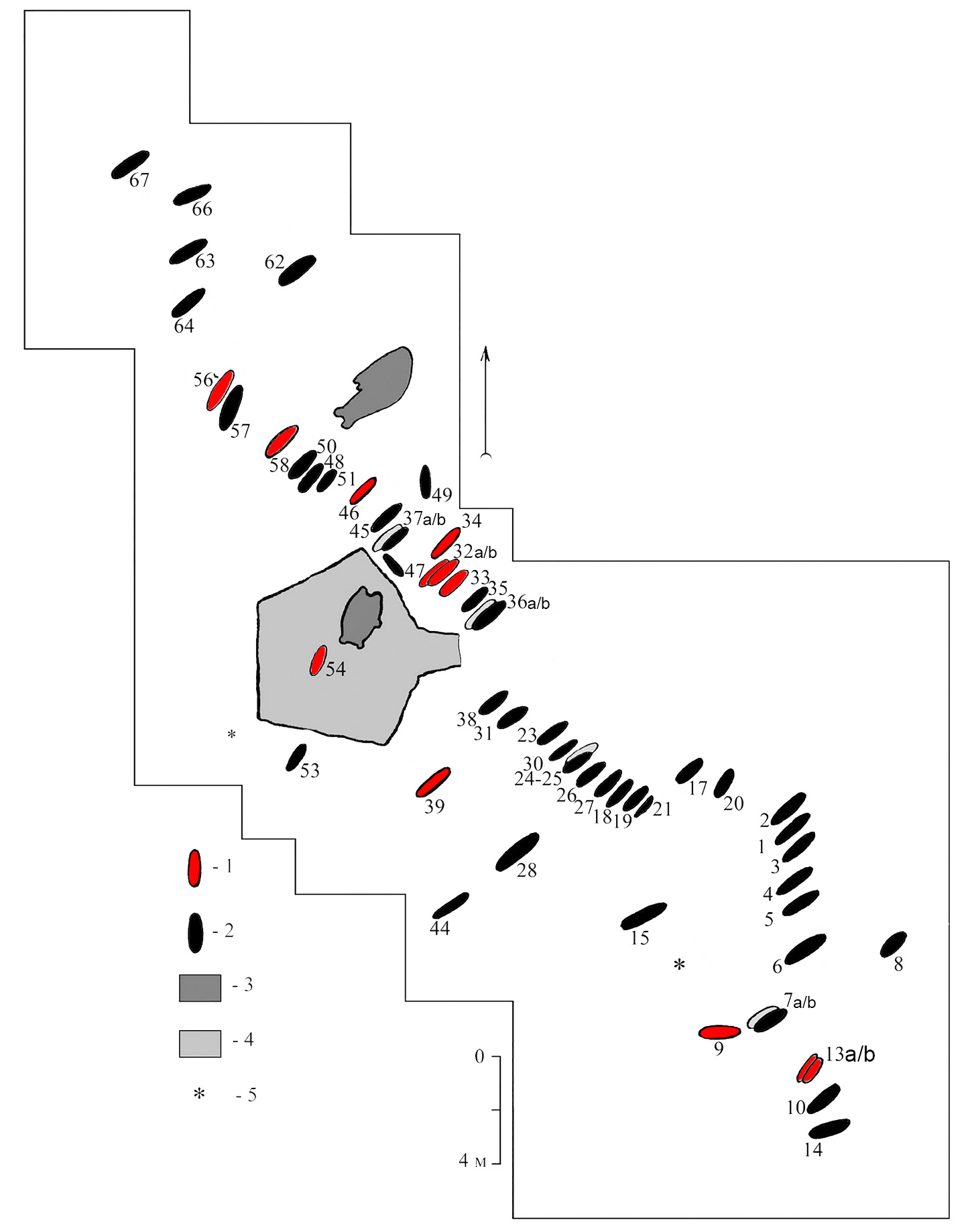


***Figure S71.*** *Volosovo burials at the Sakhtysh-2a site.* ***1*** *- burials with sequenced individuals;* ***2*** *- other burials;* ***3*** *- ritual (?) pits;* ***4*** *- ritual (?) construction;* ***5*** *- hoards (by Kostyleva and Utkin 2010, modified).*

###### Sakhtysh-1, burial 8 (individual ID I8407, female)

**Burial 8** was unearthed at a depth of 40-50 cm. The grave pit borderline has not been distinguished. An adult anthropologically identified as male, but genetically determined as female, aged 40-45, was laid in a crouched position oh his right side, oriented to the northwest. No grave goods were found (Kostyleva and Utkin, 2010). The burial is dated to 3710-3635 calBCE (4890±25 BP, PSUAMS-14533).

***Figure S72.*** *Burial 8 from the Sakhtysh-1 site (****A****) and burial 11 from the Sakhtysh-2a site (****B****) (by Kostyleva and Utkin 2010, modified).*

###### Sakhtysh-2, burial 12 (individual ID I12494, male).

Collective **burial 12** included four skeletons and was located in the cultural layer at a depth of 40-60 cm. Although the grave pit itself was not discernible, its boundaries were clearly demarcated by an ocher spot. Just above the ocher spot, accumulations of large charcoal pieces were recorded, suggesting the presence of a ritual fire. The individual I12494 is labeled as one of the skeletons from **burial 12**, without specification.

***Skeleton A*** belonged to a male individual estimated to be 30-35 years old. Positioned in the western part of the ocher spot, this skeleton was laid on the right side in a slightly crouched position, facing south-southeast. The skull was found in proximity to a severely crushed pelvis. The left leg lacked bones, except for a fragment of the knee joint, while the bones of the right foot were absent, and the thigh was displaced in relation to the lower leg. The left arm extended along the body and fell behind the back, while the right arm was bent with the hand placed near the cervical vertebrae. Beneath the skeleton, a pile of 213 beads made of bird bones was discovered.

***Skeleton B***, a female aged 25-30 years old, was positioned in the eastern half of the ocher spot. This skeleton was laid on the right side in a slightly crouched position, with the head facing north-northwest and the face oriented to the southwest. The legs were spread apart, and the arms were bent at the elbows, with the left arm drawn toward the body and the right arm in the opposite direction. The hands were joined together, and the skull from ***skeleton A*** rested upon them. Additionally, a layer of thin bird bone beads (215 pieces) was present in the belt area.

***Skeleton C*** was situated in the northeastern part of the spot, positioned behind the female skeleton and slightly deeper. The anatomical arrangement of this skeleton was severely disrupted, with only portions of the ribs and spine, fragments of an arm and pelvic bones, and a part of a leg (without the lower leg bones and both feet) remaining. The skull was missing. It appears that initially the deceased, estimated to be a child aged 5-8 years old based on bone size, was laid stretched out on the back with the head facing north-northwest. Later, a slight "shift" towards the northeast caused the skeletal disorder. Three amber pendants, one button, and one bone pendant were found beneath the skeleton in various locations. Additionally, a couple of amber pendants discovered on the eastern outskirts of the ocher spot likely belong to this individual.

***Skeleton D*** was located between the skeletons of a male and a female, closer to the male and at a slightly greater depth. This skeleton, likely that of a teenager, was entirely fragmented, with only the spine, fragments of the pelvis, and one arm bone preserved. The remaining skeletal parts, including the skull, were missing. Four amber pendants and two buttons were found along and beneath the spine. One button lay on the fragments of the male skeleton's pelvis, another was positioned near the hand of the skeleton, and two pendants were discovered beneath a layer of beads.

It is highly probable that collective **burial 12** was formed through a two-stage process. Initially, a teenager and a child were interred in a paired burial, likely positioned supine with their bodies fully extended and their heads oriented to the north-northwest. Subsequently, after a relatively short period of time, the burial was disturbed by the interment of adult individuals. This disturbance resulted in the displacement of certain bones, including the skulls, which were expelled from their original positions. Furthermore, some of the accompanying ornaments fell into the backfill. The adults were buried simultaneously, with deliberate dismemberment observed in the male individual (Kostyleva and Utkin, 2010).

The human bones from the burial are dated to 3625-3376 calBCE [R_combine: (4705±25 BP, PSUAMS-9400), (4754±50 BP, UBA-39990)]. Based on this comparatively late chronological position, Meadows et al. (2024) attributed the burial to the transitional period from the Eneolithic to the Bronze Age.

###### Sakhtysh-2, burial 13 (individual ID I12496, female).

In **burial 13**, a solitary skeleton was found in a supine position. The right hand was placed alongside the body, while the left hand was slightly bent, with the hand resting on the pelvic bones (Kostyleva and Utkin, 2010). There is no radiocarbon date for this burial yet.

***Figure S73.*** *Burials 10 at Sakhtysh-2a (****A****) and 12 at Sakhtysh-2 (****B****), and amber buttons and pendants from burial 12 (by Kostyleva and Utkin 2010, modified).*

###### Sakhtysh-2a, burial 10 (individual ID I8437, female).

**Burial 10** contained a skeleton laid in a supine position, with the head oriented to the southwest. The arms were brought together at the elbows behind the back, while the hands were positioned beneath the pelvis. The legs were crossed, with the right leg overlapping the left (Kostyleva and Utkin, 2010). The burial is dated to 3635–2896 calBCE (4540±160 BP, GIN-6234).

###### Sakhtysh-2a, burial 11 (individual ID I8419, female).

**Burial 11** (individual I8419) was atypical for the site, as the skeleton was found in a crouched position. The deceased, a female aged 20-25 years, was laid on her left side, with the left arm extended beneath the body and the right arm bent at the elbow, pressed against the chest. The leg bones exhibited significant bending, with the legs drawn towards the stomach, and the upper sections of the legs overlapping the feet. Due to soil pressure, the feet had shifted, leading to ligament rupture. It is likely that the body was initially swaddled, as indicated by the compression of the skeleton, which resulted in the fracturing of multiple bones under mechanical pressure.

Initially, the burial was attributed to the Neolithic Pit-Comb Ware Lyalovo culture, based on its stratigraphic position and the unusual burial rite for the Volosovo population (Kostyleva and Utkin, 2010). However, radiocarbon dating (3520–3193 calBCE (4616±38 BP, UBA-39999)) from the human bone contradicted this interpretation. In a recent article, the individual was assigned to the Corded Ware Fatyanovo culture and dated to the transitional period between the Eneolithic and Bronze Age phases of the Sakhtysh complex (Meadows et al., 2024). However, our genetic analysis strongly links individual I8419 to the Volosovo population.

Thus, we interpret this burial as an early manifestation of cultural changes occurring during the late phases of the Eneolithic in the Volga-Oka region, with no detectable shifts in the genetic profile of the local population compared to earlier Volosovo-associated inhabitants of Sakhtysh.

###### Sakhtysh-2a, burials 9 (individual ID I8420, male), 13 (I12500, female), 32 (I8404, male), 33 (I8408, male), 34 (I12964, male), 35 (I8414, male), 39 (I8418, male), 46 (I8405, male), 54 (I12962, female), 56 (I12498, male), 58 (I8417, male), and an unidentified burial (I8409, male)

In **burial 58** (individual ID I8417), a particularly remarkable discovery was made—a pendant in the form of an anthropomorphic figurine. This unique artifact was located near the neck vertebrae. Crafted from a thin (2 mm) and meticulously polished bone plate, the figurine depicts a standing person when viewed from the front, presenting a full-face image.

Funerary offerings were not present in all of the burials examined. However, in **burial 39** (individual ID I8418), a beaver's lower jaw was discovered near the right knee, while a bear's tusk was found near the left hip. Various funerary items including jewelry such as beads and pendants, as well as clothing adornments made of materials like stone (serpentine, slate), bones, and animal teeth (bear, elk, beaver, and wolf) were found in **burials 9** (I8420)**, 13** (I12500), **34** (I12964), **46** (I8405), **54** (I12962), **56** (I12498), and **58** (I8417). Amber, a highly prized material, was also among the discovered items, and in some burials, amber ornaments were the only grave goods, as was recorded in **burial** **39** (I8418).

**Burial 32** is dated to 3948-3768 calBCE based on a human bone/tooth [R_combine: (5060±25 BP, PSUAMS-9086), (4981±37 BP, UBA-40004)]; **33** – to 3946-3658 calBCE (5011±35 BP, UBA-39995); **34** - to 3986-3798 calBCE [R_combine: (4540±150 BP, GIN-7276), (5143±34 BP, UBA-40005)]; **35** - to 3960-3657 calBCE [R_combine: (4080±180 BP, GIN-7273), (5118±59 BP, UBA-40006)], **54** - to 3768-3649 calBCE [R_combine: (4900±30 BP, PSUAMS-9401), (4964±23 BP, AAR-15051)], **58** - to 4335-4173 calBCE [R_combine: (5430±25 BP, PSUAMS-9088), (5328±39 BP, UBA-39993)], and the **unidentified burial** (I8409) is dated to 4047–3961 calBCE based on a human bone/tooth (5195±25 BP, PSUAMS-9087).

Thus, the chronological attribution of the Volosovo burials from the Sakhtysh ensemble spans from 4400 to 3600 BCE. However, burial 12 from Sakhtysh-2 and burial 11 from Sakhtysh-2a extend beyond this interval, suggesting their association with the later period. These burials likely correspond to the transitional phase between the Eneolithic and the Bronze Age in the region.


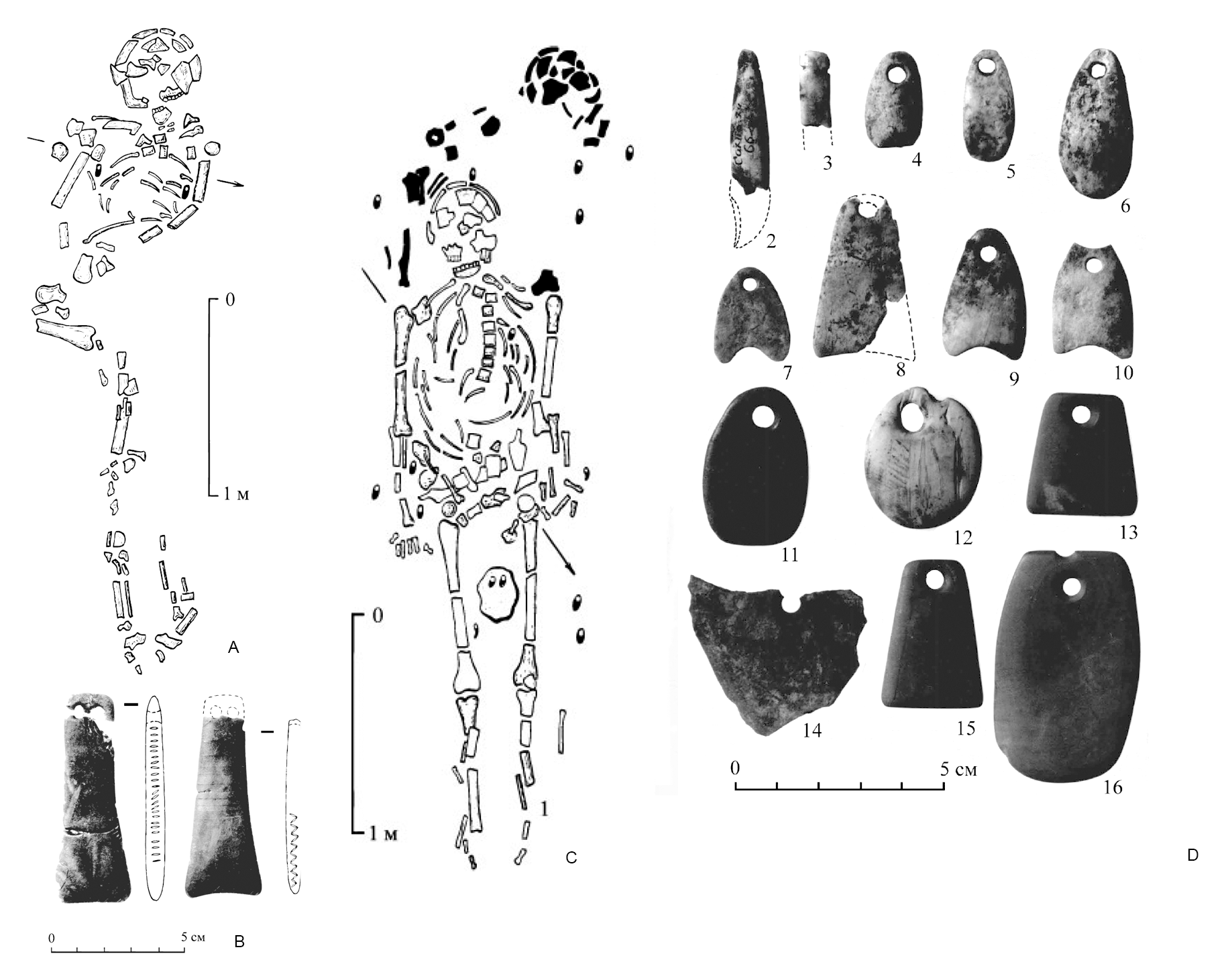


***Figure S74.*** *Burials 9 and 13a/b at the Sakhtysh-2a site.* ***A*** *- burial 9;* ***B*** *- amber pendants from burial 9;* ***C*** *- burials 13a and 13b;* ***D*** *- pendants made of teeth and amber from burials 13a and 13b (by Kostyleva and Utkin 2010, modified).*


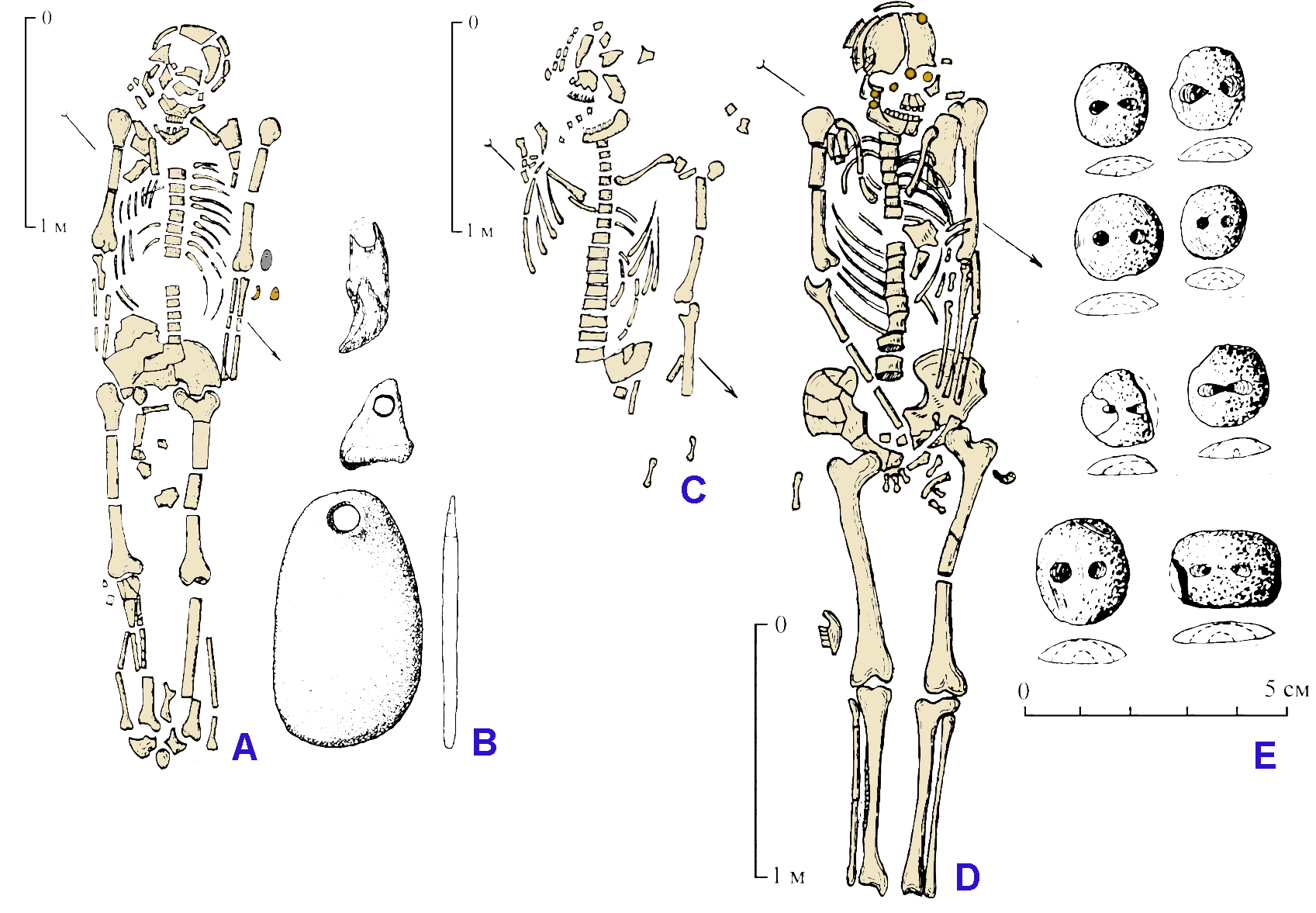


***Figure S75.*** *Burials 33, 34, 39 at the Sakhtysh-2a site.* ***A*** *- burial 34;* ***B*** *- pendants made of stone and teeth from burial 34;* ***C*** *- burial 33;* ***D*** *- burial 39;* ***E*** *- amber buttons from burial 39 (by Kostyleva and Utkin 2010, modified).*


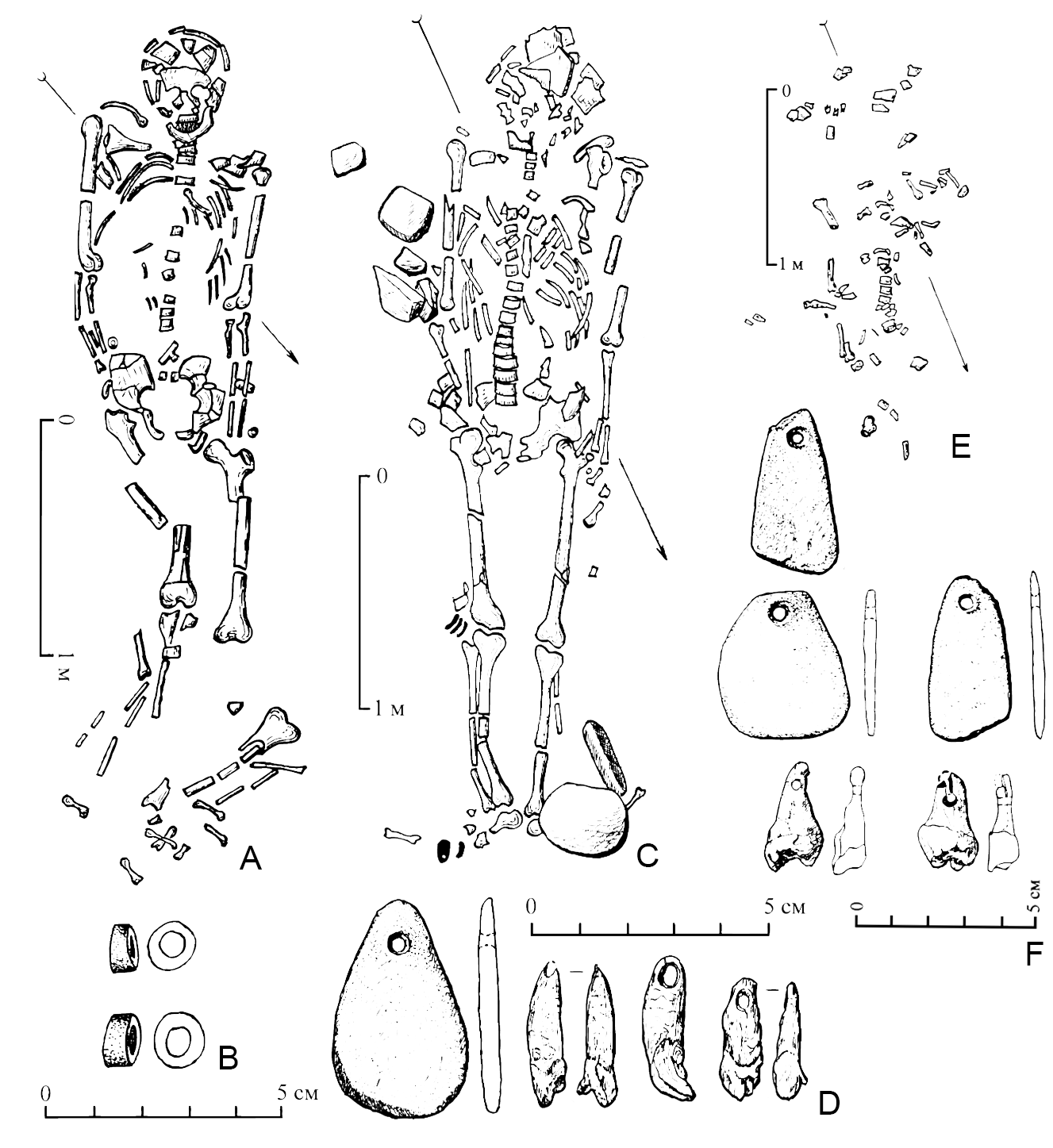


***Figure S76.*** *Burials 46, 54, 56 at the Sakhtysh-2a site.* ***A*** *- burial 46;* ***B*** *- stone beads from burial 46;* ***C*** *- burial 56;* ***D*** *- pendants made of stone and teeth from burial 56;* ***E*** *- burial 54;* ***F****- pendants made of stone and teeth from burial 54 (by Kostyleva and Utkin 2010, modified).*


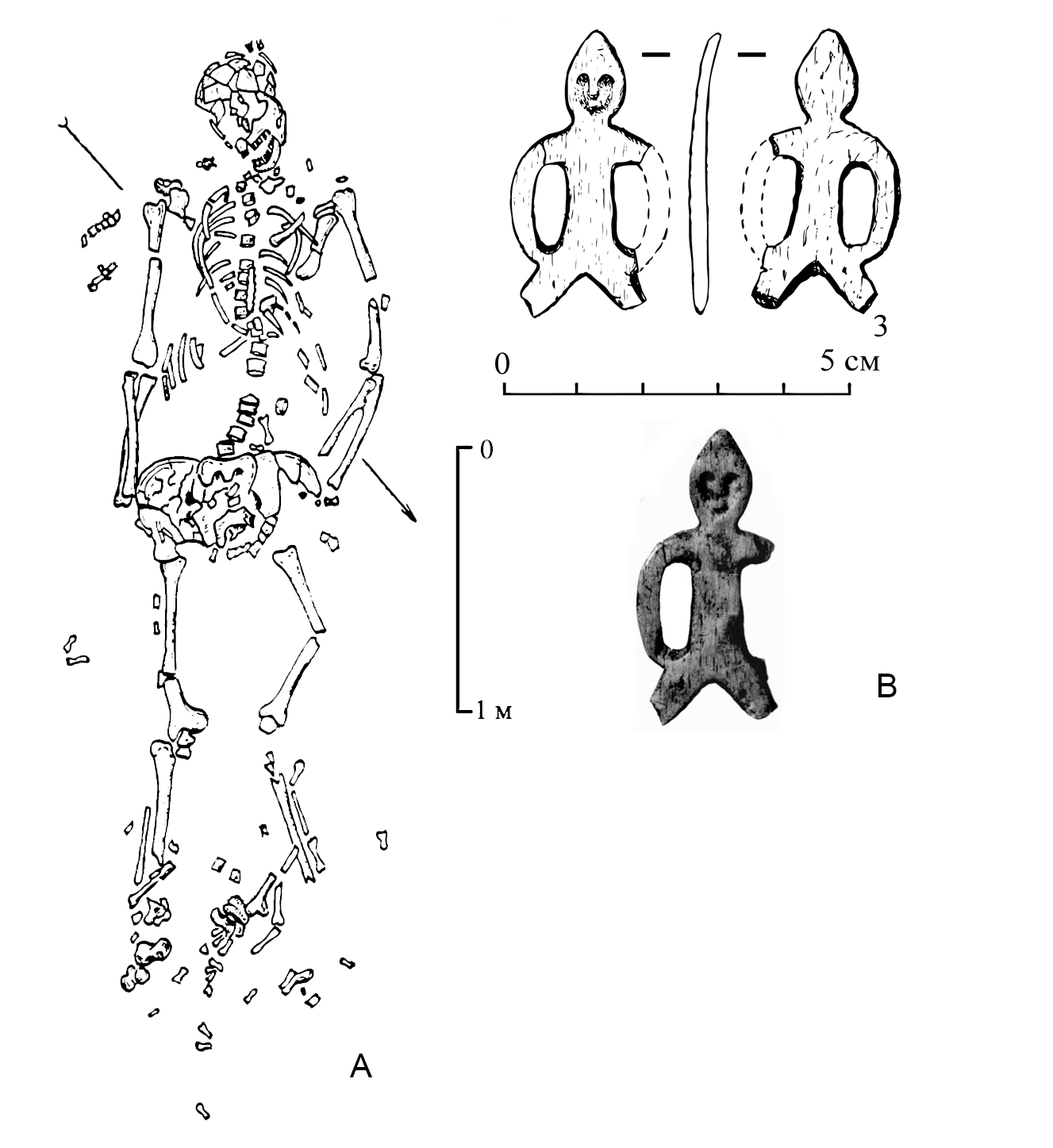


***Figure S77.*** *Burial 58 at the Sakhtysh-2a site.* ***A*** *- burial 58;* ***B*** *- anthropomorphic figurine pendant from burial 58 (by Kostyleva and Utkin 2010, modified)*.

##### Imerka-8 occupation site

The Imerka-8 archaeological site is situated on a gentle slope of a sandy dune covered with grass, located on the right bank of the Vad River. The dune reaches a height of 7 meters above the water level. The stratigraphic sequence observed at the site enabled the identification of both early and late Volosovo cultural complexes, which were subsequently overlaid by a layer containing Imerka cultural materials.

The Imerka culture layer superimposes earlier deposits and has yielded a significant number of ceramic fragments, some of which exhibit traces of metalworking activities. These ceramic artifacts are characterized by their distinct originality and lack of analogs in the materials of local cultures. The Imerka culture was first recognized in the mid-1980s through a study of the Imerka-5 settlement by A.A. Vybornov and V.P. Tretyakov. Initially, these materials were attributed to the Volosovo culture, but further research has revealed their later chronological placement, indicating that the Imerka culture succeeded and replaced the Volosovo culture in the Sura and Moksha river basins. It is now understood that the Imerka culture came to an end with the arrival and expansion of populations associated with the Fatyanovo and Balanovo Corded Ware cultures in the Sura and Moksha regions around 2300 BCE.

The Volosovo complex at the site comprises three fully excavated dwellings and an additional five that have been partially explored. Numerous fragments of ceramic vessels and tools have been recovered from these contexts, providing valuable insights into the material culture of the Volosovo people. In addition to the Volosovo cultural remains, pottery associated with the Serednii Stih culture has also been discovered at the Imerka-8 settlement, indicating possible cultural interactions or influences in the region (Korolev and Stavitskiy, 2021a).

###### Unnumbered burial (individual ID I8292, female)

The skeletal remains from this burial (individual ID I8292) are partially preserved and were discovered within the humus layer. The exact boundaries of the grave pit could not be determined. The cultural affiliation of the burial remains unclear, as it could potentially be attributed to either the Volosovo or Imerka population. The chronological placement of the skeleton closely aligns with the dating of animal bones found in the fill of Volosovo structures (Korolev, personal communication).

## Seima-Turbino-period individuals

### Overview of the Seima-Turbino phenomenon

The Seima-Turbino assemblage, also known as the Seima-Turbino transcultural phenomenon, refers to a complex cultural development that spread across Northern Eurasia during the transition from the Middle to Late Bronze Age, spanning the last centuries of the third millennium BCE to the first centuries of the second millennium BCE. Artifacts associated with the Seima-Turbino assemblage have been discovered in various locations, ranging from the Sayano-Altai region and Xinjiang to Fennoscandia and the Dniester basin.

The Seima-Turbino assemblage is characterized by a distinctive set of bronze weapons, including spearheads, celts, knives, and daggers with zoomorphic pommels (pommels with animal figurines). These artifacts were crafted using advanced thin-walled casting techniques. The majority of Seima-Turbino artifacts have been found in burial contexts, with notable sites including Seima, Turbino, and Rostovka. While human burials are infrequently encountered, the focus is primarily on memorial clusters (i.e., cenotaphs, or collections of funerary goods with no associated body). These clusters consist of shallowly buried groups of bronze tools, weapons, and stone arrowheads.

It is believed that the Seima-Turbino cultural complex originated in the region near the Dzhungarian Gate. From there, several militarized groups carrying Seima-Turbino traditions migrated westward, as evidenced by the appearance of Seima-Turbino technologies and artifact forms in cultures such as Elunino, Krotovo, and Odinovo in West Siberia in the Ob-Irtysh basin. From archaeological evidence, it is also believed that the westward spread of Seima-Turbino artefacts also involved forest hunters and fishermen from the Upper Yenisei and Baikal regions, as indicated by the presence of flint and bone arrowheads and knives with bone bases. The influence of Seima-Turbino traditions on the populations of the Sintashta and Abashevo cultures is documented in the Kama and Ural regions. In the forested areas of North Asia, the Seima-Turbino traditions can be observed within the Samus’-Kizhirovo cultural complex (Chernykh and Kuz’minykh, 2015), a trans-cultural analogue of the Seima-Turbino phenomenon with similar but slightly differing bronze artifacts.

#### Seima-Turbino necropolises (burial sites)

##### Rostovka burial site

The burial ground is located 15 km east of Omsk, on the second left bank terrace of the Omi River, a right tributary of the Irtysh River. The excavation of this site was conducted by V. I. Matyushenko in 1966–1969. Over the course of four seasons, an area of 1376 square meters was excavated, revealing 38 burials and a number of artifact assemblages outside the burials. Two phases have been identified at the Rostovka burial ground: an early phase associated with the Seima-Turbino phenomenon and a late phase belonging to the Early Iron Age.

The burials of the Seima-Turbino phase were constructed as rectangular pits, ranging in depth from 0.1 to 0.7 m from the surface, mostly measuring 0.3 to 0.5 m deep. They were usually oriented from east to west, with slight variations towards the south or north. The burial ritual included: 1) inhumation, sometimes accompanied by partial cremation; 2) off-site cremation; 3) decapitated burials; 4) burials of skulls only. One interesting detail of the burial ritual is the offering of objects to the deceased, left at the edge of the burial pits or placed slightly apart from them. Often these objects, such as casting molds, are intentionally broken or damaged. In some cases, a ritual is observed where spears and celts are thrust into the bottom, walls, or edges of the burial pit.

Many burials at Rostovka were intentionally and systematically destroyed in the past. In all the disturbed graves, only the bones of the lower limbs, either tibiae and talus bones or only feet, are preserved in anatomical order.

One of the specific features of Rostovka is the presence of burials of metalworkers, which are rare in the Eurasian necropolises of the Bronze Age. At least two such burials have been recorded here (burial 21 and the burial in square 20Ж). Fragments of casting molds are also found on the surface of the burial ground, although they may originate from disturbed burials.


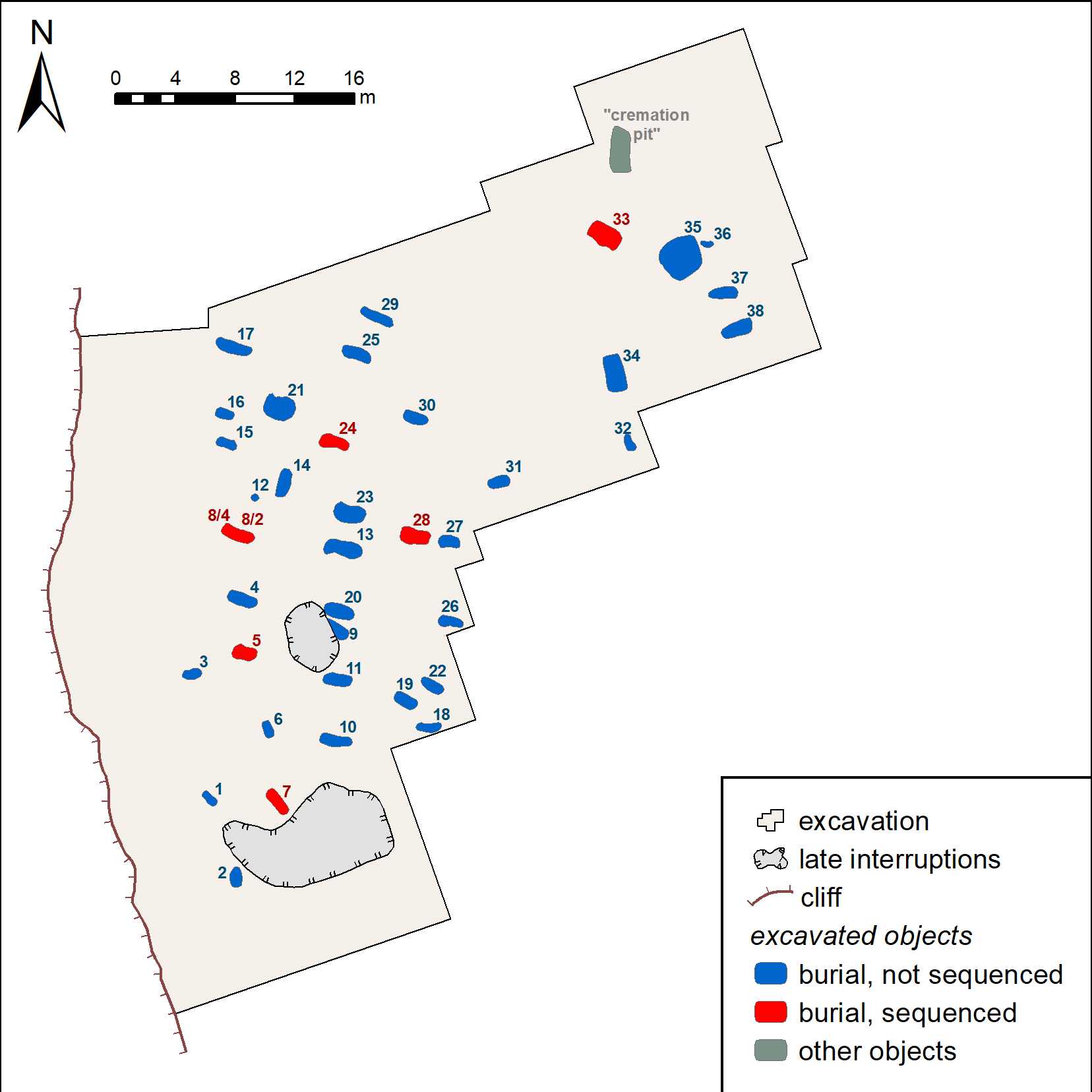
***Figure S78****. The burial ground at Rostovka (Omsk, Russia), Seima-Turbino burials.*

The burial ground has yielded a large number of artifacts, including stone arrowheads, knives, plates, bronze celts, spearheads, knives and daggers, awls and chisels, needles; bronze, gold, jade, and lapis lazuli ornaments; bone handles of knives and awls, scales; talc and clay casting molds, as well as fragments of pottery (Chernykh and Kuzminykh, 1989).


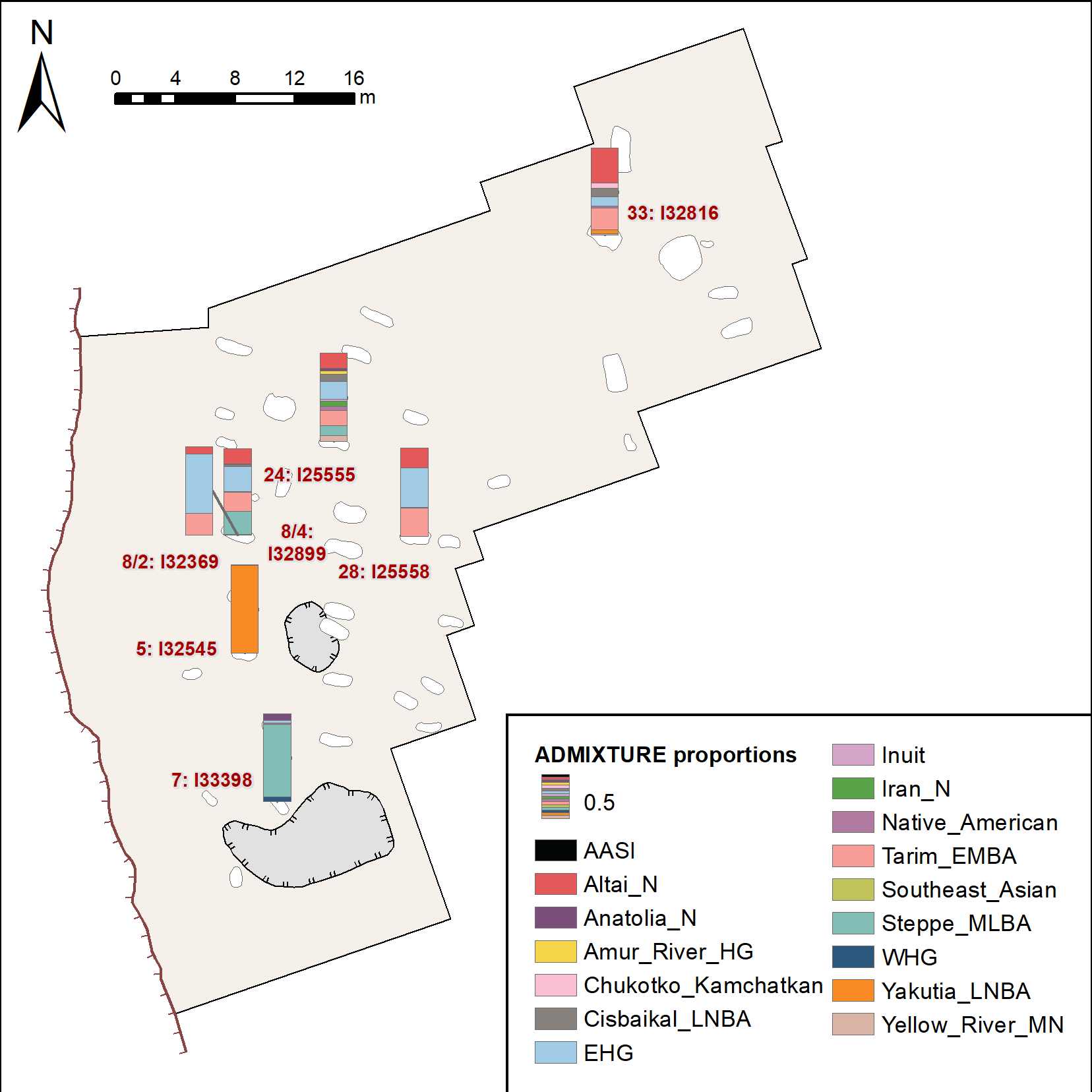


***Figure S79.*** *The burial ground at Rostovka. The ADMIXTURE results for the sequenced Seima-Turbino burials.*

The burials of the Seima-Turbino phase date back to the 22nd-18th centuries BCE. Based on the characteristics of the artifacts and a series of radiocarbon dates, two periods of burials have been identified (Marchenko et al., 2017).

**Table S2**.

| **Grave number** | **Genetic ID** | **Dated material** | **C14 date, lab code** | **R-date** | **cal date, BCE** | **Chronological markers in the grave** | **Period** | **Reference** |
| --- | --- | --- | --- | --- | --- | --- | --- | --- |
| **5** | I32545 | human bone | UBA-31379 | 3508±40 | 1938–1700 | spearhead with rhombic cross-section | 2 | Marchenko et al., 2017 |
|  |  | human tooth | UBA-31396 | 3682±40 | 2197–1951 |  |  |  |
|  |  | sequenced sample | PSUAMS-7548 | 3585±20 | 2021-1884 |  |  |  |
| **7** | I33398 | sequenced sample | PSUAMS-9093 | 3520±20 | 1926-1754 |  |  |  |
| **8** | I32899 | ind. 4, human bone | UBA-31381 | 3709±34 | 2202–1983 |  | 1 | Marchenko et al., 2017 |
|  |  | ind. 4, human tooth | UBA-31398 | 3708±30 | 2200–2023 |  |  |  |
|  |  |  |  |  |  |  |  |  |
|  | I32369 | ind. 2 |  |  |  |  |  |  |
| **14** |  |  |  |  |  | dagger with a longitudinal rib | 2 |  |
| **20** |  | human bone | IMCES (ИМКЭС)-14С982 | 3634±68 | 2201-1776 |  |  | Kovtun et al., 2017 |
| **23** |  | human bone | UBA-29311 | 3822±32 | 2452–2144 |  |  | Marchenko et al., 2017 |
| **24** | I25555 | human tooth | UBA-29313 | 3564±38 | 2054–1774 | cast mold for spearheads with rhombic cross-section; dagger with a longitudinal rib, spearhead with rhombic cross-section (found in associated assemblage) | 2 |  |
| **27** |  | human bone | UBA-31383 | 3655±40 | 2141–1918 |  |  | Marchenko et al., 2017 |
|  |  | human tooth | UBA-31399 | 3635±40 | 2135–1896 |  |  |  |
| **28** | I25558 | sequenced sample | PSUAMS-12587 | 3620±20 | 2035-1900 |  |  |  |
| **29** |  | human bone | IMCES (ИМКЭС)-14С916 | 3888±105 | 2832-2033 |  |  | Kovtun et al., 2017 |
| **33** | I32816 | human bone | UBA-31382 | 3640±31 | 2133–1919 |  | 1 | Marchenko et al., 2017 |

***Table S2****. Relative and absolute chronology of the sequenced Rostovka burials.*

###### Burial 5 (individual ID I32545, male)

**Burial 5** was situated at a depth of 30 cm from the modern surface. It appeared as a rectangular patch of gray earth with charcoal and burnt clay inclusions, and with dimensions measuring 65x150 cm. The orientation of the burial was from ENE to WSW. The skeleton of a male (sex is confirmed by genetic analysis), approximately 18-20 years old, was found lying on his back with the head to the east-northeast. The preserved bones include the ulna and humerus of the arms, the femur and lower leg of the left leg, as well as the pelvic bones, although they were displaced. The skull, chest bones, spine, and phalanges were in a very poor state of preservation. Within the grave, several artifacts were discovered, including a bronze socketed spearhead, four jasper flakes, and stone arrowheads. The spearhead was likely not placed within the grave but left after most of the filling took place, and it was found stuck into the wall of the grave. Near the grave, a cluster of artifacts was found, including several flakes and an arrowhead.


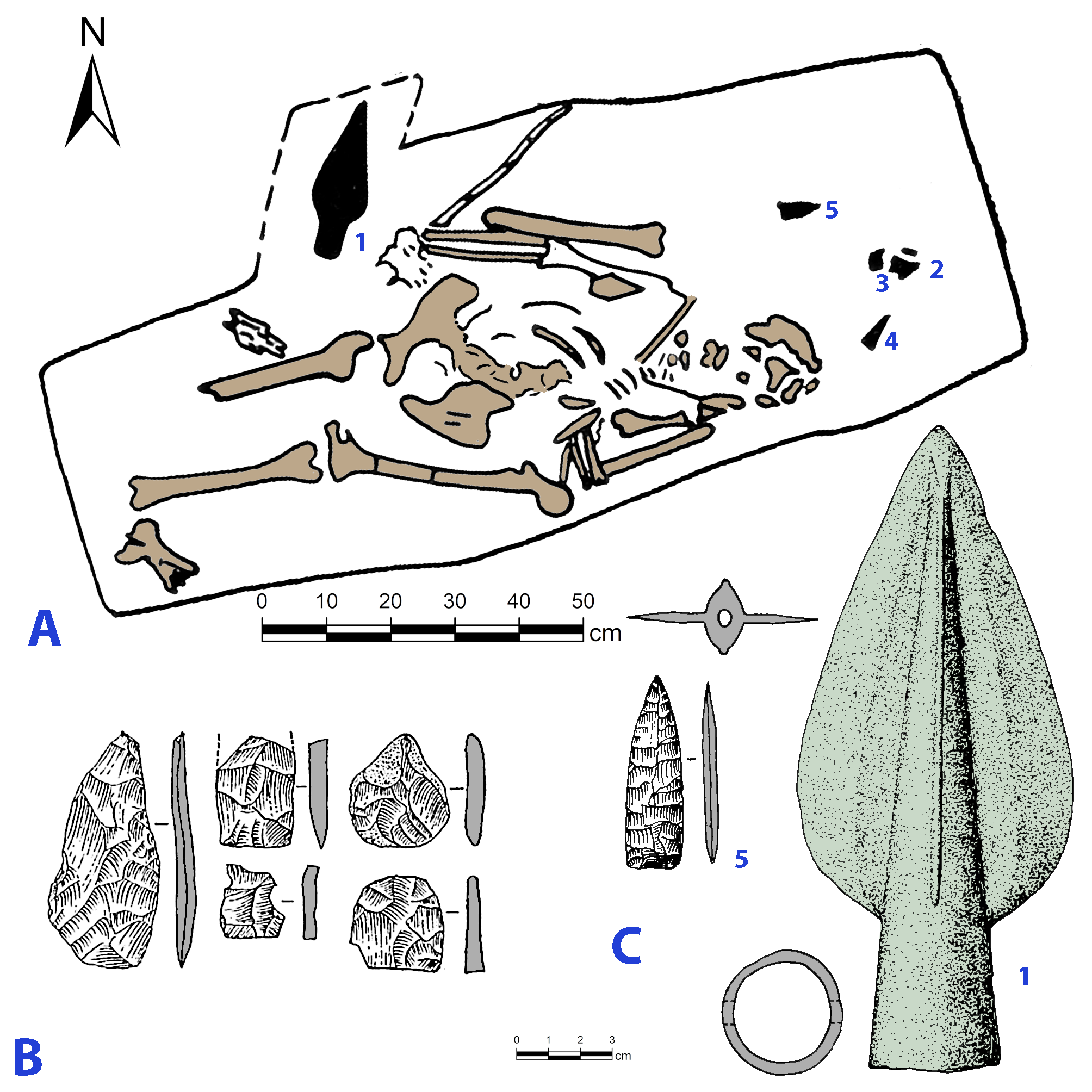


***Figure S80.*** *Rostovka, burial 5.* ***A*** *- plan of the burial,* ***B*** *- grave goods:* ***1*** *- spearhead,* ***2, 3*** *- jade flakes,* ***4****,* ***5*** *- arrowheads;* ***C*** *- cluster of finds near burial C* *(by Matyuschenko & Sinitsyna 1988, modified).*

###### Burial 7 (individual ID I33398, male)

**Burial 7** was located at a depth of 40 cm from the modern surface. It was observed as a rectangular patch of gray earth filled with charcoal and burnt subsoil. The dimensions of the burial were measured to be 70x220 cm, with the WNW to SSE orientation. During the excavation, a strip of charcoal was uncovered at the level of the initial filling, running from the northwest to the southeast. Additionally, pieces of charcoal were found in separate clusters, approximately 20 cm above the bottom of the grave, suggesting the presence of remnants of a burnt wooden ceiling or lining. Among the findings within the burial, on the first layer, a roughly worked stone artifact, a fragment of a deer antler possibly used as a handle, and an ellipsoid-shaped bone object with holes at the ends were discovered. The skeletal remains of an adult male (sex is confirmed by genetic analysis), estimated to be 35-50 years old, were found in a bent position on the left side with the feet pointing to the west. However, the preservation of the skeleton is poor, with only the left and right femur and tibia intact. Disorganized ribs and a humerus were located beneath the left thigh, while the lower jaw was found under the right ulna. The cluster of finds near burial 7 included an asymmetric quartzite knife, several fragments of bone armor, a fragment of a stone object, and a small stone.


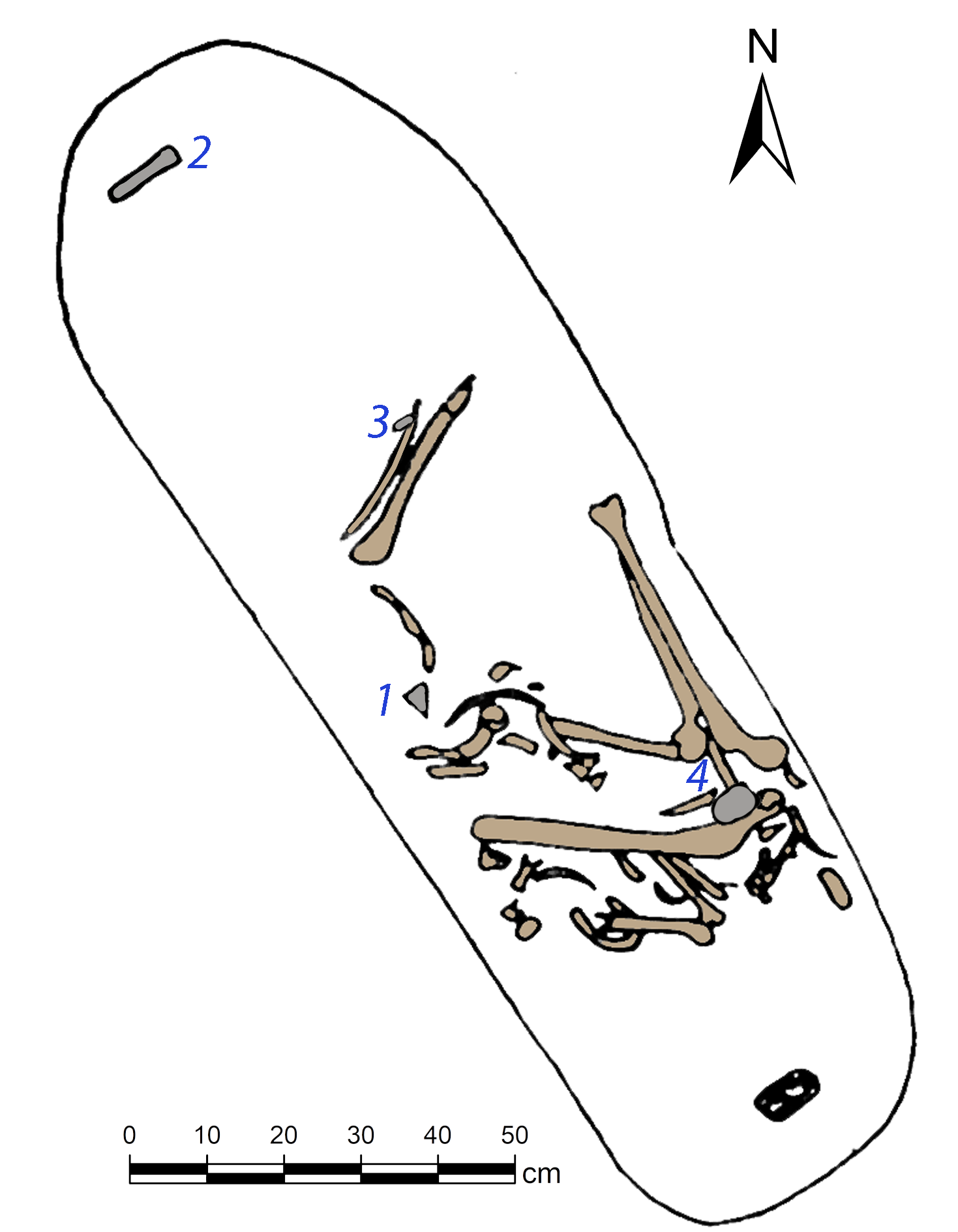


***Figure S81.*** *Rostovka, burial 7 (by Matuschenko & Sinitsyna 1988, modified).*

###### Burial 8, individuals 2 (individual ID I32369, male) and 4 (I32899, female)

**Burial 8** was situated at a depth of 40 cm. It was identified as a rectangular patch of gray mixed earth, slightly infused with charcoal. The orientation of the burial was from ENE to WSW. The burial contained the skeletal remains of four individuals.

***Individual 1*** (I) occupied the upper level in the grave. It is represented by preserved burnt human bones and skull fragments which likely belonged to an infant, indicating a cremation event in this section. The bones were undrlayed by two strips of charcoal pieces, arranged along the long walls of the grave pit. Based on these observations, the excavators of the burial supposed that the body of ***individual 1*** was cremated inside the grave.

During the excavation, five stone arrowheads were unearthed at different locations within the grave, specifically at the level of charcoal and human bones.

***Individual 2*** (II) (individual ID I32369), an adult male (sex confirmed by genetic analysis), was unearthed on the bottom of the grave, in the supine position with the head facing east. The skeleton is well-preserved, most of the bones were intact, although the skull was missing. In the southwestern part of the grave near the left knee of ***individual 2*** six stone arrowheads and two knife-shaped pieces were found, oriented with their tips to the west, suggesting that they were originally put in a quiver. On the pelvic bone, a bronze knife and a bronze awl with a bone handle were found. The knife was pointing southward, while the awl was oriented towards the north.

Two more skeletons were placed on top of ***individual 2***.

***Individual 3*** (III), an adult male, was found In the southwestern part of the grave. The skull of this individual was placed above the left half of the pelvis of ***individual 2***, with the back of the head facing upwards. Adjacent to the skull, a bone handle of a knife or dagger was excavated. The pelvic bones (two of them) of this skeleton were discovered in the eastern half of the grave.

***Individual 4*** (IV) (individual ID I32899) was excavated in the western part of the grave. The skull faced upwards and the left tibia, the right half of the pelvis, the right ulna, and the left clavicle of a female aged 40-50 years, lay above the leg bones of ***individual 2***. No grave goods are associated with this individual.

Following the removal of all the skeletons, a patch of mixed earth was discovered at the bottom of the grave in its eastern half. This patch was deepened by 15 cm from the bottom of the grave and yielded three bronze items, two spearheads, and a socketed axe (celt). All items were positioned with their blades facing west.

Near the burial, a cluster of artifacts was excavated, including 8 stone flakes, a bronze spearhead stabbed into the ground, a fragment of a spearhead crafted from shale, a knife-shaped piece, a blank for a knife made of diorite, and a pottery vessel.


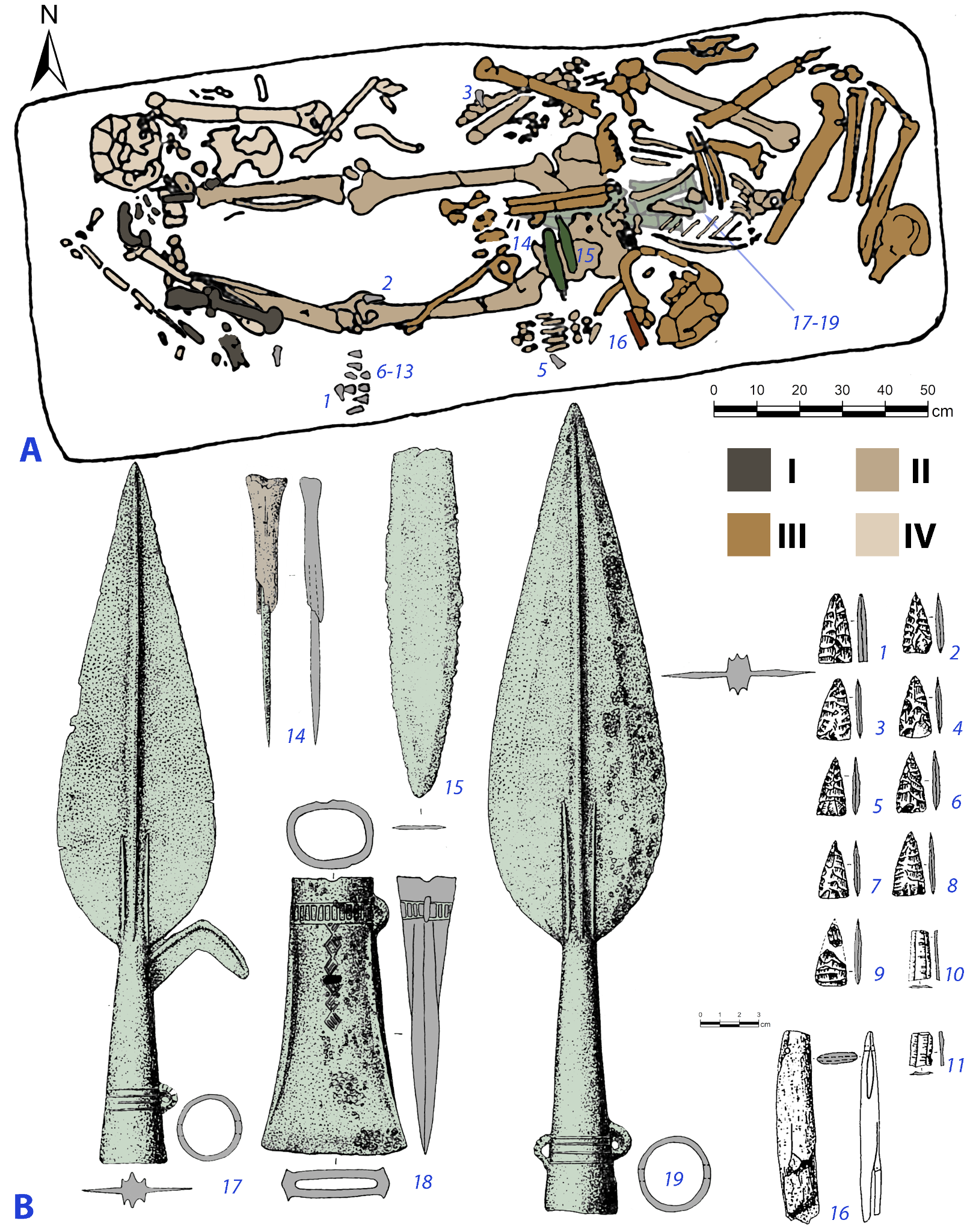


**Figure *S82.*** *Rostovka, burial 8.* ***A*** *- plan of the burial:* ***I-IV*** *- skeletons in the burial;* ***B*** *- grave goods:* ***1-9*** *- arrowheads,* ***10-11*** *- blades,* ***14*** *- awl, 15 - knife,* ***16*** *- bone handle,* ***17*** *and* ***19*** *- spearheads,* ***18*** *- socket axe (celt) (by Matuschenko & Sinitsyna 1988, modified).*

###### Burial 24 (individual ID I25555, male)

**Burial 24** was unearthed at a depth of 15 cm from the modern surface and appeared as a dark patch of mixed earth, measuring 70x200 cm. Pieces of calcined soil were found in the infill of the grave. The skeleton was anthropologically attributed to a female aged 20 years old, but genetic analysis shows male sex, with poor preservation of the skeletal remains explaining the incorrect anthropological attribution: the grave contained only parts of thighs, pelvis, tibia, right hand, and mandibula. The grave goods included a stone arrowhead with a straight base, a fragment of a clay mold used for casting a spearhead, a bronze chisel, and a bronze plate knife.

Near the grave, a cluster of finds was excavated, including two spearheads, a socketed axe (celt), an arrowhead made of clay shale, and a fragment of rock.


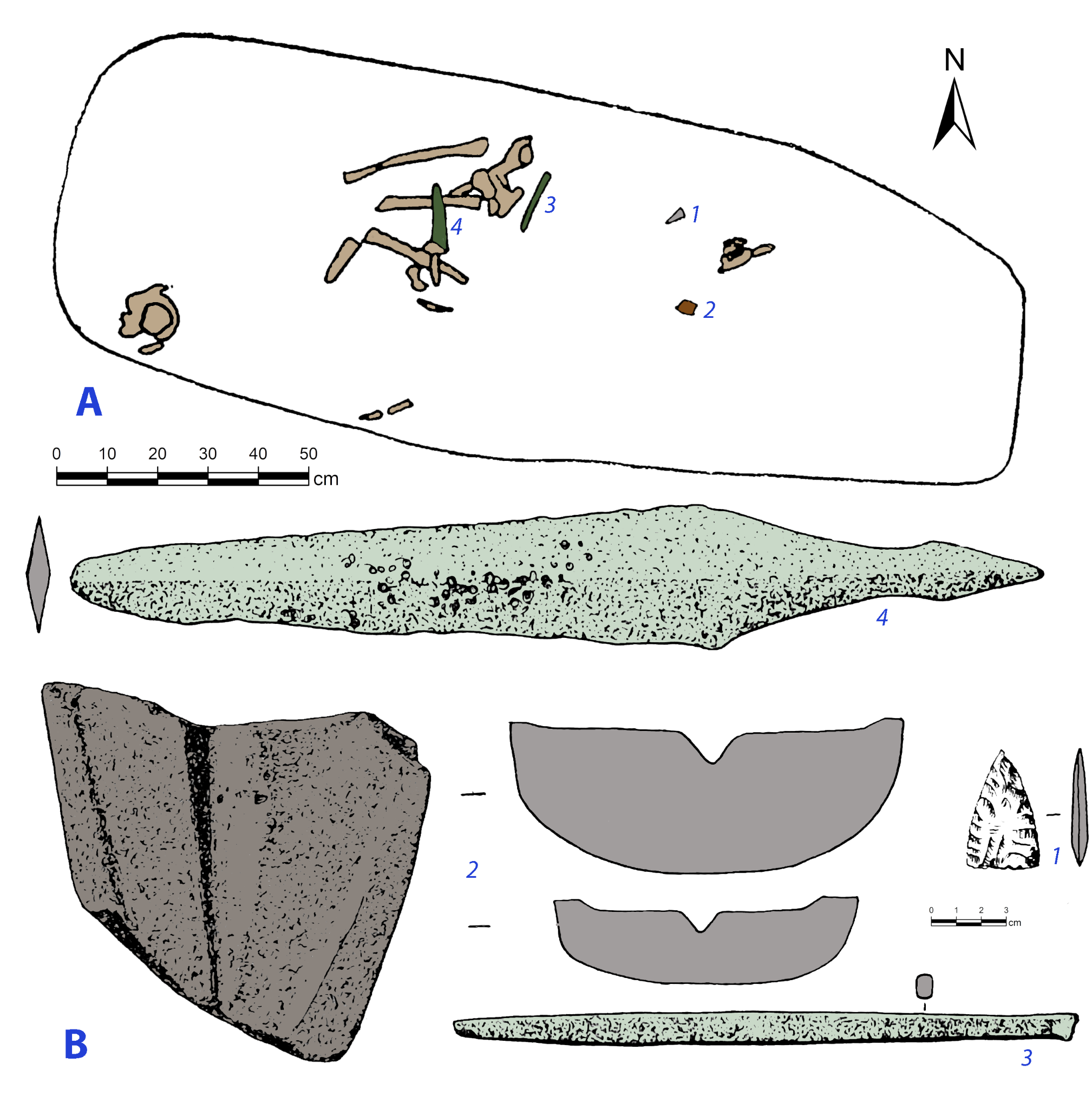


***Figure S83.*** *Rostovka, burial 24.* ***A*** *- plan of the burial;* ***B*** *- grave goods:* ***1*** *- arrowhead,* ***2*** *- a fragment of a casting mold,* ***3*** *- chisel,* ***4*** *- knife (by Matyuschenko & Sinitsyna 1988, modified).*


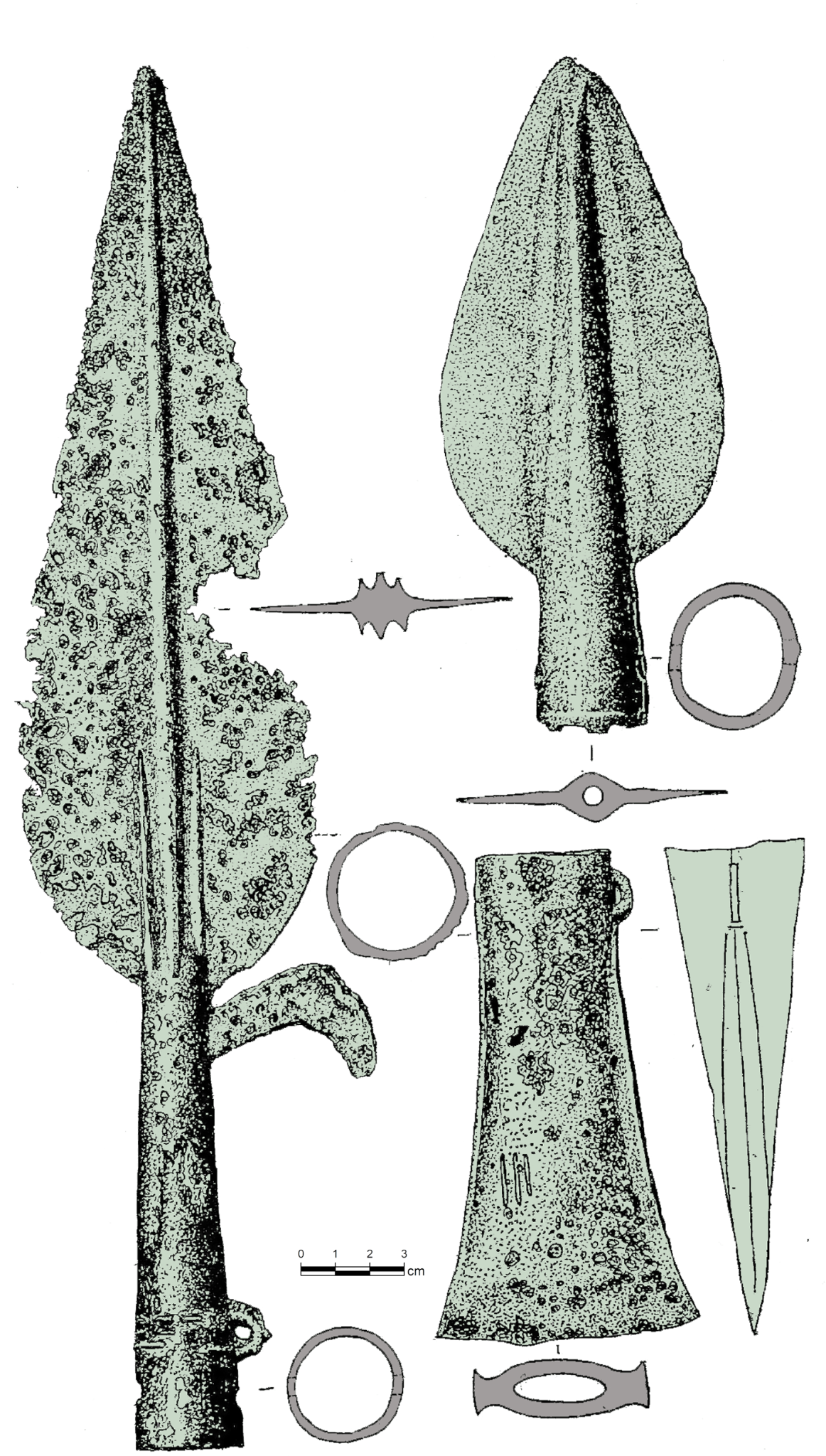


***Figure S84.*** *Cluster of finds near burial 24. Two spearheads and a socketed axe (celt) (by Matyuschenko & Sinitsyna 1988, modified).*

###### Burial 28 (individual ID I25558, female)

**Burial 28** was discovered at a depth of 60 cm from the modern surface as a patch of dark mixed soil, measuring 80x180 cm. Its orientation was from east to west. The grave contained poorly preserved incomplete human remains, anthropologically identified as male aged 30-35, but genetically identified as a female. The skeleton included the leg bones, which were found in the western half of the grave, fragments of a skull, ribs, and the left ulna. The position of the bones indicates that the deceased individual was laid with her head facing east. No grave goods were found in the grave, and a cluster of finds discovered near **graves 27** and **28** included three knife-shaped flint blades, a bronze awl, a flint blade exhibiting traces of secondary processing, six flint flakes, an accumulation of pottery sherds, a fragment of a knife-shaped flint blade, two additional flint flakes, and two fragments of a single object displaying a transverse groove on one side and a longitudinal groove on the other.


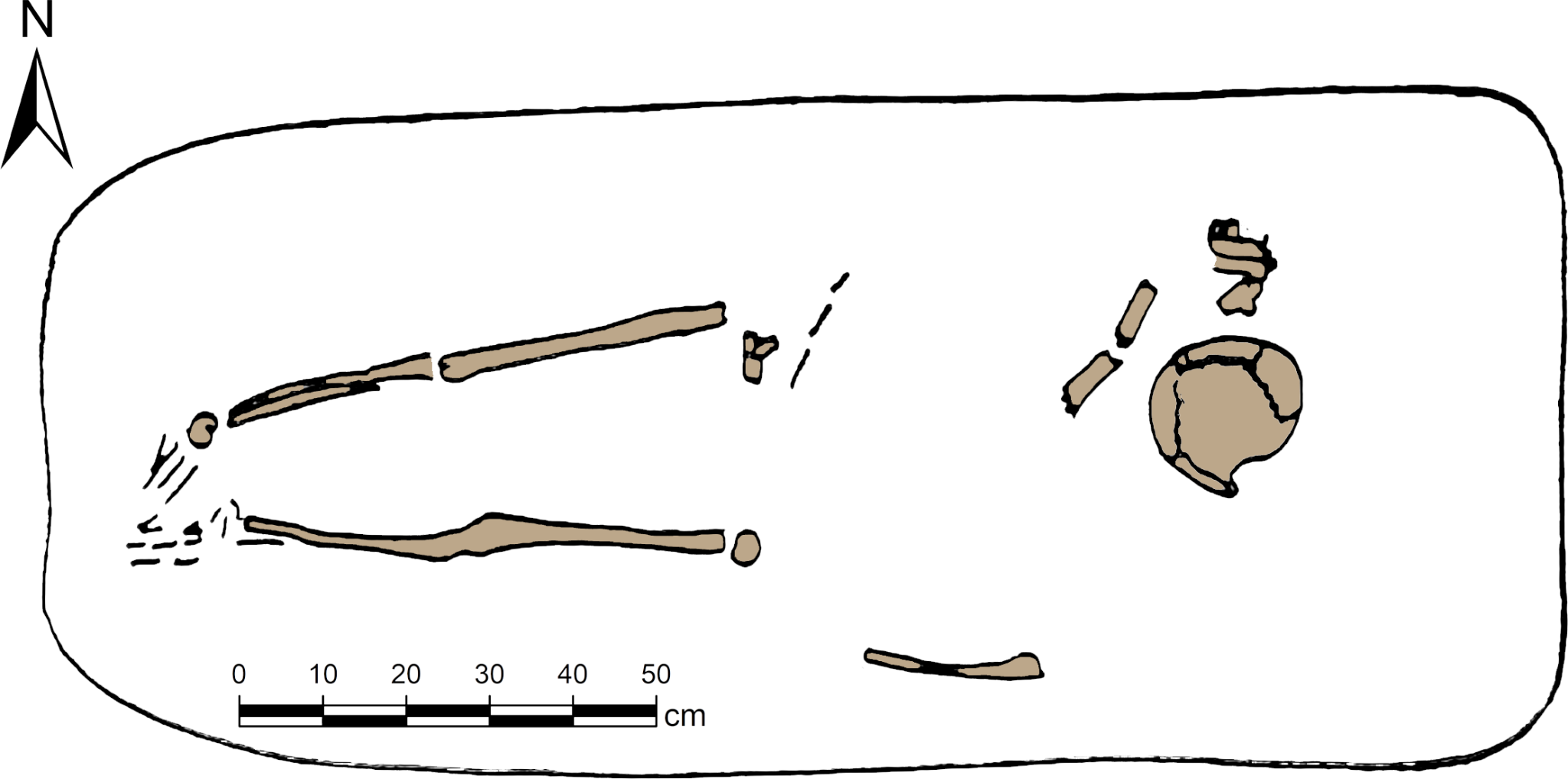


***Figure S85.*** *Rostovka, burial 28* *(by Matyuschenko & Sinitsyna 1988, modified).*

###### Burial 33 (individual ID I32816, male)

**Burial 33** was discovered at a depth of 25 cm from the modern surface. It was identified by a patch of dark calcined earth of irregular shape in plan. The dimensions of the grave were measured as 25x200 cm. At a distance of 30 cm from the southeastern wall, a spearhead was discovered, with its tip inserted into the ground. In the southwest part of the grave, a significant accumulation of bone armor plates was found in a disordered pile. To the northeast of them, a partially destroyed male skull estimated to be 20-25 years old was unearthed. The lower layer of armor plates rested at the bottom of the grave, extending in some cases towards the bottom or the wall. In the northeastern part of the grave, tibia bones were found. The skeleton likely lay in the supine position, with his head facing southeast. Among the armor plates, two golden rings and a fragment of an unfinished bone perforator were discovered, and two abrasives were located in the northern corner of the grave. In the center of the grave, a stone arrowhead with a straight base was found. The infill of the grave pit yielded additional artifacts, including the second half of a bone puncture, another stone arrowhead with a straight base, and a bone knife handle. The handle was adorned with a geometric ornament.

The cluster of finds near **grave 33** included flakes, pottery sherds, and fragments of animal bones.


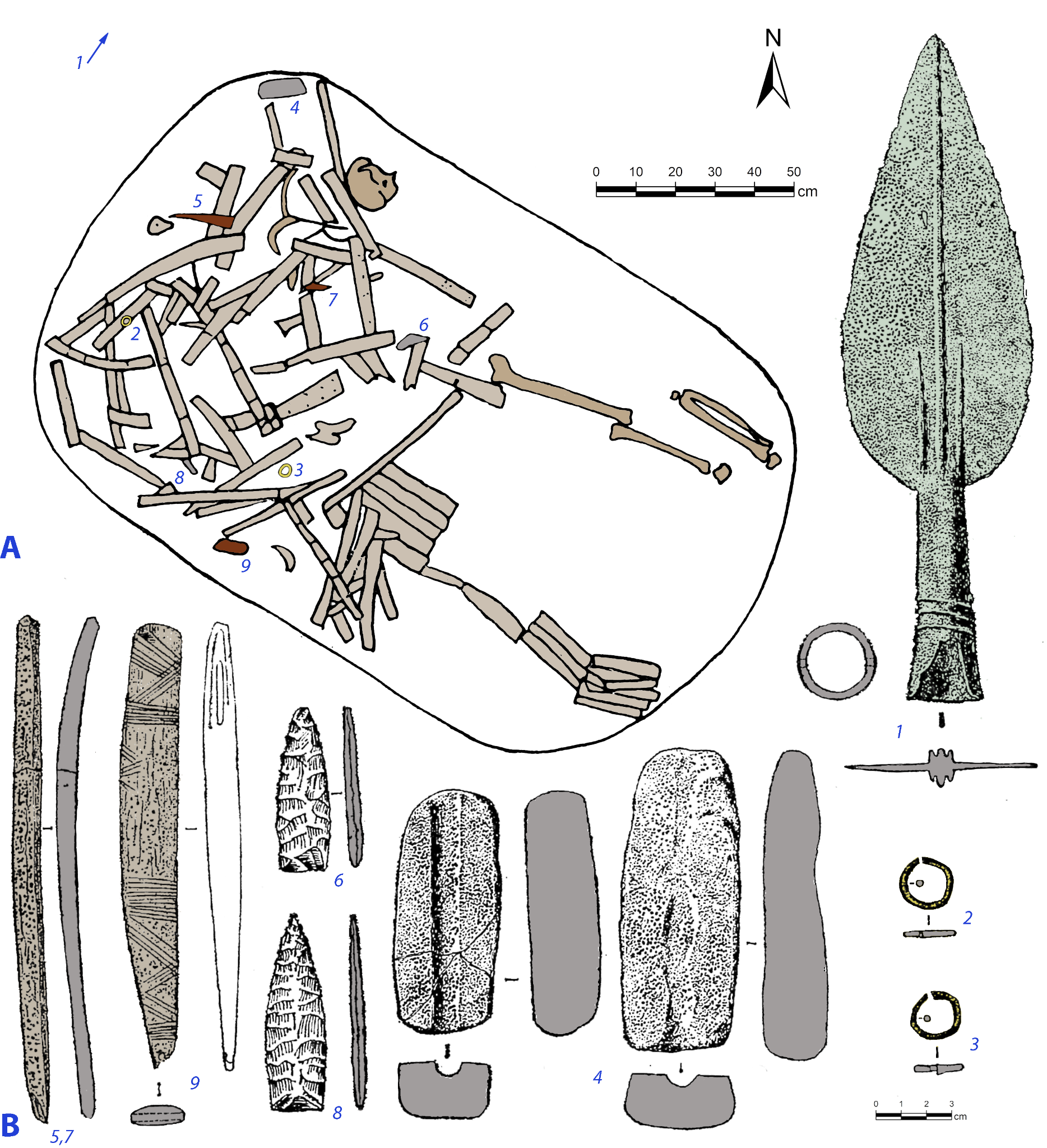


***Figure S86.*** *Rostovka, burial 33.* ***A*** *- plan of the burial;* ***B*** *- grave goods:* ***1*** *- spearhead,* ***2, 3*** *- golden rings,* ***4*** *- grinding stones,* ***5, 7*** *- bone perforator,* ***6, 8*** *- arrowheads,* ***9*** *- dagger handle (by Matyuschenko & Sinitsyna 1988, modified).*

##### Satyga-16 burial ground

The burial ground is located on the northern shore of the Satyginsky Tuman Lake, a little to the east of the place where the Gorodishe River flows into it. The Evra River flows into the lake from the northwestern side, and the Sumpanya River from the south. Satyga-16 is a multi-phase site, confined to a small oval-shaped hill, elongated in the latitudinal direction by 40-45 m with a width of up to 20 m, located at the edge of the lake terrace. The height of the hill reaches 2.5-3.0 m from the water's edge (as of July-August 2001), and the slopes are gentle.

The site was excavated in 1986 by O. N. Korochkova, V. I. Stefanov, A. A. Pogodin, and E. M. Bezprozvanny. In total, during three field seasons, an area of 529 sq. m and 41 burials of the Bronze Age were investigated. The Satyga-16 burial ground has been explored for the most part. A feature of burial grounds of the Seima-Turbino type, which is present at Satyga-16, is an abundance of finds in inter-grave space (Trufanov, 2011).


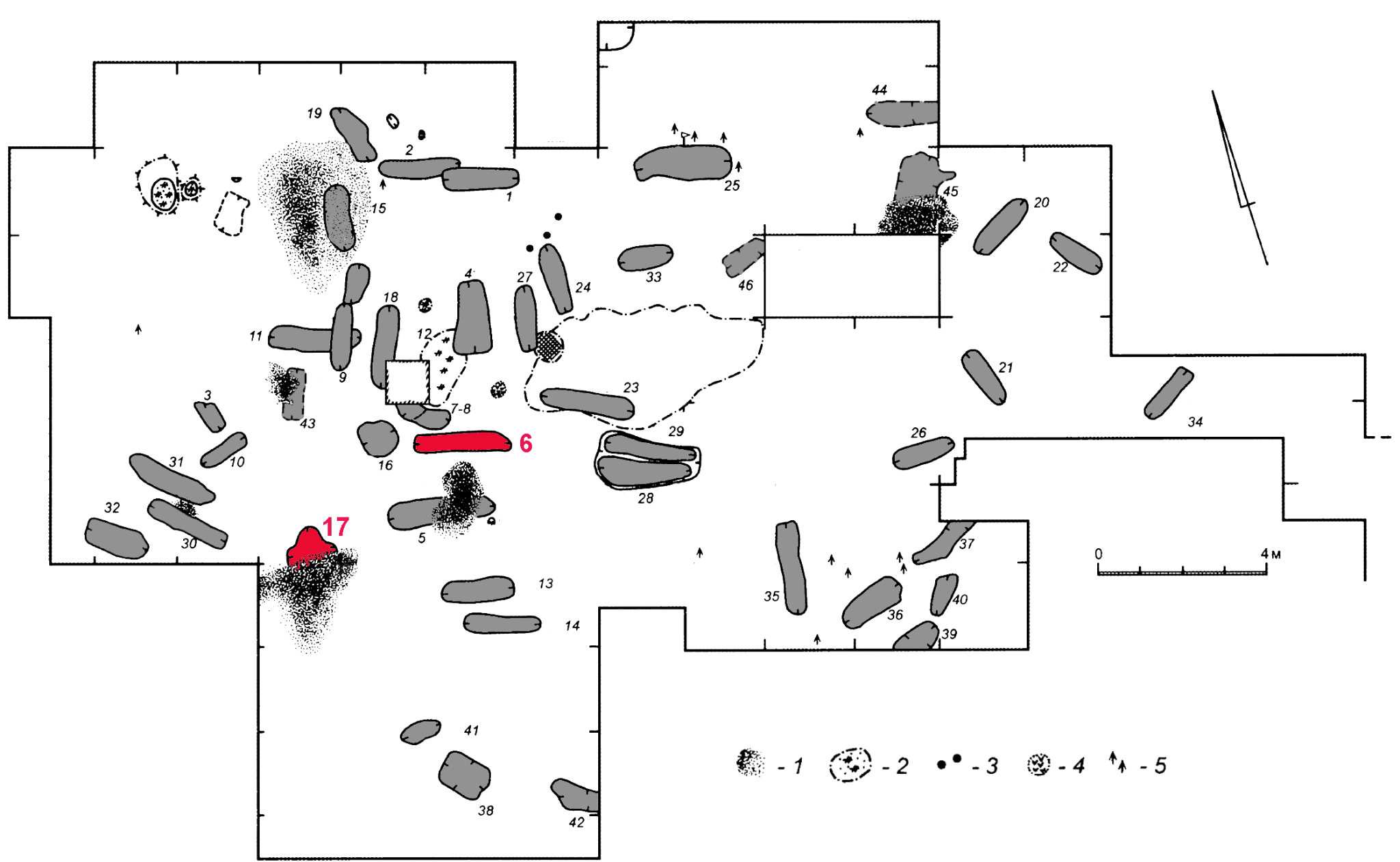


***Figure S87.*** *Cemetery Satyga-16 at the Satyga-16a occupation site.* ***1*** *- uprootings;* ***2*** *- calcinated bones;* ***3*** *- postholes;* ***4*** *- ocher;* ***5*** *- arrowhwads. The sequenced burials are labeled in red* *(image provided by Evgeniy Besprozvanny).*

###### Burial 6 (individual ID I32552, male)

The grave pit is subrectangular in plan with rounded corners, 220x46 cm in size, oriented along the west-east line. The eastern wall is cut by a small rounded pit, 35 cm in diameter. The walls of the grave pit are even, gently descending to the bottom, noted at a depth of 10-18 cm in the mainland. The eastern half of the pit is 5-10 cm deeper than the western one. The filling is brown sandy loam, covering a thin (3-6 cm) layer of pale yellow sandy loam in the bottom part, saturated with fine coals and remains of wood decay. Two carbonaceous strips (134x12x2-10 cm) located parallel to each other were unearthed 10-20 cm above the bottom along the long axis of the grave. Twelve fragments of pottery were found above the burial. At the bottom of the pit near the western wall, a skull and teeth of an adult male 30-40 years old were found. Next to the skull lay a bronze dagger in a birch bark sheath. Small fragments of ceramics were found in different places of the grave (Trufanov, 2011).

**Burial 6** is dated to 2571–2348 calBCE based on a human bone/tooth (3960±25 BP, PSUAMS-12559).


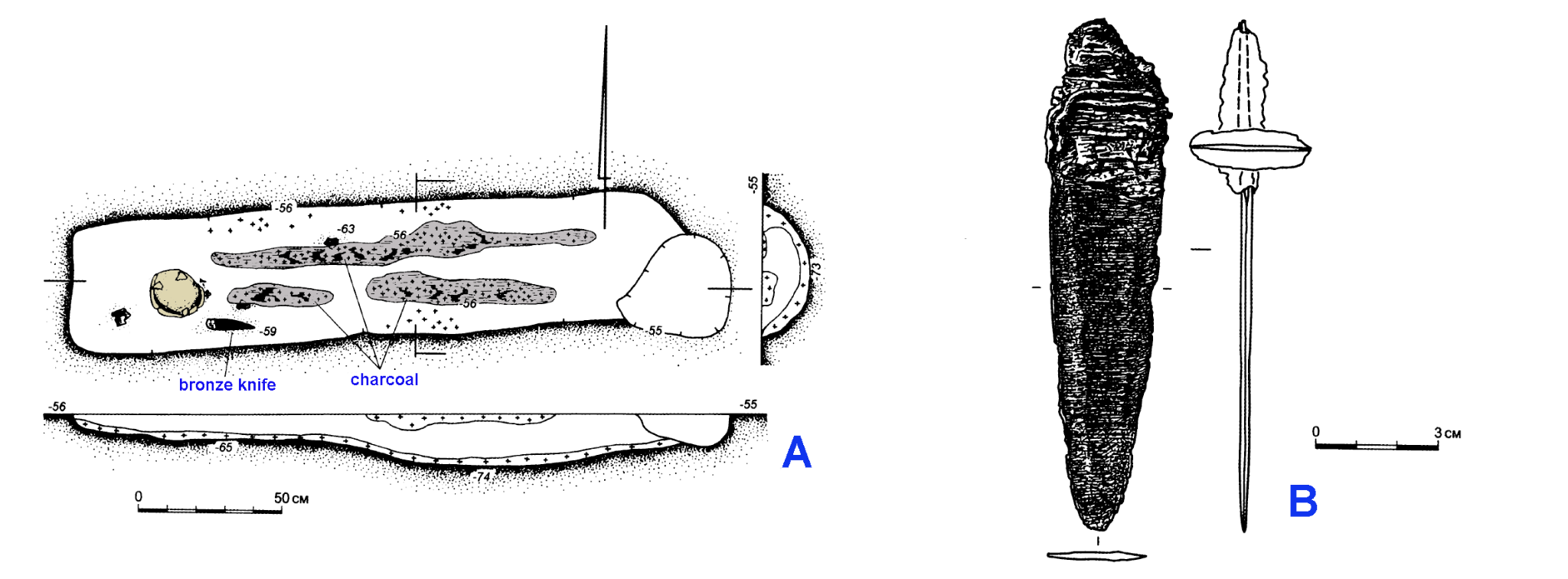


***Figure S88.*** *Burial 6 at the Satyga-16 site.* ***A*** *- burial;* ***B*** *- grave goods (image provided by Evgeniy Besprozvanny).*

###### Burial 17 (individual ID I32551, sex unidentified)

**Burial 17** suffered significant damage due to disturbance by uprooting, making it difficult to reconstruct its shape and parameters. It is believed that two graves were originally present at this location, oriented at an angle to each other. This is supported by the preserved cruciform outlines of the shared depression, as well as the presence of one skull within the pit and the discovery of another individual's skull not far from its eastern wall.

Presumably, a burial (**17a**) was initially constructed in this spot, aligned along the west-southwest axis, containing the remains of a 25-30-year-old man. Later, another pit (**17b**) was dug above it, oriented along a NNW-SSE line, approximately 0.5 m wide, in which a woman of about 35 years old was buried.

The sloping walls of this grave descended steeply to the bottom, reaching a depth of 0.2 m into the subsoil. In the redeposited layer of the uprooting pit, a bronze scraper, a bronze object resembling a chisel with remnants of a bone handle, a stone arrowhead, and a tool on a plate were found. Above the graves, an accumulation of jar vessel fragments was recorded, while fragments of a third vessel were uncovered to the south of the burials (Trufanov, 2011).

The sequenced skeleton (coming from **burials 17a** or **17b**) is dated to 2288–2058 calBCE (3770±25 BP, PSUAMS-12647).


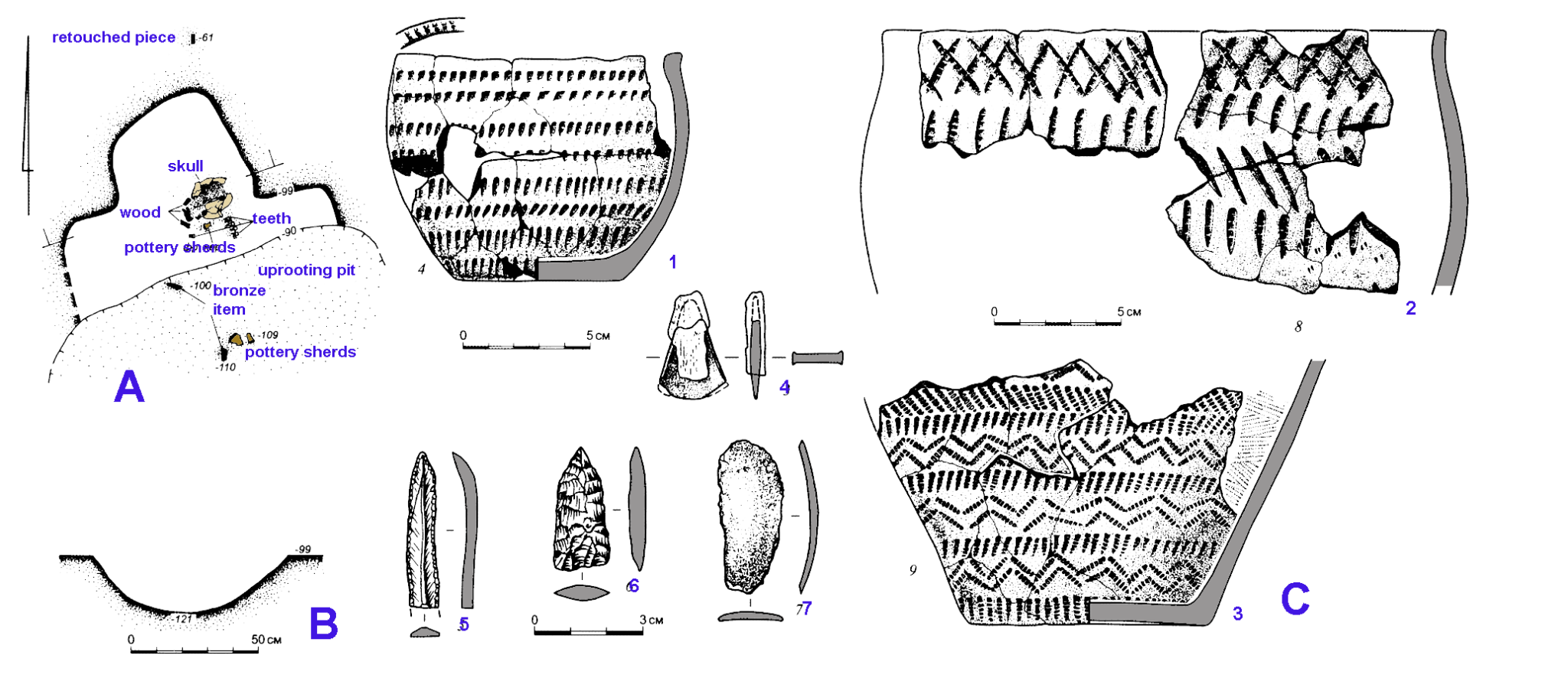


***Figure S89.*** *Burial 17 at the Satyga-16 site.* ***A*** *- burial;* ***B*** *- a section of the grave pit;* ***C*** *- grave goods (image provided by Evgeniy Besprozvanny).*

### Peripheral Area of the Seima-Turbino Phenomenon

##### Chernoozerye-1 burial site

At the Chernoozerye-1 burial site (described previously in Supplementary Information section 3.3.3.2.4), a grave possibly dated to the Seima-Turbino horizon among others that are from the Eneolithic and Late Bronze age periods was excavated. The presence of the Seima-Turbino phase is also evidenced by a find of an anthropomorhic figurine, with parallels in Rostovka and the Galich hoard (Stefanov, 2004).


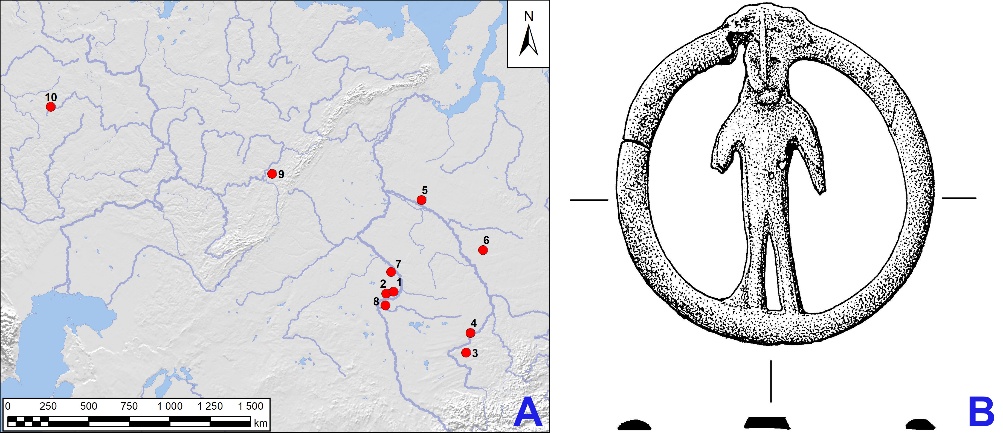


***Figure S90.*** ***A****: anthropomorphic figurines in the Bronze Age: 1 - Chernoozerye-1, 2 - Borovyanka-17, 3 - Zavyalovo-1A, 4 - Krokhalevka 13, 5 - Saigatino-6, 6 - Novy-Napas, 7 - Chudskaya-Gora, 8 - Rostovka, 9 - Bor-Lyonva, 10 - Galich treasure.* ***B****: antrhopomorhic figurine found at Chernoozerye-1 (image of the artifact is provided by Zhanna Trufanova (Trufanova 2003), map is based on Stefanov, 2004).*

###### Burial 132 (individual ID I6787, male)

The grave is aligned along the northwest-southeast axis, measuring approximately 230x75x30 cm. The southeastern part of the grave was disturbed by a ditch associated with an Early Iron Age mound, resulting in the displacement of the skull onto the chest bones. The deceased individual, presumed to be a female (genetically identified as a male) and estimated to be around 40 years old, is laid in a supine position with the head facing southeast. No grave goods accompanied this burial (Gening and Stefanova, 1994). The skeleton is dated to 2916–2884 calBCE (4285±20 BP, PSUAMS-2920).

The date looks too old for the Seima-Turbino period, but this fact can be explained by a freshwater reservoir offset of the radiocarbon dating. The dated individual had a fish-based diet, which is proved by the observed δ13C/δ13N ratio. The offsets in radiocarbon dates from the Seima-Turbino period sites are well observed in the Rostovka cemetery, where some dates demonstrate even older chronological attribution (see Table S2, dates on burials 29 and 34). Based on this observation and on the results of the genetic analysis, which revealed ancestry proportions characteristic for the Seimo-Turbino period individuals, we assume an Early-Middle Bronze Age chronological attribution of **burial 132** at the Chernoozerye-1 site.

##### Tatarka Hill site

In 1998, a flat burial ground on Tatarka Hill was discovered by A.S. Vdovin and S.A. Krasnolutsky. Excavations were conducted between 1998 and 2000 by the same archaeologists (Kuz’minykh, 2011).

The burial ground is situated on an elevated platform called  Tatarka Hill, located on the western outskirts of the village of Bolshoye Ozero, in the Sharypovsky District of the Krasnoyarsk Krai, Russia. The estimated area of the Tatarka burial ground is approximately 700-800 square meters. However, it is likely that a significant portion of the site was destroyed during the extraction of stone for road backfilling. Therefore, the original size of the site might have been around 1,500-2,000 square meters. The burials, comprising at least 50 individuals, are arranged in rows, closely spaced at intervals of several meters, located on a small elevated area that terminates in cliffs on the southwestern and southern sides. The orientation of the skeletons is towards the west-northwest. Grave goods found at the site include bronze celt axes, knives, awls, ornaments, jade rings, and stone arrowheads. Except for one flat-bottomed vessel, pottery is absent from the burials.

The cemetery at the Tatarka Hill includes graves of various periods; the latest phase dates to the Middle Ages, but most burials date back to the Late Bronze Age. The funeral rite and material culture of these burials reveal similarities with the sites of the forest-steppe “andronoid” cultures of Western Siberia (such as Elovka-2, Sopka-2, Chernoozerye-1). Among the metal artifacts found are single-edged and, less commonly, double-edged knives, “horned” bracelets, rings covered with gold foil, various plate decorations, etc. In burials 14 and 15, two false-eared celts of type K–54 of the Samus’-Kizhirovo tradition were found.

Another group of burials is characterized by connections with the cultures of the Baikal region, as indicated by stone burial structures and jade rings (Kuz’minykh, 2011). A group of burials in shallow grave pits, oriented in the northwest to southeast direction, contained individuals laid in supine positions with their heads to the southeast. The grave pits were partially covered by stone slabs. The burials lack grave goods, and their chronology could be estimated only based on radiocarbon datings.

We sequenced four individuals from Tatarka and dated two of them. The radiocarbon dates on human bones/teeth [2288–2058 calBCE (3770±25 BP, PSUAMS-9048) and 2021–1884 calBCE (3585 BP±20, PSUAMS-7545)] reveal the attribution of these burials to the Seima-Turbino period and distinguish them from the Late Bronze Age Andronovo-like burials. The freshwater reservoir offset of these dates might have explained the incongruity of the C14 data and the archaeological context, but the isotopic data reveals the highest values of δ13C among all our analyzed samples from Siberia and a relatively low value of δ13N. Thus, the δ13C/δ13N ratio supports a foraging-hunting diet rather than the intensive fish consumption by the studied individuals.  Based on this observation, we assume an Early-Middle Bronze Age phase at the Tatarka-Hill cemetery.

We found the closest parallels to the burial rites observed in those Early-Middle Bronze Age graves of the Tatarka-Hill site, in burials of the Nefteprovod-1 and 2 cemeteries of the Anzhevskiy ensemble located in Kansk forest-steppe. Alongside the geographical proximity, the Tatarka-Hill and the EMBA burials of the Anzhevski ensemble share attribution to the same chronological period.

###### Burial 2 (individual ID I20305, male)

The grave pit is covered with stone slabs, and additional slabs are placed along the sides of the grave pit. The shape of the grave pit is elongated and oval, extending in the northwest to southeast direction.

At a depth of 50-60 cm from the surface, the remains of a single individual, estimated to be 25-35 years old, were discovered. The skeleton was found in a supine position. The skeletal remains are in a poorly preserved condition, with damage to the outer layer (cortical layer) of the bones. The skull has been restored, and only small fragments of the pelvis, ribs, vertebrae, as well as the bones of the feet and hands, have survived, as the epiphyses of the long bones of the postcranial skeleton are all destroyed.

Dental and jaw pathologies observed include caries, evidence of tooth loss during the individual's lifetime, and abscesses on the upper and lower jaws, along with chips on the teeth. Arthrosis is present in the thoracic and lumbar spine, as well as in the elbows and hands of the upper extremities.

No associated grave goods or additional items were found in the burial. The skeleton is dated to 2288-2058 calBCE (3770±25 BP, PSUAMS-9048).

###### Burial 26 (individual ID I10998, female)

The grave pit exhibits an oval shape elongated in the northwest-southeast direction. The interred individual's remains were found in a supine position. The skeleton belonged to a woman estimated to be 20-25 years old, and it was discovered at a depth of 35-40 cm below the soil surface. Based on the overall orientation of the remains, the deceased was positioned with her head towards the south-southeast. The skeletal preservation is very poor, with fragments of the skull (occipital part of the braincase and frontal bone showing outer cortical layer destruction, along with a lower jaw displaying damaged condyles). The diaphyses of long bones in the postcranial skeleton show evidence of destruction of the epiphyses. Additionally, fragments of the pelvis, shoulder blades, ribs, vertebrae, as well as bones from the hands and feet were recovered.

The accompanying grave goods consist of a fang, possibly of a wolf or dog. The skeleton is dated to 2021–1884 calBCE (3585±20 BP, PSUAMS-7545).

###### Burial 27A (individual ID I20547, female)

The grave pit of **burial 27A** was uncovered at a depth of 25-30 cm from the contemporary surface. It has an oval shape elongated in the northwest-southeast direction and is partially covered by stone slabs. The interred individual, a woman estimated to be 35-40 years old, was found in a supine position with her head towards the southeast. The skeletal remains are highly deteriorated, with only small fragments of the skull remaining (due to poor preservation, the skull was excavated as a monolith with the surrounding soil, making restoration impossible). The lower jaw exhibits a destroyed left condyle. Among the postcranial skeleton, fragments of the humerus, femur, tibia, one fibula, small fragments of the pelvic bones, and the first two cervical vertebrae were recovered. Signs of arthrosis are evident in the cervical spine.

No associated grave goods or additional items were found in the burial. The skeleton is dated to 2134-1936 calBCE (3645±25 BP, PSUAMS-14846).

###### Burial 55 (individual ID I20549, female)

The grave pit of **burial 55** was discovered at a depth of 35-40 cm from the present surface. It possesses an elongated-trapezoidal shape oriented in the north-south direction and is partially covered with stone slabs. The interred individual, a woman estimated to be 30-35 years old, was found in an extended supine position. Observations on the skeleton include missing teeth, an abscess in the region of the lower second premolar, and evidence of periodontal disease. Arthrosis is apparent in various areas, including the cervical, thoracic, lumbar, lumbosacral, and sacroiliac regions, as well as the left elbow, wrist, and hand.

No associated grave goods or additional items were found in the burial. The skeleton is dated to 2454-2201 calBCE (3840±25 BP, PSUAMS-14880).

##### The Anzhevsky Complex (Nefteprovod-1 & Nefteprovod-2 sites, and Tatarka Hill)

The archaeological sites Nefteprovod-1 and Nefteprovod-2 of the Anzhevsky ensemble were discovered in 1972 by N.A. Savelyev. The ensemble is located in the Kansk forest steppe, on the right bank of the Kan River, in Ilansky district of the Krasnoyarsk Krai (Vybornov et al., 2015). The excavations in 2015-2016 carried out at the sites Nefteprovod-1 and Nefteprovod-2 revealed 29 burials at the multi-phase burial ground. The burials were preliminary attributed to several phases: the Neolithic, the Late Bronze Age (Krasnoyarsk culture), the Bronze Age (taiga cultural circle), the Early Iron Age, and the Middle Ages (Timoshchenko, Vybornov, Rybin, 2018).

The first burial at the Nefteprovod-2 site was excavated in 2011 by E.V. Knyazeva (Mandryka et al., 2018) in a survey excavation block. The skeleton was laid in a supine position with his head to the southeast, no grave goods were associated with the burial. The **buried individual** was studied genetically (individual ID kra001, sex was not identified) and dated to 2340-2064 calBCE (3790±30 BP, Beta-453083) (Kılınç et al., 2021).

Further excavations of 2011 and 2015 revealed other burials dated to the same period and located around the first one or in other areas in the Nefteprovod 1-2 sites. A group of burials around the first one were numbered from 1 to 8. They are located on the low terrace at heights 216.49 - 217.65 m (ASL), 12-13 m above the river water level, in an area of 22x21 m. The graves were placed in three rows, elongated from south to north (up the slope from the river bank). The eastern row includes graves 1, 2, 5, 6, and 8, the middle row – the grave excavated in 2011 and burial 4, and the western one includes burial 7.


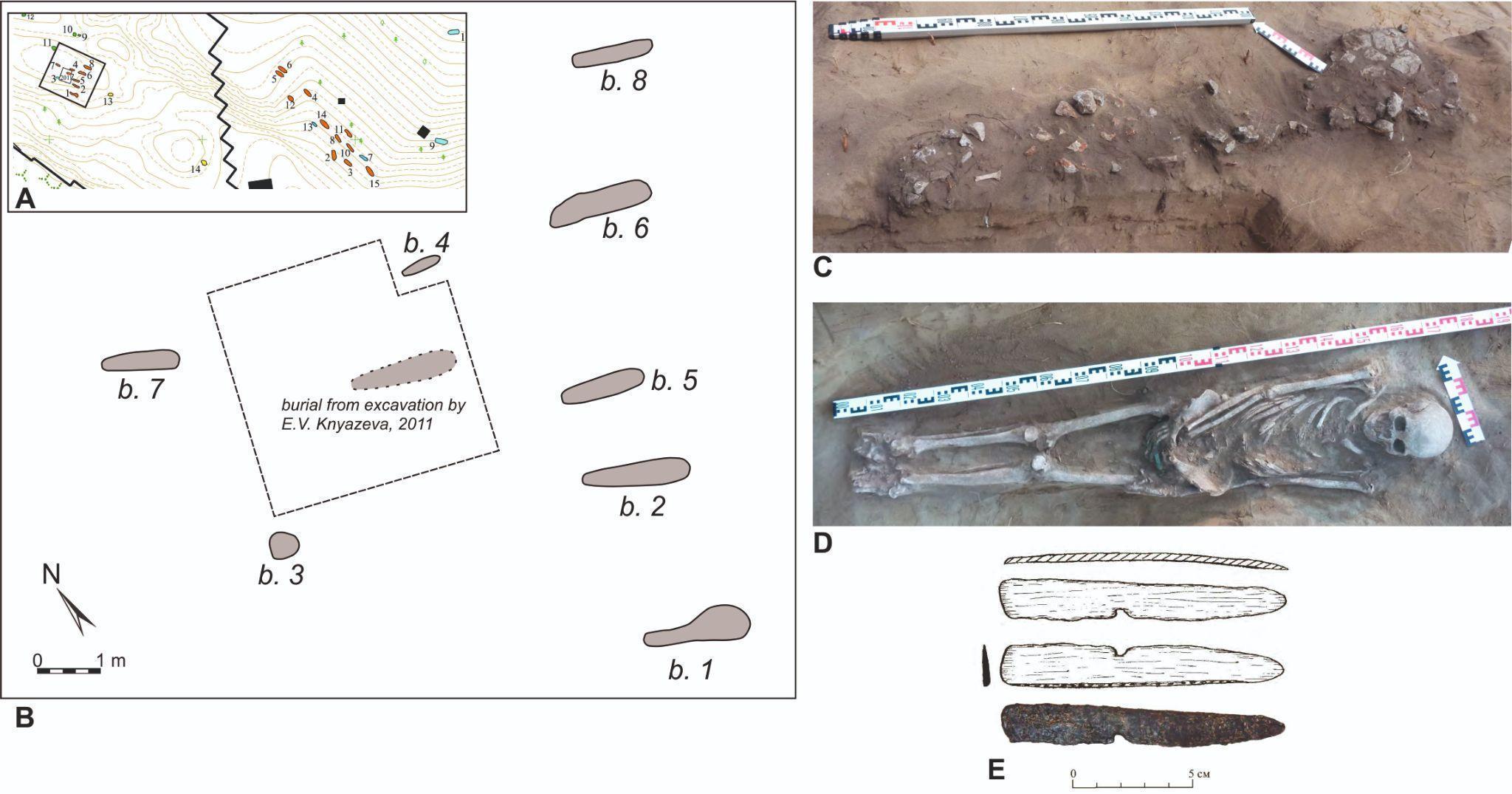


***Figure S91.*** *Anzhevsky ensemble.* ***A:*** *layout of burials at the archaeological sites Nefteprovod-1 and Nefteprovod-2.* ***B:*** *layout of burials of the lower group at Nefteprovod-2.* ***C:*** *masonry over burial 2 at Nefteprovod-2.* ***D:*** *skeleton in burial 2 at Nefteprovod-2.* ***E:*** *bronze knife in burial 2 at Nefteprovod-2 (image by Anton Vybornov).*

All these graves were sunk from the top of the second lithological layer that contained Neolithic and Bronze Age artifacts. The grave pits were amorphous and their boundaries were hard to recognize. The skeletons were put in the anatomical order in supine positions with their heads oriented upstream, to the southeast. The faces were oriented upwards, or slightly tilted to the right, when recognizable. Traces of fire were recognized on the bones of burial 5, and charred birch bark in burials 5 and 8. Elongated rubbleworks masonry (1.03–1.95 × 0.22–0.43 m) were recorded in burials 1, 2, 6, 7, and 8. Separate stones were found above burial 5. In burials 2, 6, and 8 the skeletons were complete, and in burials 1, 4, 5, and 7 some bones were missing. Burial 4 had no rubblework and its gravepit was relatively smaller, 0.67 × 0.18 m; it contained remains of a 5-6 year old child.

Grave goods include bronze knives from burials 2 and 4, bronze beads from burial 5, and fragments of bronze items on a skeleton in burial 8. No grave goods were recorded in burials 1, 6, and 7.

Another group of burials dated to the same period as individual kra001 was discovered 120 m east of it, on the edge of the upper terrace, at the Nefteprovod-1 site, in 2015-2016. It includes graves 2-6, 8, 10-12, and 14-15. The graves of this group are located along the edge of the terrace in three rows, elongated from the northwest to southeast, at the heights of 219.3 - 223.74 m (ASL), 15-19 m above the river water level. The graves are elongated along a northwest-southeast line, the skeletons were put in supine positions, their heads are oriented to the southeast. Above graves 2 and 3 some stones were found, above graves 5, 6, and 14 an elongated rubblework was recorded, and above grave 8 a circle-shaped rubblework was recorded. In grave 10, a bronze knife and a bronze piece were found near the skull.

Radiocarbon dates have been obtained for two burials of this group of graves, burial 14 (4248–3902 calBP (3733±58 BP, GV-02873)) and burial 15 (3886–3574 calBP (3473±59 BP, GV-02874)).


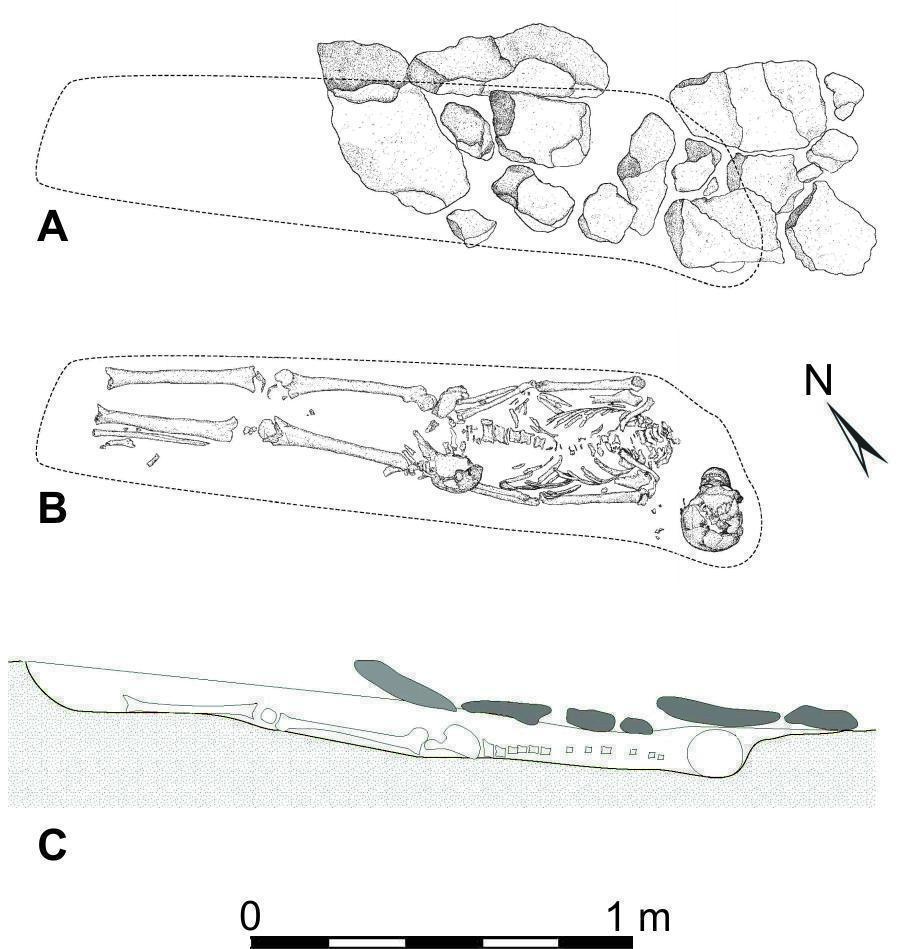


***Figure S92.*** *Anzhevsky ensemble.* ***A:*** *rubblework over burial 14 at Nefteprovod-1.* ***B:*** *skeleton in burial 14 at Nefteprovod-1.* ***C:*** *longitudinal section of burial 14 at Nefteprovod-1 (image by Anton Vybornov).*

In conclusion, we identify a group of burials at the Nefteprovod 1 and 2 sites that share common details in burial rites and are dated to 4200–3900 calBP.

At the Nefteprovod-2 site, those are burials in shallow and narrow grave pits containing skeletons put in supine positions with their skulls in the southeastern parts of grave pits. Above some burials, elongated rubbleworks were recorded. In some cases, scarce grave goods were found, including bronze knives in the hands of the dead and beads in the head areas. In two cases, there were traces of fire on the bones and fragments of charred birch bark. One of the burials of this group, located in the middle row and explored in 2011 by E.V. Knyazeva has a radiocarbon date of 4280–4085 calBP [Kılınç et al., 2021). Apparently, the entire group of similar burials should be dated to the same period.

At the Nefteprovod-1 site, a group of burials could be associated with the same population as the individual kra001. They share such burial rites details as shallow grave pits, the supine position of the dead, orientation to the southeast, and presence of rubble works covering the graves. Some differences were also distinguished in the planigraphy of the rows of graves, the location of bronze knives, and the absence of any traces of fire on the bones of the buried persons.

## Cisbaikal_LNBA-rich outliers from the Krasnoyarsk region

The Late Bronze Age period in the Minusinsk Basin has a long history of study and interpretation. The first steps of it are dated back to the 1920s century when S.A. Teploukhov described it as the Karasuk culture. Several decades later, due to accumulation of data nonuniformity of the Karasuk-associated assemblage became apparent, and  M.P. Gryaznov was the first researcher who in the 1960s reattributed some "non-typical" Karasuk artifacts as a specific phase of the cultural development called Kamenny-Log. Later, the chronological definitions of the "Classic Karasuk" and the "Kamenny-Log Karasuk" were elaborated, and the latter one was attributed to 1000–800 BCE while the "classic" one to an earlier date, 1300–1000 BCE (Gryaznov 1979).

Although this view became conventional among most archaeologists, being supported by G.A. Maksimenkov, B.N. Pyatkin, and others (Maksimenkov 1975), some researchers were inclined to interpret the "non-typical" Karasuk assemblage as a specific cultural group, and not as a stage of linear cultural development. This opinion was most clearly articulated by N.L. Chlenova, who described them as a specific Lugavskaya culture, which, she postulated, developed from local Neolithic and Okunevo traditions and later co-existed with the Karasuk one in the Minusinsk Basin during 1400/1300–700/600 BCE (Chlenova 1972).

Later studies expounded one of these two competing models in one way or another. In the most recent publication, a compromising model was suggested by A.V. Polyakov (2022). He subdivided the Late Bronze Age period in the Minusinsk basin into several stages (calendar dates are approximate):

- LBA-I (Karasuk) with substages A and B - 1450–1250 BCE;
- LBA-II (Karasuk-Lugavskaya) - 1300–1075 BCE;
- LBA-III (Lugavskaya)  with substages A, B, and C - 1025–825 BCE;
- LBA-IV (Bainov)  with substages A and B - 850–750 BCE.

This chronological scheme slightly modifies earlier models of stadial cultural development. Polyakov and Lazaretov (2008) assume the gradual replacement of the Karasuk population by newcomers.
[truncated: 402,115 more chars]
