# Supplementary material for: Ancient DNA reveals the prehistory of the Uralic and Yeniseian peoples
Source: Nature. Author manuscript; Available in PMC 2025 Aug 12. (PMC12342343; doi:10.1038/s41586-025-09189-3)
Supplement: Supplementary Information Guide [file NIHMS2095973-supplement-Supplementary_Information_Guide.docx]

**Supplementary Information Guide**

**Ancient DNA reveals the prehistory of the Uralic and Yeniseian peoples**

# **Supplementary Information (all supplementary Information as concatenated PDF)**

This file includes Supplementary Information sections 1-11, figures S1-S101, and Tables S1-S35. It includes discussion of archaeological context, details of sample preparation, details of population genetic analysis udsing PCA, *ADMIXTURE*, *qpAdm* and other formal methods such as *f_4_*-statistics, relatedness analysis, uniparental markers, and also linguistic discussion and archeological interpretation.

## Supplementary Information section 1

Supplementary Information Section 1 discusses the geophysical and climatic context for our samples.

- In 1.1, we introduce the geographic terms that we use throughout this manuscript and in the Supplementary Information.
- In 1.2, we introduce important climatic and biogeographic zones to provide context for important geographic terms such as “forest zone” or “forest-steppe”.
- In 1.3, we discuss the role climatic changes may have played in the migrations and genetic transformations of Northern Eurasia discussed in this manuscript.

References are listed at the end of the section.

## Supplementary Information section 2

Details the procedure by which we obtained Radiocarbon and Isotope data for our samples. We present plots of isotope ratios, and discuss how they rule out the possibility of reservoir effects for samples from some cultures (e.g. the “Anzhevsky complex”), while increasing the probability of such effects for some others.

## Supplementary Information section 3

Presents the archaeological context for all individuals newly published in this manuscript, as well as for important previously-published individuals. It is organized geographically and thematically.

For sections 3.1-3.5, each section covers a subregion of Northern Eurasia as defined in Supplementary Information section 1. For each region, we first discuss major archaeological cultures and cultural chronology for that region, before going into the specific cultures and sites from which the anthropological materials were sampled.

- In 3.1, we discuss archaeological sites and cultures of the Eastern part of Central Siberia, including the Sumnagin and Dyuktai cultures (3.1.1.1), as well as the Ymyyakhtakh culture (3.1.1.4). In 3.1.1.4, we also discuss the regional distribution of Bone armor in Siberia.
- In 3.2, we discuss archaeological sites and cultures of the Southern part of Central Siberia and the Baikal region, including the Bronze Age dispersal of the Glazkovo culture and pottery to the Middle Angara (3.2.2.3).
- In 3.3, we discuss archaeological sites and cultures of the Southern part of Western Siberia and the Altai-Sayan region, including the Kuznetsk-Altai culture (3.3.2.1).
- In 3.4, we discuss archaeological sites and cultures of the Urals.
- In 3.4, we discuss archaeological sites and cultures of the Temperate zone of Eastern Europe.

The next sections, 3.6 to 3.7, are thematically organized.

- As a trans-cultural and geographically widespread phenomenon, the Seima-Turbino phenomenon and Seima-Turbino-period individuals are discussed under their own sections (3.6).
- In section 3.7, we conducted further archaeological investigation into two previously-published Late Bronze Age outliers high in Cisbaikal_LNBA ancestry, and conclude that they derive from the Lugavskaya culture.

References are listed at the end of the section (3.8).

## Supplementary Information section 4

Covers the procedures we used to generate each of the PCAs in our paper, as well as the major inferences we can draw from them.

## Supplementary Information section 5

Covers the ADMIXTURE analysis.

## Supplementary Information section 6

Covers the procedure, based on f_4_-statistics, that we use to cluster individuals from Central Siberia, Northeast Siberia, and the Baikal region into genetic populations, forming a 10-member transect that we analyze further.

## Supplementary Information section 7

Introduces and discusses the the major sets of qpAdm analyses that we perform in the next sections (sections 8, 9, 10, 11 and 13), as well as procedures that we took to make sure our results were robust to allelic biases.

## Supplementary Information section 8

Presents detailed analyses of the 10-member transect produced in Supplementary Information section 5. For a summary of our overall conclusions, refer to section 8.1.4. For how our conclusions also match simple patterns in f4-statistics, refer to figures S94-96.

For how Cisbaikal_LNBA as a “Route 1” population shares high levels of drift with present-day populations around the Yenisei, but Yakutia_LNBA, Syalakh-Belkachi, Kolyma_M_10.1kya and Dzhilinda_M_N_8.4kya as members of “Route 2” share high levels of drift with present-day populations around the Bering Straits, refer to Figure S94 and S95 and the discussion in 8.1.4.1.

## Supplementary Information section 9

Investigated the relationship between members of the 10-member transect in Section 8 with later populations on either side of the Bering Straits.

## Supplementary Information section 10

Presents qpAdm analyses of NEAHG cline populations.

## Supplementary Information section 11

Investigates the genetic legacy left by Cisbaikal_LNBA populations in later AIEA (Admixed Inner Eurasian) populations.

- We discuss evidence from f4-statistics that suggests Cisbaikal_LNBA is important for AIEA populations around the Yenisei River (Supplementary Information section 11.1, Figure S101).
- For how Cisbaikal_LNBA is a source in all passing models for target populations around the Yenisei river, refer to Figure S99 and the discussion in Supplementary Information section 11.2.
- We discuss how we ensure the robustness of our major conclusions to allelic biases introduced by different wet-lab procedures (Supplementary Information section 11.3).
- Includes a discussion of Y-chromosomal evidence corroborating the results of autosomal analyses (Supplementary Information section 11.4).
- We discuss qpAdm models of two Cisbaikal_LNBA-rich outliers of the Lugavskaya culture in section 11.5.

## Supplementary Information section 12

Linguistic notes on the Yeniseian urheimat and the history of language contact between Yeniseian languages and populations speaking other languages.

## Supplementary Information section 13

Investigates the genetic legacy left by Yakutia_LNBA populations in later AIEA (Admixed Inner Eurasian) populations.

- We discuss evidence from f4-statistics that suggests Yakutia_LNBA is important for Uralic-speaking populations (Supplementary Information section 13.1, Figure S101).
- For how Yakutia_LNBA is a source in all passing models for target populations that are Uralic-speaking, refer to Figure S100 and the discussion in Supplementary Information section 13.2.
- We discuss how we ensure the robustness of our major conclusions to allelic biases introduced by different wet-lab procedures (Supplementary Information section 13.3).
- Includes a discussion of Y-chromosomal evidence corroborating the results of autosomal analyses (Supplementary Information section 13.4).
- We discuss qpAdm models of Tatarka_BA showing that they may have been part of the initial dispersal of Yakutia_LNBA ancestry west in section 13.5.

## Supplementary Information section 14

Linguistic notes on the history of language contact between Uralic languages and other languages.

## Supplementary Information section 15

Details the qpAdm analyses we performed for individuals from Seima-Turbino-period sites and contextualizes these findings alongside results from other methods such as ADMIXTURE and PCA.

# **Supplementary Data S1-S7 (seven individual Excel files)**

## Supplementary Data 1

Is an Excel file with details of all published and newly reported individuals used in this analysis; each table is on a different sheet or tab.

- The sheet labeled *Present-day Individuals* Supplementary Data S1 Table 1 lists all present-day individuals used in any analysis.
- The sheet labeled *Ancient Individuals* lists all ancient individuals used in analysis, as well as three additional newly reported individuals that were detected as first-degree relatives of other individuals with higher coverage (I11747, I13100, I11744), that were not used in analyses as their higher-coverage relative was used instead.
- The sheet labeled *Population Labels of Ancients:* Due to the large number of analyses and the different roles that any given individual sample may play in each analysis, this sheet presents individual-level information about the clustering results, group labels (which determine the individual’s membership in grouping used for *qpAdm* and other analyses) and groupings (e.g., for color and point-shape assignment in PCAs) for each ancient and present-day individual across any analysis in this paper.
- The sheet labeled *C14 & Stable Isotopes* presents C14 and isotopic data for newly-reported samples.
- The sheet labeled *Table of Archaeological Cultures* presents a synchronization table for all newly-reported samples and their contemporaneous archaeological entities.
- The sheet labeled *Bone samples and Libraries* presents data on all bone samples and libraries.
- The sheet labeled *Newly-Published samples* presents a subset of the data in Table 1, restricted to all newly-published samples.

## Supplementary Data 2

Presents results (all models, whether passing or failing, of the combinatorial rotating outgroup *qpAdm* protocol) for all *qpAdm* sets for *NEAHG* populations and other ancient Siberians that are combinations of *EHG*, *ANE* and East Asian ancestry. For details of how each table of qpAdm results is organized and sorted, see Methods section under the sub-section “f4-statistics & qpAdm”. The file contains two sheets:

- The sheet labeled *Distal NEAHG qpAdms* presents distal qpAdms of populations along the NEAHG cline.
- The sheet labeled *Proximal NEAHG qpAdms* presents proximal qpAdms of populations along the NEAHG cline.

There are two sets of results in two tables (separate sheets). Details on the procedures used to produce the results can be found in Supplementary Information section 10 For more information on how the tables of qpAdm results are organized, see Methods section under the sub-section “f4-statistics & qpAdm”.

## Supplementary Data 3

Presents results (all models, whether passing or failing, of the combinatorial rotating outgroup *qpAdm* protocol) for all *qpAdm* sets for East Siberian populations, aimed at investigating their mutual relationships, with the results summarized in Figure 2C. There are 16 sets of qpAdm results, on 16 sheets (for details of how each table of qpAdm results is organized and sorted, see Methods section under the sub-section “f4-statistics & qpAdm”). one sheet of f4-statistics, one sheet labeled “Individuals Involved” in Supplementary Data 3. We recommend reviewing these results under the guidance of our detailed modeling procedure presented in Supplementary Information Section 8.

- The sheet labeled *Individuals Involved* presents the individuals assigned to each population and used across all *qpAdm* analyses (i.e., used in the calculation of f2-statistics; see “qpAdm and f4-statistics” under the “Methods” section of the Main Text) in Supplementary Information Section 8.
- The sheet labeled *Tranche 1 (Naïve)* presents qpAdm results discussed in Supplementary Information section 8.1.2.1.
- The sheet labeled *Tranche 2 (Naïve)* presents qpAdm results discussed in Supplementary Information section 8.1.2.2.
- The sheet labeled *Tranche 3 (Naïve, Set A)* presents qpAdm results discussed in Supplementary Information section 8.1.2.3 and 8.1.3.1.
- The sheet labeled *Tranche 3 (Naïve, Set B)* presents qpAdm results discussed in Supplementary Information section 8.1.2.3.
- The sheet labeled *Tranche 3 (Set B)* presents qpAdm results discussed in Supplementary Information section 8.1.3.1 and 8.1.3.2.
- The sheets labeled *Tranche 4 (Set A)* & *Tranche 4 (Set B)* presents qpAdm results discussed in Supplementary Information section 8.1.3.3.
- The sheet labeled *Tranche 4 (Naïve, Set A)* & *Tranche 4 (Naïve, Set B)* presents qpAdm results discussed in Supplementary Information section 8.1.2.4.
- The sheet labeled *Tranche 5 (Naïve, Set A)* & *Tranche 5 (Naïve, Set B) & 6* presents qpAdm results discussed in Supplementary Information section 8.1.2.5. and 8.1.2.6.
- The sheet labeled *Tranche 5 (Set A)* & *Tranche 5 (Set B) & 6* presents qpAdm results discussed in Supplementary Information section 8.1.3.4.
- The sheet labeled *Tranche 6 (Naïve, Set B)* presents qpAdm results discussed in Supplementary Information section 8.1.2.6.
- The sheet labeled *Cisbaikal_LNBA Tranche-Finding* presents qpAdm results discussed in Supplementary Information section 8.1.3.1.
- The sheet labeled *Symmetry tests* in Supplementary Data 3 presents a set of *f_4_*-statistics discussed in further detail in Supplementary Information section 8.1.4.3.

Details of the procedures used to produce the 16 sheets of *qpAdm* results can be found in Supplementary Information section 8.1, and details of the procedures used to produce the *f_4_*-statistics can be found in Supplementary Information section 8.1.4.3.

## Supplementary Data 4

Presents results of the clustering analysis in Supplementary Information section 6. Details of the procedures used to produce the *f_4_*-statistics can be found in Supplementary Information section 6.1.

- The sheet labeled *Individuals Involved* presents individuals involved in the analysis and their cluster memberships.
- The sheets labeled *Siberia, Grouping F4s* and *Siberia, Grouping F4s (Transv)* present the results of the F4 statistics used for the clustering procedure detailed in Supplementary Information section 6.1 for individuals from Siberia East and North of Lake Baikal. The results on the sheet *Siberia, Grouping F4s (Transv)* restricts the analyses to transversions only. The results are discussed in greater detail in Supplementary Information section 6.2.
- The sheets labeled *Cisbaikal, Grouping F4s* and *Cisbaikal, Grouping F4s (Transv)* present the results of the F4 statistics used for the clustering procedure detailed in Supplementary Information section 6.1 for individuals from the Cisbaikal region. The results on the sheet *Cisbaikal, Grouping F4s (Transv)* restricts the analyses to transversions only. The results are discussed in greater detail in Supplementary Information section 6.3.
- The sheet labeled *Group Homogeneity qpAdms* lists qpAdm results that test for group homogeneity for the clusters produced in Supplementary Information section 6.2, and are further discussed there.

## Supplementary Data 5

Presents results of the *qpAdm* analyses (all models, whether passing or failing, of the combinatorial rotating outgroup *qpAdm* protocol) described in Supplementary Information section 9, where ancient populations from either side of the Bering Strait are modeled as descending from the previously modeled populations in Siberia in Supplementary Information Section 8. One sheet presents the individuals assigned to each population used across all qpAdm models in Supplementary Information Section 9. There are also three sets of qpAdm results shown in three sheets.

- The sheet labeled *Individuals Involved* lists all individuals involved in the analysis (i.e., individuals used in the calculation of f2-statistics; see “qpAdm and f4-statistics” under the “Methods” section of the Main Text), and their group label assignments in all qpAdms detailed in Supplementary Information section 9.
- The sheet labeled *Saqqaq qpAdms* presents all passing models for the Saqqaq Paleo-Inuit. They are discussed in Supplementary Information Section 9.1.
- The sheet labeled *Beringian qpAdms* presents qpAdm results discussed in Supplementary Information section 9.2. & 9.3.
- The sheet labeled *Beringian qpAdms (NNA+SNA)* presents qpAdm results discussed in Supplementary Information section 9.2. & 9.3.

## Supplementary Data 6

Presents results of the *qpAdm* analyses (all models, whether passing or failing, of the combinatorial rotating outgroup *qpAdm* protocol) described in Supplementary Information section 11 and 13, where the *Yakutia_LNBA* and *Cisbaikal_LNBA* admixture proportions among AIEA populations are estimated using *qpAdm*. There is one sheet detailing the individuals assigned to each population used across all the qpAdms detailed in Supplementary Information Sections 11 and 13, six sets of qpAdm results on six sheets, and six sheets summarizing and comparing the qpAdm results. We recommend reviewing the results under the guidance of the text in Supplementary Information sections 13 and 11, starting with 13 and ending with 11.

- The sheet *AIEA Yakutia_LNBA qpAdms* presents the results of the qpAdm detailed in Supplementary Information section 13.2.
- The sheet *Results (Yakutia_LNBA)* summarizes the passing models in the sheet *AIEA Yakutia_LNBA qpAdms.*
- The sheet *AIEA Yakutia_LNBA qpAdms (1240K)* presents the results of the qpAdm detailed in Supplementary Information section 13.2, but with all shotgun-sequenced samples removed from all group labels (i.e. only 1240k sequences retained).
- The sheet *Results (Yakutia_LNBA 1240k)* summarizes the passing models in the sheet *AIEA Yakutia_LNBA qpAdms (1240K).*
- The sheet *Yakutia_LNBA Comparison Table* compares the passing models in *Results (Yakutia_LNBA)* against the passing models in *AIEA Yakutia_LNBA qpAdms (1240K)* to show that the major conclusions drawn from qpAdm analyses presented in Supplementary Information section 13 are robust to the effects of allelic bias, discussed in more detail in Supplementary Information section 13.3.
- The sheet *AIEA Cisbaikal_LNBA qpAdms* presents the results of the qpAdm detailed in Supplementary Information section 11.2.
- The sheet *Results (Cisbaikal_LNBA)* summarizes the passing models in the sheet *AIEA Cisbaikal_LNBA qpAdms.*
- The sheet *AIEA Cisbaikal LNBA qpAdms (1240K)* presents the results of the qpAdm detailed in Supplementary Information section 11.2, but with all shotgun-sequenced samples removed from all group labels (i.e. only 1240k sequences retained).
- The sheet *Results (Cisbaikal_LNBA 1240k)* summarizes the passing models in the sheet *AIEA Cisbaikal_LNBA qpAdms (1240K).*
- The sheet *Cisbaikal_LNBA Comparison Table* compares the passing models in *Results (Cisbaikal_LNBA)* against the passing models in *AIEA Cisbaikal_LNBA qpAdms (1240K)* to show that the major conclusions drawn from qpAdm analyses presented in Supplementary Information section 11 are robust to the effects of allelic bias, discussed in more detail in Supplementary Information section 11.3.
- The sheet *Tatarka_BA qpAdms* presents qpAdm results for the Tatarka_BA population, discussed in Supplementary Information section 13.5.

## Supplementary Data 7

Presents the results of the *qpAdm* analyses (all models, whether passing or failing, of the combinatorial rotating outgroup *qpAdm* protocol) described in Supplementary Information section 15, investigating the ancestry of Seima-Turbino (ST) individuals. Includes one sheet indicating the individuals involved in all qpAdm analyses and their group label assignments, and three sets of qpAdm results in three sheets.

- The sheet labeled *Individuals Involved* lists the individuals used in the qpAdm analyses (i.e., individuals used in the calculation of f2-statistics; see “qpAdm and f4-statistics” under the “Methods” section of the Main Text),.across all qpAdm analyses detailed in Supplementary Information section 15.
- The sheet labeled *Distal ST qpAdms* lists the results of the distal qpAdm analyses detailed in Supplementary Information section 15.
- The sheet labeled *Proximal ST qpAdms* lists the results of the proximal qpAdm analyses detailed in Supplementary Information section 15.
- The sheet labeled *Proximal ST qpAdms + Cisbaikal* lists the results of the proximal qpAdm analyses, with Cisbaikal_LNBA used as an additional source, detailed in Supplementary Information section 15. This analysis is especially relevant for individual I32816.

# **Supplementary Images in Supplementary Information**

We list each image with its title, legend, and any references.

## Figure S1: Geographic regions of Northern Eurasia.

1 - East European Plain, 2 - Urals, 3 - West Siberia, 4 - Central Siberia (4.1 - Central Yakutia, 4.2 - Aldan), 5 - Altai-Sayan (5.1 - Kuznetsk-Salair, 5.2 - Altai, 5.3 - Sayan with 5.3.1 - Minusinsk Basin, 5.4 - Tyva), 6 - Baikal (6.1 - Baikal, 6.2 - Transbaikalia, 6.3 - Baikal-Stanovoy), 7 - North-East Siberia, 8 - North Pacific, 9 - Amur-Sakhalin (8 and 9 taken together are called Russian Far East) (boundaries of regions and provinces based on Map of the physical-geographical zoning of the USSR 1983, Gvozdevskiy, N. A., & Mikhailov, N. I. (1978); biogeographic regions Olson et al. 2001, generalized).

## Figure S2: Rivers and River Basins of Northern Eurasia

The rivers discussed in the article are highlighted.

**Figure S3. δ13С and δ15N isotopic collagen values of newly dated human bone samples (top), and against modern fish from Eurasia (bottom).**

## Figure S4. The Kangalassy burial.

**A** - burial at the moment of discovery; **B** - grave goods (by Novgorodov, 1960, modified).

## Figure S5. Grave goods from the Ogonyok burial.

**1-3** - blades; **4** - prismatic core; **5** - flint arrowhead; **6** - bone needle; **7** - bone composite arrowhead with a blade in the groove; **8** - bone polisher; **9** - anthropomorphic figurine made from a mammoth tusk; **10** - polished chisel (by Alekseev et al., 2022).

## Figure S6. Neolithic sites in the Baikal region.

1 – Rasputino; 2 - Ust'-Belaya; 3 – Obkhoy; 4 - Manzurka-2; 5 – Fofonovo; 6 - Ulyarba-2; 7 - Khuzhir-2.

## Figure S7. Map of Ulyarba site.

**A** - sequenced burials; **B** - other Neolithic and Bronze Age burials; **C** - Early Iron Age burials; **D** - rocks (by Goryunova 2002, modified).

## Figure S8. Ulyarba site.

**A** - photo of burial 35 before excavation, **B** - plan of the rubblework; **C** - section of the burial; D - finds; E - canine skeleton position in the grave (by Goryunova et al., 2004, modified).

## Figure S9. Ulyarba site, burial 35.

A - plan of the burial; B - grave goods (by Goryunova et al., 2004, modified).

## Figure S10. Ulyarba site, burial 36.

A - plan and profile of the burial structure, B - plan of the burial, C - grave goods (by Goryunova et al., 2004, modified).

## Figure S11. **Burial at the Kamenka-1 site.**

**A** - rubblework, upper level; **B** - rubblework, lower level; **C** - burial: **1** - polished knife; **2** - chisel; **3** - retouched bifacial knife; **4** - stone arrowheads; **5** - bone arrowhead; **6** - antler bow plates; **7-10** - bone perforators; **11** - harpoon; **12** - bone tool blank; **13** - pottery vessel; **14** - accumulation of flakes, chips, cores, and bone awls; **15** - antler pendant (by Zaika, 2009, modified).

## Figure S12. Partially preserved burials at the Dolgoye Ozero site

(by Maksimenkov, 1964, modified).

## Figure S13. Early Neolithic burials at the Zarechnoye-1 site.

**A**: mound 2, burial 3, plan; **B**: mound 2, burial 4, section; **C**: artifacts from burial 3, **1** - bone tool, **2** - blades, **3** - stone axe; **D**: mound 2, burial 4, plan (by Zakh, 1985, modified).

## Figure S14. Neolithic burials at Firsovo-11.

Involved individuals are labeled in red (image by Kirill Kiryushin).

Figure S15. Grave goods from burial 14 at Firsovo-11.

**1-13** - skeleton 1; 1**4-20** - skeleton 2; **21** - burial 14. **1** - polished knife; **2** - polished axe; **3, 4** - retouched flakes; **5** - fragment of a polished knife; **6** - fragment of a tubular bone; **7-9, 11** - tool blanks; **10, 12, 13, 19, 20** - blades; **14, 15** - scrapers; **16** - retouched flake; **17** - serrated and notched tool; **18** - fragment of a bone tool (image by Kirill Kiryushin).

## Figure S16. Ornaments and costume details from burial 14 at Firsovo-11

(photo by Kirill Kiryushin).

## Figure S17. Ornaments from a horse chisel burial 17 and an arrowhead from burial 15 at Firsovo-11

(image by Kirill Kiryushin).

## Figure S18. Grave goods from burial 42 at Firsovo-11.

**1-9** - skeleton 1; **10-20** - skeleton 2. **1** - knife on a silicified shale; **2, 19, 20** - hatchets; **3** - bone slab; **4-17** - an arrowhead; **18** - a bracelet made of beaver incisors (image by Kirill Kiryushin).

## Figure S19. Chumysh-Perekat burial site.

**A**: plan of the cemetery, sequenced burials are marked in red; **B**:  burial 13 at Chumysh-Perekat; **C**: reconstruction of the costume; **D**: artifacts from the grave. **C, D: 1** - animal tooth, **2** - beaver incisor, **3** - knife, **4** - fish-shaped pendant, **5** - flake,  **6** - bone dagger tip, **12** - metacarpal bones of a bird; **7** - perforated bone, **8** - animal teeth, **9-11** - wood pieces, **13** - charcoal, **14** - stone pendant (image by Alexey Fribus and Sergey Grushin).

## Figure S20. Grave goods from the burials at Vas’kovo-4.

Attribution of the goods to specific burials is unknown. **1-3, 7-10** - arrowheads; **4-5** - pointed rods; **6** - knife base; **11-15** - zoomorphic figurines (no scale); **16** - bifacial knife; **17-21** - plate pendants; **22-24, 27-29, 31-33** - pendants made of animal teeth; **25-26, 30, 34** - other items made of bone and antler (by Borodkin 1976).

## Figure S21. Bolshoy Mys burial ground.

The burials containing sequenced individuals are labeled in red. **A** - approximate position of the burial destroyed in 1954 during the construction of a dugout building (an individual from this burial was sequenced) (image by Yurii Kiryushin).

## Figure S22. Burial 2 at Itkul’ (Bolshoy Mys).

**1-11** - grave goods: **1-9** - pendants; **10** - stone hatchet; **11** - grinding block (image by Yurii Kiryushin).

## Figure S23. Burials 3, 12, and 8 at Itkul’ (Bolshoy Mys).

**A** - burial 3: **1-2** - teeth of beaver and badger; **3** - quartzite flake; **B** - burial 12; **C** - burial 8: **1-3** - beaver’s and badger’s incisors; **4** - pendant; **5** - gypsum desert roses; **6** - a fragment of a fishing hook; **7** - perforated animal teeth (image by Yurii Kiryushin).

## Figure S24. Burial 17 at Itkul’ (Bolshoy Mys).

**A** - burial 17; **B** - grave goods: **1** - needle case; **2** - nacre clothing appliques; **3-4** - pendants (image by Yurii Kiryushin).

## Figure S25. Burial 5 at Kostenkova Izbushka.

**A** - burial 5 (**1** - arrowhead; **2** - pottery vessel); **B** - position of the arrowhead in the burial; **C** - pottery vessel (image by Yurii Kiryushin).

## Figure S26. Burial 4 at Ust’-Isha.

**1** - stone dagger; **2** - bone dagger; **3** - pendants (image by Yurii Kiryushin).

## Figure S27. Burials 6 and 7 at Ust’-Isha.

**A** - burial 6 (**1** - point of a javelin); **B** - burial 7 (image by Yurii Kiryushin).

## Figure S28. Burial 8 at Ust’-Isha.

**1** - arrowheads; **2** - stone dagger; **3** - bone daggers; **4** - nacre beads and pendants made of stone and bone; **5** - beaver’s incisors; **6** - eight-shaped pendants; **7** - shell (image by Yurii Kiryushin).

## Figure S29. Burial 10 or 11 at Ust’-Isha

(image by Yurii Kiryushin).

## Figure S30. Burial 267 at Firsovo-14.

**1** - antler spatula; **2** - grave goods (image by Yurii Kiryushin).

## Figure S31. Neolithic sites in the Upper Ob and Kuznetsk Depression.

1 - Razdum'ye-1; 2 - Tuzovskie-Bugry-1 (Vasino-5); 3 - Firsovo-11; 4 - Zarechnoye-1; 5 - Chumysh-Perekat-1; 6 - Itkul (Bolshoy-Mys) and Kostenkova-Izbushka; 7 - Vas'kovo-4; 8 - Lebedi-2; 9 - Ust'-Isha; 10 - Solontcy-5.

## Figure S32. Burial 1 at Solontsy-5.

**A** - burial 1 (dark color - child bones, light color - adult bones): **a** - flakes; **b** - musk deer tusks; **c** - bone appliques; **d** - teeth appliques; **1** - fishing rods; **2** - grinding stone; **3** - harpoon tips; **4** - chisels; **5** - knives; **6** - slotted dagger; **7** - “small flat iron” with a handle; **8-9** - shells; **10** - bone hooked rods; **11** - bones; **12** - scraper; **13** - fabricator; **14** - arrowheads; **15** - scraper made from a flake; **16** - stone; **B** - bone tools; **C** - various grave goods; **D** - ornaments, pendants, and appliques; **E** - bifacial axe and stone plates; **F** - slotted bone dagger; **G** - stone dagger; **H** - stone implements and arrowheads; **I** - photo of stone tools; **J** - photo of stone fishing rods (by Kungurova 2003, modified).

## Figure S33. Burial 7 at Solontsy-5.

**1** - arrowhead; **2** - bifacial dagger; **3** - musk deer tusks; **4** - appliques; **5** - animal teeth; **6** - ocher spot (by Kungurova 2005, modified).

## Figure S34. Tuzovskiye Bugry-1 burial ground.

**A** - a plan of the cemetery, sequenced individuals are labeled in red; **B** - burial 33: **1** - tusks of musk deer, **2** - Corbicula shells, **3** - Dentalium shells, **4** - necklace with 18 small animal incisors, **5** - necklace with 6 small animal incisors, **6** - necklace with 25 marmot incisors, **7** - skull fragments; **C** - grave goods from burial 33: **1** - mollusk shells, **2** - tusks of musk deer, **3** - pendant from the upper fang of a badger and small incisors of a musk deer (image by Kirill Kiryushin).

## Figure S35. Burial 7 at the Razdumye-1 site

(by Umanskiy 1987, modified).

## Figure S36. *Neolithic sites in the Middle Irtysh region*.

*1 - Omsk; 2 - Ostrov-2; 3 - Chernoozerye-1; 4 - Borovyanka-17; 5 - Okunevo; 6 - Korchugan-1; 7 - Protoka.*

## Figure S37. The Korchugan-1a cemetery

(*based on Marchenko, 2009*).

## Figure S38. Burial 3 at the Korchugan-1a burial ground.

**1** - stone axe; **2** - flake (by Molodin et al., 1999, modified).

## Figure S39. Burial 7 at the Korchugan-1a burial ground.

**1** - necklace; **2** - rodent incisors (by Molodin et al., 1999, modified).

## Figure S40. The Protoka cemetery.

**A** - Neolithic burials covered by mound 5 (**1** - ceramics; **2** - pendants; **3** - adze; **4** - arrowhead (bone); **5** - arrowhead (stone); **7** - abrasive; **8** - core, **9** - vessel, **10** - a jaw of a dog, **11** - burial number; **12** - bones of the lower horizon; **a** - a vessel; **b** - knife-like plate; **h** - core); **B** - burial 3 (**1 -** chisel; **2** - pendants; **3** - arrowheads (bone); bones of the child's skeleton are cross-hatched); **C** - grave goods from burial 3 (**1,2** - arrowheads; **3** - chisel; **4** - pottery sherd) (by Polos’mak et al., 1989, modified).

## Figure S41. Burial 4 at Borovyanka-17

(image by Olga Sherstobitova).

## Figure S42. Burial 36C at Borovyanka-17

(image by Olga Sherstobitova).

## Figure S43. Burial 42 at Borovyanka-17

(image by Olga Sherstobitova).

## Figure S44. Burial 66 at Borovyanka-17

(image by Olga Sherstobitova).

## Figure S45. Burial 83 at Borovyanka-17.

Sequenced skulls D and F are labeled in red (image by Olga Sherstobitova). Skeletons are labelled by numbers, and skulls by letters.

## Figure S46. Okunevo site complex, burial 47B. Grave goods

(by Matyuschenko, Polevodov 1994, modified).

## Figure S47. Okunevo site complex, burial 59.

A plan of the burial and the grave goods (by Matyuschenko, Polevodov 1994, modified).

## Figure S48. Okunevo site complex, burial 62.

A plan of the burial and the grave goods (by Matyuschenko, Polevodov 1994, modified).

## Figure S49. Okunevo site complex, burial 65.

A plan of the burial and the grave goods (by Matyuschenko, Polevodov 1994, modified).

## Figure S50. Okunevo site complex, burial 69.

**A** - plan of the burial and **B** - grave goods (by Matyuschenko, Polevodov 1994, modified).

## Figure S51. Okunevo site complex, burial 79.

A plan of the burial and the grave goods (by Matyuschenko, Polevodov 1994, modified).

## Figure S52. Okunevo site complex, burial 168.

A stone artifact found in the grave pit (by Matyuschenko, Polevodov 1994, modified).

## Figure S53. Okunevo, burial 172. Grave goods

(by Matyuschenko, Polevodov 1994, modified).

## Figure S54. Okunevo site complex, burial 189.

A plan of the burial and the grave goods (by Matyuschenko, Polevodov 1994, modified).

## Figure S55. Okunevo site complex, burial 222.

A plan of the burial (by Matyuschenko, Polevodov 1994, modified).

## Figure S56. The Eneolithic burial at the Gladunino-3 site.

**A** - burial; **B** - stone mace; **C** - pottery sherd (by Shilov and Maslyuzhenko 2002, modified).

## Figure S57. Map with position of caves Kulmetovskiy-Grot and Kamen’-Dozhdevoy.

## Figure S58. Burials 1 and 2 in the Kamen’-Dozhdevoy cave.

**A** - excavation plan; **B** - burial 1; **1, 2** - flakes; **3** - pottery sherds; **4** - scrappers; **5** - perforated animal teeth (by Serikov, 1993, modified).

## Figure S59. Grave goods from burial 1 in the Kamen’-Dozhdevoy cave.

**1** - pottery sherd; **2** - flake; **3** - scrapper; **4, 5** - moose incisors (by Serikov, 1993).

## Figure S60. Burial B at the Chekalino-4 site

(image provided by Aleksandr Khokhlov, based on Mamonov, 1995).

## Figure S61. Labazy burial mounds.

**A** - plan of mound 4 (the burials that contained sequenced individuals are labeled in red), **B** - burial 2, **C** - burial 3 (image provided by Aleksandr Khokhlov, based on Kuptsova et al., 2019).

## Figure S62. Burial 12 at the Lebyazhinka-5 site

(image provided by Aleksandr Khokhlov).

**Figure S63.** **Eneolithic burials 102 and 118 at the Murzikha-2 site, & Grave goods from Eneolithic burials at the Murzikha-2 site.**

***Top - Eneolithic burials 102 and 118 at the Murzikha-2 site*** ***A*** *- burial 102;* ***B*** *- grave goods from burial 102;* ***C*** *- burial 118;* ***D*** *- limestone phallus sculpture from burial 118 (by Chizhevskiy, 2008, modified)**.* ***Bottom - Grave goods from Eneolithic burials at the Murzikha-2 site.*** ***A*** *- a dagger and stone axes from burial 128;* ***B*** *- harpoon from burial 104;* ***C*** *- antler arrowhead from burial 131;* ***D*** *- antler model of bird’s head from burial 104;* ***E*** *- pottery vessel from grave 124 (by Chizhevskiy, 2008, modified).*

## Figure S64. Burial 1 at the Maksimovka-1 site

(by Tsibin and Shalapinin, 2018, modified).

## Figure S65. Burial 1 at the Maksimovka-1 site. Grave goods

(by Tsibin and Shalapinin, 2018).

## Figure S66. Pit-Comb Lyalovo burial site at Sakhtysh-2a.

**1** - male burials; **2** - female burials; **3** - child’s burial; **4** - unidentified burial; **5** - grave rows. Sequenced individuals are marked by red arrows (by Kostyleva and Utkin 2010, modified).

## Figure S67. Pit-Comb Lyalovo burials at the Sakhtysh-2 site.

**A** - burial 19; **B** - burial 20 (by Kostyleva and Utkin 2010, modified).

## Figure S68. Burial 22 at Sakhtysh-2a.

**1** - skeleton; **2, 3** - daggers; **4** - harpoon; **5** - pendant (**2-5** - tools made of bone) (by Kostyleva and Utkin 2010, modified).

## Figure S69. Pit-Comb Lyalovo burials at the Sakhtysh-2a site.

**A** - burial 40; **B** - bone tool (kochedyk) from burial 40; **C** - burial 42 (by Kostyleva and Utkin 2010, modified).

## Figure S70. Pit-Comb Lyalovo burials at the Sakhtysh-2a site.

**1** - burial 43; **2** - burial 61; **3, 4** - bone tools from burial 61 (by Kostyleva and Utkin 2010, modified).

## Figure S71. Volosovo burials at the Sakhtysh-2a site.

**1** - burials with sequenced individuals; **2** - other burials; **3** - ritual (?) pits; **4** - ritual (?) construction; **5** - hoards (by Kostyleva and Utkin 2010, modified).

Figure S72. Burial 8 from the Sakhtysh-1 site

(**A**) and burial 11 from the Sakhtysh-2a site (**B**) (by Kostyleva and Utkin 2010, modified).

## Figure S73. Burials 10 at Sakhtysh-2a

(**A**) and 12 at Sakhtysh-2 (**B**), and amber buttons and pendants from burial 12 (by Kostyleva and Utkin 2010, modified).

Figure S74. Burials 9 and 13a/b at the Sakhtysh-2a site.

**A** - burial 9; **B** - amber pendants from burial 9; **C** - burials 13a and 13b; **D** - pendants made of teeth and amber from burials 13a and 13b (by Kostyleva and Utkin 2010, modified).

## Figure S75. Burials 33, 34, 39 at the Sakhtysh-2a site.

**A** - burial 34; **B** - pendants made of stone and teeth from burial 34; **C** - burial 33; **D** - burial 39; **E** - amber buttons from burial 39 (by Kostyleva and Utkin 2010, modified).

## Figure S76. Burials 46, 54, 56 at the Sakhtysh-2a site.

**A** - burial 46; **B** - stone beads from burial 46; **C** - burial 56; **D** - pendants made of stone and teeth from burial 56; **E** - burial 54; **F**- pendants made of stone and teeth from burial 54 (by Kostyleva and Utkin 2010, modified).

## Figure S77. Burial 58 at the Sakhtysh-2a site.

**A** - burial 58; **B** - anthropomorphic figurine pendant from burial 58 (by Kostyleva and Utkin 2010, modified).

## Figure S78. The burial ground at Rostovka (Omsk, Russia), Seima-Turbino burials.

## Figure S79. The burial ground at Rostovka. The ADMIXTURE results for the sequenced Seima-Turbino burials.

## Figure S80. Rostovka, burial 5.

**A** - plan of the burial, **B** - grave goods: **1** - spearhead, **2, 3** - jade flakes, **4**, **5** - arrowheads; **C** - cluster of finds near burial C (by Matyuschenko & Sinitsyna 1988, modified).

## Figure S81. Rostovka, burial 7

(by Matyuschenko & Sinitsyna 1988, modified).

## Figure S82. Rostovka, burial 8.

**A** - plan of the burial: **I-IV** - skeletons in the burial; **B** - grave goods: **1-9** - arrowheads, **10-11** - blades, **14** - awl, 15 - knife, **16** - bone handle, **17** and **19** - spearheads, **18** - socket axe (celt) (by Matyuschenko & Sinitsyna 1988, modified).

## Figure S83. Rostovka, burial 24.

**A** - plan of the burial; **B** - grave goods: **1** - arrowhead, **2** - a fragment of a casting mold, **3** - chisel, **4** - knife (by Matyuschenko & Sinitsyna 1988, modified).

## Figure S84. Cluster of finds near burial 24.

Two spearheads and a socketed axe (celt) (by Matyuschenko & Sinitsyna 1988, modified).

## Figure S85. Rostovka, burial 28

(by Matyuschenko & Sinitsyna 1988, modified).

## Figure S86. Rostovka, burial 33.

**A** - plan of the burial; **B** - grave goods: **1** - spearhead, **2, 3** - golden rings, **4** - grinding stones, **5, 7** - bone perforator, **6, 8** - arrowheads, **9** - dagger handle (by Matyuschenko & Sinitsyna 1988, modified).

## Figure S87. Cemetery Satyga-16 at the Satyga-16a occupation site.

**1** - uprootings; **2** - calcinated bones; **3** - postholes; **4** - ocher; **5** - arrowheads. The sequenced burials are labeled in red (image provided by Evgeniy Besprozvanny).

## Figure S88. Burial 6 at the Satyga-16 site.

**A** - burial; **B** - grave goods (image provided by Evgeniy Besprozvanny).

## Figure S89. Burial 17 at the Satyga-16 site.

**A** - burial; **B** - a section of the grave pit; **C** - grave goods (image provided by Evgeniy Besprozvanny).

## Figure S90. A: anthropomorphic figurines in the Bronze Age:

1 - Chernoozerye-1, 2 - Borovyanka-17, 3 - Zavyalovo-1A, 4 - Krokhalevka 13, 5 - Saigatino-6, 6 - Novy-Napas, 7 - Chudskaya-Gora, 8 - Rostovka, 9 - Bor-Lyonva, 10 - Galich treasure. **B**: antrhopomorhic figurine found at Chernoozerye-1 (image of the artifact is provided by Zhanna Trufanova (Trufanova 2003), map is based on Stefanov, 2004).

## Figure S91. Anzhevsky ensemble.

**A:** layout of burials at the archaeological sites Nefteprovod-1 and Nefteprovod-2. **B:** layout of burials of the lower group at Nefteprovod-2. **C:** masonry over burial 2 at Nefteprovod-2. **D:** skeleton in burial 2 at Nefteprovod-2. **E:** bronze knife in burial 2 at Nefteprovod-2 (image by Anton Vybornov).

## Figure S92. Anzhevsky ensemble.

**A:** rubblework over burial 14 at Nefteprovod-1. **B:** skeleton in burial 14 at Nefteprovod-1. **C:** longitudinal section of burial 14 at Nefteprovod-1 (image by Anton Vybornov).

## Figure S93. Cross-validation error for our ADMIXTURE run at each K*.*

## Figure S94. Statistics of the form f4(Ethiopia_4500BP.SG, Target, Kolyma_M_10.1kya/Dzhilinda1_M_N_8.4kya, Ust_Kyakhta_14kya/Cisbaikal_LNBA).

Central Siberian populations from the Yenisei Basin (including Kets and South Siberian Turks) are highlighted in brown, while Arctic North American and Asian populations on either side of the Bering Straits populations are highlighted in blue. Despite the similarity of the APS-rich populations in this comparison (all being admixtures between APS ancestry and East Asian ancestry), present-day groups of the Bering Straits are generally closer to groups with “Route 2” APS ancestry (i.e., Kolyma_M_10.1kya → Dzhilinda1_8.4kya → Syalakh-Belkachi → Yakutia_LNBA), while Central Siberian populations of the Yenisei Basin are always closer to Cisbaikal_LNBA and Ust_Kyakhta_14kya. Bering Straits populations that are heavily European-admixed (Aleut and Yukagir_forest) are colored dark blue, while Samoyedic populations (Enets, Selkup, and Nganasan) are colored violet.

## Figure S95. Statistics of the form f4(Ethiopia_4500BP.SG, Target, China_Paleolithic, Yana_UP.SG) on the y-axis, plotted against f4(Ethiopia_4500BP.SG, Target, X, Yana_UP.SG).

Where X are ancient Native Americans (USR1.SG, Peru_Laramate_900BP) and wide range of ancient populations from Beringia (Saqqaq.SG, OldBeringSea_Ekven, OldBeringSea_Uelen, USA_AK_PaleoAleut, USA_AK_NeoAleut, USA_AK_Ancient_Athabaskan_1100BP, and Russia_Magadan_BA). Notice that ancient populations in the second route: Dzhilinda1_M_N_8.4kya, Kolyma_M_10.1kya, Syalakh-Belkachi, and Yakutia_LNBA are consistently shifted to the left of the curve formed by other NEAHG populations as they share more drift with ancient Beringian populations than similar populations on the NEAHG cline. We removed the following group labels in the NEAHG cline from this analysis, due to their being single individuals with low coverage (< 0.1):, resulting in wide confidence intervals and high variability in the F4-statistics: Russia_AngaraRiver_EN/ANG001, Russia_Okunevsky_Eneolithic_o1/I6950, Russia_KuangaRiver_N_1.SG/brn001.SG, Russia_MiddleAngara_N_possible/I10900_d, Russia_Zhuravlevo_N_possible/I25462, Russia_unknown_N/I7616, Russia_Popovo_HG/Popovo2, Russia_Omskay_MN/I11449, and Russia_Tyumen_LN_o1/I1958.

## Figure S96 | Statistics of the form F4(Ethiopia_4500BP.SG, X, China_NEastAsia_Inland_EN, China_AmurRiver_Mesolithic 14K), plotted against F4(Ethiopia_4500BP.SG, X, China_Paleolithic, MA1_HG.SG) (top) and F4(Ethiopia_4500BP.SG, X, China_Paleolithic, Peru_Laramate_900BP) (bottom), where X are ancient populations in Northeast Asia and Siberia.

These statistics indicate that substructure exists within the East Asian ancestry of admixed groups in Northeast East Asia and Siberia, with differentiation between an Inland East Asian-related source represented by the *Yumin* hunter-gatherer under the population label *China_NEastAsia_Inland_EN*, and an Amur-River-related source represented by *China_AmurRiver_Mesolithic_14K*. Populations from the Amur River region throughout its history share high affinity with *China_AmurRiver_Mesolithic_14K*, while populations on the Mongolian Plateau and the Baikal area share more affinity with the Yumin hunter-gatherer. A geographic exception to this rule is the individual *KhatystyrCave_M_10.2kya*, which is extremely Amur-River-related despite occurring northeast of the Baikal region in Southern Yakutia. Interestingly, affinity to *China_NEastAsia_Inland_EN* also increases in agriculturalist populations of the Yellow River Valley as one moves west, suggesting that this population was more widely distributed. Groups rich in APS ancestry, such as *Dzhilinda1_M_N_8.4kya, Kolyma_M_10.1kya*, and *Syalakh-Belkachi*, tend to be almost equally related to both groups, with the exception of *Cisbaikal_LNBA*, which is strongly Inland Northeast Asian-related.

## Figure S97. F4-statistics showing that ancient Athabaskans are not more related to Saqqaq.SG than to other Asian APS-rich populations when compared to Native Americans.

The X-axis displays the results of the F4-statistic F4(Taiwan_Hanben_IA, Peru_Laramate_900BP, Target, Ethiopia_Mota.SG), with points further to the right more related to East Asians (due to presumably greater APS ancestry), and points further to the left more related to Native Americans (due to lack of APS ancestry). The Y-axis displays the results of the F4-statistic F4(Greenland_Saqqaq.SG, X, Target, Ethiopia_Mota.SG), where X is another population rich in APS ancestry from the Asian side of the Bering Straits (including Cisbaikal_LNBA, Dzhilinda_M_N_8.4kya, Khaiyrgas_16.7kya, KolymA_M_10.1kya, Russia_Magadan_BA, Syalakh-Belkachi, Ust_Kyakhta_14kya, and Yakutia_LNBA). Points further to the top are related to Greenland_Saqqaq.SG, and points further to downwards are more related to the other APS population. Native Americans from south of the Arctic Tundra (purple points) are approximately equally related to Saqqaq.SG and other APS-rich populations, with their points hovering around zero on the y-axis, but Paleo-Ekimos, Neo-Eskimos, Paleo-Aleuts and Neo-Aleuts are significantly shifted in the direction of Saqqaq.SG and do not overlap with Native Americans in this statistic. Like non-Arctic Native Americans, Ancient Athabaskans are also approximately equally related to Saqqaq.SG and other APS-rich populations.

Figure S98. F4-statistics showing that ancient Athabaskans are not more related to Saqqaq.SG than to other Asian APS-rich populations when compared to Native Americans.

This diagram shows a similar analysis as that shown in Figure S101, with the exception that the X-axis displays the results of the F4-statistic F4(Taiwan_Hanben_IA, USA_AK_USR1.SG, Target, Ethiopia_Mota.SG), instead, where USR1.SG is a basal Native American individual. As before, points further to the right more related to East Asians (due to presumably greater APS ancestry), and points further to the left more related to Native Americans (due to lack of APS ancestry). The Y-axis displays the results of the F4-statistic F4(Greenland_Saqqaq.SG, X, Target, Ethiopia_Mota.SG), where X is another population rich in APS ancestry from the Asian side of the Bering. As before, Native Americans from south of the Arctic Tundra (purple points) are approximately equally related to Saqqaq.SG and other APS-rich populations, but Paleo-Ekimos, Neo-Eskimos, Paleo-Aleuts and Neo-Aleuts are significantly shifted in the direction of Saqqaq.SG. Like non-Arctic Native Americans, Ancient Athabaskans are also approximately equally related to Saqqaq.SG and other APS-rich populations.

**Figure S99. *qpAdm* results for Admixed Inner Asian populations,** **with estimated proportions of *Cisbaikal_LNBA* ancestry.**

Note that no passing model here contains both *Cisbaikal_LNBA* and an *ANE*-rich source such as *Altai_N_7.5-6kya* and *Tyumen_N_HG*, which makes us caution against taking the estimated levels of *Cisbaikal_LNBA* ancestry at face value.

References: [*China_Paleolithic, Iran_GanjDareh_N, Morocco_Iberomaurusian, Altai_N_9kya, Russia_AfontovaGora3, Italy_South_HG_Ostuni, Turkey_Boncuklu_N, Peru_Laramate_900BP, China_SEastAsia_Island_EN, China_NEastAsia_Inland_EN, Syalakh-Belkachi, USA_AK_PaleoAleut, USA_AK_NeoAleut, Cisbaikal_LNBA*]

Sources: [*Russia_Srubnaya, Hungary_Koros_EN, EHG, Tyumen_N, Altai_N_7.5-6kya, Yakutia_LNBA, Mongolia_N, China_YR_MN, Germany_EN_LBK, BMAC, Cisbaikal_LNBA*]

**Figure S100. *qpAdm* results for Admixed Inner Asian populations**

One orange dot indicates that all East Asian ancestry can be modeled as deriving from *Yakutia_LNBA*; two orange dots indicate that all passing models include *Yakutia_LNBA* among the sources. A grey dot indicates that all passing models include *Cisbaikal_LNBA* in the sources when *Cisbaikal_LNBA* was placed in the references, with the option of being rotated into the sources. All models displayed are of the simplest model that passes with p>0.01; if multiple models pass, the model with the highest p-value is plotted. Populations without models passing at the p>0.01 level are indicated with an asterisk; in such cases, the model with the highest p-value is plotted.

References: [*China_Paleolithic, Iran_GanjDareh_N, Morocco_Iberomaurusian, Altai_N_9kya, Russia_AfontovaGora3, Italy_South_HG_Ostuni, Turkey_Boncuklu_N, Peru_Laramate_900BP, China_SEastAsia_Island_EN, China_NEastAsia_Inland_EN, Syalakh-Belkachi, USA_AK_PaleoAleut, USA_AK_NeoAleut*]

Sources: [*Russia_Srubnaya, Hungary_Koros_EN, EHG, Tyumen_N, Altai_N_7.5-6kya, Yakutia_LNBA, Mongolia_N, China_YR_MN, Germany_EN_LBK, BMAC*].

## Figure S101 | PCA of F4-Statistics of AIEA populations.

PC2 plotted against PC1 and PC3 for a PCA over F4-statistics of the form F4(Ethiopia_4500BP.SG, *AIEA*, AG3, EA) where EA is any East Asian population from the following set: [*China_AmurRiver_N, Mongolia_N_North, Transbaikal_N_HG, Cisbaikal_LNBA, Yakutia_LNBA*]. PC1 highlights proportions of West and East Eurasian ancestry, while PC2 highlights similarity to *Yakutia_LNBA*. PC3 highlights similarity to *Cisbaikal_LNBA*; 3 ancient individuals from the Karasuk period, and present-day populations from the Yenisei Basin, including South Siberian Turkics, Kets and Selkups are shifted in the direction indicated by the biplot to involve increased shared drift with *Cisbaikal_LNBA* out of East Asian populations. This is investigated in further detail in SI VI.C.iii. The population codes are: ATN, Altaian; ATN_C, Altaian_Chelkan; BSK, Bashkir; BSM, Besermyan; BRY, Buryat; XNB_AR, China_AR_Xianbei_IA; CVS, Chuvash; DUR, Daur; DGN, Dolgan; DGX, Dongxiang; ENT, Enets; EST, Estonian; EVN, Even; EVN_E, Evenk_FarEast; EVN_T, Evenk_Transbaikal; FIN.SG, FIN.SG; LVL, Finland_Levanluhta; SAM, Finland_Saami_Modern.SG; FIN, Finnish; HZN, Hezhen; KLM, Kalmyk; KKP, Karakalpak; KRL, Karelian; KZK, Kazakh; KZK_C, Kazakh_China; BRL, Kazakhstan_Berel_IA; SARM_C, Kazakhstan_CaspianSteppe_Sarmatian; SARM_C.SG, Kazakhstan_CaspianSteppe_Sarmatian.SG; SAKA_K, Kazakhstan_CentralKazakhSteppe_Saka; SARM_K, Kazakhstan_CentralKazakhSteppe_Sarmatian.SG; KRK, Kazakhstan_Karakhanid.SG; KLK_1, Kazakhstan_Karluk_1.SG; KLK_2, Kazakhstan_Karluk_2.SG; KMK, Kazakhstan_Kimak.SG; KPC_1, Kazakhstan_Kipchak1.SG; KPC_2, Kazakhstan_Kipchak2.SG; SAKA_TS, Kazakhstan_Kyrgystan_TianShan_Saka; BRL_P, Kazakhstan_Pazyryk_Berel; TSM, Kazakhstan_Tasmola; SARM_W, Kazakhstan_WesternKazakhSteppe_Sarmatian; KET, Ket; KKS, Khakass; KKS_K, Khakass_Kachin; KMG, Khamnegan; KHT, Khanty; KOM, Komi_Zyrian; KRG_C, Kyrgyz_China; KRG_K, Kyrgyz_Kyrgyzstan; KRG_T, Kyrgyz_Tajikistan; TUR, Kyrgyzstan_Turk.SG; MNS, Mansi; MRI, Mari.SG; SCY, Moldova_Scythian; MGL, Mongol; MGA, Mongola; XNB_M, Mongolia_IA_Xianbei; MDV, Mordovian; NNI, Nanai; NGD, Negidal; NGS, Nganasan; NVH, Nivh; NGI_A, Nogai_Astrakhan; NGI_K, Nogai_Karachay_Cherkessia; NGI_S, Nogai_Stavropol; ORQ, Oroqen; ADB, Russia_Aldy_Bel; BLS, Russia_Bolshoy; MHE_1, Russia_EarlyMedieval_Heshui_Mohe_1; MHE_2, Russia_EarlyMedieval_Heshui_Mohe_2; SARM_S, Russia_EarlySarmatian_SouthernUrals.SG; KRS_o1, Russia_Karasuk_o1.SG; KRS_o, Russia_Karasuk_oRISE.SG; KRS, Russia_Karasuk.SG; SARM_L, Russia_LateSarmatian.SG; SARM_S.SG, Russia_MiddleSarmatian_SouthernUrals.SG; SARM, Russia_Sarmatian; SARM.SG, Russia_Sarmatian.SG; TGR, Russia_Tagar.SG; SAM.DG, Saami.DG; SKP, Selkup; SHR_K, Shor_Khakassia; SHR_M, Shor_Mountain; TTR_A, Tatar_Astrakhan; TTR_I, Tatar_Irtysh_Barabinsk.SG; TTR_K, Tatar_Kazan; TTR_M, Tatar_Mishar; TTR_S, Tatar_Siberian; TTR_Z, Tatar_Siberian_Zabolotniye; TTR_T, Tatar_Tomsk.SG; TTR_V, Tatar_Volga.SG; TDZ, Todzin; TFL, Tofalar; TBL, Tubalar; TKM, Turkmen; TVN, Tuvinian; UDM, Udmurt; SCY_U, Ukraine_Scythian; ULC, Ulchi; UYG, Uyghur; UZB, Uzbek; VPS, Veps; XIB, Xibo; YKT, Yakut; YKG_F, Yukagir_Forest; YKG_T, Yukagir_Tundra; KNY.SG, Russia_Yenisei_Krasnoyarsk_LBA.SG; KNY_o1.SG, Russia_Yenisei_Krasnoyarsk_LBA_o1.SG.

# **Tables in Supplementary Information**

## Table S1 | Most common geographic terms in main text

## Table S2. Relative and absolute chronology of the sequenced Rostovka burials.

## Table S3. Individuals from Northeast Siberia grouped in grouping procedure.

## Table S4. Individuals from the Cisbaikal region grouped in grouping procedure.

## Table S5. Tranches for qpAdm procedure.

## Table S6. Passing 2-way model for Khaiyrgas.

## Table S7. Passing 2-way models for Ust_Kyakhta_14kya.

## Table S8 & S9. Passing 2-way models for KhatystyrCave_M_10.2kya/*Yakutia_Mesolithic* & *Kolyma_M_10.1kya*.

## Table S10 & S11. Passing 2-way models for *Dzhilinda1_M_N_8.4kya* & Altai_N_9kya.

## Table S12 & S13. Passing models for the populations in Tranche 5.

## Table S14 & S15. Passing models, or the best failing model, for the populations in Tranche 6.

## Table S16 & S17. Best failing models for *Cisbaikal_LNBA* as a member of tranche 5 and 4 respectively.

## Table S18 & S19. Passing 2-way models for *Cisbaikal_LNBA*.

## Table S20. Passing models for the populations in Tranche 3, set B, with *Cisbaikal_LNBA*.

## Table S21 & S22. Passing models for the populations in Tranche 4, with *Cisbaikal_LNBA* in tranche 2.

## Table S23 & S24. Passing models for the populations in Tranche 5 with the addition of *Cisbaikal_LNBA* as a new member of this tranche, as well as *Transbaikal_EMN_9-8kya* in tranche 3, *Altai_N_9kya* in tranche 4.

## Table S25. Passing models for Peru_Laramate_900BP modeled with references/sources used for tranche 1.

## Table S26. Passing models for the Saqqaq individual with all populations from Siberia and Northeast Asia in the references.

## Table S27. Passing models for ancient Beringians and Arctic North Americans with all populations from Siberia and Northeast Asia in the references.

## Table S28. Passing models for ancient Beringians and Arctic North Americans with all populations from Siberia and Northeast Asia in the references, plus NNA_10000-4000BP.SG in the sources and SNA_12000-400BP.SG in the references.

## Table S29. Passing models for ancient Athabaskans with all populations from Siberia and Northeast Asia in the references.

## Table S30. Passing models for ancient Athabaskans with all populations from Siberia and Northeast Asia in the references, plus NNA_10000-4000BP.SG in the sources and SNA_12000-400BP.SG in the references.

## Table S31. Passing models for *Russia_MiddleAngara_EBA* modeled with references/sources used for Siberians and ancient Beringians in section VI.B.i (i.e. Tranche 1 + 2 + 3 + 4 + 5 + 6).

## Table S32. Passing models for *Russia_Yenisei_Krasnoyarsk_o1.SG* & *Russia_Karasuk_o1.SG* modeled with references/sources used for *AIEA* populations in section VI.D.ii.

## Table S33. Passing models for *Russia_Tatarka_BA* modeled with with all populations from Siberia and Northeast Asia in the references.

## Table S34. Passing models for ST individuals with references used for AIEA populations in section VI.C.ii, and a subset of the sources used for AIEA populations in section VI.C.ii (dropping *BMAC* and *Germany_EN_LBK*).

## Table S35. Passing models for ST individuals with a modified list of the references used for AIEA populations in section VI.C.ii (adding *Altai_N_7.5-6kya,* *Tyumen_N* and *Yakutia_LNBA*), and modified list of the sources used for AIEA populations in section VI.C.ii (dropping *BMAC* and *Germany_EN_LBK*, and adding the Tatarka_BA population and 5 NEAHG sources from the Trans-Ural region).

# **References**

**For images, in order of appearance:**

- - - 1. Novgorodov, I. D., Kangalassy burial. Collection of scientific articles 3, 80-87 (Yakutia publishing house, 1960).
      2. Alekseev, A. N., Dyakonov, V. M., Solovyova, E. N., Nikolaev, E. N. & Boeskorov, G. G. The burial Ogonyok in the middle Lena River region: a new site of the Bel'kachi Culture. Bulletin of Archeology, Anthropology, and Ethnography 1 (56), 2071-0437-2022-56-1-6 (2022).
      3. Goryunova O. I. Ancient Burial Grounds of the Baikal Region (Neolithic-Bronze Age) (Irkutsk St. Univ. publ. house, 2002).

1. Goryunova, O. I., Novikov, A. G., Zyablin, L.P., Smotrova, V.I. Ancient burials of the Ulyarba burial sites on the Lake Baikal (Neolithic-Paleometal Age) (Inst. of Archaeol. and Ethnogr. SB RAS, 2004).
2. Privalikhin, V. I., Drozdov, N. I. & Makulov, V. I. in Archaeological research of the antiquities of the Lower Angara and adjacent territories (ed. Karnauhova, L. L.) 42-56 (2013).
3. Zaika, A. L. Neolithic burial in the mouth of the Kamenka River on the lower Angara. News of the Laboratory of Ancient Technologies 1 (7), 60-72 (2009).
4. Privalikhin, V. I. in Yeniseyskaya provintsiya (ed. Vdovin, A. S. et al). 4, 300-310 (2009).
5. Privalikhin, V. I. Sosnovy Mys–2 – a new multi-temporal burial ground of Northern Priangary. In Siberian Inter-museum Collection (ed. Paramonov, V. I.) 72–87 (1998).
6. Maksimenkov, G. A. The Burial Ground near the Dolgoye Lake in Kansk. Questions of Anthropology 18, 132-134 (1964).
7. Zakh, V. A. in Western Siberia in Antiquity and the Middle Ages (ed. Vasil’evsky, R. S.) 23-29 (Tumen St. Univ. publ. house, 1985).
8. Borodkin, Y. M. Works of visual art from the Neolithic burials of the Vaskovo burial ground. News of the Laboratory of Ancient Technologies 7, 99-109 (1976).
9. Kungurova, N. Yu. Materials from the Solontsy-5 Burial Ground (on the Issue of Ethnocultural Connections of the Bearers of the Kuznetsk-Altai Culture). Archeology, Ethnography and Anthropology of Eurasia 2 (14), 30-40 (2003).
10. Kungurova, N. Yu. Solontsy 5 Burial Ground. Culture of Neolithic Burials in Altai (Barnaul Law institute, 2005).
11. Umanskiy, A. P. Archaeological sites of the Razdumye tract. In Archaeological Research in Altai: Interuniversity Collection of Scientific Articles, 81–99 (Altai State University Press, Barnaul, 1987).
12. Molodin, V. I., Novikov, A. V. & Chikisheva, T. A. The Neolithic cemetery of Korchugan on the Middle Tara River. In Problems of the Neolithic and Eneolithic of the Southern Part of Western Siberia, 66–98 (Kuzbassvuzizdat, Kemerovo, 1999).
13. Polos'mak, N. V., Chikisheva, T. A. & Balueva, T. S. Neolithic cemeteries of Northern Baraba (Nauka, Siberian Branch, Novosibirsk, 1989).
14. Matyushchenko, V. I. & Polevodov, A. V. A complex of archaeological sites on the Tatarsky Uval near the village of Okunevo (Novosibirsk, 1994).
15. Shilov, S. N. & Maslyuzhenko, D. N. in Questions of the Archeology of the Urals (ed. Kovaleva, V. T. et al) 24, 165-191 (Ural St. Univ. publ. house, 2002).
16. Serikov, Yu. B. In Questions of the Archeology of the Urals (ed. Kosinskaya, L. L.) 21, 120–143 (Ural State University Publishing House, 1993).
17. Chizhevskiy, A. A. in Proceedings of the 2 (18) All-Russian Archaeological Congress in Suzdal (ed. Derevyanko, A. P. & Makarov, N. A.) 1, 367-37 (Archeology Institute publ. house, 2008).
18. Tsibin, V. A. & Shalapinin, A. A. The Eneolithic Burial of Maksimovka I flat Burial Ground from the Samara Trans-Volga Region. Bulletin of Volgograd State University. History. Regional studies. International relations 3 (24), jvolsu4.2019.3.1 (2019).
19. Kostyleva E. L. & Utkin A. V. Neo-Eneolithic burial grounds of the Upper Volga region and the Volga-Oka interfluve: Planigraphic and chronological structures (TAUS, 2010).
20. Matyushchenko, V. I. & Sinitsyna, G. V. The cemetery near the village of Rostovka near Omsk (Tomsk State University Press, Tomsk, 1988).
21. Stefanov, V. I. The Forgotten Find from the Chernoozer'e Cemetery. Russian Archaeology 4, 114-118 (2004).
